# Regioselective Aldol Reactions Directed by Charge and Orbital Effects in Cyclic Germyl Dienolates: Synthesis of Three Distinct Regio‐ and Stereoisomers

**DOI:** 10.1002/chem.71422

**Published:** 2026-07-17

**Authors:** Taishi Nojima, Akihito Konishi, Makoto Yasuda

**Affiliations:** ^1^ Department of Applied Chemistry Graduate School of Engineering The University of Osaka Suita Osaka Japan; ^2^ Innovative Catalysis Science Division Institute for Open and Transdisciplinary Research Initiatives (ICS‐OTRI) The University of Osaka Suita Osaka Japan; ^3^ Center for Future Innovation Graduate School of Engineering The University of Osaka Suita Osaka Japan

**Keywords:** aldol reaction, charge‐/orbital‐controlled selectivity, dienolate, germanium, regioselectivity

## Abstract

Regioselective aldol reactions of dienolates represent a valuable method for the construction of complex molecular scaffolds; however, controlling the reaction site of the dienolate remains a significant challenge. This study presents a method for regioselectivity switching in the aldol reactions of cyclic *Ge*‐dienolates, which are generated from the (4 + 1) cycloaddition of allenoates with Ge(II) salts. Through systematic tuning of the charge and orbital distributions of the *Ge*‐dienolates and their aldehyde reaction partners, regio‐ and stereoselective access to three distinct aldol adducts (*syn*‐*α*‐, *anti*‐*α*‐, and *anti*‐*γ*‐adducts) is achieved. The aldol reaction of *Ge*‐dienolates generally proceeds via an orbital‐controlled pathway to afford the *anti*‐*γ*‐adduct through a cyclic transition state. Activation of the aldehyde with a Lewis acid shifts the reaction to a charge‐controlled pathway, resulting in the *syn*‐*α*‐adduct via an acyclic transition state. Notably, coordination of a ligand or solvent to the Ge center of the cyclic *Ge*‐dienolates increases electron density, thereby favoring a charge‐controlled pathway, promoting selective *α*‐addition to yield the *anti*‐*α*‐adduct through a distinct cyclic transition state. The synthetic utility of these types of adducts is further demonstrated via gold‐catalyzed intramolecular C─H/C–Ge cross‐coupling, which enables the efficient synthesis of various fused‐ring systems.

## Graphical abstract

Charge and orbital control represent fundamental principles in organic chemistry. In this study, these principles are applied to the aldol reactions of a cyclic *Ge*‐dienolate. By systematically tuning the charge and orbital distributions of the *Ge*‐dienolates and their aldehyde reaction partners, regio‐ and stereoselective access to three distinct aldol adducts (*syn*‐*α*‐, *anti*‐*α*‐, and *anti*‐*γ*‐adducts) is achieved. The synthetic utility of these adducts is further demonstrated through selective C─Ge bond modification.

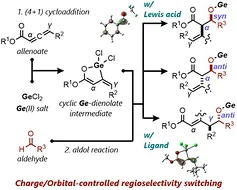

## 1 Introduction

Charge‐ and orbital‐controlled mechanisms are widely recognized as fundamental principles that govern organic reactions [1, 2, 3, 4]. According to the ‘Hard and Soft Acids and Bases’ (HSAB) theory, reactions involving highly polarized species proceed via charge‐controlled mechanisms at the sites with the highest charge density, while reactions involving less polarized species follow orbital‐controlled mechanisms at the sites with the highest orbital coefficient [5, 6, 7]. In reactions involving ambident nucleophiles, in which the distributions of electron density and orbital coefficient differ, the hardness or softness of the electrophile determines the regioselectivity. Enolates exemplify this phenomenon, that is, their negative charge is primarily localized on the oxygen atom, whereas the highest HOMO coefficient is located on the *α*‐carbon [8, 9]. As a result, their reactions with harder electrophiles, such as protons, alkyl tosylates [10], chlorosilanes [11, 12], or acid anhydrides [13], predominantly occur at the oxygen atom, producing enols, vinyl ethers, *Si*‐enolates, or enol acetates, respectively. Conversely, the use of softer electrophiles, such as alkyl iodides [10] or aldehydes [14], lead to alkylation or aldol‐type reactions at the *α*‐carbon atom (Figure 1a). Numerous studies have demonstrated that the regioselectivity of enolates can be rationalized in terms of charge and orbital effects.

**FIGURE 1:**
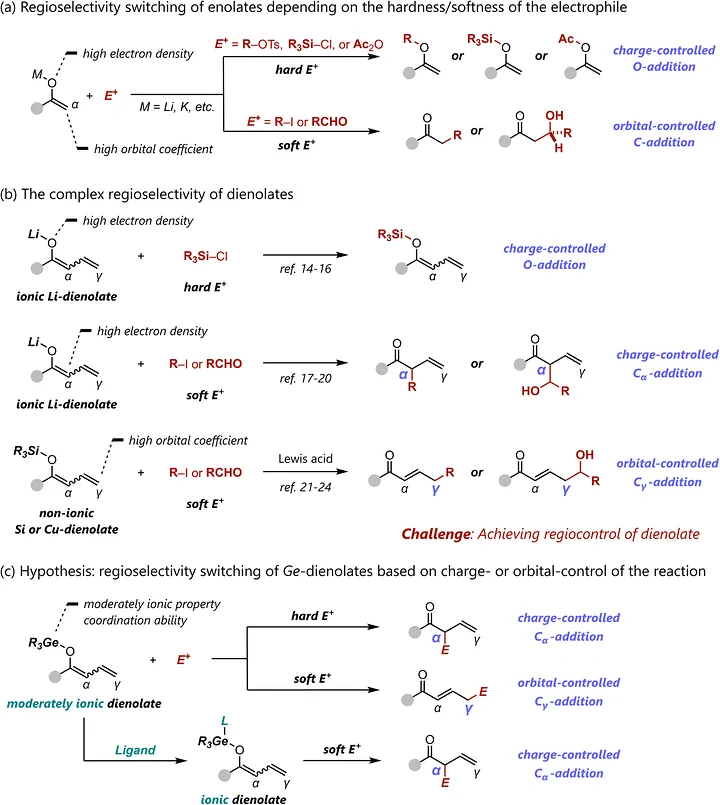
(a) Regioselectivity switching in the nucleophilic additions of enolates depending on the hardness or softness of the reaction partner. (b) The greater complexity of regioselectivity in the nucleophilic addition reactions of dienolates compared to that of enolates. (c) Hypothesis: Controllable regioselectivity switching of *Ge*‐dienolate species can be achieved via inducing a charge‐ or orbital‐controlled reaction pathway.

Dienolates are valuable nucleophiles that can directly yield *δ*‐hydroxy ketones, a key structural motif in macrolides, via vinylogous aldol reactions [15, 16, 17]. However, the regioselectivity of these aldol reactions is more complicated than those of conventional enolates, as nucleophilic addition can occur at the oxygen, the *α*‐carbon, or the *γ*‐carbon atoms [4, 18, 19, 20, 21]. As with conventional enolates, the reactions of dienolates with hard electrophiles such as chlorosilanes typically occur at the oxygen atom to produce the corresponding *Si*‐dienolates [22, 23, 24]. The regioselectivity between the *α*‐ and *γ*‐positions is primarily governed by the character of the metal counterion of the dienolate. Alkylations [25, 26] and aldol reactions [27, 28] involving more polar dienolates, such as *Li*‐dienolates, preferentially occur at the *α*‐position, where electron density is high. In contrast, reactions involving less polar *Si*‐ [29, 30] or *Cu*‐dienolates [31, 32] occur selectively at the *γ*‐position, where the HOMO coefficient is highest (Figure 1b) [33].

Several seminal studies [34, 35, 36, 37] have achieved regioselectivity in the addition reactions of dienolates by using bulky Lewis acids [38, 39] or exploiting the reversibility of *α*‐adducts via a retro‐aldol process [27, 28, 40, 41, 42, 43, 44, 45]. However, the influence of charge and orbital effects on regioselectivity in dienolates remains unexplored, despite the potential as a straightforward and effective strategy to address the inherent complexity of dienolates and provide significant value to synthetic chemistry.

Two key prerequisites are necessary to achieve regioselectivity switching of a dienolate via the induction of a charge‐ or orbital‐controlled reaction mechanism (Figure 1c). The first is a suitable balance between ionic and covalent character for the metal counterion of the dienolate. The second is the ability to tune the electron density of the dienolate via a response to external additives or solvents. Based on these considerations, *Ge*‐dienolates emerge as suitable nucleophiles [24, 46, 47, 48]. Germanium exhibits higher ionic character than silicon while retaining covalent properties, thus endowing *Ge*‐dienolates with inherent duality, which can be further modulated via coordination of external ligands to the Ge center, thus enabling a precise tuning of the electronic structure of the dienolate.

Recently, we have reported a mild method for the generation of various nucleophilic species using divalent germanium salts [45, 49, 50, 51, 52, 53, 54, 55, 56, 57]. For example, *Ge*‐dienolates **II** were generated from allenones **I** using GeCl_2_ (Figure 2a) [45]. The combination of the rigid cyclic structure of the *Ge*‐enolates with the moderate Lewis acidity of the Ge center [56] effectively controls the facial selectivity to achieve perfect diastereoselectivity. Furthermore, complete control of the regioselectivity could be attained via the use of a Lewis acid additive and the choice of solvent to afford either *syn*‐*α*‐**IV** in acetonitrile or *anti*‐*γ*‐**IV** in THF. However, the regioselectivity switching of **II** hinged on the reversible retro‐aldol nature of the *α*‐adducts, which constrained the practical utility of the methodology. To overcome this intrinsic limitation, we redesigned the reaction system by replacing the allenone precursors **I** with allenoates **1**, enabling the generation of a fundamentally different *Ge*‐dienolate (**2**) with tunable electronic properties (Figure 2b). The reaction of **2** with a soft aldehyde electrophile (**3**) proceeds under orbital control to produce the *γ*‐adduct (*anti*‐*γ*‐**4**). In contrast, in the presence of a Lewis acid additive, aldehyde **3** becomes polarized, which induces a reaction via an ionic pathway to yield the *α*‐adduct (*syn*‐*α*‐**4**). The coordination of a ligand or solvent to the Ge center furnishes a highly coordinated dienolate [**2**‐Ligand] whose increased ionic character facilitates selective *α*‐addition, producing the *anti*‐*α*‐adduct.

**FIGURE 2:**
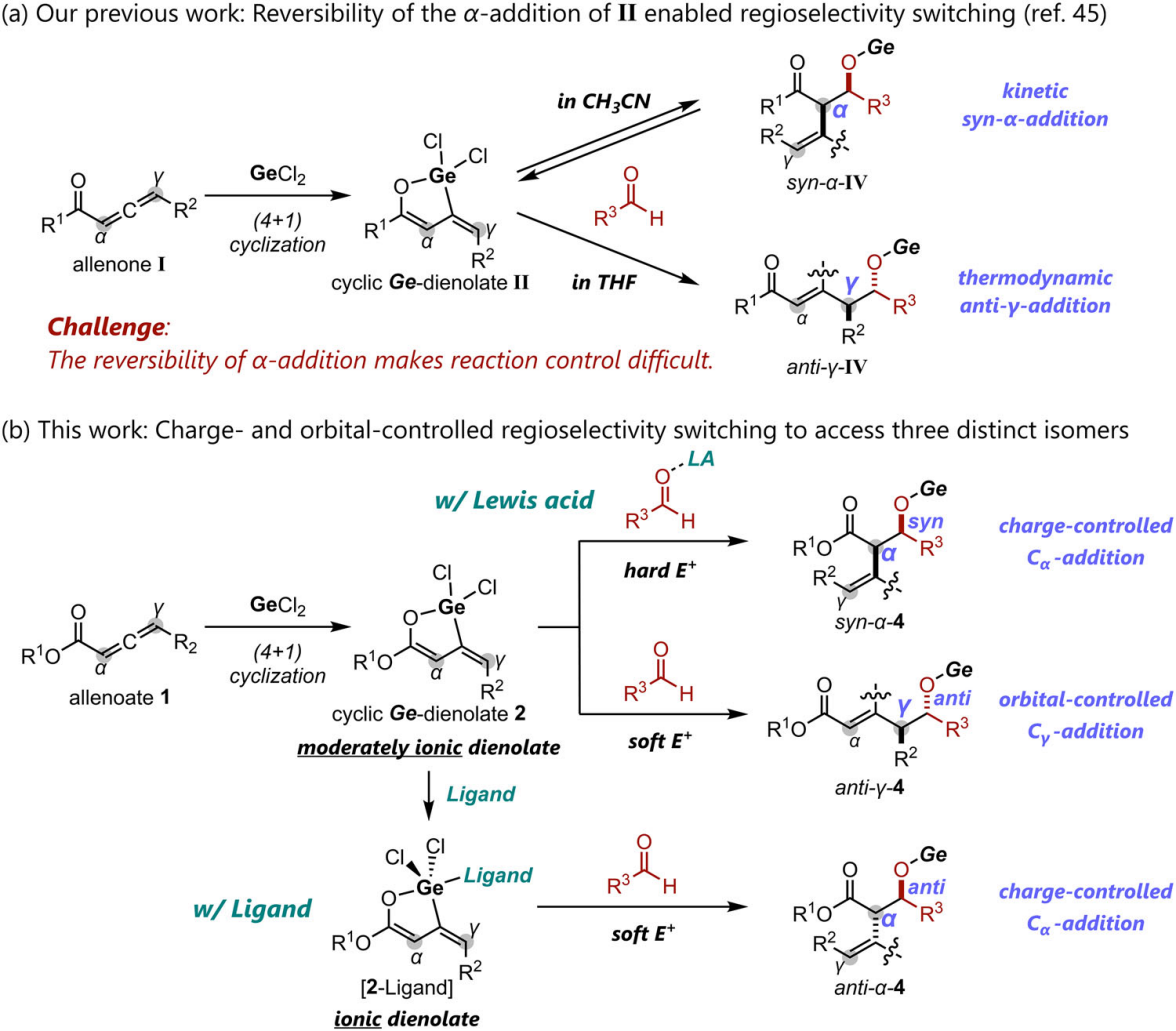
(a) Our previous work: The reversibility of the *α*‐addition of cyclic *Ge*‐dienolate **II** enabled regioselectivity switching in the aldol reaction. (b) This work: Regioselectivity switching via charge or orbital control of the aldol reaction of cyclic *Ge*‐dienolate **2** provides access to three distinct isomers.

## 2 Results and Discussion

We first investigated the generation of dienolate **2a** from ethyl allenoate **1a** using GeCl_2_‐dioxane (Figure 3a) based on our previously developed synthesis for dienolate **IIa** [45]. While ^1^H NMR measurements in typical deuterated solvents did not suggest the formation of **2a**, high‐resolution mass spectrometry detected the corresponding molecular ion peak (for details, see *Synthetic Procedures* in the Supporting Information). Quantum‐chemical calculations at the ωB97XD/6‐311G**(SMD)//ωB97XD/6‐31G* level revealed that the formation of **IIa** is an exergonic process (Δ*G* = –11.8 kcal/mol), whereas the formation of **2b** from methyl allenoate **1b** is an endergonic process (Δ*G* = 1.8 kcal/mol). These results would explain the difficulties associated with observing **2a** via NMR spectroscopy (Figure S10). Stabilizing the dienolate **2a** by adding external ligands was also attempted, but the generation of **2a** in the presence of ligands failed, resulting in the recovery of **1a** and Ge(II) salt.

**FIGURE 3:**
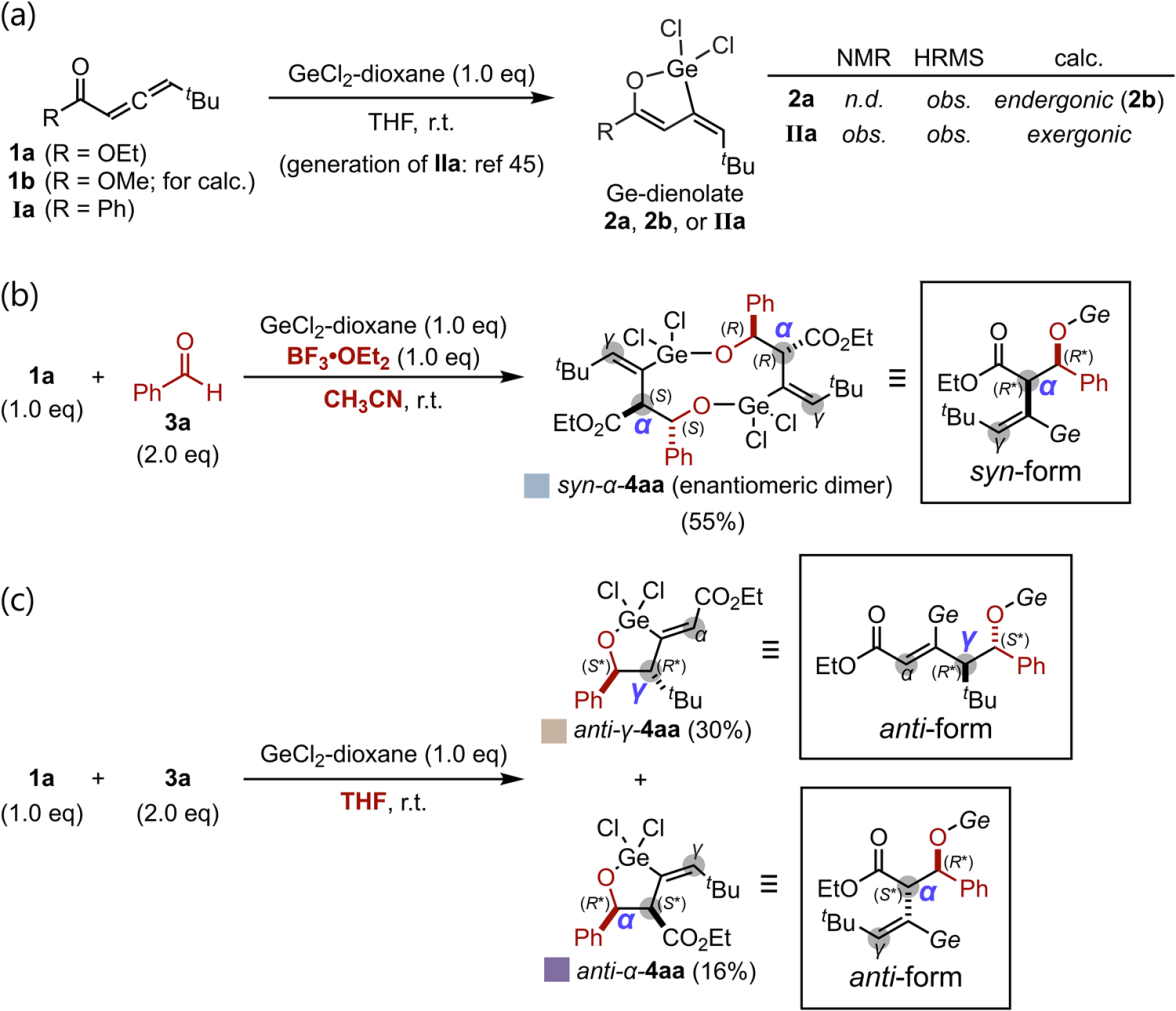
(a) Attempts to observe the formation of cyclic *Ge*‐dienolates **2a** and **IIa** [45] (b) Aldol reaction of *in situ*‐generated **2a** with **3a** in the presence of BF_3_ **·**OEt_2_ in CH_3_CN to give *syn*‐*α*‐**4aa**. (c) Aldol reaction of *in situ*‐generated **2a** with **3a** in THF to give a mixture of *anti*‐*α*‐**4aa** and *anti*‐*γ*‐**4aa**.

Next, we investigated the trapping of **2a** using benzaldehyde **3a**. The three‐component reaction of **1a**, **3a**, and GeCl_2_‐dioxane was carried out in CH_3_CN in the presence of BF_3_ **·**OEt_2_ under our previously reported conditions (Figure 3b) [45]. Gradual crystallization from the reaction mixture furnished single crystals suitable for X‐ray analysis, which revealed that aldol adduct *syn*‐*α*‐**4aa** was produced selectively and was present exclusively in the form of dimers of enantiomeric pairs (Figure 4a and S1−S4). This result is consistent with the reaction of **II** in the presence of an external Lewis acid. S1−S4 In contrast, the three‐component reaction in THF without an external Lewis acid, in which **II** affords the *anti*‐*γ*‐adduct, gave not only *anti*‐*γ*‐**4aa** (30%) but also its regioisomer *anti*‐*α*‐**4aa** (16%; Figure 3c). The solid‐state structures of the three regio‐ and stereoisomers were unambiguously determined via X‐ray crystallography (Figure 4).

**FIGURE 4:**
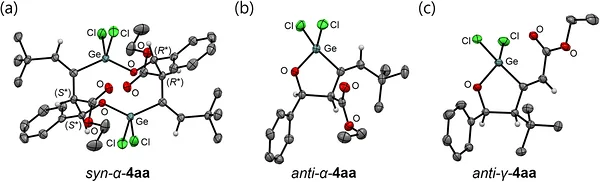
Molecular structures of aldol adducts (a) *syn*‐*α*‐**4aa**, (b) *anti*‐*α*‐**4aa**, and (c) *anti*‐*γ*‐**4aa** with thermal ellipsoids at 50% probability; some hydrogen atoms are omitted for clarity.

In the aldol reaction of **II**, the regioselectivity switching strongly depends on the reversibility of the *α*‐addition (Figure 2a), that is, only the insoluble and kinetic *syn*‐*α*‐adducts or the thermodynamically stable *anti*‐*γ*‐adducts are obtained [45]. In contrast to the previous studies [45], a *retro*‐aldol reaction was not observed for **2**, which could potentially generate up to four isomers via regioisomerism (*α*‐ or *γ*‐adduct) and diastereomerism (*syn*‐ or *anti*‐adduct). These findings indicate that the reaction conditions can directly influence the intrinsic reactivity of the dienolate. This observation provides a strong rationale for an investigation of the regioselectivity switching in nucleophilic addition reactions of dienolates via charge‐ or orbital‐controlled reaction pathways.

The reactions of allenoate **1a**, benzaldehyde **3a**, and GeCl_2_‐dioxane with or without additives afforded three distinct aldol adducts (*syn*‐*α*‐**4aa**, *anti*‐*α*‐**4aa**, and *anti*‐*γ*‐**4aa**). Three distinct reaction modes were identified, that is, (i) charge‐controlled *α*‐addition via activation by an external Lewis acid, (ii) charge‐controlled *α*‐addition via coordination to the Ge center, and (iii) orbital‐controlled *γ*‐addition in the absence of additives. The reaction conditions were systematically optimized to provide selective access to each of these modes (Table 1). As mentioned earlier, activation using a Lewis acid additive (BF_3_·OEt_2_ in CH_3_CN) afforded *syn*‐*α*‐**4aa** exclusively (entry 2; see also Figure 3b). In contrast, simply omitting the Lewis acid shifted the selectivity to favor *anti*‐*α*‐**4aa** (entry 1). *Anti*‐*α*‐**4aa** was obtained in similarly high selectivity in other coordinating solvents, such as acetone and EtOAc (entries 3–4), whereas the use of ethereal solvents resulted in decreased regioselectivity (entries 5 and 7). Moreover, *anti*‐*α*‐**4aa** was generated exclusively in the presence of *^n^* Bu_4_NCl in THF, albeit in low yield. This result suggests that Cl^–^ coordinates to the Ge center of **2a** to form a highly coordinated dienolate [**2a**‐Cl]^–^, which effectively promotes *α*‐addition (entry 6) [58, 59]. Conversely, non‐coordinating solvents, such as halogenated (PhCl; entry 8) and hydrocarbon (benzene or hexane; entries 9–10) solvents, primarily afforded *anti*‐*γ*‐**4aa**.

**TABLE 1:** Optimization of the reaction conditions for the selective synthesis of *syn*‐*α*‐**4aa**, *anti*‐*α*‐**4aa**, and *anti*‐*γ*‐**4aa**.^a^ ^.^

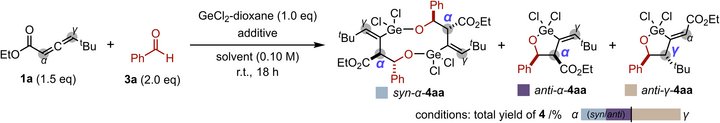				
entry	solvent	additive	yield / %	*syn*‐*α*‐4aa / *anti*‐*α*‐4aa / *anti*‐*γ*‐4aa^b^
1	CH_3_CN	—	71	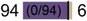
2^c^	CH_3_CN	BF_3_·OEt_2_ (2.0 eq)	75 (72)^d^	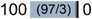
3	acetone	—	67	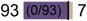
4	EtOAc	—	84 (63)^d^	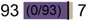
5	THF	—	62	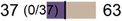
6	THF	*^n^* Bu_4_NCl (1.0 eq)	15	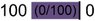
7	1,4‐dioxane	—	92	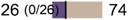
8	PhCl	—	84	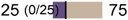
9	benzene	—	85	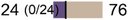
10	hexane	—	89 (100)^d^	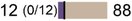

^a^ Yield and regioselectivity were determined based on ^1^H NMR measurements using 1,1,2,2‐tetrachloroethane as the internal standard.

^b^ Bar‐chart legend: blue = *syn*‐*α*‐**4aa**; purple = *anti*‐*α*‐**4aa**; yellow = *anti*‐*γ*‐**4aa**. The primary values to the left and right of the bars represent the *α*/*γ* ratio; the *syn*‐*α*/*anti*‐*α* ratio is provided in parentheses.

^c^ 1.5 equivalents of **3a** were employed.

^d^ Reactions were conducted on 1 mmol scales. The details are summarized in the SI.

To obtain deeper insight into the reaction‐condition‐dependent regio‐ and diastereoselectivity in the aldol reactions of **2a** with **3a**, quantum‐chemical calculations were performed at the ωB97XD/6‐311G**(SMD)//ωB97XD/6‐31G* level. Figure 5 summarizes the energy profiles for the nucleophilic attack of dienolate **2b** on **3a** under each condition.

**FIGURE 5:**
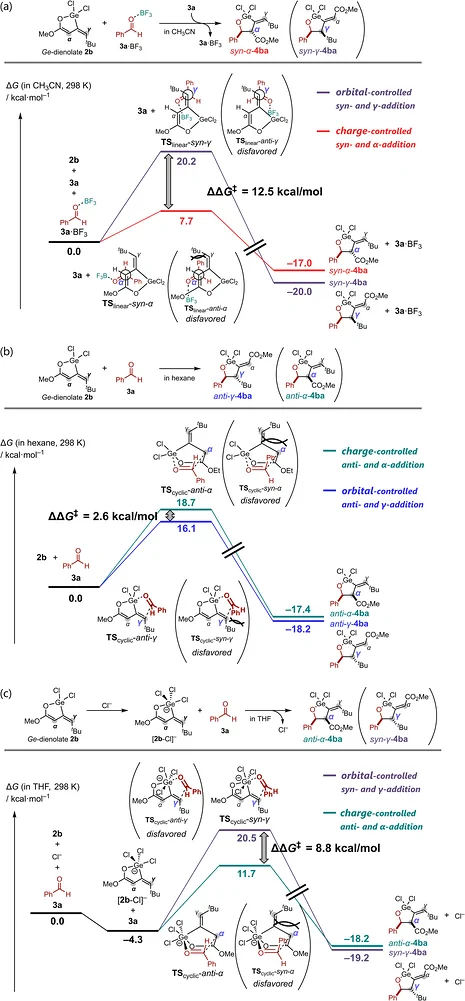
Energy profiles of the aldol reaction between **2b** and **3a** (a) in the presence of BF_3_ **·**OEt_2_ in acetonitrile, (b) in *n*‐hexane, and (c) in the presence of chloride anions in THF. DFT calculations were performed at the ωB97XD/6‐311G**(SMD)//ωB97XD/6‐31G* level. The structure of *syn*‐*α*‐**4aa** in Figure 5a is depicted as a monomeric geometry. All nucleophilic attacks are assumed to occur at the *Si*‐face of dienolate **2b**.

When the reaction is carried out in the presence of BF_3_ **·**OEt_2_ in CH_3_CN, BF_3_ acts as an external Lewis acid that activates **3a** to form complex **3a**·BF_3_ (Figure 5a). The coordination of the oxygen atoms in **2b** to BF_3_ may be possible, but it is an endergonic process. The subsequent aldol reaction between **2b** and **3a**·BF_3_ proceeds via an acyclic transition state. Among the four possible transition states leading to the different regio‐ and stereoisomers, **TS**_linear_‐*syn*‐*α* (associated with *α*‐addition) and **TS**_linear_‐*syn*‐*γ* (associated with *γ*‐addition) are energetically favored due to substituent effects (Figure S11). The *α*‐addition predominantly follows a charge‐controlled pathway, whereas the *γ*‐addition proceeds through an orbital‐controlled mechanism [1]. Activation of **3a** by the Lewis acid enhances the carbonyl polarity, thereby facilitating the charge‐controlled *α*‐addition (ΔΔ*G* ^‡^ = 12.5 kcal/mol; Figure 5a). A natural‐bond‐orbital (NBO) analysis indicated that BF_3_ coordination increases the positive charge at the carbonyl carbon from +0.440 in **3a** to +0.500 in **3a**·BF_3_ (Figure S15). This observation is consistent with previous findings for aldol reactions involving allenone‐derived dienolates **II** [45].

In the absence of additives, the Ge center of **2b** itself acts as the Lewis acid, and the reaction with **3a** proceeds through a cyclic transition state. Analysis of steric repulsion identifies **TS**_cyclic_‐*anti*‐*α* (for *α*‐addition) and **TS**_cyclic_‐*anti*‐*γ* (for *γ*‐addition) as the preferred pathways (Figure S12). Under these conditions, **TS**_cyclic_‐*anti*‐*γ* presents a lower activation barrier of 16.1 kcal/mol compared to 18.7 kcal/mol for **TS**_cyclic_‐*anti*‐*α*, resulting in the selective formation of *anti*‐*γ*‐**4ba** (Figure 5b). At the transition state of the reaction pathway, an envelope‐like 5‐membered cyclic transition state is adopted, resulting in the *γ*‐addition. These findings indicate that, in the absence of the external activation of aldehyde **3a**, orbital‐controlled *γ*‐addition is favored over *α*‐addition. In contrast, *Si*‐dienolates exclusively undergo *γ*‐addition under all the conditions examined, highlighting the unique tunability of *Ge*‐dienolates.

When an external ligand or coordinating solvent is present in the reaction mixture, it binds to the Ge center of **2b** to generate a highly coordinated dienolate. When a chloride anion was used as a model ligand (Table 1, entry 6), coordination of Cl^–^ to the Ge center of **2b** was found to stabilize the system and yield [**2b**‐Cl]^–^ quantitatively. In the subsequent addition to **3a**, the Ge center within [**2b**‐Cl]^–^ serves as an internal Lewis acid, facilitating the reaction through a cyclic transition state. The aldol reaction is anticipated to proceed through a boat‐like 6‐membered cyclic transition state due to the rigid cyclic structure of *Ge*‐enolates [60]. The moderate Lewis acidity of the highly coordinated Ge center is expected to enhance the facial selectivity of aldehydes. An analysis of substituent effects indicated that **TS**_cyclic_‐*anti*‐*α* and **TS**_cyclic_‐*syn*‐*γ* are the preferred pathways (Figure S13). Of the two, **TS**_cyclic_‐*anti*‐*α* has a lower activation barrier (Δ*G* ^‡^ = 11.7 kcal/mol) than **TS**_cyclic_‐*syn*‐*γ* (Δ*G* ^‡^ = 20.5 kcal/mol), resulting in the selective formation of *anti*‐*α*‐**4ba** (Figure 5c).

Figure 6a shows the HOMO and NBO charge at the *α*‐ and *γ*‐carbon atoms of **2b** and [**2b**‐Cl]^–^, as well as the corresponding silyl dienolate **2b′** and [**2b′**‐Cl]^–^. The NBO‐charge values indicate a significant increase in electron density upon coordination of chloride to the Ge center (see also Figure S15). Even though the energy levels of the HOMOs are almost comparable, the NBO‐charge value at the *α*‐carbon atom of [**2b**‐Cl]^–^ is larger than that of [**2b′**‐Cl]^–^. The formation of the highly coordinated dienolate also leads to a notable change in the HOMO distribution, that is, the HOMO of **2b** displays diene character, while that of [**2b**‐Cl]^–^ resembles an allyl anion. The optimized bond lengths of **2b** and [**2b**‐Cl]^–^ support this notion. Upon coordination of chloride to the Ge center, the O─C*_ipso_* and C*_α_* ─C*_β_* bonds of **2b** are compressed, resulting in an alleviation of the bond‐length alternation between the C*_α_* ─C*_β_* and C*_β_* ─C*_γ_* bonds (Figures 6b and S16). The geometric differences between **2b** to [**2b**‐Cl]^–^ indicate an enhanced contribution of the allyl‐anion character in the highly coordinated enolate [**2b**‐Cl]^–^ (Figure 6c). This transformation from a diene‐like to an allyl‐anion‐like electronic structure provides a direct mechanistic basis for the observed switch from orbital‐ to charge‐controlled reactivity. Consistent with our initial hypothesis (Figure 1c), the coordination ability of the Ge center allows effective modulation of both the electron density and orbital distribution of the dienolate, enabling regioselectivity switching.

**FIGURE 6:**
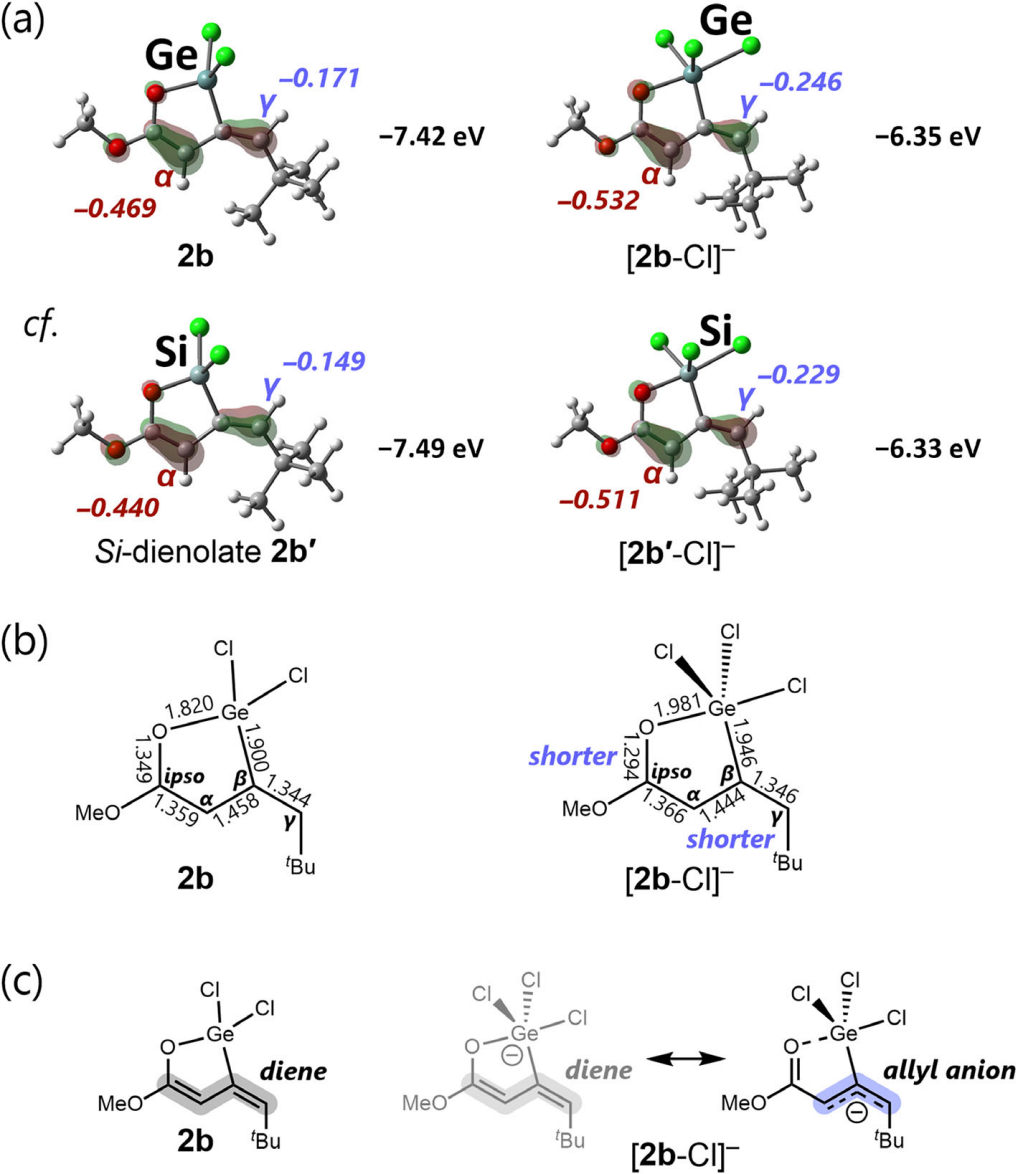
(a) Calculated orbital‐energy diagram of the HOMO and NBO charge at the *α*‐ and *γ*‐carbon atoms for **2b** and [**2b**‐Cl]^–^, as well as the corresponding silyl dienolate **2b′** and [**2b′**‐Cl]^–^. (b) Optimized bond lengths in **2b** and [**2b**‐Cl]^–^. Theoretical calculations were performed at the ωB97XD/6‐311G**(SMD)//ωB97XD/6‐31G* level. (c) Estimated resonance structures of **2b** and [**2b**‐Cl]^–^.

With the optimized conditions in hand, the reactions of various carbonyl compounds with **1a** were investigated (Scheme 1). A benzaldehyde bearing an electron‐withdrawing substituent (CO_2_Me; **3c**) gave the corresponding products *syn*‐*α*‐**4ac**, *anti*‐*α*‐**4ac**, and *anti*‐*γ*‐**4ac**. In contrast, electron‐donating aromatic aldehydes (**3b**, **3i**) yielded only *anti*‐*α*‐**4**, and *anti*‐*γ*‐**4**; the absence of the *syn*‐*α*‐**4** product in these cases was attributed to its inherent instability under the applied reaction conditions. Steric effects were evaluated using *o*‐, *m*‐, and *p*‐methylbenzaldehydes (**3d**–**f**). Regardless of the position of the methyl group, the corresponding products were obtained in good yield with high regio‐ and diastereoselectivity. Aldehydes having a naphthyl group (**3** **g** and **3** **h**) were also applicable. Unfortunately, the regio‐ and diastereoselectivity is largely restricted to aromatic aldehydes. Only the *anti*‐*γ*‐adduct was obtained in the reactions with *trans*‐cinnamaldehyde **3j** in very low yields. Notably, the high reactivity of dienolate **2a** enabled a reaction with typically challenging substrates, such as the bulky alkyl aldehyde **3k** and aromatic ketone **3l**. However, these substrates only gave the *anti*‐*α*‐**4** product under all conditions due to their distinct electrophilicity compared to benzaldehyde **3a**.

**SCHEME 1:**
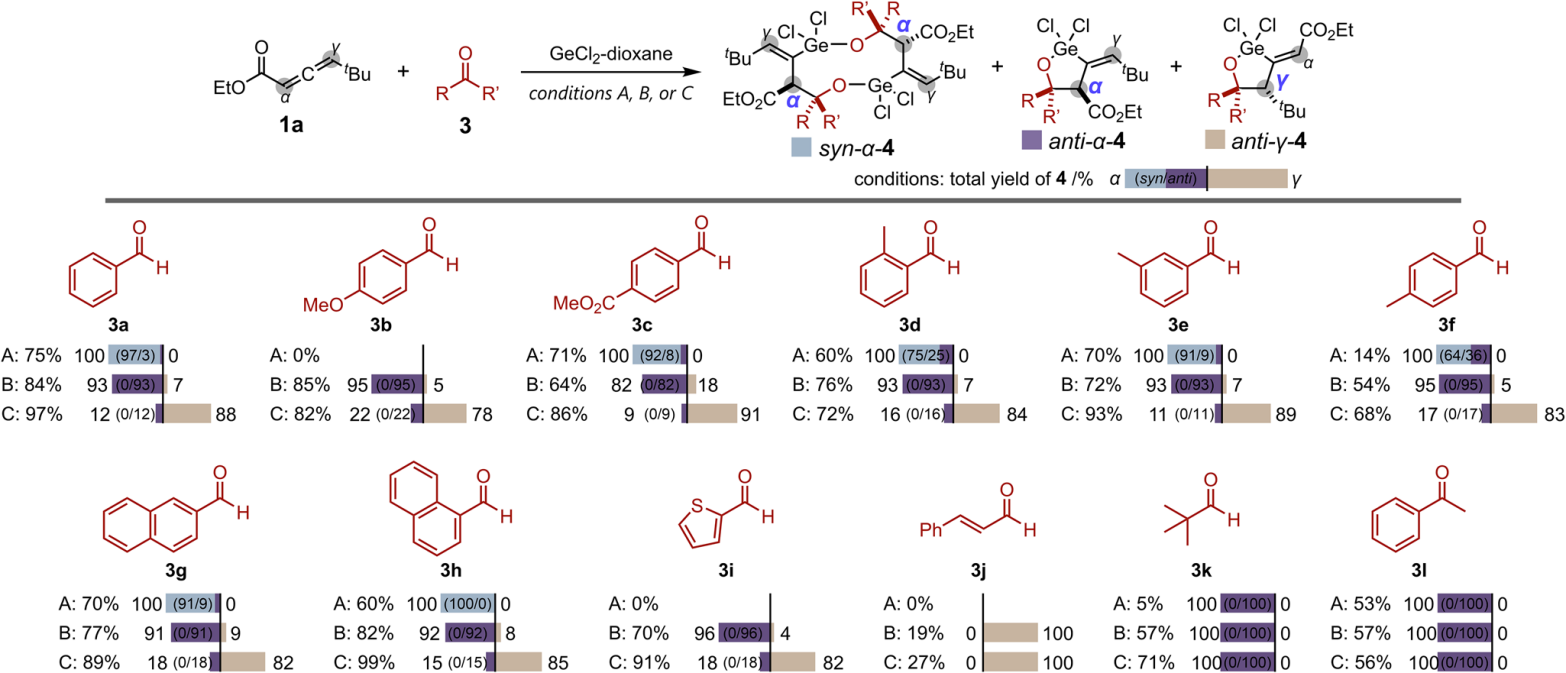
Substrate scope of carbonyl compounds **3**. *Conditions A*: Allenoate 1a (0.15 mmol), carbonyl compound 3 (0.15 mmol), GeCl_2_‐dioxane (0.10 mmol), BF_3_·OEt_2_ (0.20 mmol), CH_3_CN (1.0 mL), r.t., 18 h. *Conditions B*: Allenoate **1a** (0.15 mmol), carbonyl compound **3** (0.20 mmol), GeCl_2_‐dioxane (0.10 mmol), EtOAc (1.0 mL), r.t., 18 h. *Conditions C*: Allenoate **1a** (0.20 mmol), carbonyl compound **3** (0.20 mmol), GeCl_2_‐dioxane (0.10 mmol), hexane (1.0 mL), r.t., 18 h. Yield and regioselectivity were determined based on the isolated yield for *syn*‐*α*‐**4** and ^1^H NMR measurements for *anti*‐*α*‐**4** and *anti*‐*γ*‐**4** using 1,1,2,2‐tetrachloroethane as the internal standard. Bar‐chart legend: blue = *syn*‐*α*‐**4aa**; purple = *anti*‐*α*‐**4aa**; yellow = *anti*‐*γ*‐**4aa**. The primary values to the left and right of the bars represent the *α*/*γ* ratio; the *syn*‐*α*/*anti*‐*α* ratio is provided in parentheses.

Next, the scope of allenoates **1** was examined (Scheme 2). With regards to the ester moiety (CO_2_R^1^), benzyl ester **1c** reacted efficiently, producing all three isomeric products with excellent selectivity. In contrast, phenyl ester **1d** yielded only the *anti*‐*α*‐**4da** and *anti*‐*γ*‐**4da** adducts, likely due to differences in the reactivity of the dienolates derived from **1a** and **1d**. Allenoate esters with bearing substituents at the *γ*‐positions are essentially restricted to tertiary and bulky *tert*‐butyl (*^t^* Bu) and *tert*‐butyldimethylsilyl (TBS) groups. The substituent at the *γ*‐position (R^3^) was found to be critical. Allenoate **1e**, which features an isopropyl instead of a *tert*‐butyl group, produced a complex mixture of isomers that could not be identified. In contrast, allenoate **1f**, which contains a *tert*‐BuMe_2_Si group, afforded *anti*‐*α*‐**4fa** and *anti*‐*γ*‐**4fa** with excellent selectivity. Sterically demanding R^3^ groups promoted selective product formation under all conditions. Under conditions A, the electronic properties of R^3^ likely influence the preference between *syn*‐*α*‐**4aa** and *anti*‐*α*‐**4aa**.

**SCHEME 2:**
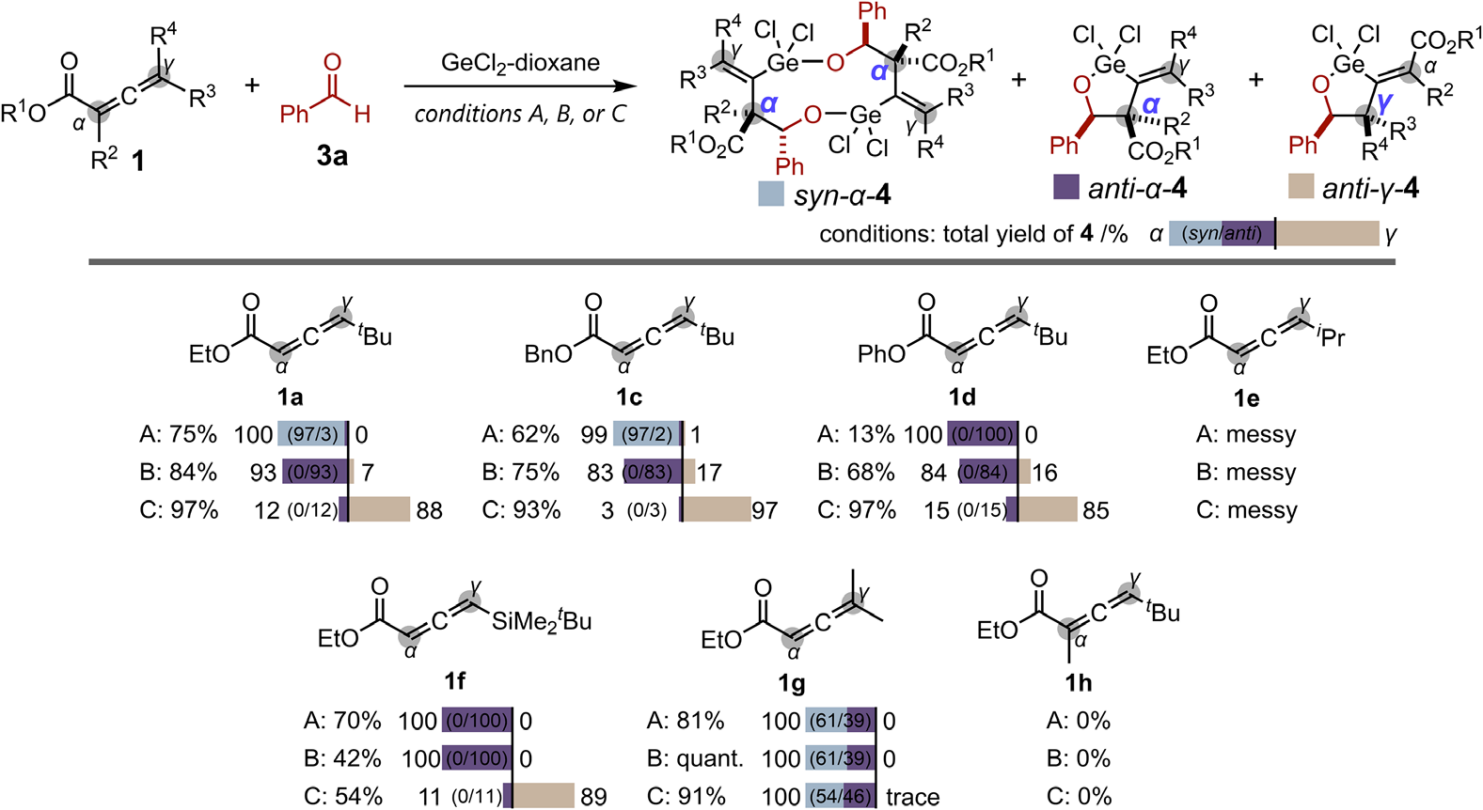
Substrate scope of allenoates **1**. *Conditions A*: Allenoate **1** (0.15 mmol), benzaldehyde **3a** (0.15 mmol), GeCl_2_‐dioxane (0.10 mmol), BF_3_·OEt_2_ (0.20 mmol), CH_3_CN (1.0 mL), r.t., 18 h. *Conditions B*: Allenoate **1** (0.15 mmol), benzaldehyde **3a** (0.20 mmol), GeCl_2_‐dioxane (0.10 mmol), EtOAc (1.0 mL), r.t., 18 h. *Conditions C*: Allenoate **1** (0.20 mmol), benzaldehyde **3a** (0.20 mmol), GeCl_2_‐dioxane (0.10 mmol), hexane (1.0 mL), r.t., 18 h. Yield and regioselectivity were determined based on the isolated yield for *syn*‐*α*‐**4** and ^1^H NMR measurements for *anti*‐*α*‐**4** and *anti*‐*γ*‐**4** using 1,1,2,2‐tetrachloroethane as the internal standard. Bar chart legend: blue = *syn*‐*α*‐**4aa**; purple = *anti*‐*α*‐**4aa**; yellow = *anti*‐*γ*‐**4aa**. The primary values to the left and right of the bars represent the *α*/*γ* ratio; the *syn*‐*α*/*anti*‐*α* ratio is provided in parentheses.

The reaction of trisubstituted allenoates was also investigated. The *γ*‐disubstituted allenoate **1** **g** predominantly gave *α*‐adducts *α*‐**4ga** with almost no selectivity, indicating that the *γ*‐dimethyl substituents suppress the reactivity at the *γ*‐position. Allenoate ester bearing substituent at the *α*‐position is not tolerated. The introduction of an *α*‐substituent (**1** **h**) severely suppressed the reaction, leading to decomposition of the dienolate.

Then, transformations of aldol adduct **4aa** were investigated (Scheme 3). Initially, we attempted the direct transformation of the germanium moiety of the aldol adducts **4aa** using Tamao‐Fleming oxidation, an Au‐ or Pd‐catalyst, strong acids (TFA or CF_3_SO_3_H), a fluoride ion, and a halogenated reagent (NBS or NIS). Unfortunately, all conditions resulted in complicated mixtures of unidentified products. Motivated by recent advances in the use of organogermanium derivatives in synthetic [61, 62, 63] and pharmaceutical chemistry [64, 65, 66, 67, 68, 69, 70] as well as materials science [71, 72, 73, 74, 75, 76, 77, 78], we aimed to synthesize multifunctionalized organogermanium compounds. Methylation of the Ge center in compounds **4** with a slight excess of MeMgBr produced *anti*‐*α*‐**5** and *anti*‐*γ*‐**5**, both of which retained their stereocenters. The use of a large excess of MeMgBr converted the ethyl‐ester groups into *tert*‐alcohols (C(Me)_2_OH), which can act as leaving groups in transition‐metal‐catalyzed *β*‐carbon elimination [79, 80, 81, 82, 83, 84]. This procedure afforded *anti*‐*α*‐**6**, *anti*‐*γ*‐**6**, and *syn*‐*α*‐**6**.

**SCHEME 3:**
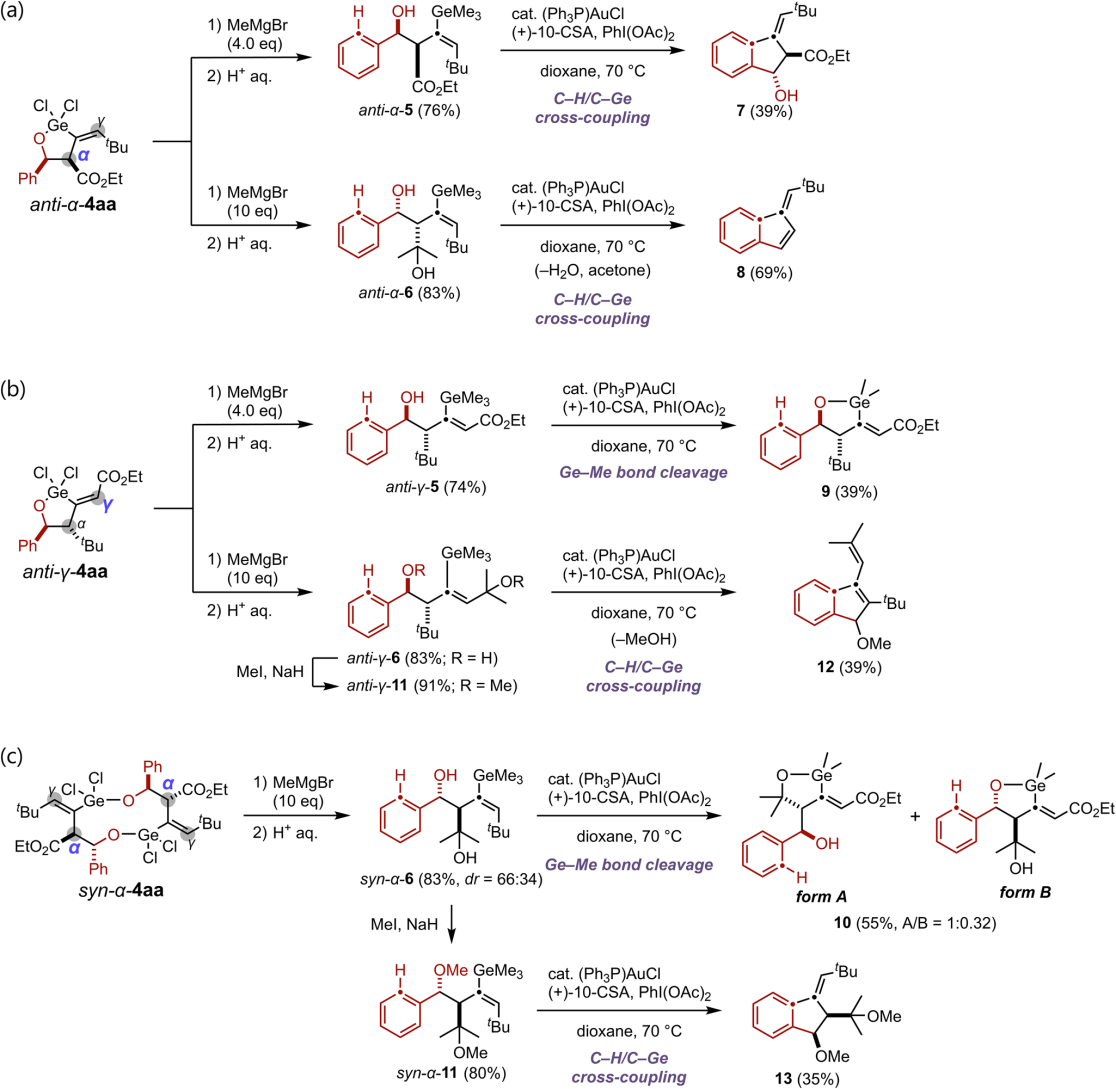
Transformations of (a) *anti*‐*α*‐**4aa**, (b) *anti*‐*γ*‐**4aa**, and (c) *syn*‐*α*‐**4aa**.

Subsequently, various reaction conditions were evaluated for the transformation of the GeMe_3_ groups, and the gold‐catalyzed C─H/C─Ge cross‐coupling method reported by Schoenebeck and coworkers was found to be effective [85]. *Anti*‐*α*‐**5** underwent intramolecular C─H/C─Ge coupling to yield methyleneindane derivative **7**, wherein the stereochemistry was retained. In contrast, *anti*‐*α*‐**6** produced benzofulvene derivative **8** through intramolecular coupling followed by *in situ* elimination of H_2_O and acetone.

For *anti*‐*γ*‐**5** and *syn*‐*α*‐**6**, Ge─Me bond cleavage was preferred over coupling, resulting in the formation of oxagermacycles **9** and **10**. Protection of the hydroxyl group as a methyl ether (*syn*‐*α*‐**11** and *anti*‐*γ*‐**11**) enabled efficient intramolecular coupling, yielding indene derivative **12** (with the concurrent elimination of methanol) and methyleneindane derivative **13**. C(sp^2^)─Ge bonds are typically more reactive than C(sp^3^)─Ge bonds [61, 62, 63], the selective cleavage of the C(sp^3^)─Ge bond in *syn*‐*α*‐**6** and *anti*‐*γ*‐**5** is likely promoted by intramolecular coordination of the hydroxyl group to the Ge center [86, 87]. The differing outcomes for diastereomers *anti*‐*α*‐**6** and *syn*‐*α*‐**6** indicate that subtle conformational differences significantly affect the reactivity at the Ge center. Further studies on the synthesis of multi‐functionalized organogermanes, particularly the construction of stereogenic Ge centers via selective C─Ge bond functionalization [88], are currently in progress in our laboratory.

## 3 Conclusion

We have developed an aldol reaction of cyclic *Ge*‐dienolates **2**, which are obtained from the by (4+1) cycloaddition of allenoates **1** with germanium(II) salts. This method provides access to three isomers via switching between charge‐ and orbital‐controlled pathways. Regiocontrol was achieved by balancing the charge density and orbital coefficients of *Ge*‐dienolates **2** and aldehydes **3**. The use of BF_3_·OEt_2_ as a Lewis acid, or the addition of a coordinating ligand such as Cl^–^ or a coordinating solvent, promotes charge‐controlled *α*‐addition to yield *syn*‐*α*‐**4aa** or *anti*‐*α*‐**4aa**. Without these additives, the reaction proceeds under orbital control to give *anti*‐*γ*‐**4aa**. The moderate ionic character and tunable coordination number of the Ge center are essential for this selectivity.

We have furthermore demonstrated the utility of this approach via the Au‐catalyzed intramolecular C─H/C─Ge cross‐coupling of the aldol adducts, which provides access to diverse fused‐ring systems. This work thus significantly expands the scope of dienolate chemistry and provides a versatile platform for the construction of stereogenic Ge centers through selective C─Ge bond modification.

## Conflicts of Interest

The authors declare no conflicts of interest.

## Supporting information



The authors have cited additional references within the Supporting Information [89, 90, 91, 92, 93, 94, 95, 96, 97, 98, 99]. Deposition Numbers 2548107 (*syn*‐*α*‐**4aa**), 2548108 (*syn*‐*α*‐**4ac**), 2548109 (*syn*‐*α*‐**4ae**), 2548110 (*syn*‐*α*‐**4ah**), 2548111 (*anti*‐*α*‐**4aa**), 2548112 (*anti*‐*γ*‐**4aa**), 2548113 (*anti*‐*γ*‐**5**), 2548114 (*anti*‐*γ*‐**6**), and 2548115 (*anti*‐*γ*‐**11**) contain the supplementary crystallographic data for this paper. These data can be obtained free of charge via the joint Cambridge Crystallographic Data Centre (CCDC) and the Fachinformationszentrum Karlsruhe Access Structures service.
**Supporting File 1**: chem71422‐sup‐0001‐SuppMat.pdf

## Data Availability

The data that supports the findings of this study are available in the supplementary material of this article.

## References

[1] I. Fleming , Molecular Orbitals and Organic Chemical Reactions, *Reference Edition* (John Wiley & Sons, 2010).

[2] N. S. Isaacs , Physical Organic Chemistry, *Second Edition* (Longman Scientific & Technical, 1995).

[3] M. Braun , Modern Enolate Chemistry (Wiley‐VCH, 2015).

[4] S. E. Denmark , J. R. Heemstra Jr. , and G. L. Beutner , “Catalytic, Enantioselective, Vinylogous Aldol Reactions,” Angewandte Chemie International Edition 44 (2005): 4682–4698. 10.1002/anie.200462338 15940727

[5] R. G. Pearson , “Hard and Soft Acids and Bases, HSAB, Part 1: Fundamental Principles,” Journal of Chemical Education 45 (1968): 581.

[6] R. G. Pearson , “Hard and Soft Acids and Bases, HSAB, Part II: Underlying Theories,” Journal of Chemical Education 45 (1968): 643.

[7] R. G. Pearson , “Hard and Soft Acids and Bases,” Journal of the American Chemical Society 85 (1963): 3533–3539.

[8] P. A. Chopard , R. F. Hudson , and G. Klopman , “241. Nucleophilic reactivity. Part VI. The reaction Between Triphenylphosphine and α‐Halogenated Ketones,” Journal of the Chemical Society (1965): 1379–1386, 10.1039/JR9650001379.

[9] R. Mahrwald , Modern Methods in Stereoselective Aldol Reactions (Wiley‐VCH, 2013).

[10] A. L. Kurts , N. K. Genkina , A. Macias , L. P. Beletskaya , and O. A. Reutov , “Reactivity of Ambident Anions,” Tetrahedron 27 (1971): 4777–4785.

[11] G. Stork and P. F. Hudrlik , “Isolation of Ketone Enolates as Trialkylsilyl Ethers,” Journal of the American Chemical Society 90 (1968): 4462–4464.

[12] H. O. House , L. J. Czuba , M. Gall , and H. D. Olmstead , “Chemistry of Carbanions. XVIII. Preparation of Trimethylsilyl Enol Ethers,” The Journal of Organic Chemistry 34 (1969): 2324–2336.

[13] F. W. Semmler , “Zur Kenntnis Der Bestandteile Ätherischer Öle (über *enol‐n*‐Heptanal (Önanthaldehyd)‐Acetat und “*Enol‐n*‐Octanal‐Acetat” *Berichte der Dtsch*),” Chemische Gesellschaft 42 (1909): 1161–1163.

[14] H. O. House , D. S. Crumrine , A. Y. Teranishi , and H. D. Olmstead , “Chemistry of Carbanions. XXIII. Use of Metal Complexes to Control the Aldol Condensation,” Journal of the American Chemical Society 95 (1973): 3310–3324.

[15] M. Kalesse , M. Cordes , G. Symkenberg , and H.‐H. Lu , “The Vinylogous Mukaiyama Aldol Reaction (VMAR) in Natural Product Synthesis,” Natural Product Reports 31 (2014): 563–594. 10.1039/c3np70102f 24595879

[16] M. Cordes and M. Kalesse , “Very Recent Advances in Vinylogous Mukaiyama Aldol Reactions and Their Applications to Synthesis,” Molecules 24 (2019): 3040. 10.3390/molecules24173040 PMC6749529 31443344

[17] S. Hosokawa , “Recent Development of Vinylogous Mukaiyama Aldol Reactions,” Tetrahedron Letters 59 (2018): 77–88.

[18] G. Casiraghi , F. Zanardi , G. Appendino , and G. Rassu , “The Vinylogous Aldol Reaction: A Valuable, Yet Understated Carbon−Carbon Bond‐Forming Maneuver,” Chemical Reviews 100 (2000): 1929–1972. 10.1021/cr990247i 11749280

[19] G. Casiraghi , L. Battistini , C. Curti , G. Rassu , and F. Zanardi , “The Vinylogous Aldol and Related Addition Reactions: Ten Years of Progress,” Chemical Reviews 111 (2011): 3076–3154. 10.1021/cr100304n 21434642

[20] C. Curti , L. Battistini , A. Sartori , and F. Zanardi , “New Developments of the Principle of Vinylogy as Applied to π‐Extended Enolate‐Type Donor Systems,” Chemical Reviews 120 (2020): 2448–2612. 10.1021/acs.chemrev.9b00481 PMC7993750 32040305

[21] M. Oiarbide and C. Palomo , “Extended Enolates: Versatile Intermediates for Asymmetric C–H Functionalization via Noncovalent Catalysis,” Chemistry: A European Journal 27 (2021): 10226–10246. 10.1002/chem.202100756 PMC8361983 33961323

[22] I. Fleming , J. Goldhill , and I. Paterson , “γ‐Sulphenylation of αβ‐Unsaturated Aldehydes, Ketones, and Esters: The use of O‐Silylated Dienolates,” Tetrahedron Letters 20 (1979): 3205–3208.

[23] T.‐H. Chan and P. Brownbridge , “Reaction of Electrophiles With 1,3‐bis(trimethylsiloxy)‐1‐Methoxybuta‐1,3‐Diene, a Dianion Equivalent of Methyl Acetoacetate,” Journal of the Chemical Society, Chemical Communications 15 (1979): 578.

[24] Y. Yamamoto , S. Hatsuya , and J. Yamada , “Stabilization and Activation of Dienolates With Germanium and Tin. Stereo‐ and Regioselective Aldol Reactions, Regioselective Coupling Reactions, and Regioselective Synthesis of Amino Acid Derivatives,” The Journal of Organic Chemistry 55 (1990): 3118–3128.

[25] M. W. Rathke and D. Sullivan , “The Preparation and Reactions of Enolate Anions Derived From α,β‐unsaturated Esters,” Tetrahedron Letters 13 (1972): 4249–4252.

[26] J. L. Herrmann , G. R. Kieczykowski , and R. H. Schlessinger , “Deconjugative Alkylation of the Enolate Anion Derived From Ethyl Crotonate,” Tetrahedron Letters 14 (1973): 2433–2436.

[27] I. Casinos and R. Mestres , “Synthesis of Unsaturated 5‐Hydroxy‐ and 7‐Oxo‐Acids by Addition of Unsaturated Carboxylic Acid Dienolates to Carbonyl Compounds,” Journal of the Chemical Society, Perkin Transactions 1 (1978): 1651.

[28] R. W. Dugger and C. H. Heathcock , “A General Synthesis of 5,6‐Dihydro‐.Alpha.‐Pyrones,” The Journal of Organic Chemistry 45 (1980): 1181–1185.

[29] T. Mukaiyama and A. Ishida , “A Convenient Method For the Preparation of δ‐Alkoxy‐α,β‐Unsaturated Aldehydes by Reaction of Acetals With 1‐Trimethylsiloxy‐1,3‐Butadiene,” Chemistry Letters 4 (1975): 319–322.

[30] I. Fleming , J. Goldhill , and I. Paterson , “γ‐Alkylation of Unsaturated Ketones and Esters: Zinc Bromide‐Catalysed Alkylation of *O*‐Silylated Dienolates,” Tetrahedron Letters 20 (1979): 3209–3212.

[31] J. A. Katzenellenbogen and A. L. Crumrine , “Regioselectivity in the Alkylation of Lithium and Copper Ester Dienolates. Allylic Transposition associated with .Gamma.‐Alkylation of Copper Dienolates. Allylic Transposition Associated with γ‐Alkylation of Copper Dienolates,” Journal of the American Chemical Society 96 (1974): 5662–5663.

[32] J. A. Katzenellenbogen and A. L. Crumrine , “Selective .Gamma. Alkylation of Dienolate Anions Derived From .Alpha,.Beta.‐Unsaturated Acids. Applications to the Synthesis of Isoprenoid Olefins,” Journal of the American Chemical Society 98 (1976): 4925–4935.

[33] R. Gompper and H.‐U. Wagner , “The Concept of Allopolarization. Effects of Substituents on the Reactions of Ambifunctional Anions,” Angewandte Chemie International Edition in English 15 (1976): 321–333.

[34] M. Woyciechowska , G. Forcher , S. Buda , and J. Mlynarski , “General Switch in Regioselectivity in the Mukaiyama Aldol Reaction of Silyloxyfuran With Aldehydes in Aqueous Solvents,” Chemical Communications 48 (2012): 11029–11031. 10.1039/c2cc36656h 23037879

[35] A. Adamkiewicz , I. Węglarz , A. Butkiewicz , M. Woyciechowska , and J. Mlynarski , “Lewis Acid‐Catalyzed Stereoselective *α*‐Addition of Chiral Aldehydes to Cyclic Dienol Silanes: Aqueous Synthesis of Chiral Butenolides,” Advanced Synthesis & Catalysis 362 (2020): 667–678.

[36] J. R. Green , M. Majewski , B. I. Alo , and V. Snieckus , “γ‐Silylated α,β‐Unsaturated Amides. Formation by [1,5] Silicon Migration From O‐Silylated Vinyl Ketene Aminals and Fluoride‐ and Lewis Acid‐Mediated Aldol Condensations,” Tetrahedron Letters 27 (1986): 535–538.

[37] J. R. Green , B. I. Alo , M. Majewski , and V. Snieckus , “γ‐Silylated α,β‐Unsaturated Amides — Preparation by [1,5]‐Sigmatropic Rearrangement and Use as Masked Dienolate Equivalents in Carbonyl Condensations,” Canadian Journal of Chemistry 87 (2009): 745–759.

[38] S. Saito , M. Shiozawa , M. Ito , and H. Yamamoto , “Conceptually New Directed Aldol Condensation Using Aluminum Tris(2,6‐diphenylphenoxide),” Journal of the American Chemical Society 120 (1998): 813–814.

[39] J. A. Gazaille and T. Sammakia , “The Vinylogous Aldol Reaction of Unsaturated Esters and Enolizable Aldehydes Using the Novel Lewis Acid Aluminum Tris(2,6‐di‐2‐naphthylphenoxide),” Organic Letters 14 (2012): 2678–2681. 10.1021/ol300738f PMC3427784 22621176

[40] P. R. Johnson and J. D. White , “Condensation of Crotonic and Tiglic Acid Dianions With Aldehydes and Ketones,” The Journal of Organic Chemistry 49 (1984): 4424–4429.

[41] P. Ballester , A. Costa , A. García‐Raso , and R. Mestres , “Unsaturated Carboxylic Acid Dienolates. Addition to Substituted Cyclohexanones. Inverted Kinetic and Thermodynamic Stereoselectivities,” Tetrahedron Letters 28 (1987): 3853–3856.

[42] G. Cardillo , M. Orena , G. Porzi , and S. Sandri , “Synthesis of Malyngolide, an Antibiotic From the Marine Blue‐Green Alga Lyngbya majuscula Gomont,” The Journal of Organic Chemistry 46 (1981): 2439–2442.

[43] H. L. van Maanen , H. Kleijn , J. T. B. H. Jastrzebski , M. T. Lakin , A. L. Spek , and G. van Koten , “Dualistic Reactivity of Lithium and Zinc Dienolates With Imines: Effects of Counterion, Temperature, and Substituents on α‐ and γ‐Coupling,” Journal of Organic Chemistry 59 (1994): 7839–7848.

[44] R. Fan and T. Hudlicky , “Tin Analogs of the Vinylogous Reformatsky Reaction. Regio‐ and Stereochemical Considerations,” Tetrahedron Letters 30 (1989): 5533–5536.

[45] T. Nojima , A. Konishi , and M. Yasuda , “Aldol Reactions of Germyl Dienolates with Precise Control over Regioselectivity and Perfect Diastereoselectivity,” Advanced Synthesis & Catalysis 368 (2026): e70404.

[46] Y. Yamamoto , S. Hatsuya , and J. Yamada , “Carbon–Carbon Bond Formation at the γ‐position of Dienolates via the Germanium Masked Dienolates,” Journal of the Chemical Society, Chemical Communications 24 (1988): 1639–1640.

[47] Y. Yamamoto , H. Okano , and J. Yamada , “Regiocontrolled Conjugate Addition of Germanium‐Masked Dienolates to Michael Acceptors,” Tetrahedron Letters 32 (1991): 4749–4752.

[48] Y. Yamamoto , S. Hatsuya , and J. Yamada , “Regioselective Synthesis of either α‐ or γ‐ amino Acid Derivatives via Li, Sn‐Masked, and/or Ge‐Masked Dienolates,” Tetrahedron Letters 30 (1989): 3445–3448.

[49] M. Yasuda , S. Tanaka , and A. Baba , “Reductive Cross‐Aldol Reaction Using Bromoaldehyde and an Aldehyde Mediated by Germanium(II): One‐Pot, Large‐Scale Protocol,” Organic Letters 7 (2005): 1845–1848. 10.1021/ol050533i 15844921

[50] S. Tanaka , M. Yasuda , and A. Baba , “Germanium(II)‐Mediated Reductive Cross‐Aldol Reaction of Aldehydes: Synthesis of Aldols With Diastereocontrolled Quaternary Carbon Centers,” Synlett 2007 (2007): 1720–1724.

[51] S. Tanaka , N. Tagashira , K. Chiba , M. Yasuda , and A. Baba , “Germanium(II)‐Mediated Reductive Mannich‐Type Reaction of *α*‐Bromoketones to *N*‐Alkylimines,” Angewandte Chemie International Edition 47 (2008): 6620–6623. 10.1002/anie.200800194 18646232

[52] S. Tanaka , N. Tagashira , K. Chiba , M. Yasuda , and A. Baba , “Germanium(II)‐Mediated Reductive Cross‐Aldol Reaction of Bromoaldehydes With Aldehydes: NMR Studies and Ab Initio Calculations,” The Journal of Organic Chemistry 73 (2008): 6312–6320. 10.1021/jo800904u 18630967

[53] A. Konishi , Y. Minami , T. Hosoi , K. Chiba , and M. Yasuda , “First Isolation and Characterization of the Highly Coordinated Group 14 Enolates: Effects of the Coordination Controls on the Geometry and Tautomerization of Germyl Enolates,” Chemistry—A European Journal 22 (2016): 12688–12691. 10.1002/chem.201603147 27377796

[54] Y. Minami , A. Konishi , and M. Yasuda , “Stereocontrolled Synthesis of Triols Containing Four Asymmetric Centers: Application of *C*,*O*‐Chelated Germyl Enolates to a Diastereoselective Aldol Reaction,” Organic Letters 20 (2018): 4148–4152. 10.1021/acs.orglett.8b01778 29938514

[55] Y. Minami , A. Konishi , and M. Yasuda , “Synthesis of α‐Alkenyl α,β‐Unsaturated Ketones via Dehydrogermylation of Oxagermacycles With Regeneration of the Germanium(II) Species,” Organic Letters 21 (2019): 9818–9823. 10.1021/acs.orglett.9b03454 31682128

[56] Y. Minami , K. Nishida , A. Konishi , and M. Yasuda , “Characterization of Highly Coordinated Allylgermanes: Pivotal Players for Enhanced Nucleophilicity and Stereoselectivity,” Chemistry—An Asian Journal 15 (2020): 1852–1857. 10.1002/asia.202000392 32274892

[57] T. Nojima , Y. Izumi , A. Konishi , and M. Yasuda , “Toward Multi‐Functionalized Organogermaniums: Diastereoselective Transformations of Germacycles Derived From Cheletropic Reactions of Boron‐Substituted Butadienes With Ge(II) Salts,” European Journal of Organic Chemistry 27 (2024): e202400960.

[58] M. Yasuda , K. Hayashi , Y. Katoh , I. Shibata , and A. Baba , “Highly Controlled Chemoselectivity of Tin Enolate by Its Hybridization State. Anionic Complex of Tin Enolate Coordinated by Tetrabutylammonium Bromide as Halo Selective Reagent,” Journal of the American Chemical Society 120 (1998): 715–721.

[59] M. Yasuda , K. Chiba , and A. Baba , “An ab Initio Computational Study on the Reaction of Organotin Enolates: Comparison of Highly Coordinated Tin Reagent With Noncoordinated Reagent,” Journal of the American Chemical Society 122 (2000): 7549–7555.

[60] C. J. Cowden and I. Paterson , “Asymmetric Aldol Reactions Using Boron Enolates,” Organic Reactions 51 (1997): 1–200.

[61] Q.‐H. Xu and B. Xiao , “Organogermanium(IV) Compounds in Photo‐Induced Radical Reactions,” Organic Chemistry Frontiers 9 (2022): 7016–7027.

[62] C. Fricke and F. Schoenebeck , “Organogermanes as Orthogonal Coupling Partners in Synthesis and Catalysis,” Accounts of Chemical Research 53 (2020): 2715–2725. 10.1021/acs.accounts.0c00527 33118804

[63] T. Rogova , E. Ahrweiler , M. D. Schoetz , and F. Schoenebeck , “Recent Developments With Organogermanes: Their Preparation and Application in Synthesis and Catalysis,” Angewandte Chemie International Edition 63 (2024): e202314709. 10.1002/anie.202314709 37899306

[64] T. Yamakawa , H. Kagechika , E. Kawachi , Y. Hashimoto , and K. Shudo , “Retinobenzoic Acids. 5. Retinoidal Activities of Compounds Having a Trimethylsilyl or Trimethylgermyl Group(s) in human Promyelocytic Leukemia Cells HL‐60,” Journal of Medicinal Chemistry 33 (1990): 1430–1437. 10.1021/jm00167a024 2329565

[65] R. Tacke , D. Reichel , P. G. Jones , et al., “Pharmacological Discrimination Between Enantiomeric germanes by Muscarinic Receptors: A Study on germanium/Silicon Bioisosterism,” Journal of Organometallic Chemistry 521 (1996): 305–323.

[66] R. Tacke , M. Merget , R. Bertermann , M. Bernd , T. Beckers , and T. Reissmann , “Syntheses and Properties of Silicon‐ and Germanium‐Containing α ‐Amino Acids and Peptides: A Study on C/Si/Ge Bioisosterism,” Organometallics 19 (2000): 3486–3497.

[67] R. Tacke , T. Kornek , T. Heinrich , et al., “Syntheses and Pharmacological Characterization of Achiral and Chiral Enantiopure C/Si/Ge‐analogous Derivatives of the Muscarinic Antagonist Cycrimine: A Study on C/Si/Ge Bioisosterism,” Journal of Organometallic Chemistry 640 (2001): 140–165.

[68] M. Merget , K. Günther , M. Bernd , E. Günther , and R. Tacke , “Syntheses of Racemic and Non‐Racemic Silicon‐ and Germanium‐Containing α‐Amino Acids of the Formula Type H_2_NCH(CH_2_ElR_3_)COOH (El=Si, Ge; R=Organyl) and Incorporation of d‐H_2_NCH(CH_2_SiMe_3_)COOH and d‐H_2_NCH(CH_2_GeMe_3_)COOH Into Biologically Active Decapeptides: A study on C/Si/Ge Bioisosterism,” Journal of Organometallic Chemistry 628 (2001): 183–194.

[69] S. Fujii , Y. Miyajima , H. Masuno , and H. Kagechika , “Increased Hydrophobicity and Estrogenic Activity of Simple Phenols With Silicon and Germanium‐Containing Substituents,” Journal of Medicinal Chemistry 56 (2013): 160–166. 10.1021/jm3013757 23214428

[70] S. Fujii , “Expanding the Chemical Space of Hydrophobic Pharmacophores: The Role of Hydrophobic Substructures in the Development of Novel Transcription Modulators,” MedChemComm 7 (2016): 1082–1092.

[71] F. C. Leavitt , T. A. Manuel , and F. Johnson , “Novel Heterocyclo Pentadienes,” Journal of the American Chemical Society 81 (1959): 3163–3164.

[72] S. Yamaguchi , Y. Itami , and K. Tamao , “Group 14 Metalloles With Thienyl Groups on 2,5‐Positions: Effects of Group 14 Elements on Their π‐Electronic Structures,” Organometallics 17 (1998): 4910–4916.

[73] B. L. Lucht , M. A. Buretea , and T. D. Tilley , “Poly(2,5‐diphenylgermole): Incorporation of a Germole Ring Into a Conjugated Polymer,” Organometallics 19 (2000): 3469–3475.

[74] C. M. Amb , S. Chen , K. R. Graham , et al., “Dithienogermole as a Fused Electron Donor in Bulk Heterojunction Solar Cells,” Journal of the American Chemical Society 133 (2011): 10062–10065. 10.1021/ja204056m 21644517

[75] Y.‐C. Pao , Y.‐L. Chen , Y.‐T. Chen , et al., “Synthesis and Molecular Properties of Tricyclic Biselenophene‐Based Derivatives With Nitrogen, Silicon, Germanium, Vinylidene, and Ethylene Bridges,” Organic Letters 16 (2014): 5724–5727. 10.1021/ol502793e 25337789

[76] S. Bähr , H. Ogasawara , S. Yamaguchi , and M. Oestreich , “An Expedient Procedure for the Synthesis of Benzo[4,5]silolo[2,3‐ b ]thiophenes and Related Systems,” Organometallics 36 (2017): 4013–4019.

[77] Y. Suzuki , T. Sasamori , J.‐D. Guo , and N. Tokitoh , “A Redox‐Active Bis(ferrocenyl)germylene and Its Reactivity,” Chemistry—A European Journal 24 (2018): 364–368. 10.1002/chem.201705598 29193403

[78] C. R. W. Reinhold , M. Schmidtmann , B. Tumanskii , and T. Müller , “Radicals and Anions of Siloles and Germoles,” Chemistry—A European Journal 27 (2021): 12063–12068. 10.1002/chem.202101415 PMC8453960 33978965

[79] Y. Terao , H. Wakui , T. Satoh , M. Miura , and M. Nomura , “Palladium‐Catalyzed Arylative Carbon−Carbon Bond Cleavage of α,α‐Disubstituted Arylmethanols,” Journal of the American Chemical Society 123 (2001): 10407–10408. 10.1021/ja016914i 11604000

[80] P. Zhao , C. D. Incarvito , and J. F. Hartwig , “Direct Observation of β‐Aryl Eliminations From Rh(I) Alkoxides,” Journal of the American Chemical Society 128 (2006): 3124–3125. 10.1021/ja058550q 16522075

[81] E. Ozkal , B. Cacherat , and B. Morandi , “Cobalt(III)‐Catalyzed Functionalization of Unstrained Carbon–Carbon Bonds Through β‐Carbon Cleavage of Alcohols,” ACS Catalysis 5 (2015): 6458–6462.

[82] H. Wang , I. Choi , T. Rogge , N. Kaplaneris , and L. Ackermann , “Versatile and Robust C–C Activation by “Chelation‐Assisted Manganese Catalysis”,” Nature Catalysis 1 (2018): 993–1001.

[83] Y. Qiu , A. Scheremetjew , and L. Ackermann , “Electro‐Oxidative C–C Alkenylation by Rhodium(III) Catalysis,” Journal of the American Chemical Society 141 (2019): 2731–2738. 10.1021/jacs.8b13692 30636408

[84] R. Deng , J. Xi , Q. Li , and Z. Gu , “Enantioselective Carbon‐Carbon Bond Cleavage for Biaryl Atropisomers Synthesis,” Chemistry 5 (2019): 1834–1846.

[85] C. Fricke , A. Dahiya , W. B. Reid , and F. Schoenebeck , “Gold‐Catalyzed C–H Functionalization With Aryl Germanes,” ACS Catalysis 9 (2019): 9231–9236. 10.1021/acscatal.9b02841 PMC6781487 31608162

[86] K. Matsuoka , N. Komami , M. Kojima , et al., “Chemoselective Cleavage of Si‐C(sp^3^) Bonds in Unactivated Tetraalkylsilanes Using Iodine Tris(trifluoroacetate),” Journal of the American Chemical Society 143 (2021): 103–108. 10.1021/jacs.0c11645 33356223

[87] M.‐Y. Xu and B. Xiao , “Germatranes and Carbagermatranes: (hetero)Aryl and Alkyl Coupling Partners in Pd‐Catalyzed Cross‐Coupling Reactions,” Chemical Communications 57 (2021): 11764–11775. 10.1039/d1cc04373k 34661207

[88] T. He , H. F. T. Klare , and M. Oestreich , “Arenium‐Ion‐Catalysed Halodealkylation of Fully Alkylated Silanes,” Nature 623 (2023): 538–543. 10.1038/s41586-023-06646-9 37821704

[89] O. V. Dolomanov , L. J. Bourhis , R. J. Gildea , J. A. K. Howard , and H. Puschmann , “OLEX2: A Complete Structure Solution, Refinement and Analysis Program,” Journal of Applied Crystallography 42 (2009): 339–341.

[90] R. W. Lang and H. Hansen , “Eine einfache Allencarbonsäureester‐Synthese Mittels Der Wittig ‐Reaktion,” Helvetica Chimica Acta 63 (1980): 438–455.

[91] G. Himbert and D. Fink , “Cycloadditionen. 26. Einfluß von Substituenten in der Allenischen ω‐Position Auf Das Thermische Verhalten Von Allencarbonsäurearylester,” Journal für Praktische Chemie/Chemiker‐Zeitung 339 (1997): 233–242.

[92] A. S. K. Hashmi , R. Döpp , C. Lothschütz , M. Rudolph , D. Riedel , and F. Rominger , “Scope and Limitations of Palladium‐Catalyzed Cross‐Coupling Reactions With Organogold Compounds,” Advanced Synthesis & Catalysis 352 (2010): 1307–1314.

[93] Y. Kita , Y. Tsuzuki , S. Kitagaki , and S. Akai , “Chemistry of Silylketenes: Simple Preparation of Silylallenes by the Reaction of Silylketenes With Phosphorus Ylides,” Chemical and Pharmaceutical Bulletin 42 (1994): 233–236.

[94] G. E. Keck , R. L. Giles , V. J. Cee , C. A. Wager , T. Yu , and M. B. Kraft , “Total Synthesis of Epothilones B and D: Stannane Equivalents for β‐Keto Ester Dianions,” The Journal of Organic Chemistry 73 (2008): 9675–9691. 10.1021/jo802215v PMC2736362 18991385

[95] P. Selig and W. Raven , “A Convenient Allenoate‐Based Synthesis of 2‐Quinolin‐2‐yl Malonates and β‐Ketoesters,” Organic Letters 16 (2014): 5192–5195. 10.1021/ol502554e 25216060

[96] R. W. Lang and H. Hansen , “Allenic Esters From α‐Phosphoranylidene Esters and Acid Chlorides: Ethyl 2,3‐Pentadienoate,” Organic Syntheses 62 (1984): 202.

[97] M. J. Frisch , G. W. Trucks , H. B. Schlegel , et al., Gaussian 16 (2016), *Revision C.01*.

[98] A. E. Reed , L. A. Curtiss , and F. Weinhold , “Intermolecular Interactions From a Natural Bond Orbital, Donor‐Acceptor Viewpoint,” Chemical Reviews 88 (1988): 899–926.

[99] E. D. Glendening , C. R. Landis , and F. Weinhold , “Natural Bond Orbital Methods,” WIREs Computational Molecular Science 2 (2012): 1–42.

