# Supplementary material for: Regioselective Aldol Reactions Directed by Charge and Orbital Effects in Cyclic Germyl Dienolates: Synthesis of Three Distinct Regio‐ and Stereoisomers
Source: Chemistry. 2026 Jul 17;32(38):e71422. doi: 10.1002/chem.71422 (PMC13660488; doi:10.1002/chem.71422)
Supplement: Supplementary file 1 [file CHEM-32-e71422-s001.pdf]

Supporting Information

Regioselective Aldol Reactions Directed by Charge and Orbital Effects in Cyclic Germyl Dienolates: Synthesis of Three Distinct Regio- and Stereoisomers

Taishi Nojima,[a] Akihito Konishi,*[a,b,c] and Makoto Yasuda*[a,b]

[a] Department of Applied Chemistry, Graduate School of Engineering, The University of Osaka, 2-1 Yamadaoka, Suita, Osaka 565-0871, Japan

[b] Innovative Catalysis Science Division, Institute for Open and Transdisciplinary Research Initiatives (ICS-OTRI), The University of Osaka, Suita, Osaka 565-0871, Japan

[c] Center for Future Innovation, Graduate School of Engineering, The University of Osaka, Suita, Osaka 565-0871, Japan

Table of contents

1. General 4

2. Materials 4

3. Synthetic procedures 5

3-1. Generation and detection of cyclic-*Ge*-dienolate 2a from the reaction of allenoate 1a with $GeCl_2$-dioxane 5

3-2. *syn-α*-Selective aldol reaction of allenoate 1a with aldehyde 3 using $GeCl_2$-dioxane 6

3-3. *anti-α*-Selective aldol reaction of allenoate 1a with aldehyde 3 using $GeCl_2$-dioxane 10

3-4. *anti-γ*-Selective aldol reaction of allenoate 1a with aldehyde 3 using $GeCl_2$-dioxane 30

3-5. *syn-α*-Selective aldol reaction of allenoate 1 with benzaldehyde 3a using $GeCl_2$-dioxane 48

3-6. *anti-α*-Selective aldol reaction of allenoate 1 with benzaldehyde 3a using $GeCl_2$-dioxane 54

3-7. *anti-γ*-Selective aldol reaction of allenoate 1 with benzaldehyde 3a using $GeCl_2$-dioxane 63

3-8. Derivatizations of *anti-α*-4aa 70

3-9. Derivatizations of *anti-γ*-4aa 76

3-10. Derivatizations of *syn-α*-4aa 84

3-11. Synthesis of starting allenoates 1 93

4. X-ray crystallographic data 95

4-1. Diethyl (3*E*,4*R,5*R**,8*E*,9*S**,10*S**)-2,2,7,7-tetrachloro-3,8-bis(2,2-dimethylpropylidene)-5,10-diphenyl-1,6,2,7-dioxadigermecane-4,9-dicarboxylate *syn-α*-4aa** 95

4-2. Diethyl (3*E*,4*R,5*R**,8*E*,9*S**,10*S**)-2,2,7,7-tetrachloro-3,8-bis(2,2-dimethylpropylidene)-5,10-bis{4-(methoxycarbonyl)phenyl}-1,6,2,7-dioxadigermecane-4,9-dicarboxylate *syn-α*-4ac** 95

4-3. Diethyl (3*E*,4*R,5*R**,8*E*,9*S**,10*S**)-2,2,7,7-tetrachloro-3,8-bis(2,2-dimethylpropylidene)-5,10-di-*m*-tolyl-1,6,2,7-dioxadigermecane-4,9-dicarboxylate *syn-α*-4ae** 96

4-4. Diethyl (3*E*,4*R,5*R**,8*E*,9*S**,10*S**)-2,2,7,7-tetrachloro-3,8-bis(2,2-dimethylpropylidene)-5,10-di(naphthalen-1-yl)-1,6,2,7-dioxadigermecane-4,9-dicarboxylate *syn-α*-4ah** 96

4-5. Ethyl (4*R,5*S**,*E*)-2,2-dichloro-3-(2,2-dimethylpropylidene)-5-phenyl-1,2-oxagermolane-4-carboxylate *anti-α*-4aa** 97

4-6. Ethyl (*Z*)-2-{(4*R,5*S**)-4-(*tert*-butyl)-2,2-dichloro-5-phenyl-1,2-oxagermolan-3-ylidene)acetate *anti-γ*-4aa** 97

4-7. Ethyl (*R,*Z*)-4-{(*S**)-hydroxy(phenyl)methyl}-5,5-dimethyl-3-(trimethylgermyl)hex-2-enoate *anti-γ*-5** 98

4-8. (1*R,2*S**,*Z*)-2-(*tert*-Butyl)-5-methyl-1-phenyl-3-(trimethylgermyl)hex-3-ene-1,5-diol *anti-γ*-6** 98

4-9. [(*R,*Z*)-2-Methoxy-5-{(*S**)-methoxy(phenyl)methyl}-2,6,6-trimethylhept-3-en-4-yl]trimethylgermane *anti-γ*-11** 99

5. Theoretical calculations 100

5-1. General 100

5-2. Summary for molecular geometries included in the estimated reaction pathway 101

5-2-1. Formation of the dienolate 2b or IIa 101

5-2-2. Aldol reaction of 2b with 3a·BF_3 in acetonitrile 102

5-2-3. Aldol reaction of 2b with 3a in hexane 104
5-2-4. Aldol reaction of [2a-Cl]$^{-}$ with 3a in THF 106
5-3. Changes in electronic and structural properties of 2b and 3c upon additive activation 108
5-4. Cartesian coordinates for optimized geometries 110
5-4-1. Formation of the dienolate 2b or IIa in THF 110
5-4-2. Aldol reaction of 2b with 3a·BF_3 in acetonitrile 112
5-4-3. Aldol reaction of 2b with 3a in hexane 115
5-4-4. Aldol reaction of [2b-Cl]$^{-}$ with 3a in THF 117
5-4-5. The structures of corresponding cyclic-*Si*-dienolates 2b′ and [2b′-Cl]$^{-}$ 119
6. References.. 120

1. General

NMR spectra were recorded on JEOL-AL400, JEOL-ECS400 (400 MHz for ^{1}H, 100 MHz for ^{13}C, and 78.7 MHz for ^{29}Si). For ^{1}H NMR and ^{13}C NMR spectra, $Si(CH_3)_4$ and $CHCl_3$ were used as internal standards, respectively. For ^{29}Si NMR spectra, Me_4Si in $CDCl_3$ was used as an external standard. ^{1}H and ^{13}C NMR signals of compounds were assigned using HMQC, HSQC, HMBC, COSY, 1D ^{13}C NMR under conditions of proton off-resonance spin decoupling. Positive FAB/CI, EI, MALDI-TOF, ESI/DART mass spectra were recorded on a JEOL JMS-700, a Shimadzu GCMS-QP2010 Ultra, a JEOL JMS-S3000, and a JEOL JMS-T100LP respectively. IR spectra were recorded as thin films or as solids in KBr pellets on a JASCO FT/IR 6200 spectrophotometer, or as solids in ATR-mode on a JASCO FT/IR-4X spectrophotometer. Data collection for X-ray crystal analysis was performed on Rigaku/XtaLAB Synergy-S/Mo (MoK$_\alpha$ λ = 0.71075 Å) and Rigaku/XtaLAB Synergy-S/Cu (CuK$_\alpha$ λ = 1.54187 Å) diffractometers. All non-hydrogen atoms were refined with anisotropic displacement parameters and hydrogen atoms were placed at calculated positions and refined "riding" on their corresponding carbon atoms by Olex2[1] program.

2. Materials

Anhydrous dichloromethane, acetonitrile, acetone, ethyl acetate, THF, 1,4-dioxane, benzene and hexane were purchased and used as obtained. Anhydrous chlorobenzene was prepared by distillation from CaH_2. All reagents were obtained from commercial suppliers and used as received. All reactions were carried out under nitrogen or in a nitrogen-filled glovebox. The allenoates **1a**,[2] **1d**,[3] **1e**,[4] **1f**,[5] **1g**,[6] and **1h**[7] were prepared by using a slightly modified literature-known procedure.[2,8]

3. Synthetic procedures

3-1. Generation and detection of cyclic-*Ge*-dienolate 2a from the reaction of allenoate 1a with $GeCl_2$-dioxane

2,2-Dichloro-3-(2,2-dimethylpropylidene)-5-ethoxy-2,3-dihydro-1,2-oxagermole 2a

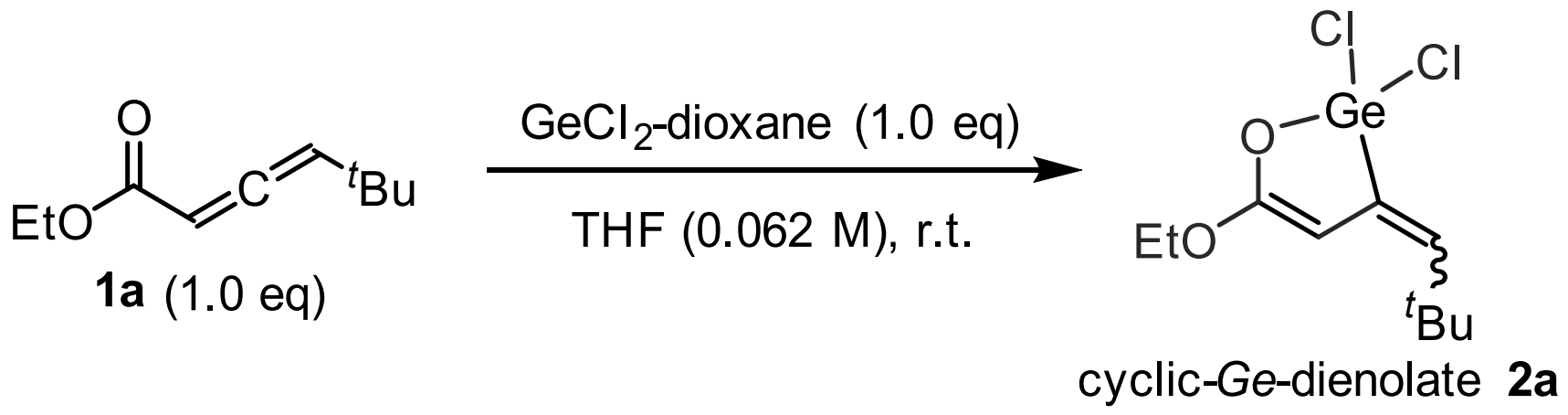

To a mixture of **1a** (0.00830 g, 0.0493 mmol) in THF (0.80 mL) was added $GeCl_2$-dioxane (0.0115 g, 0.496 mmol). The cyclic-*Ge*-dienolate **2a** was observed by HRMS at room temperature. However, **2a** was not observed NMR measurement in the common deuterated solvent ($CDCl_3$, CD_2Cl_2, CD_3CN, C_6D_6, THF-d_8, DMF-d_7).

HRMS (DART$^+$)

Calculated ($C_{10}H_{17}O_2Cl_2Ge$): 312.9806 ($[M+H]^+$)

Found: 312.9821 ($[M+H]^+$)

Obs.

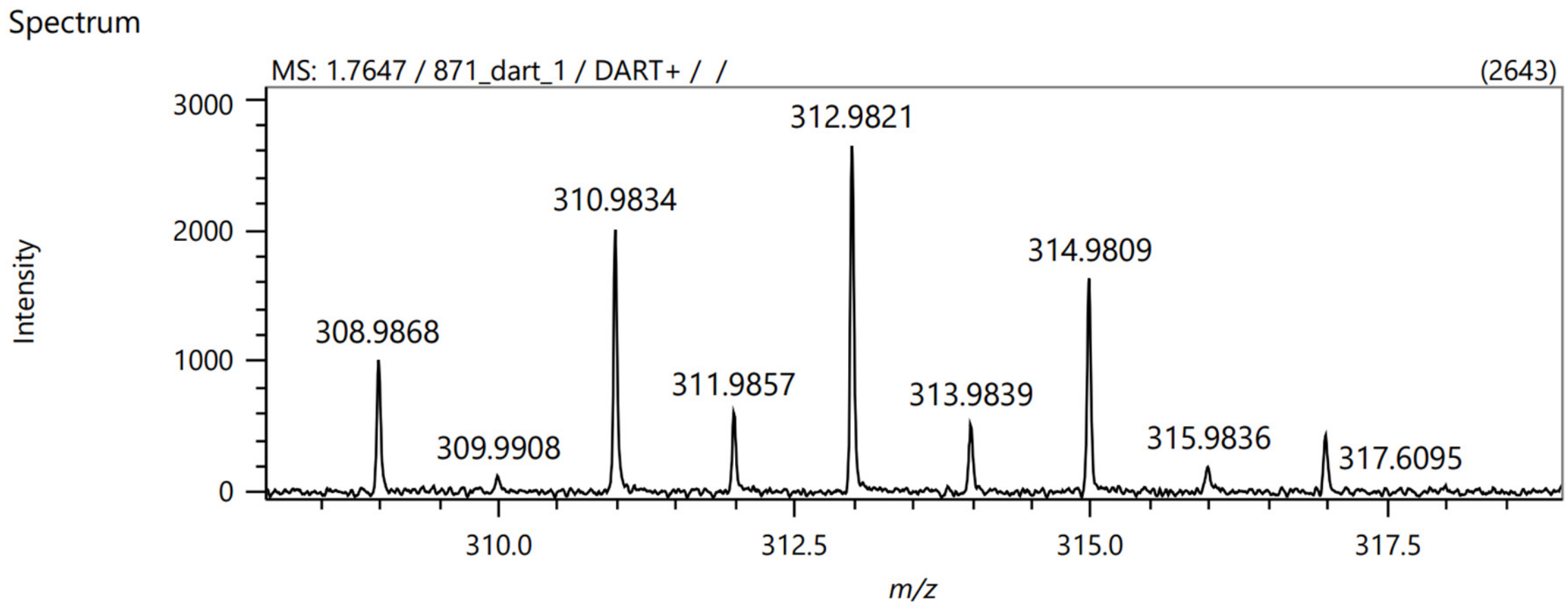

Sim.

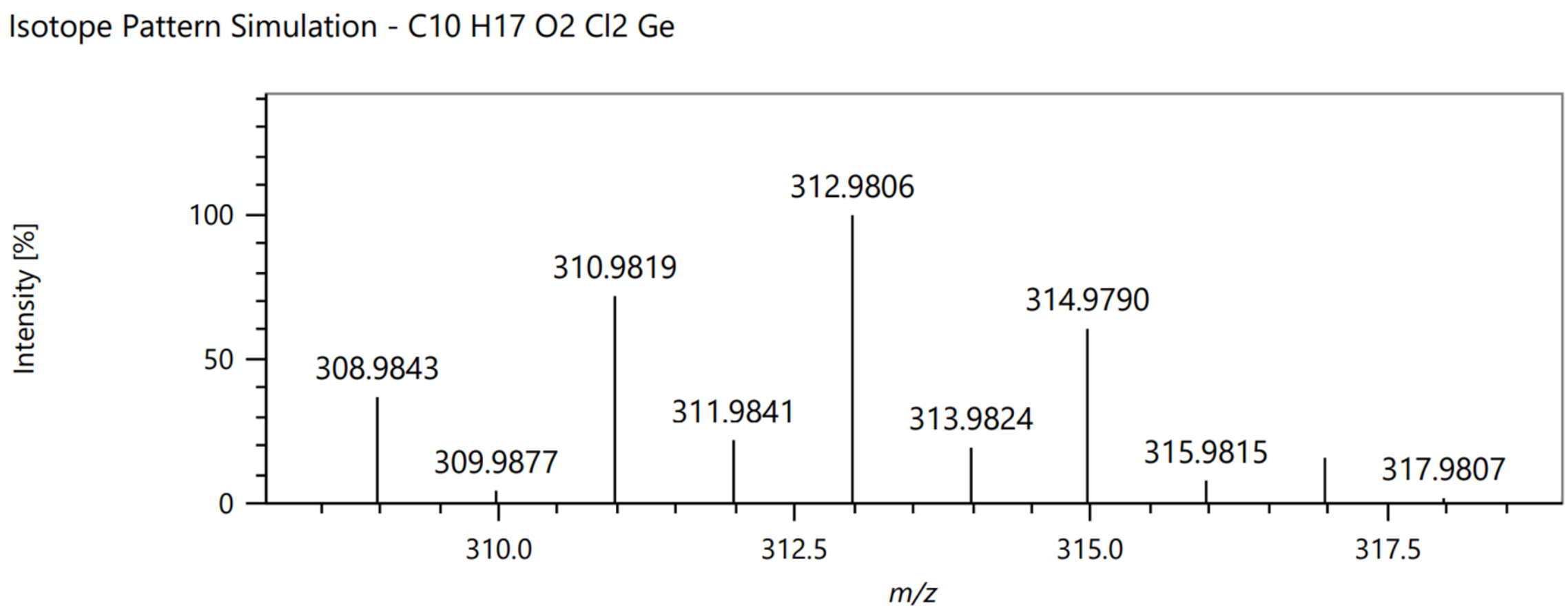

3-2. *syn-α*-Selective aldol reaction of allenoate 1a with aldehyde 3 using $GeCl_2$-dioxane

Diethyl (3*E*,4*R,5*R**,8*E*,9*S**,10*S**)-2,2,7,7-tetrachloro-3,8-bis(2,2-dimethylpropylidene)-5,10-diphenyl-1,6,2,7-dioxadigermecane-4,9-dicarboxylate *syn-α*-4aa**

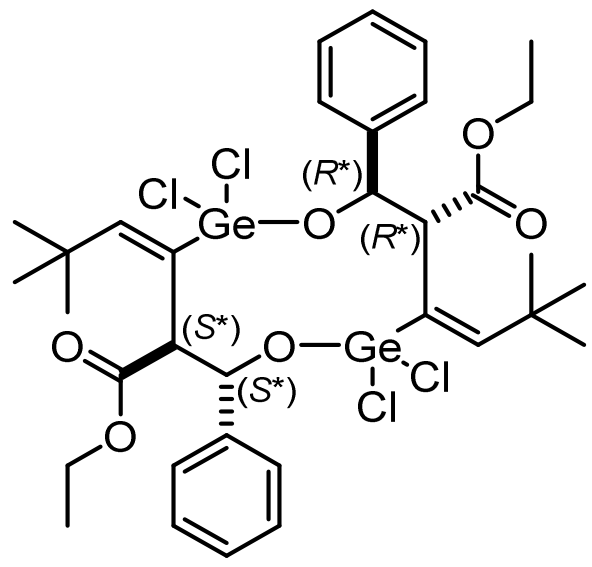

To a mixture of **1a** (0.0249 g, 0.148 mmol), benzaldehyde **3a** (0.0152 mL, 0.150 mmol), and $BF_3{\cdot}OEt_2$ (0.0252 mL, 0.100 mmol) in CH_3CN (1.0 mL) was added $GeCl_2$-dioxane (0.0234 g, 0.101 mmol). After the reaction mixture was left to stand at room temperature for 18 h, the precipitated colorless solid was filtrated and washed with CH_3CN, to give the *syn-α*-**4aa** as the enantiomeric dimer (0.0306 g, 73%). The filtrate was evaporated under vaccum, to give *anti-α*-**4aa** (2%) determined by ^{1}H NMR using 1,1,2,2-tetrachloroethane as an internal standard. The total yield of **4aa** and the ratio of two isomers are 75% and *syn-α*-**4aa**/*anti-α*-**4aa** = 97/3. The *syn-α*-**4aa** is promptly decomposed at the solution in any solvents, which hampered recording any NMR spectrum.

mp 174.5–175.4 °C; **IR** (ATR) 3065 (w), 2959 (m), 1689 (s), 1469 (w), 1374 (m), 1330 (m), 1191 (m), 1083 (w), 1047 (s) cm^{-1}; **Analysis** $C_{34}H_{44}Cl_4Ge_2O_6$ (835.78) Calculated: C, 48.86; H, 5.31, Found: C, 48.95; H, 5.40.

Large-scale synthesis

To a mixture of **1a** (0.252 g, 1.50 mmol), benzaldehyde **3a** (0.152 mL, 1.50 mmol), and $BF_3{\cdot}OEt_2$ (0.252 mL, 1.00 mmol) in CH_3CN (5.0 mL) was added $GeCl_2$-dioxane (0.232 g, 1.00 mmol). After the reaction mixture was left to stand at room temperature for 18 h, the precipitated colorless solid was filtrated and washed with CH_3CN, to give the *syn-α*-**4aa** as the enantiomeric dimer (0.301 g, 72%).

Diethyl (3*E*,4*R,5*R**,8*E*,9*S**,10*S**)-2,2,7,7-tetrachloro-3,8-bis(2,2-dimethylpropylidene)-5,10-bis{4-(methoxycarbonyl)phenyl}-1,6,2,7-dioxadigermecane-4,9-dicarboxylate *syn*-*α*-4ac**

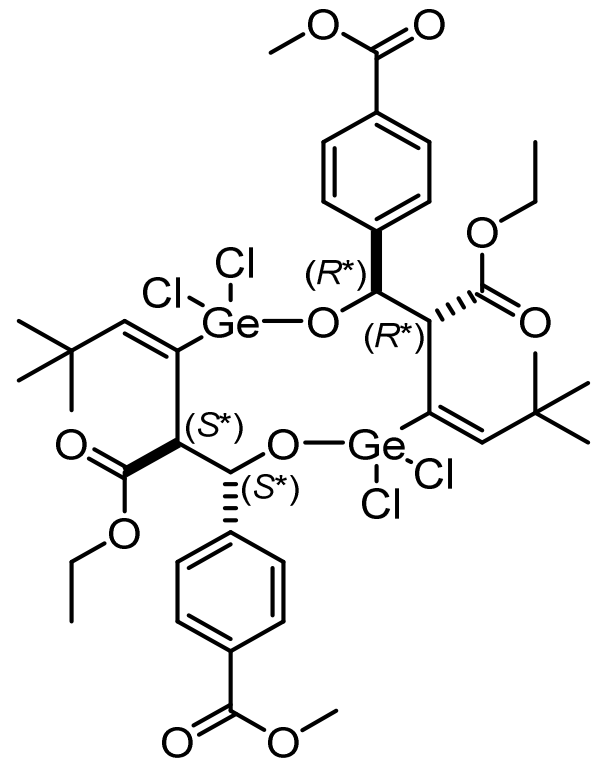

To a mixture of **1a** (0.0253 g, 0.150 mmol), methyl 4-formylbenzoate **3c** (0.0246 g, 0.150 mmol), and $BF_3 \cdot OEt_2$ (0.0252 mL, 0.100 mmol) in CH_3CN (1.0 mL) was added $GeCl_2$-dioxane (0.0232 g, 0.100 mmol). After the reaction mixture was left to stand at room temperature for 18 h, the precipitated colorless solid was filtrated and washed with CH_3CN, to give the *syn*-*α*-**4ac** as the enantiomeric dimer (0.0309 g, 65%). The filtrate was evaporated under vaccum, to give *anti*-*α*-**4ac** (6%) determined by ^{1}H NMR using 1,1,2,2-tetrachloroethane as an internal standard. The total yield of **4ac** and the ratio of two isomers are 71% and *syn*-*α*-**4ac**/*anti*-*α*-**4ac** = 92/8. The *syn*-*α*-**4ac** is promptly decomposed at the solution in any solvents, which hampered recording any NMR spectrum.

mp 108.8–109.5 °C; **IR** (ATR) 3034 (w), 2957 (m), 1720 (s), 1441 (m), 1370 (m), 1284 (s), 1194 (m), 1112 (m), 1055 (m) cm^{-1}; **Analysis** $C_{38}H_{48}Cl_4Ge_2O_{10}$ (951.85) Calculated: C, 47.95; H, 5.08, Found: C, 48.09; H, 5.13.

Diethyl (3*E*,4*R,5*R**,8*E*,9*S**,10*S**)-2,2,7,7-tetrachloro-3,8-bis(2,2-dimethylpropylidene)-5,10-di-*o*-tolyl-1,6,2,7-dioxadigermecane-4,9-dicarboxylate *syn*-*α*-4ad**

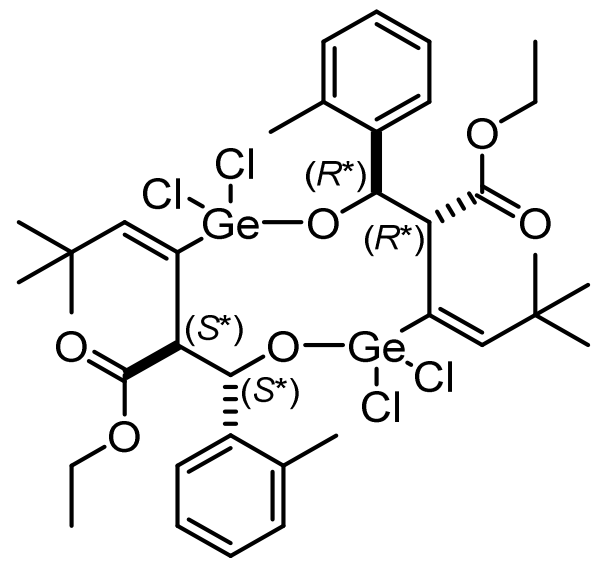

To a mixture of **1a** (0.0252 g, 0.150 mmol), 2-methylbenzaldehyde **3d** (0.0173 mL, 0.150 mmol), and $BF_3 \cdot OEt_2$ (0.0252 mL, 0.100 mmol) in CH_3CN (1.0 mL) was added $GeCl_2$-dioxane (0.0232 g, 0.100 mmol). After the reaction mixture was left to stand at room temperature for 18 h, the precipitated colorless solid was filtrated and washed with CH_3CN, to give the *syn*-*α*-**4ad** as the enantiomeric dimer (0.0192 g, 45%). The filtrate was evaporated under vaccum, to give *anti*-*α*-**4ad** (15%) determined by ^{1}H NMR using 1,1,2,2-tetrachloroethane as an internal standard. The total yield of **4ad** and the ratio of two isomers are 60% and *syn*-*α*-**4ad**/*anti*-*α*-**4ad** = 75/25. The *syn*-*α*-**4ad** is promptly decomposed at the solution in any solvents, which hampered recording any NMR spectrum.

mp 189.2–190.1 °C; **IR** (ATR) 3073 (w), 2966 (m), 1704 (s), 1461 (m), 1371 (m), 1329 (m), 1299 (m), 1200 (m), 1031 (s) cm^{-1}; **Analysis** $C_{36}H_{48}Cl_4Ge_2O_6$ (863.83) Calculated: C, 50.06; H, 5.60, Found: C, 50.07; H, 5.71.

Diethyl (3*E*,4*R,5*R**,8*E*,9*S**,10*S**)-2,2,7,7-tetrachloro-3,8-bis(2,2-dimethylpropylidene)-5,10-di-*m*-tolyl-1,6,2,7-dioxadigermecane-4,9-dicarboxylate *syn-α*-4ae**

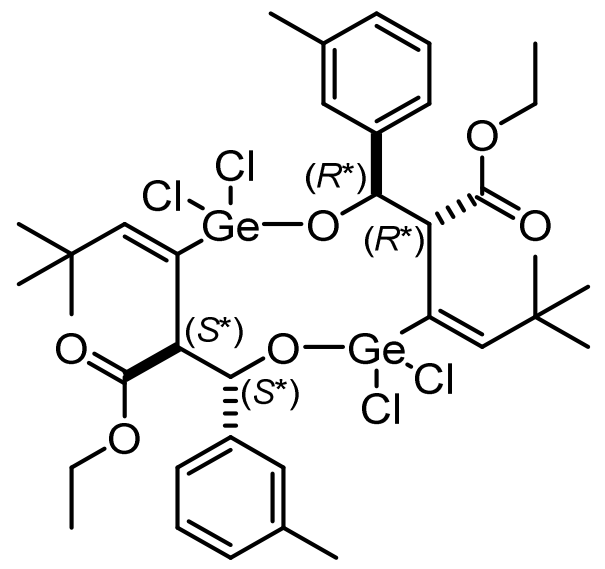

To a mixture of **1a** (0.0252 g, 0.150 mmol), 3-methylbenzaldehyde **3e** (0.0177 mL, 0.150 mmol), and $BF_3 \cdot OEt_2$ (0.0252 mL, 0.100 mmol) in CH_3CN (1.0 mL) was added $GeCl_2$-dioxane (0.0231 g, 0.0997 mmol). After the reaction mixture was left to stand at room temperature for 18 h, the precipitated colorless solid was filtrated and washed with CH_3CN, to give the *syn-α*-**4ae** as the enantiomeric dimer (0.0277 g, 64%). The filtrate was evaporated under vaccum, to give *anti-α*-**4ae** (6%) determined by ^{1}H NMR using 1,1,2,2-tetrachloroethane as an internal standard. The total yield of **4ae** and the ratio of two isomers are 70% and *syn-α*-**4ae**/*anti-α*-**4ae** = 91/9. The *syn-α*-**4ae** is promptly decomposed at the solution in any solvents, which hampered recording any NMR spectrum.
mp 171.2–172.0 °C; **IR** (ATR) 3051 (w), 2960 (m), 1689 (s), 1469 (w), 1373 (m), 1328 (s), 1294 (m), 1204 (m), 1048 (s) cm^{-1}; **Analysis** $C_{36}H_{48}Cl_4Ge_2O_6$ (863.83) Calculated: C, 50.06; H, 5.60, Found: C, 50.27; H, 5.71.

Diethyl (3*E*,4*R,5*R**,8*E*,9*S**,10*S**)-2,2,7,7-tetrachloro-3,8-bis(2,2-dimethylpropylidene)-5,10-di-*p*-tolyl-1,6,2,7-dioxadigermecane-4,9-dicarboxylate *syn-α*-4af**

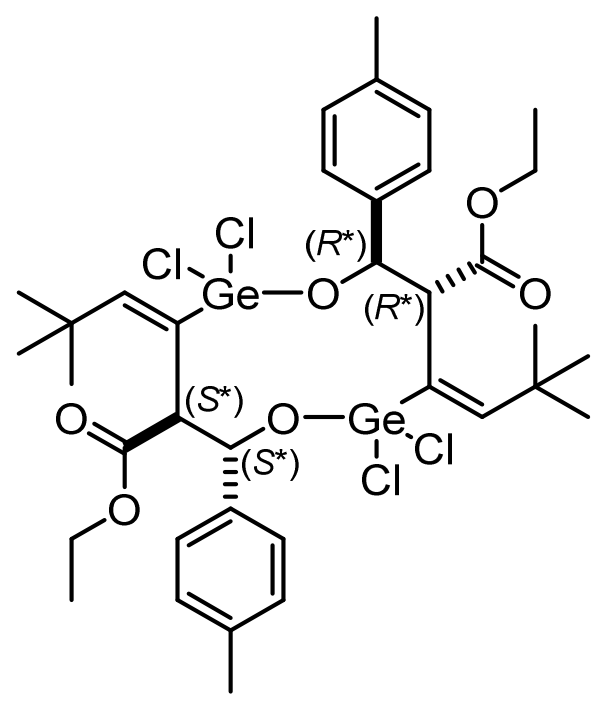

To a mixture of **1a** (0.0252 g, 0.150 mmol), 4-methylbenzaldehyde **3f** (0.0177 mL, 0.150 mmol), and $BF_3 \cdot OEt_2$ (0.0252 mL, 0.100 mmol) in CH_3CN (1.0 mL) was added $GeCl_2$-dioxane (0.0230 g, 0.0993 mmol). After the reaction mixture was left to stand at room temperature for 18 h, the precipitated colorless solid was filtrated and washed with CH_3CN, to give the *syn-α*-**4af** as the enantiomeric dimer (0.0038 g, 9%). The filtrate was evaporated under vaccum, to give *anti-α*-**4af** (5%) determined by ^{1}H NMR using 1,1,2,2-tetrachloroethane as an internal standard. The total yield of **4af** and the ratio of two isomers are 14% and *syn-α*-**4af**/*anti-α*-**4af** = 64/36. The *syn-α*-**4af** is promptly decomposed at the solution in any solvents, which hampered recording any NMR spectrum.
mp 157.8–158.5 °C; **IR** (ATR) 3031 (w), 2966 (m), 1735 (s), 1696 (s), 1469 (w), 1324 (m), 1227 (m), 1051 (s), 799 (m) cm^{-1}; **Analysis** $C_{36}H_{48}Cl_4Ge_2O_6$ (863.83) Calculated: C, 50.06; H, 5.60, Found: C, 50.10; H, 5.72.

Diethyl (3*E*,4*R,5*R**,8*E*,9*S**,10*S**)-2,2,7,7-tetrachloro-3,8-bis(2,2-dimethylpropylidene)-5,10-di(naphthalen-2-yl)-1,6,2,7-dioxadigermecane-4,9-dicarboxylate** *syn*-*α*-**4ag**

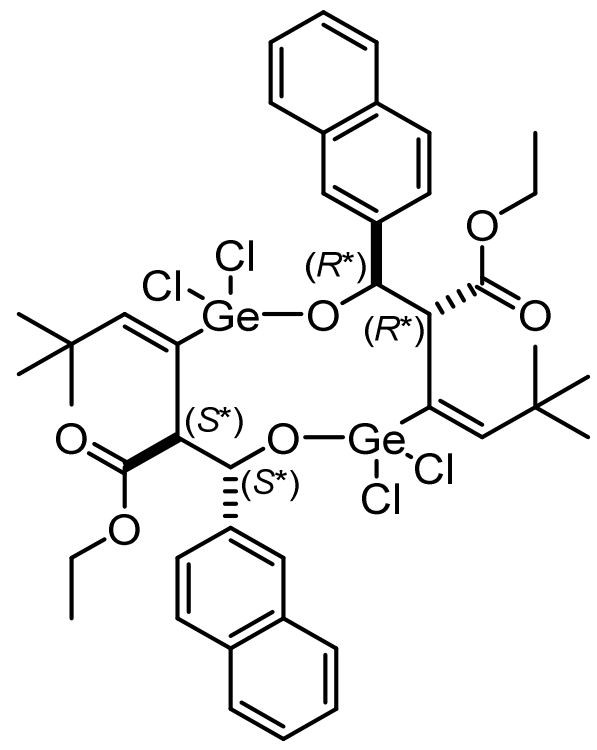

To a mixture of **1a** (0.0252 g, 0.150 mmol), 2-naphthaldehyde **3g** (0.0234 g, 0.150 mmol), and $BF_3 \cdot OEt_2$ (0.0252 mL, 0.100 mmol) in CH_3CN (1.0 mL) was added $GeCl_2$-dioxane (0.0231 g, 0.0997 mmol). After the reaction mixture was left to stand at room temperature for 18 h, the precipitated colorless solid was filtrated and washed with CH_3CN, to give the *syn*-*α*-**4ag** as the enantiomeric dimer (0.0290 g, 64%). The filtrate was evaporated under vaccum, to give *anti*-*α*-**4ag** (6%) determined by ^1H NMR using 1,1,2,2-tetrachloroethane as an internal standard. The total yield of **4ag** and the ratio of two isomers are 70% and *syn*-*α*-**4ag**/*anti*-*α*-**4ag** = 91/9. The *syn*-*α*-**4ag** is promptly decomposed at the solution in any solvents, which hampered recording any NMR spectrum.

mp 155.1–156.0 °C; **IR** (ATR) 3046 (w), 2964 (m), 1686 (s), 1374 (m), 1327 (s), 1285 (m), 1204 (m), 1064 (s), 1018 (m) cm^{-1}; **Analysis** $C_{42}H_{48}Cl_4Ge_2O_6$ (935.90) Calculated: C, 53.90; H, 5.17, Found: C, 53.78; H, 5.19.

Diethyl (3*E*,4*R,5*R**,8*E*,9*S**,10*S**)-2,2,7,7-tetrachloro-3,8-bis(2,2-dimethylpropylidene)-5,10-di(naphthalen-1-yl)-1,6,2,7-dioxadigermecane-4,9-dicarboxylate** *syn*-*α*-**4ah**

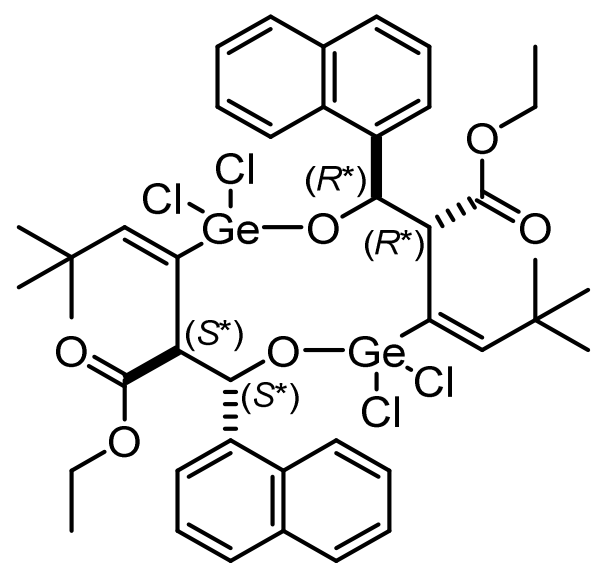

To a mixture of **1a** (0.0250 g, 0.149 mmol), 1-naphthaldehyde **3h** (0.0204 mL, 0.150 mmol), and $BF_3 \cdot OEt_2$ (0.0252 mL, 0.100 mmol) in CH_3CN (1.0 mL) was added $GeCl_2$-dioxane (0.0230 g, 0.0993 mmol). After the reaction mixture was left to stand at room temperature for 18 h, the precipitated colorless solid was filtrated and washed with CH_3CN, to give the *syn*-*α*-**4ah** as the enantiomeric dimer (0.0277 g, 60%). The *syn*-*α*-**4ah** is promptly decomposed at the solution in any solvents, which hampered recording any NMR spectrum.

mp 186.0–186.6 °C; **IR** (ATR) 3065 (w), 2960 (m), 1696 (s), 1373 (m), 1329 (m), 1284 (m), 1203 (m), 1060 (s), 1002 (m) cm^{-1}; **Analysis** $C_{42}H_{48}Cl_4Ge_2O_6$ (935.90) Calculated: C, 53.90; H, 5.17, Found: C, 53.81; H, 5.19.

3-3. *anti-α*-Selective aldol reaction of allenoate 1a with aldehyde 3 using $GeCl_2$-dioxane

Ethyl (4*R,5*S**,*E*)-2,2-dichloro-3-(2,2-dimethylpropylidene)-5-phenyl-1,2-oxagermolane-4-carboxylate** *anti-α*-**4aa**

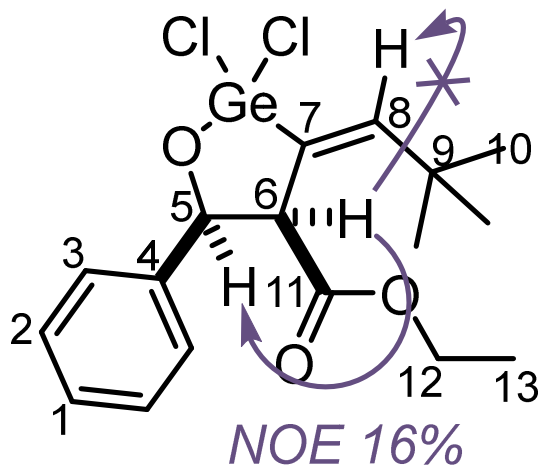

To a mixture of **1a** (0.0253 g, 0.150 mmol) and benzaldehyde **3a** (0.0202 mL, 0.200 mmol) in EtOAc (1.0 mL) was added $GeCl_2$-dioxane (0.0231 g, 0.0997 mmol). After the reaction mixture was stirred at room temperature for 18 h, the solvent was removed under vacuum. The yield was determined by ^{1}H NMR of the crude product using 1,1,2,2-tetrachloroethane as an internal standard (84%, *anti*-*α*-**4aa**/*anti*-*γ*-**4aa** = 93/7, single diastereomer).

To obtain the spectrodata of the isolated product, recrystallization from hexane was performed to give *anti*-*α*-**4aa** in pure form as a colorless solid.

mp 117.1–117.8 °C; **IR** (KBr) 3032 (w), 2963 (m), 1717 (s), 1459 (m), 1370 (m), 1185 (m), 1030 (m), 867 (m) cm^{-1}; **^{1}H NMR** (400 MHz, $CDCl_3$) 7.42–7.27 (m, 5H), 6.36 (d, *J* = 1.6 Hz, 1H, 8-H), 5.22 (d, *J* = 4.8 Hz, 1H, 5-H), 4.18 (dd, *J* = 5.0, 1.4 Hz, 1H, 6-H), 3.81 (dq, *J* = 10.8, 7.2 Hz, 1H, 12-H), 3.73 (dq, *J* = 10.8, 7.2 Hz, 1H, 12-H), 1.20 (s, 9H, 10-H), 0.90 (t, *J* = 7.2 Hz, 3H, 13-H); **^{13}C{^{1}H} NMR** (100 MHz, $CDCl_3$) 169.7 (s, C-11), 156.9 (d, C-8), 138.6 (s, C-4), 132.3 (s, C-7), 128.4 (d, C-2), 128.3 (d, C-1), 125.6 (d, C-3), 79.4 (d, C-5), 61.2 (t, C-12), 53.2 (d, C-6), 36.7 (s, C-9), 30.0 (q, C-10), 13.8 (q, C-13); **HRMS** (DART$^+$) Calculated ($C_{17}H_{23}O_3Cl_2Ge$): 419.0231 ($[M+H]^+$), Found: 419.0230 ($[M+H]^+$).

Large-scale synthesis

To a mixture of **1a** (0.253 g, 1.50 mmol) and benzaldehyde **3a** (0.202 mL, 2.00 mmol) in EtOAc (5.0 mL) was added $GeCl_2$-dioxane (0.232 g, 1.00 mmol). After the reaction mixture was stirred at room temperature for 18 h, the solvent was removed under vacuum. The yield was determined by ^{1}H NMR of the crude product using 1,1,2,2-tetrachloroethane as an internal standard (63%, *anti*-*α*-**4aa**/*anti*-*γ*-**4aa** = 78/22, single diastereomer).

^{1}**H NMR** (400 MHz, $CDCl_3$)

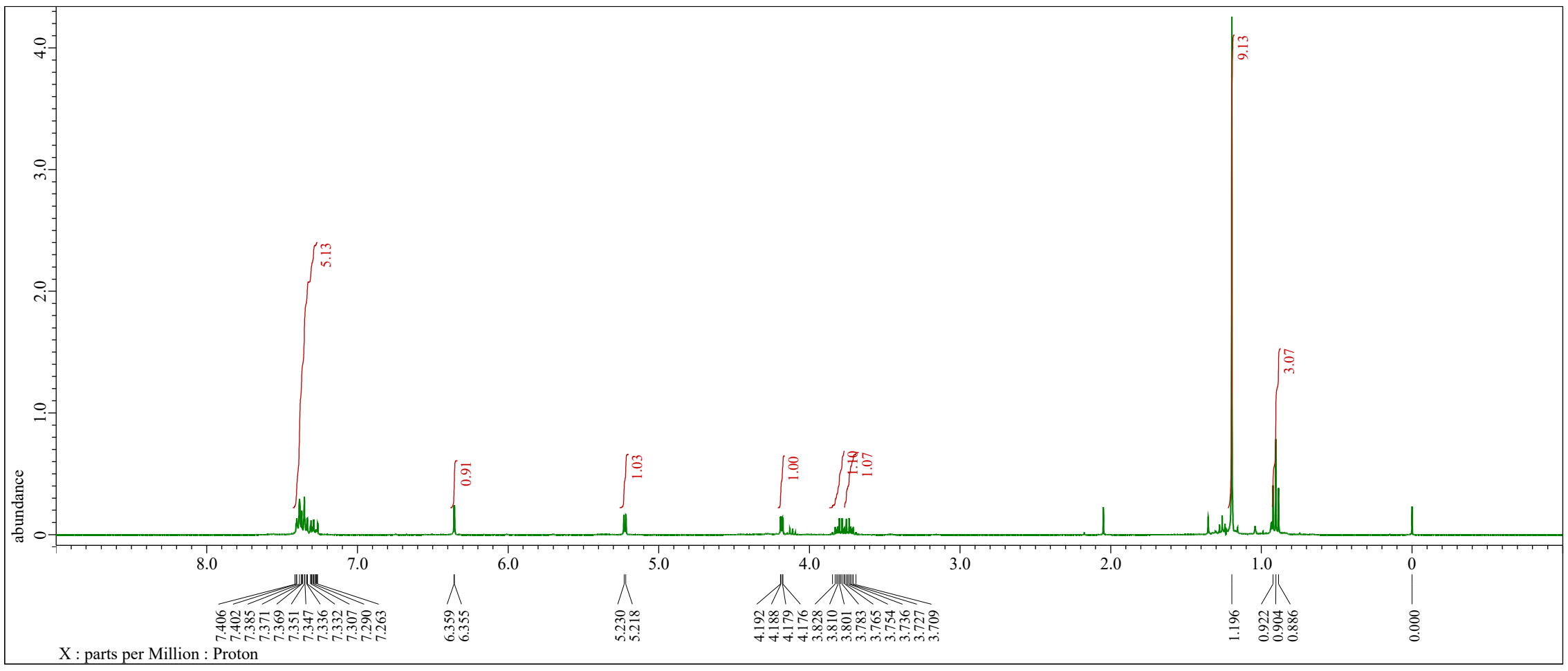

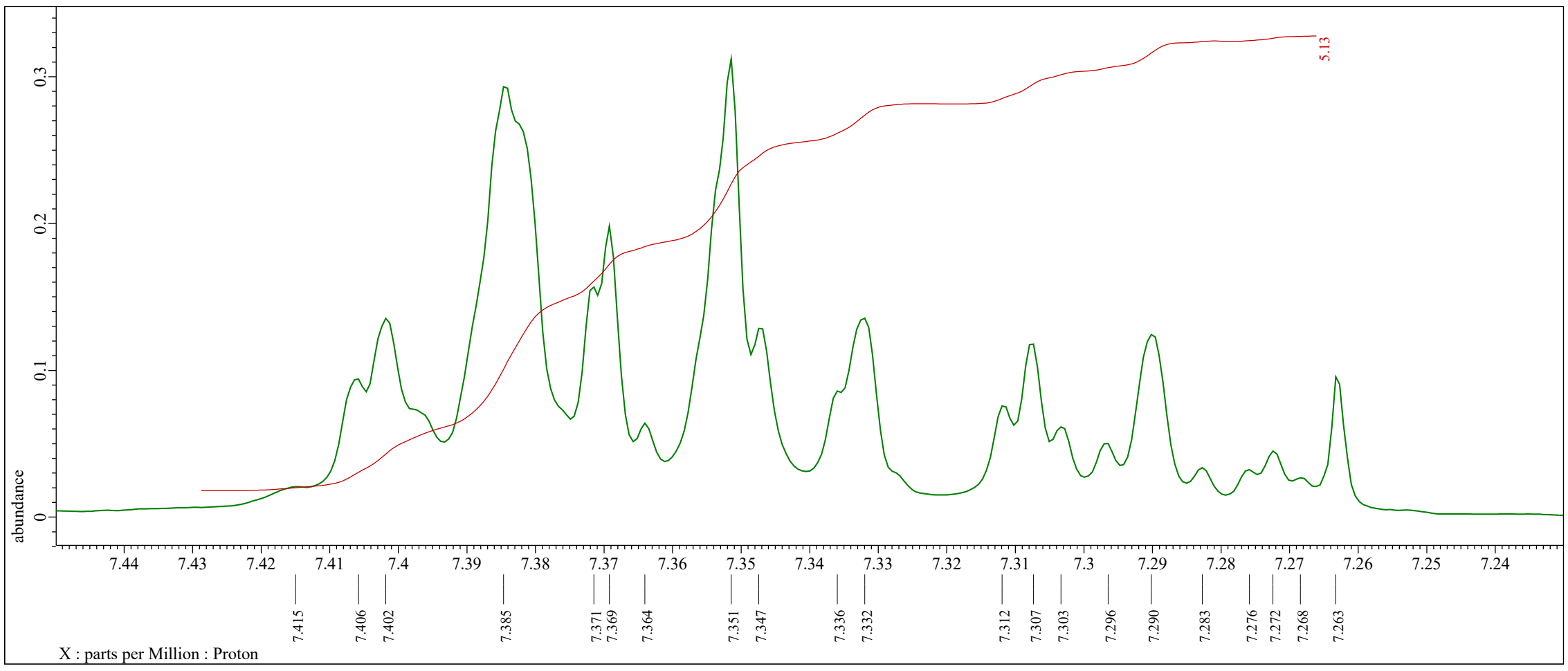

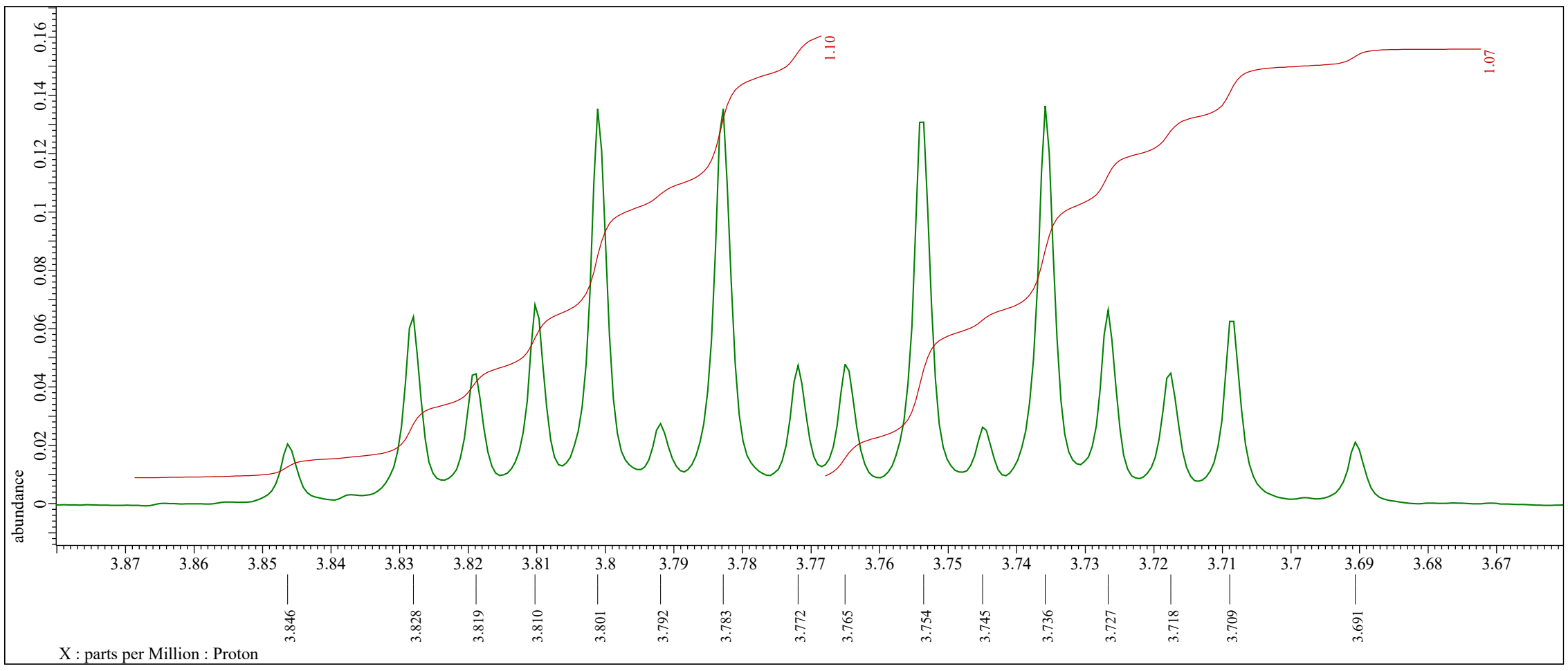

^{13}C{^{1}H} NMR (100 MHz, $CDCl_3$)

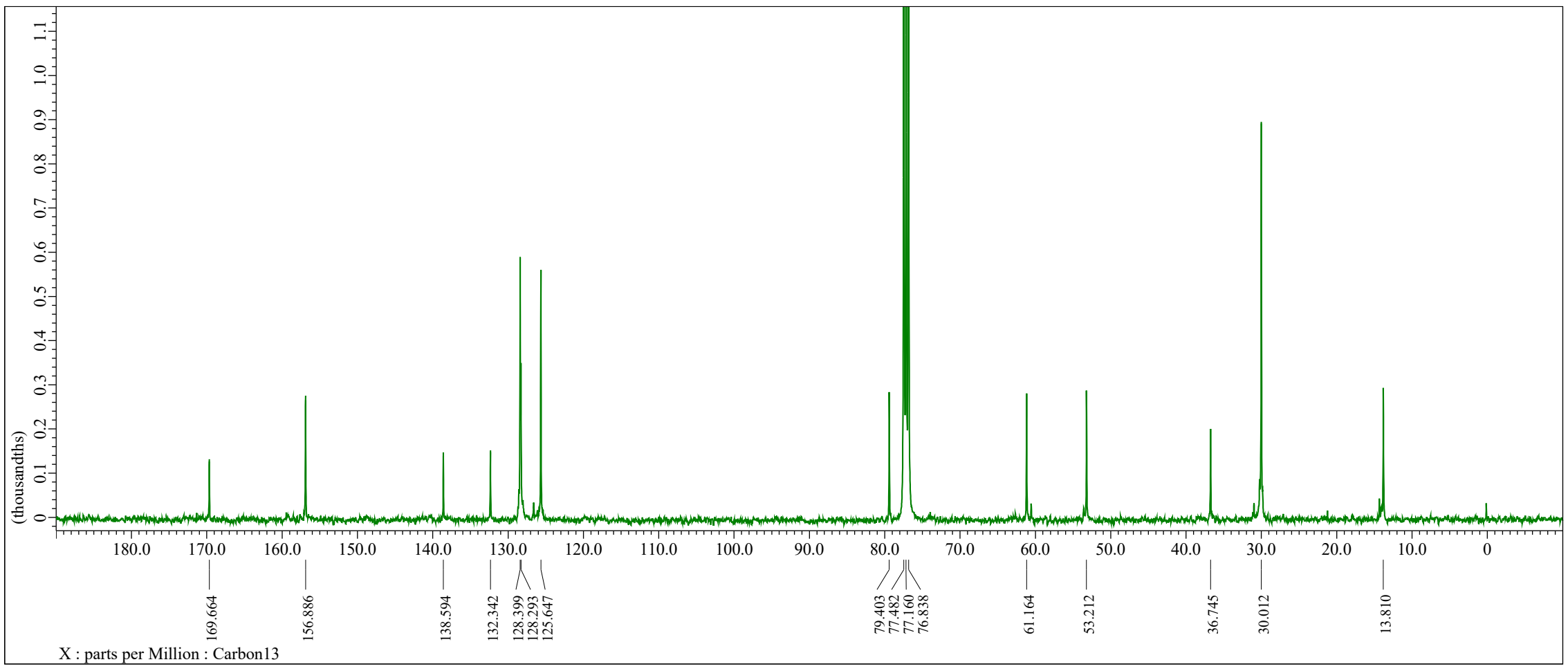

Ethyl (4*R,5*S**,*E*)-2,2-dichloro-3-(2,2-dimethylpropylidene)-5-(4-methoxyphenyl)-1,2-oxagermolane-4-carboxylate *anti*-*α*-4ab**

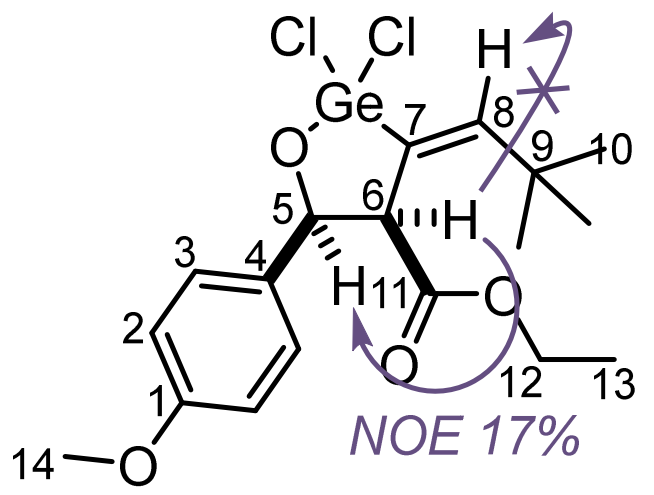

To a mixture of **1a** (0.0252 g, 0.150 mmol) and 4-methoxybenzaldehyde **3b** (0.0243 mL, 0.200 mmol) in EtOAc (1.0 mL) was added $GeCl_2$-dioxane (0.0232 g, 0.100 mmol). After the reaction mixture was stirred at room temperature for 18 h, the solvent was removed under vacuum. The yield was determined by ^{1}H NMR of the crude product using 1,1,2,2-tetrachloroethane as an internal standard (85%, *anti*-*α*-**4ab**/*anti*-*γ*-**4ab** = 95/5, single diastereomer).

To obtain the spectrodata of the isolated product, some conditions were modified. To a mixture of **1a** (1.1 eq) and 4-methoxybenzaldehyde **3b** (0.095 eq) in EtOAc (1.0 mL) was added $GeCl_2$-dioxane (1.0 eq). After the reaction mixture was stirred at room temperature for 18 h, the solvent was removed under vacuum. Careful extraction of the crude mixture with hexane gave *anti*-*α*-**4ab** in pure form as colorless neat.

IR (KBr) 3044 (w), 2962 (m), 1717 (m), 1514 (s), 1468 (m), 1251 (s), 1179 (m), 1035 (m), 832 (m) cm^{-1}; **^{1}H NMR** (400 MHz, $CDCl_3$) 7.31 (d, J = 8.8 Hz, 2H, 3-H), 6.88 (d, J = 8.4 Hz, 2H, 2-H), 6.34 (d, J = 1.2 Hz, 1H, 8-H), 5.18 (d, J = 4.8 Hz, 1H, 5-H), 4.14 (dd, J = 4.8, 1.6 Hz, 1H, 6-H), 3.85 (dq, J = 10.8, 7.6 Hz, 1H, 12-H), 3.80 (s, 3H, 14-H), 3.79 (dq, J = 10.8, 7.2 Hz, 1H, 12-H), 1.19 (s, 9H, 10-H), 0.97 (t, J = 7.4 Hz, 3H, 13-H); **^{13}C{^{1}H} NMR** (100 MHz, $CDCl_3$) 169.7 (s, C-11), 159.6 (s, C-1), 156.8 (d, C-8), 132.5 (s, C-7), 130.7 (s, C-4), 126.9 (d, C-3), 113.8 (d, C-2), 79.2 (d, C-5), 61.2 (t, C-12), 55.4 (q, C-14), 53.4 (d, C-6), 36.8 (s, C-9), 30.0 (q, C-10), 13.9 (q, C-13); **HRMS** (DART$^+$) Calculated ($C_{18}H_{25}O_4Cl_2Ge$): 449.0336 ([M+H]$^+$), Found: 449.0356 ([M+H]$^+$).

^{1}H NMR (400 MHz, $CDCl_3$)

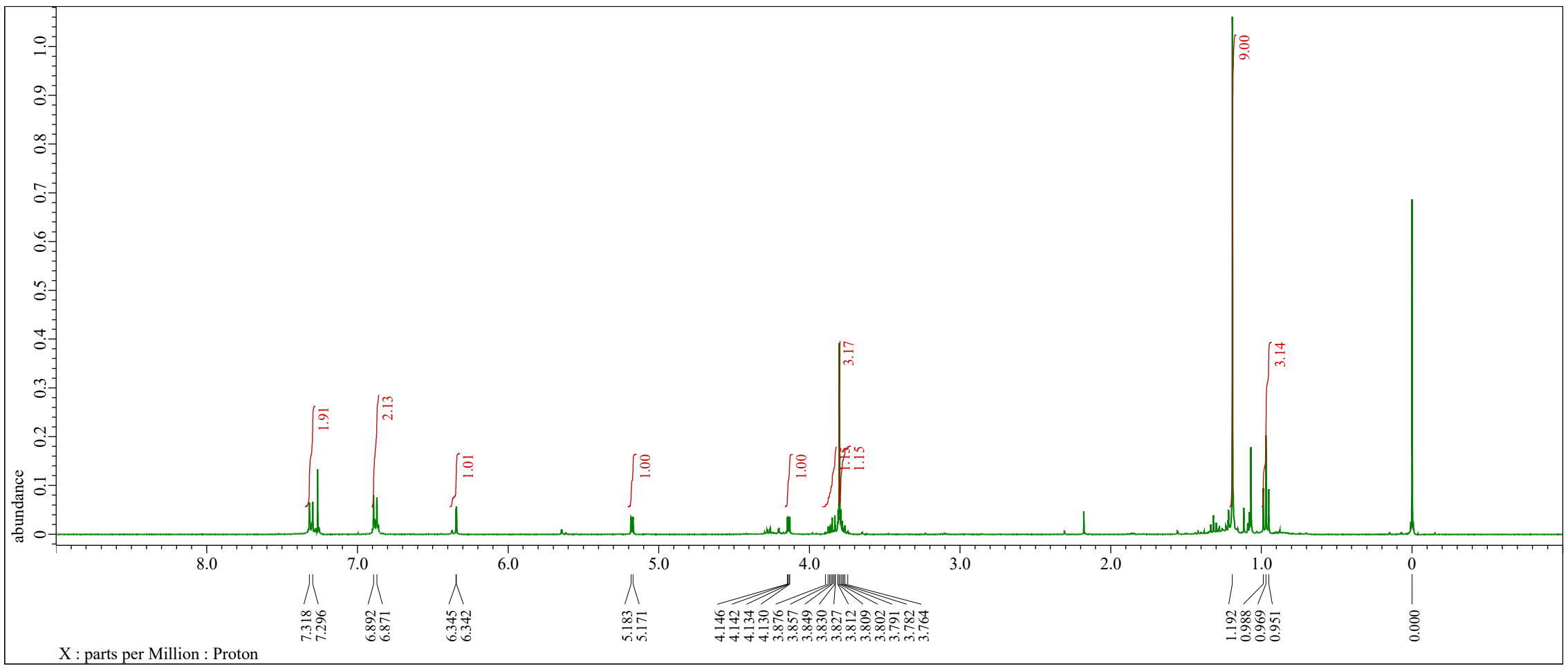

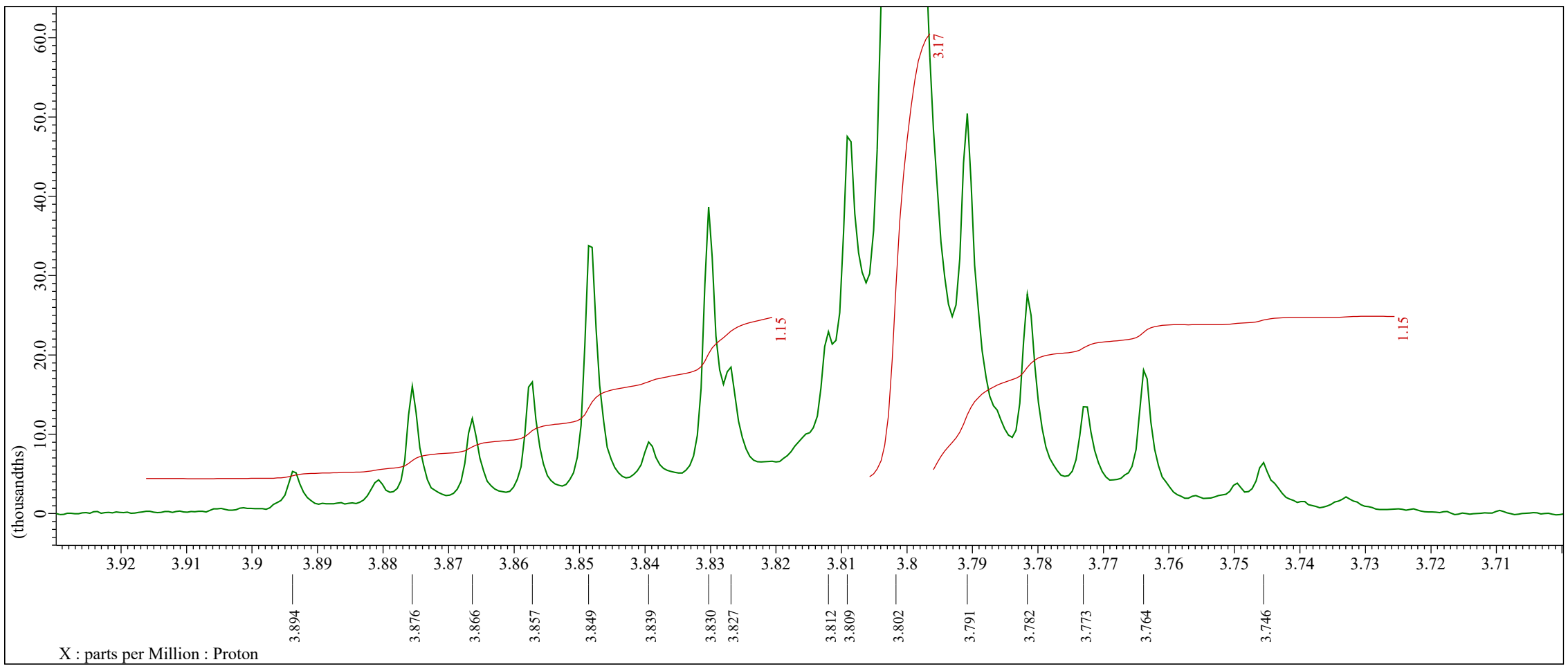

^{13}C{^{1}H} NMR (100 MHz, $CDCl_3$)

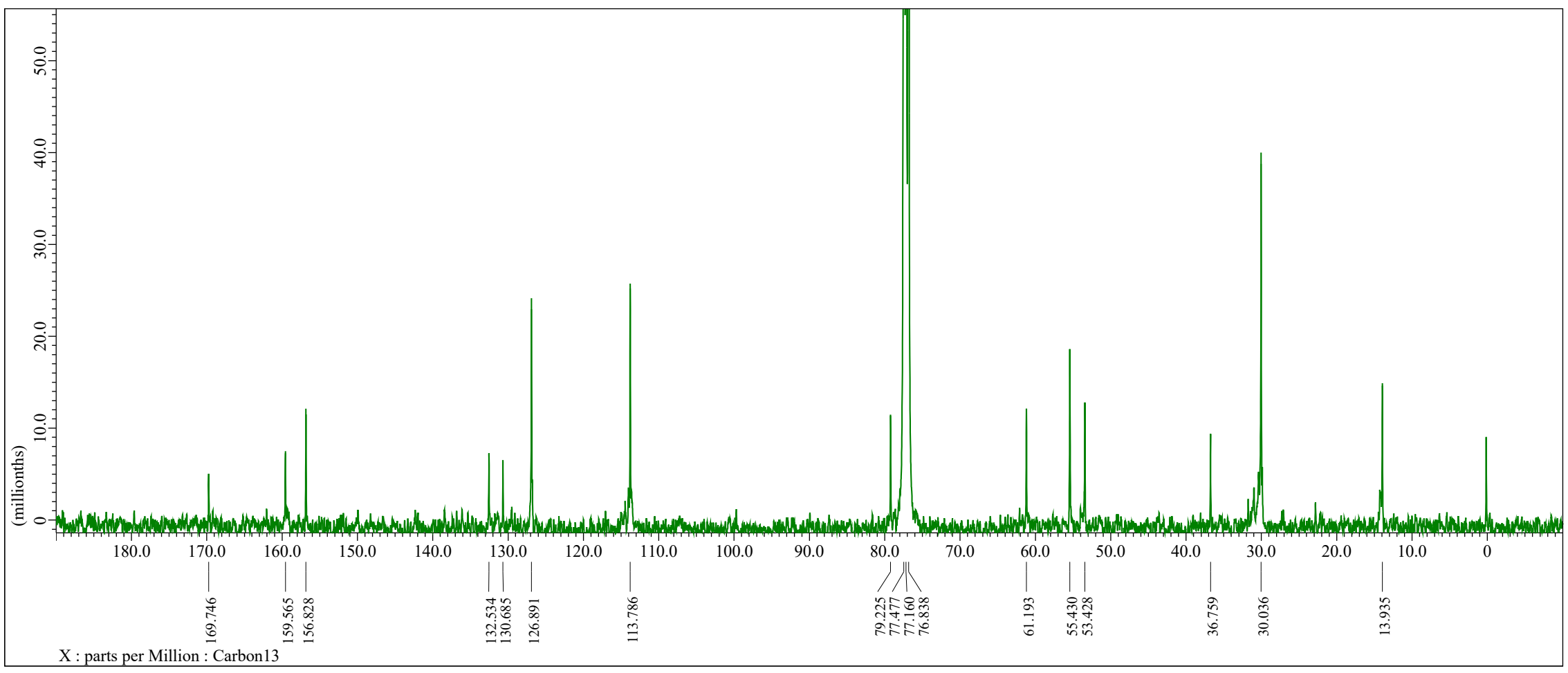

Ethyl (4*R,5*S**,*E*)-2,2-dichloro-3-(2,2-dimethylpropylidene)-5-{4-(methoxycarbonyl)phenyl}-1,2-oxagermolane-4-carboxylate *anti*-*α*-4ac**

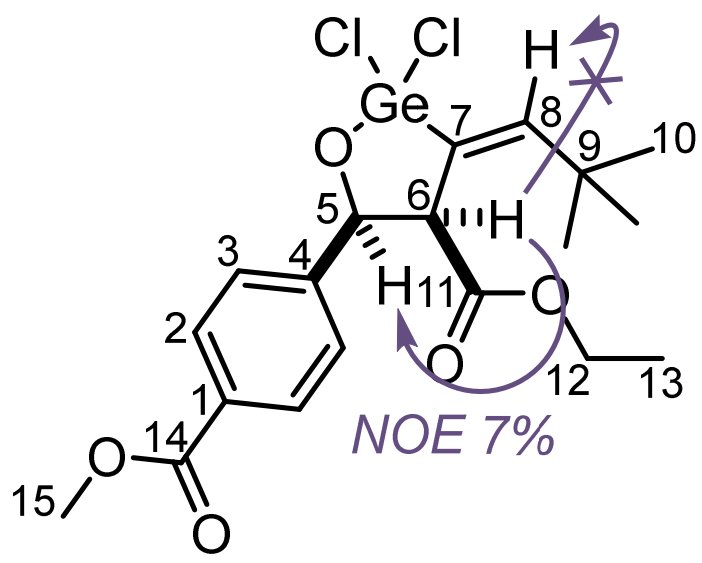

To a mixture of **1a** (0.0252 g, 0.150 mmol) and methyl 4-formylbenzoate **3c** (0.0328 g, 0.200 mmol) in EtOAc (1.0 mL) was added $GeCl_2$-dioxane (0.0231 g, 0.0997 mmol). After the reaction mixture was stirred at room temperature for 18 h, the solvent was removed under vacuum. The yield was determined by ^{1}H NMR of the crude product using 1,1,2,2-tetrachloroethane as an internal standard (64%, *anti*-*α*-**4ac**/*anti*-*γ*-**4ac** = 82/18, single diastereomer).

To obtain the spectrodata of the isolated product, some conditions were modified. To a mixture of **1a** (1.1 eq) and methyl 4-formylbenzoate **3c** (0.095 eq) in EtOAc (1.0 mL) was added $GeCl_2$-dioxane (1.0 eq). After the reaction mixture was stirred at room temperature for 18 h, the solvent was removed under vacuum. Careful extraction of the crude mixture with hexane gave *anti*-*α*-**4ac** in pure form as a colorless solid.

mp 142.0–143.0 °C; **IR** (KBr) 3064 (w), 2958 (m), 1724 (s), 1438 (m), 1369 (m), 1281 (s), 1183 (m), 1110 (m), 863 (m) cm^{-1}; **^{1}H NMR** (400 MHz, $CDCl_3$) 8.03 (d, *J* = 8.4 Hz, 2H, 2-H), 7.49 (d, *J* = 8.0 Hz, 2H, 3-H), 6.37 (d, *J* = 1.2 Hz, 1H, 8-H), 5.26 (d, *J* = 4.8 Hz, 1H, 5-H), 4.23 (dd, *J* = 4.8, 1.6 Hz, 1H, 6-H), 3.92 (s, 3H, 15-H), 3.81 (dq, *J* = 11.0, 7.4 Hz, 1H, 12-H), 3.72 (dq, *J* = 10.8, 7.2 Hz, 1H, 12-H), 1.20 (s, 9H, 10-H), 0.91 (t, *J* = 7.2 Hz, 3H, 13-H); **^{13}C{^{1}H} NMR** (100 MHz, $CDCl_3$) 169.2 (s, C-11), 166.9 (s, C-14), 157.2 (d, C-8), 143.8 (s, C-4), 131.8 (s, C-7), 130.0 (s, C-1), 129.7 (d, C-2), 125.8 (d, C-3), 78.9 (d, C-5), 61.3 (t, C-12), 52.8 (d, C-6), 52.3 (q, C-15), 36.8 (s, C-9), 30.0 (q, C-10), 13.8 (q, C-13); **HRMS** (DART$^+$) Calculated ($C_{19}H_{25}O_5Cl_2Ge$): 477.0285 ([M+H]$^+$), Found: 477.0267 ([M+H]$^+$).

^{1}H NMR (400 MHz, $CDCl_3$)

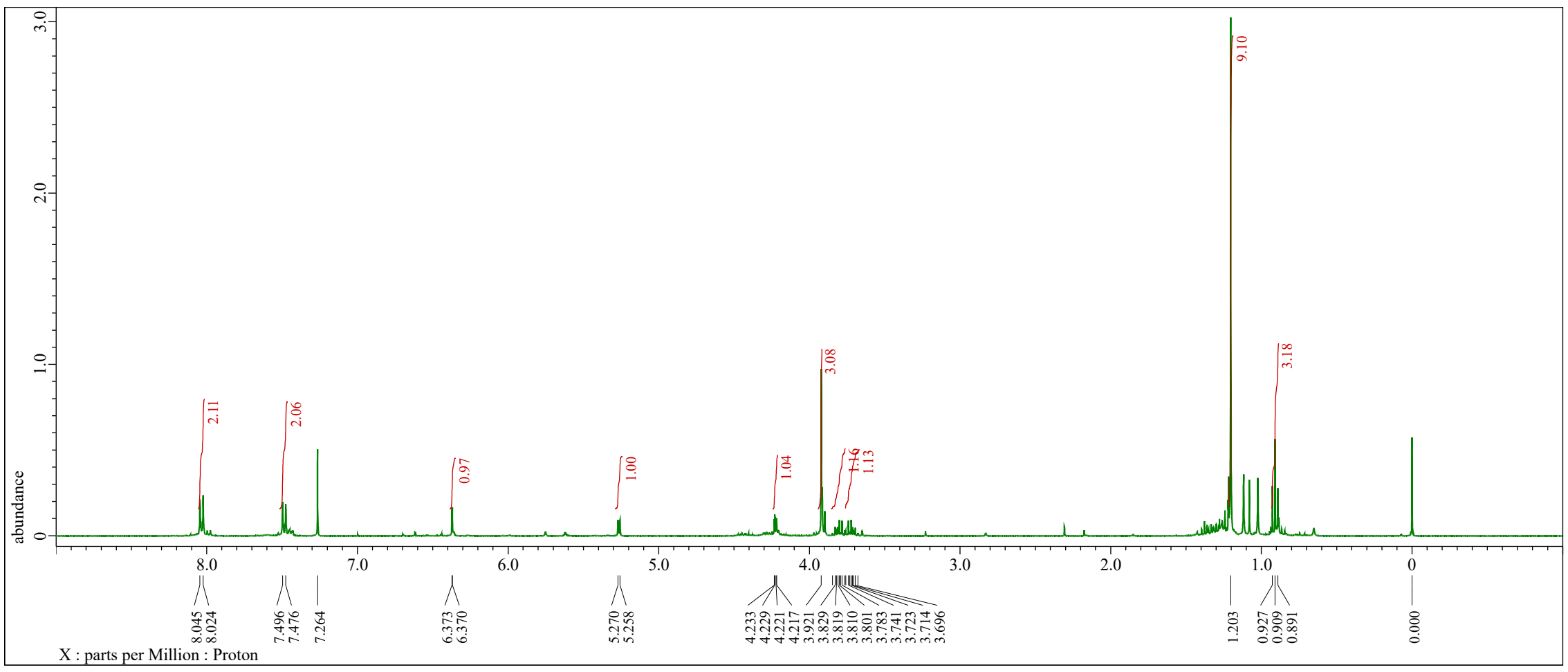

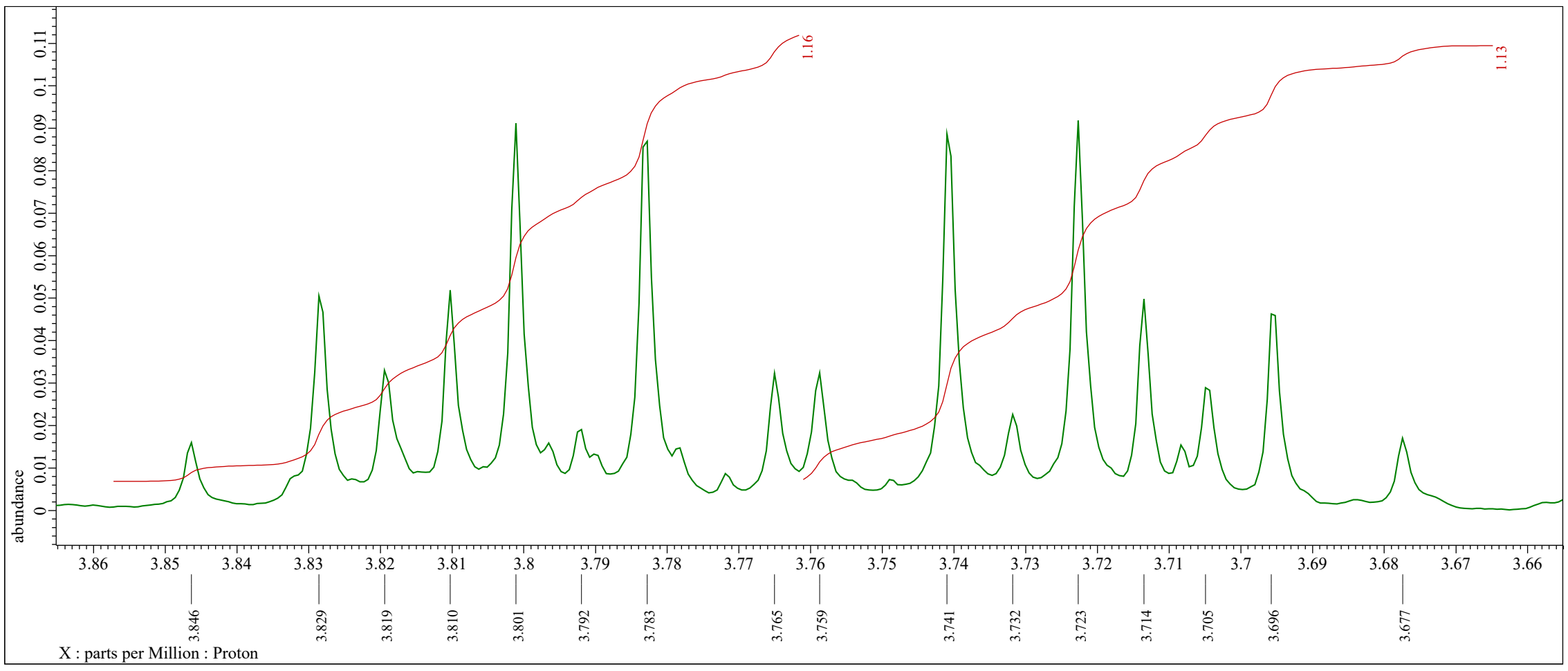

$^{13}C\{^{1}H\}$ **NMR** (100 MHz, $CDCl_3$)

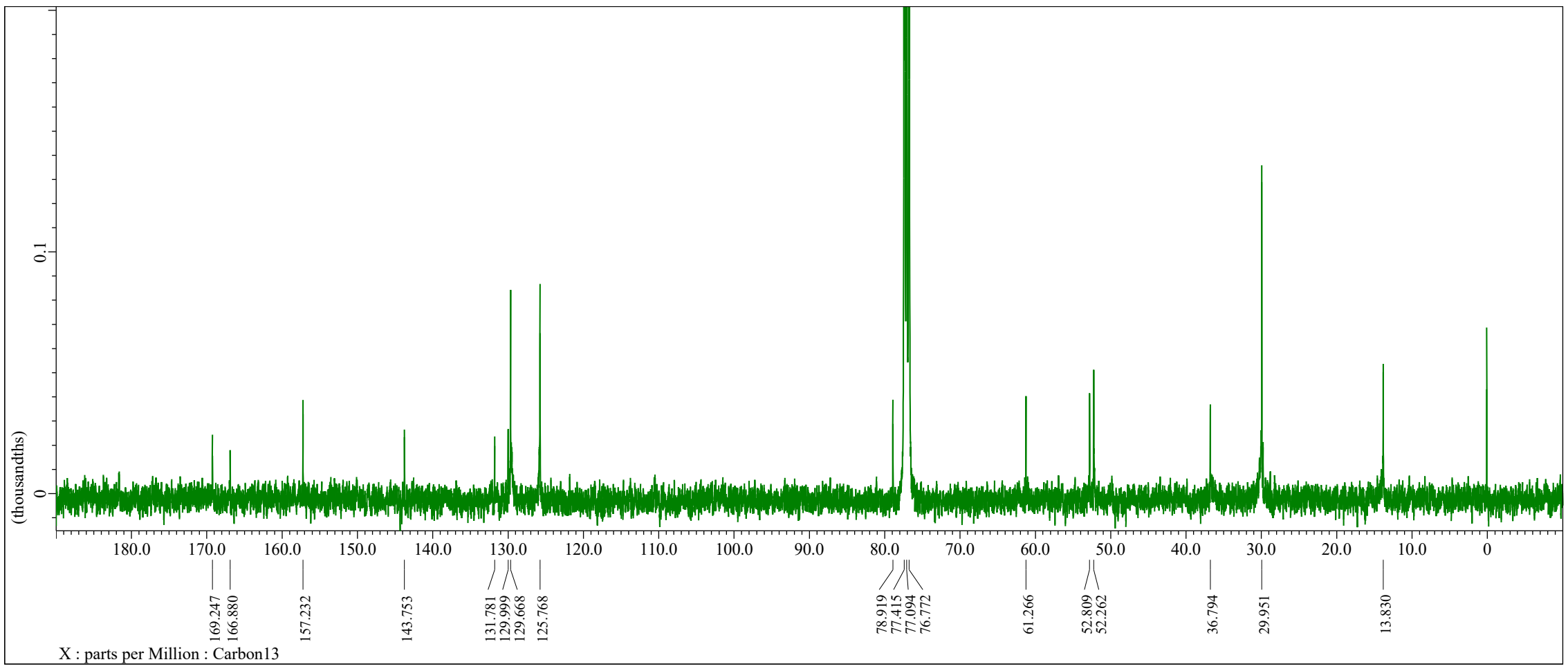

Ethyl (4*R,5*S**,*E*)-2,2-dichloro-3-(2,2-dimethylpropylidene)-5-(*o*-tolyl)-1,2-oxagermolane-4-carboxylate** *anti-α*-**4ad**

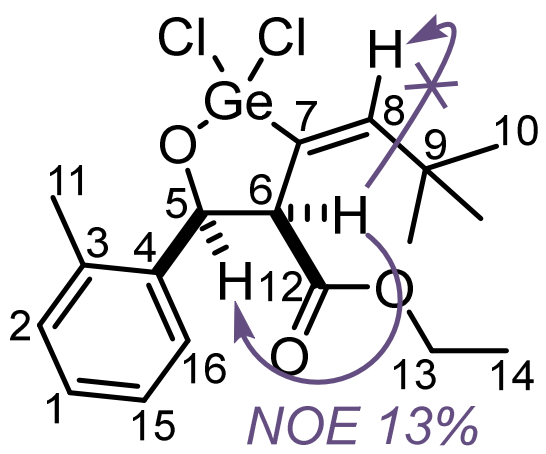

To a mixture of **1a** (0.0251 g, 0.149 mmol) and 2-methylbenzaldehyde **3d** (0.0231 mL, 0.200 mmol) in EtOAc (1.0 mL) was added $GeCl_2$-dioxane (0.0233 g, 0.101 mmol). After the reaction mixture was stirred at room temperature for 18 h, the solvent was removed under vacuum. The yield was determined by ^{1}H NMR of the crude product using 1,1,2,2-tetrachloroethane as an internal standard (76%, *anti-α*-**4ad**/*anti-γ*-**4ad** = 93/7, single diastereomer).

To obtain the spectrodata of the isolated product, some conditions were modified. To a mixture of **1a** (1.0 eq) and 2-methylbenzaldehyde **3d** (1.0 eq) in EtOAc (1.0 mL) was added $GeCl_2$-dioxane (1.0 eq). After the reaction mixture was stirred at room temperature for 18 h, the generated white solid was filtered off with hexane. The filtrate was

evaporated to give *anti*-α-**4ad** in pure form as colorless neat.

IR (KBr) 3065 (w), 2963 (m), 1720 (s), 1464 (m), 1370 (m), 1330 (m), 1180 (s), 1030 (m), 865 (m) cm^{-1}; **^{1}H NMR** (400 MHz, $CDCl_3$) 7.57–7.55 (m, 1H), 7.21–7.13 (m, 3H), 6.38 (d, *J* = 1.6 Hz, 1H, 8-H), 5.35 (d, *J* = 4.8 Hz, 1H, 5-H), 4.28 (dd, *J* = 5.0, 1.4 Hz, 1H, 6-H), 3.77 (dq, *J* = 10.8, 7.2 Hz, 1H, 13-H), 3.68 (dq, *J* = 11.0, 7.0 Hz, 1H, 13-H), 2.34 (s, 3H, 11-H), 1.19 (s, 9H, 10-H), 0.88 (t, *J* = 7.2 Hz, 3H, 14-H); **^{13}C{^{1}H} NMR** (100 MHz, $CDCl_3$) 169.6 (s, C-12), 156.9 (d, C-8), 136.7 (s), 133.9 (s), 132.5 (s), 130.2 (d), 128.0 (d), 126.2 (d), 125.4 (d), 76.8 (d, C-5), 61.1 (t, C-13), 50.3 (d, C-6), 36.6 (s, C-9), 30.0 (q, C-10), 19.1 (q, C-11), 13.8 (q, C-14); **HRMS** (DART^{+}) Calculated ($C_{18}H_{25}O_3Cl_2Ge$): 433.0387 ($[M+H]^{+}$), Found: 433.0375 ($[M+H]^{+}$).

^{1}H NMR (400 MHz, $CDCl_3$)

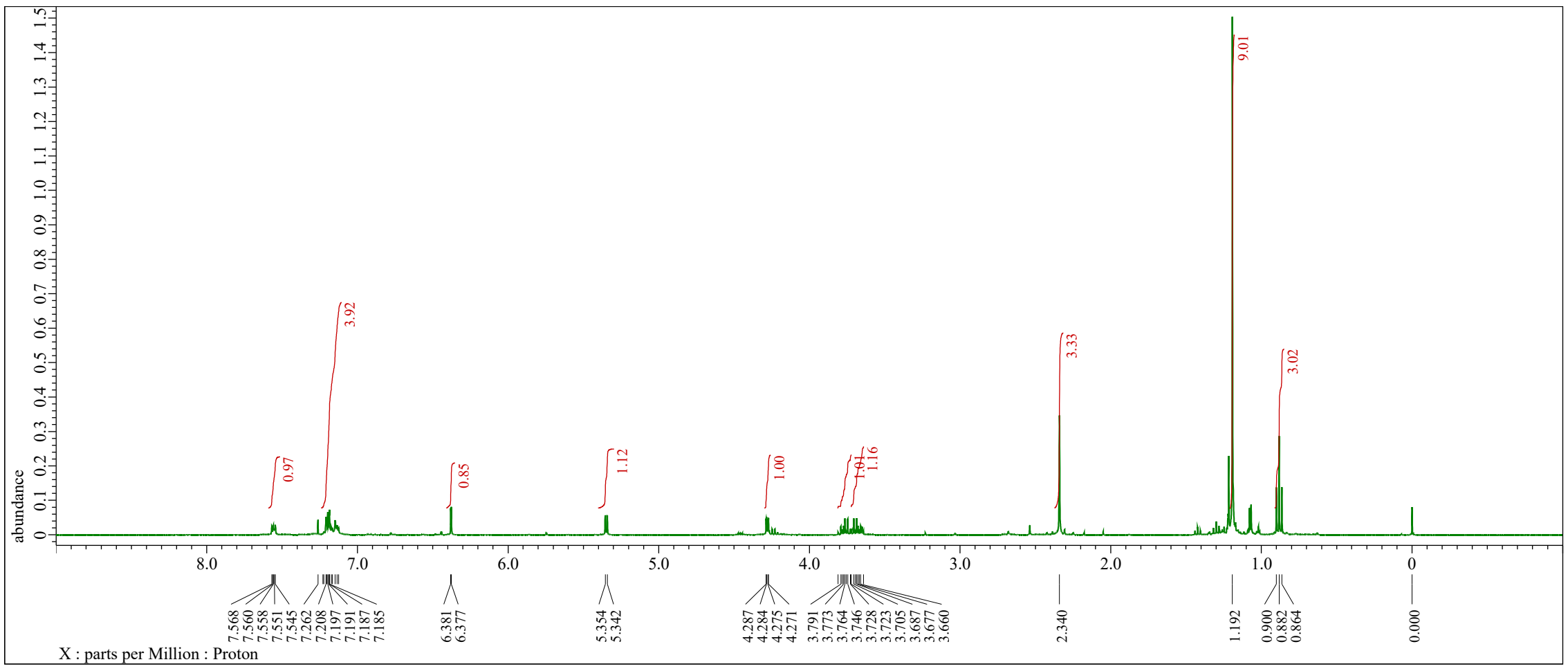

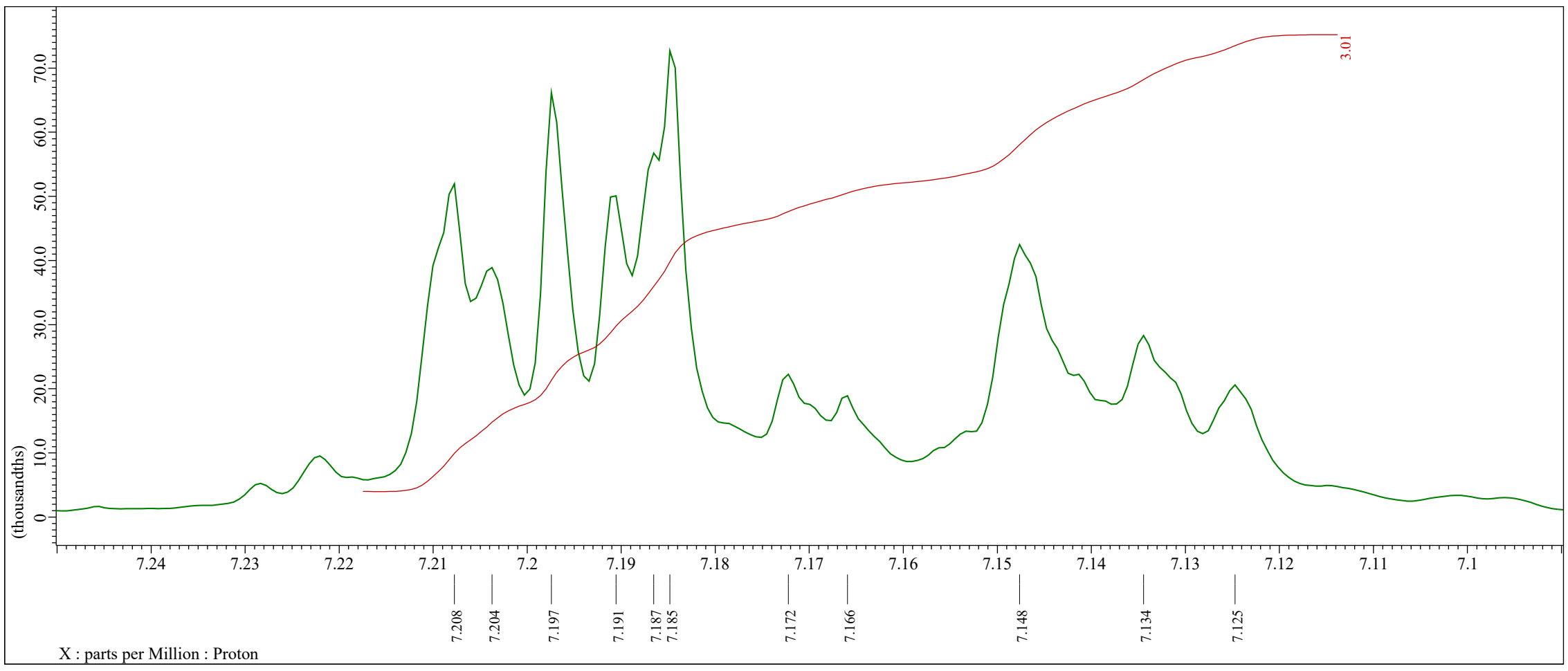

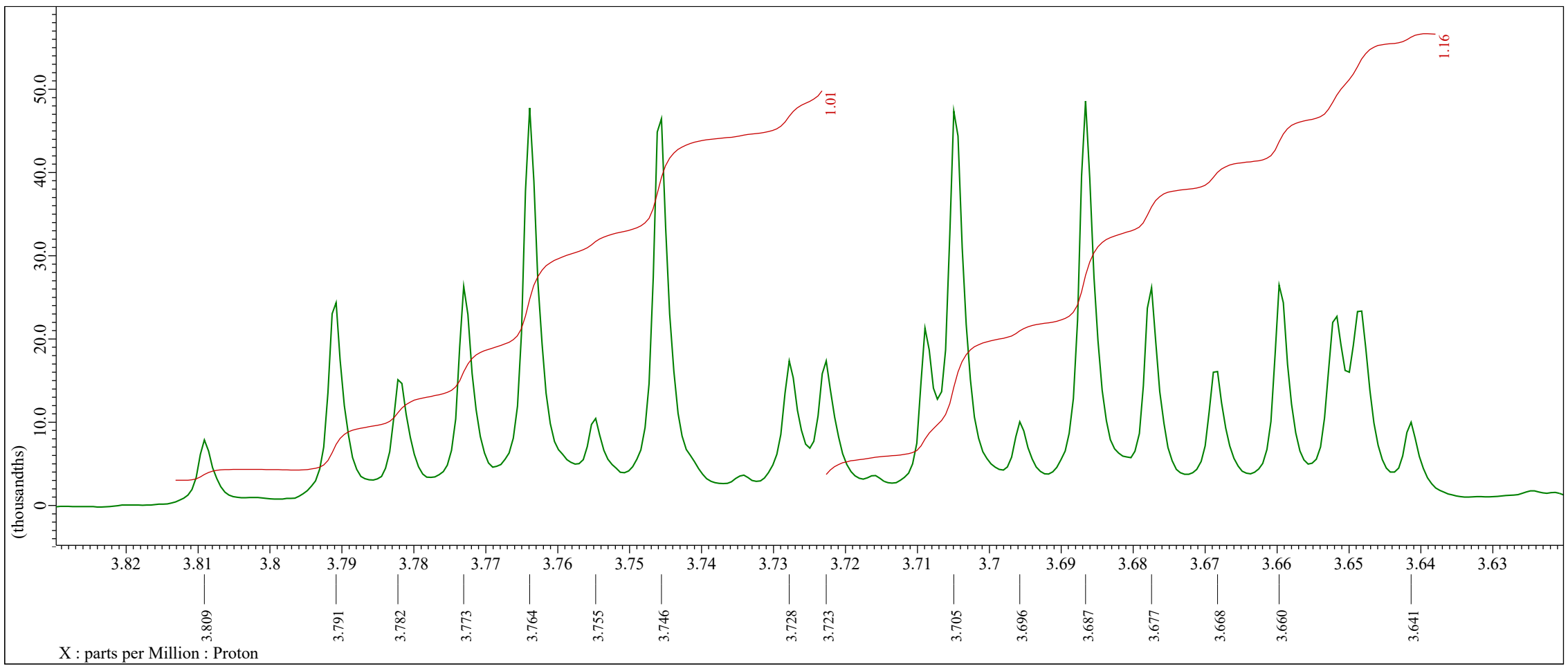

$^{13}C\{^1H\}$ NMR (100 MHz, $CDCl_3$)

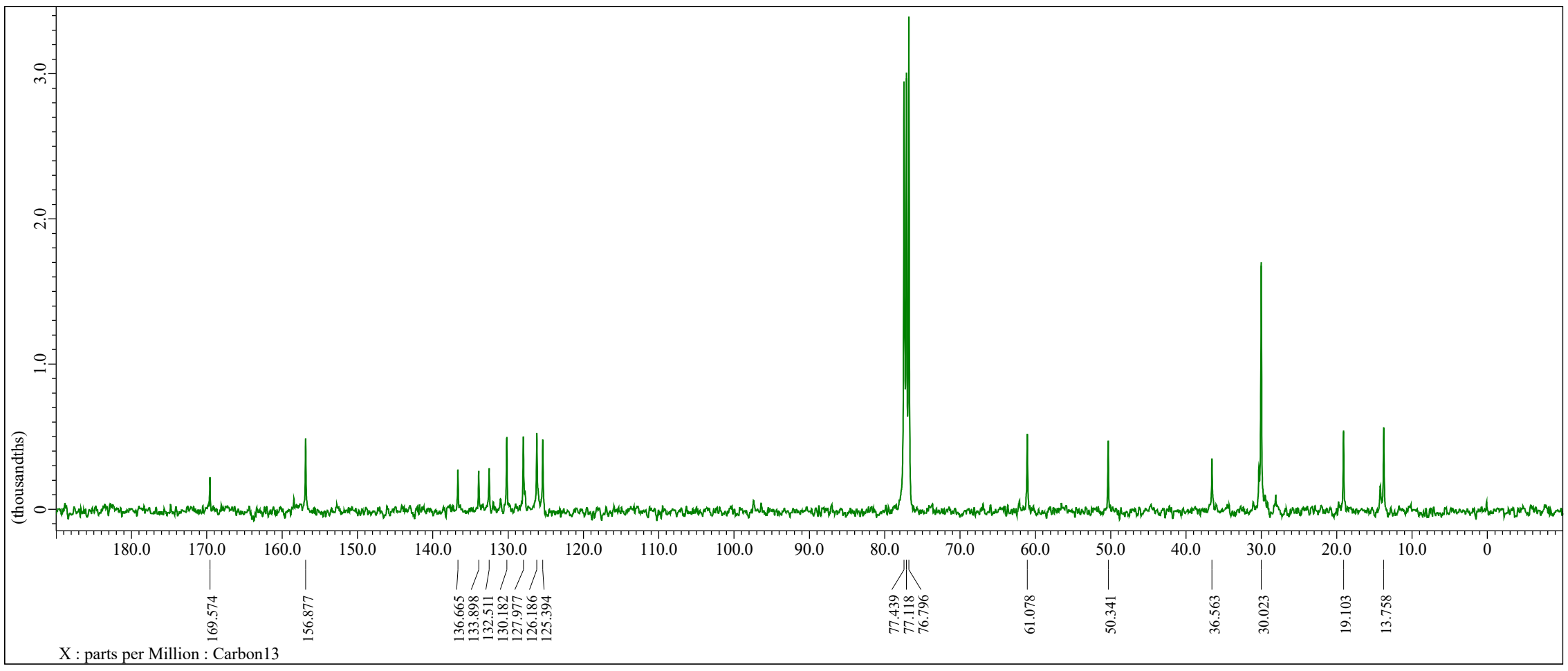

Ethyl (4*R,5*S**,*E*)-2,2-dichloro-3-(2,2-dimethylpropylidene)-5-(*m*-tolyl)-1,2-oxagermolane-4-carboxylate** *anti*-*α*-**4ae**

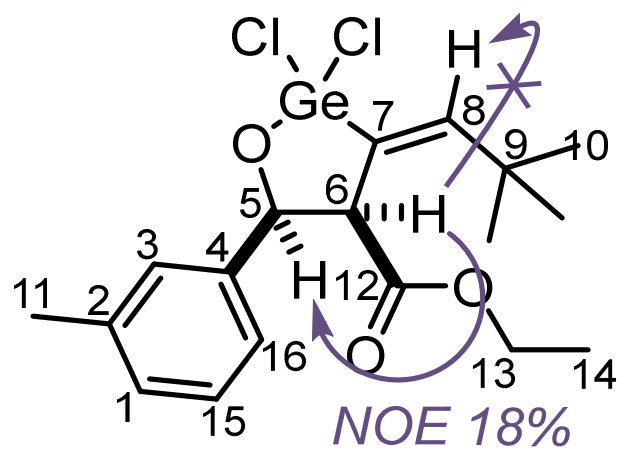

To a mixture of **1a** (0.0252 g, 0.150 mmol) and 3-methylbenzaldehyde **3e** (0.0236 mL, 0.200 mmol) in EtOAc (1.0 mL) was added $GeCl_2$-dioxane (0.0232 g, 0.100 mmol). After the reaction mixture was stirred at room temperature for 18 h, the solvent was removed under vacuum. The yield was determined by ^1H NMR of the crude product using 1,1,2,2-tetrachloroethane as an internal standard (72%, *anti*-*α*-**4ae**/*anti*-*γ*-**4ae** = 93/7, single diastereomer).

To obtain the spectrodata of the isolated product, some conditions were modified. To a mixture of **1a** (1.0 eq) and 3-methylbenzaldehyde **3e** (1.0 eq) in EtOAc (1.0 mL) was added $GeCl_2$-dioxane (1.0 eq). After the reaction mixture was stirred at room temperature for 16 h, the generated white solid was filtered off with hexane. The filtrate was

evaporated to give *anti*-*α*-**4ae** in pure form as colorless neat.

IR (KBr); 3102 (w), 2963 (s), 1719 (s), 1469 (m), 1370 (m), 1326 (m), 1186 (s), 1032 (s), 862 (m) cm^{-1}; **^{1}H NMR** (400 MHz, $CDCl_3$) 7.26–7.09 (m, 5H), 6.35 (s, 1H, 8-H), 5.18 (d, *J* = 4.8 Hz, 1H, 5-H), 4.16 (d, *J* = 4.8 Hz, 1H, 6-H), 3.82 (dq, *J* = 10.8, 7.2 Hz, 1H, 13-H), 3.76 (dq, *J* = 10.8, 7.2 Hz, 1H, 13-H), 2.35 (s, 3H, 11-H), 1.20 (s, 9H, 10-H), 0.93 (t, *J* = 7.2 Hz, 3H, 14-H); **^{13}C{^{1}H} NMR** (100 MHz, $CDCl_3$) 169.6 (s, C-12), 156.8 (d, C-8), 138.4 (s), 138.0 (s), 132.4 (s), 129.0 (d), 128.3 (d), 126.1 (d), 122.7 (d), 79.5 (d, C-5), 61.1 (t, C-13), 53.2 (d, C-6), 36.7 (s, C-9), 30.0 (q, C-10), 21.6 (q, C-11), 13.8 (q, C-14); **HRMS** (DART$^+$) Calculated ($C_{18}H_{25}O_3Cl_2Ge$): 433.0387 ($[M+H]^+$), Found: 433.0385 ($[M+H]^+$).

^{1}H NMR (400 MHz, $CDCl_3$)

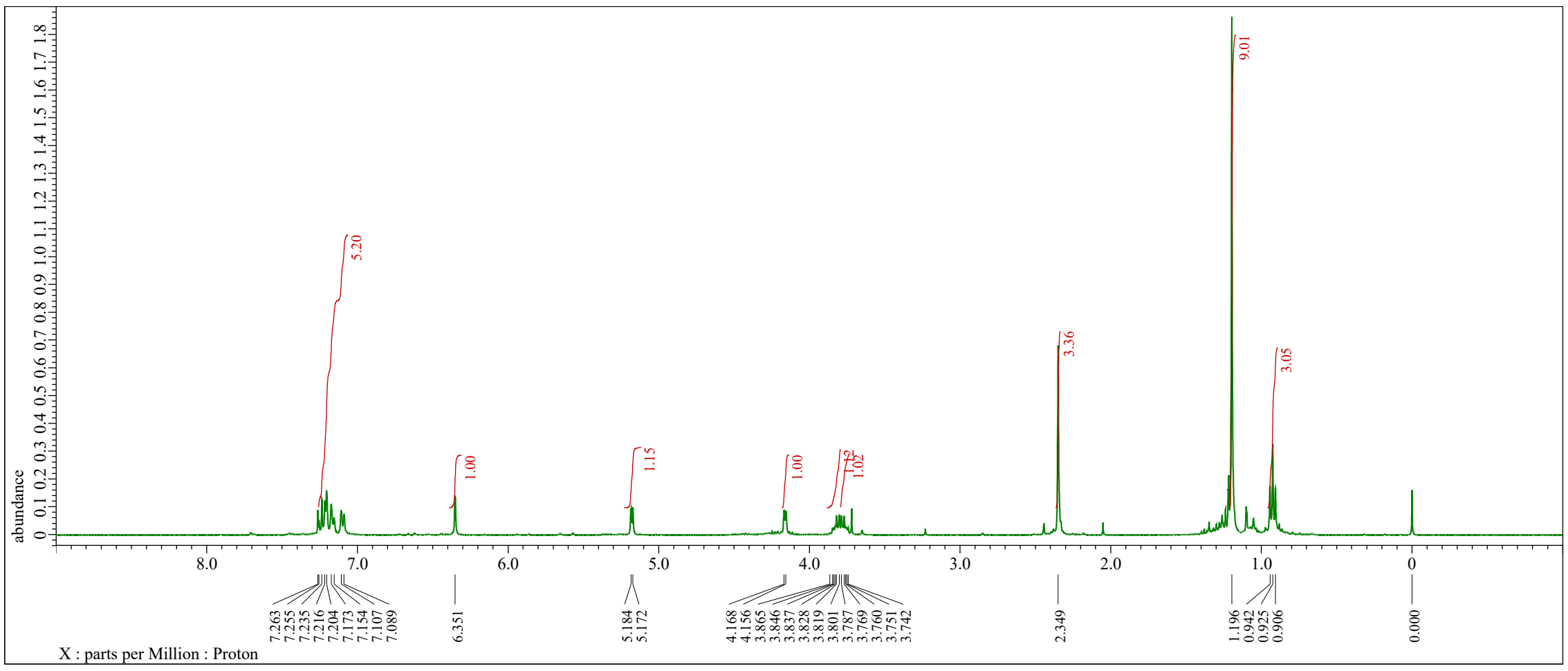

^{13}C{^{1}H} NMR (100 MHz, $CDCl_3$)

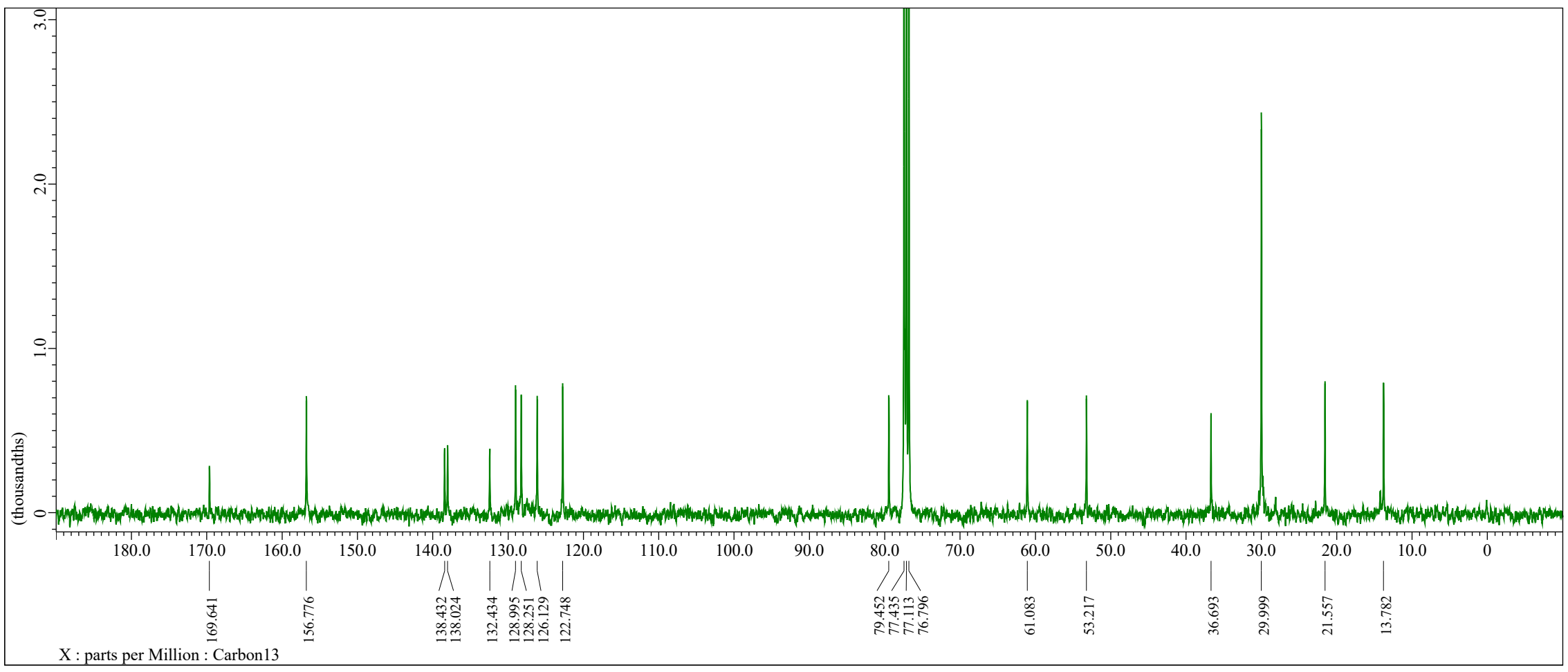

Ethyl (4*R,5*S**,*E*)-2,2-dichloro-3-(2,2-dimethylpropylidene)-5-(*p*-tolyl)-1,2-oxagermolane-4-carboxylate *anti*-*α*-4af**

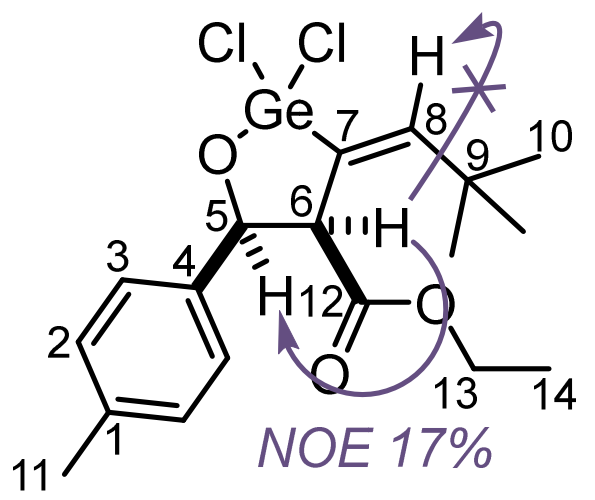

To a mixture of **1a** (0.0251 g, 0.149 mmol) and 4-methylbenzaldehyde **3f** (0.0236 mL, 0.200 mmol) in EtOAc (1.0 mL) was added $GeCl_2$-dioxane (0.0231 g, 0.0997 mmol). After the reaction mixture was stirred at room temperature for 18 h, the solvent was removed under vacuum. The yield was determined by ^{1}H NMR of the crude product using 1,1,2,2-tetrachloroethane as an internal standard (54%, *anti*-*α*-**4af**/*anti*-*γ*-**4af** = 95/5, single diastereomer).

To obtain the spectrodata of the isolated product, some conditions were modified. To a mixture of **1a** (1.0 eq) and 4-methylbenzaldehyde **3f** (1.0 eq) in EtOAc (1.0 mL) was added GeCl2-dioxane (1.0 eq). After the reaction mixture was stirred at room temperature for 18 h, the solvent was removed under vacuum. Careful extraction of the crude mixture with heptane gave *anti*-*α*-**4af** in pure form as colorless neat.

IR (KBr) 3095 (w), 2963 (m), 1718 (s), 1469 (m), 1370 (m), 1325 (m), 1182 (s), 1032 (m), 868 (m) cm^{-1}; **^{1}H NMR** (400 MHz, $CDCl_3$) 7.27 (d, *J* = 8.0 Hz, 2H), 7.15 (d, *J* = 8.0 Hz, 2H), 6.34 (d, *J* = 1.2 Hz, 1H, 8-H), 5.19 (d, *J* = 4.8 Hz, 1H, 5-H), 4.15 (dd, *J* = 5.0, 1.4 Hz, 1H, 6-H), 3.83 (dq, *J* = 10.8, 7.2 Hz, 1H, 13-H), 3.77 (dq, *J* = 11.0, 7.4 Hz, 1H, 13-H), 2.33 (s, 3H, C-11), 1.19 (s, 9H, 10-H), 0.94 (t, *J* = 7.2 Hz, 3H, 14-H); **^{13}C{^{1}H} NMR** (100 MHz, $CDCl_3$) 169.7 (s, C-12), 156.8 (d, C-8), 137.9 (s), 135.6 (s), 132.5 (s), 129.1 (d), 125.5 (d), 79.4 (d, C-5), 61.2 (t, C-13), 53.4 (d, C-6), 36.7 (s, C-9), 30.0 (q, C-10), 21.3 (q, C-11), 13.8 (q, C-14); **HRMS** (DART$^+$) Calculated ($C_{18}H_{25}O_3Cl_2Ge$): 433.0387 ([M+H]$^+$), Found: 433.0380 ([M+H]$^+$).

^{1}H NMR (400 MHz, $CDCl_3$)

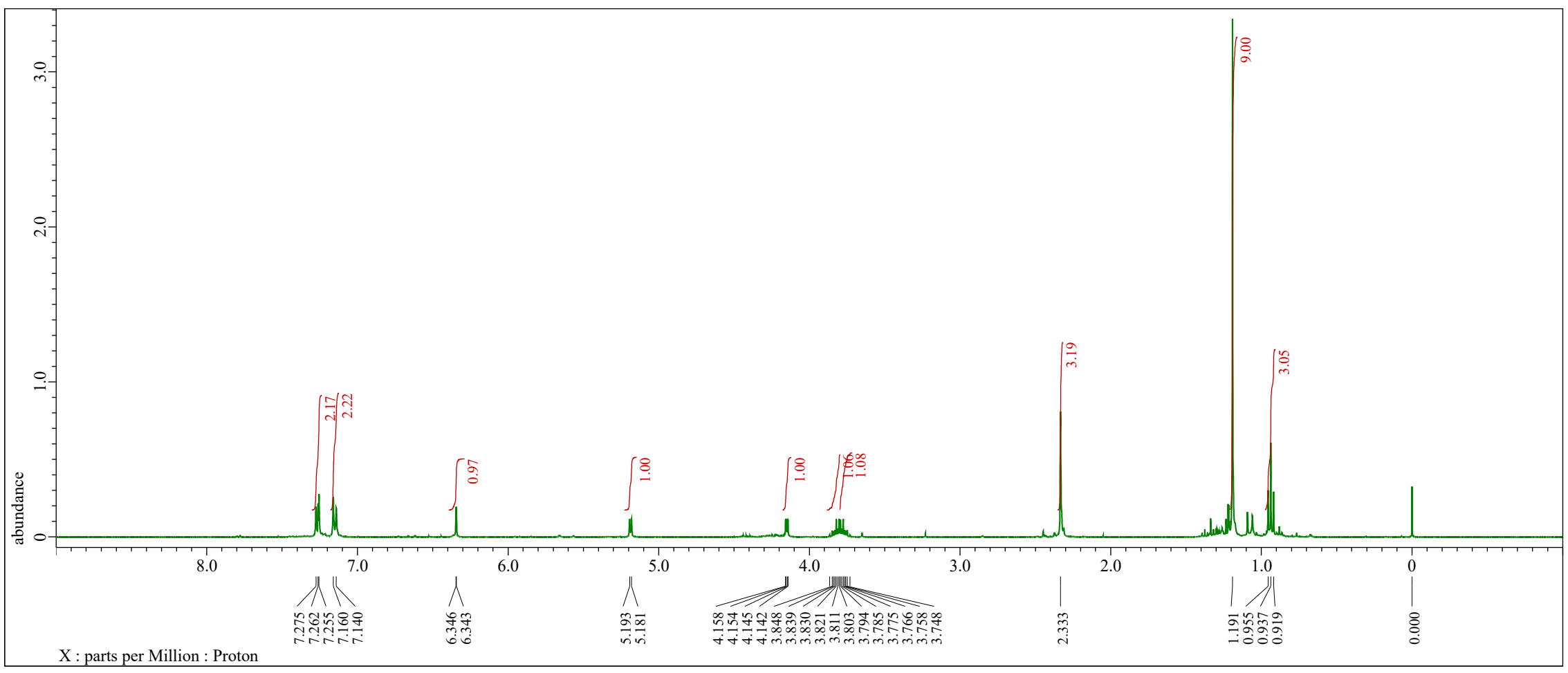

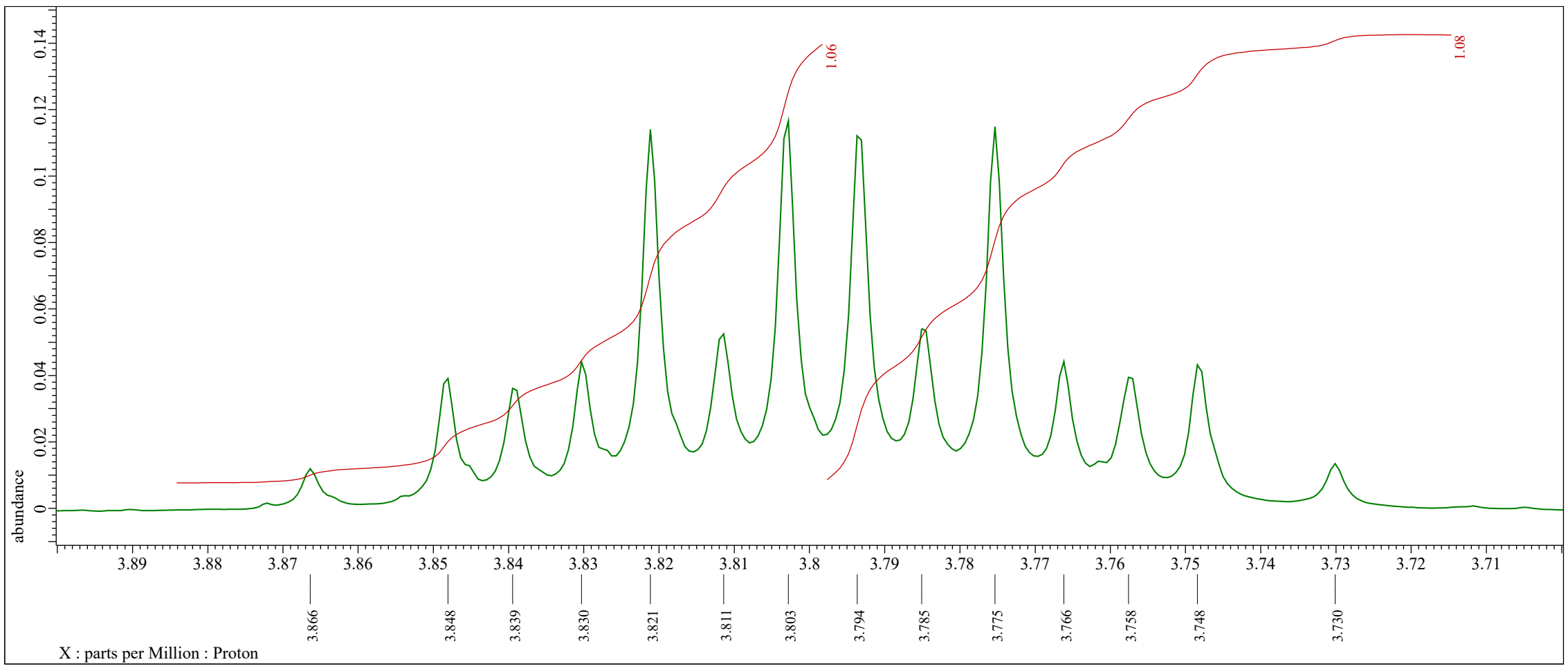

^{13}C{^{1}H} NMR (100 MHz, $CDCl_3$)

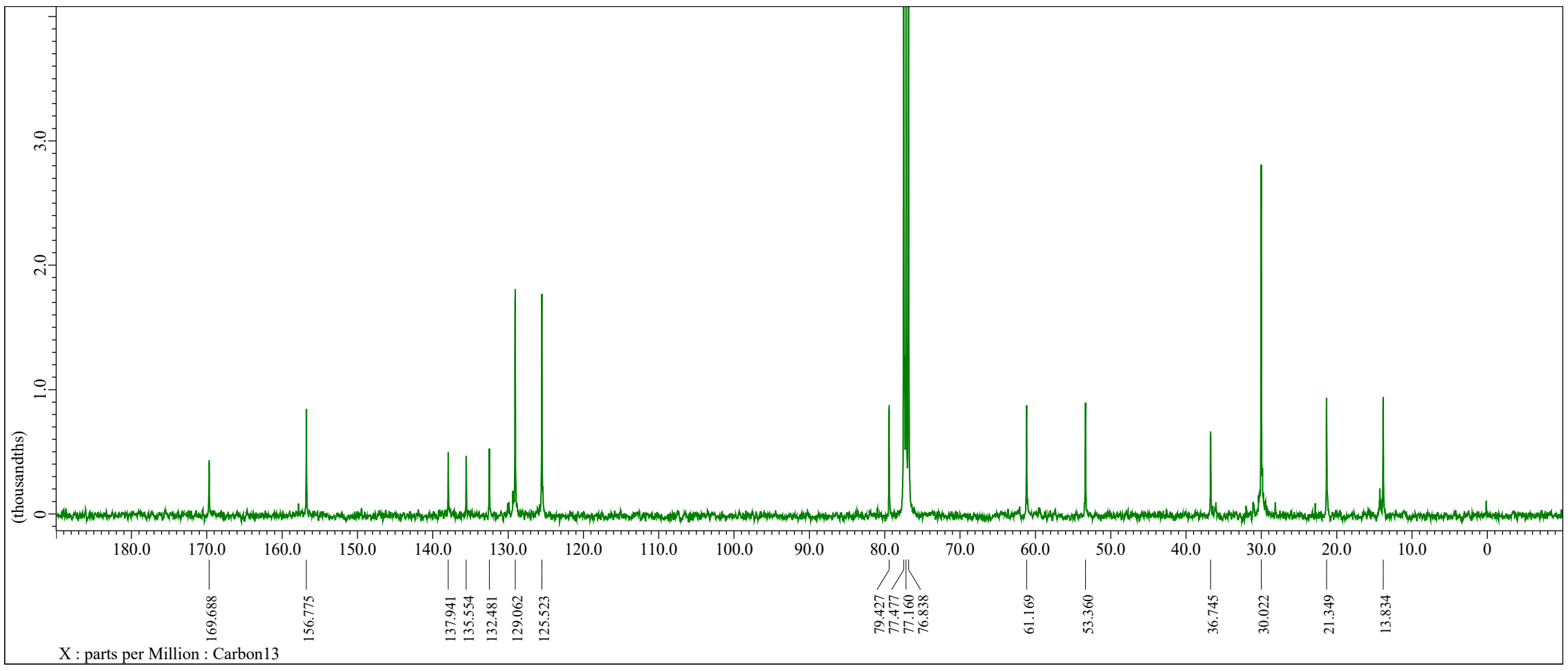

Ethyl (4*R,5*S**,*E*)-2,2-dichloro-3-(2,2-dimethylpropylidene)-5-(naphthalen-2-yl)-1,2-oxagermolane-4-carboxylate** *anti*-*α*-**4ag**

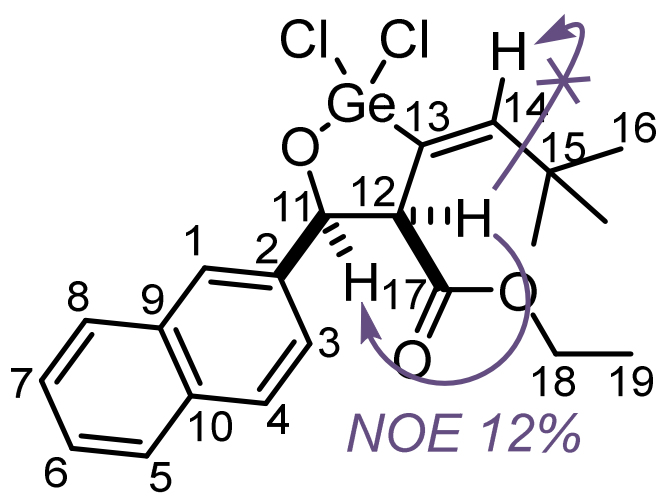

To a mixture of **1a** (0.0253 g, 0.150 mmol) and 2-naphthaldehyde **3g** (0.0310 g, 0.198 mmol) in EtOAc (1.0 mL) was added $GeCl_2$-dioxane (0.0231 g, 0.100 mmol). After the reaction mixture was stirred at room temperature for 18 h, the solvent was removed under vacuum. The yield was determined by ^{1}H NMR of the crude product using 1,1,2,2-tetrachloroethane as an internal standard (77%, *anti*-*α*-**4ag**/*anti*-*γ*-**4ag** = 91/9, single diastereomer).

To obtain the spectrodata of the isolated product, some conditions were modified. To a mixture of **1a** (1.1 eq) and 2-naphthaldehyde **3g** (0.090 eq) in EtOAc (1.0 mL) was added $GeCl_2$-dioxane (1.0 eq). After the reaction mixture

was stirred at room temperature for 18 h, the solvent was removed under vacuum. Careful extraction of the crude mixture with hexane gave *anti*-α-**4ag** in pure form as a colorless solid.

mp 54.0–55.0 °C; **IR** (KBr) 3058 (w), 2962 (m), 1717 (s), 1469 (m), 1368 (m), 1328 (m), 1184 (m), 1031 (m), 859 (m) cm^{-1}; **^{1}H NMR** (400 MHz, $CDCl_3$) 7.92 (s, 1H, 1-H), 7.86–7.81 (m, 3H), 7.51–7.43 (m, 3H), 6.39 (d, J = 1.6 Hz, 1H, 14-H), 5.38 (d, J = 4.8 Hz, 1H, 11-H), 4.29 (dd, J = 5.2, 1.2 Hz, 1H, 12-H), 3.73 (dq, J = 10.8, 7.2 Hz, 1H, 18-H), 3.63 (dq, J = 10.8, 7.2 Hz, 1H, 18-H), 1.22 (s, 9H, 16-H), 0.74 (t, J = 7.2 Hz, 3H, 19-H); **^{13}C{^{1}H} NMR** (100 MHz, $CDCl_3$) 169.5 (s, C-17), 156.9 (d, C-14), 136.0, 133.3, 133.2, 132.4, 128.2, 128.0, 127.8, 126.3, 126.2, 124.7 (d, C-1), 123.5, 79.5 (d, C-11), 61.1 (t, C-18), 53.1 (d, C-12), 36.7 (s, C-15), 30.0 (q, C-16), 13.6 (q, C-19); **HRMS** (DART^{+}) Calculated ($C_{21}H_{25}O_3Cl_2Ge$): 469.0387 ([M+H]$^{+}$), Found: 469.0384 ([M+H]$^{+}$).

^{1}H NMR (400 MHz, $CDCl_3$)

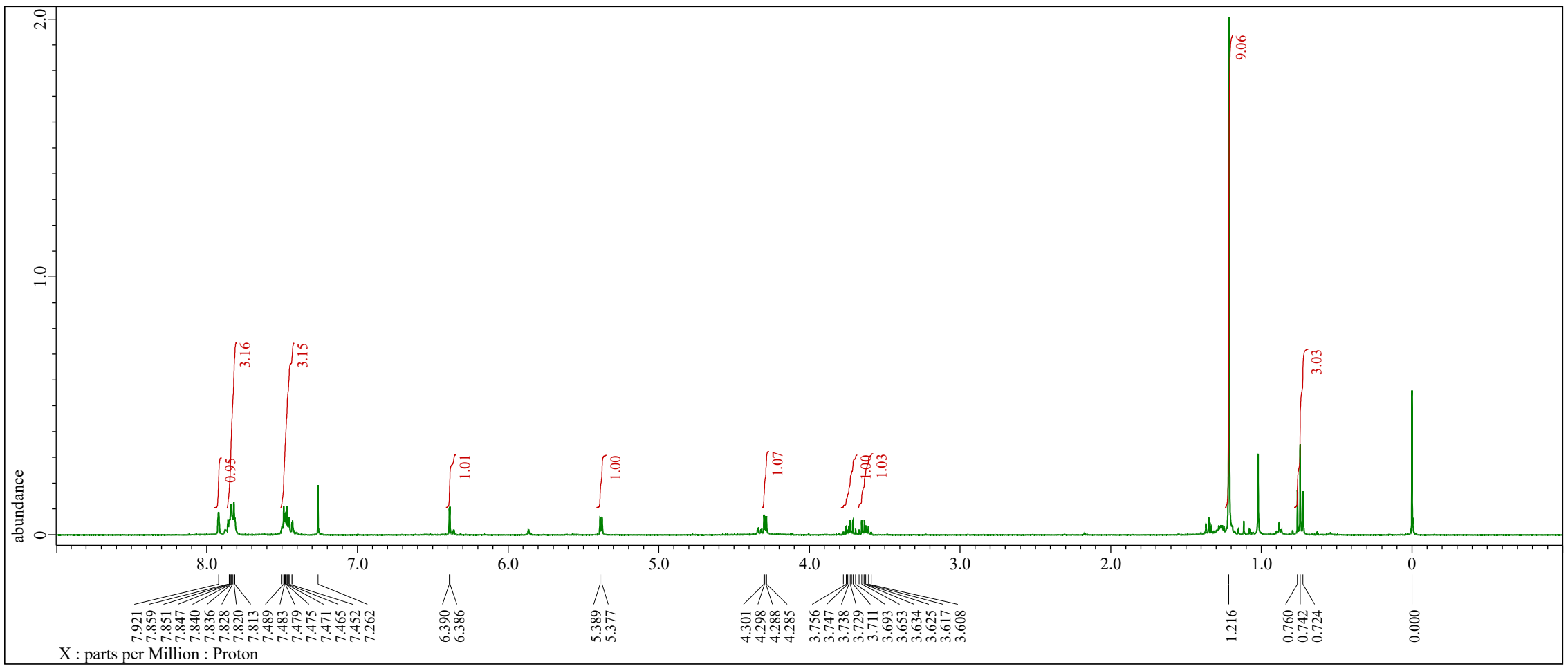

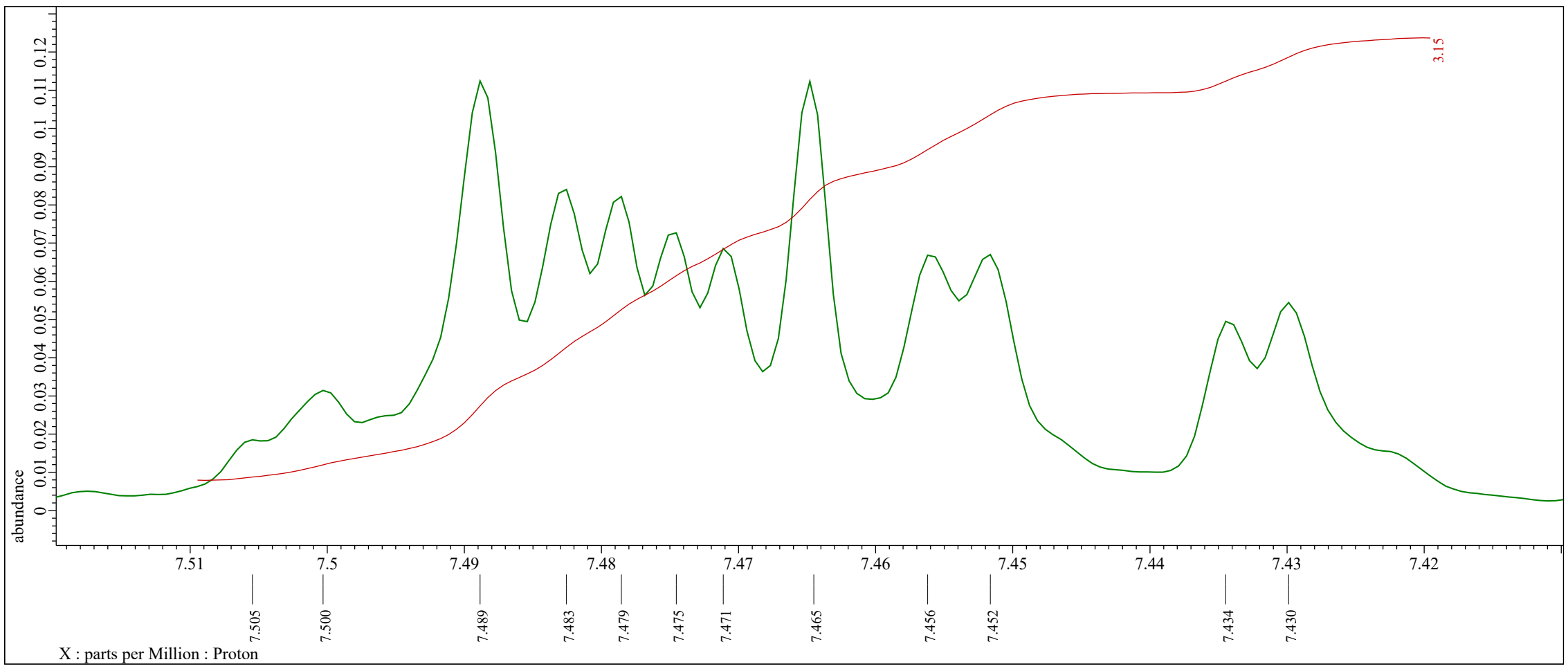

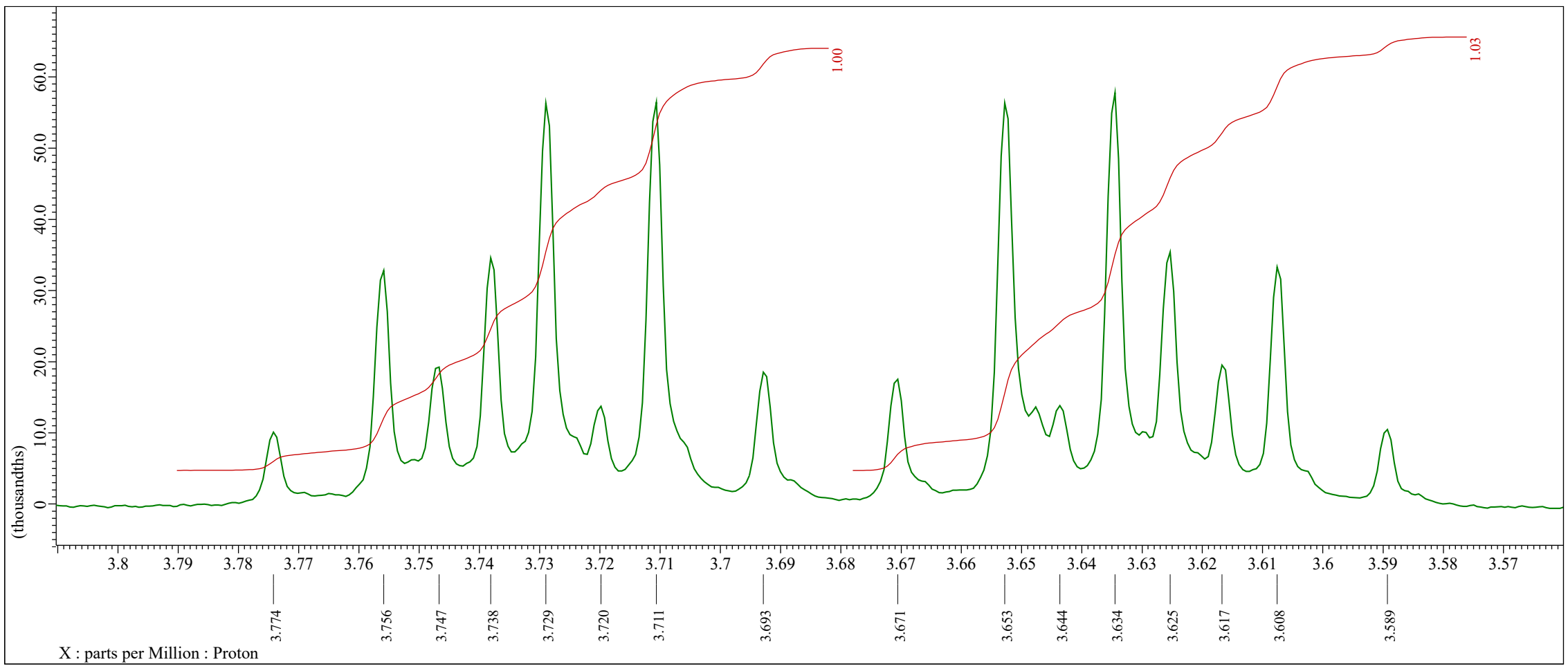

^{13}C{^{1}H} NMR (100 MHz, $CDCl_3$)

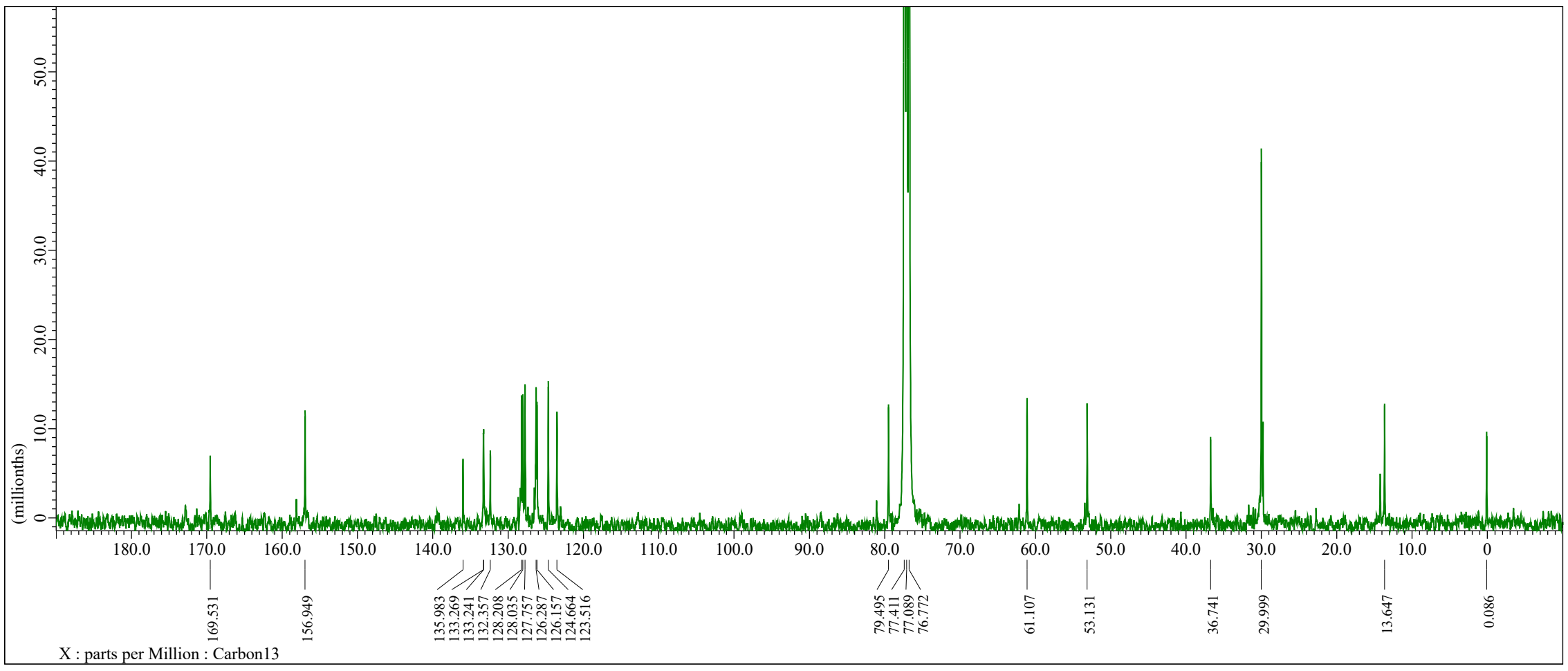

Ethyl (4*R,5*S**,*E*)-2,2-dichloro-3-(2,2-dimethylpropylidene)-5-(naphthalen-1-yl)-1,2-oxagermolane-4-carboxylate** *anti*-*α*-**4ah**

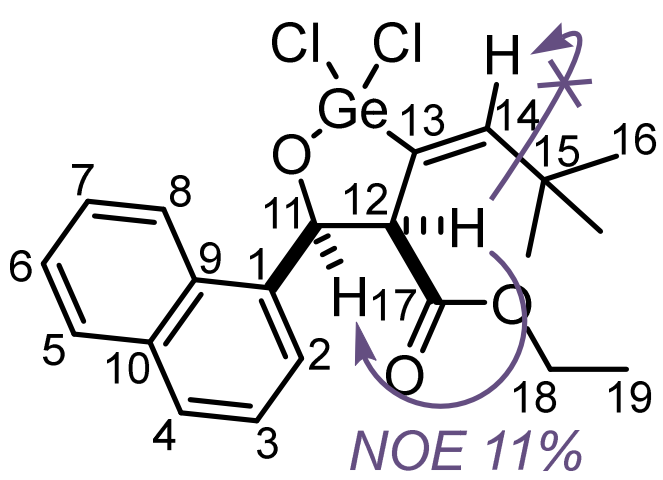

To a mixture of **1a** (0.0256 g, 0.152 mmol) and 1-naphthaldehyde **3h** (0.0272 mL, 0.200 mmol) in EtOAc (1.0 mL) was added $GeCl_2$-dioxane (0.0233 g, 0.101 mmol). After the reaction mixture was stirred at room temperature for 18 h, the solvent was removed under vacuum. The yield was determined by ^{1}H NMR of the crude product using 1,1,2,2-tetrachloroethane as an internal standard (82%, *anti*-*α*-**4ah**/*anti*-*γ*-**4ah** = 92/8, single diastereomer).

To obtain the spectrodata of the isolated product, some conditions were modified. To a mixture of **1a** (1.1 eq) and 1-naphthaldehyde **3h** (0.095 eq) in EtOAc (1.0 mL) was added $GeCl_2$-dioxane (1.0 eq). After the reaction mixture

was stirred at room temperature for 18 h, the solvent was removed under vacuum. Careful extraction of the crude mixture with hexane gave *anti*-*α*-**4ah** in pure form as colorless neat.

IR (KBr) 3060 (w), 2961 (m), 1718 (s), 1469 (w), 1369 (w), 1331 (w), 1183 (m), 1031 (m), 860 (w) cm^{-1}; ^{1}**H NMR** (400 MHz, $CDCl_3$) 7.90 (dd, J = 8.0, 1.2 Hz, 1H), 7.81 (d, J = 7.6 Hz, 3H), 7.59–7.46 (m, 3H), 6.43 (d, J = 1.2 Hz, 1H, 14-H), 5.98 (d, J = 4.8 Hz, 1H, 11-H), 4.50 (dd, J = 4.6, 1.4 Hz, 1H, 12-H), 3.69–3.50 (m, 2H, 18-H), 1.21 (s, 9H, 16-H), 0.67 (t, J = 7.2 Hz, 3H, 19-H); ^{13}**C{^{1}H} NMR** (100 MHz, $CDCl_3$) 169.6 (s, C-17), 157.0 (d, C-14), 134.1, 133.6, 132.9, 130.1, 129.4, 128.8, 126.7, 125.7, 125.6, 123.5, 121.6, 76.5 (d, C-11), 61.0 (t, C-18), 52.0 (d, C-12), 36.6 (s, C-15), 30.1 (q, C-16), 13.6 (q, C-19); **HRMS** (DART^{+}) Calculated ($C_{21}H_{25}O_3Cl_2Ge$): 469.0387 ($[M+H]^{+}$), Found: 469.0406 ($[M+H]^{+}$).

^{1}**H NMR** (400 MHz, $CDCl_3$)

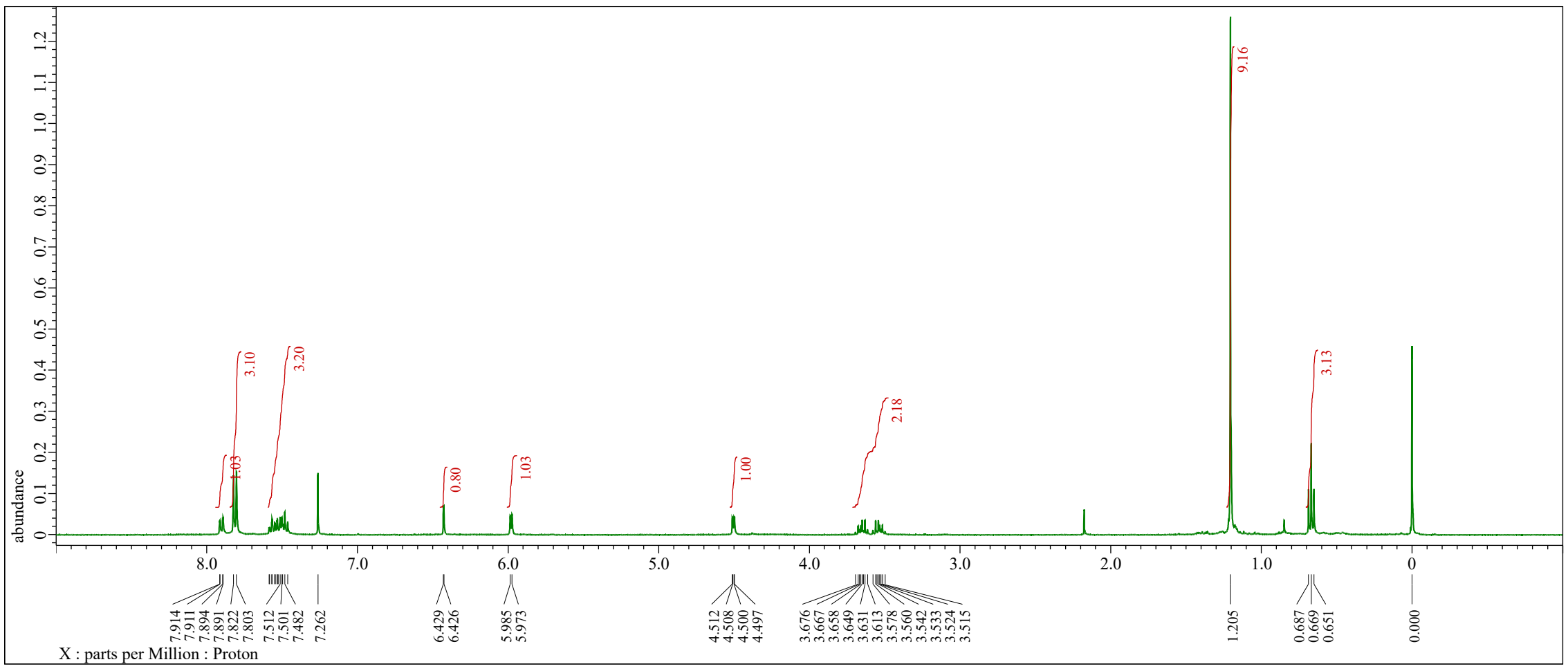

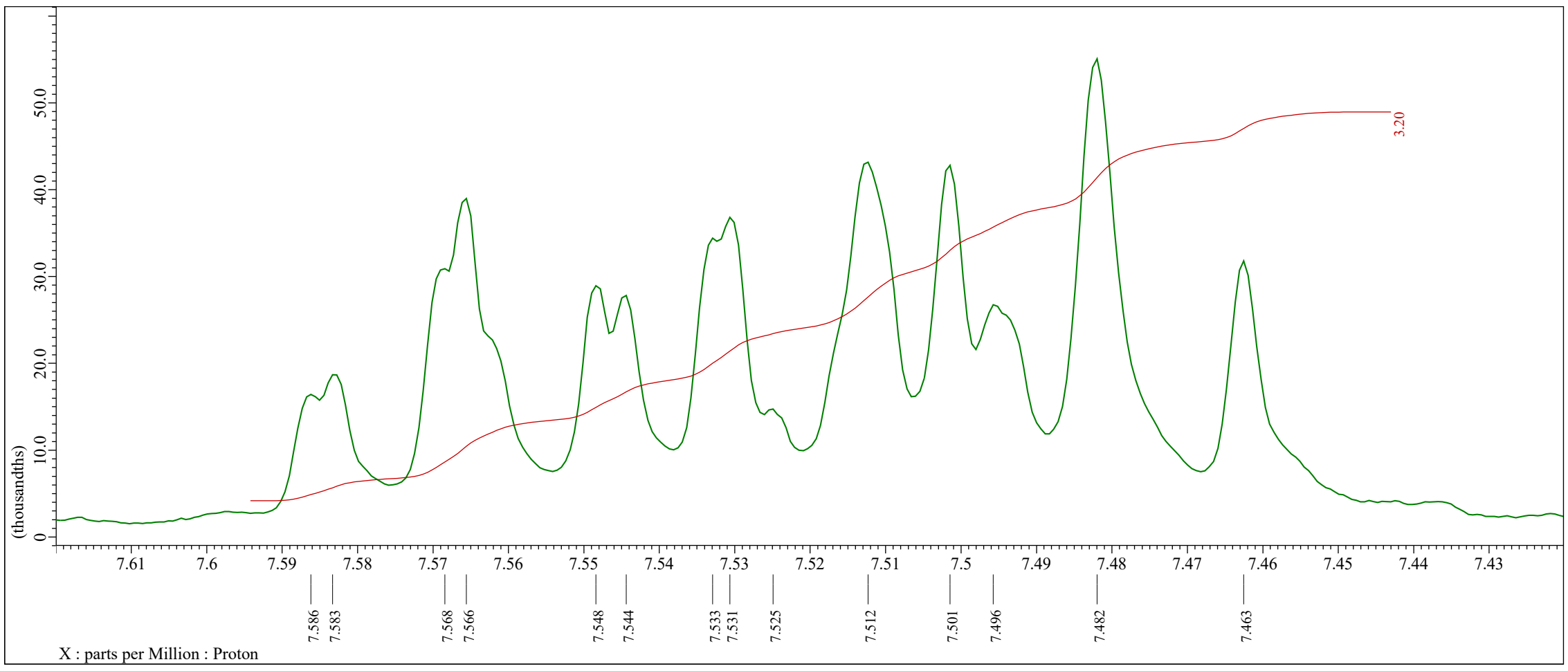

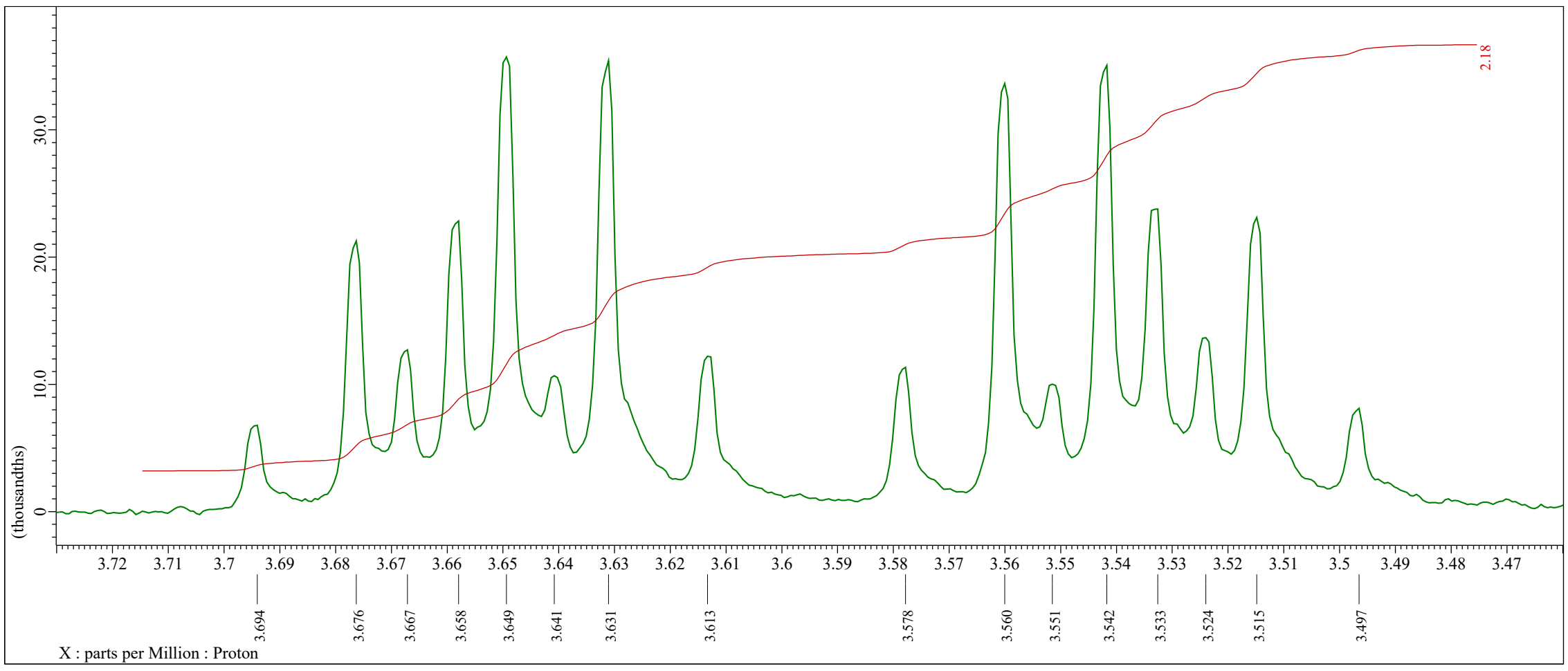

^{13}C{^{1}H} NMR (100 MHz, $CDCl_3$)

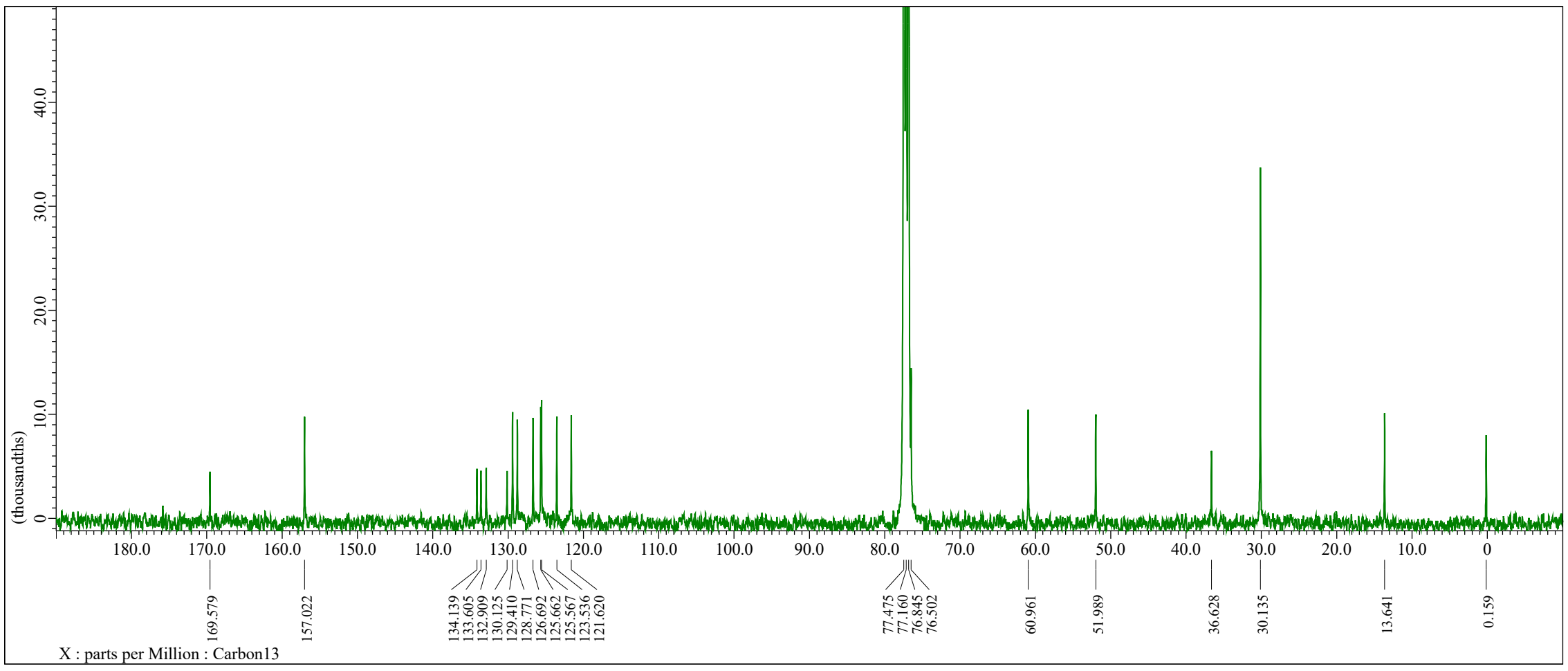

Ethyl (4*R,5*S**,*E*)-2,2-dichloro-3-(2,2-dimethylpropylidene)-5-(thiophen-2-yl)-1,2-oxagermolane-4-carboxylate** *anti*-*α*-**4ai**

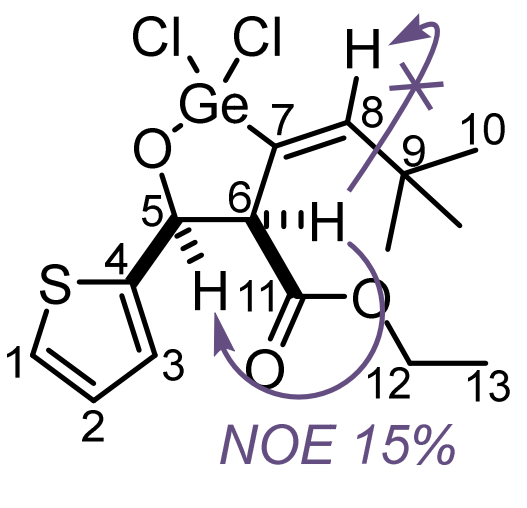

To a mixture of **1a** (0.0251 g, 0.149 mmol) and thiophene-2-carbaldehyde **3i** (0.0182 mL, 0.200 mmol) in EtOAc (1.0 mL) was added $GeCl_2$-dioxane (0.0232 g, 0.100 mmol). After the reaction mixture was stirred at room temperature for 18 h, the solvent was removed under vacuum. The yield was determined by ^{1}H NMR of the crude product using 1,1,2,2-tetrachloroethane as an internal standard (70%, *anti*-*α*-**4ai**/*anti*-*γ*-**4ai** = 96/4, single diastereomer).

To obtain the spectrodata of the isolated product, some conditions were modified. To a mixture of **1a** (1.1 eq) and

thiophene-2-carbaldehyde **3i** (2.0 eq) in EtOAc (1.0 mL) was added $GeCl_2$-dioxane (1.0 eq). After the reaction mixture was stirred at room temperature for 16 h, the solvent was removed under vacuum. Careful extraction of the crude mixture with hexane gave *anti*-*α*-**4ai** in pure form as colorless neat.

IR (KBr) 3110 (w), 2965 (s), 1718 (s), 1470 (w), 1370 (m), 1329 (m), 1214 (m), 1031 (m), 857 (m) cm^{-1}; **^{1}H NMR** (400 MHz, $CDCl_3$) 7.27 (dd, J = 5.0, 1.4 Hz, 1H, 1-H), 7.06 (dt, J = 3.6, 1.1 Hz, 1H, 3-H), 6.99 (dd, J = 5.2, 3.6 Hz, 1H, 2-H), 6.34 (d, J = 1.2 Hz, 1H, 8-H), 5.44 (d, J = 5.0, 1.0 Hz, 1H, 5-H), 4.21 (dd, J = 5.0, 1.4 Hz, 1H, 6-H), 3.96 (dq, J = 11.2, 6.8 Hz, 1H, 12-H), 3.92 (dq, J = 11.2, 6.8 Hz, 1H, 12-H), 1.21 (s, 9H, 10-H), 1.06 (t, J = 7.6 Hz, 3H, 13-H); **^{13}C{^{1}H} NMR** (100 MHz, $CDCl_3$) 169.4 (s, C-11), 157.2 (d, C-8), 141.9 (s, C-4), 131.9 (s, C-7), 126.8 (d, C-2), 125.1 (d, C-1), 124.4 (d, C-3), 76.6 (d, C-5), 61.6 (t, C-12), 53.6 (d, C-6), 36.9 (s, C-9), 30.0 (q, C-10), 13.9 (q, C-13); **HRMS** ($DART^+$) Calculated ($C_{15}H_{21}O_3SCl_2Ge$): 424.9795 ($[M+H]^+$), Found: 424.9809 ($[M+H]^+$).

^{1}H NMR (400 MHz, $CDCl_3$)

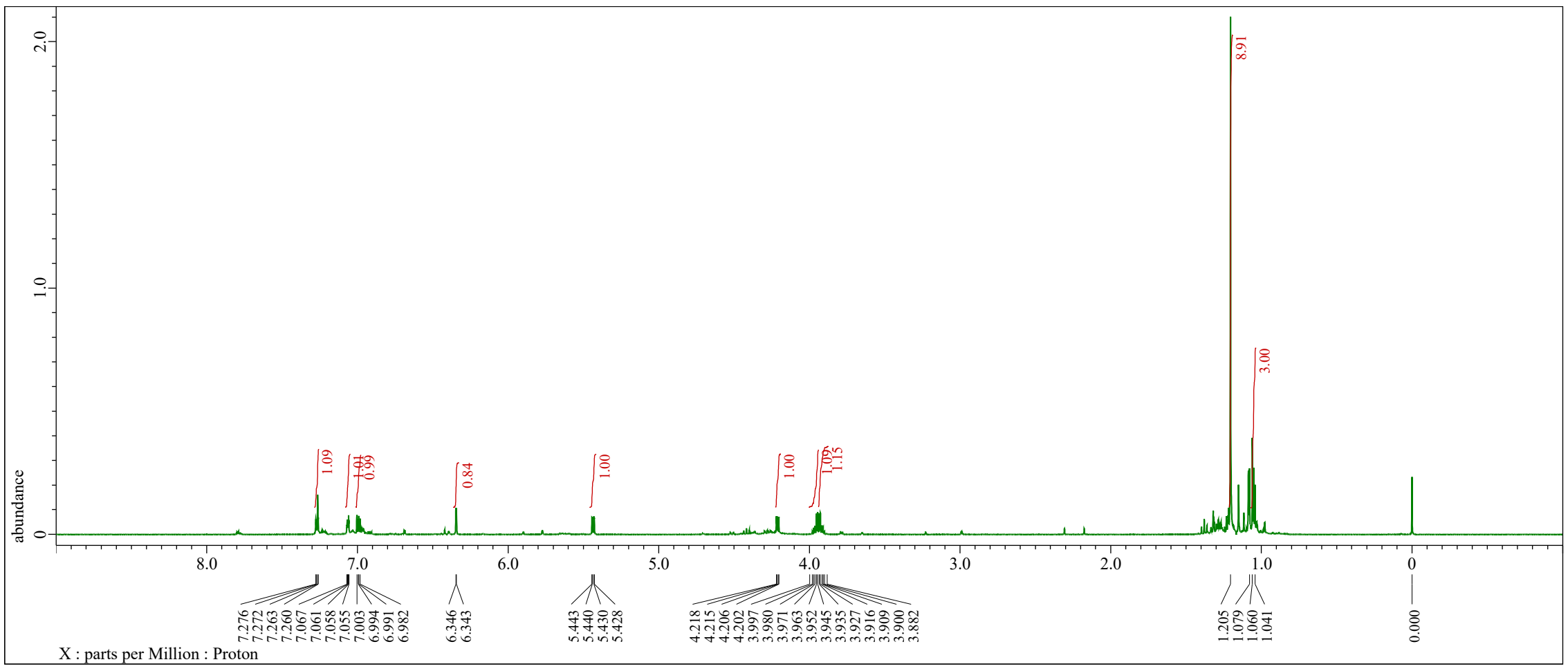

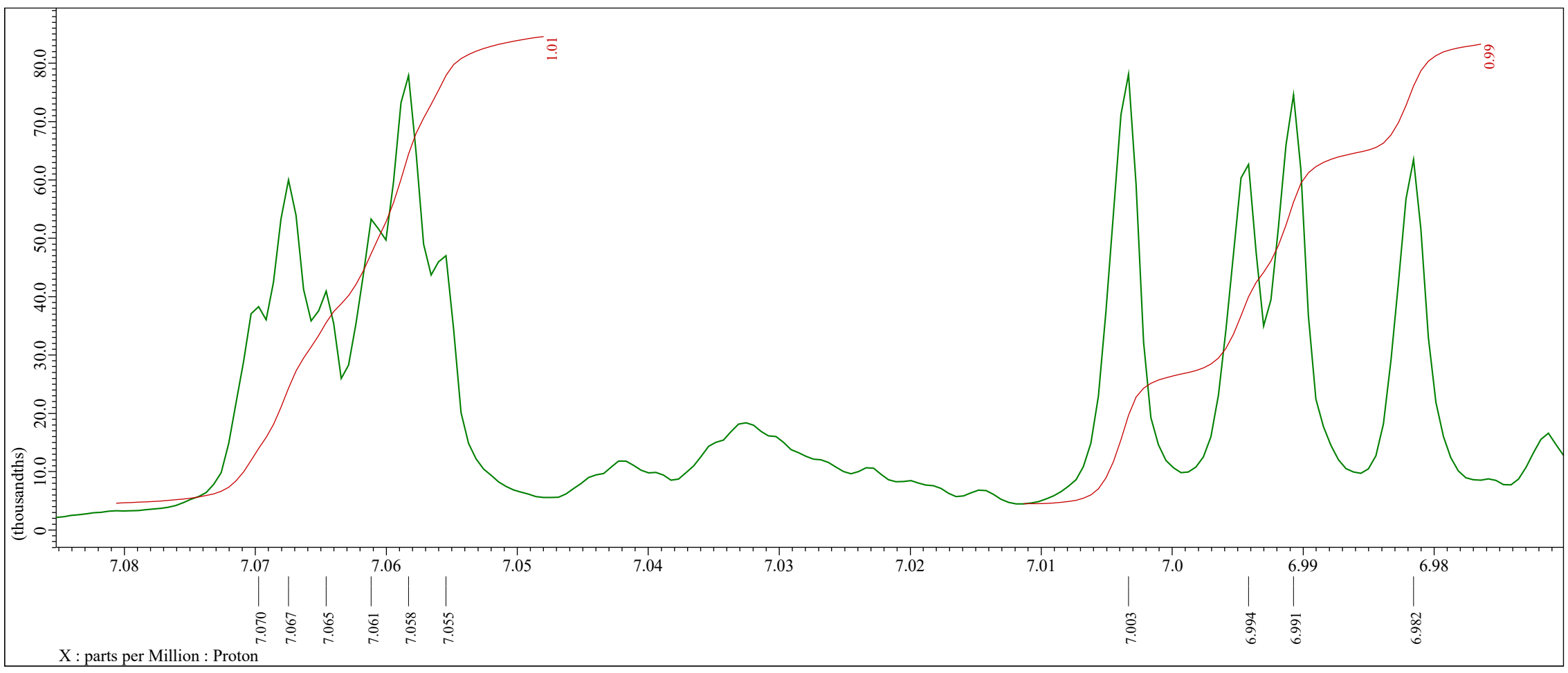

$^{13}C\{^{1}H\}$ **NMR** (100 MHz, $CDCl_3$)

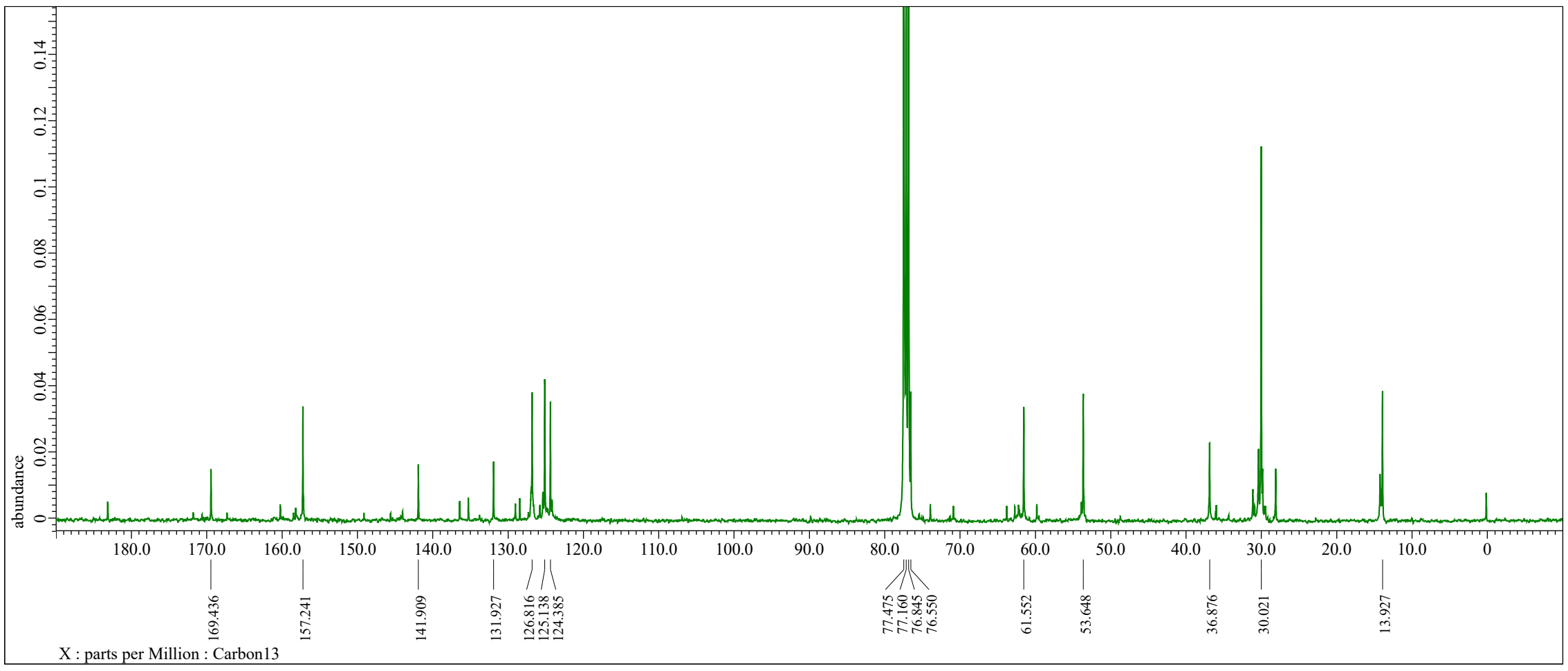

Ethyl (4*R,5*S**,*E*)-5-(*tert*-butyl)-2,2-dichloro-3-(2,2-dimethylpropylidene)-1,2-oxagermolane-4-carboxylate** *anti*-*α*-**4ak**

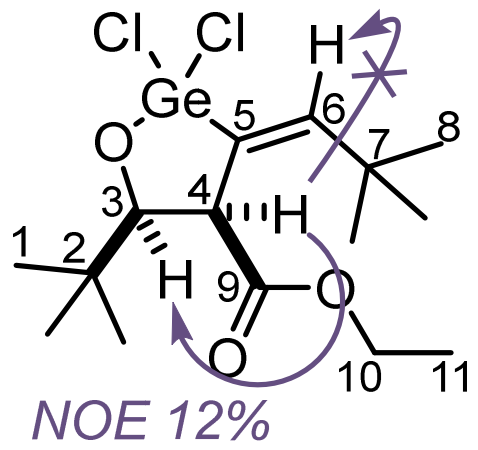

Conditions A

To a mixture of **1a** (0.0251 g, 0.149 mmol), pivalaldehyde **3k** (0.0163 mL, 0.200 mmol), and $BF_3 \cdot OEt_2$ (0.0252 mL, 0.100 mmol) in CH_3CN (1.0 mL) was added $GeCl_2$-dioxane (0.0230 g, 0.0993 mmol). After the reaction mixture was left to stand at room temperature for 18 h, the solvent was removed under vacuum. The yield was determined by ^{1}H NMR of the crude product using 1,1,2,2-tetrachloroethane as an internal standard (5%, single isomer).

Conditions B

To a mixture of **1a** (0.0253 g, 0.150 mmol) and pivalaldehyde **3k** (0.0217 mL, 0.200 mmol) in EtOAc (1.0 mL) was added $GeCl_2$-dioxane (0.0232 g, 0.100 mmol). After the reaction mixture was stirred at room temperature for 18 h, the solvent was removed under vacuum. The yield was determined by ^{1}H NMR of the crude product using 1,1,2,2-tetrachloroethane as an internal standard (57%, single isomer).

Conditions C

To a mixture of **1a** (0.0336 g, 0.200 mmol) and pivalaldehyde **3k** (0.0217 mL, 0.200 mmol) in hexane (1.0 mL) was added $GeCl_2$-dioxane (0.0233 g, 0.101 mmol). After the reaction mixture was stirred at room temperature for 18 h, the solvent was removed under vacuum. The yield was determined by ^{1}H NMR of the crude product using 1,1,2,2-tetrachloroethane as an internal standard (71%, single isomer).

To obtain the spectrodata of the isolated product, some conditions were modified. To a mixture of **1a** (1.1 eq) and pivalaldehyde **3k** (2.0 eq) in hexane (1.0 mL) was added $GeCl_2$-dioxane (1.0 eq). After the reaction mixture was stirred at room temperature for 20 h, the solvent was removed under vacuum. Careful extraction of the crude mixture

with heptane gave *anti*-α-**4ak** in pure form as a colorless solid.

mp 50.0–50.8 °C; **IR** (KBr); 2962 (s), 1721 (s), 1475 (m), 1369 (m), 1283 (m), 1176 (s), 1020 (m), 976 (w), 883 (m) cm^{-1}; **^{1}H NMR** (400 MHz, $CDCl_3$) 6.22 (d, J = 1.2 Hz, 1H, 6-H), 4.15 (q, J = 7.3 Hz, 2H, 10-H), 3.97 (dd, J = 4.0, 1.2 Hz, 1H, 4-H), 3.57 (d, J = 4.4 Hz, 1H, 3-H), 1.27 (t, J = 7.2 Hz, 3H, 11-H), 1.17 (s, 9H, 8-H), 1.02 (s, 9H, 1-H); **^{13}C{^{1}H} NMR** (100 MHz, $CDCl_3$) 171.3 (s, C-9), 155.3 (d, C-6), 134.6 (s, C-5), 86.6 (d, C-3), 61.4 (t, C-10), 48.2 (d, C-4), 36.6 (s, C-7), 35.7 (s, C-2), 30.1 (q, C-8), 26.0 (q, C-1), 14.0 (q, C-11); **HRMS** (DART^{+}) Calculated ($C_{15}H_{27}O_3Cl_2Ge$): 399.0544 ($[M+H]^+$), Found: 399.0559 ($[M+H]^+$).

^{1}H NMR (400 MHz, $CDCl_3$)

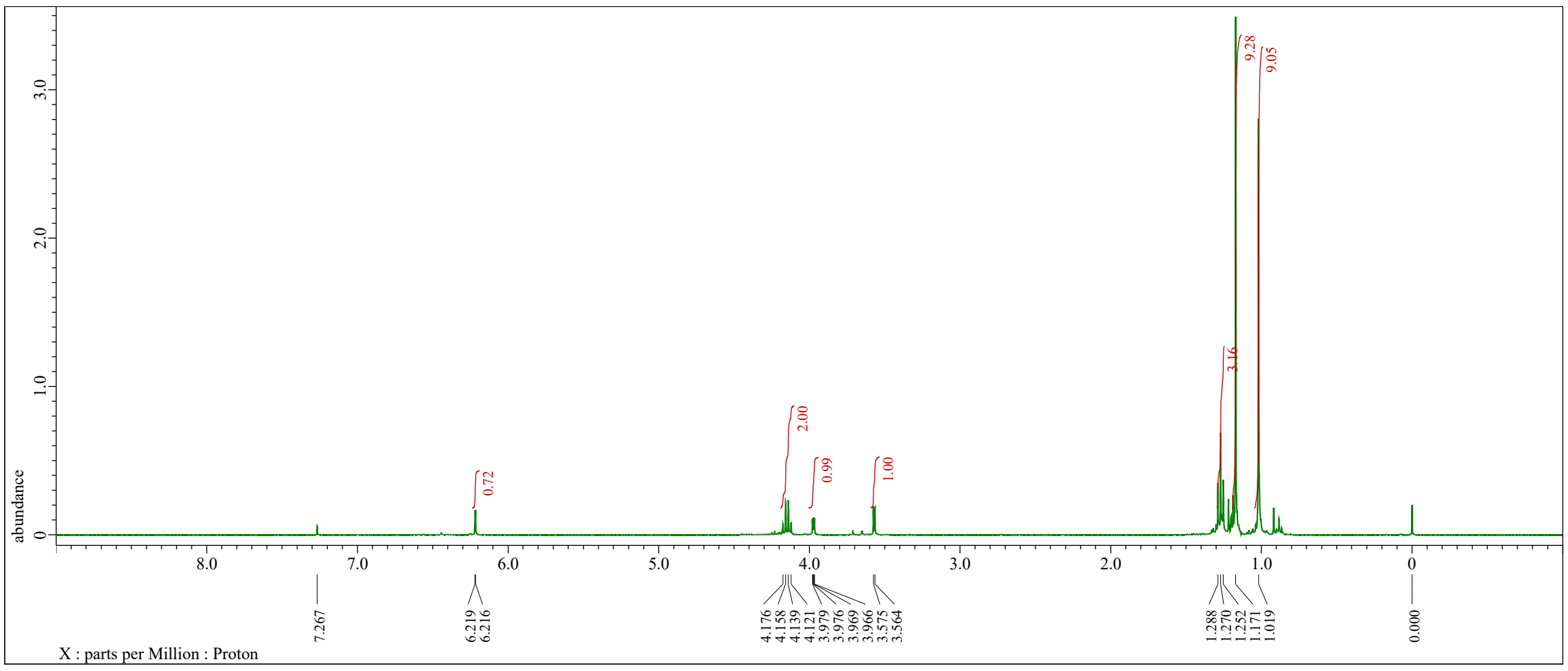

^{13}C{^{1}H} NMR (100 MHz, $CDCl_3$)

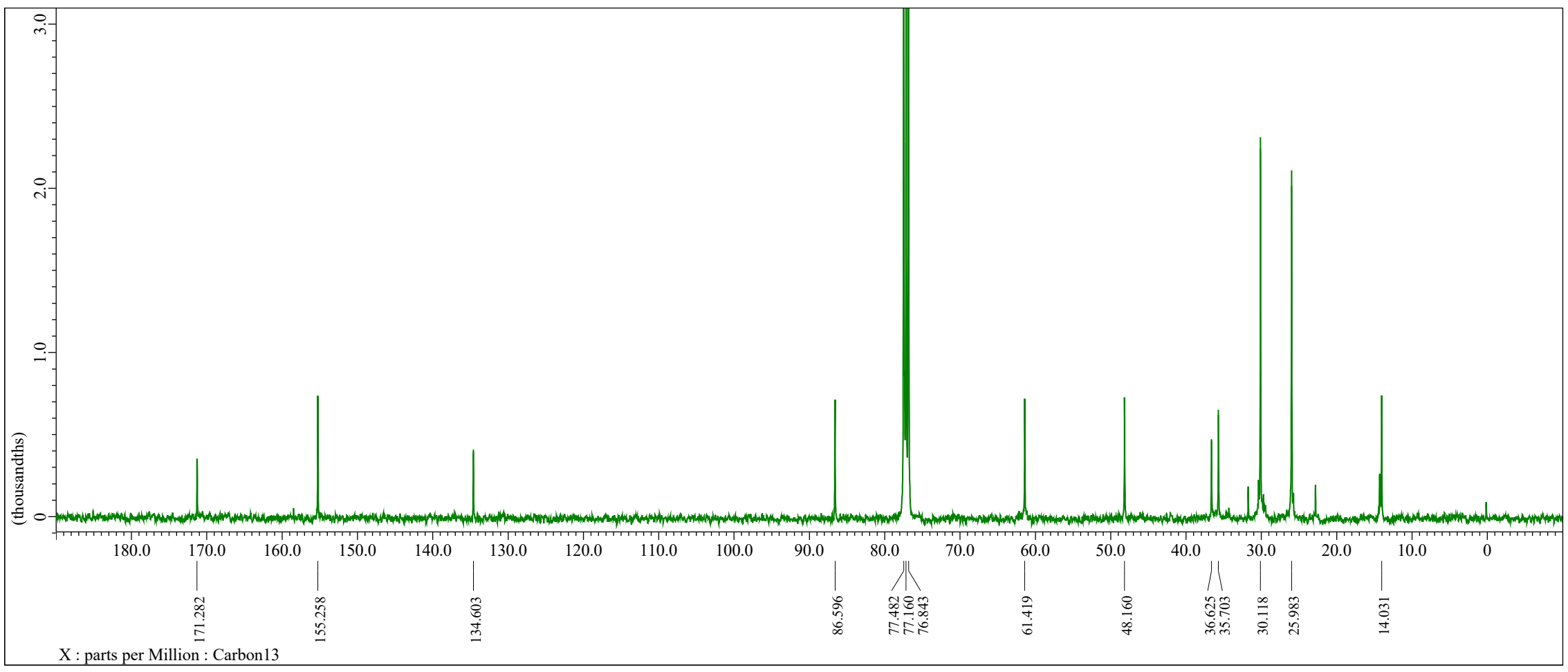

Ethyl (4*R,5*S**,*E*)-2,2-dichloro-3-(2,2-dimethylpropylidene)-5-methyl-5-phenyl-1,2-oxagermolane-4-carboxylate *anti*-*α*-4al**

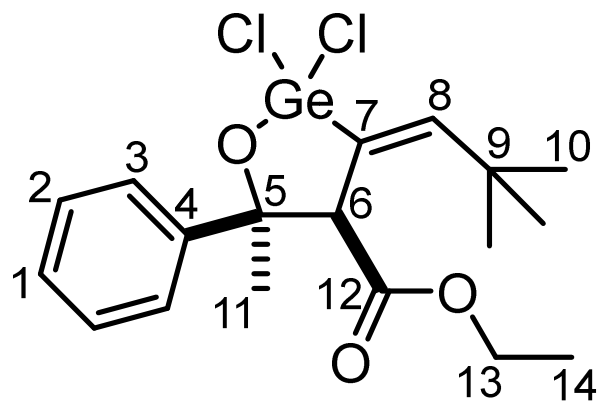

Conditions A

To a mixture of **1a** (0.0252 g, 0.150 mmol), acetophenone **3l** (0.0175 mL, 0.200 mmol), and $BF_3{\cdot}OEt_2$ (0.0252 mL, 0.100 mmol) in CH_3CN (1.0 mL) was added $GeCl_2$-dioxane (0.0230 g, 0.0993 mmol). After the reaction mixture was left to stand at room temperature for 18 h, the solvent was removed under vacuum. The yield was determined by ^{1}H NMR of the crude product using 1,1,2,2-tetrachloroethane as an internal standard (53%, single isomer).

Conditions B

To a mixture of **1a** (0.0251 g, 0.149 mmol) and acetophenone **3l** (0.0233 mL, 0.200 mmol) in EtOAc (1.0 mL) was added $GeCl_2$-dioxane (0.0231 g, 0.0997 mmol). After the reaction mixture was stirred at room temperature for 18 h, the solvent was removed under vacuum. The yield was determined by ^{1}H NMR of the crude product using 1,1,2,2-tetrachloroethane as an internal standard (57%, single isomer).

Conditions C

To a mixture of **1a** (0.0335 g, 0.199 mmol) and acetophenone **3l** (0.0233 mL, 0.200 mmol) in hexane (1.0 mL) was added $GeCl_2$-dioxane (0.0231 g, 0.0997 mmol). After the reaction mixture was stirred at room temperature for 18 h, the solvent was removed under vacuum. The yield was determined by ^{1}H NMR of the crude product using 1,1,2,2-tetrachloroethane as an internal standard (56%, single isomer).

To obtain the spectrodata of the isolated product, recrystallization from hexane was performed to give *anti*-*α*-**4al** in pure form as a colorless solid. The relative configuration was determined by X-ray crystallographic analysis.

mp 134.0–135.0 °C; **IR** (KBr); 3059 (w), 2968 (s), 1715 (s), 1445 (m), 1368 (s), 1317 (s), 1183 (s), 909 (s), 861 (m) cm^{-1}; **^{1}H NMR** (400 MHz, $CDCl_3$); 7.51 (d, *J* = 7.6 Hz, 2H, 3-H), 7.34 (t, *J* = 7.8 Hz, 2H, 2-H), 7.26 (d, *J* = 7.2 Hz, 1H, 1-H), 6.43 (d, *J* = 0.8 Hz, 1H, 8-H), 4.16 (d, *J* = 1.2 Hz, 1H, 6-H), 3.69 (dq, *J* = 10.8, 7.6 Hz, 1H, 13-H), 3.62 (dq, *J* = 10.8, 7.2 Hz, 1H, 13-H), 1.61 (s, 3H, 11-H), 1.22 (s, 9H, 10-H), 0.82 (t, *J* = 7.2 Hz, 3H, 14-H); **^{13}C{^{1}H} NMR** (100 MHz, $CDCl_3$) 170.0 (s, C-12), 157.4 (d, C-8), 143.0 (s, C-4), 132.7 (s, C-7), 128.1 (d, C-2), 127.6 (d, C-1), 125.5 (d, C-3), 83.5 (s, C-5), 61.1 (t, C-13), 57.5 (d, C-6), 36.5 (s, C-9), 30.9 (q, C-11), 30.2 (q, C-10), 13.7 (q, C-14); **HRMS** (DART$^+$) Calculated ($C_{18}H_{28}NO_3Cl_2Ge$): 450.0653 ([M+NH_4]$^+$), Found: 450.0674([M+NH_4]$^+$).

^{1}H NMR (400 MHz, $CDCl_3$)

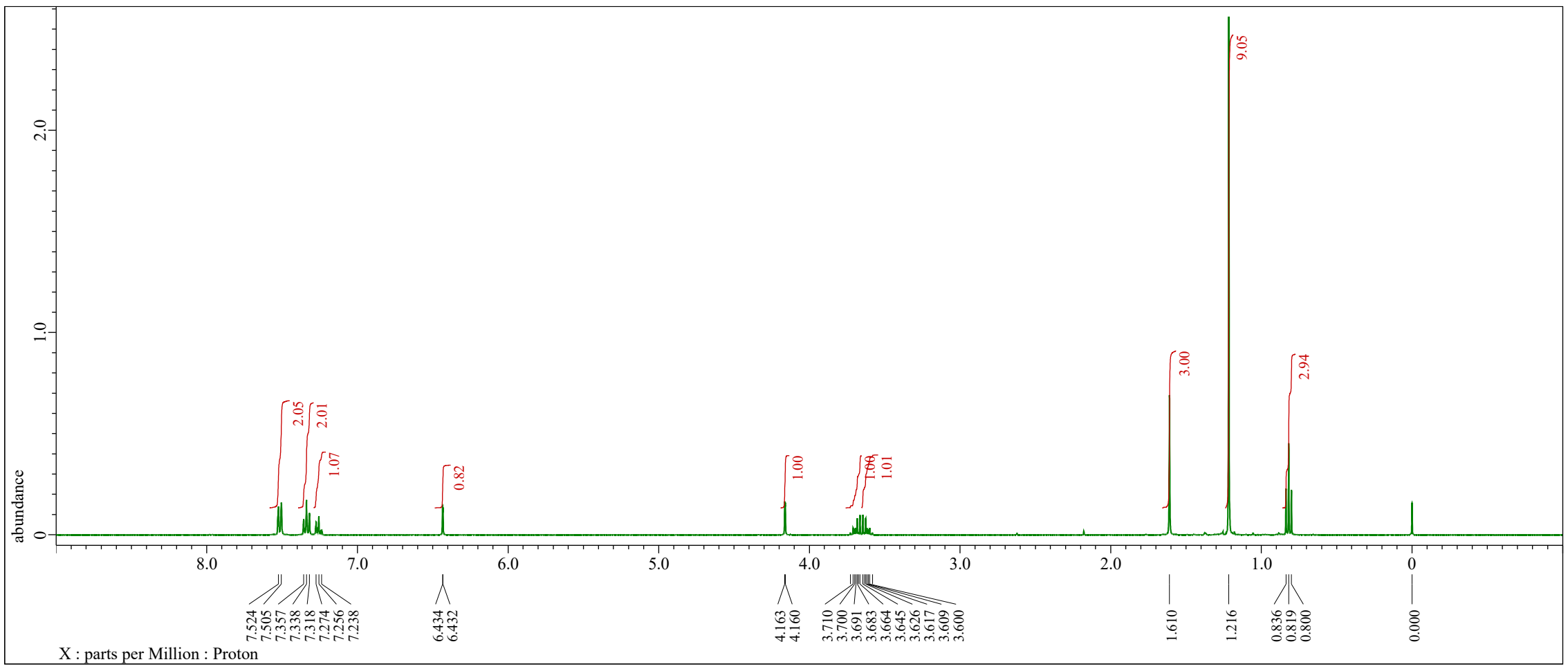

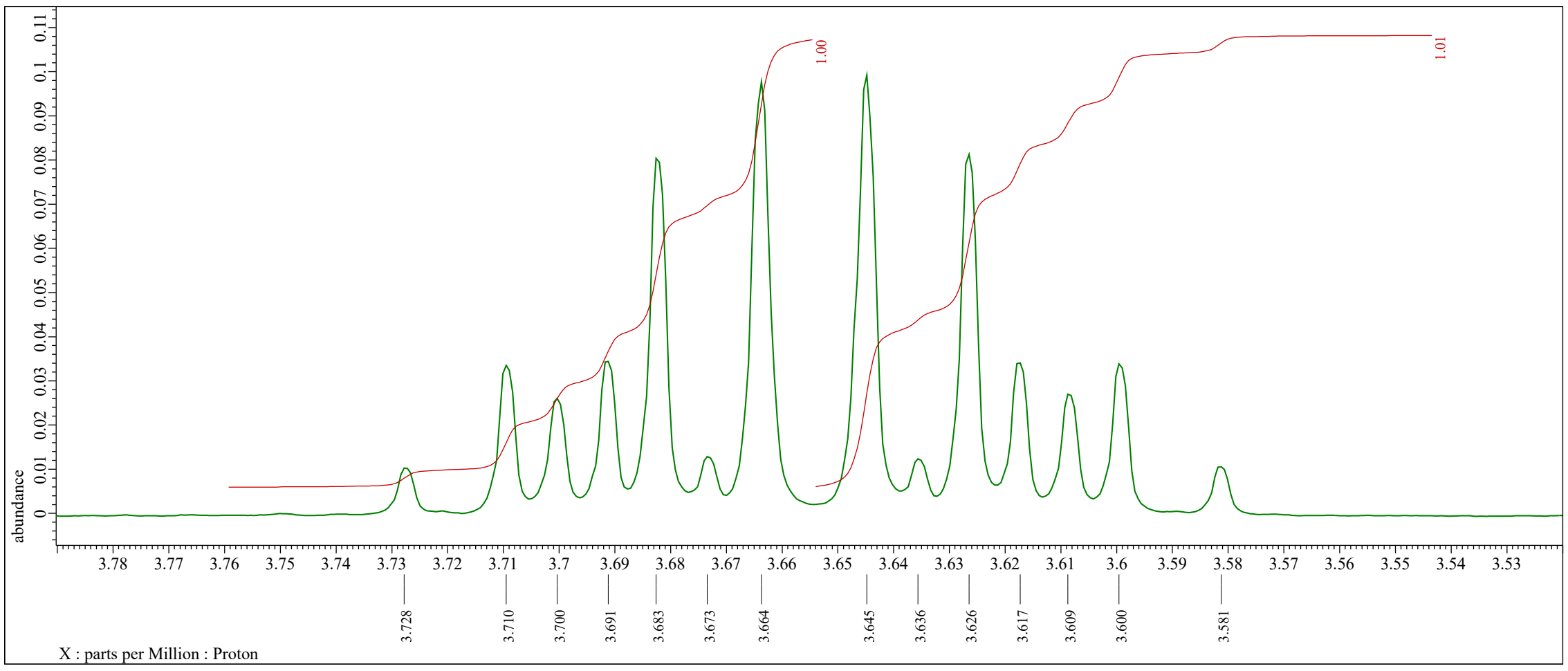

^{13}C{^{1}H} NMR (100 MHz, $CDCl_3$)

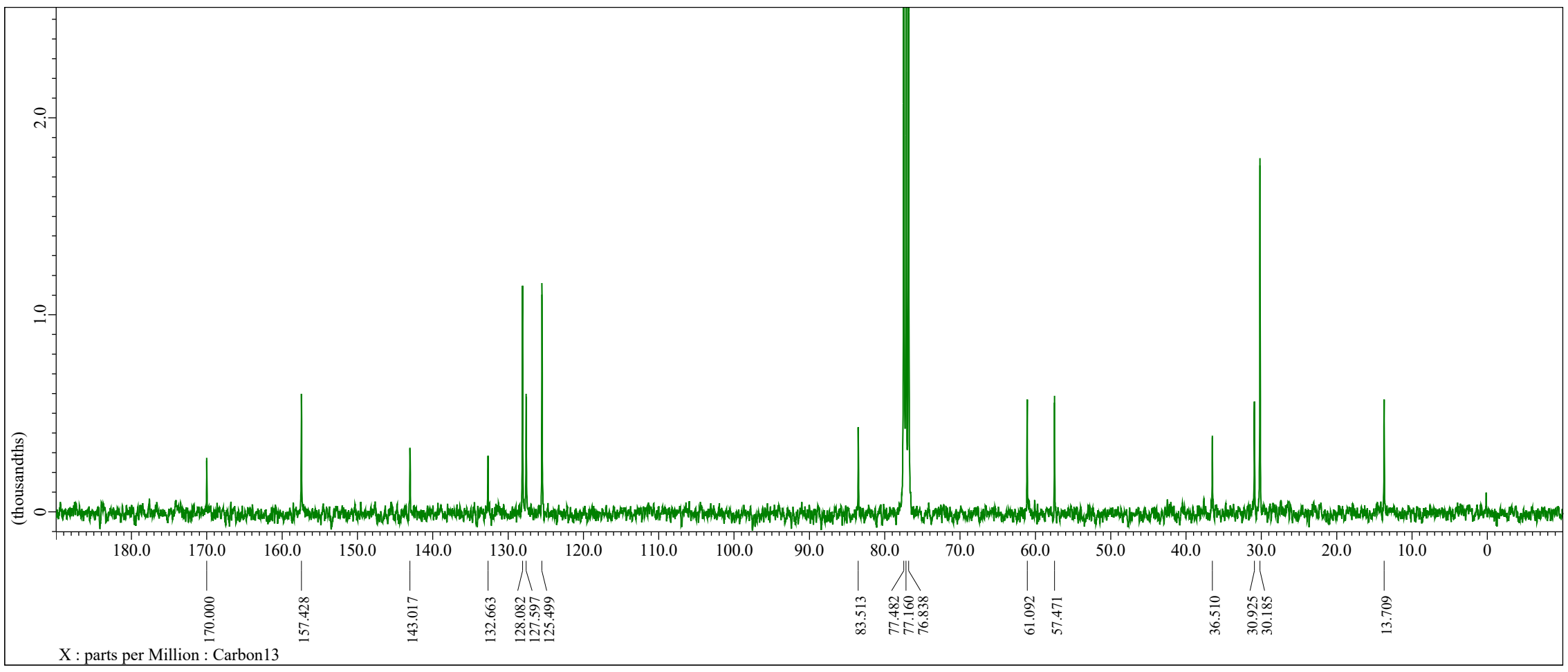

3-4. *anti-γ*-Selective aldol reaction of allenoate 1a with aldehyde 3 using $GeCl_2$-dioxane

Ethyl (*Z*)-2-{(4*R,5*S**)-4-(*tert*-butyl)-2,2-dichloro-5-phenyl-1,2-oxagermolan-3-ylidene}acetate *anti-γ*-4aa**

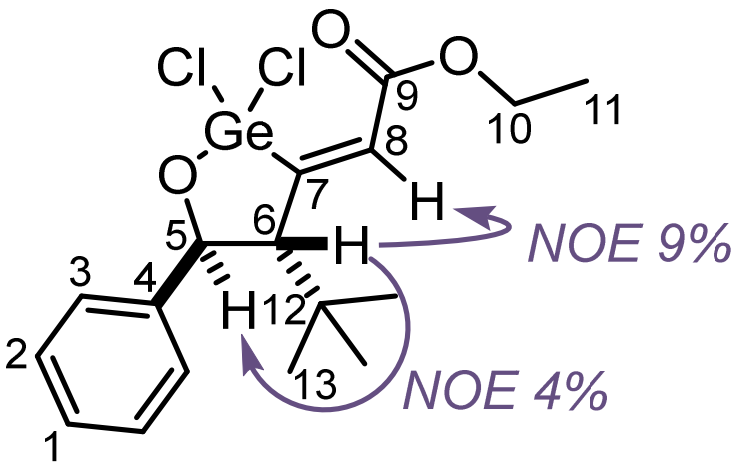

To a mixture of **1a** (0.0339 g, 0.202 mmol) and benzaldehyde **3a** (0.0202 mL, 0.200 mmol) in hexane (1.0 mL) was added $GeCl_2$-dioxane (0.0232 g, 0.100 mmol). After the reaction mixture was stirred at room temperature for 18 h, the solvent was removed under vacuum. The yield was determined by ^{1}H NMR of the crude product using 1,1,2,2-tetrachloroethane as an internal standard (97%, *anti-α*-**4aa**/*anti-γ*-**4aa** = 12/88, single diastereomer).

To obtain the spectrodata of the isolated product, recrystallization from hexane was performed to give *anti-γ*-**4aa** in pure form as a colorless solid.

mp 101.5–102.2 °C; **IR** (KBr) 3029 (w), 2966 (s), 1687 (m), 1373 (m), 1328 (s), 1257 (m), 1216 (m), 1102 (m), 1009 (m) cm^{-1}; **^{1}H NMR** (400 MHz, $CDCl_3$) 7.36–7.30 (m, 4H), 7.25–7.21 (m, 1H, 1-H), 6.62 (d, *J* = 1.6 Hz, 1H, 8-H), 5.59 (s, 1H, 5-H), 4.45 (dq, *J* = 10.8, 7.2 Hz, 1H, 10-H), 4.39 (dq, *J* = 11.0, 7.4 Hz, 1H, 10-H), 2.86 (dd, *J* = 1.6, 0.9 Hz, 1H, 6-H), 1.38 (t, *J* = 7.3 Hz, 3H, 11-H), 1.11 (s, 9H, 13-H); **^{13}C{^{1}H} NMR** (100 MHz, $CDCl_3$) 167.2 (s, C-9), 157.1 (s, C-7), 144.9 (s, C-4), 128.8 (d, C-8), 128.6 (d, C-2), 127.4 (d, C-1), 125.8 (d, C-3), 77.8 (d, C-5), 63.7 (t, C-10), 59.6 (d, C-6), 36.1 (s, C-12), 28.2 (q, C-13), 14.3 (q, C-11); **HRMS** (DART$^+$) Calculated ($C_{17}H_{23}O_3Cl_2Ge$): 419.0231 ([M+H]$^+$), Found: 419.0246 ([M+H]$^+$).

Large-scale synthesis

To a mixture of **1a** (0.337 g, 2.00 mmol) and benzaldehyde **3a** (0.202 mL, 2.00 mmol) in hexane (5.0 mL) was added $GeCl_2$-dioxane (0.232 g, 1.00 mmol). After the reaction mixture was stirred at room temperature for 18 h, the solvent was removed under vacuum. The yield was determined by ^{1}H NMR of the crude product using 1,1,2,2-tetrachloroethane as an internal standard (100%, *anti-α*-**4aa**/*anti-γ*-**4aa** = 5/95, single diastereomer).

^{1}H NMR (400 MHz, $CDCl_3$)

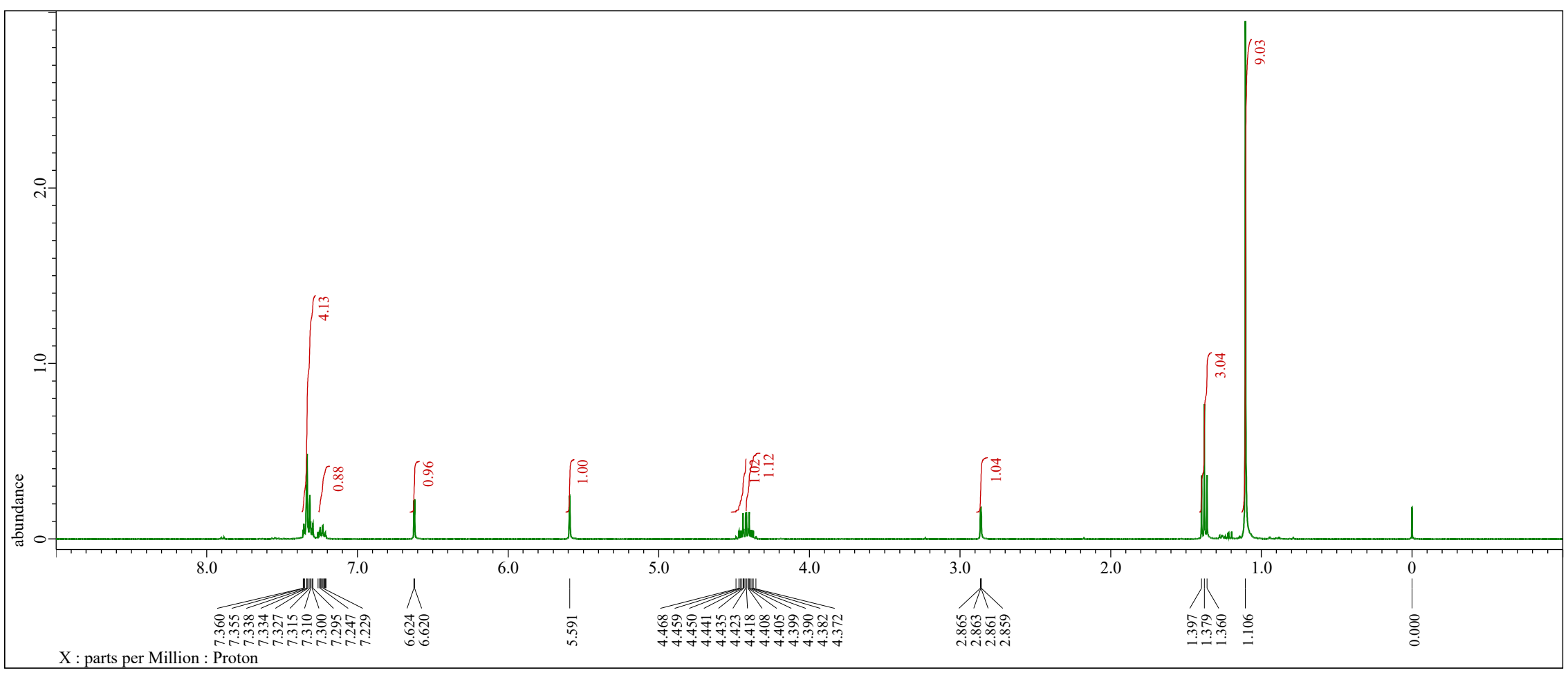

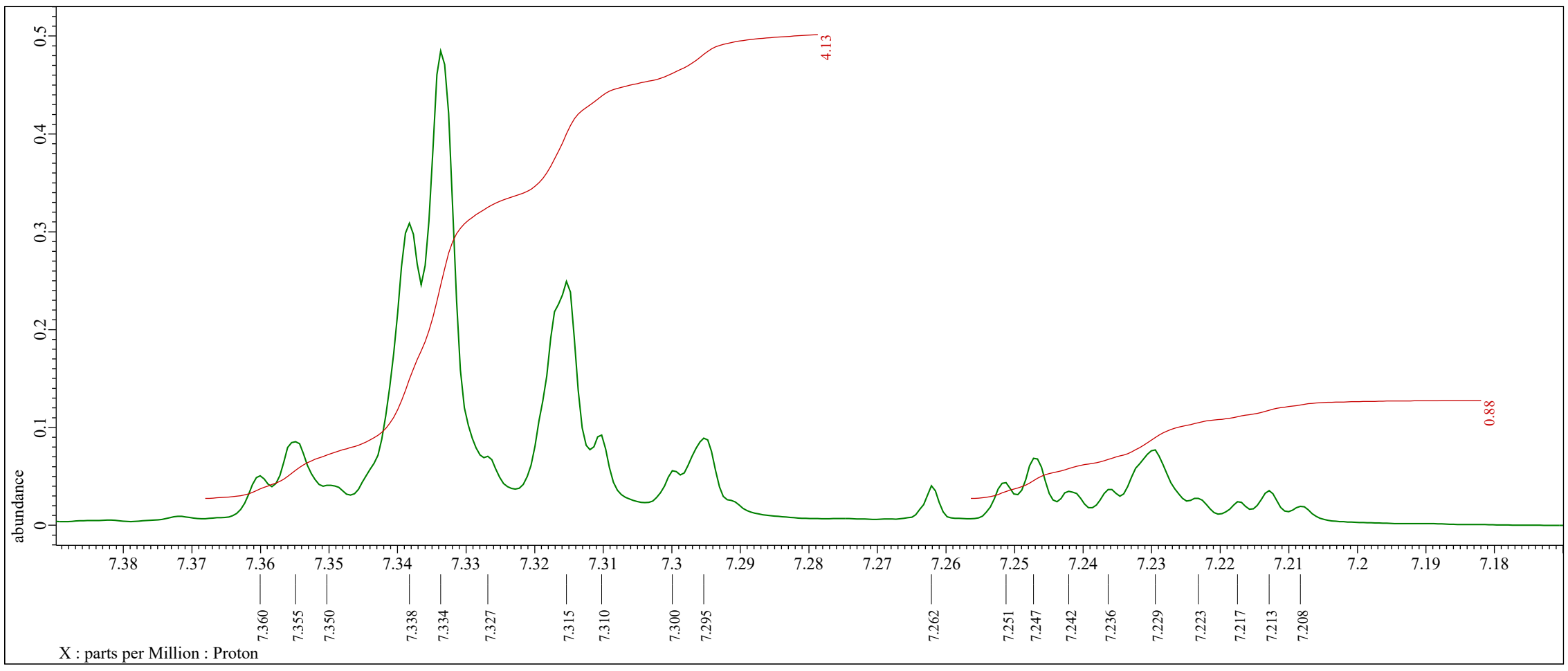

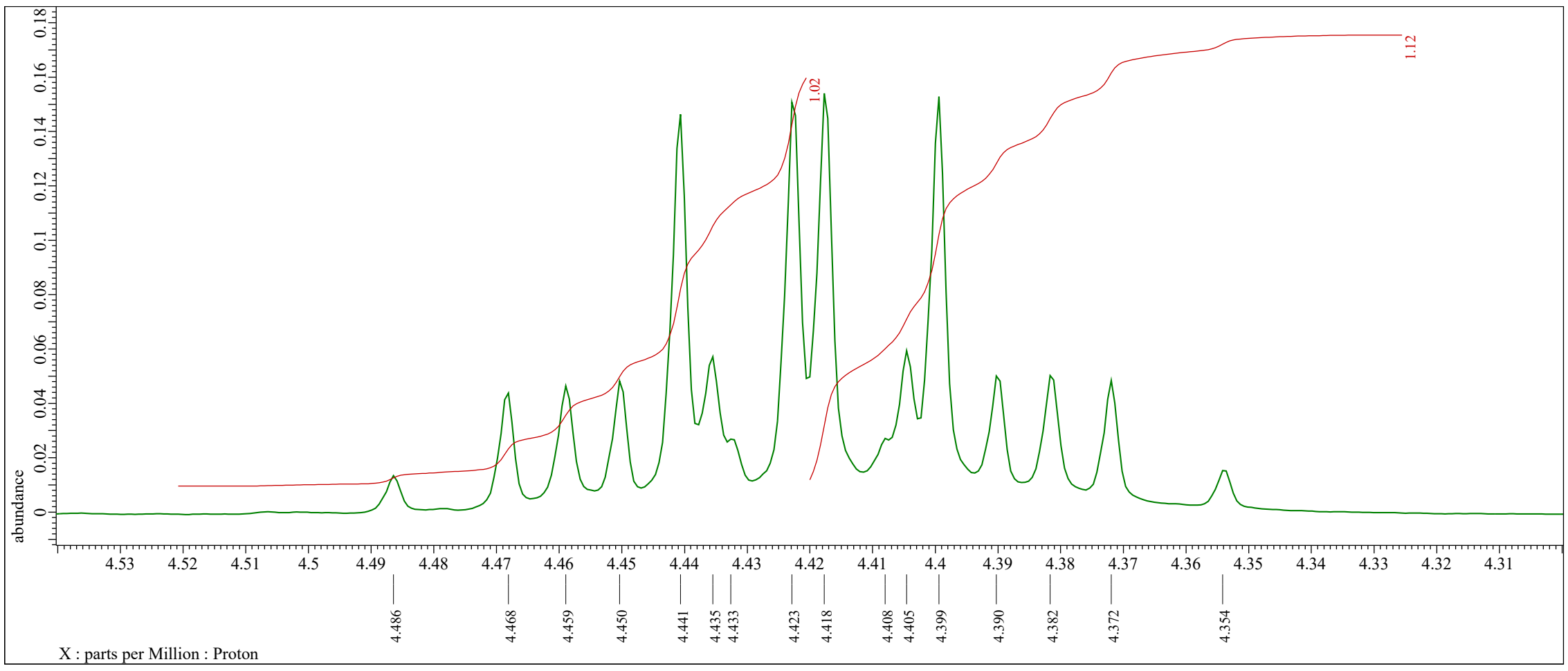

$^{13}C\{^{1}H\}$ NMR (100 MHz, $CDCl_3$)

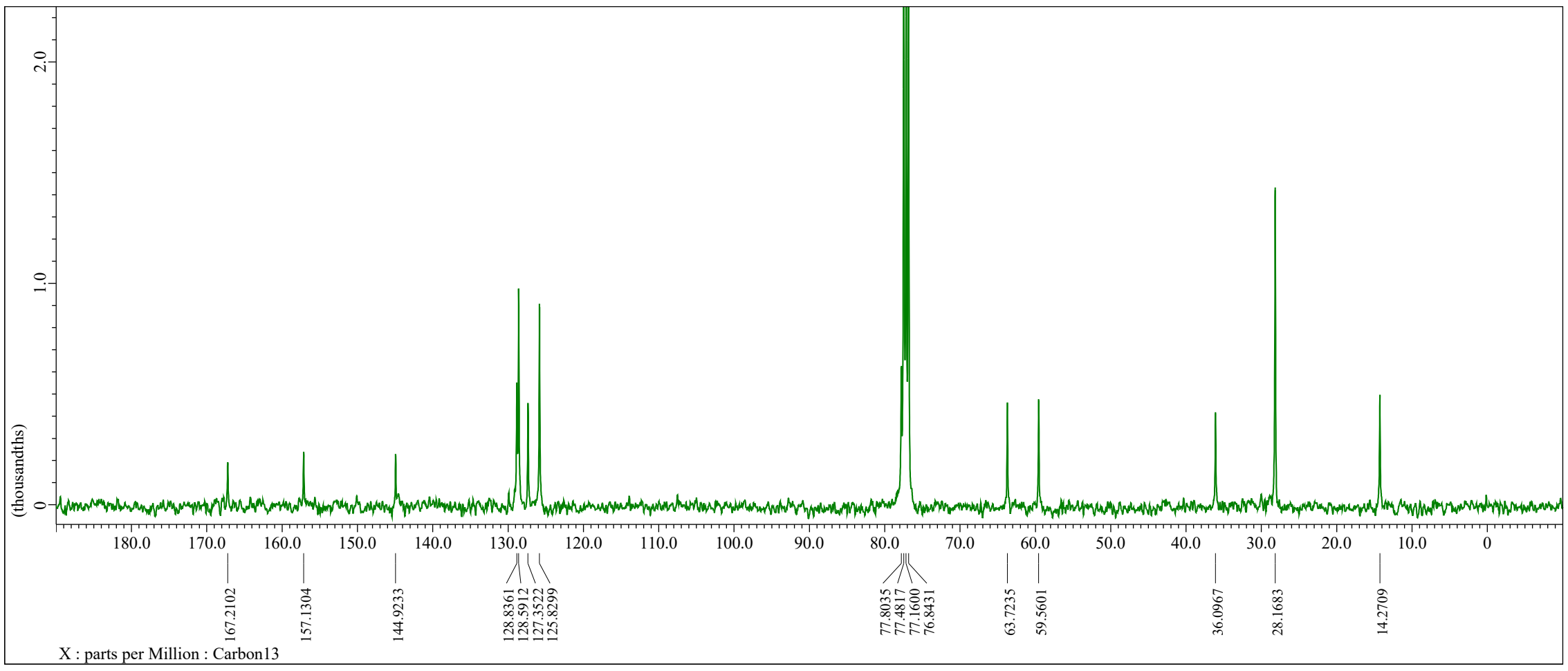

Ethyl (*Z*)-2-{(4*R,5*S**)-4-(*tert*-butyl)-2,2-dichloro-5-(4-methoxyphenyl)-1,2-oxagermolan-3-ylidene)acetate *anti*-γ-4ab**

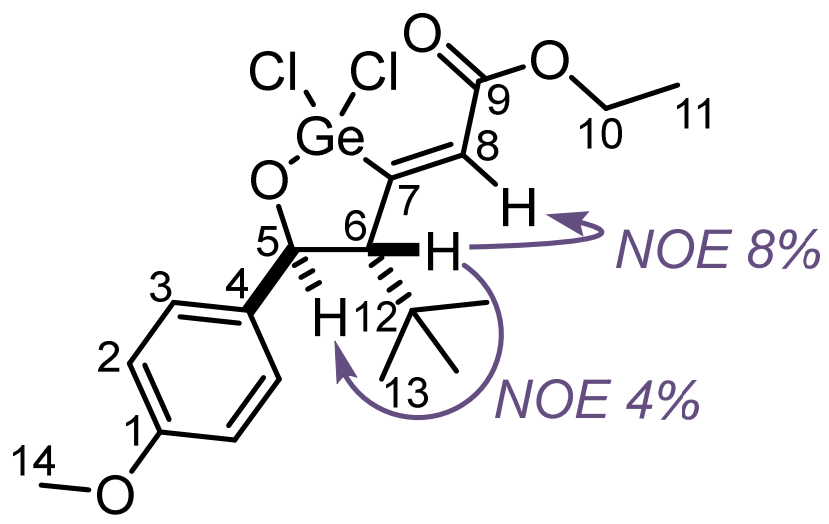

To a mixture of **1a** (0.0337 g, 0.200 mmol) and 4-methoxybenzaldehyde **3b** (0.0243 mL, 0.200 mmol) in hexane (1.0 mL) was added $GeCl_2$-dioxane (0.0232 g, 0.100 mmol). After the reaction mixture was stirred at room temperature for 18 h, the solvent was removed under vacuum. The yield was determined by ^{1}H NMR of the crude product using 1,1,2,2-tetrachloroethane as an internal standard (82%, *anti*-α-**4ab**/*anti*-γ-**4ab** = 22/78, single diastereomer).

To obtain the spectrodata of the isolated product, some conditions were modified. To a mixture of **1a** (1.1 eq) and 4-methoxybenzaldehyde **3b** (0.095 eq) in hexane (1.0 mL) was added $GeCl_2$-dioxane (1.0 eq). After the reaction mixture was stirred at room temperature for 18 h, the solvent was removed under vacuum. Careful extraction of the crude mixture with hexane gave *anti*-γ-**4ab** in pure form as colorless neat.

IR (KBr) 3075 (w), 2964 (m), 1687 (m), 1513 (s), 1329 (s), 1253 (s), 1176 (m), 1032 (m), 830 (m) cm^{-1}; **^{1}H NMR** (400 MHz, $CDCl_3$) 7.25 (d, J = 8.8 Hz, 2H, 3-H), 6.84 (d, J = 9.2 Hz, 2H, 2-H), 6.63 (d, J = 2.0 Hz, 1H, 8-H), 5.55 (s, 1H, 5-H), 4.44 (dq, J = 11.0, 7.0 Hz, 1H, 10-H), 4.39 (dq, J = 11.0, 7.4 Hz, 1H, 10-H), 3.78 (s, 3H, 14-H), 2.85 (dd, J = 1.6, 0.8 Hz, 1H, 6-H), 1.38 (t, J = 7.2 Hz, 3H, 11-H), 1.09 (s, 9H, 13-H); **^{13}C{^{1}H} NMR** (100 MHz, $CDCl_3$) 167.1 (s, C-9), 158.8 (s, C-1), 157.3 (s, C-7), 137.0 (s, C-4), 128.8 (d, C-8), 127.1 (d, C-3), 113.8 (d, C-2), 77.4 (d, C-5), 63.6 (t, C-10), 59.4 (d, C-6), 55.3 (q, C-14), 36.0 (s, C-12), 28.1 (q, C-13), 14.2 (q, C-11); **HRMS** (DART$^+$) Calculated ($C_{18}H_{25}O_4Cl_2Ge$): 449.0336 ([M+H]$^+$), Found: 449.0352 ([M+H]$^+$).

^{1}H NMR (400 MHz, $CDCl_3$)

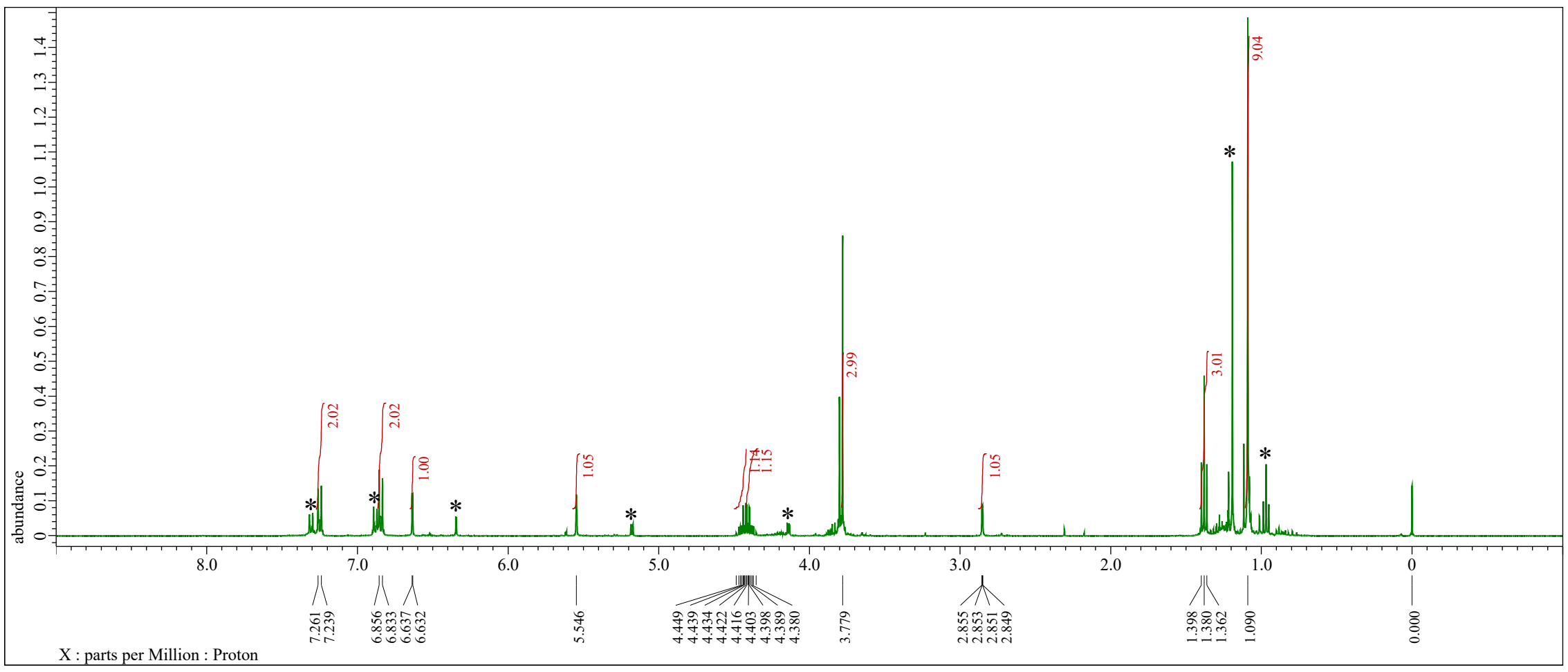

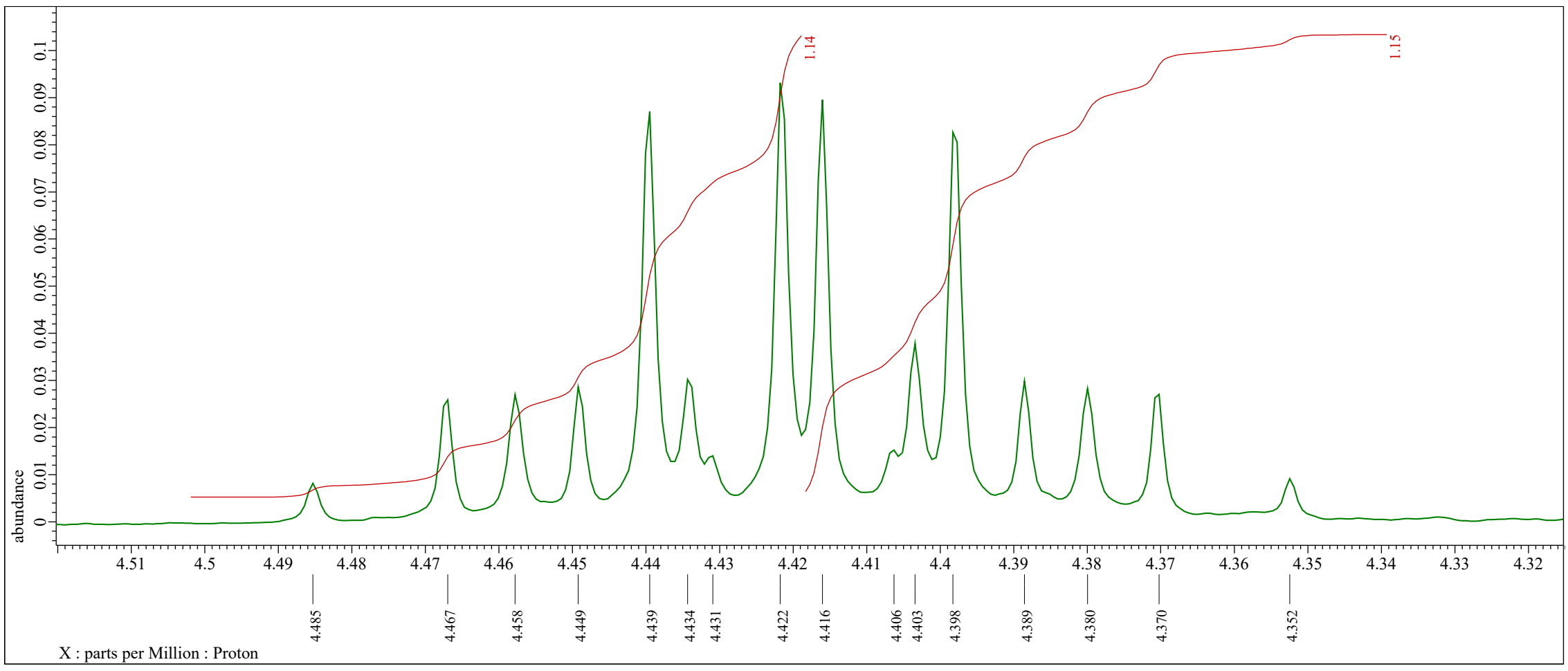

$^{13}C\{^1H\}$ NMR (100 MHz, $CDCl_3$)

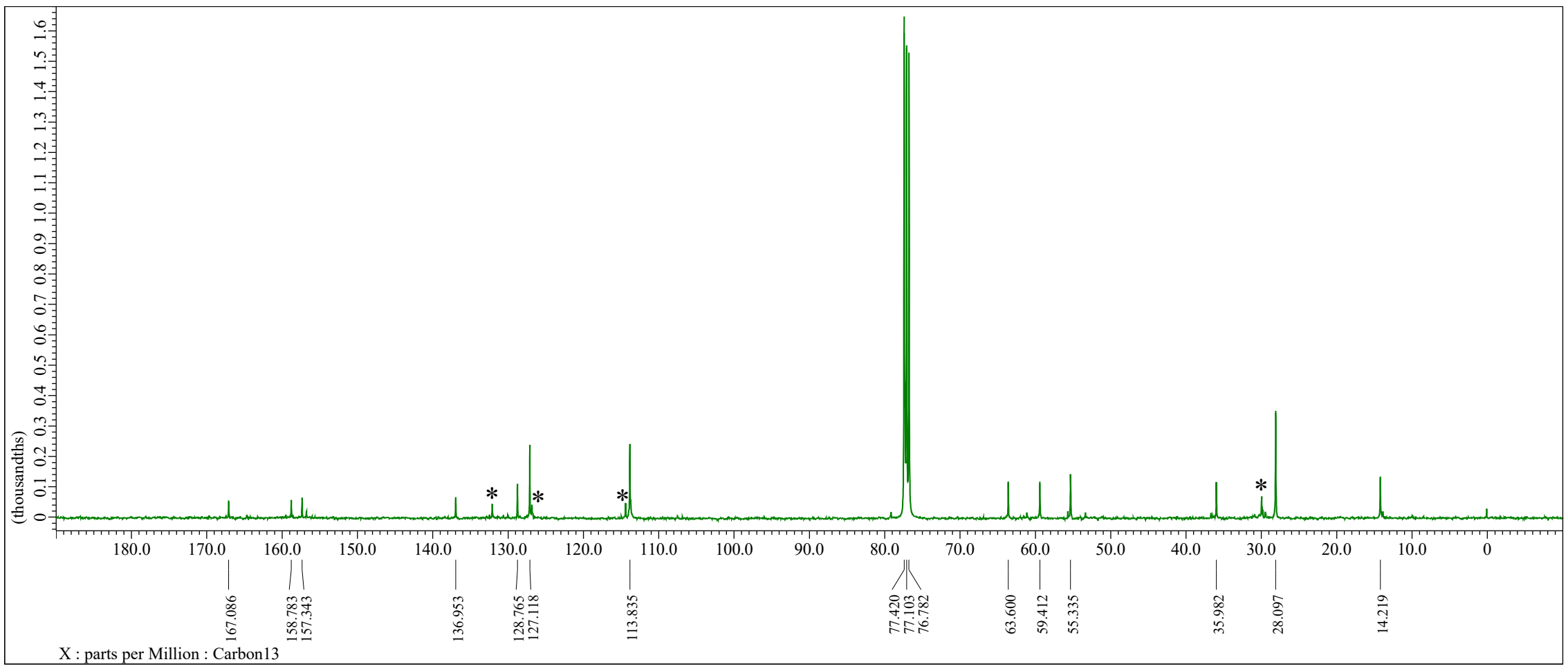

Asterisk marks (*) represent the signals of the inseparatable *anti*-*α*-**4ab**.

Methyl 4-{(4*R,5*S**,*Z*)-4-(*tert*-butyl)-2,2-dichloro-3-(2-ethoxy-2-oxoethylidene)-1,2-oxagermolan-5-yl}benzoate** *anti*-*γ*-**4ac**

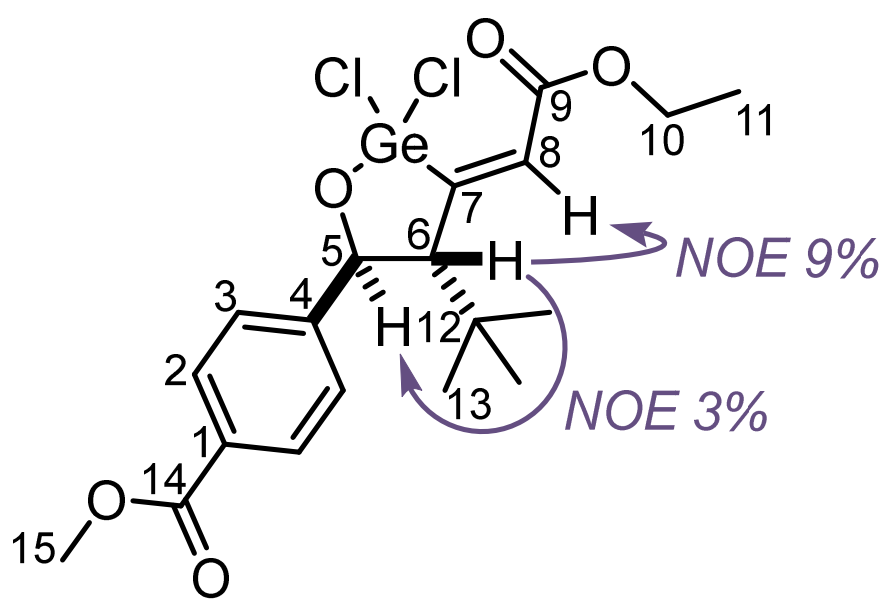

To a mixture of **1a** (0.0335 g, 0.199 mmol) and methyl 4-formylbenzoate **3c** (0.0328 g, 0.200 mmol) in hexane (1.0 mL) was added $GeCl_2$-dioxane (0.0233 g, 0.101 mmol). After the reaction mixture was stirred at room temperature for 18 h, the solvent was removed under vacuum. The yield was determined by ^1H NMR of the crude product using 1,1,2,2-tetrachloroethane as an internal standard (86%, *anti*-*α*-**4ac**/*anti*-*γ*-**4ac** = 9/91, single diastereomer).

To obtain the spectrodata of the isolated product, some conditions were modified. To a mixture of **1a** (1.1 eq) and methyl 4-formylbenzoate **3c** (0.095 eq) in hexane (1.0 mL) was added $GeCl_2$-dioxane (1.0 eq). After the reaction mixture was stirred at room temperature for 18 h, the solvent was removed under vacuum. Careful extraction of the crude mixture with hexane gave *anti*-*γ*-**4ac** in pure form as a colorless solid.

mp 90.0–91.0 °C; **IR** (KBr); 3033 (w), 2960 (m), 1722 (s), 1373 (m), 1330 (s), 1281 (s), 1108 (m), 1017 (m), 882 (m) cm^{-1}; **^{1}H NMR** (400 MHz, $CDCl_3$) 7.99 (d, *J* = 8.8 Hz, 2H, 2-H), 7.44 (d, *J* = 8.4 Hz, 2H, 3-H), 6.62 (d, *J* = 1.6 Hz, 1H, 8-H), 5.62 (s, 1H, 5-H), 4.45 (dq, *J* = 10.8, 7.2 Hz, 1H, 10-H), 4.40 (d, *J* = 11.0, 7.0 Hz, 1H, 10-H), 3.90 (s, 3H, 15-H), 2.83 (dd, *J* = 1.6, 0.8 Hz, 1H, 6-H), 1.38 (t, *J* = 7.2 Hz, 3H, 11-H), 1.12 (s, 9H, 13-H); **^{13}C{^{1}H} NMR** (100 MHz, $CDCl_3$) 167.3 (s, C-9), 167.0 (s, C-14), 156.7 (s, C-7), 150.1 (s, C-4), 129.9 (d, C-2), 129.1 (s, C-1), 128.9 (d, C-8), 125.8 (d, C-3), 77.4 (d, C-5), 63.9 (t, C-10), 59.5 (d, C-6), 52.3 (q, C-15), 36.2 (s, C-12), 28.2 (q, C-13), 14.3 (q, C-11); **HRMS** (DART$^+$) Calculated ($C_{19}H_{25}O_5Cl_2Ge$): 477.0285 ($[M+H]^+$), Found: 477.0282 ($[M+H]^+$).

^{1}H NMR (400 MHz, $CDCl_3$)

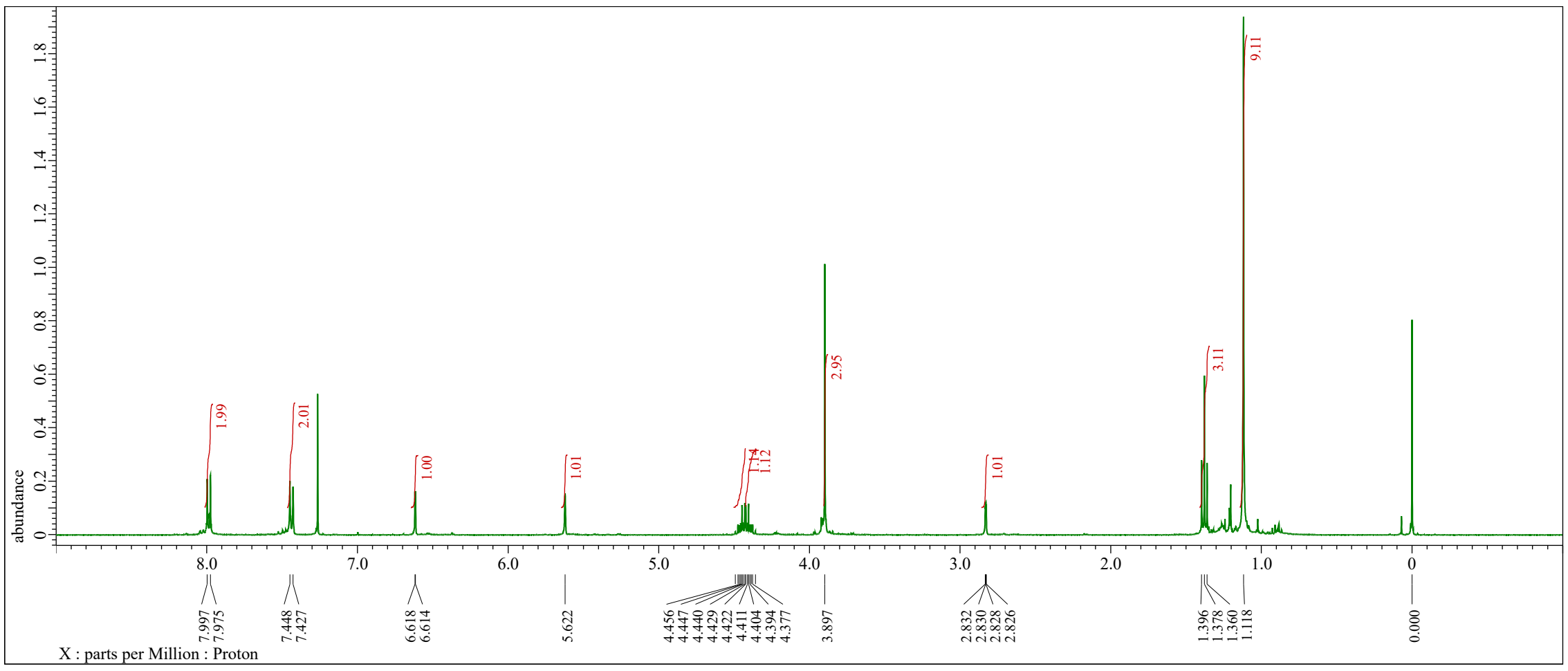

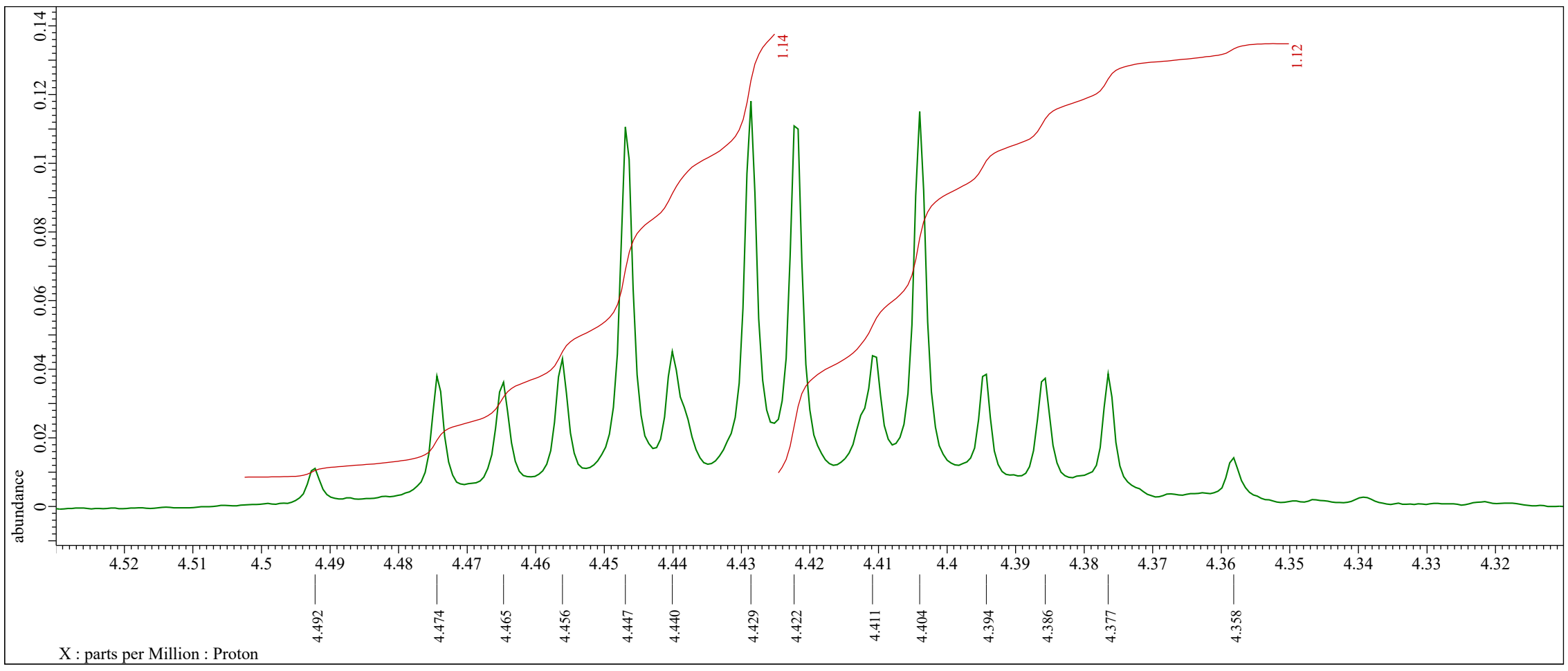

^{13}C{^{1}H} NMR (100 MHz, $CDCl_3$)

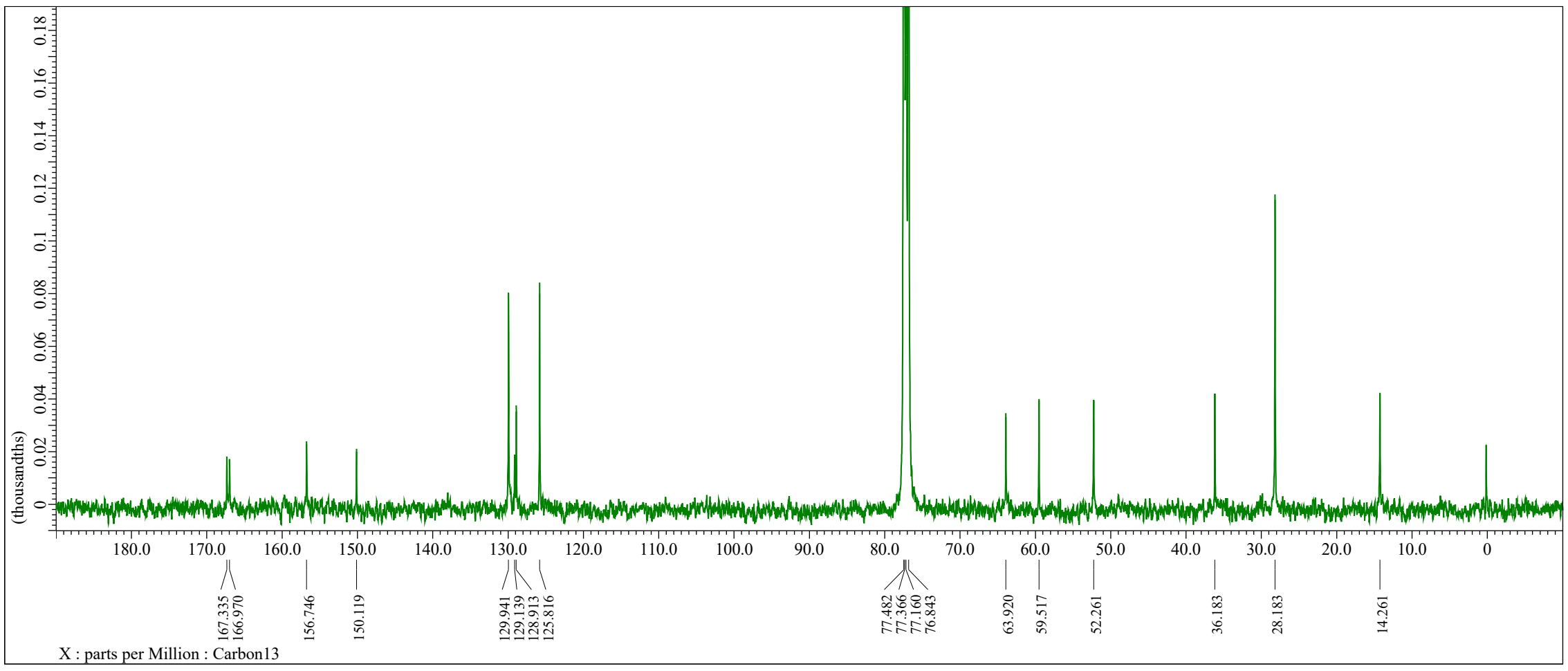

Ethyl (*Z*)-2-{(4*R,5*S**)-4-(*tert*-butyl)-2,2-dichloro-5-(*o*-tolyl)-1,2-oxagermolan-3-ylidene}acetate *anti*-*γ*-4ad**

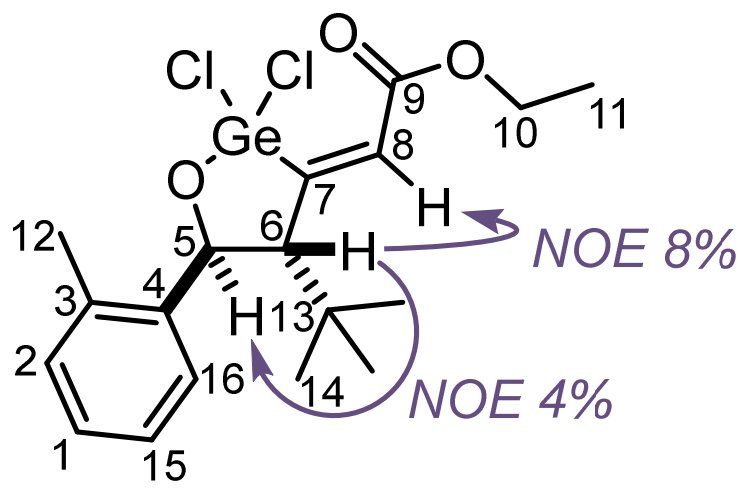

To a mixture of **1a** (0.0336 g, 0.200 mmol) and 2-methylbenzaldehyde **3d** (0.0231 mL, 0.200 mmol) in hexane (1.0 mL) was added $GeCl_2$-dioxane (0.0231 g, 0.0997 mmol). After the reaction mixture was stirred at room temperature for 18 h, the solvent was removed under vacuum. The yield was determined by ^{1}H NMR of the crude product using 1,1,2,2-tetrachloroethane as an internal standard (72%, *anti*-*α*-**4ad**/*anti*-*γ*-**4ad** = 16/84, single diastereomer).

To obtain the spectrodata of the isolated product, some conditions were modified. To a mixture of **1a** (1.0 eq) and 2-methylbenzaldehyde **3d** (1.2 eq) in hexane (1.0 mL) was added $GeCl_2$-dioxane (1.0 eq). After the reaction mixture was stirred at room temperature for 18 h, the generated white solid was filtered off with hexane. The filtrate was evaporated to give *anti*-*γ*-**4ad** in pure form as colorless neat.

IR (KBr) 3064 (w), 2964 (s), 1688 (s), 1466 (m), 1328 (s), 1216 (s), 1092 (m), 1027 (m), 883 (m) cm^{-1}; **^{1}H NMR** (400 MHz, $CDCl_3$) 7.21–7.08 (m, 3H), 6.93–6.91 (m, 1H), 6.78 (d, *J* = 1.6 Hz, 1H, 8-H), 5.75 (s, 1H, 5-H), 4.48 (dq, *J* = 11.0, 7.4 Hz, 1H, 10-H), 4.44 (dq, *J* = 10.8, 7.6 Hz, 1H, 10-H), 3.03 (s, 1H, 6-H), 2.54 (s, 3H, 12-H), 1.42 (t, *J* = 7.2 Hz, 3H, 11-H), 1.07 (s, 9H, 14-H); **^{13}C{^{1}H} NMR** (100 MHz, $CDCl_3$) 167.3 (s, C-9), 159.1 (s), 141.8 (s), 136.8 (s), 131.1 (d), 127.9 (d), 127.8 (d, C-8), 126.2 (d), 126.1 (d), 74.2 (d, C-5), 63.7 (t, C-10), 56.6 (d, C-6), 36.2 (s, C-13), 28.1 (q, C-14), 19.8 (q, C-12), 14.3 (q, C-11); **HRMS** (DART^{+}) Calculated ($C_{18}H_{25}O_3Cl_2Ge$): 433.0387 ($[M+H]^{+}$), Found: 433.0382 ($[M+H]^{+}$).

^{1}H NMR (400 MHz, $CDCl_3$)

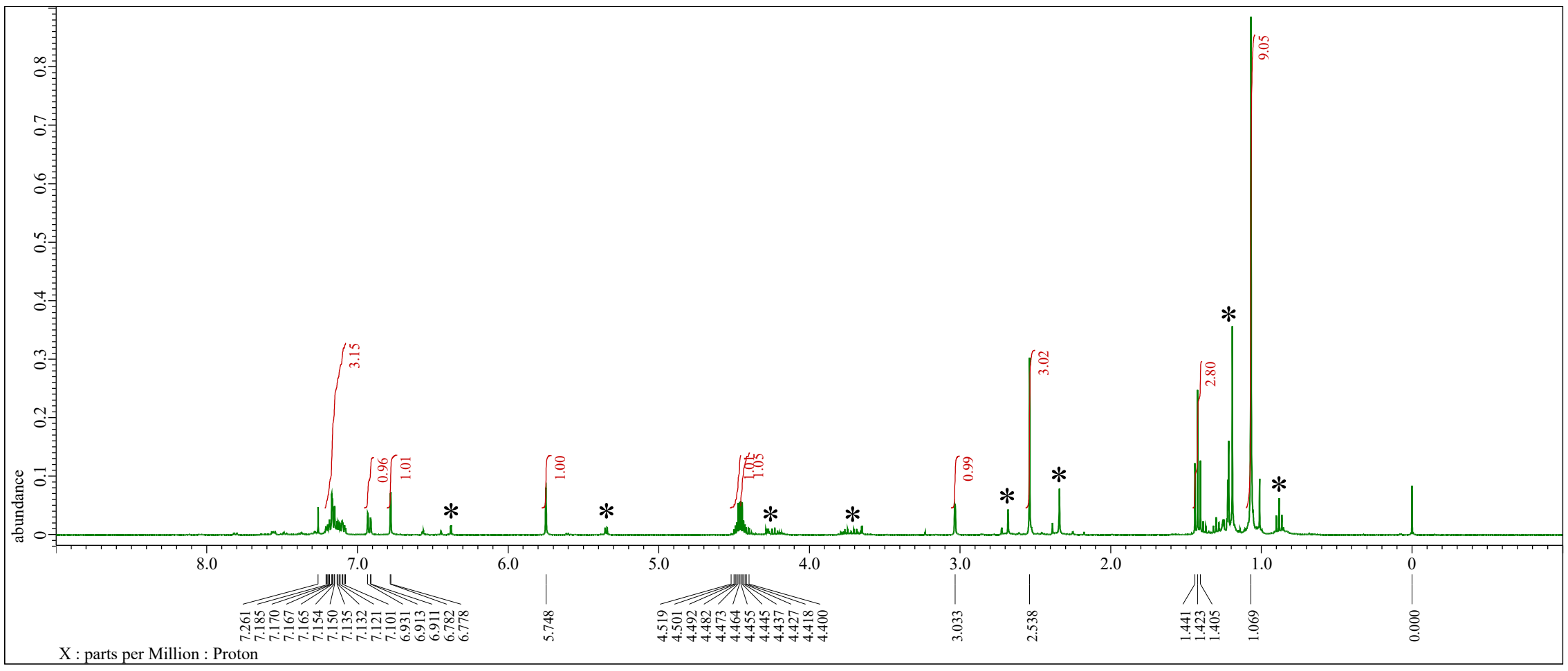

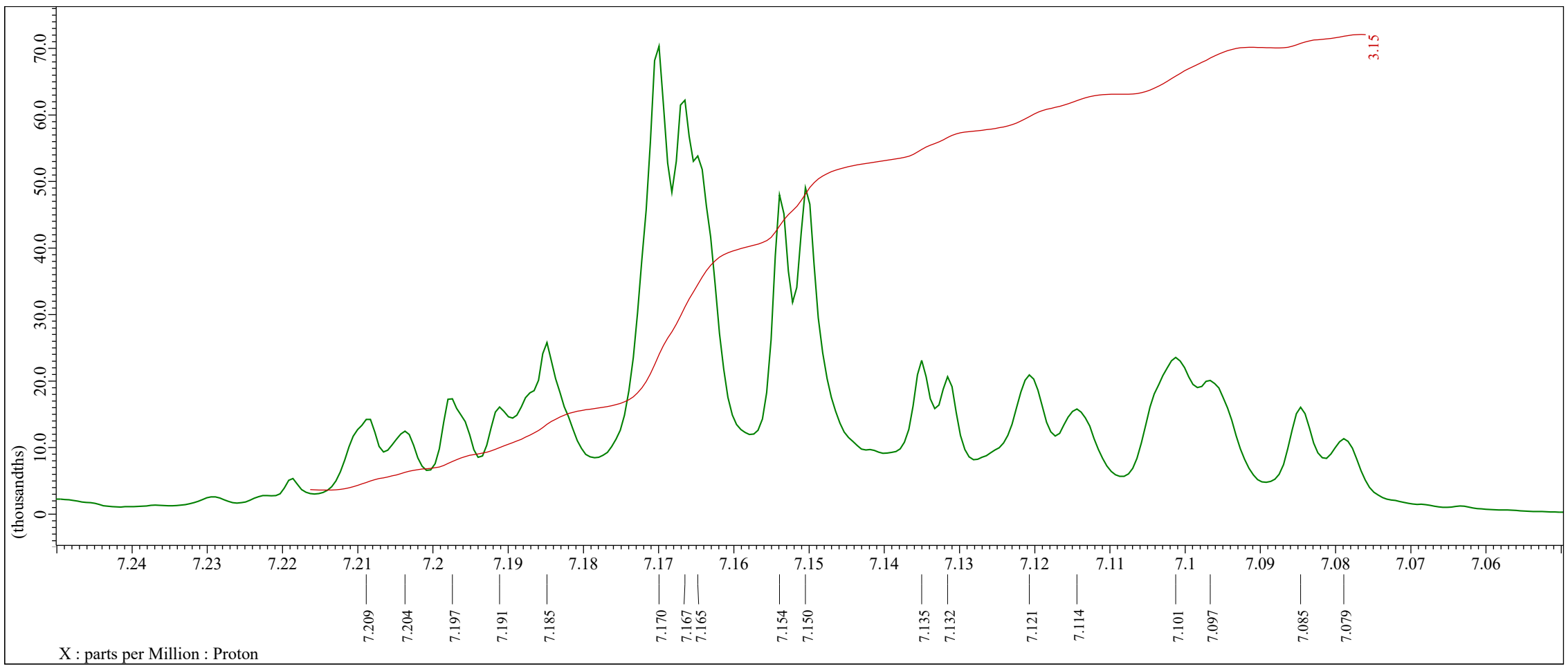

^{13}C{^{1}H} NMR (100 MHz, $CDCl_3$)

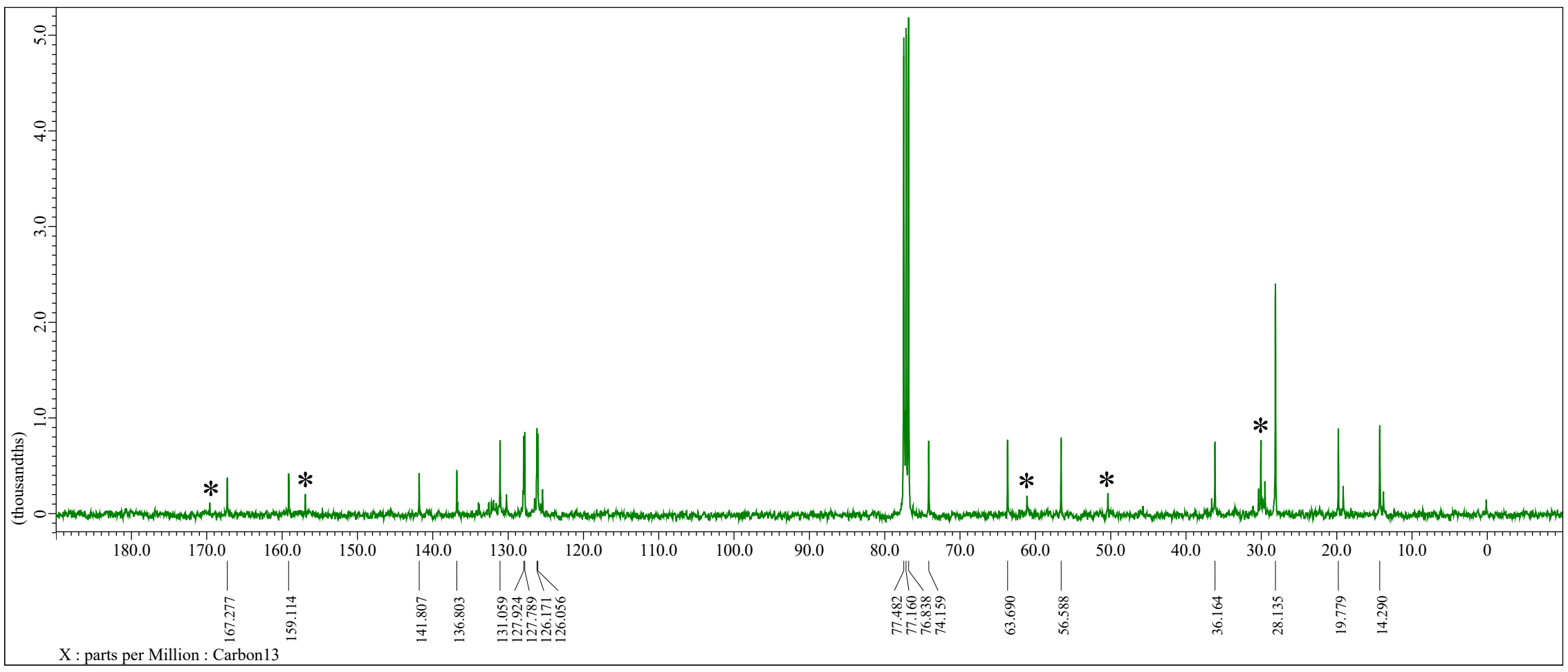

Asterisk marks (*) represent the signals of the inseparatable *anti*-*α*-**4ad**.

Ethyl (*Z*)-2-{(4*R,5*S**)-4-(*tert*-butyl)-2,2-dichloro-5-(*m*-tolyl)-1,2-oxagermolan-3-ylidene}acetate *anti*-*γ*-4ae**

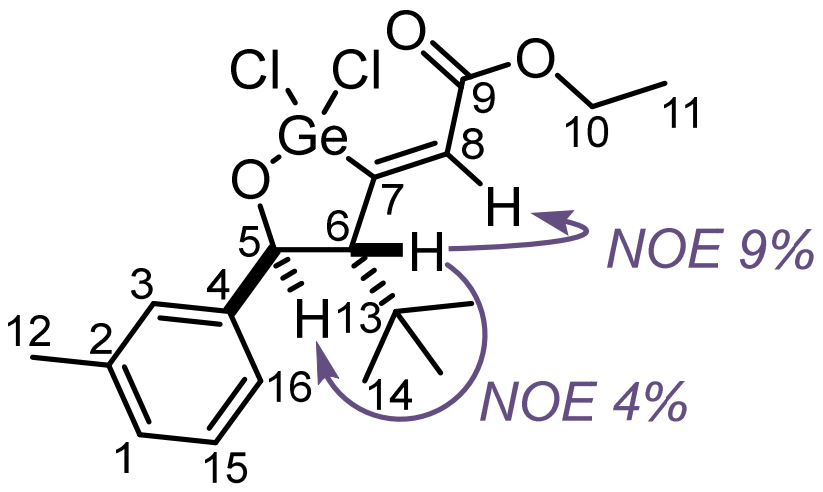

To a mixture of **1a** (0.0335 g, 0.199 mmol) and 3-methylbenzaldehyde **3e** (0.0236 mL, 0.200 mmol) in hexane (1.0 mL) was added $GeCl_2$-dioxane (0.0232 g, 0.100 mmol). After the reaction mixture was stirred at room temperature for 18 h, the solvent was removed under vacuum. The yield was determined by ^{1}H NMR of the crude product using 1,1,2,2-tetrachloroethane as an internal standard (93%, *anti*-*α*-**4ae**/*anti*-*γ*-**4ae** = 11/89, single diastereomer).

To obtain the spectrodata of the isolated product, some conditions were modified. To a mixture of **1a** (1.0 eq) and 3-methylbenzaldehyde **3e** (1.2 eq) in hexane (1.0 mL) was added $GeCl_2$-dioxane (1.0 eq). After the reaction mixture was stirred at room temperature for 16 h, the generated white solid was filtered off with hexane. The filtrate was evaporated to give *anti*-*γ*-**4ae** in pure form as colorless neat.

IR (KBr) 3099 (w), 2965 (m), 1688 (m), 1472 (m), 1373 (m), 1328 (s), 1216 (m), 1025 (m), 880 (m) cm^{-1}; **^{1}H NMR** (400 MHz, $CDCl_3$) 7.24–7.03 (m, 4H), 6.62 (d, *J* = 1.6 Hz, 1H, 8-H), 5.56 (s, 1H, 5-H), 4.44 (dq, *J* = 11.0, 7.0 Hz, 1H, 10-H), 4.40 (dq, *J* = 11.0, 7.0 Hz, 1H, 10-H), 2.85 (dd, *J* = 1.6, 1.2 Hz, 1H, 6-H), 2.33 (s, 3H, 12-H), 1.38 (t, *J* = 7.2 Hz, 3H, 11-H), 1.10 (s, 9H, 14-H); **^{13}C{^{1}H} NMR** (100 MHz, $CDCl_3$) 167.2 (s, C-9), 157.2 (s), 144.9 (s), 138.3 (s), 128.8 (d, C-8), 128.5 (d), 128.1 (d), 126.6 (d), 122.8 (d), 77.8 (d, C-5), 63.7 (t, C-10), 59.6 (d, C-6), 36.1 (s, C-13), 28.2 (q, C-14), 21.7 (q, C-12), 14.3 (q, C-11); **HRMS** (DART$^+$) Calculated ($C_{18}H_{25}O_3Cl_2Ge$): 433.0387 ([M+H]$^+$), Found: 433.0404 ([M+H]$^+$).

^{1}H NMR (400 MHz, $CDCl_3$)

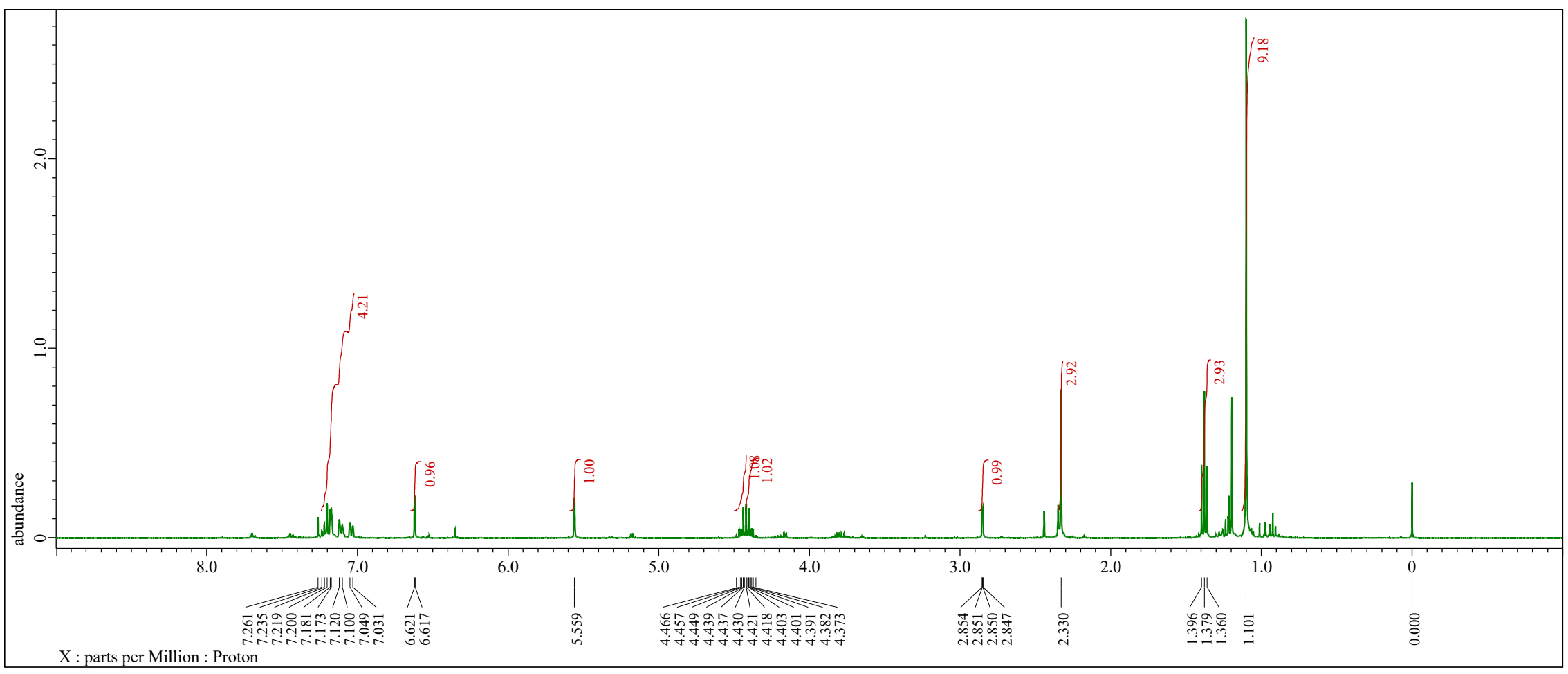

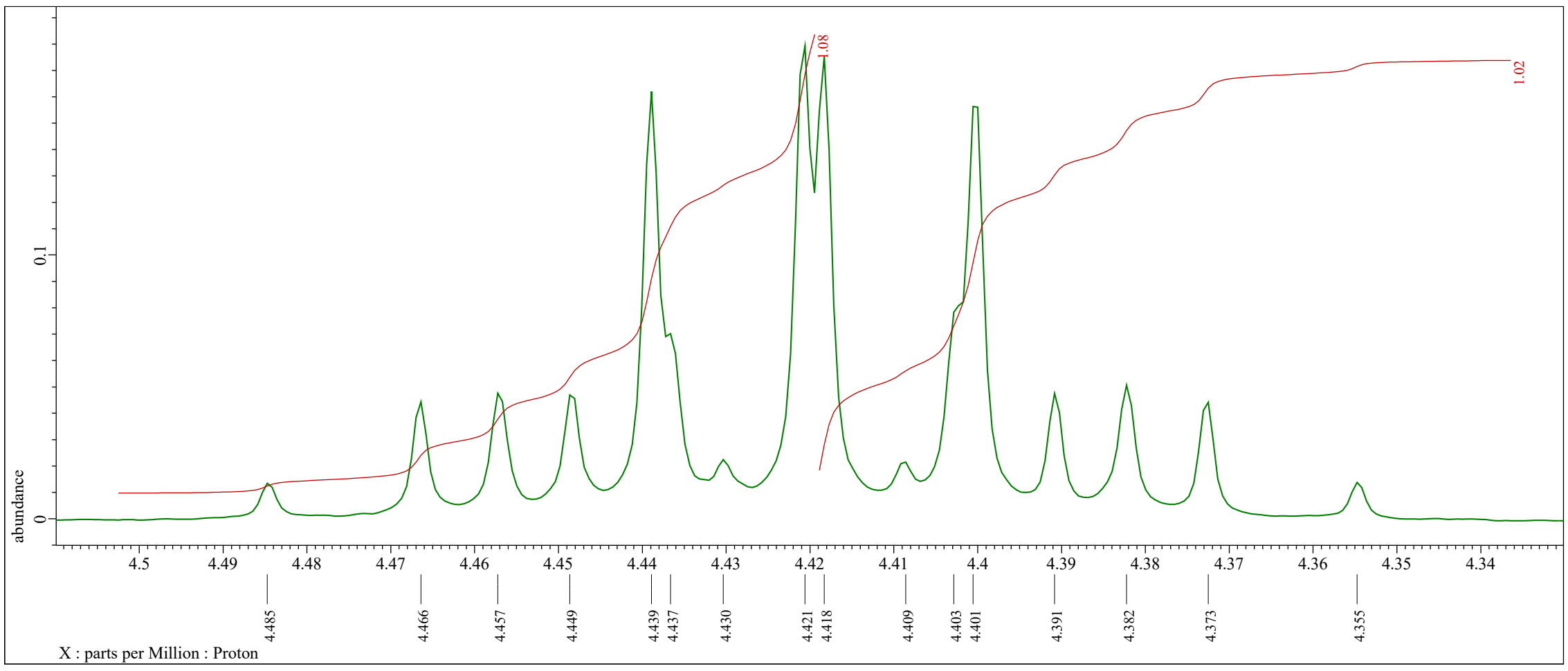

$^{13}C\{^{1}H\}$ **NMR** (100 MHz, $CDCl_3$)

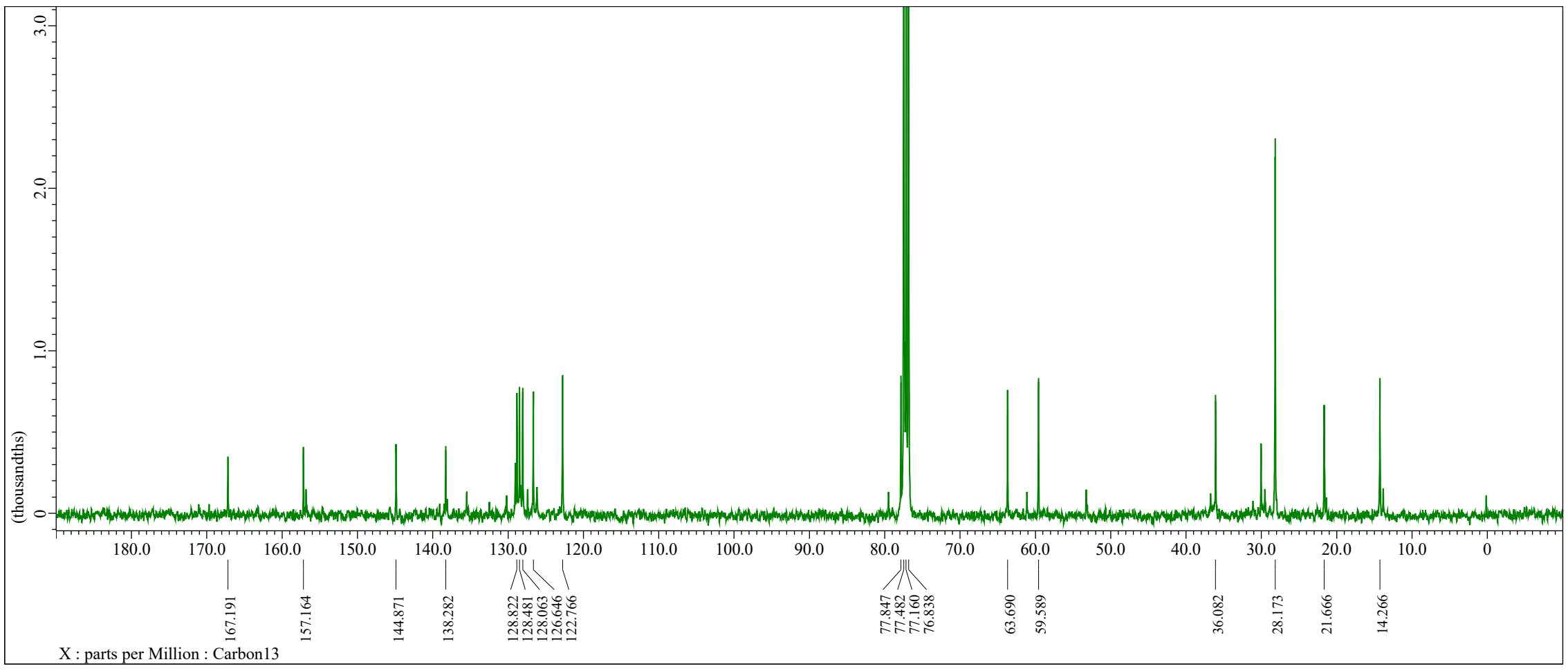

Ethyl (*Z*)-2-{(4*R,5*S**)-4-(*tert*-butyl)-2,2-dichloro-5-(*p*-tolyl)-1,2-oxagermolan-3-ylidene}acetate** *anti*-*γ*-**4af**

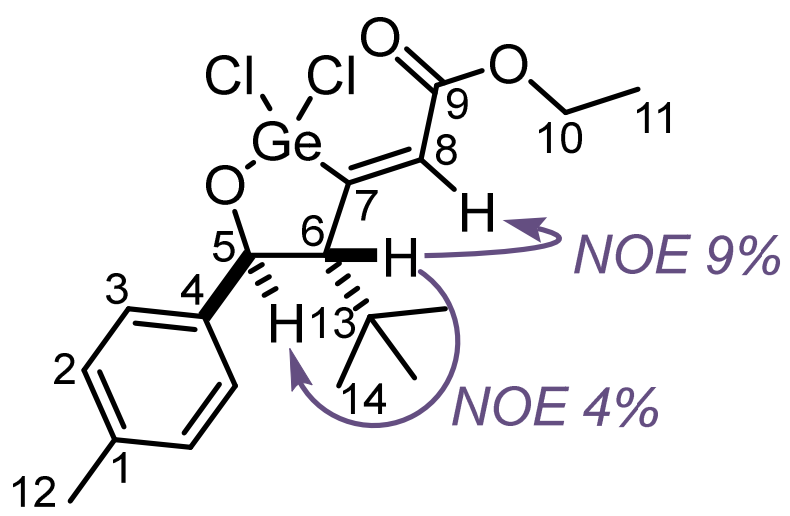

To a mixture of **1a** (0.0336 g, 0.200 mmol) and 4-methylbenzaldehyde **3f** (0.0236 mL, 0.200 mmol) in hexane (1.0 mL) was added $GeCl_2$-dioxane (0.0230 g, 0.0993 mmol). After the reaction mixture was stirred at room temperature for 18 h, the solvent was removed under vacuum. The yield was determined by ^{1}H NMR of the crude product using 1,1,2,2-tetrachloroethane as an internal standard (68%, *anti*-*α*-**4af**/*anti*-*γ*-**4af** = 17/83, single diastereomer).

To obtain the spectrodata of the isolated product, some conditions were modified. To a mixture of **1a** (1.0 eq) and 4-methylbenzaldehyde **3f** (1.2 eq) in hexane (1.0 mL) was added $GeCl_2$-dioxane (1.0 eq). After the reaction mixture was stirred at room temperature for 18 h, the generated white solid was filtered off with hexane. The filtrate was

evaporated to give *anti*-*γ*-**4af** in pure form as colorless neat.

IR (KBr) 3050 (w), 2965 (m), 1688 (m), 1472 (m), 1328 (s), 1215 (m), 1100 (m), 1024 (m), 815 (w) cm^{-1}; ^{1}**H NMR** (400 MHz, $CDCl_3$) 7.23 (d, *J* = 8.0 Hz, 2H), 7.12 (d, *J* = 8.4 Hz, 2H), 6.62 (d, *J* = 1.6 Hz, 1H, 8-H), 5.56 (s, 1H, 5-H), 4.44 (dq, *J* = 10.8, 7.2 Hz, 1H, 10-H), 4.39 (dq, *J* = 11.0, 7.0 Hz, 1H, 10-H), 2.85 (s, 1H, 6-H), 2.31 (s, 3H, 12-H), 1.38 (t, *J* = 7.4 Hz, 3H, 11-H), 1.09 (s, 9H, 14-H); ^{13}**C{^{1}H} NMR** (100 MHz, $CDCl_3$) 167.2 (s, C-9), 157.2 (s), 141.9 (s), 137.0 (s), 129.2 (d), 128.8 (d, C-8), 125.8 (d), 77.7 (d, C-5), 63.7 (t, C-10), 59.6 (d, C-6), 36.0 (s, C-13), 28.2 (q, C-14), 21.2 (q, C-12), 14.3 (q, C-11); **HRMS** (DART^{+}) Calculated ($C_{18}H_{25}O_3Cl_2Ge$): 433.0387 ([M+H]$^{+}$), Found: 433.0396 ([M+H]$^{+}$).

^{1}**H NMR** (400 MHz, $CDCl_3$)

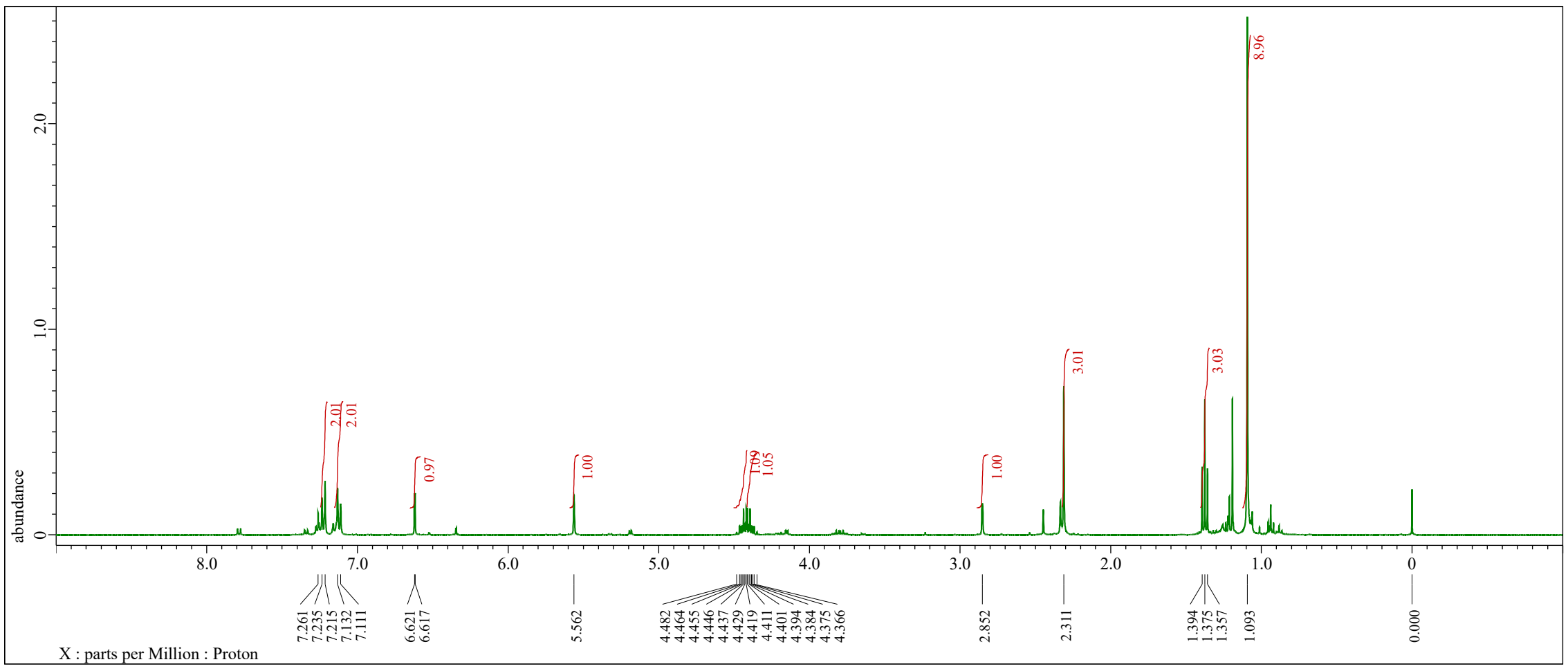

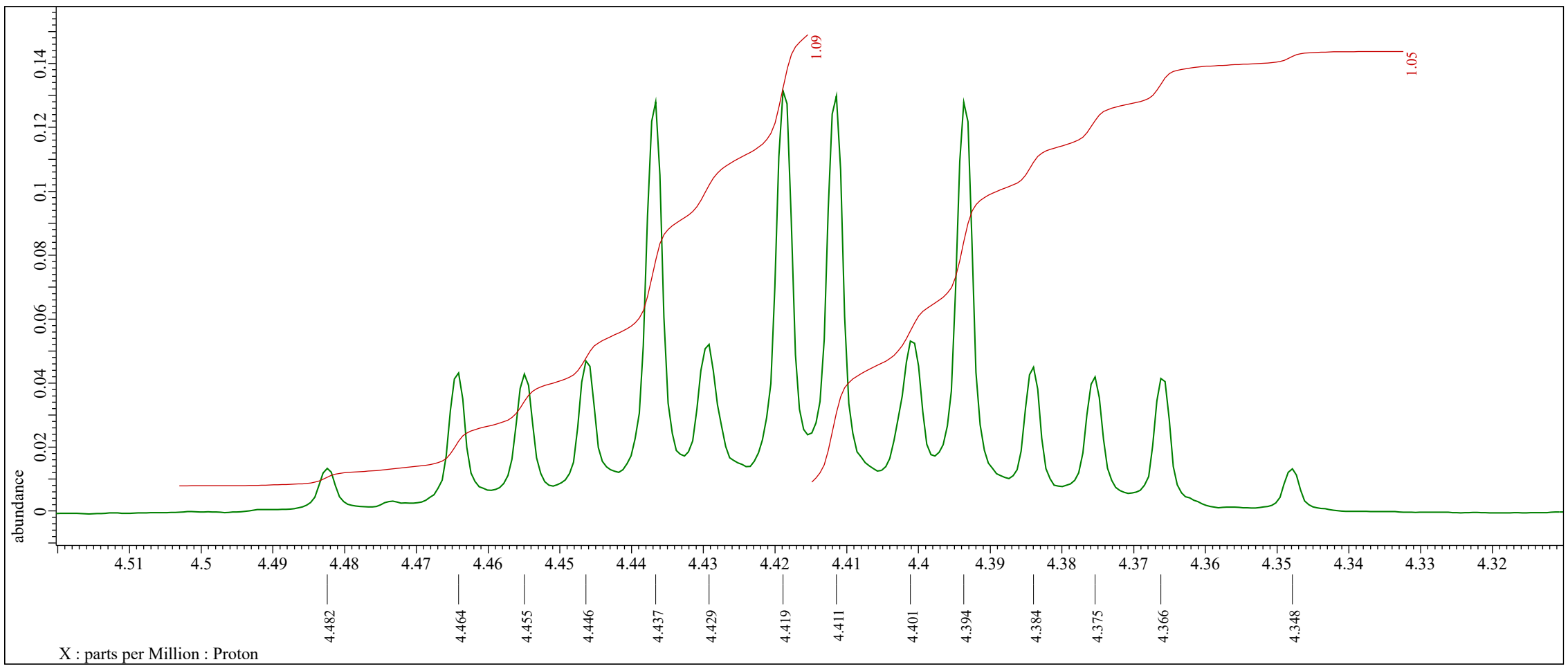

$^{13}C\{^1H\}$ **NMR** (100 MHz, $CDCl_3$)

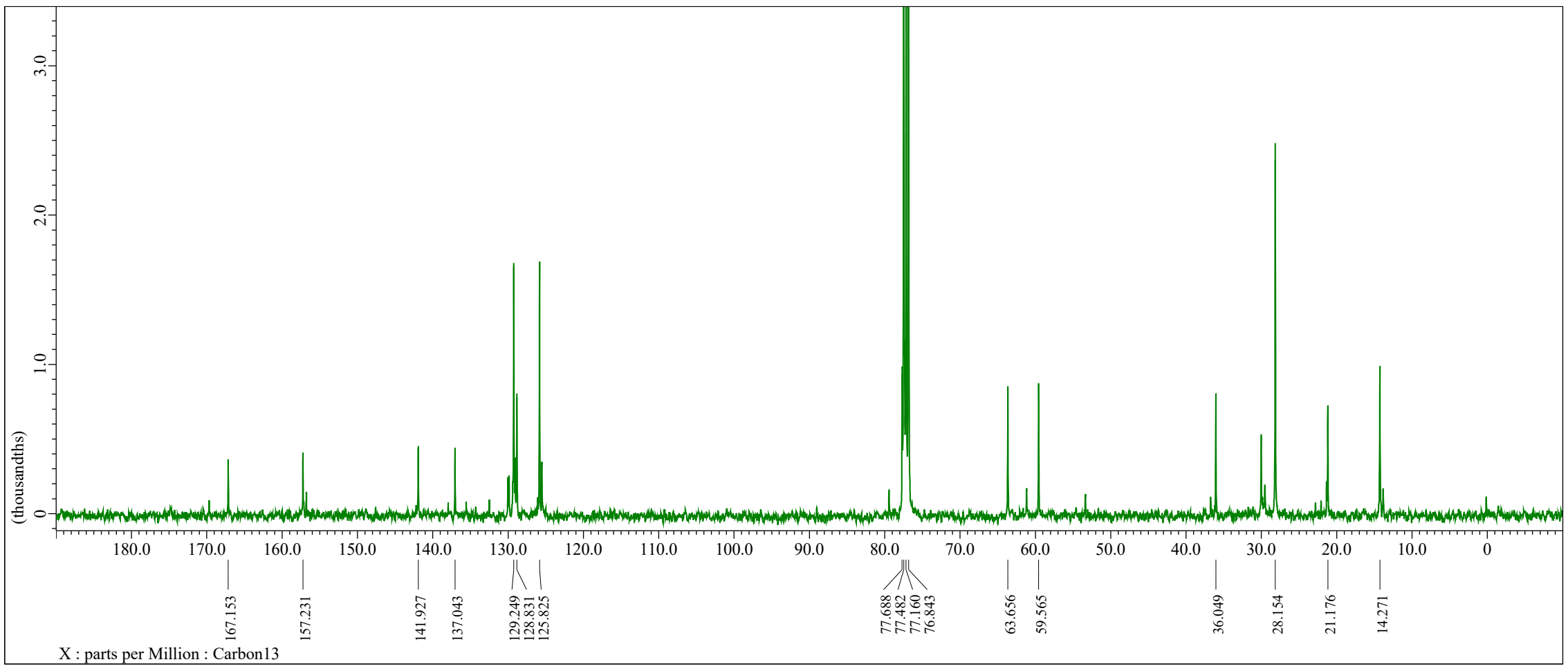

Ethyl (*Z*)-2-{(4*R,5*S**)-4-(*tert*-butyl)-2,2-dichloro-5-(naphthalen-2-yl)-1,2-oxagermolan-3-ylidene)acetate** *anti*-*γ*-**4ag**

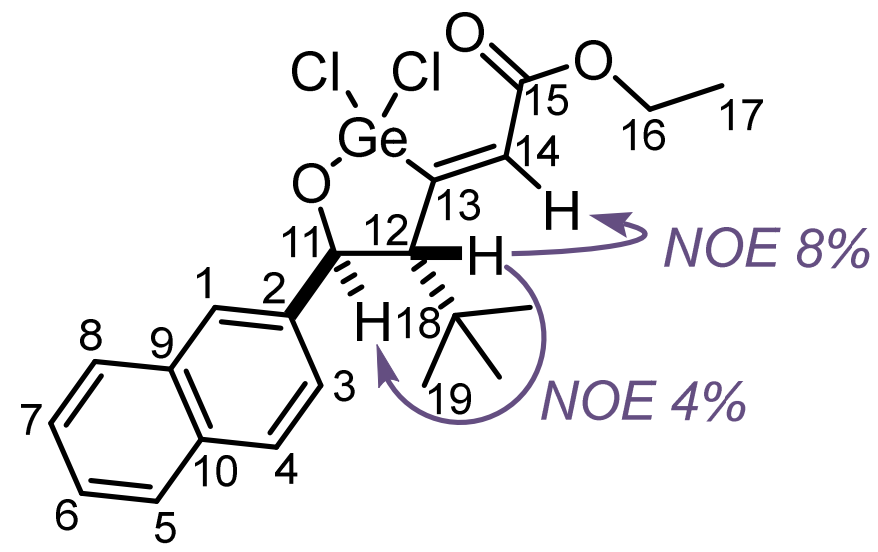

To a mixture of **1a** (0.0339 g, 0.202 mmol) and 2-naphthaldehyde **3g** (0.0312 g, 0.200 mmol) in hexane (1.0 mL) was added $GeCl_2$-dioxane (0.0231 g, 0.100 mmol). After the reaction mixture was stirred at room temperature for 18 h, the solvent was removed under vacuum. The yield was determined by ^{1}H NMR of the obtained crude product using 1,1,2,2-tetrachloroethane as an internal standard (89%, *anti*-*α*-**4ag**/*anti*-*γ*-**4ag** = 18/82, single diastereomer).
To obtain the spectrodata of the isolated product, some conditions were modified. To a mixture of **1a** (1.1 eq) and 2-naphthaldehyde **3g** (0.090 eq) in hexane (1.0 mL) was added $GeCl_2$-dioxane (1.0 eq). After the reaction mixture was stirred at room temperature for 18 h, the solvent was removed under vacuum. Careful extraction of the crude mixture with hexane gave *anti*-*γ*-**4ag** in pure form as a colorless solid.
mp 41.0–42.0 °C; **IR** (KBr) 3057 (w), 2964 (m), 1685 (m), 1373 (m), 1327 (s), 1259 (m), 1214 (m), 1023 (m), 856 (m) cm^{-1}; ^{1}**H NMR** (400 MHz, $CDCl_3$) 7.92 (s, 1H, 1-H), 7.86–7.78 (m, 3H), 7.49–7.42 (m, 2H), 7.35 (dd, J = 8.4, 1.6 Hz, 1H, 3-H), 6.61 (d, J = 1.6 Hz, 1H, 14-H), 5.76 (s, 1H, 11-H), 4.43 (dq, J = 11.0, 7.0 Hz, 1H, 16-H), 4.38 (dq, J = 11.0, 7.4 Hz, 1H, 16-H), 2.94 (s, 1H, 12-H), 1.36 (t, J = 7.2 Hz, 3H, 17-H), 1.15 (s, 9H, 19-H); $^{13}C\{^1H\}$ **NMR** (100 MHz, $CDCl_3$) 167.2 (s, C-15), 157.0 (s, C-13), 142.1 (s, C-2), 133.2 (s), 132.6 (s), 129.0 (d, C-14), 128.4 (d), 128.3 (d), 127.6 (d), 126.4 (d), 126.1 (d), 124.7 (d, C-1), 123.8 (d, C-3), 77.9 (d, C-11), 63.7 (t, C-16), 59.6 (d, C-12), 36.1 (s, C-18), 28.2 (q, C-19), 14.2 (q, C-17); **HRMS** (DART$^+$) Calculated ($C_{21}H_{25}O_3Cl_2Ge$): 469.0387 ($[M+H]^+$), Found: 469.0392 ($[M+H]^+$).

^{1}H NMR (400 MHz, $CDCl_3$)

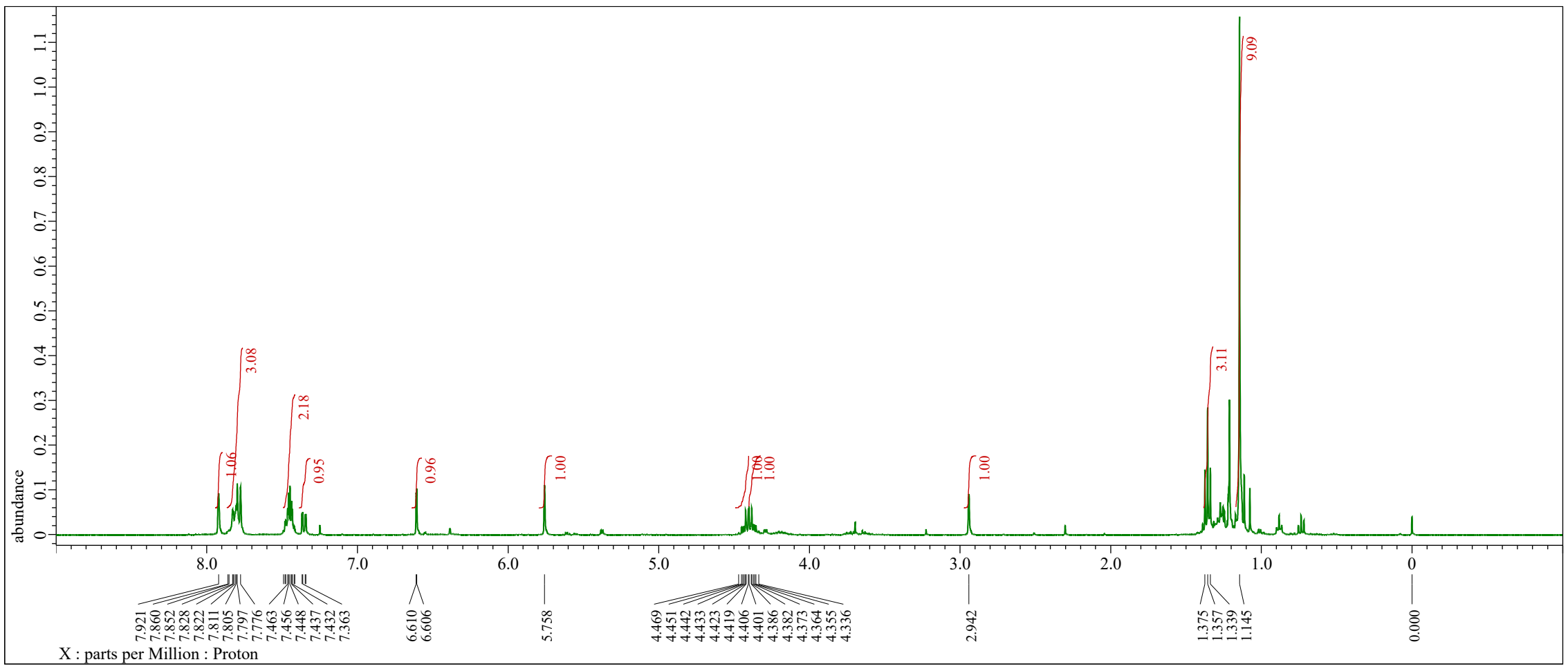

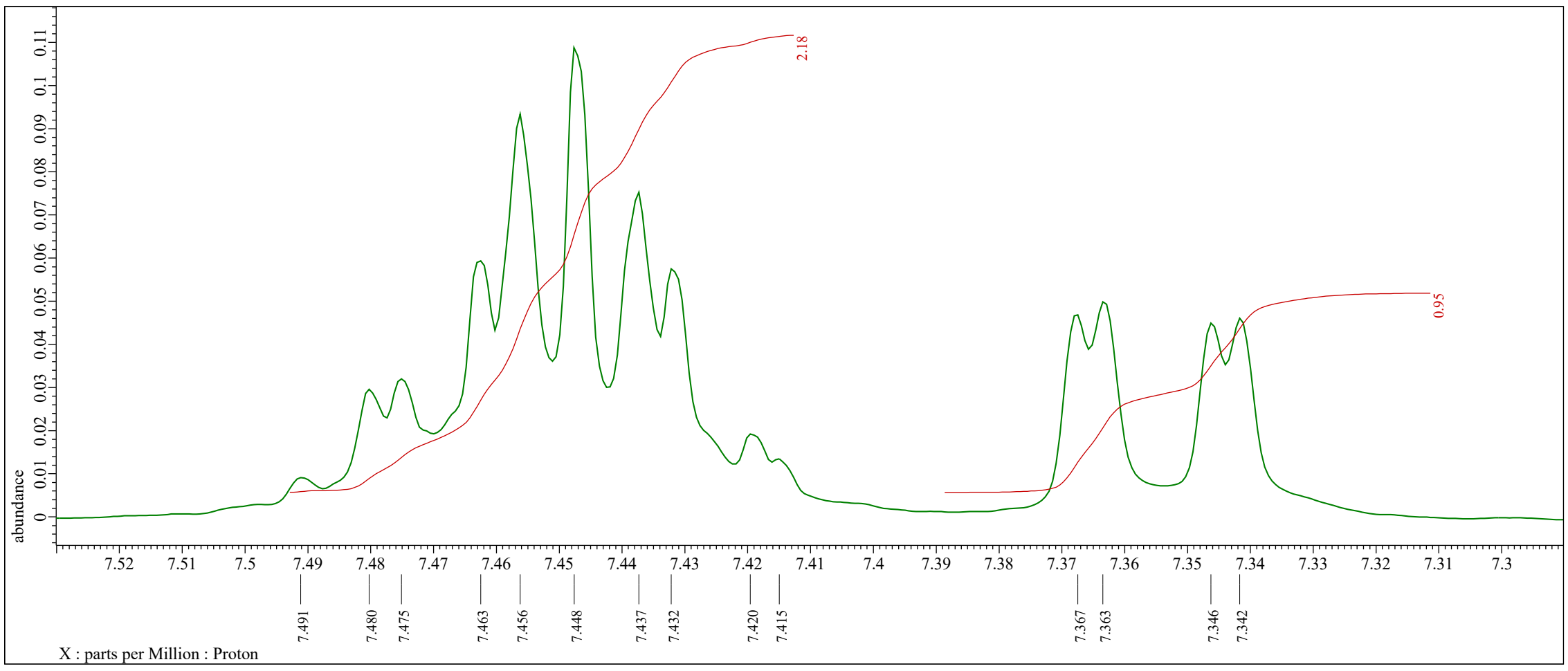

^{13}C{^{1}H} NMR (100 MHz, $CDCl_3$)

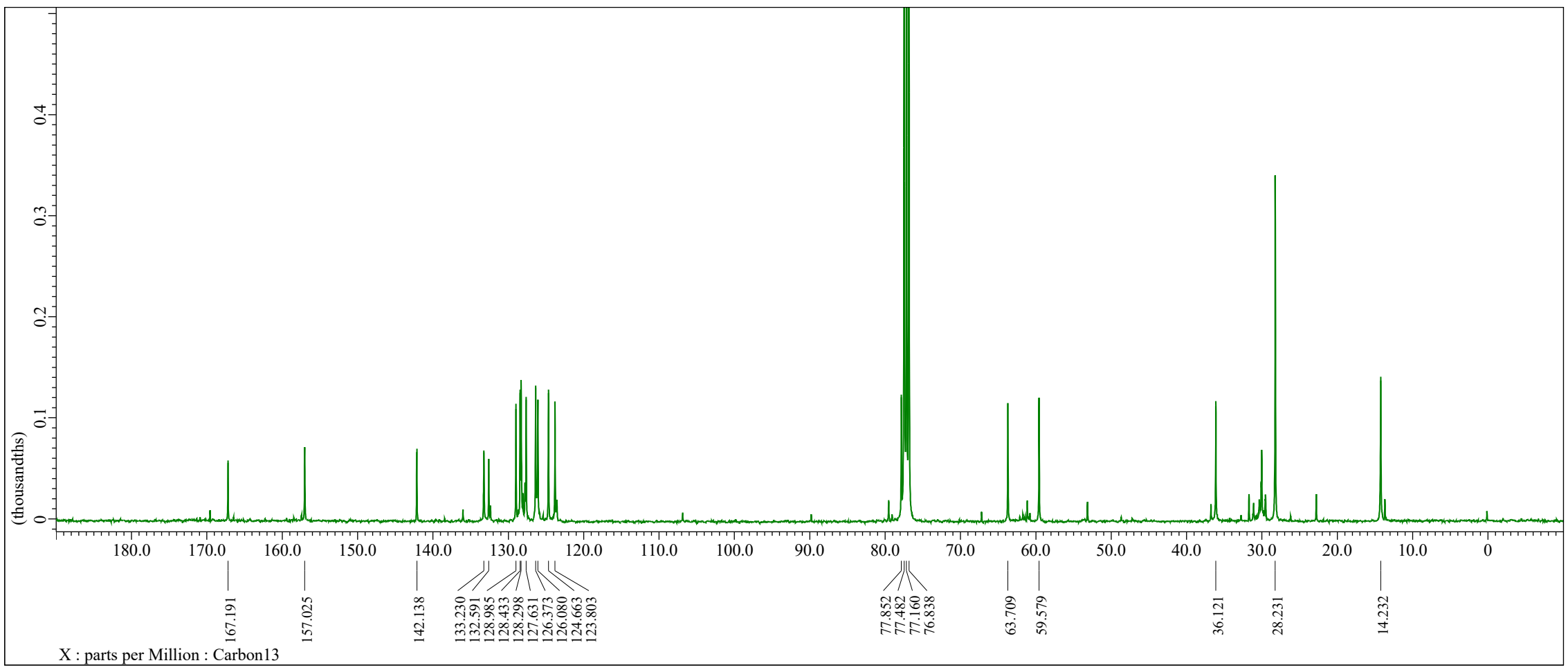

Ethyl (*Z*)-2-{(4*R,5*S**)-4-(*tert*-butyl)-2,2-dichloro-5-(naphthalen-1-yl)-1,2-oxagermolan-3-ylidene}acetate *anti*-γ-4ah**

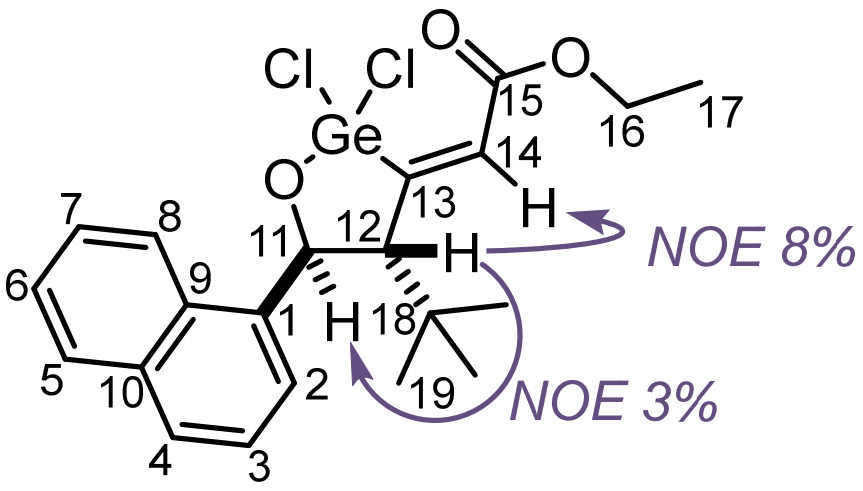

To a mixture of **1a** (0.0334 g, 0.199 mmol) and 1-naphthaldehyde **3h** (0.0272 mL, 0.200 mmol) in hexane (1.0 mL) was added $GeCl_2$-dioxane (0.0233 g, 0.101 mmol). After the reaction mixture was stirred at room temperature for 18 h, the solvent was removed under vacuum. The yield was determined by ^{1}H NMR of the obtained crude product using 1,1,2,2-tetrachloroethane as an internal standard (99%, *anti*-α-**4ah**/*anti*-γ-**4ah** = 15/85, single diastereomer). To obtain the spectrodata of the isolated product, some conditions were modified. To a mixture of **1a** (1.1 eq) and 1-naphthaldehyde **3h** (0.095 eq) in hexane (1.0 mL) was added $GeCl_2$-dioxane (1.0 eq). After the reaction mixture was stirred at room temperature for 18 h, the solvent was removed under vacuum. Careful extraction of the crude mixture with hexane gave *anti*-γ-**4ah** in pure form as colorless neat.

IR (KBr) 3049 (w), 2963 (m), 1685 (m), 1328 (s), 1255 (m), 1215 (m), 1097 (m), 995 (m) cm^{-1}; **^{1}H NMR** (400 MHz, $CDCl_3$) 8.47 (d, J = 8.4 Hz, 1H, 8-H), 7.85 (d, J = 8.4 Hz, 1H, 5-H), 7.77 (d, J = 8.4 Hz, 1H, 4-H), 7.60 (t, J = 7.2 Hz, 1H, 7-H), 7.52 (t, J = 7.2 Hz, 1H, 6-H), 7.34 (t, J = 7.6 Hz, 1H, 3-H), 7.14 (d, J = 7.2 Hz, 1H, 2-H), 6.85 (d, J = 1.2 Hz, 1H, 14-H), 6.31 (s, 1H, 11-H), 4.50–4.42 (m, 2H, 16-H), 3.29 (s, 1H, 12-H), 1.42 (t, J = 7.2 Hz, 3H, 17-H), 1.14 (s, 9H, 19-H); **^{13}C{^{1}H} NMR** (100 MHz, $CDCl_3$) 167.3 (s, C-15), 159.5 (s, C-13), 139.2 (s, C-1), 134.2 (s, C-10), 132.1 (s, C-9), 129.0 (d, C-4), 128.8 (d, C-5), 128.0 (d, C-14), 126.7 (d, C-7), 126.1 (d, C-6), 124.9 (d, C-3), 124.5 (d, C-8), 123.3 (d, C-2), 73.3 (d, C-11), 63.7 (t, C-16), 56.0 (d, C-12), 36.3 (s, C-18), 28.4 (q, C-19), 14.3 (q, C-17); **HRMS** (DART$^+$) Calculated ($C_{21}H_{25}O_3Cl_2Ge$): 469.0387 ($[M+H]^+$), Found: 469.0388 ($[M+H]^+$).

^{1}H NMR (400 MHz, $CDCl_3$)

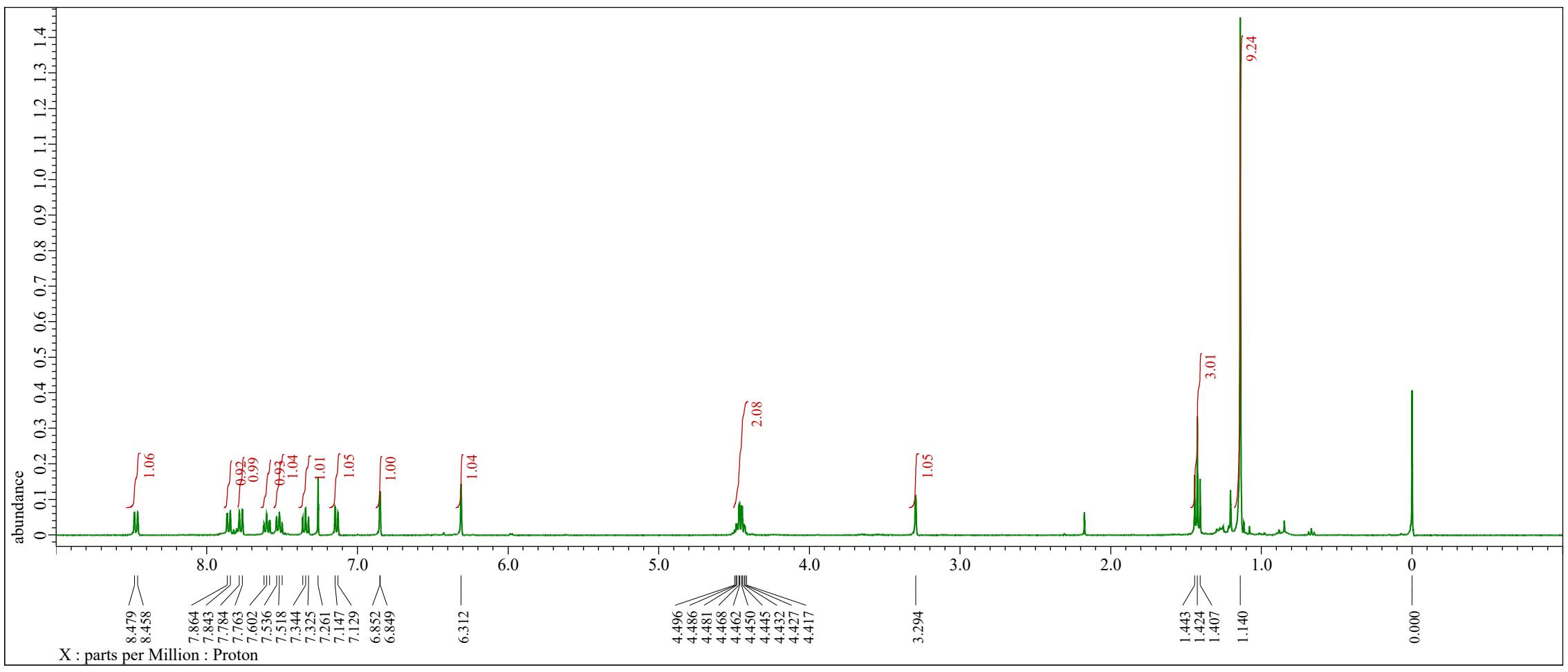

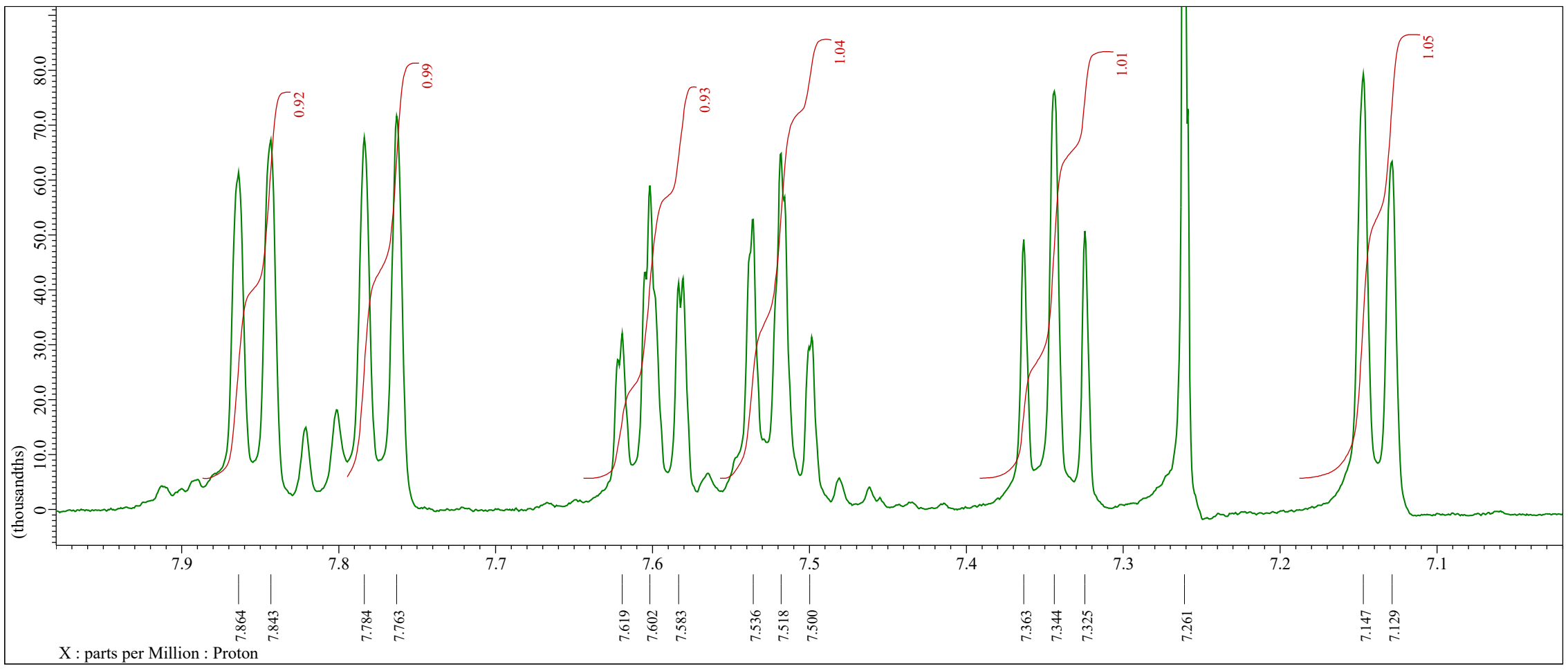

$^{13}C\{^{1}H\}$ NMR (100 MHz, $CDCl_3$)

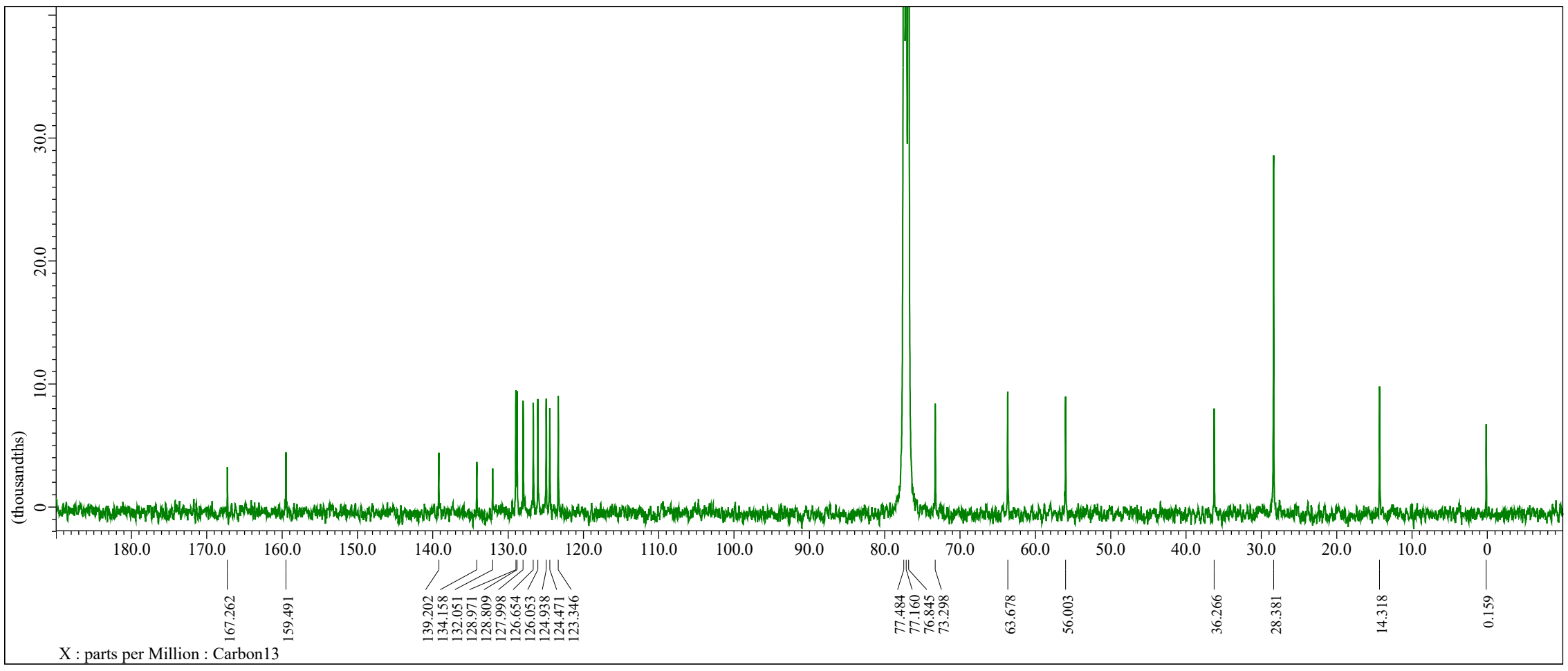

Ethyl (*Z*)-2-{(4*R,5*S**)-4-(*tert*-butyl)-2,2-dichloro-5-(thiophen-2-yl)-1,2-oxagermolan-3-ylidene}acetate** *anti*-*γ*-**4ai**

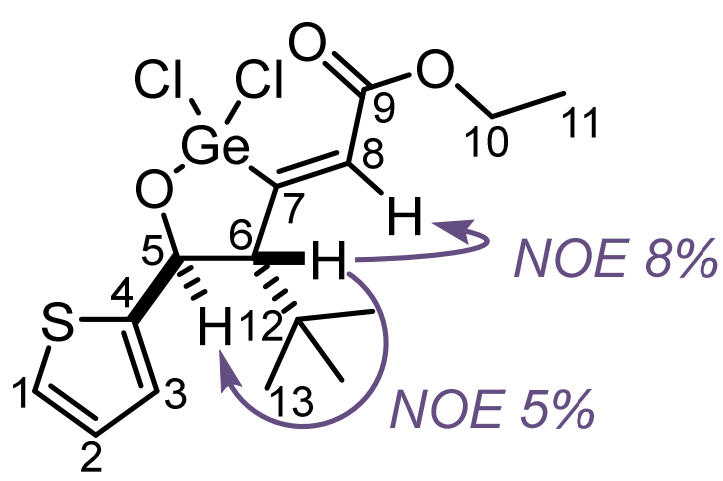

To a mixture of **1a** (0.0339 g, 0.149 mmol) and thiophene-2-carbaldehyde **3i** (0.0182 mL, 0.202 mmol) in hexane (1.0 mL) was added $GeCl_2$-dioxane (0.0233 g, 0.101 mmol). After the reaction mixture was stirred at room temperature for 18 h, the solvent was removed under vacuum. The yield was determined by ^{1}H NMR of the crude product using 1,1,2,2-tetrachloroethane as an internal standard (91%, *anti*-*α*-**4ai**/*anti*-*γ*-**4ai** = 18/82, single diastereomer).

To obtain the spectrodata of the isolated product, some conditions were modified. To a mixture of **1a** (1.1 eq) and

thiophene-2-carbaldehyde **3i** (2.0 eq) in hexane (1.0 mL) was added $GeCl_2$-dioxane (1.0 eq). After the reaction mixture was stirred at room temperature for 16 h, the solvent was removed under vacuum. Careful extraction of the crude mixture with hexane gave *anti*-*γ*-**4ai** in pure form as colorless neat.

IR (KBr) 3110 (w), 2966 (m), 1685 (m), 1374 (m), 1328 (s), 1259 (m), 1215 (m), 1026 (m), 910 (m) cm^{-1}; **^{1}H NMR** (400 MHz, $CDCl_3$) 7.22 (dd, *J* = 4.8, 1.2 Hz, 1H, 1-H), 6.96 (dt, *J* = 3.6, 1.1 Hz, 1H, 3-H), 6.92 (dd, *J* = 5.0, 3.4 Hz, 1H, 2-H), 6.69 (d, *J* = 1.6 Hz, 1H, 8-H), 5.77 (s, 1H, 5-H), 4.44 (dq, *J* = 11.0, 7.4 Hz, 1H, 10-H), 4.40 (dq, *J* = 11.0, 7.0 Hz, 1H, 10-H), 2.99 (dd, *J* = 1.6, 0.8 Hz, 1H, 6-H), 1.38 (t, *J* = 7.2 Hz, 3H, 11-H), 1.09 (s, 9H, 13-H); **^{13}C{^{1}H} NMR** (100 MHz, $CDCl_3$) 167.2 (s, C-9), 157.1 (s, C-7), 149.1 (s, C-4), 129.0 (d, C-8), 126.8 (d, C-2), 125.1 (d, C-1), 124.1 (d, C-3), 73.9 (d, C-5), 63.7 (t, C-10), 59.7 (d, C-6), 35.9 (s, C-12), 28.0 (q, C-13), 14.2 (q, C-11); **HRMS** ($DART^+$) Calculated ($C_{15}H_{21}O_3SCl_2Ge$): 424.9795 ($[M+H]^+$), Found: 424.9787 ($[M+H]^+$).

^{1}H NMR (400 MHz, $CDCl_3$)

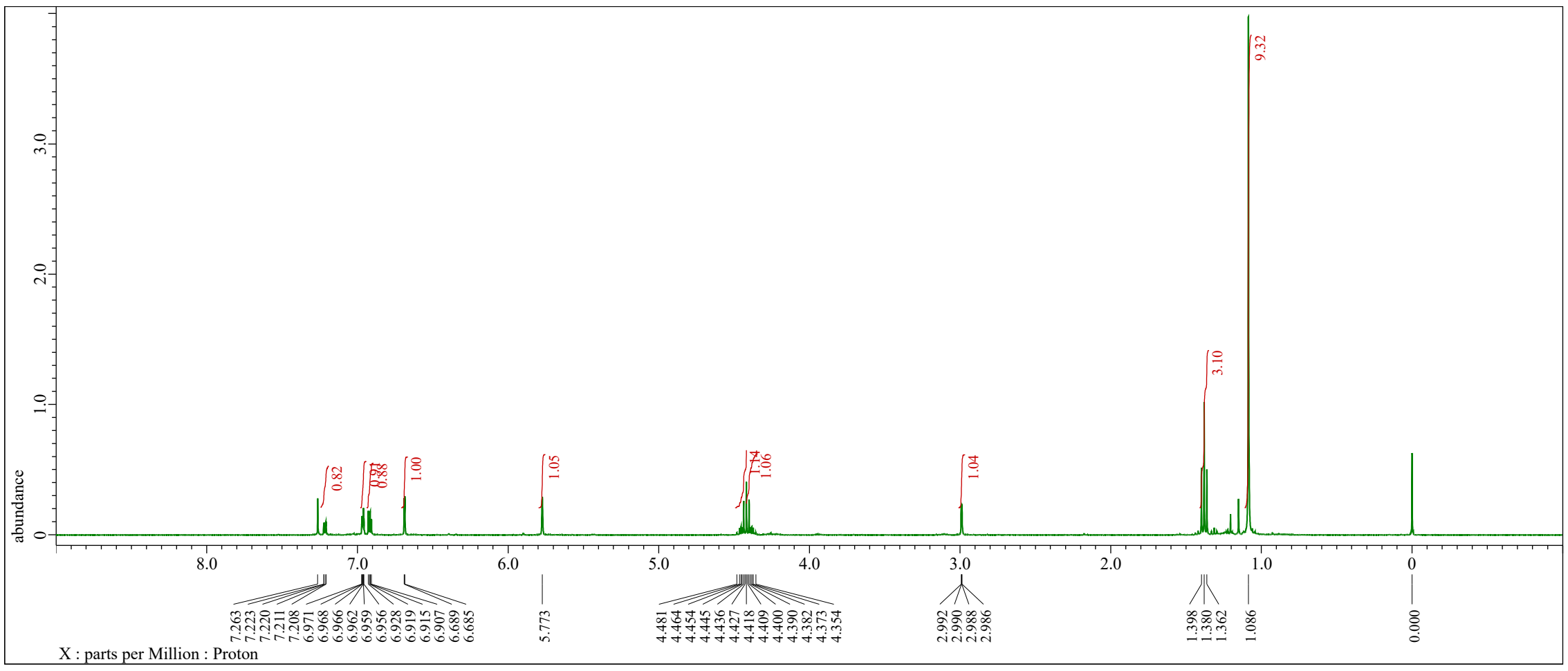

^{13}C{^{1}H} NMR (100 MHz, $CDCl_3$)

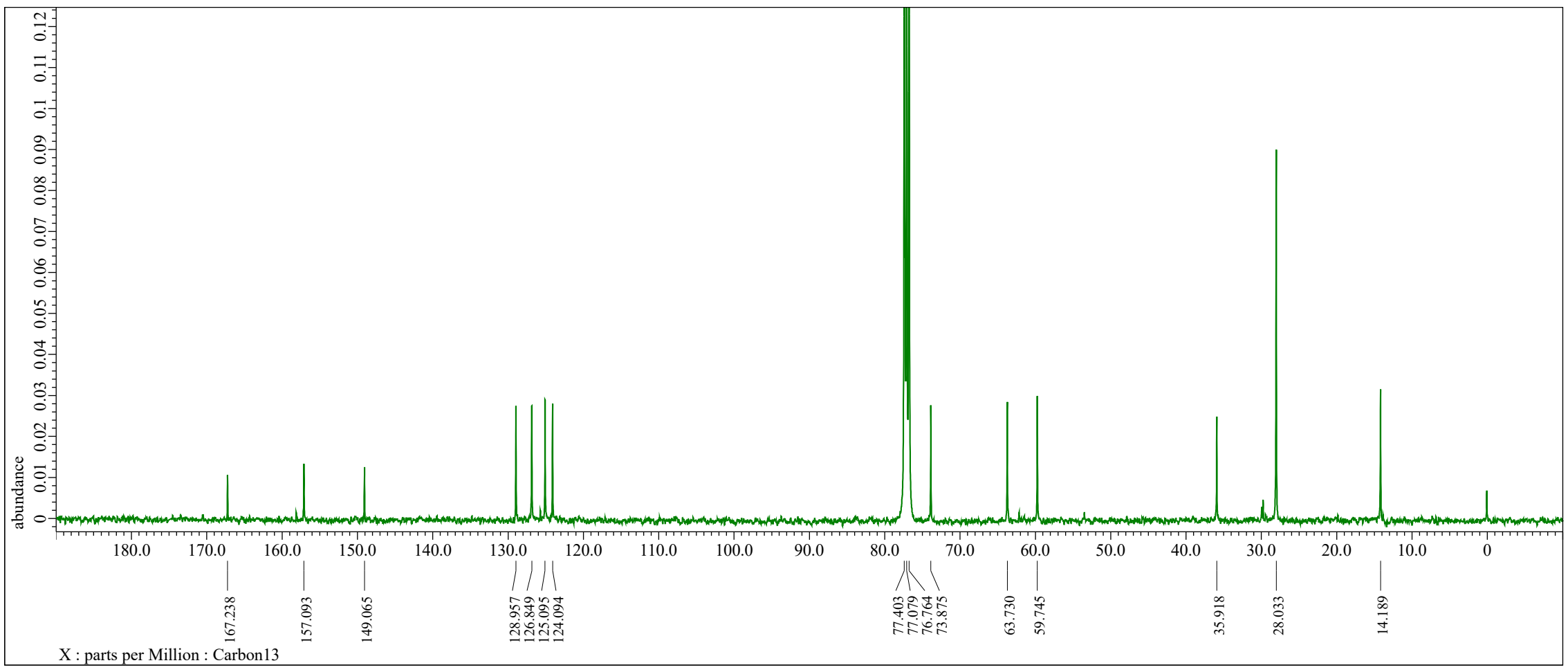

Ethyl (*Z*)-2-[(4*R,5*R**)-4-(*tert*-butyl)-2,2-dichloro-5-{(*E*)-styryl}-1,2-oxagermolan-3-ylidene]acetate** *anti*-*γ*-**4aj**

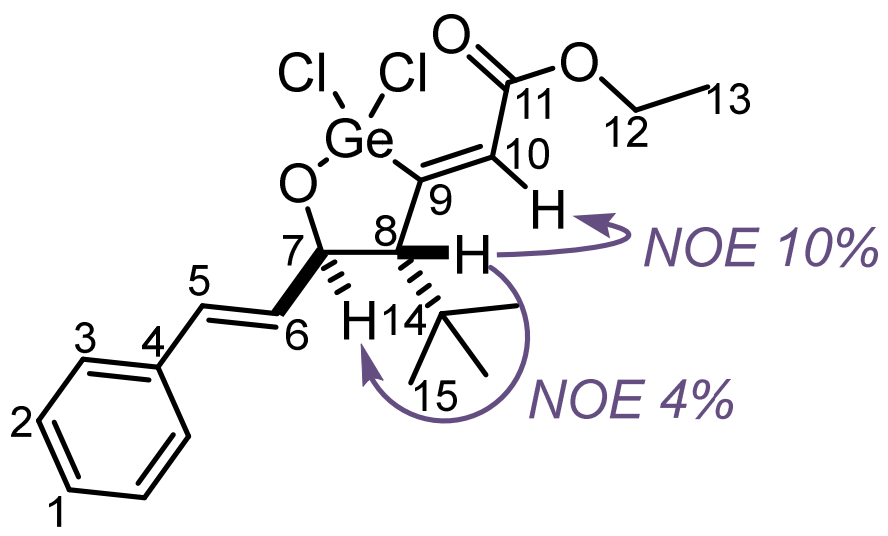

Conditions B

To a mixture of **1a** (0.0253 g, 0.200 mmol) and cinnamaldehyde **3j** (0.0252 mL, 0.200 mmol) in EtOAc (1.0 mL) was added $GeCl_2$-dioxane (0.0232 g, 0.100 mmol). After the reaction mixture was stirred at room temperature for 18 h, the solvent was removed under vacuum. The yield was determined by ^{1}H NMR of the crude product using 1,1,2,2-tetrachloroethane as an internal standard (19%, single isomer).

Conditions C

To a mixture of **1a** (0.0338 g, 0.201 mmol) and cinnamaldehyde **3j** (0.0252 mL, 0.200 mmol) in hexane (1.0 mL) was added $GeCl_2$-dioxane (0.0232 g, 0.100 mmol). After the reaction mixture was stirred at room temperature for 18 h, the solvent was removed under vacuum. The yield was determined by ^{1}H NMR of the crude product using 1,1,2,2-tetrachloroethane as an internal standard (27%, single isomer).

To obtain the spectrodata of the isolated product, some conditions were modified. To a mixture of **1a** (1.0 eq) and cinnamaldehyde **3j** (2.0 eq) in hexane (1.0 mL) was added $GeCl_2$-dioxane (1.0 eq). After the reaction mixture was stirred at room temperature for 18 h, the solvent was removed under vacuum, then the remaining cinnamaldehyde **3j** was removed in vacuo (0.021 Torr, 120 °C, 1 h). Careful extraction of the crude mixture with hexane gave *anti*-*γ*-**4aj**. The product was obtained as a mixture with residual cinnamaldehyde **3j**, which hampered recording IR and NMR spectra in the pure form.

^{1}H NMR (400 MHz, $CDCl_3$) 7.36–7.27 (m, 4H), 7.24–7.20 (m, 1H, 1-H), 6.67 (dd, *J* = 15.8, 1.4 Hz, 1H, 5-H), 6.66 (d, *J* = 1.6 Hz, 1H, 10-H), 6.04 (dd, *J* = 15.8, 5.8 Hz, 1H, 6-H), 5.20 (d, *J* = 5.6 Hz, 1H, 7-H), 4.45 (dq, *J* = 11.2, 7.2 Hz, 1H, 12-H), 4.40 (dq, *J* = 11.0, 7.0 Hz, 1H, 12-H), 2.63 (dd, *J* = 1.6, 0.8 Hz, 1H, 8-H), 1.38 (t, *J* = 7.2 Hz, 3H, 13-H), 1.05 (s, 9H, 15-H); **^{13}C{^{1}H} NMR** (100 MHz, $CDCl_3$) 167.2 (s, C-11), 157.4 (s, C-9), 136.6 (s, C-4), 132.2 (d, C-6), 130.3 (d, C-5), 129.2 (d, C-10), 128.8 (d, C-2), 127.8 (d, C-1), 126.7 (d, C-3), 76.5 (d, C-7), 63.7 (t, C-12), 58.3 (d, C-8), 35.8 (s, C-14), 28.1 (q, C-15), 14.3 (q, C-13); **HRMS** (DART$^+$) Calculated ($C_{19}H_{25}O_3Cl_2Ge$): 445.0387 ([M+H]$^+$), Found: 445.0380 ([M+H]$^+$).

^{1}H NMR (400 MHz, $CDCl_3$)

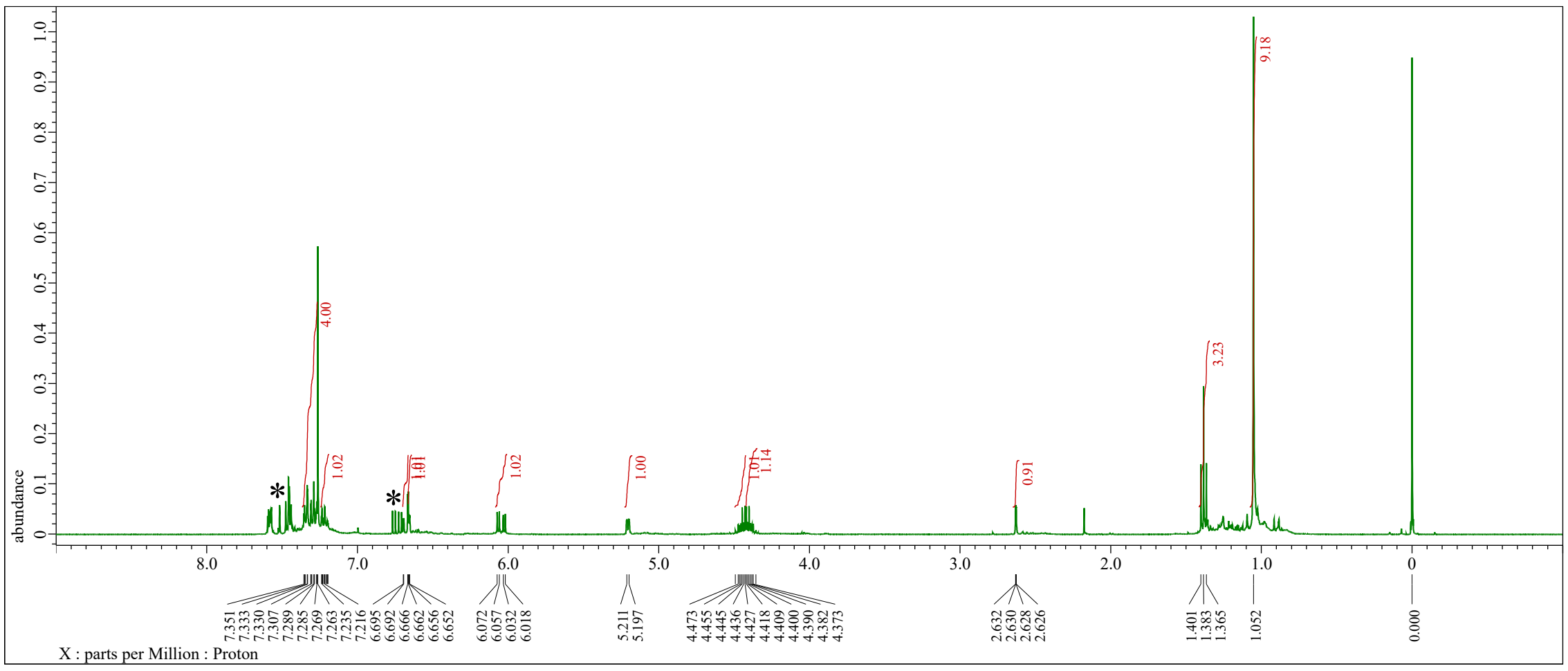

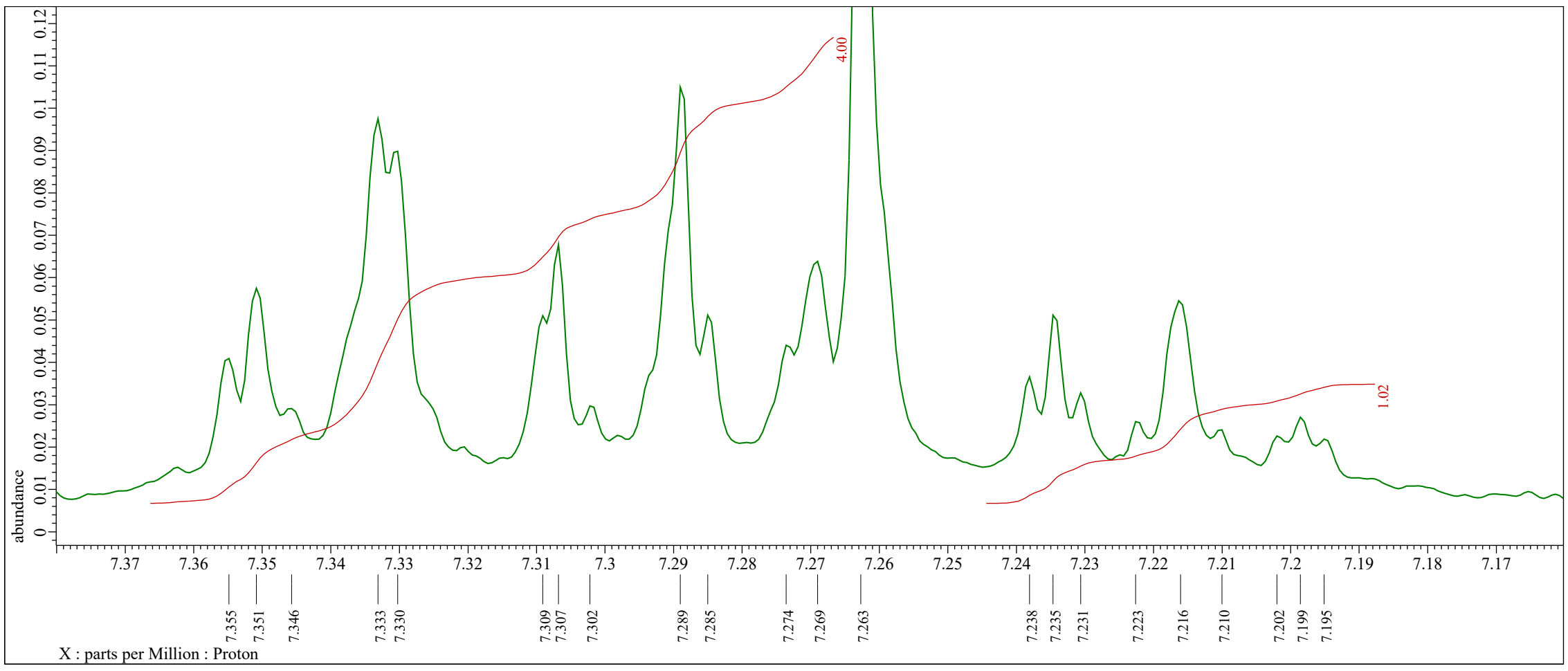

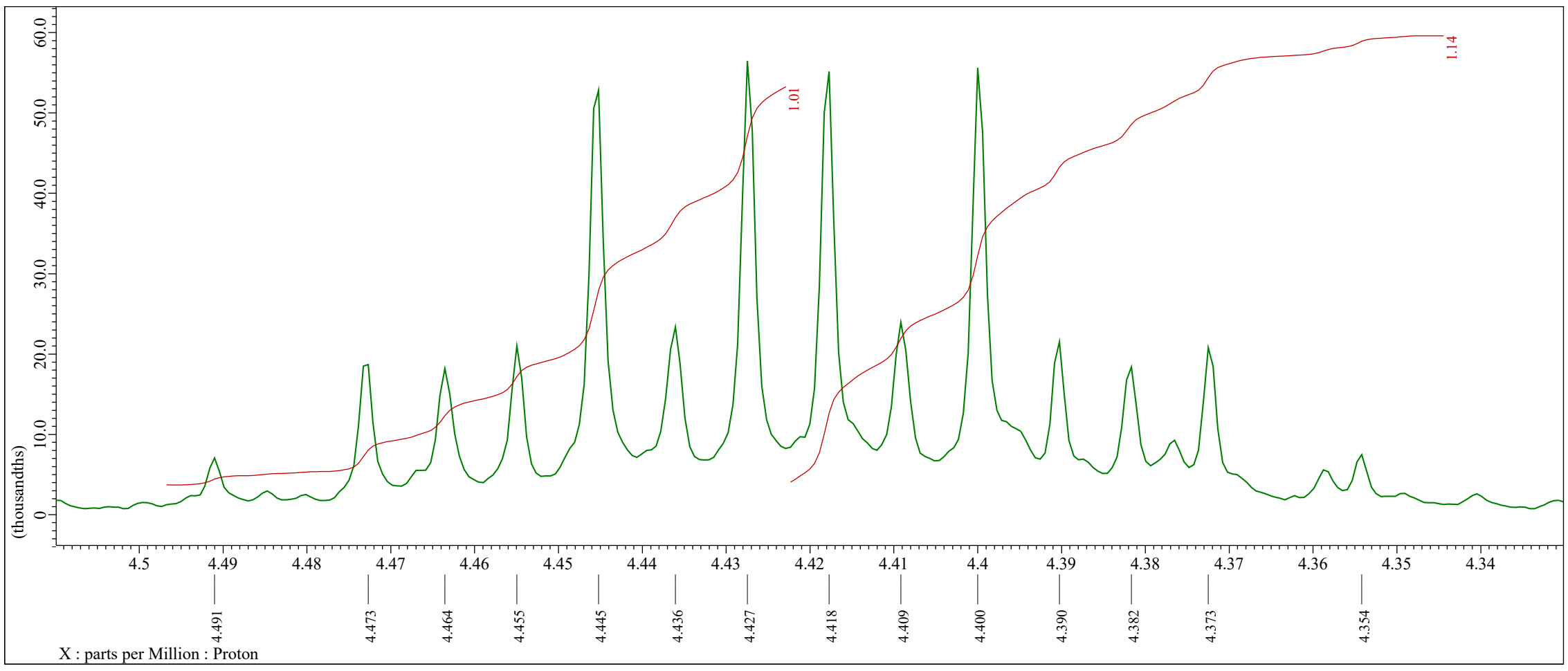

$^{13}C\{^1H\}$ **NMR** (100 MHz, $CDCl_3$)

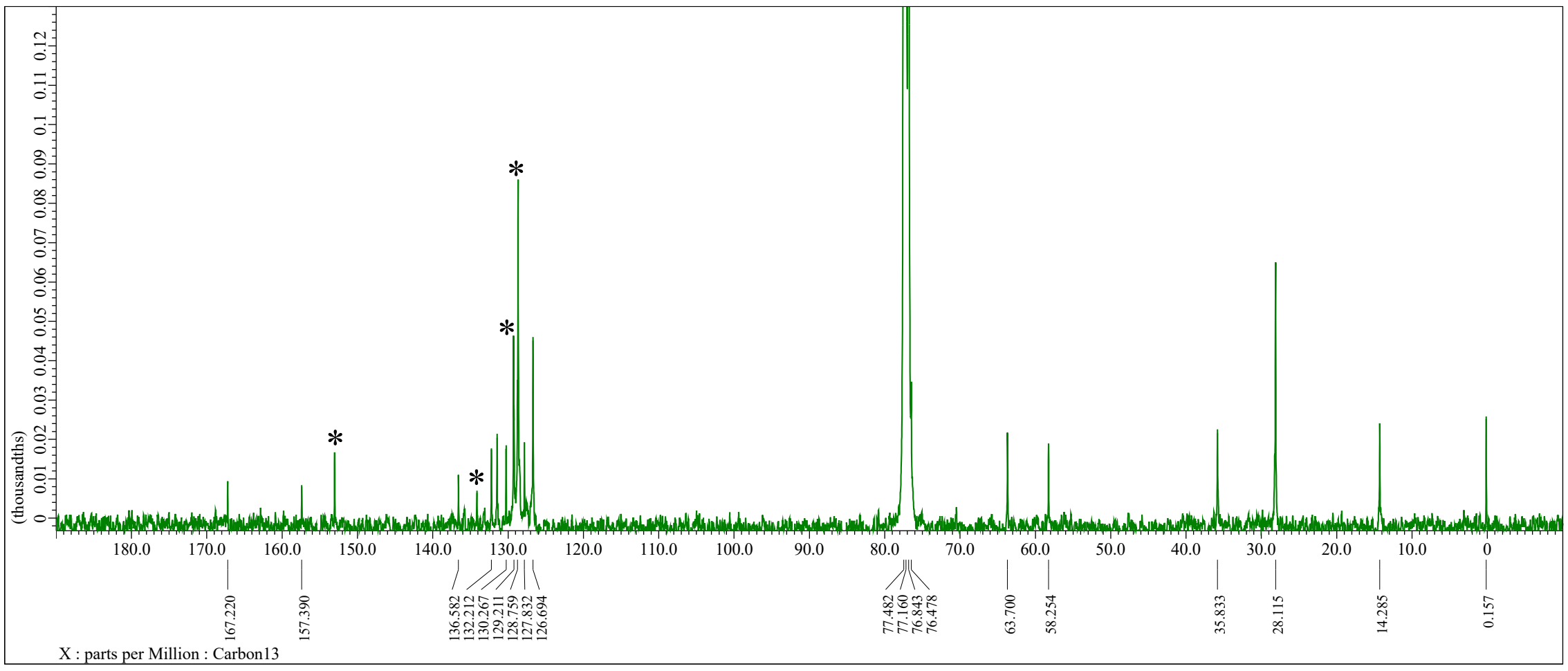

Asterisk marks (*) represent the signals of the inseparatable cinnamaldehyde **3j**.

3-5. *syn-α*-Selective aldol reaction of allenoate 1 with benzaldehyde 3a using $GeCl_2$-dioxane

Dibenzyl (3*E*,4*R,5*R**,8*E*,9*S**,10*S**)-2,2,7,7-tetrachloro-3,8-bis(2,2-dimethylpropylidene)-5,10-diphenyl-1,6,2,7-dioxadigermecane-4,9-dicarboxylate *syn-α*-4ca**

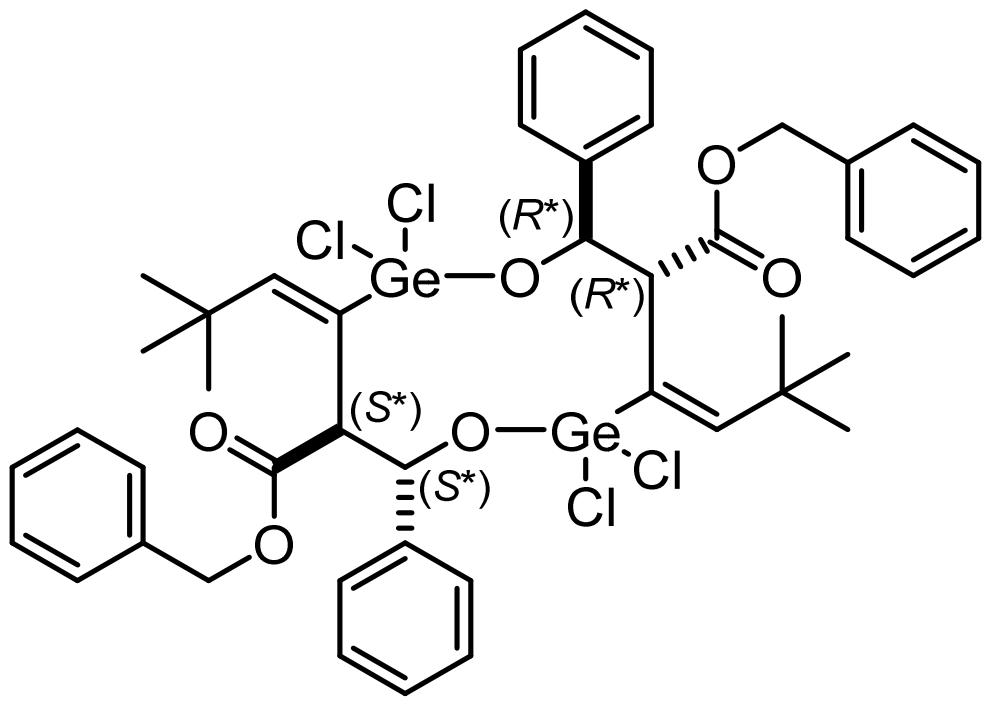

To a mixture of **1c** (0.0344 g, 0.149 mmol), benzaldehyde **3a** (0.0152 mL, 0.150 mmol), and BF_3·OEt_2 (0.0252 mL, 0.100 mmol) in CH_3CN (1.0 mL) was added $GeCl_2$-dioxane (0.0232 g, 0.100 mmol). After the reaction mixture was left to stand at room temperature for 18 h, the obtained colorless solid was filtrated and washed with CH_3CN, to give the *syn-α*-**4ca** as the enantiomeric dimer (0.0287 g, 60%). The filtrate was evaporated under vaccum, to give the mixture of *anti-α*-**4ca** and *anti-γ*-**4ca** (1 and 1%). The yields and ratio was determined by ^{1}H NMR using 1,1,2,2-tetrachloroethane as an internal standard. The total yield of **4** and the ratio of three isomers are 62% and *syn-α*-**4ca**/*anti-α*-**4ca**/*anti-γ*-**4ca** = 97/2/1. The *syn-α*-**4ca** is promptly decomposed at the solution in any solvents, which hampered recording any NMR spectrum.

mp 166.3–167.2 °C; **IR** (ATR) 3033 (w), 2961 (m), 1695 (s), 1454 (w), 1331 (m), 1262 (m), 1188 (m), 1081 (m), 1048 (s) cm^{-1}; **Analysis** $C_{44}H_{48}Cl_4Ge_2O_6$ (959.92) Calculated: C, 55.05; H, 5.04, Found: C, 54.80; H, 5.10.

Ethyl 2,2-dichloro-5-phenyl-3-(propan-2-ylidene)-1,2-oxagermolane-4-carboxylate *α*-4ga

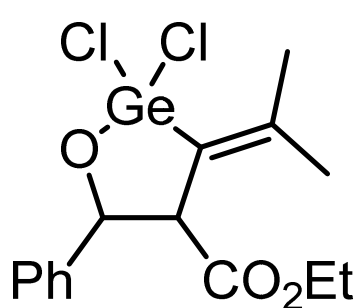

α-**4ga**

Ethyl (*Z*)-2-(2,2-dichloro-4,4-dimethyl-5-phenyl-1,2-oxagermolan-3-ylidene)acetate *γ*-4ga

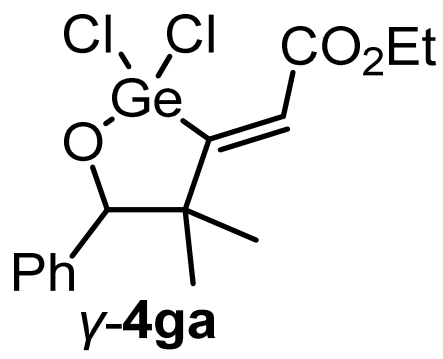

γ-**4ga**

Conditions A

To a mixture of **1g** (0.0209 g, 0.149 mmol), benzaldehyde **3a** (0.0152 mL, 0.150 mmol), and BF_3·OEt_2 (0.0252 mL, 0.100 mmol) in CH_3CN (1.0 mL) was added $GeCl_2$-dioxane (0.0232 g, 0.100 mmol). After the reaction mixture was left to stand at room temperature for 18 h, the solvent was removed under vacuum. The yield was determined by ^{1}H NMR of the crude product using 1,1,2,2-tetrachloroethane as an internal standard (81%, *syn-α*-**4ga**/*anti-α*-**4ga** = 61/39).

Conditions B

To a mixture of **1g** (0.0208 g, 0.148 mmol) and benzaldehyde **3a** (0.0202 mL, 0.200 mmol) in EtOAc (1.0 mL) was added $GeCl_2$-dioxane (0.0230 g, 0.0993 mmol). After the reaction mixture was stirred at room temperature for 18 h, the solvent was removed under vacuum. The yield was determined by ^1H NMR of the crude product using 1,1,2,2-tetrachloroethane as an internal standard (quant., *syn*-*α*-**4ga**/*anti*-*α*-**4ga** = 61/39).

Conditions C

To a mixture of **1g** (0.0276 g, 0.197 mmol) and benzaldehyde **3a** (0.0202 mL, 0.200 mmol) in hexane (1.0 mL) was added $GeCl_2$-dioxane (0.0234 g, 0.101 mmol). After the reaction mixture was stirred at room temperature for 18 h, the solvent was removed under vacuum. The yield was determined by ^1H NMR of the crude product using 1,1,2,2-tetrachloroethane as an internal standard (91%, *syn*-*α*-**4ga**/*anti*-*α*-**4ga**/*γ*-**4ga** = 54/46/trace).

In these conditions, the trace amount of *γ*-**4ga** was observed. However, the difficult separation from the other two isomer hampered recording any isolated data.

To obtain the spectrodata of the isolated *syn*-*α*-**4ga** and *anti*-*α*-**4ga**, some conditions were modified. To a mixture of ethyl 4-methylpenta-2,3-dienoate (1.0 eq) and benzaldehyde (2.0 eq) in EtOAc (1.0 mL) was added $GeCl_2$-dioxane (1.0 eq). After the reaction mixture was stirred at room temperature for 18 h, the solvent was removed under vacuum. Careful extraction of the crude mixture with hexane gave the mixture of *syn*-*α*-**4ga** and *anti*-*α*-**4ga** as colorless neat, which was used for the isolated data of *anti*-*α*-**4ga**. The remaining solid was further washed with hexane and extracted with Et_2O to give *syn*-*α*-**4ga** in pure form as a colorless solid.

Major isomer: Ethyl (4*R,5*R**)-2,2-dichloro-5-phenyl-3-(propan-2-ylidene)-1,2-oxagermolane-4-carboxylate**
syn-*α*-**4ga**

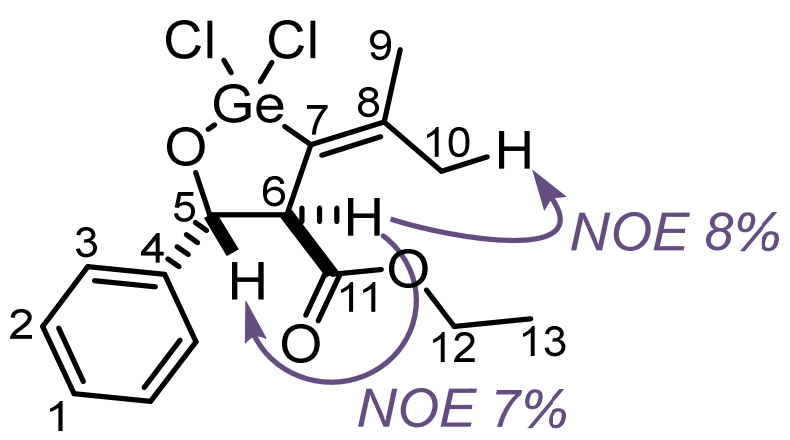

mp 55.3–56.2 °C; **IR** (ATR) 3031 (w), 2983 (w), 1729 (s), 1450 (m), 1370 (m), 1263 (s), 1216 (s), 1024 (s), 875 (m) cm^{-1}; **^{1}H NMR** (400 MHz, $CDCl_3$) 7.40–7.26 (m, 5H), 5.59 (d, *J* = 1.6 Hz, 1H, 5-H), 4.23 (q, *J* = 6.8 Hz, 2H, 12-H), 3.82 (s, 1H, 6-H), 2.12 (s, 3H, 9-H), 1.88 (s, 3H, 10-H), 1.29 (t, *J* = 7.2 Hz, 3H, 13-H); **^{13}C{^{1}H} NMR** (100 MHz, $CDCl_3$) 171.5 (s, C-11), 153.3 (s, C-8), 142.5 (s, C-4), 128.8 (d, C-2), 128.1 (d, C-1), 125.6 (d, C-3), 124.4 (s, C-7), 80.5 (d, C-5), 61.9 (t, C-12), 55.4 (d, C-6), 28.3 (s, C-9), 22.5 (q, C-10), 14.3 (q, C-13); **HRMS** ($DART^+$) Calculated ($C_{15}H_{19}O_3Cl_2Ge$): 390.9918 ($[M+H]^+$), Found: 390.9899 ($[M+H]^+$).

¹H NMR (400 MHz, $CDCl_3$)

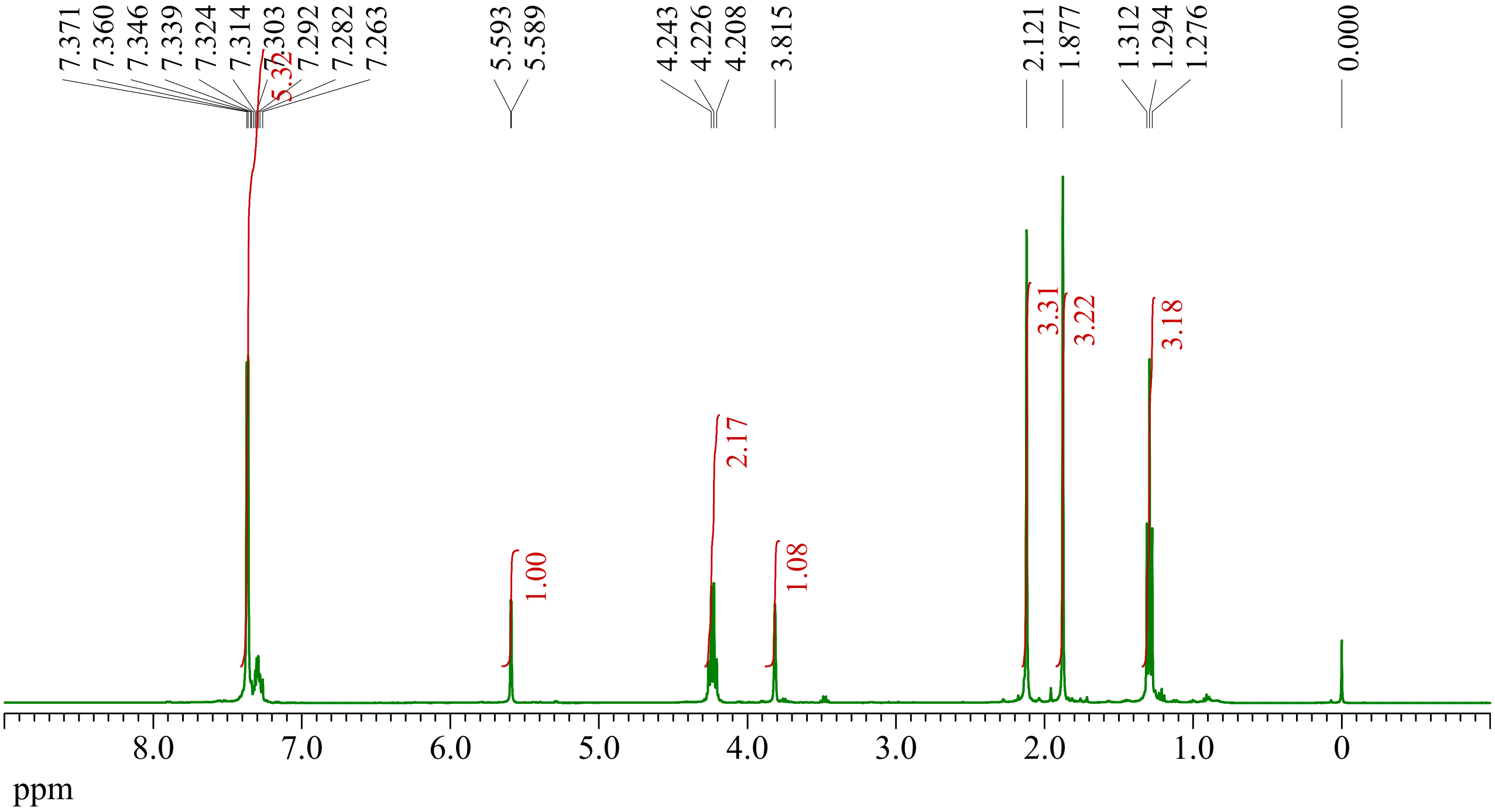

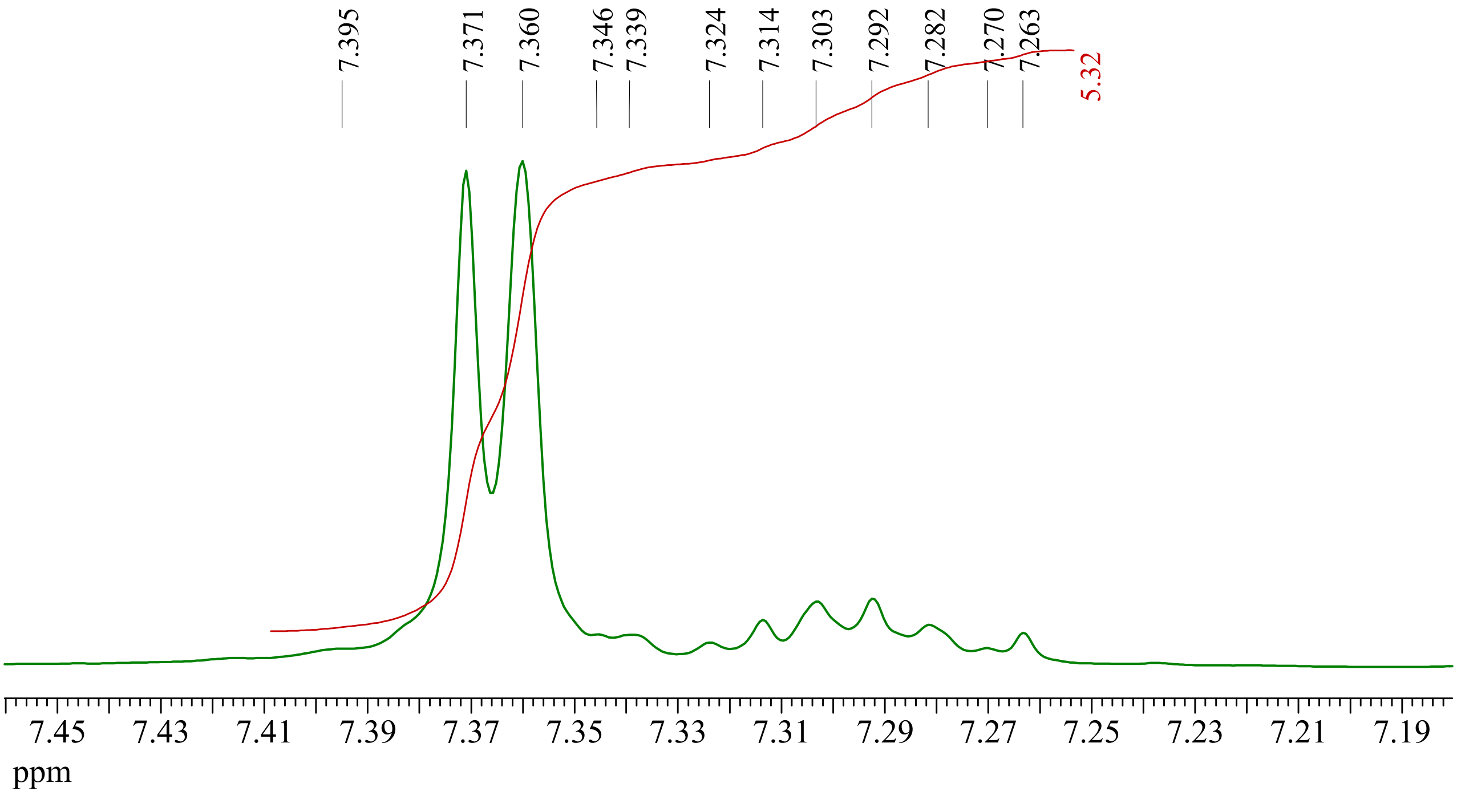

$^{13}C\{^{1}H\}$ NMR (100 MHz, $CDCl_3$)

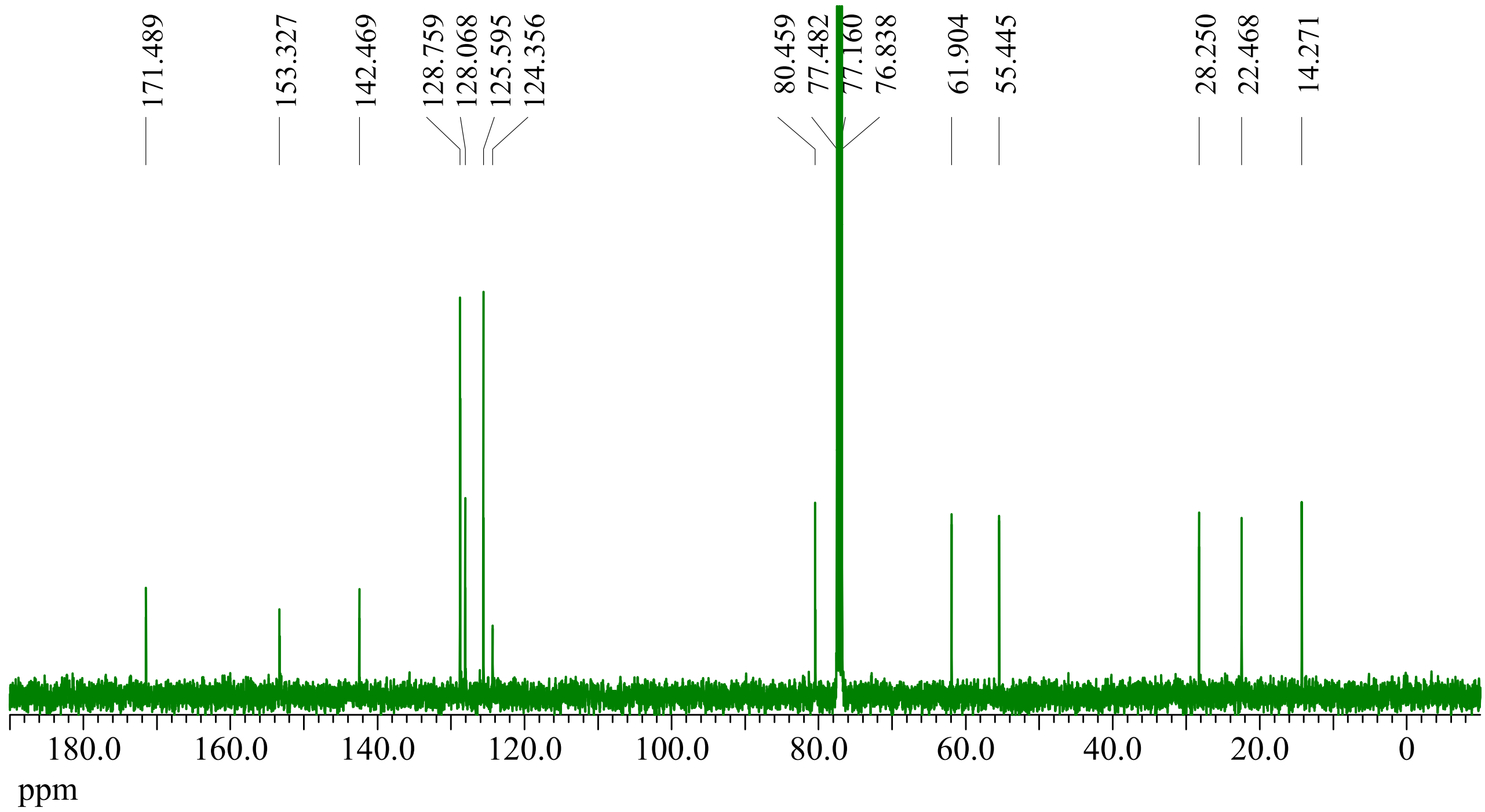

Minor isomer: Ethyl (4*R,5*S**)-2,2-dichloro-5-phenyl-3-(propan-2-ylidene)-1,2-oxagermolane-4-carboxylate** *anti-α*-**4**

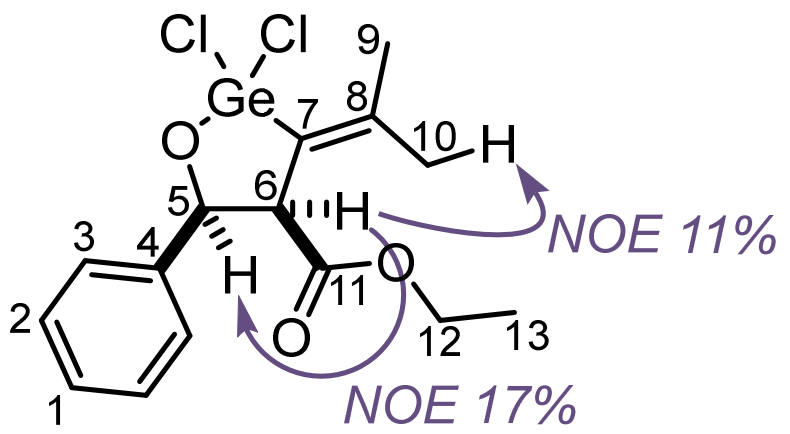

^{1}H NMR (400 MHz, $CDCl_3$) 7.41–7.25 (m, 5H), 5.28 (d, J = 5.2 Hz, 1H, 5-H), 3.90 (d, J = 5.2 Hz, 1H, 6-H), 3.75 (q, J = 7.2 Hz, 2H, 12-H), 2.14 (s, 3H, 9-H), 1.96 (s, 3H, 10-H), 0.91 (t, J = 7.2 Hz, 3H, 13-H); **$^{13}C\{^{1}H\}$ NMR** (100 MHz, $CDCl_3$) 169.8 (s, C-11), 152.1 (s, C-8), 138.8 (s, C-4), 128.5 (s, C-7), 128.3 (d, C-2), 128.1 (d, C-1), 125.7 (d, C-3), 78.7 (d, C-5), 61.0 (t, C-12), 54.8 (d, C-6), 27.8 (s, C-9), 22.2 (q, C-10), 13.8 (q, C-13).

¹H NMR (400 MHz, $CDCl_3$)

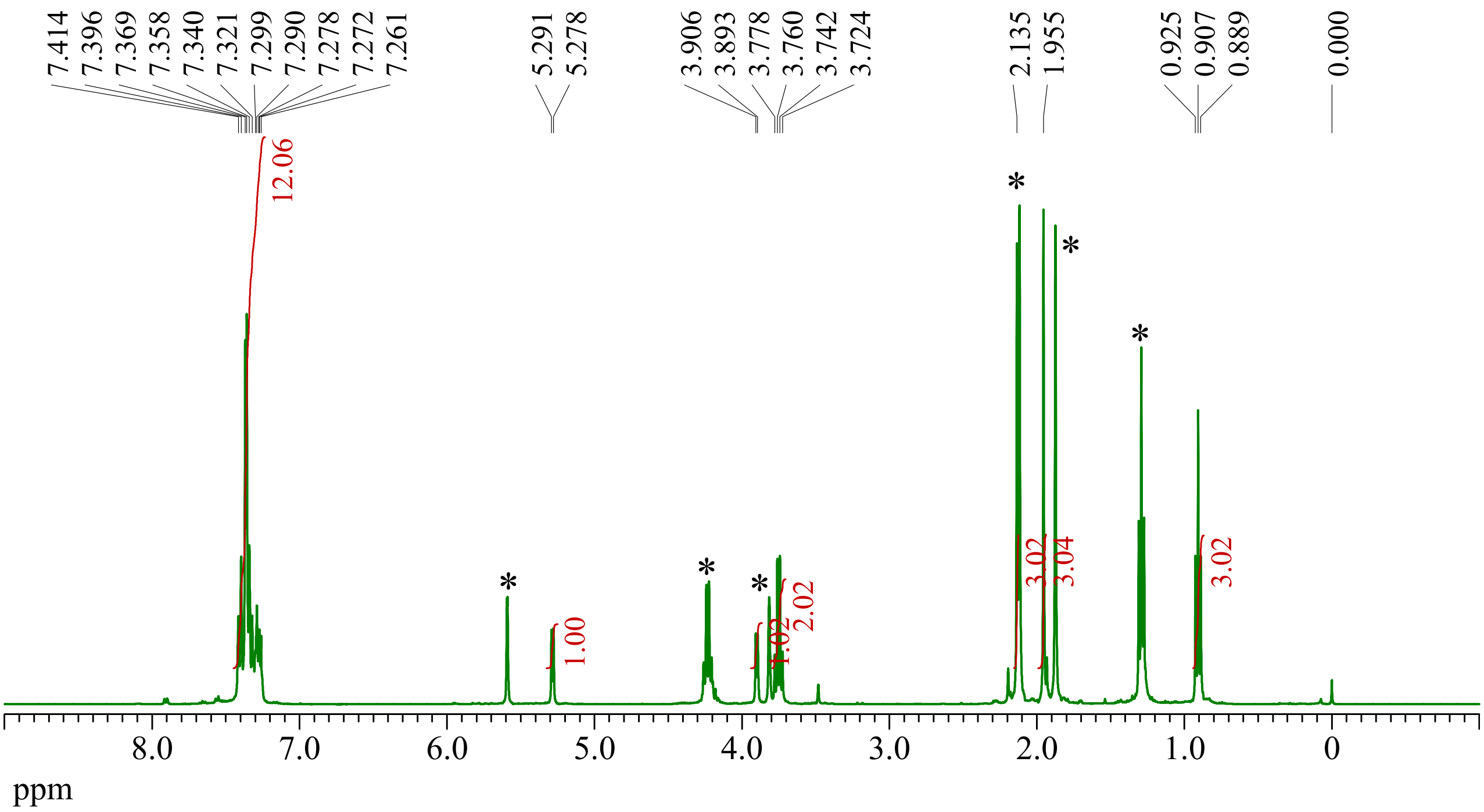

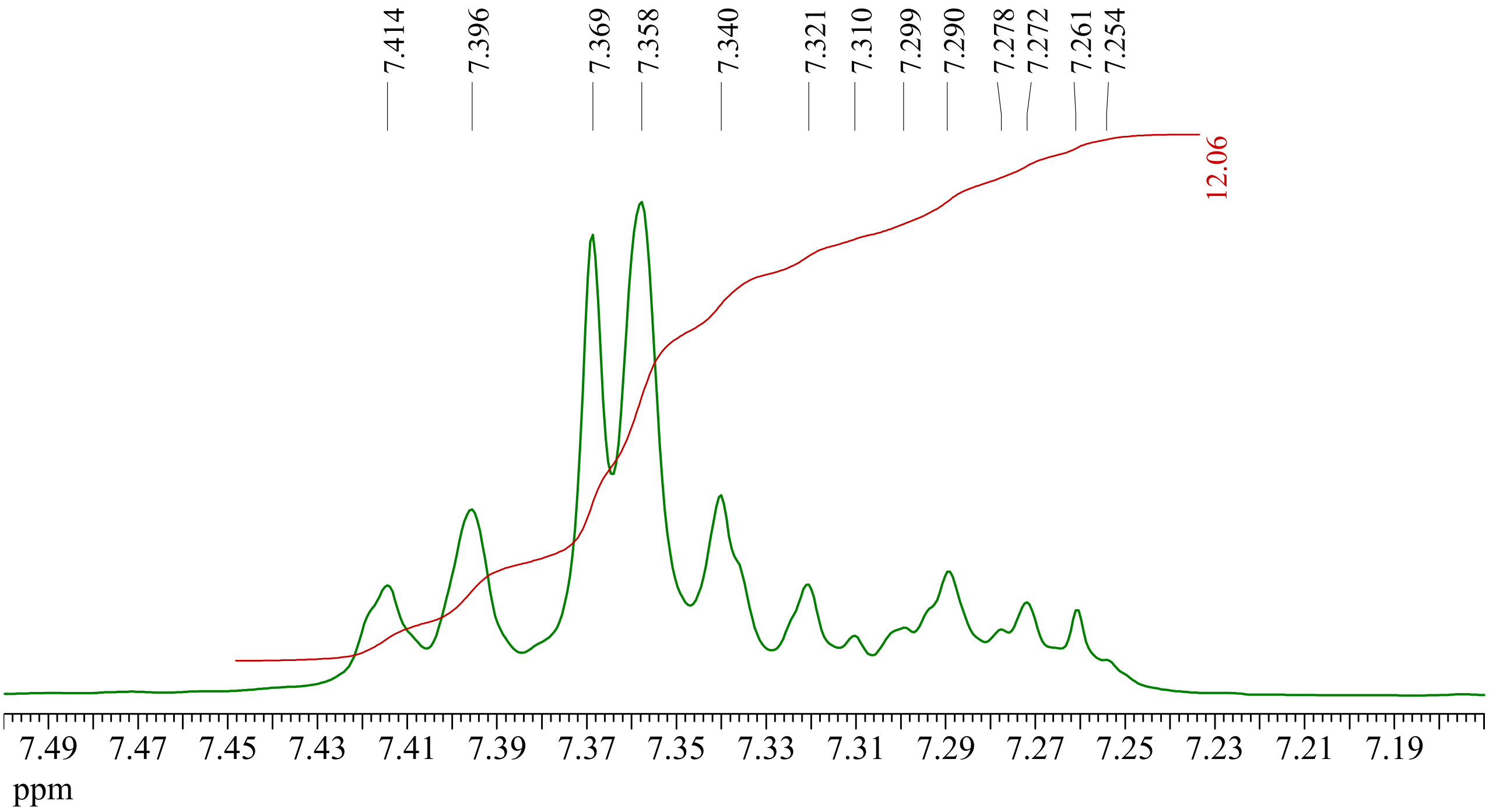

$^{13}C\{^1H\}$ NMR (100 MHz, $CDCl_3$)

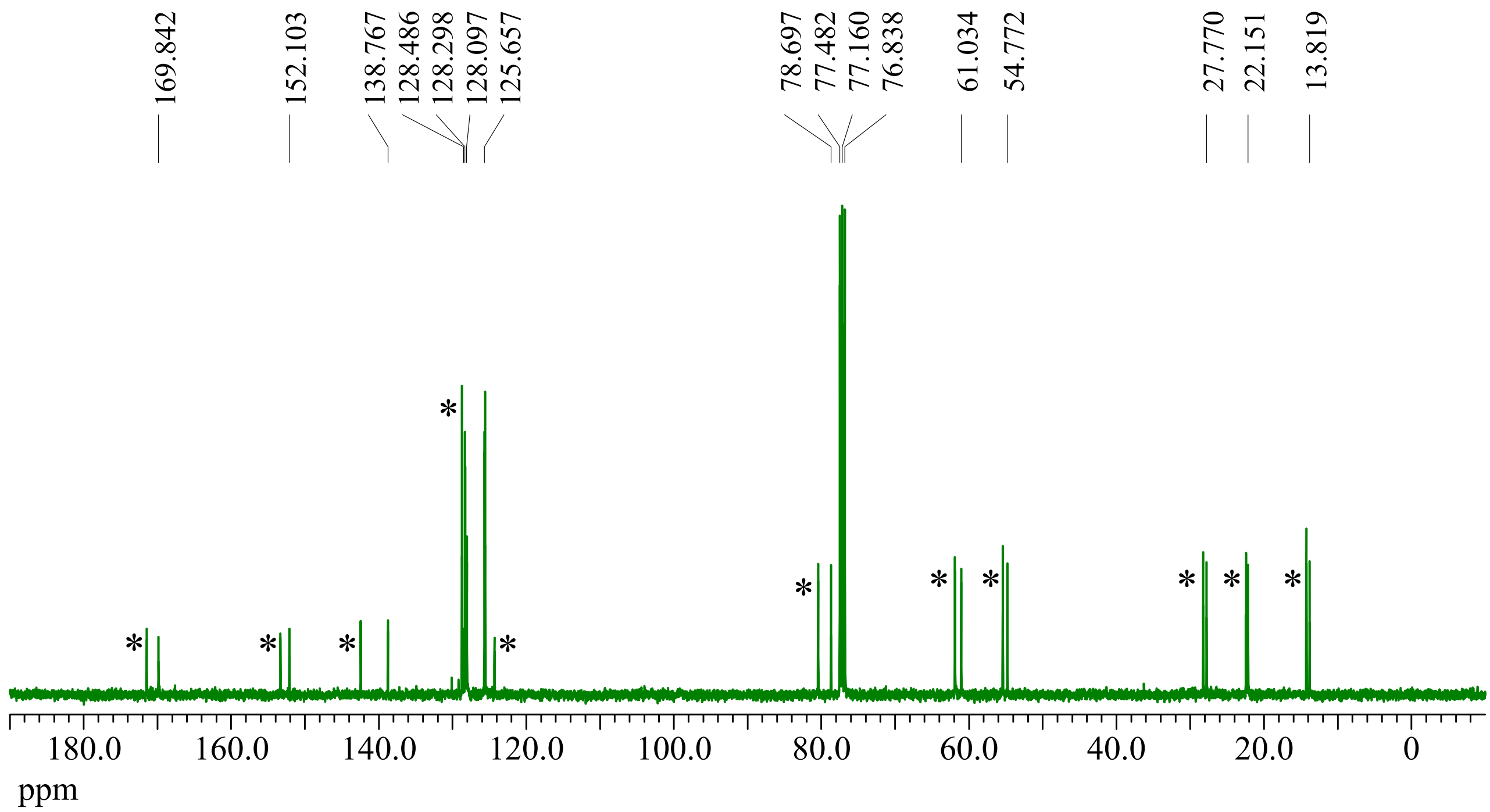

Asterisk marks (*) represent the signals of the inseparatable *anti*-*α*-**4ga**.

3-6. *anti-α*-Selective aldol reaction of allenoate 1 with benzaldehyde 3a using $GeCl_2$-dioxane

Benzyl (4*R,5*S**,*E*)-2,2-dichloro-3-(2,2-dimethylpropylidene)-5-phenyl-1,2-oxagermolane-4-carboxylate *anti-α*-4ca**

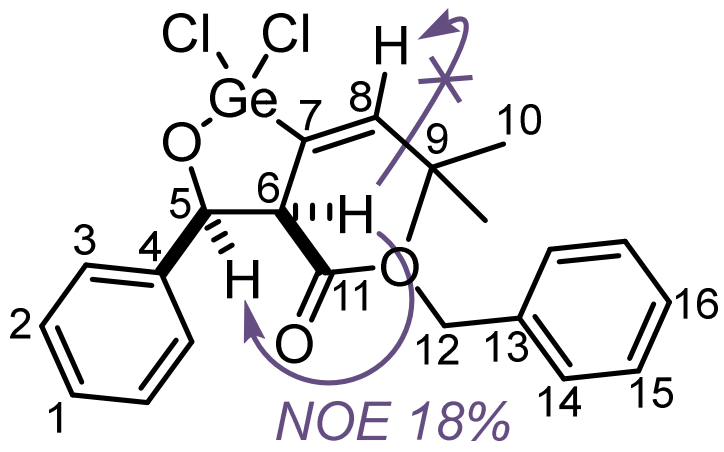

To a mixture of **1c** (0.0346 g, 0.150 mmol) and benzaldehyde **3a** (0.0202 mL, 0.200 mmol) in EtOAc (1.0 mL) was added $GeCl_2$-dioxane (0.0230 g, 0.0993 mmol). After the reaction mixture was stirred at room temperature for 18 h, the solvent was removed under vacuum. The yield was determined by ^{1}H NMR of the crude product using 1,1,2,2-tetrachloroethane as an internal standard (75%, *anti*-*α*-**4ca**/*anti*-*γ*-**4ca** = 83/17, single diastereomer).

To obtain the spectrodata of the isolated product, some conditions were modified. To a mixture of **1c** (1.0 eq) and benzaldehyde **3a** (2.0 eq) in CH_3CN (1.0 mL) was added $GeCl_2$-dioxane (1.0 eq). After the reaction mixture was stirred at room temperature for 18 h, the generated white solid was filtered off with CH_3CN. The filtrate was evaporated and careful extraction of the crude mixture with hexane gave *anti*-*α*-**4ca** in pure form as colorless neat.

IR (ATR) 3033 (w), 2961 (s), 1724 (s), 1456 (m), 1322 (m), 1263 (m), 1170 (s), 1027 (m), 872 (m) cm^{-1}; **^{1}H NMR** (400 MHz, $CDCl_3$) 7.37–7.26 (m, 8H), 7.02–6.99 (m, 2H, 14-H), 6.35 (d, *J* = 1.2 Hz, 1H, 8-H), 5.22 (d, *J* = 5.2 Hz, 1H, 5-H), 4.82 (d, *J* = 12.4 Hz, 1H, 12-H), 4.61 (d, *J* = 12.0 Hz, 1H, 12-H), 4.21 (dd, *J* = 5.0, 1.0 Hz, 1H, 6-H), 1.13 (s, 9H, 10-H); **^{13}C{^{1}H} NMR** (100 MHz, $CDCl_3$) 169.5 (s, C-11), 157.1 (d, C-8), 138.5 (s, C-4), 134.9 (s, C-13), 132.2 (s, C-7), 129.2 (d), 128.9 (d, C-14), 128.52 (d), 128.46 (d), 128.4 (d), 125.7 (d, C-3), 79.5 (d, C-5), 67.0 (t, C-12), 53.3 (d, C-6), 36.7 (s, C-9), 30.0 (q, C-10); **HRMS** (DART$^+$) Calculated ($C_{22}H_{25}O_3Cl_2Ge$): 481.0387 ($[M+H]^+$), Found: 481.0379 ($[M+H]^+$).

^{1}H NMR (400 MHz, $CDCl_3$)

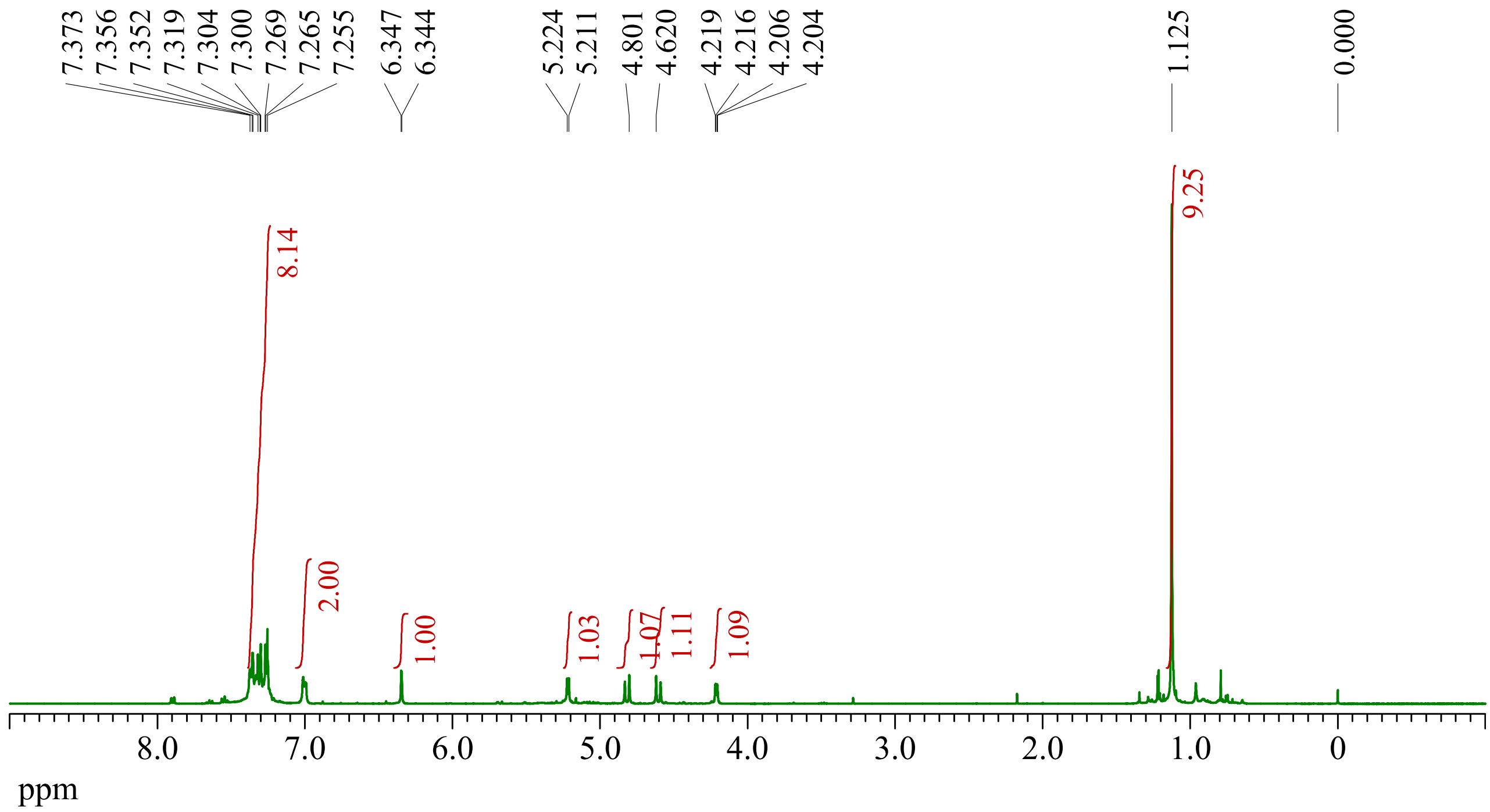

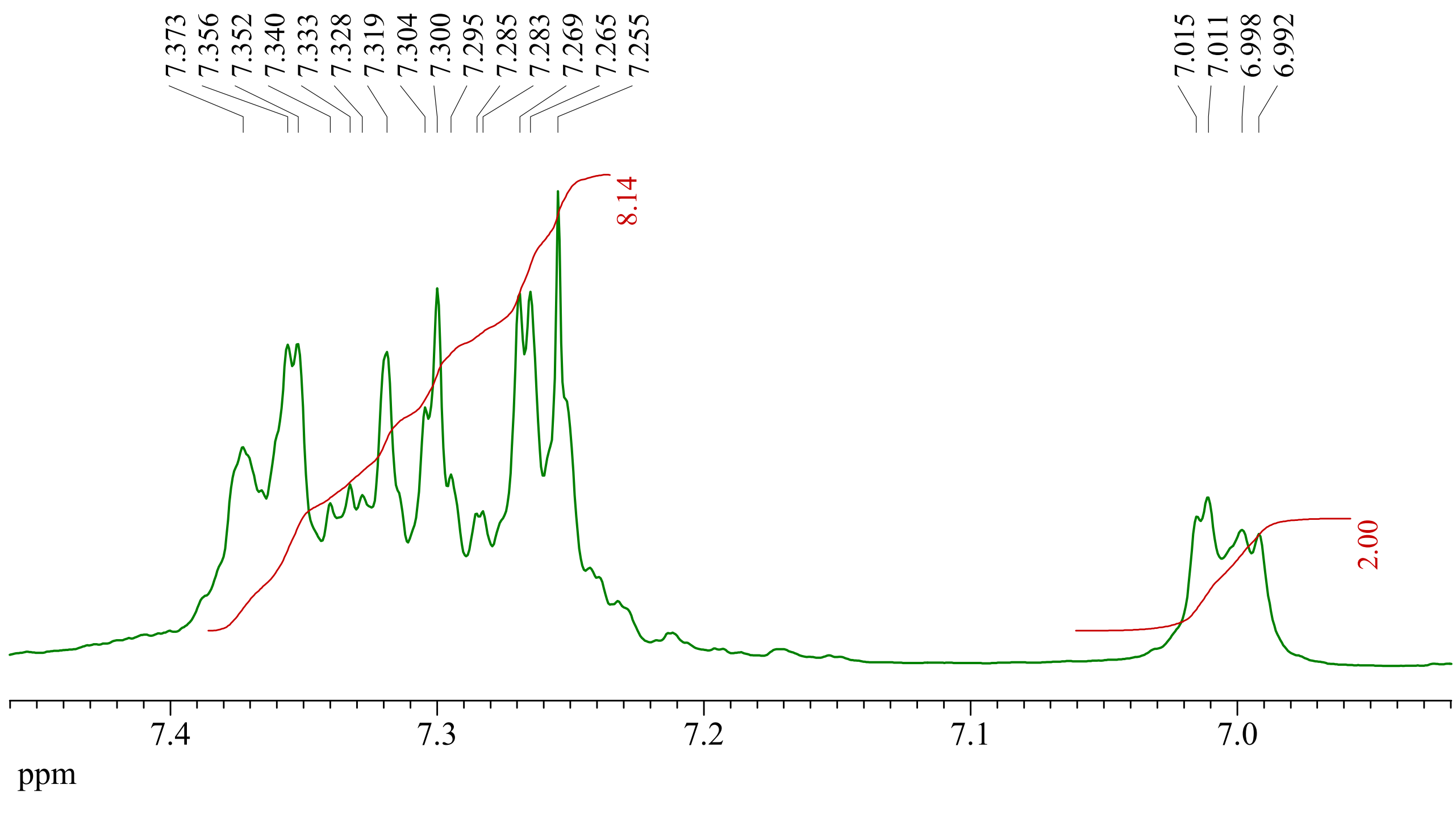

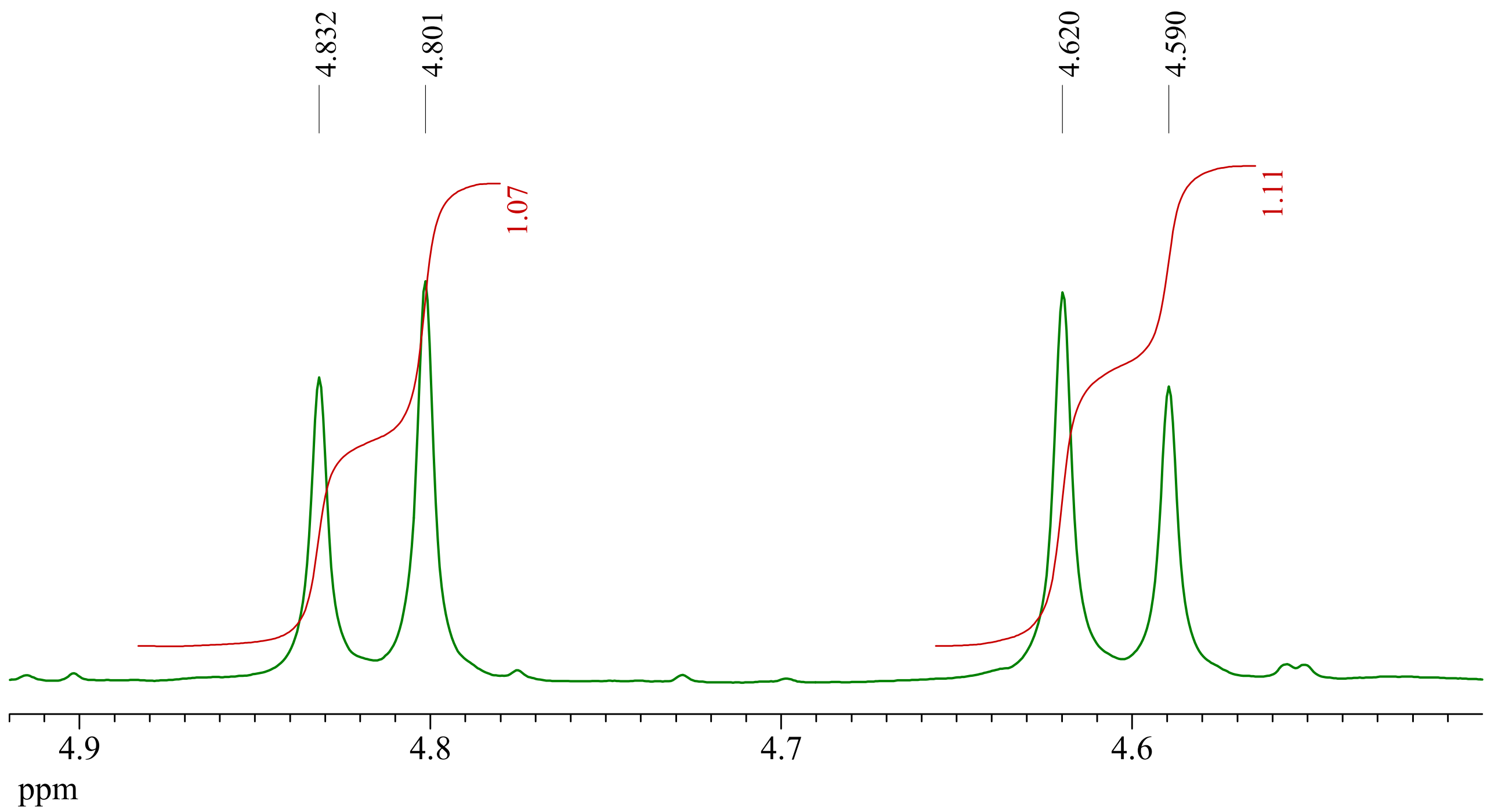

$^{13}C\{^1H\}$ NMR (100 MHz, $CDCl_3$)

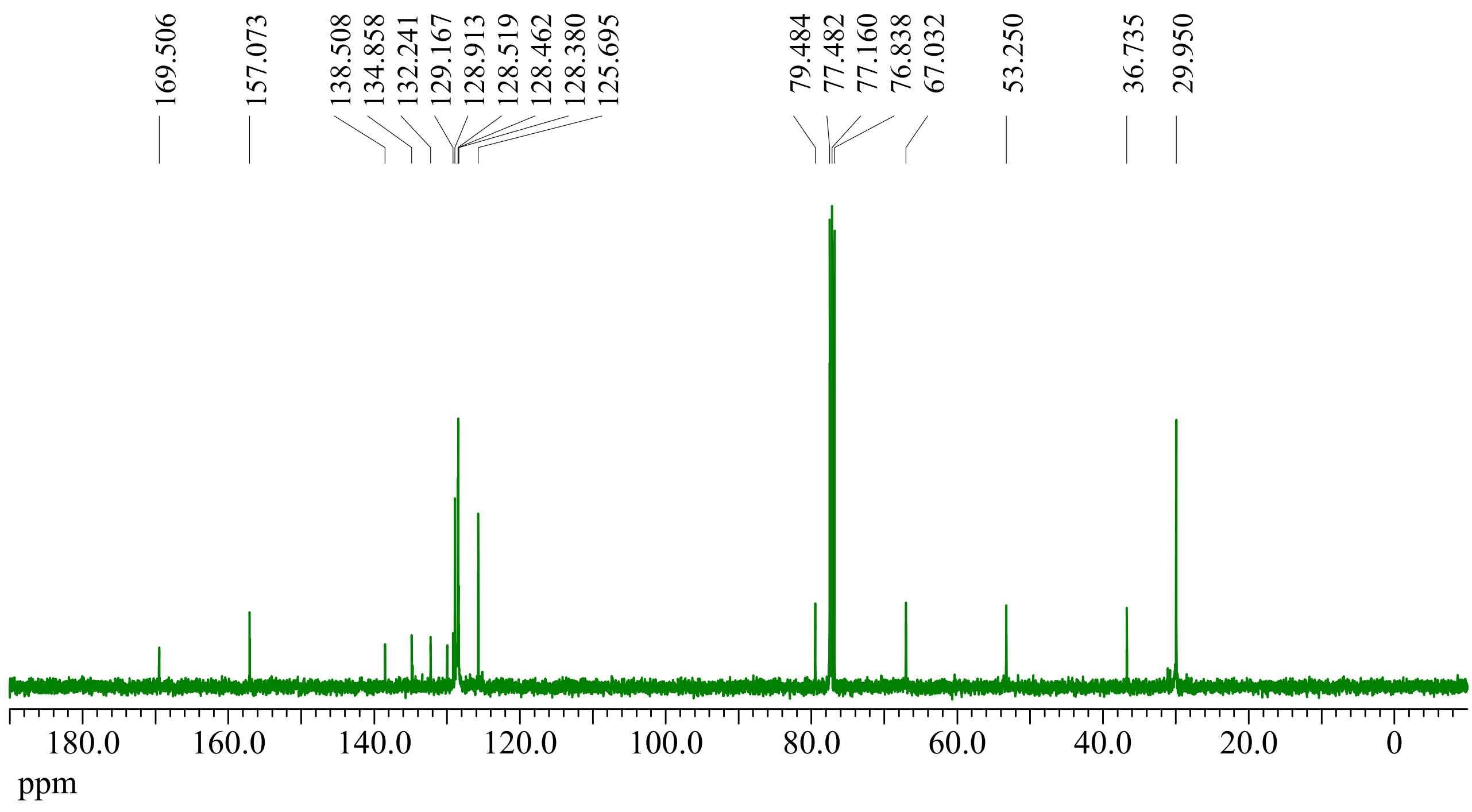

Phenyl (4*R,5*S**,*E*)-2,2-dichloro-3-(2,2-dimethylpropylidene)-5-phenyl-1,2-oxagermolane-4-carboxylate** *anti*-*α*-**4da**

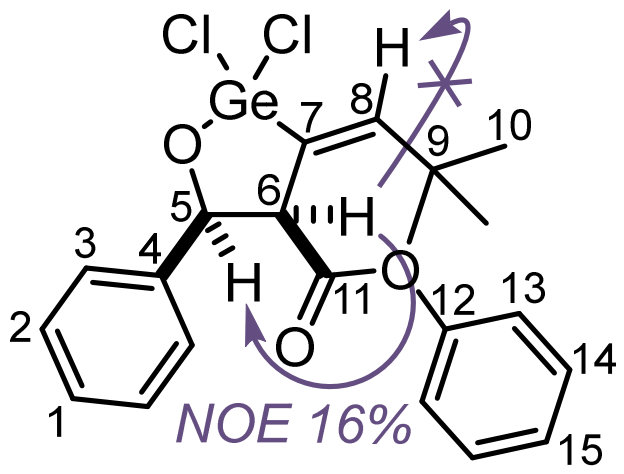

Conditions A

To a mixture of **1d** (0.0323 g, 0.0149 mmol), benzaldehyde **3a** (0.0202 mL, 0.200 mmol), and $BF_3·OEt_2$ (0.0252 mL, 0.200 mmol) in CH_3CN (1.0 mL) was added $GeCl_2$-dioxane (0.0231 g, 0.0997 mmol). After the reaction mixture was left to stand at room temperature for 18 h, the solvent was removed under vacuum. The yield was determined by ^{1}H NMR of the crude product using 1,1,2,2-tetrachloroethane as an internal standard (13%, *anti*-*α*-**4da**/*anti*-*γ*-**4da** = 100/0, single isomer).

Conditions B

To a mixture of **1d** (0.0326 g, 0.0151 mmol) and benzaldehyde **3a** (0.0202 mL, 0.200 mmol) in EtOAc (1.0 mL) was added $GeCl_2$-dioxane (0.0230 g, 0.0993 mmol). After the reaction mixture was stirred at room temperature for 18 h, the solvent was removed under vacuum. The yield was determined by ^{1}H NMR of the crude product using 1,1,2,2-tetrachloroethane as an internal standard (68%, *anti*-*α*-**4da**/*anti*-*γ*-**4da** = 84/16, single diastereomer).

To obtain the spectrodata of the isolated product, the crude mixture of ***conditions B*** was washed with hexane and Et_2O, and careful extraction with benzene gave *anti*-*α*-**4da** in pure form as a pale brown solid.

mp 161.5–162.4 °C; **IR** (ATR) 3064 (w), 2963 (m), 1747 (m), 1592 (w), 1491 (m), 1325 (w), 1190 (s), 1164 (s), 1025 (m) cm^{-1}; **^{1}H NMR** (400 MHz, $CDCl_3$) 7.52 (d, *J* = 7.2 Hz, 2H, 3-H), 7.43 (t, *J* = 7.2 Hz, 2H, 2-H), 7.38 (t, *J* = 7.2 Hz, 1H, 1-H), 7.22 (t, *J* = 7.8 Hz, 2H, 14-H), 7.13 (t, *J* = 7.4 Hz, 1H, 15-H), 6.46–6.43 (m, 3H, 8-H, 13-H), 5.37 (d, *J* = 5.2 Hz, 1H, 5-H), 4.45 (dd, *J* = 5.0, 1.4 Hz, 1H, 6-H), 1.29 (s, 9H, 10-H); **^{13}C{^{1}H} NMR** (100 MHz, $CDCl_3$) 168.2 (s, C-11), 157.7 (d, C-8), 149.7 (s, C-12). 138.5 (s, C-4), 131.9 (s, C-7), 129.4 (d, C-14), 128.7 (d, C-2), 128.5 (d, C-1), 126.2 (d, C-15), 126.0 (d, C-3), 121.2 (d, C-13), 79.3 (d, C-5), 52.9 (d, C-6), 36.9 (s, C-9), 30.1 (q, C-10); **HRMS** (DART$^+$) Calculated ($C_{21}H_{23}O_3Cl_2Ge$): 467.0231 ([M+H]$^+$), Found: 467.0240 ([M+H]$^+$).

[1]**H NMR** (400 MHz, $CDCl_3$)

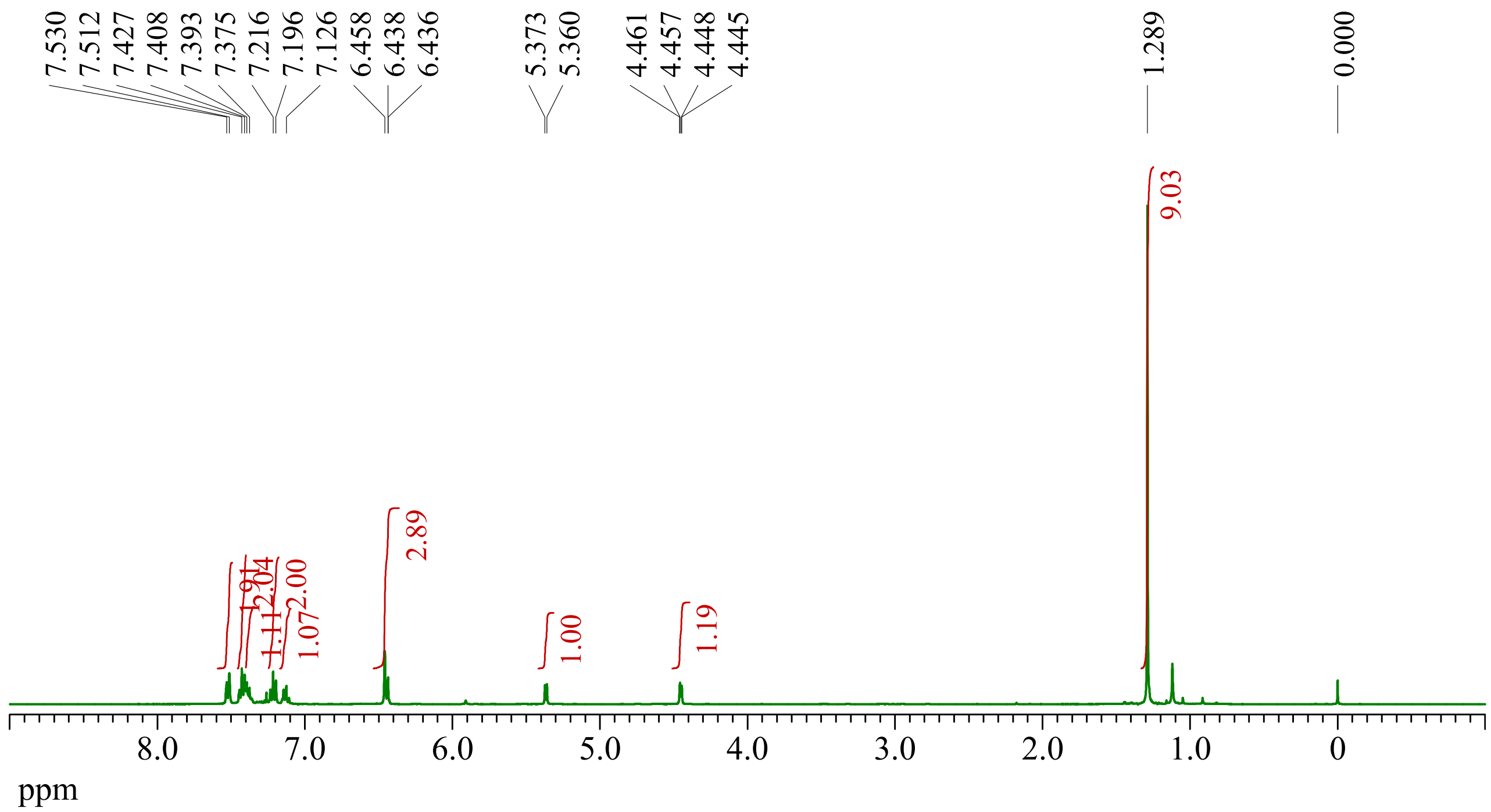

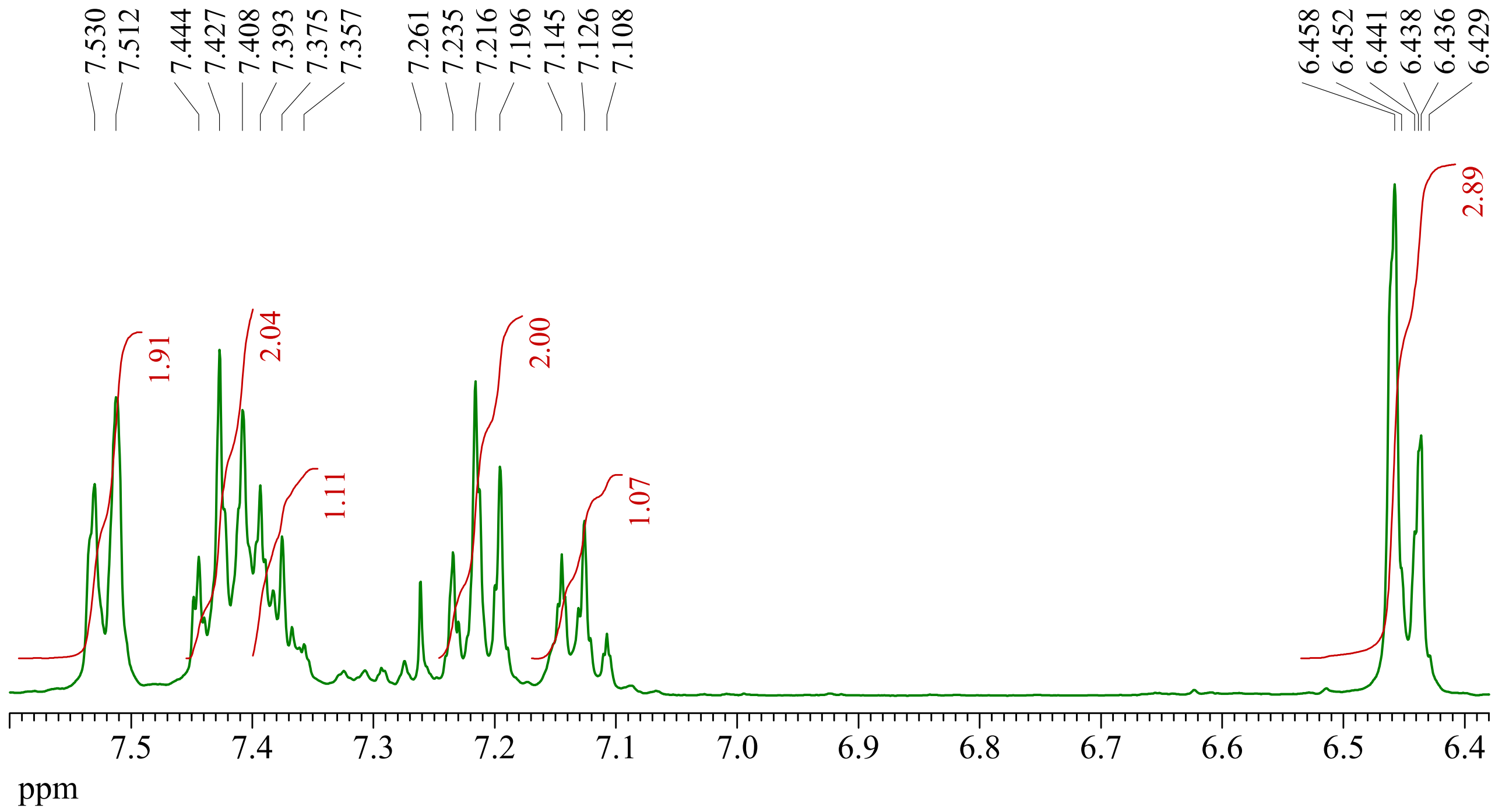

$^{13}C\{^1H\}$ NMR (100 MHz, $CDCl_3$)

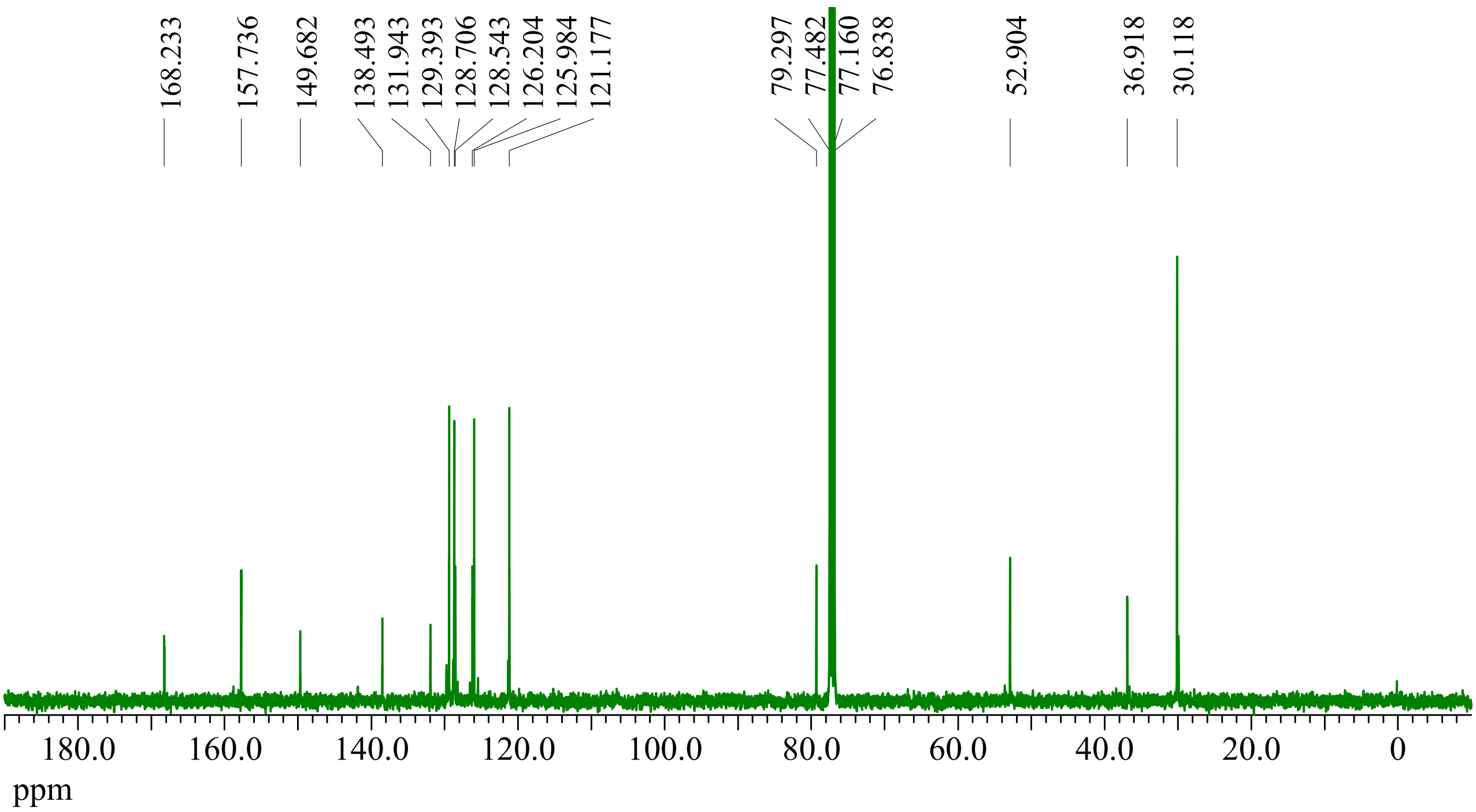

Ethyl (4*R,5*S**,*E*)-3-{(*tert*-butyldimethylsilyl)methylene}-2,2-dichloro-5-phenyl-1,2-oxagermolane-4-carboxylate** ***anti*-α-4fa**

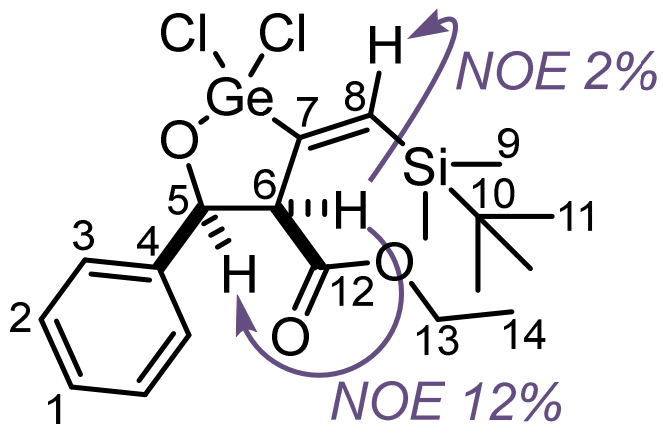

Conditions A

To a mixture of **1f** (0.0167 g, 0.0738 mmol), benzaldehyde **3a** (0.0101 mL, 0.100 mmol), and $BF_3 \cdot OEt_2$ (0.0126 mL, 0.100 mmol) in CH_3CN (0.50 mL) was added $GeCl_2$-dioxane (0.0114 g, 0.0492 mmol). After the reaction mixture was left to stand at room temperature for 18 h, the obtained colorless solid was filtrated and washed with CH_3CN, to give *anti*-*α*-**4fa** as a colorless solid (0.0165 g, 70%, single isomer).

Conditions B

To a mixture of **1f** (0.0170 g, 0.0751 mmol) and benzaldehyde **3a** (0.0101 mL, 0.100 mmol) in EtOAc (0.50 mL) was added $GeCl_2$-dioxane (0.0114 g, 0.0492 mmol). After the reaction mixture was stirred at room temperature for 18 h, the solvent was removed under vacuum. The yield was determined by ^1H NMR of the crude product using 1,1,2,2-tetrachloroethane as an internal standard (42%, single isomer).

To obtain the spectrodata of the isolated product, the crude mixture of ***conditions B*** was washed with hexane and Et_2O to give the target germacycle in pure form as a pale brown solid.

mp 146.5–147.4 °C; **IR** (ATR) 3064 (w), 2930 (m), 2858 (m), 1719 (s), 1468 (m), 1254 (s), 1185 (s), 1025 (s), 822 (s) cm^{-1}; **^{1}H NMR** (400 MHz, $CDCl_3$) 7.46 (d, *J* = 7.6 Hz, 2H, 3-H), 7.36 (t, *J* = 7.6 Hz, 2H, 2-H), 7.27 (t, *J* = 7.4 Hz, 1H, 1-H), 6.70 (s, 1H, 8-H), 5.93 (s, 1H, 5-H), 4.43 (dq, *J* = 11.2, 7.6 Hz, 1H, 13-H), 4.39 (dq, *J* = 11.2, 7.2 Hz, 1H, 13-H), 3.66 (s, 1H, 6-H), 1.34 (t, *J* = 7.2 Hz, 3H, 14-H), 0.71 (s, 9H, 11-H), –0.37 (s, 3H, 9-H), –0.39 (s, 3H, 9-H); **^{13}C{^{1}H} NMR** (100 MHz, $CDCl_3$) 173.1 (s, C-12), 151.7 (s, C-7), 148.8 (d, C-8), 141.0 (s, C-4), 128.4 (d, C-2), 128.1 (d, C-1), 126.5 (d, C-3), 76.8 (d, C-5), 63.0 (t, C-13), 58.2 (d, C-6), 26.2 (q, C-11), 17.1 (s, C-10), 14.2 (q, C-14), –5.1 (q, C-9), –5.5 (q, C-9); **^{29}Si{^{1}H} NMR** (78.5 MHz, $CDCl_3$, Me_4Si in $CDCl_3$ was employed as an external standard (0.00 ppm).) 0.71; **HRMS** (DART$^+$) Calculated ($C_{19}H_{29}O_3SiCl_2Ge$): 477.0469 ($[M+H]^+$); Found: 451.0476 ($[M+H]^+$).

^{1}H NMR (400 MHz, $CDCl_3$)

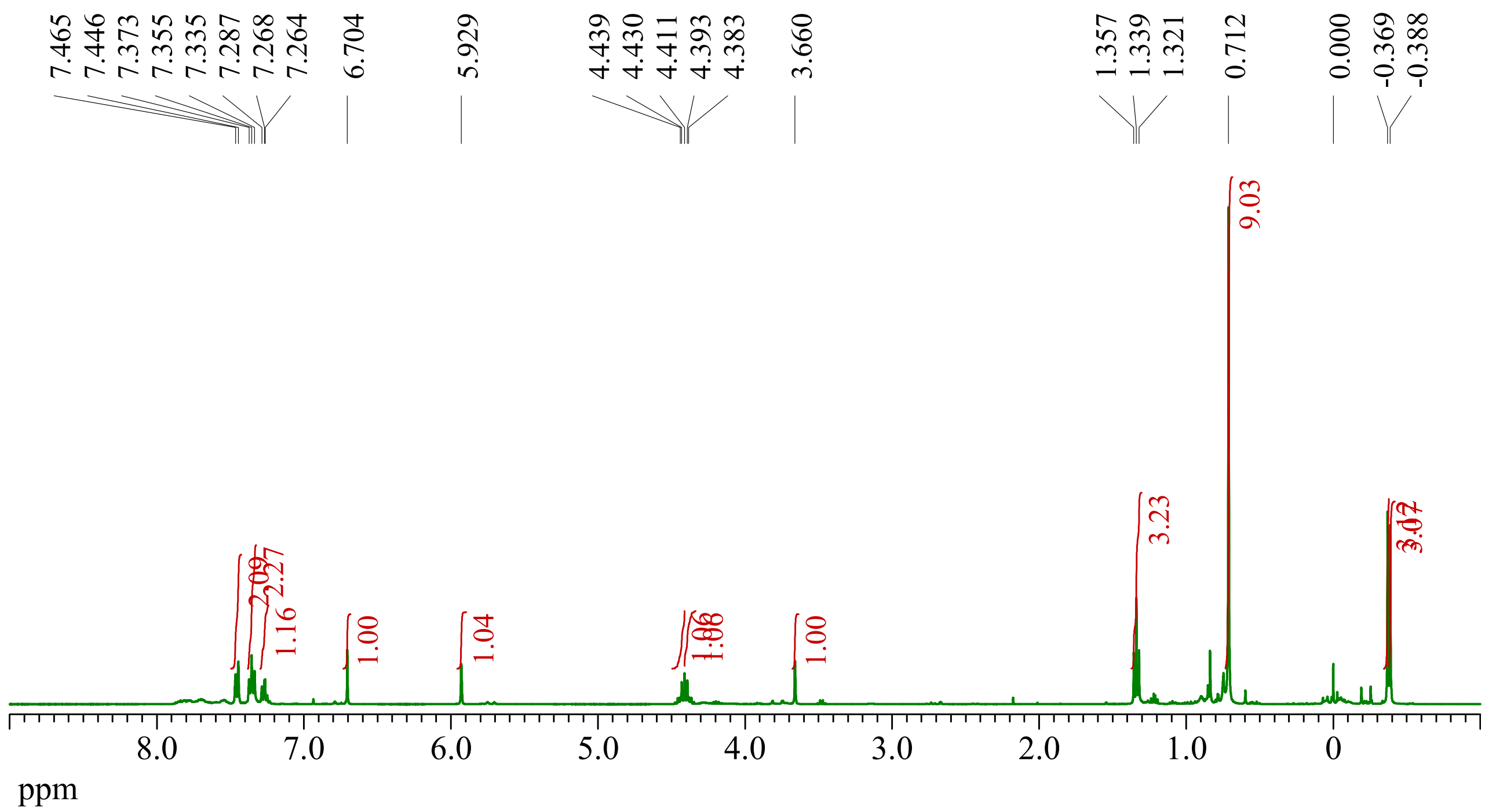

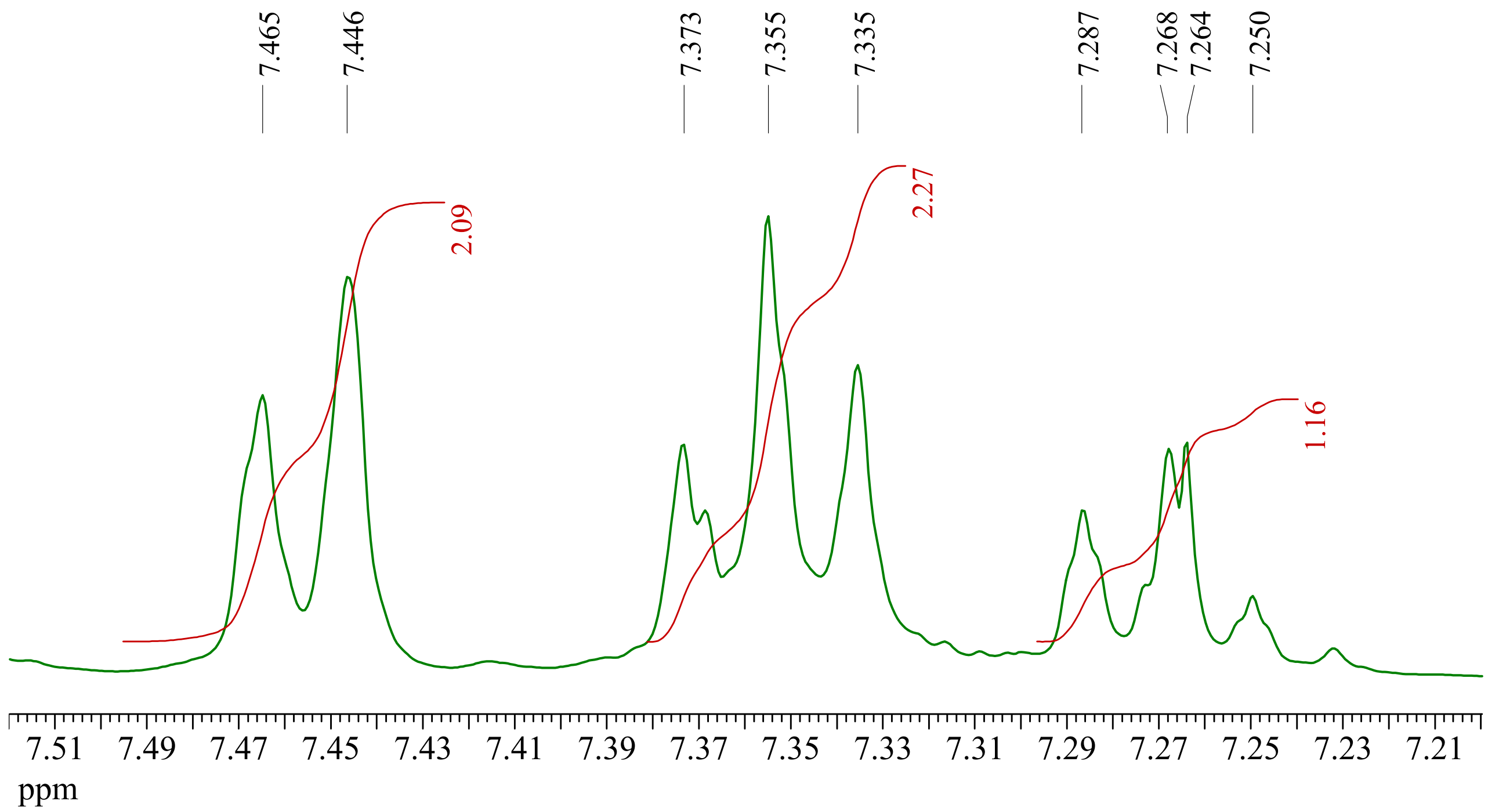
7.465
7.446
7.373
7.355
7.335
7.287
7.268
7.264
7.250
2.09
2.27
1.16
7.51 7.49 7.47 7.45 7.43 7.41 7.39 7.37 7.35 7.33 7.31 7.29 7.27 7.25 7.23 7.21
ppm

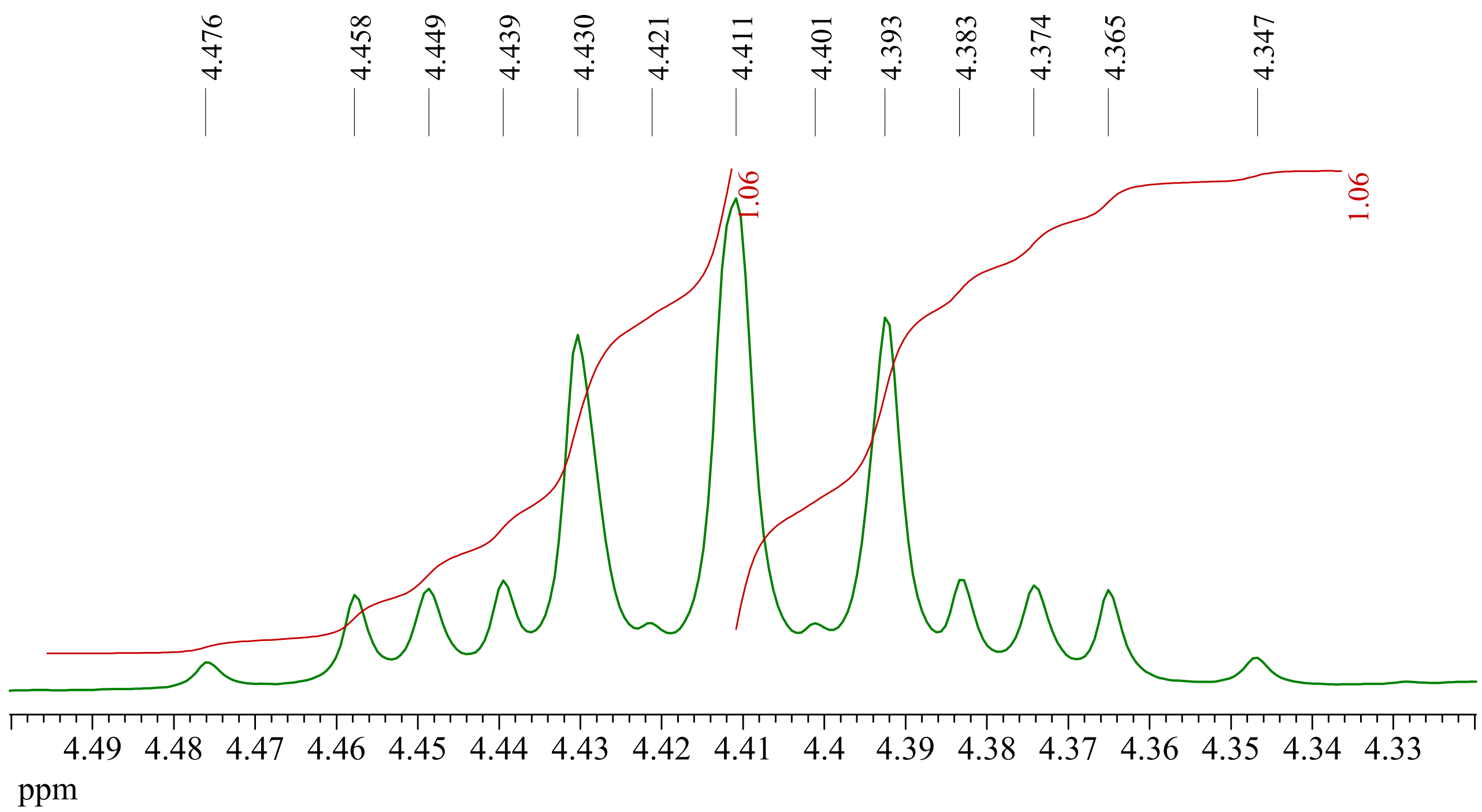
4.476
4.458
4.449
4.439
4.430
4.421
4.411
4.401
4.393
4.383
4.374
4.365
4.347
1.06
1.06
4.49 4.48 4.47 4.46 4.45 4.44 4.43 4.42 4.41 4.4 4.39 4.38 4.37 4.36 4.35 4.34 4.33
ppm

$^{13}C\{^1H\}$ NMR (100 MHz, $CDCl_3$)

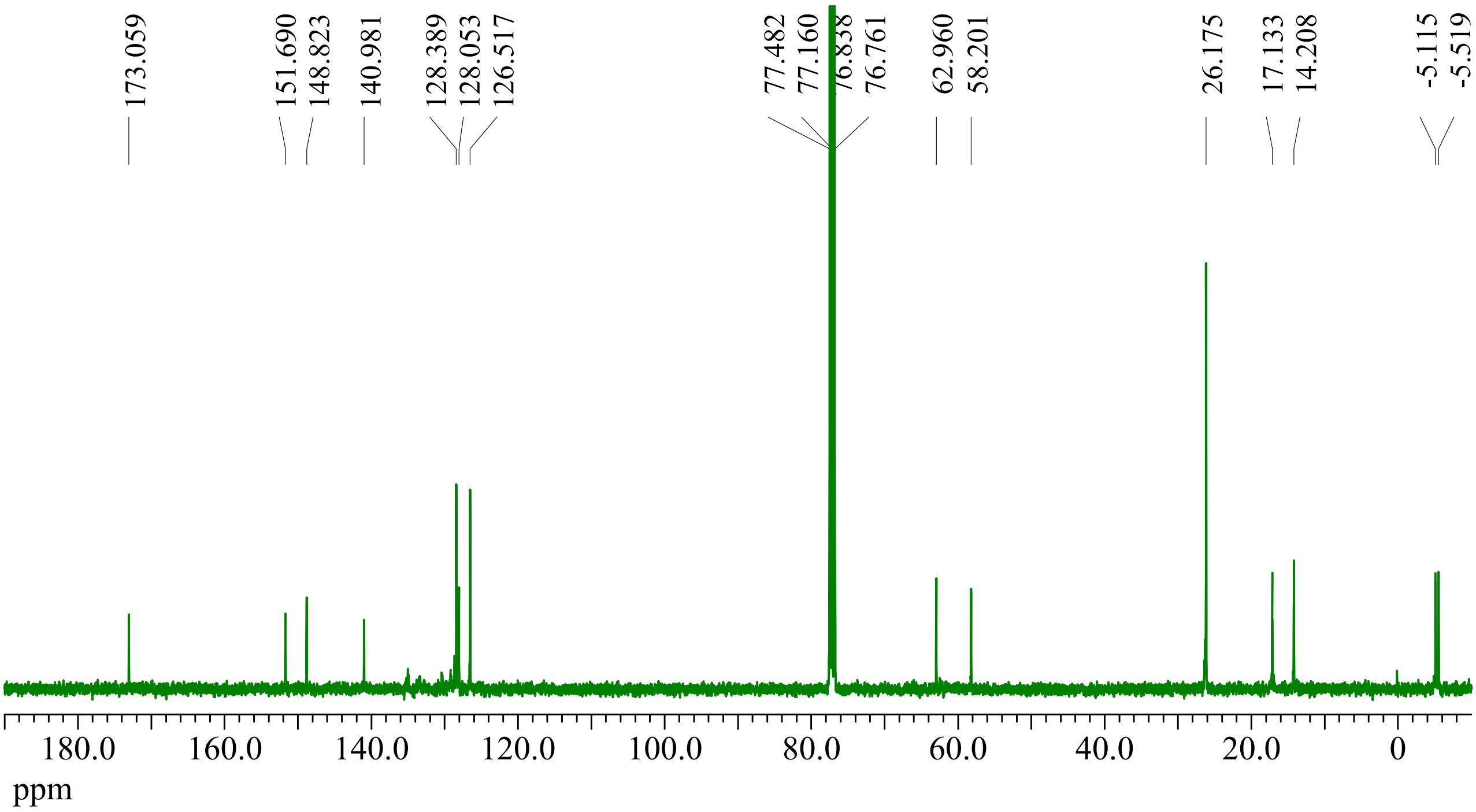

$^{29}Si\{^1H\}$ NMR (78.5 MHz, $CDCl_3$, Me_4Si in $CDCl_3$ was employed as an external standard (0.00 ppm).)

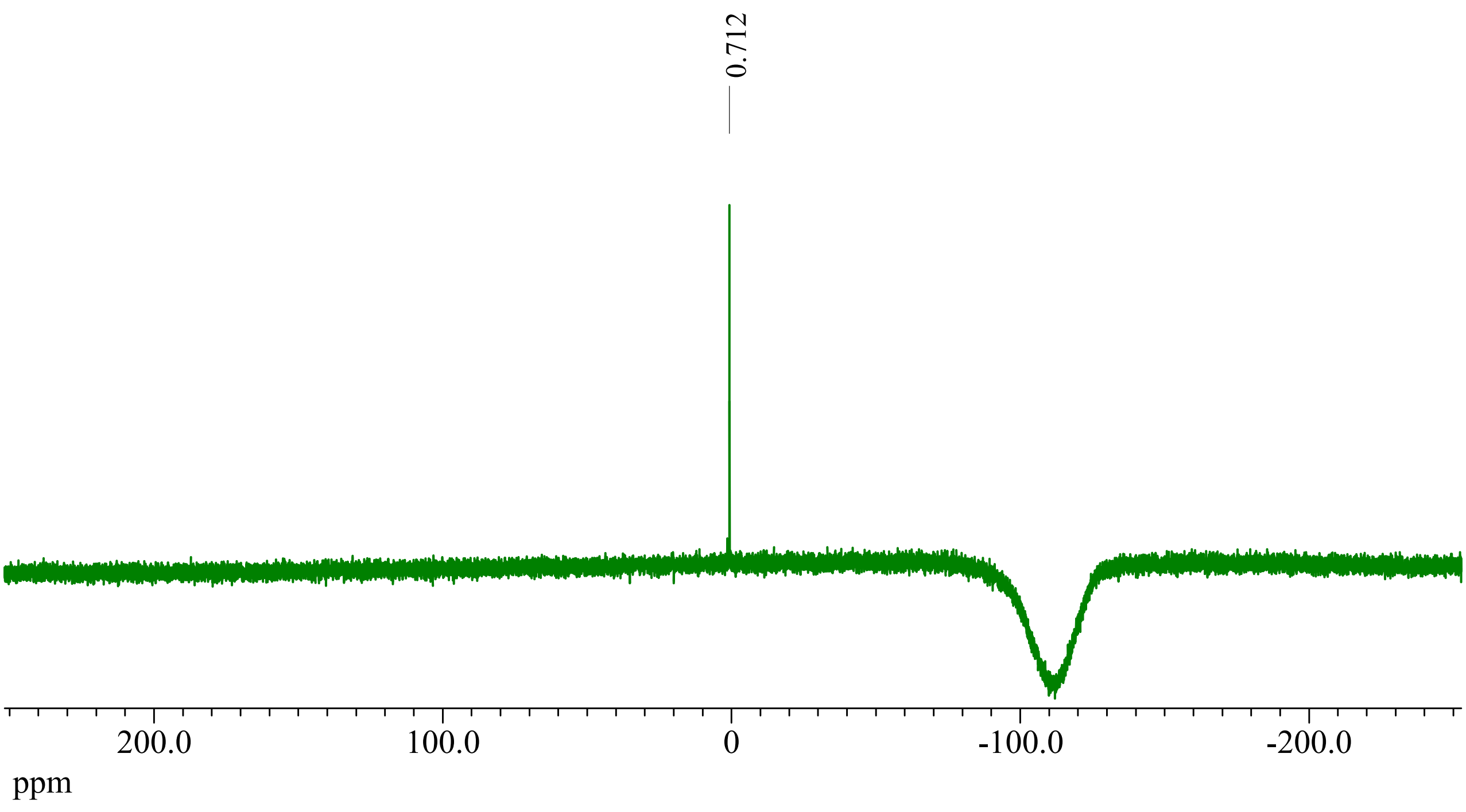

3-7. *anti-γ*-Selective aldol reaction of allenoate 1 with benzaldehyde 3a using $GeCl_2$-dioxane

Benzyl (*Z*)-2-{(4*R,5*S**)-4-(*tert*-butyl)-2,2-dichloro-5-phenyl-1,2-oxagermolan-3-ylidene}acetate** ***anti-γ*-4ca**

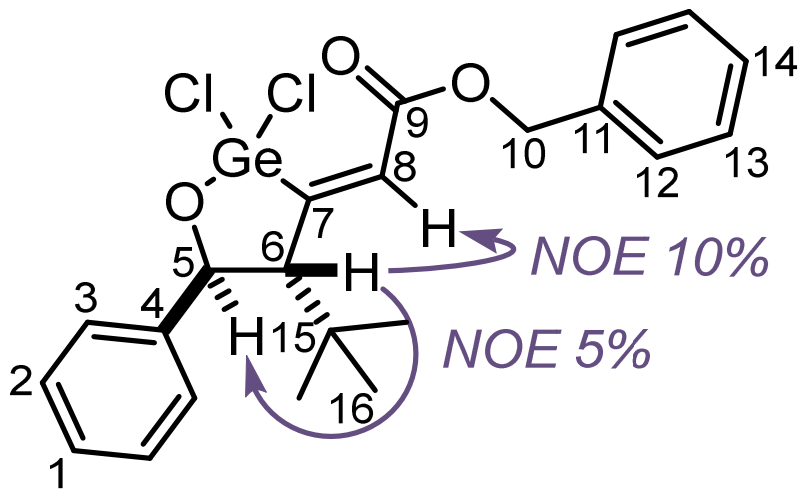

To a mixture of **1c** (0.0465 g, 0.202 mmol) and benzaldehyde **3a** (0.0202 mL, 0.200 mmol) in hexane (1.0 mL) was added $GeCl_2$-dioxane (0.0231 g, 0.0997 mmol). After the reaction mixture was stirred at room temperature for 18 h, the solvent was removed under vacuum. The yield was determined by ^{1}H NMR of the crude product using 1,1,2,2-tetrachloroethane as an internal standard (93%, *anti-α*-**4ca**/*anti-γ*-**4ca** = 3/97, single diastereomer).

To obtain the spectrodata of the isolated product, some conditions were modified. To a mixture of **1c** (1.0 eq) and benzaldehyde **3a** (2.0 eq) in hexane (1.0 mL) was added $GeCl_2$-dioxane (1.0 eq). After the reaction mixture was stirred at room temperature for 18 h, the generated white solid was filtered off with hexane. The filtrate was evaporated and careful extraction of the crude mixture with hexane gave *anti-γ*-**4ca** in pure form as colorless neat.

IR (ATR) 3033 (w), 2964 (m), 1688 (m), 1454 (w), 1386 (w), 1326 (s), 1212 (m), 1101 (w), 1007 (w) cm^{-1}; **^{1}H NMR** (400 MHz, $CDCl_3$) 7.42–7.20 (m, 10H), 6.65 (d, J = 1.6 Hz, 1H, 8-H), 5.61 (s, 1H, 5-H), 5.41 (d, J = 12.0 Hz, 1H, 10-H), 5.32 (d, J = 12.4 Hz, 1H, 10-H), 2.86 (s, 1H, 6-H), 1.10 (s, 9H, 16-H); **^{13}C{^{1}H} NMR** (100 MHz, $CDCl_3$) 167.1 (s, C-9), 157.8 (s, C-7), 144.8 (s, C-4), 134.3 (s, C-11), 129.2 (d), 129.1 (d), 128.9 (d), 128.7 (d, C-8), 128.6 (d), 127.4 (d), 125.8 (d), 77.9 (d, C-5), 69.3 (t, C-10), 59.7 (d, C-6), 36.2 (s, C-15), 28.2 (q, C-16); **HRMS** (DART$^+$) Calculated ($C_{22}H_{25}O_3Cl_2Ge$): 481.0387 ([M+H]$^+$), Found: 481.0384 ([M+H]$^+$).

^{1}H NMR (400 MHz, $CDCl_3$)

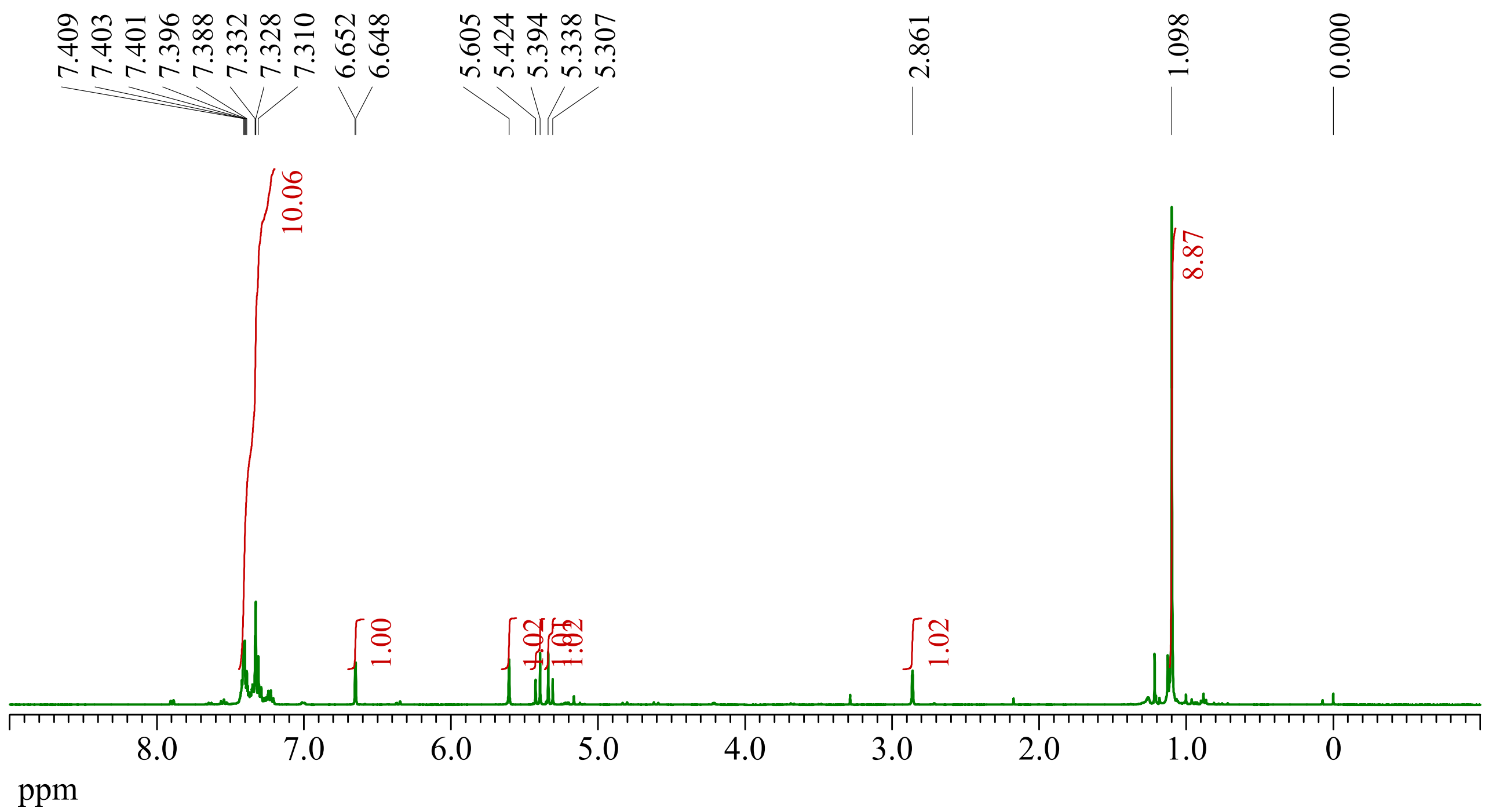

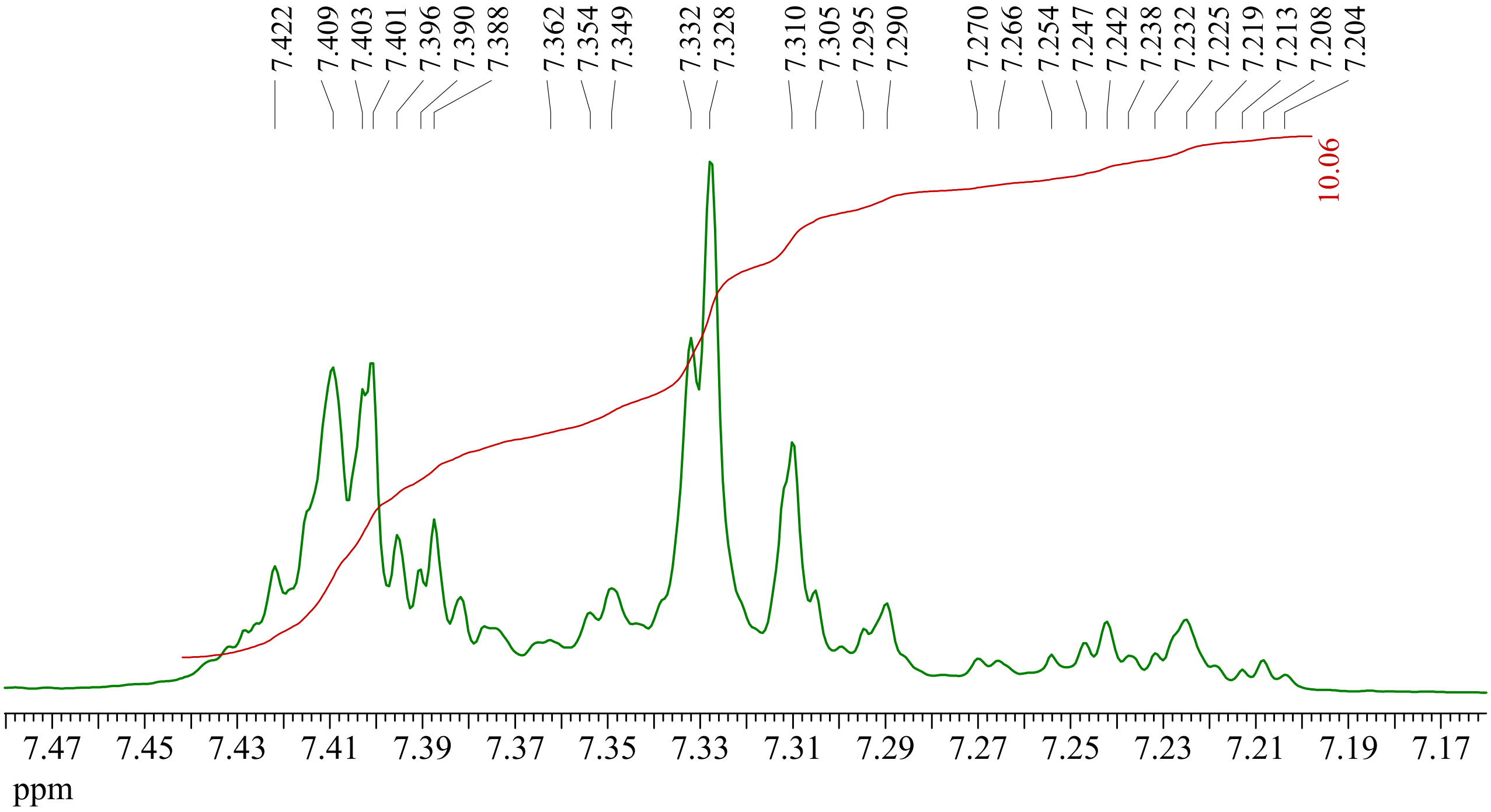

$^{13}C\{^1H\}$ NMR (100 MHz, $CDCl_3$)

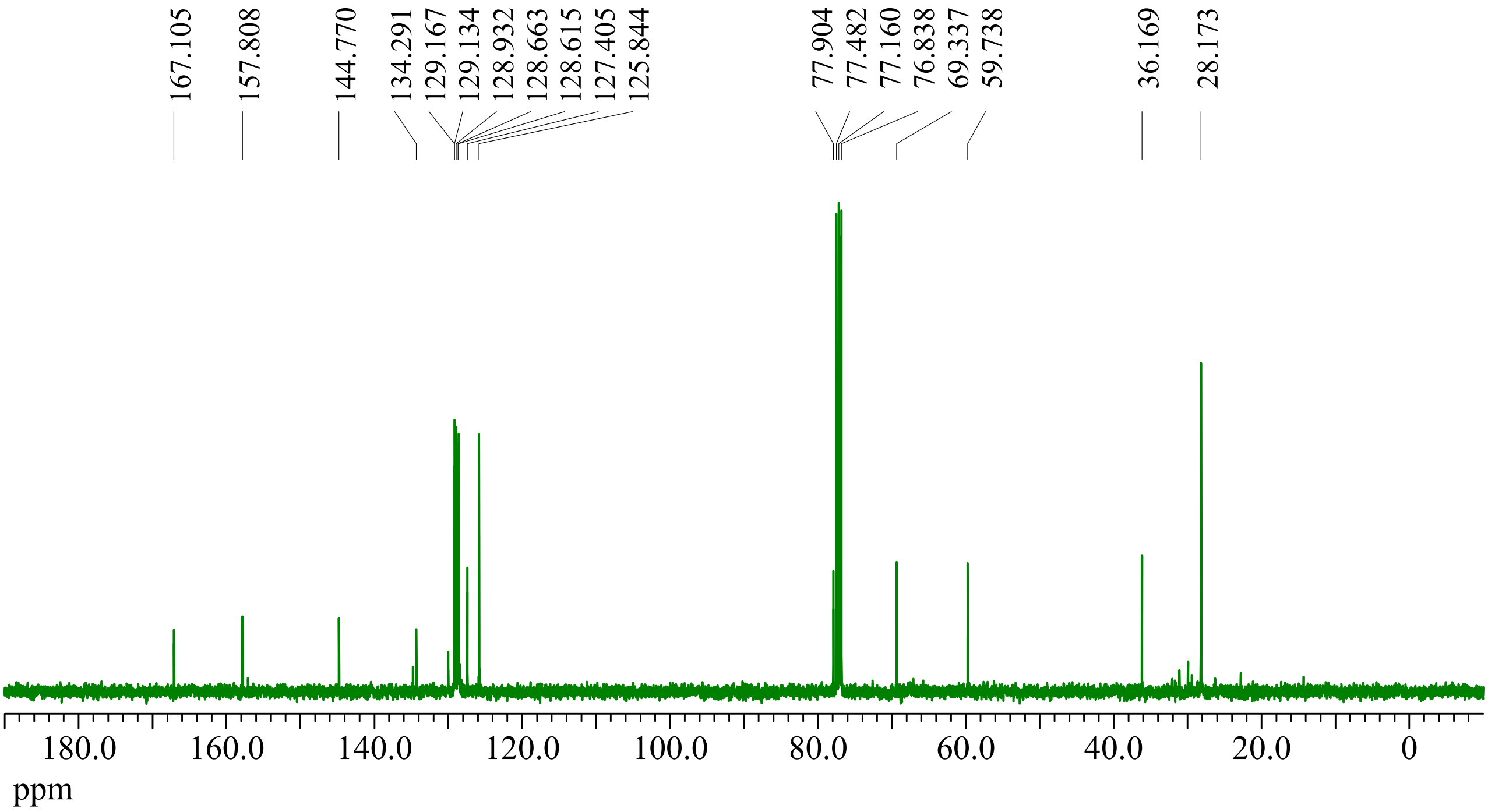

Phenyl (*Z*)-2-{(4*R,5*S**)-4-(*tert*-butyl)-2,2-dichloro-5-phenyl-1,2-oxagermolan-3-ylidene}acetate *anti*-*γ*-4da**

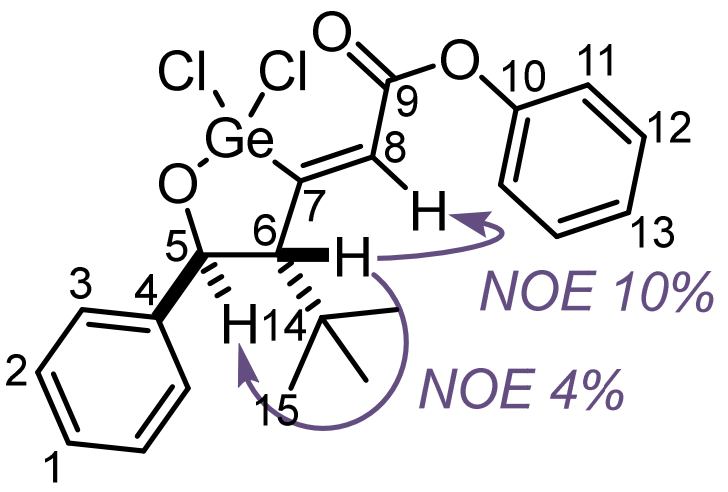

To a mixture of **1d** (0.0437 g, 0.202 mmol) and benzaldehyde **3a** (0.0202 mL, 0.200 mmol) in hexane (1.0 mL) was added $GeCl_2$-dioxane (0.0231 g, 0.0997 mmol). After the reaction mixture was stirred at room temperature for 18 h, the solvent was removed under vacuum. The yield was determined by ^{1}H NMR of the crude product using 1,1,2,2-tetrachloroethane as an internal standard (97%, *anti*-*α*-**4da**/*anti*-*γ*-**4da** = 15/85, single diastereomer).

To obtain the spectrodata of the isolated product, some conditions were modified. To a mixture of **1d** (1.0 eq) and benzaldehyde **3a** (2.0 eq) in hexane (1.0 mL) was added $GeCl_2$-dioxane (1.0 eq). After the reaction mixture was stirred at room temperature for 18 h, the generated white solid was filtered off with hexane. The filtrate was evaporated and careful extraction of the crude mixture with hexane gave *anti*-*γ*-**4da** in pure form as colorless neat.

IR (ATR) 3031 (w), 2965 (m), 1708 (m), 1491 (m), 1327 (s), 1253 (m), 1205 (s), 1096 (m), 1008 (m) cm^{-1}; **^{1}H NMR** (400 MHz, $CDCl_3$) 7.42 (t, *J* = 7.8 Hz, 2H, 12-H), 7.37 (d, *J* = 8.0 Hz, 2H, 3-H), 7.34 (t, *J* = 8.4 Hz, 2H, 2-H), 7.28 (t, *J* = 7.6 Hz, 1H, 13-H), 7.26 (t, *J* = 7.6 Hz, 1H, 1-H), 7.21 (d, *J* = 8.4 Hz, 2H, 11-H), 6.84 (d, *J* = 1.6 Hz, 1H, 8-H), 5.64 (s, 1H, 5-H), 2.96 (s, 1H, 6-H), 1.16 (s, 9H, 15-H); **^{13}C{^{1}H} NMR** (100 MHz, $CDCl_3$) 165.2 (s, C-9), 158.6 (s, C-7), 150.3 (s, C-10), 144.6 (s, C-4), 129.8 (d, C-12), 129.1 (d, C-8), 128.7 (d, C-2), 127.5 (d, C-1), 126.8 (d, C-13), 125.8 (d, C-3), 121.1 (d, C-11), 78.1 (d, C-5), 60.5 (d, C-6), 36.3 (s, C-14), 28.3 (q, C-15); **HRMS** (DART$^+$) Calculated ($C_{21}H_{23}O_3Cl_2Ge$): 467.0231 ($[M+H]^+$), Found: 467.0231 ($[M+H]^+$).

^{1}H NMR (400 MHz, $CDCl_3$)

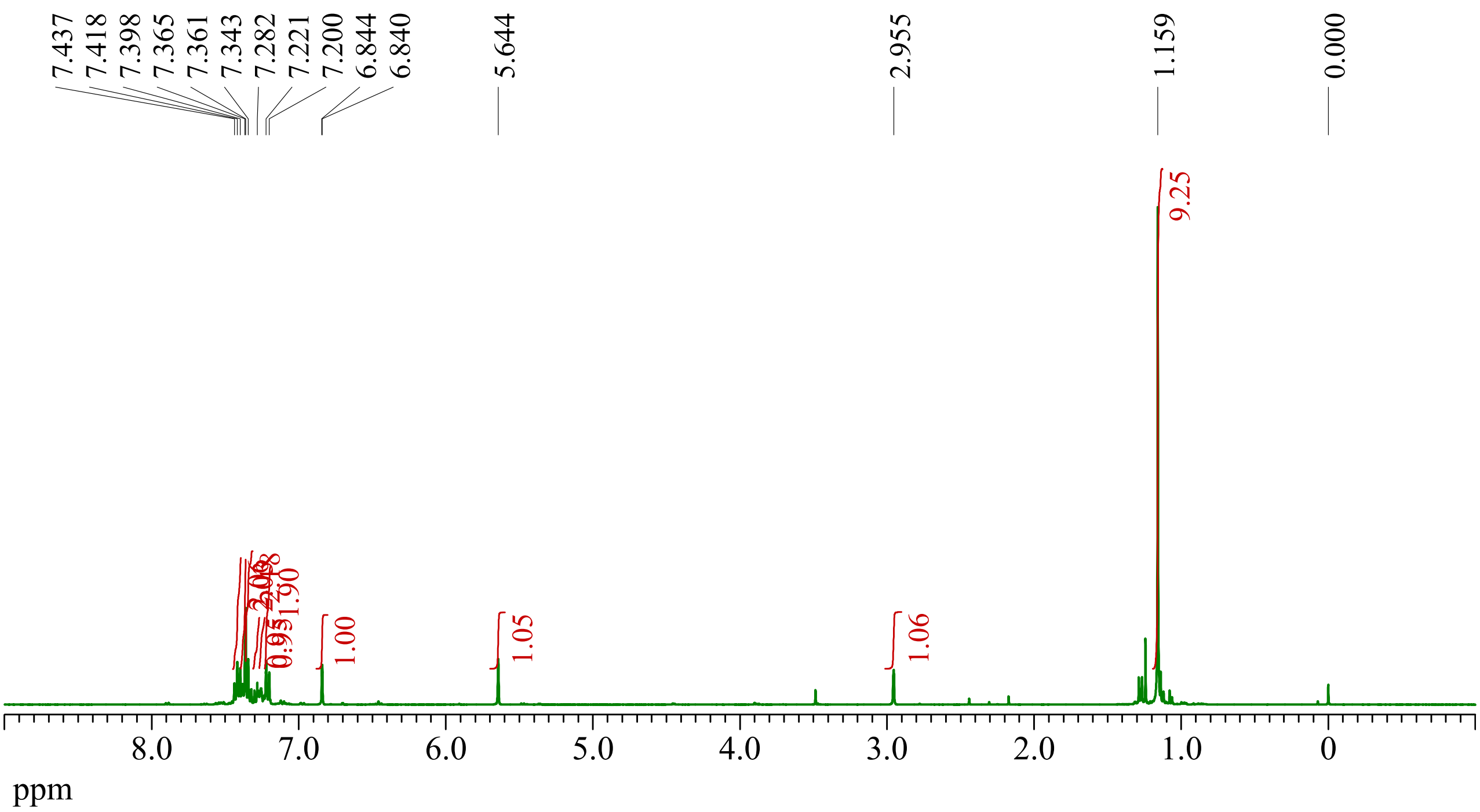

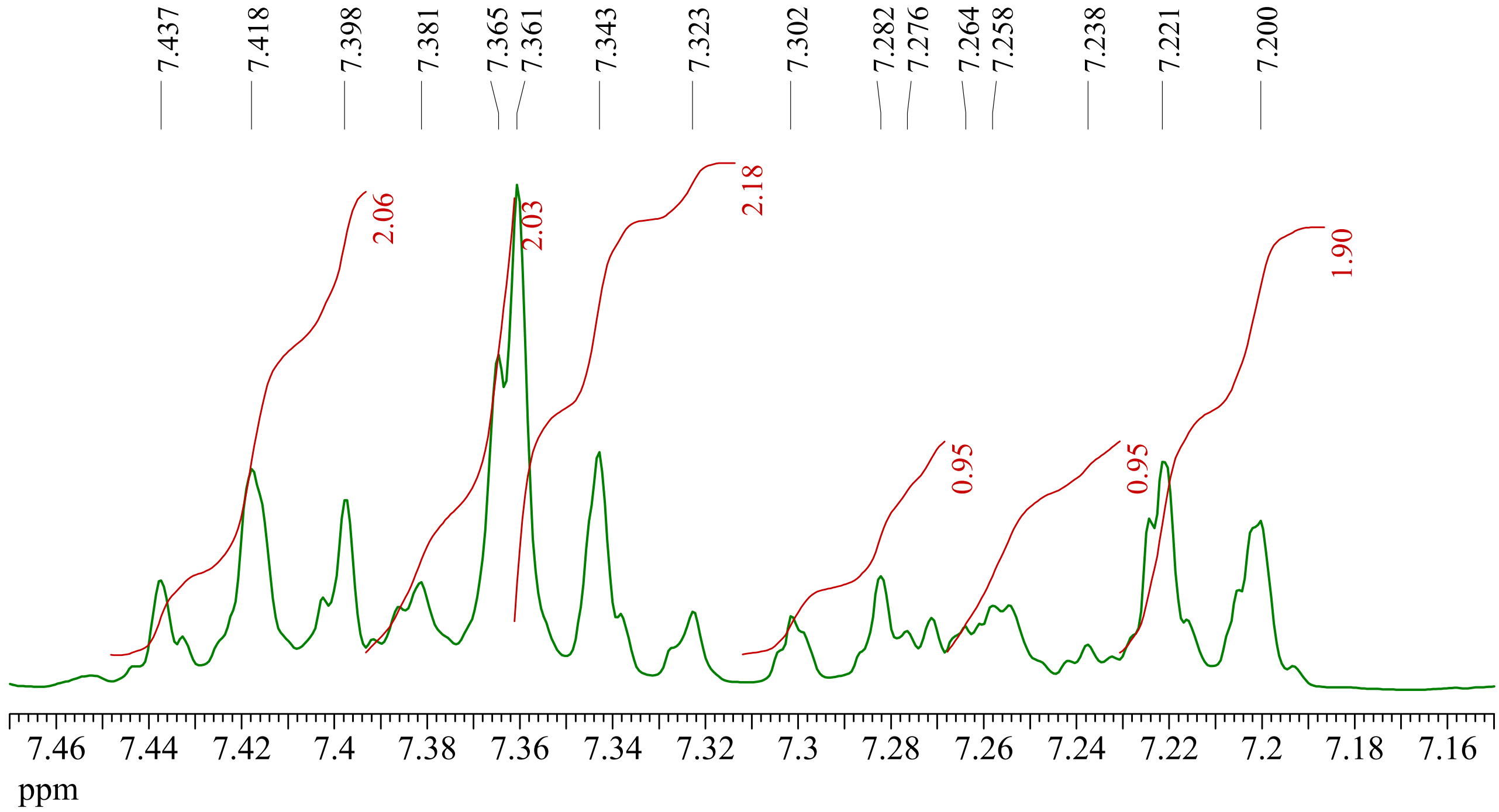

^{13}C{^{1}H} NMR (100 MHz, $CDCl_3$)

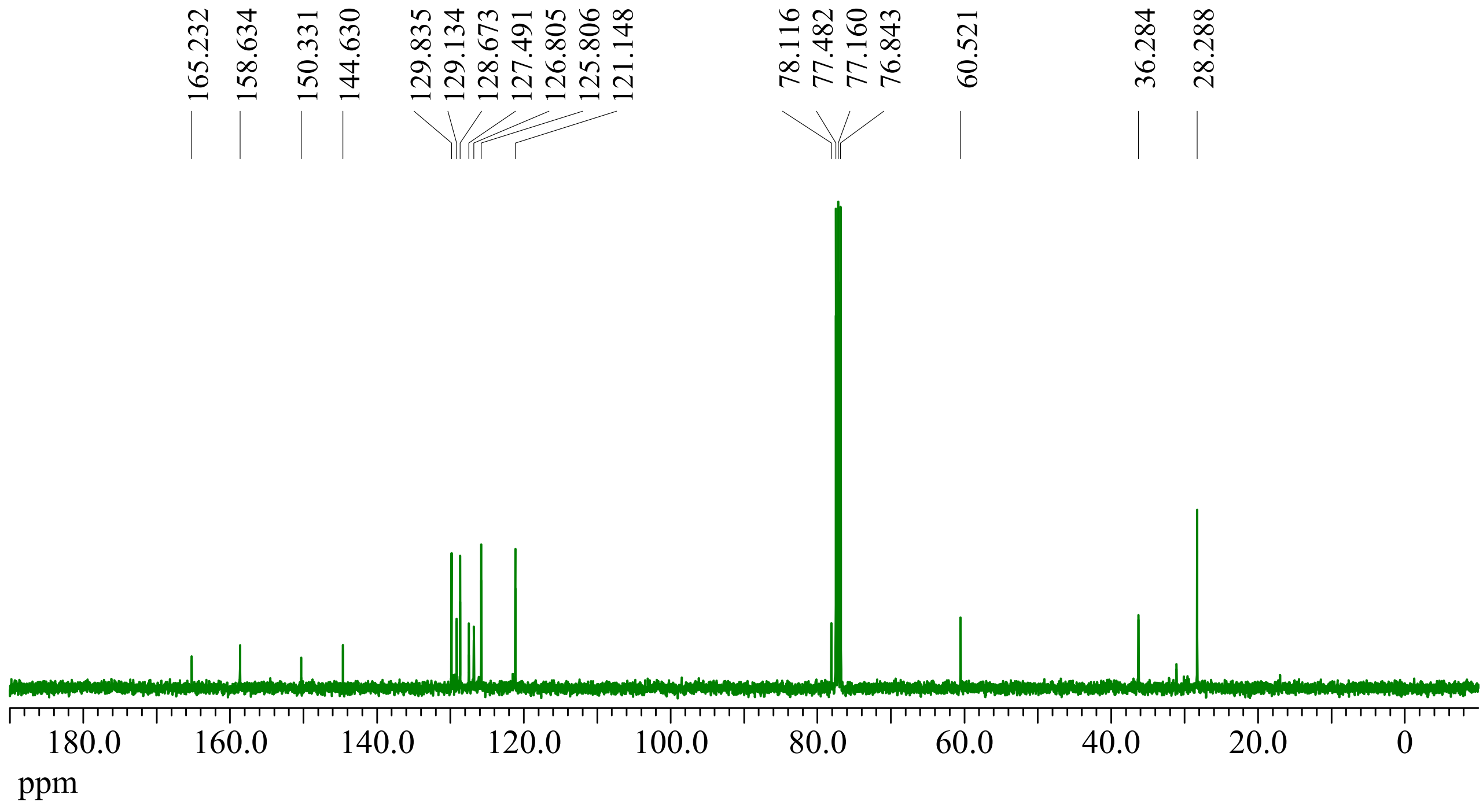

Ethyl (*Z*)-2-{(4*R,5*R**)-4-(*tert*-butyldimethylsilyl)-2,2-dichloro-5-phenyl-1,2-oxagermolan-3-ylidene}acetate *anti*-*γ*-4fa**

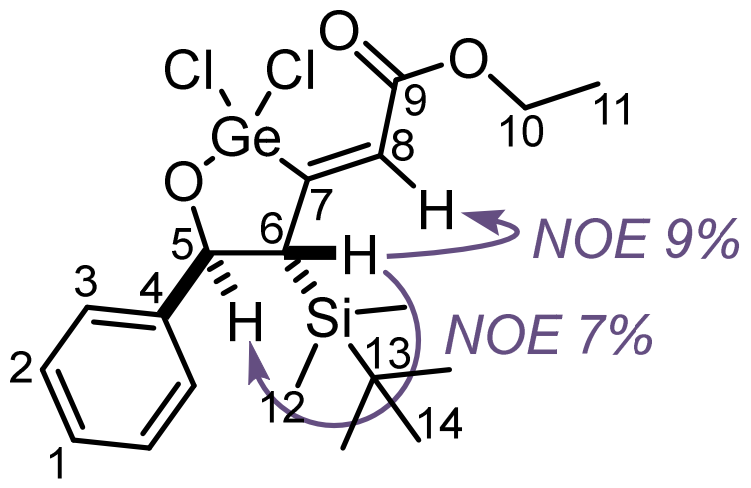

To a mixture of **1f** (0.0222 g, 0.0981 mmol) and benzaldehyde **3a** (0.0101 mL, 0.100 mmol) in hexane (0.50 mL) was added $GeCl_2$-dioxane (0.0114 g, 0.0492 mmol). After the reaction mixture was stirred at room temperature for 18 h, the solvent was removed under vacuum. The yield was determined by ^{1}H NMR of the crude product using 1,1,2,2-tetrachloroethane as an internal standard (54%, *anti*-*α*-**4fa**/*anti*-*γ*-**4fa** = 11/89, single diastereomer).

To obtain the spectrodata of the isolated product, careful extraction of the crude mixture with hexane gave *anti*-*γ*-**4fa** in pure form as pale yellow neat.

IR (ATR) 3031 (w), 2929 (m), 2858 (m), 1673 (m), 1468 (m), 1328 (s), 1256 (s), 1011 (s), 803 (s) cm^{-1}; **^{1}H NMR** (400 MHz, $CDCl_3$) 7.32–7.26 (m, 5H), 6.39 (d, *J* = 2.4 Hz, 1H, 8-H), 5.60 (s, 1H, 5-H), 4.44 (dq, *J* = 11.0, 7.4 Hz, 1H, 10-H), 4.38 (dq, *J* = 11.2, 7.2 Hz, 1H, 10-H), 3.15 (d, *J* = 2.0 Hz, 1H, 6-H), 1.38 (t, *J* = 7.2 Hz, 3H, 11-H), 1.00 (s, 9H, 14-H), 0.27 (s, 3H, 12-H), 0.06 (s, 3H, 12-H); **^{13}C{^{1}H} NMR** (100 MHz, $CDCl_3$) 168.5 (s, C-9), 160.2 (s, C-7), 145.0 (s, C-4), 128.7 (d, C-2), 127.7 (d, C-1), 126.3 (d, C-3), 119.7 (d, C-8), 77.4 (d, C-5), 63.5 (t, C-10), 41.4 (d, C-6), 27.2 (q, C-14), 18.6 (s, C-13), 14.3 (q, C-11), –5.6 (q, C-12), –6.6 (q, C-12); **^{29}Si{^{1}H} NMR** (78.5 MHz, $CDCl_3$, Me_4Si in $CDCl_3$ was employed as an external standard (0.00 ppm).) 13.0; **HRMS** (DART$^+$) Calculated ($C_{19}H_{29}O_3SiCl_2Ge$): 477.0469 ([M+H]$^+$), Found: 451.0465 ([M+H]$^+$).

^{1}H NMR (400 MHz, $CDCl_3$)

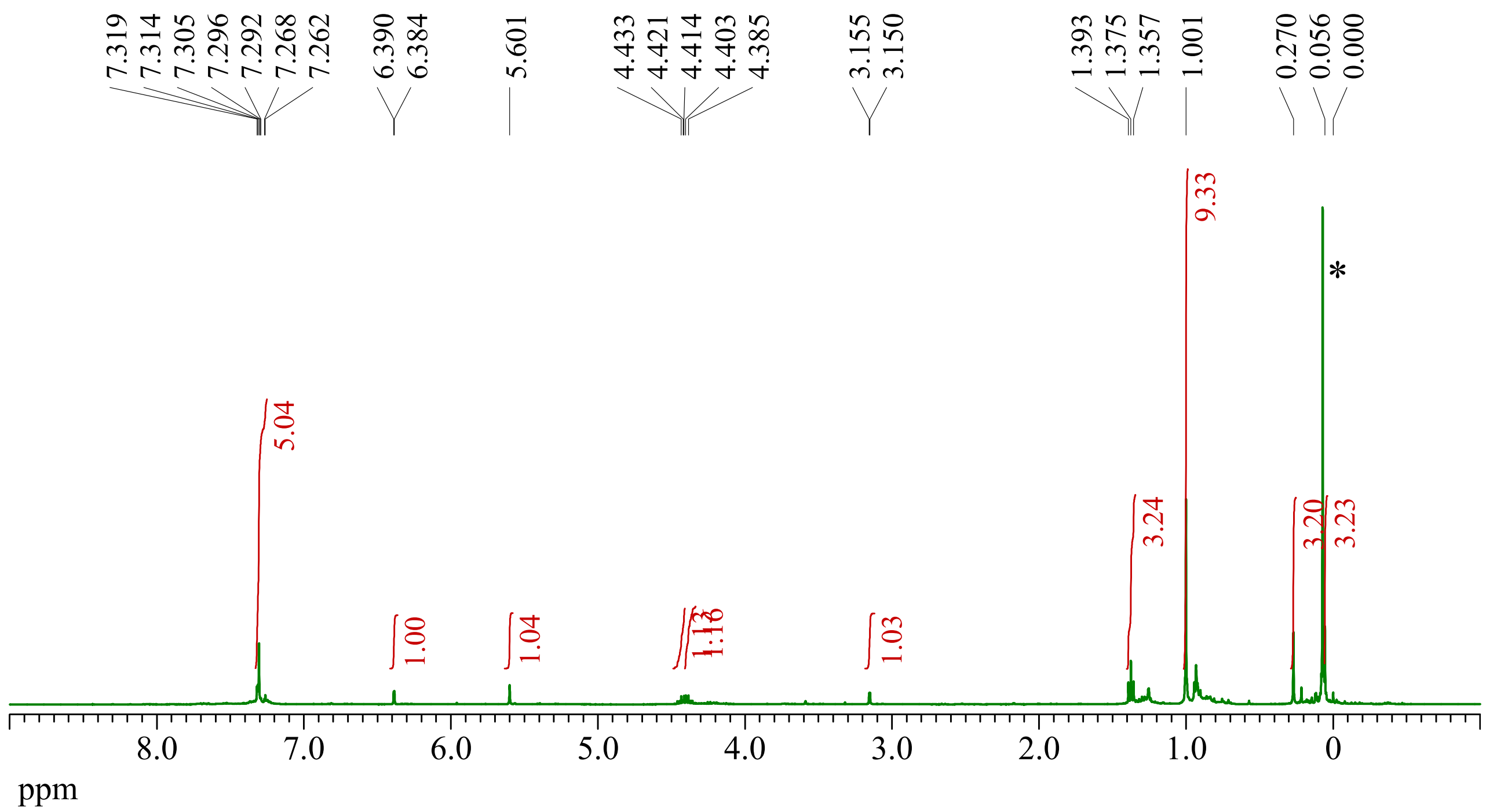

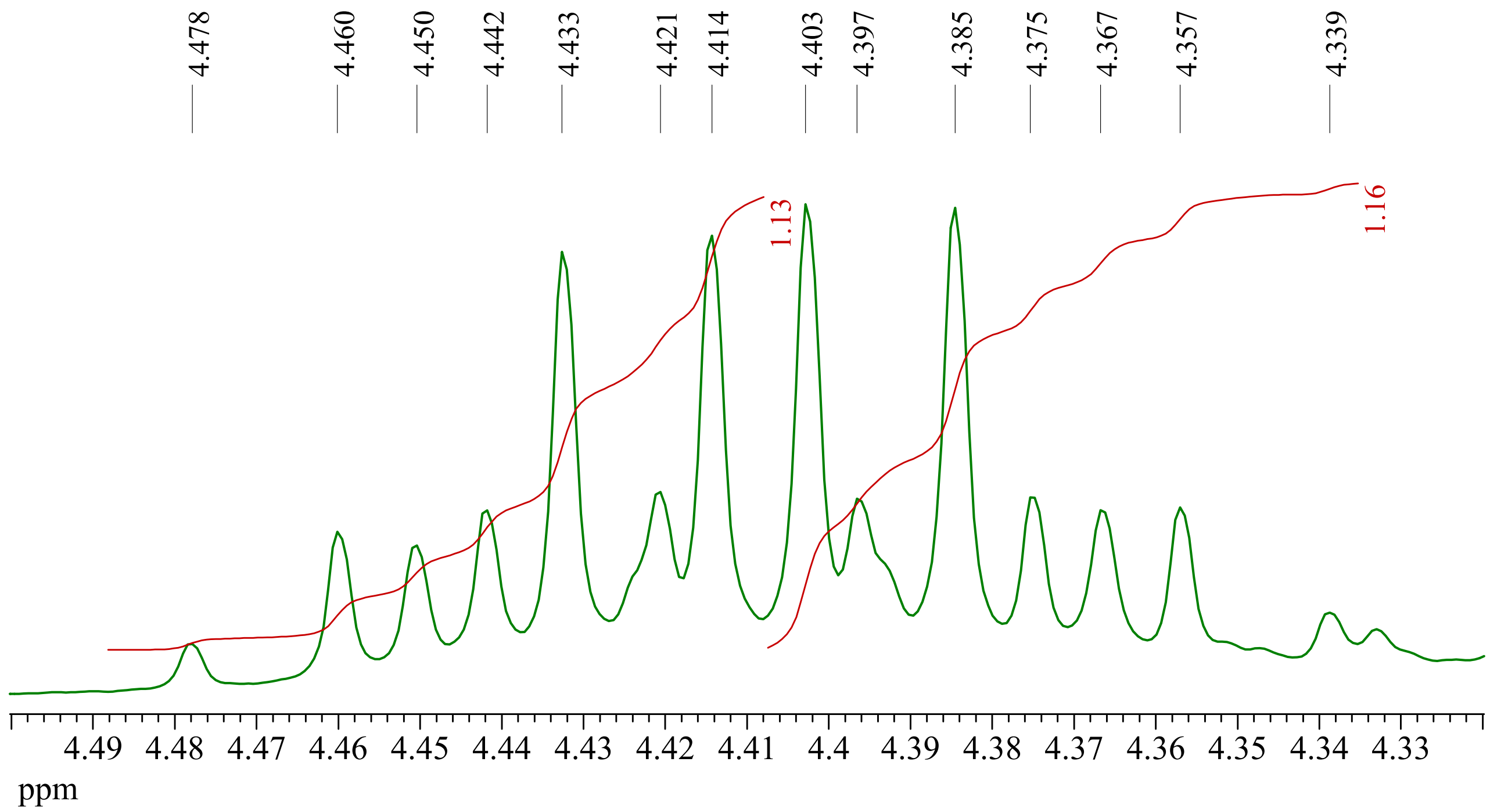

$^{13}C\{^1H\}$ NMR (100 MHz, $CDCl_3$)

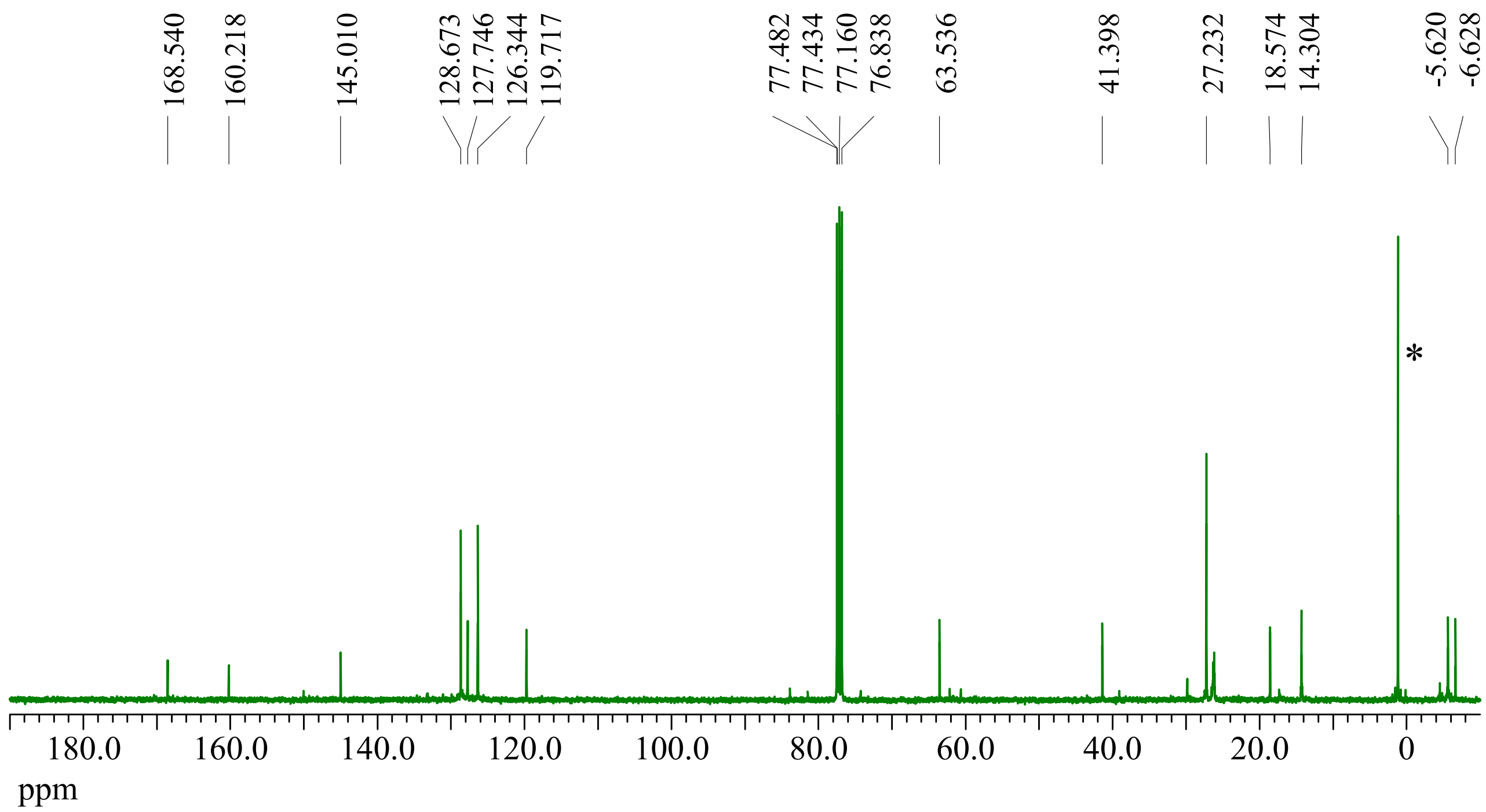

$^{29}Si\{^1H\}$ NMR (78.5 MHz, $CDCl_3$, Me_4Si in $CDCl_3$ was employed as an external standard (0.00 ppm).)

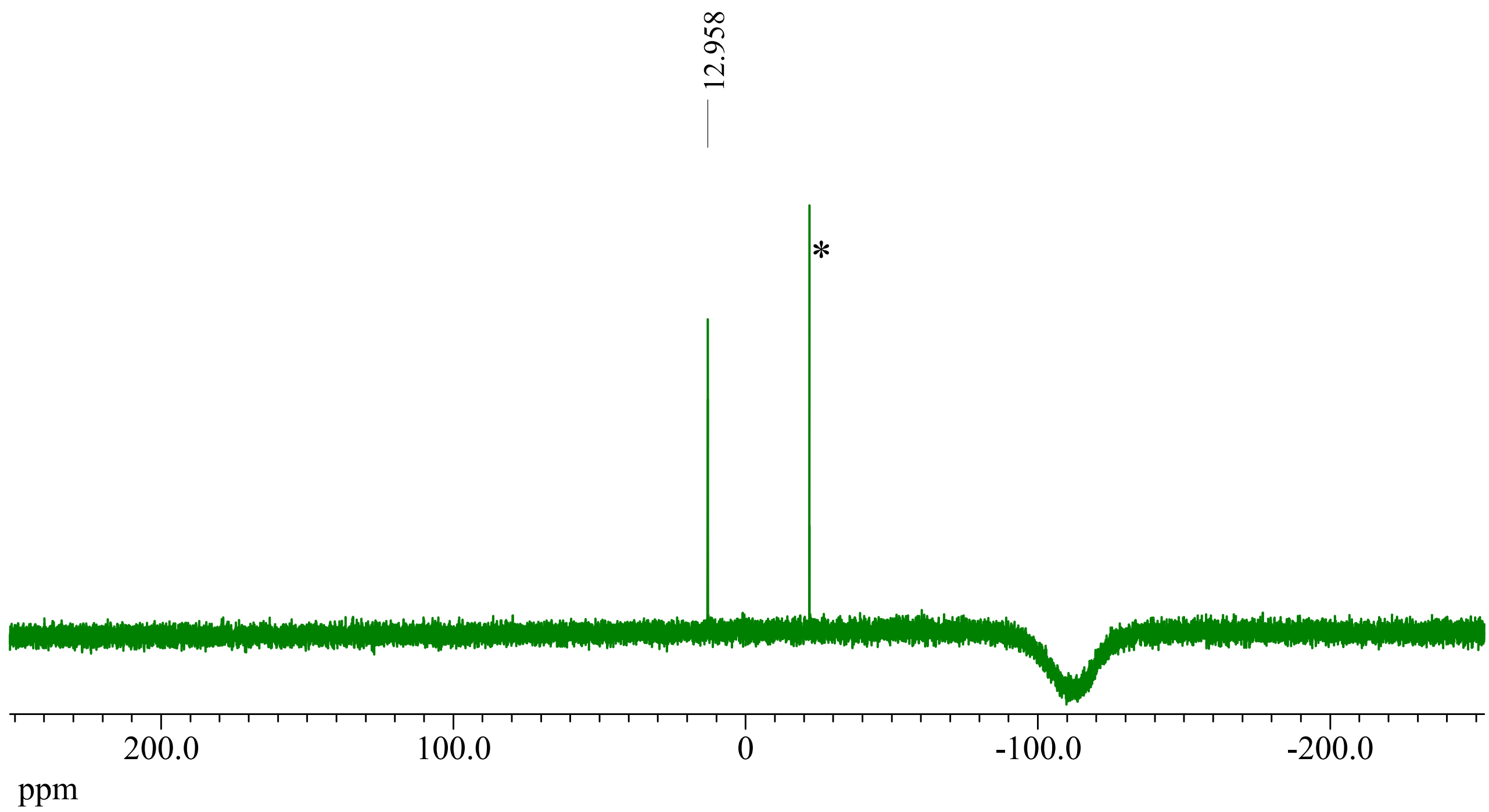

Asterisk marks (*) represent the signals of the inseparatable impurity.

3-8. Derivatizations of *anti-α*-4aa

Ethyl (*R,*E*)-2-{(*S**)-hydroxy(phenyl)methyl}-5,5-dimethyl-3-(trimethylgermyl)hex-3-enoate** *anti-α*-**5**

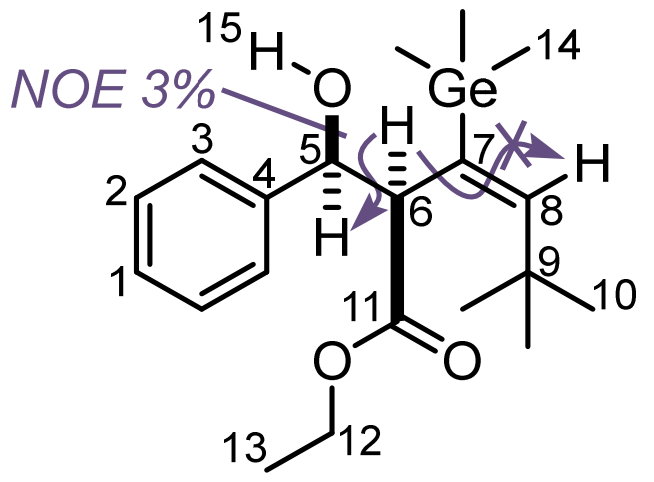

To a mixture of *anti-α*-**4aa** (0.210 mmol) in THF (2.1 mL) was added MeMgBr (0.280 mL, 3 M in Et_2O, 0.840 mmol) at 0 °C. The reaction mixture was stirred for 3 h at room temperature and quenched with NH_4Cl aq. The organic phase was extracted with Et_2O and dried over Na_2SO_4. The filtrate was evaporated and the residue was purified by column chromatography on SiO_2 (Hexane/EtOAc = 85:15) to give *anti-α*-**5** as a colorless solid of the single diastereomer (0.0625 g, 76%).

mp 59.0–60.0 °C; **IR** (ATR) 3483 (m), 3033 (w), 2960 (s), 1731 (s), 1454 (w), 1299 (m), 1176 (m), 1038 (m), 827 (m) cm^{-1}; **^{1}H NMR** (400 MHz, $CDCl_3$) 7.33‑7.20 (m, 5H), 5.50 (s, 1H, 8-H), 5.10 (dd, *J* = 9.2, 2.0 Hz, 1H, 5-H), 4.34 (d, *J* = 9.2 Hz, 1H, 6-H), 4.19 (q, *J* = 7.2 Hz, 2H, 12-H), 3.62 (d, *J* = 2.8 Hz, 1H, 15-H), 1.27 (t, *J* = 7.2 Hz, 3H, 13-H), 0.80 (s, 9H, 10-H), 0.30 (s, 9H, 14-H); **^{13}C{^{1}H} NMR** (100 MHz, $CDCl_3$) 174.6 (s, C-11), 152.5 (d, C-8), 141.5 (s, C-4), 134.8 (s, C-7), 128.0 (d, C-2), 127.8 (d, C-1), 127.0 (d, C-3), 73.9 (d, C-5), 61.1 (t, C-12), 55.5 (d, C-6), 35.5 (s, C-9), 31.0 (q, C-10), 14.2 (q, C-13), 1.47 (q, C-14); **HRMS** (ESI$^+$) Calculated ($C_{20}H_{32}O_3NaGe$): 417.1455 ([M+Na]$^+$), Found: 417.1448 ([M+Na]$^+$).

^{1}H NMR (400 MHz, $CDCl_3$)

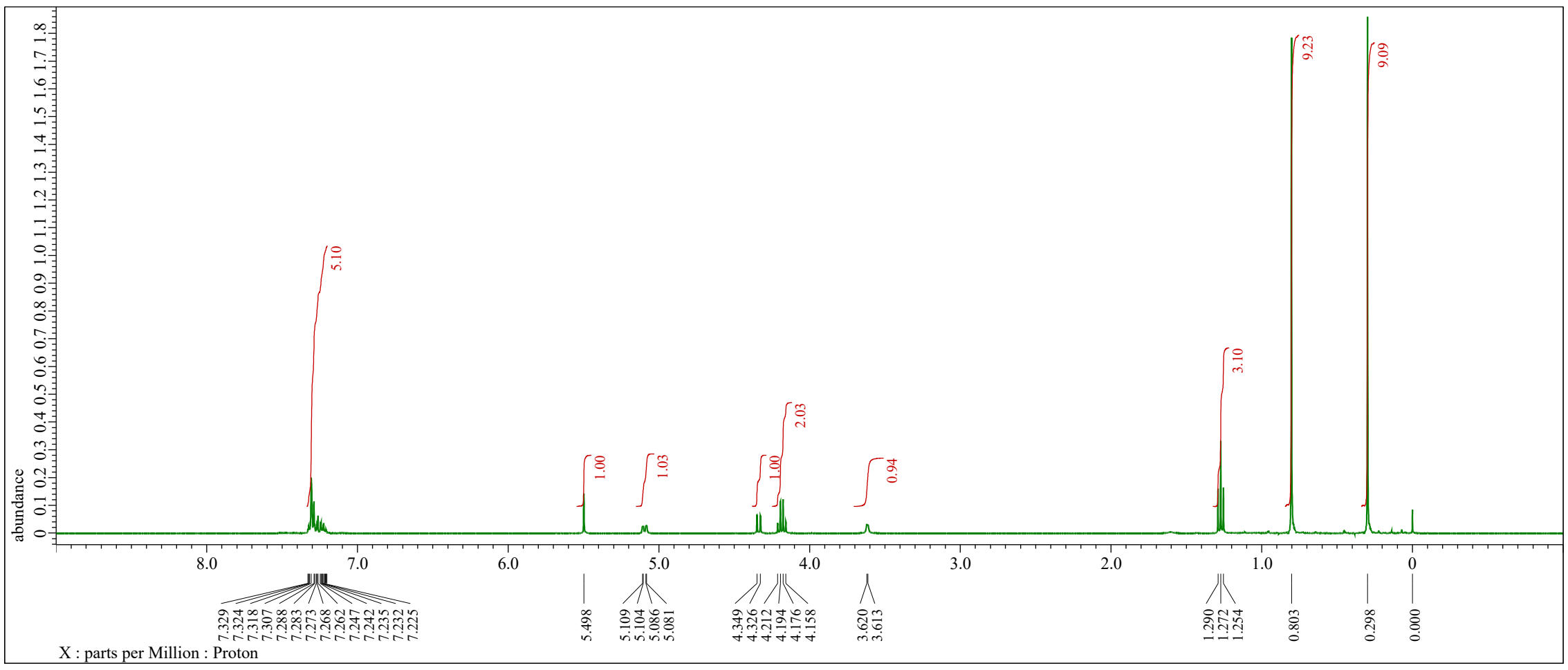

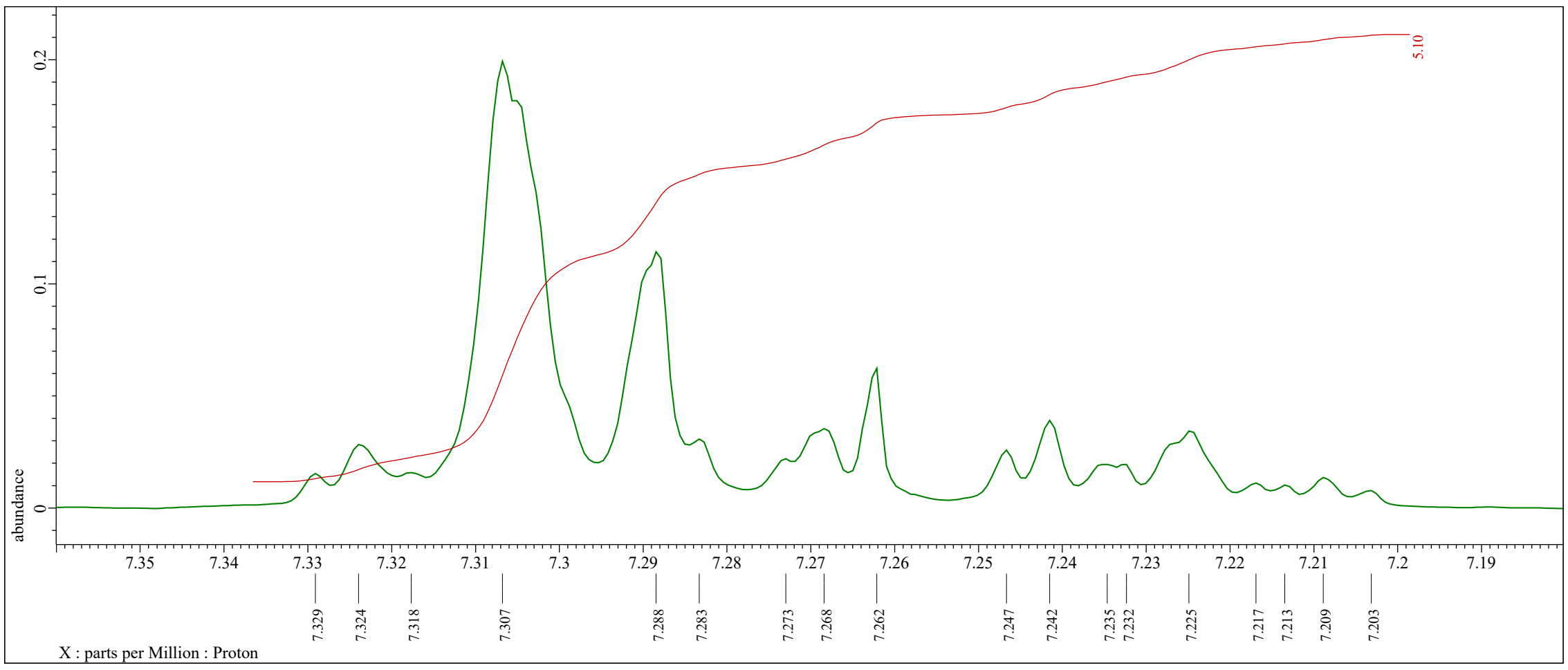

^{13}C{^{1}H} NMR (100 MHz, $CDCl_3$)

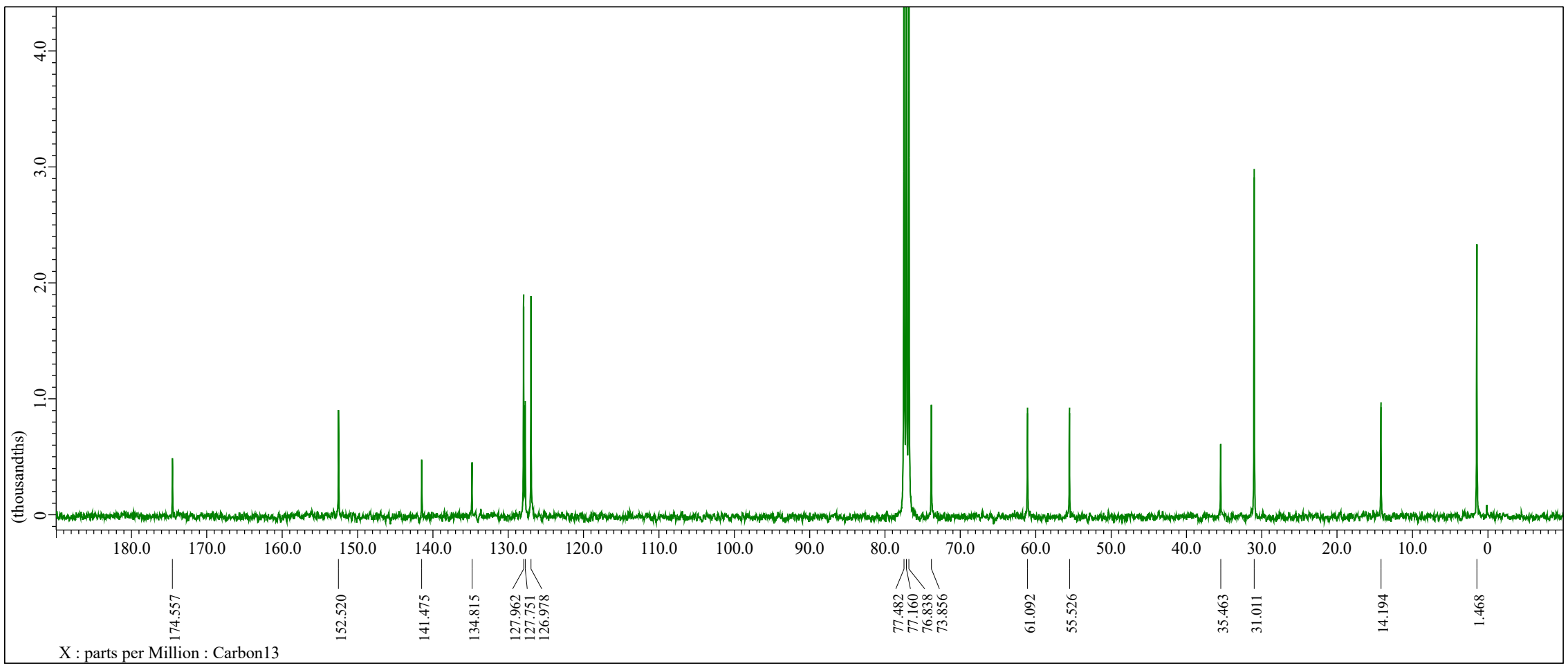

(1*R,2*S**)-2-{(*E*)-3,3-Dimethyl-1-(trimethylgermyl)but-1-en-1-yl}-3-methyl-1-phenylbutane-1,3-diol** *anti-α*-**6**

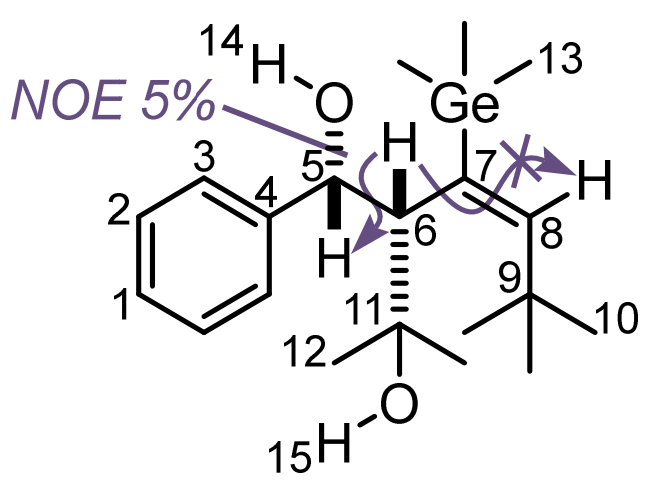

To a mixture of *anti*-*α*-**4aa** (0.169 mmol) in THF (1.7 mL) was added MeMgBr (0.563 mL, 3 M in Et_2O, 1.69 mmol) at 0 °C. The reaction mixture was stirred for 16 h at room temperature and quenched with NH_4Cl aq. The organic phase was extracted with Et_2O and dried over Na_2SO_4. The filtrate was evaporated and the residue was purified by column chromatography on SiO_2 (Hexane/EtOAc = 70:30) to give *anti*-*α*-**6** as a colorless solid of the single diastereomer (0.0531 g, 83%).

mp 154.7–155.5 °C; **IR** (ATR) 3206 (s), 3032 (m), 2954 (s), 1453 (w), 1382 (w), 1236 (w), 1164 (w), 1012 (w), 823 (m) cm^{-1}; **^{1}H NMR** (400 MHz, $CDCl_3$) 7.38–7.28 (m, 4H), 7.25–7.20 (m, 1H, 1-H), 5.24 (s, 1H, 8-H), 5.06 (d,

J = 10.0 Hz, 1H, 5-H), 3.72 (d, *J* = 10.0 Hz, 1H, 6-H), 1.47 (s, 3H, 12-H), 1.26 (s, 3H, 12-H), 0.79 (s, 9H, 10-H), 0.34 (s, 9H, 13-H); **^{13}C{^{1}H} NMR** (100 MHz, $CDCl_3$) 151.9 (d, C-8), 144.2 (s, C-4), 136.4 (s, C-7), 128.1 (d, C-2), 127.8 (d, C-1), 127.3 (d, C-3), 76.4 (d, C-5), 74.9 (s, C-11), 56.5 (d, C-6), 36.8 (s, C-9), 31.3 (q, C-12), 31.0 (q, C-10), 27.3 (q, C-12), 3.4 (q, C-13); **HRMS** (ESI$^+$) Calculated ($C_{20}H_{34}O_2NaGe$): 403.1663 ([M+Na]$^+$), Found: 403.1661 ([M+Na]$^+$).

^{1}H NMR (400 MHz, $CDCl_3$)

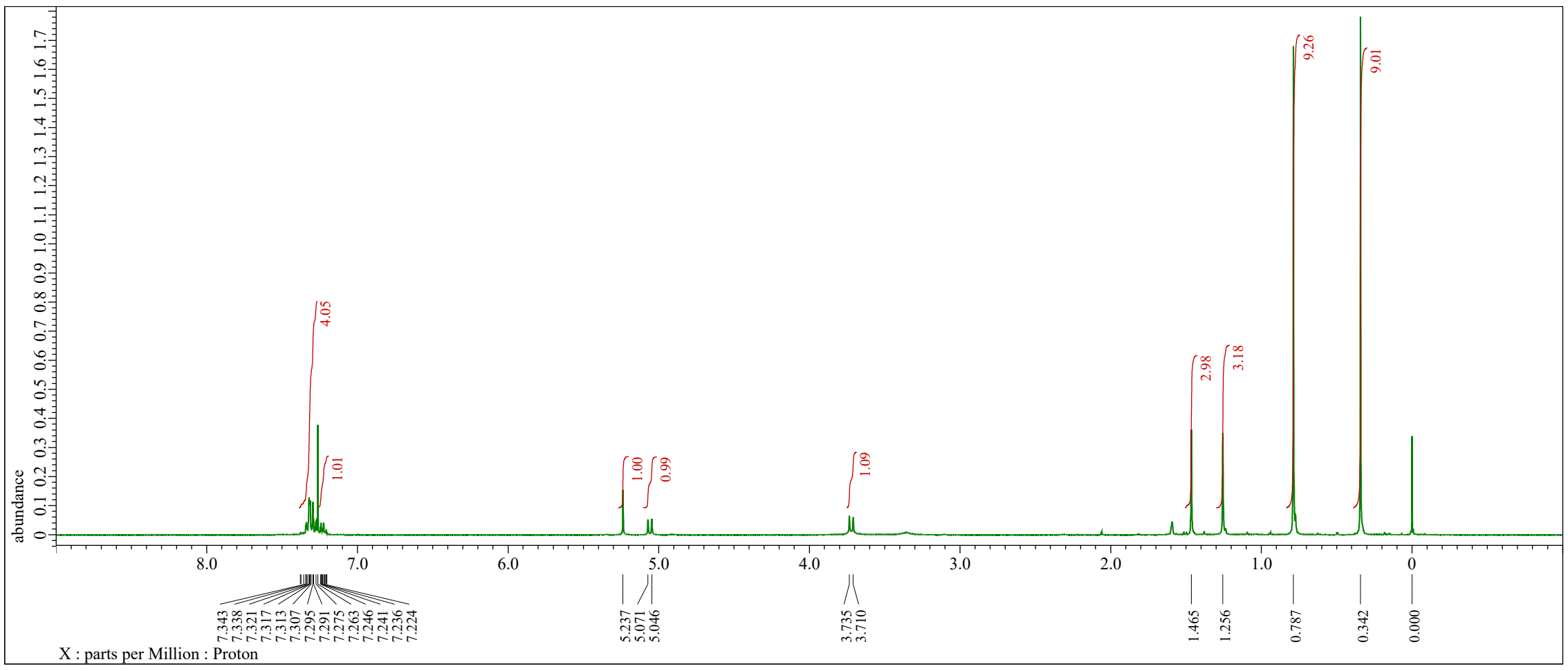

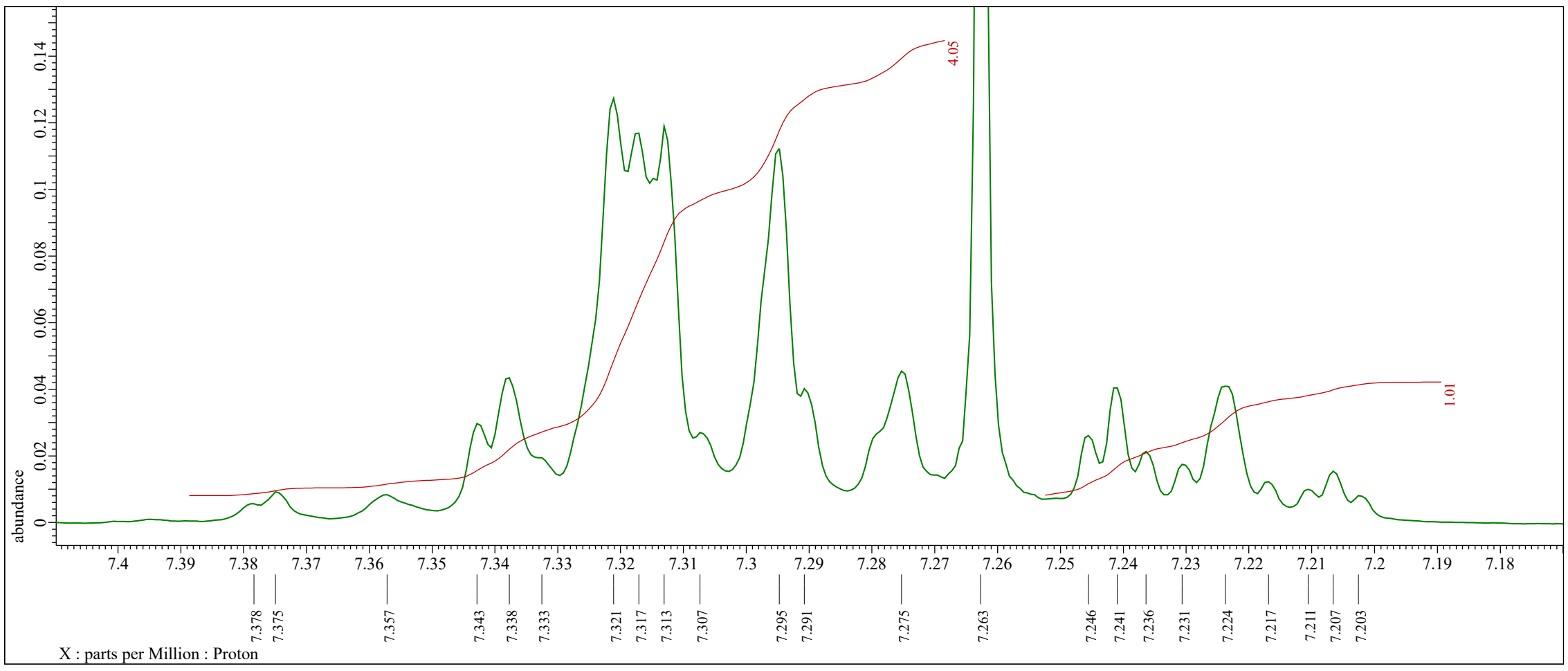

^{13}C{^{1}H} NMR (100 MHz, $CDCl_3$)

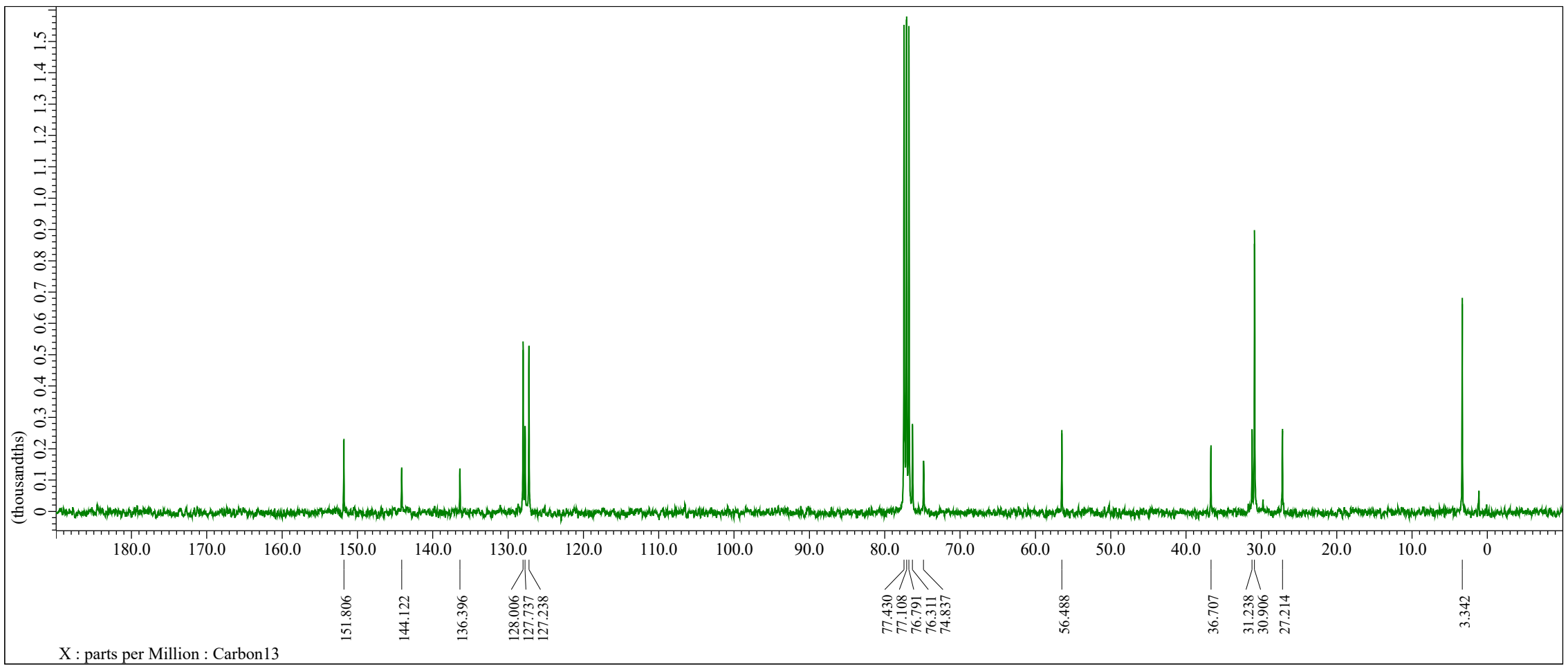

Ethyl (2*R,3*S**,*E*)-1-(2,2-dimethylpropylidene)-3-hydroxy-2,3-dihydro-1*H*-indene-2-carboxylate 7**

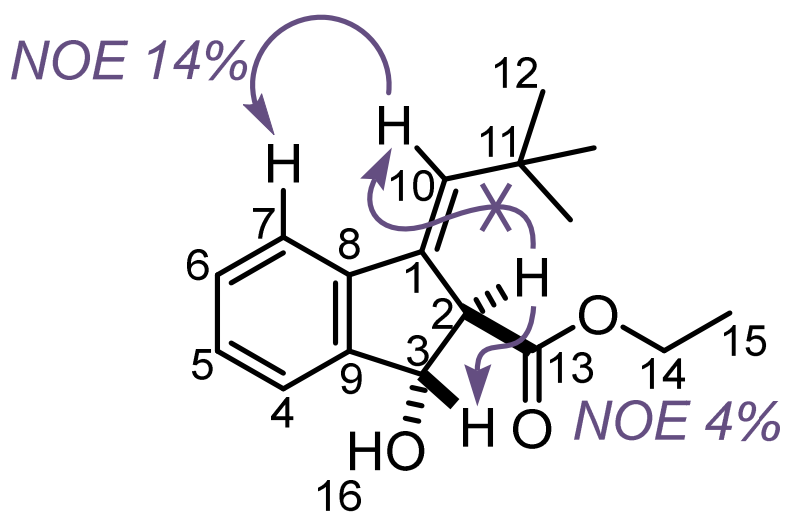

In a nitrogen filled glovebox, *anti*-*α*-**5** (0.0427 g, 0.109 mmol), $(Ph_3P)AuCl$ (2.47 mg, 5.00 μmol) (+)-10-camphorsulfonic acid (0.0348 g, 0.150 mmol) and $PhI(OAc)_2$ (0.0483 g, 0.0150 mmol) were dissolved in 1,4-dioxane (1.0 mL). After the reaction mixture was stirred at 70 °C for 18 h, the solvent was removed under vacuum and the residue was purified by column chromatography on SiO_2 (Hexane/EtOAc = 80:20) to give **7** as colorless neat of the single diastereomer (0.0159 g, 39%).

IR (ATR) 3426 (m), 3027 (w), 2957 (s), 1724 (s), 1469 (m), 1365 (m), 1227 (s), 1153 (s), 1028 (s) cm^{-1}; **^{1}H NMR** (400 MHz, $CDCl_3$) 7.51 (d, *J* = 7.6 Hz, 1H, 7-H), 7.42 (d, *J* = 7.6 Hz, 1H, 4-H), 7.33 (t, *J* = 7.6 Hz, 1H, 6-H), 7.26 (t, *J* = 7.4 Hz, 1H, 5-H), 6.24 (d, *J* = 1.2 Hz, 1H, 10-H), 5.24 (d, *J* = 4.0 Hz, 1H, 3-H), 4.14 (q, *J* = 7.2 Hz, 2H, 14-H), 4.07 (s, 1H, 2-H), 1.99 (d, *J* = 4.0 Hz, 1H, 16-H), 1.24 (t, *J* = 7.0 Hz, 3H, 15-H), 1.20 (s, 9H, 12-H); **^{13}C{^{1}H} NMR** (100 MHz, $CDCl_3$) 172.7 (s, C-13), 142.9 (s, C-8), 142.7 (s, C-9), 136.3 (d, C-10), 133.1 (s, C-1), 129.7 (d, C-6), 128.4 (d, C-5), 125.5 (d, C-4), 120.4 (d, C-7), 78.4 (d, C-3), 61.2 (t, C-14), 56.8 (d, C-2), 34.0 (s, C-11), 30.4 (q, C-12), 14.3 (q, C-15); **HRMS** (EI^+, 70 eV) Calculated ($C_{17}H_{22}O_3$): 274.1569 (M^+), Found: 274.1561 (M^+).

^{1}H NMR (400 MHz, $CDCl_3$)

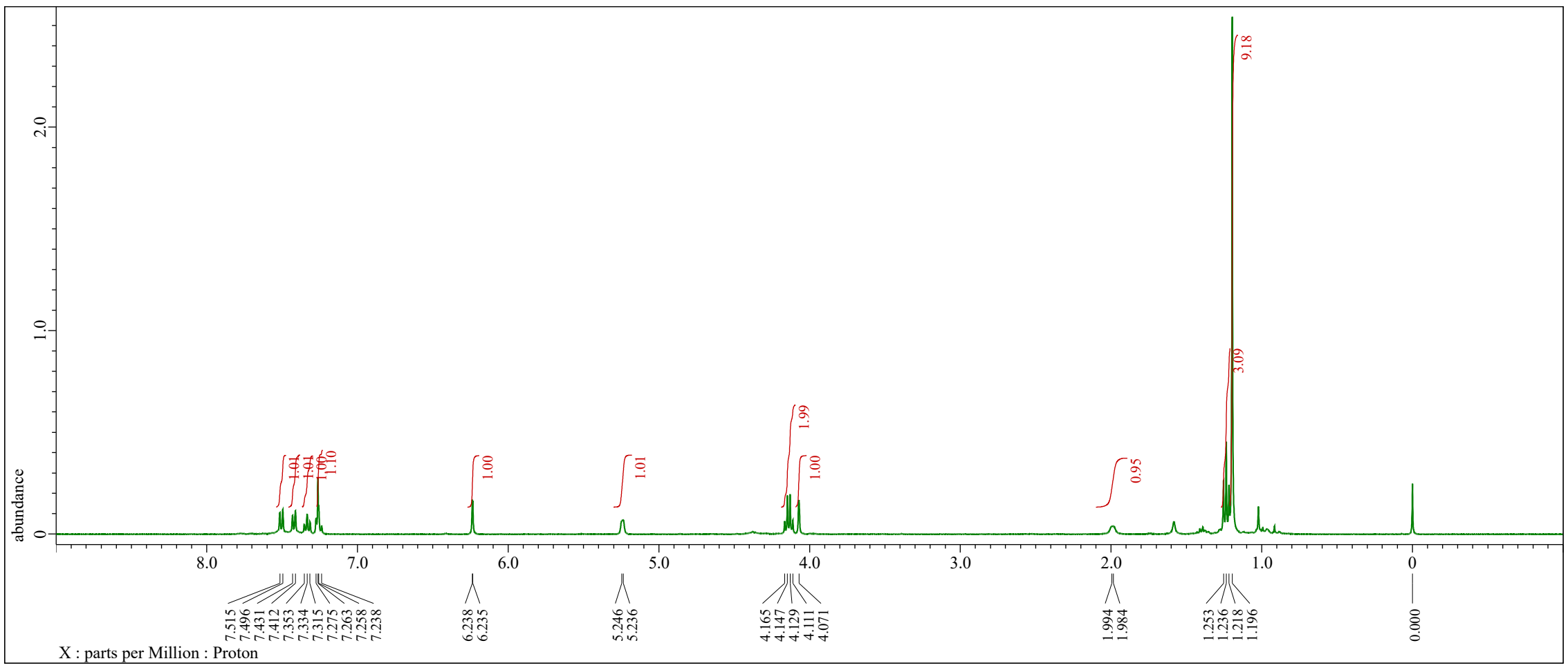

^{13}C{^{1}H} NMR (100 MHz, $CDCl_3$)

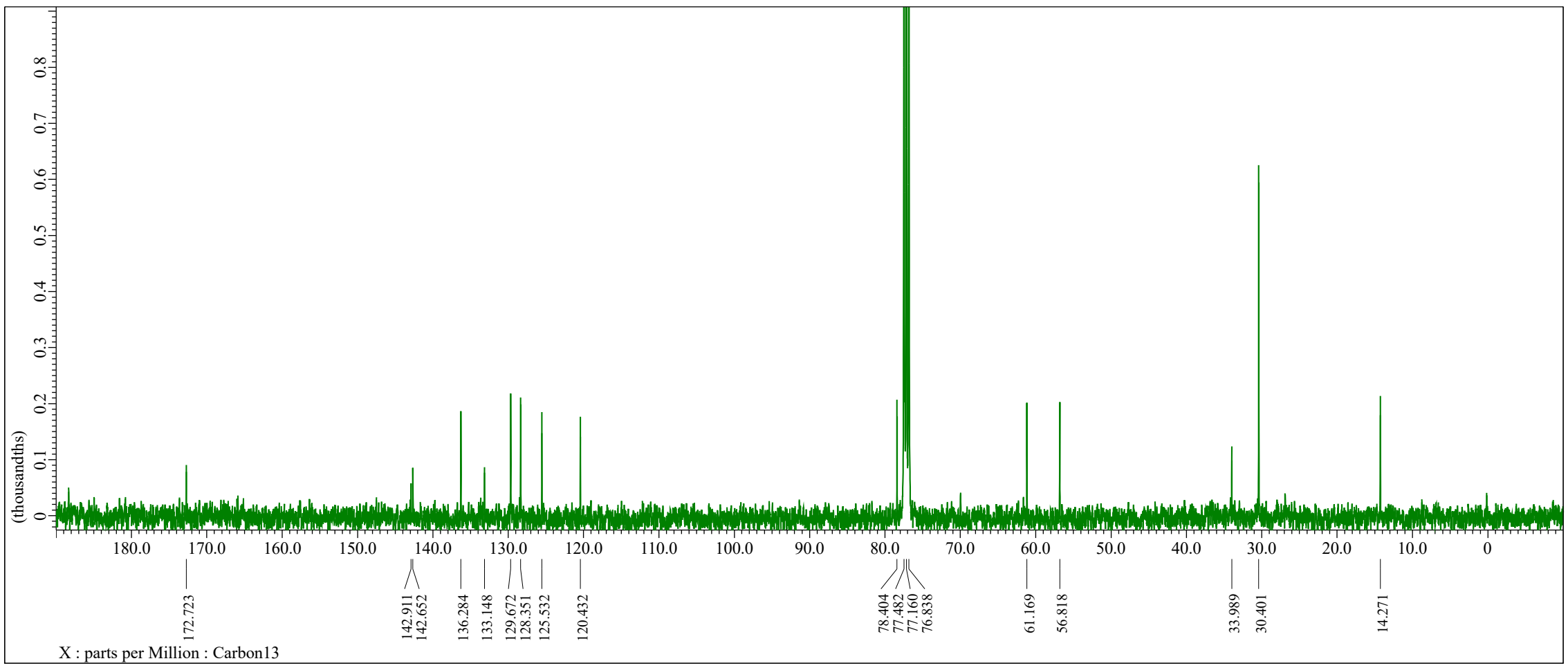

(*E*)-1-(2,2-Dimethylpropylidene)-1*H*-indene 8

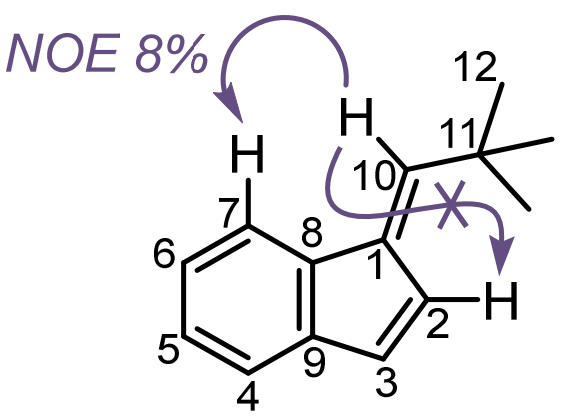

In a nitrogen filled glovebox, *anti*-*α*-**6** (0.0379 g, 0.100 mmol), $(Ph_3P)AuCl$ (2.47 mg, 5.00 μmol) (+)-10-camphorsulfonic acid (0.0348 g, 0.150 mmol) and $PhI(OAc)_2$ (0.0483 g, 0.0150 mmol) were dissolved in 1,4-dioxane (1.0 mL). After the reaction mixture was stirred at 70 °C for 5 h, the solvent was removed under vacuum and the residue was purified by column chromatography on SiO_2 (Hexane/EtOAc = 90:10) to give **8** as colorless neat (0.0127 g, 69%).

IR (ATR) 3070 (w), 2958 (s), 1637 (m), 1453 (s), 1362 (m), 1335 (m), 1263 (m), 1175 (w), 1017 (w), 917 (w) cm^{-1}; **^{1}H NMR** (400 MHz, $CDCl_3$) 7.57 (d, *J* = 6.8 Hz, 1H, 7-H), 7.29 (d, *J* = 6.8 Hz, 1H, 4-H), 7.22 (t, *J* = 7.0 Hz, 1H,

5-H), 7.18 (t, J = 7.4 Hz, 1H, 6-H), 7.00 (d, J = 5.6 Hz, 1H, 2-H), 6.93 (d, J = 5.6 Hz, 1H, 3-H), 6.71 (s, 1H, 10-H), 1.34 (s, 9H, 12-H); **^{13}C{^{1}H} NMR** (100 MHz, $CDCl_3$) 143.1 (d, C-10), 141.2 (s, C-9), 138.2 (s, C-8), 137.0 (s, C-1), 132.7 (d, C-3), 127.0 (d, C-5), 125.9 (d, C-2), 124.9 (d, C-6), 120.7 (d, C-4), 118.7 (d, C-7), 35.2 (s, C-11), 31.6 (q, C-12); **HRMS** (EI$^+$, 70 eV) Calculated ($C_{14}H_{16}$): 184.1252 (M$^+$), Found: 184.1250 (M$^+$)

^{1}H NMR (400 MHz, $CDCl_3$)

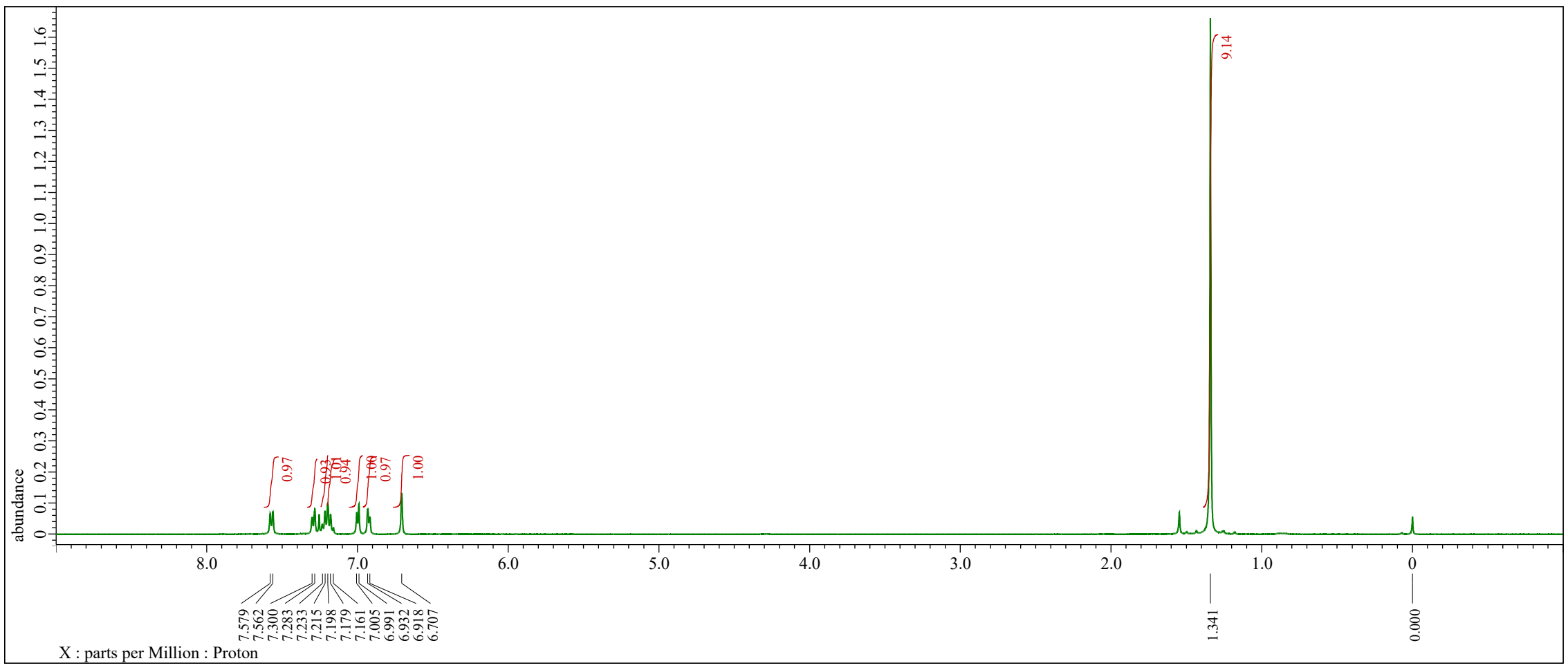

^{13}C{^{1}H} NMR (100 MHz, $CDCl_3$)

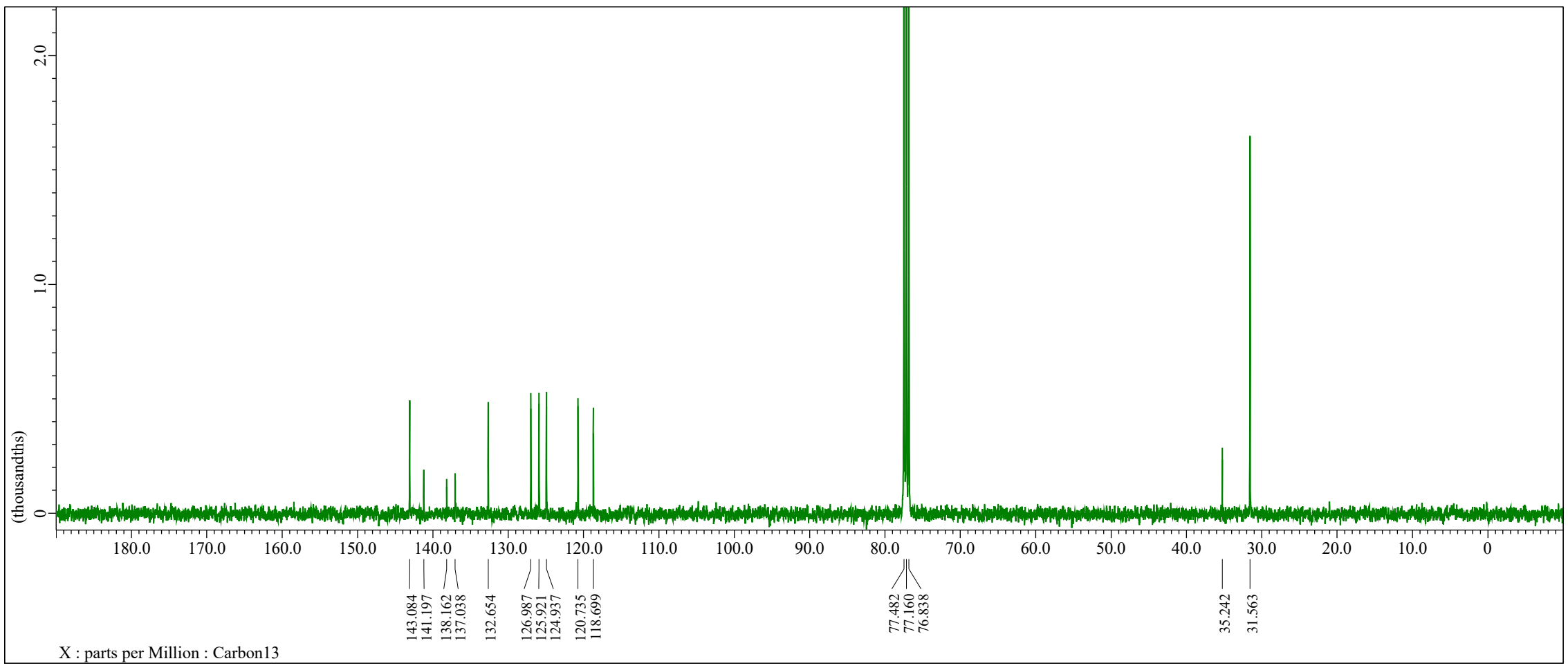

3-9. Derivatizations of *anti-γ*-4aa

Ethyl (*R,*Z*)-4-{(*S**)-hydroxy(phenyl)methyl}-5,5-dimethyl-3-(trimethylgermyl)hex-2-enoate** *anti-γ*-**5**

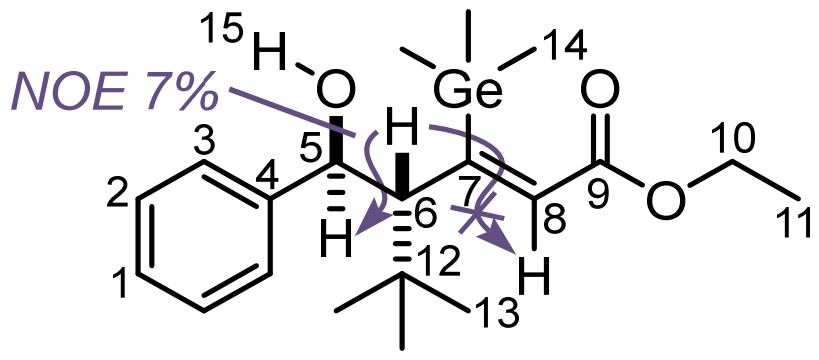

To a mixture of *anti-γ*-**4aa** (0.398 mmol) in THF (4.0 mL) was added MeMgBr (0.531 mL, 3 M in Et_2O, 1.59 mmol) at 0 °C. The reaction mixture was stirred for 3 h at 0 °C and quenched with NH_4Cl aq. The organic phase was extracted with Et_2O and dried over Na_2SO_4. The filtrate was evaporated and the residue was purified by column chromatography on SiO_2 (Hexane/EtOAc = 85:15) to give *anti-γ*-**5** as a colorless solid of the single diastereomer (0.116 g, 74%).

mp 63.0–64.0 °C; **IR** (ATR) 3507 (m), 3027 (w), 2959 (s), 1718 (s), 1595 (m), 1371 (m), 1194 (s), 1052 (m), 831 (m) cm^{-1}; **^{1}H NMR** (400 MHz, $CDCl_3$) 7.31–7.27 (m, 2H, 2-H), 7.22–7.18 (m, 3H), 7.02 (s, 1H, 8-H), 5.22 (s, 1H, 5-H), 4.21 (dq, *J* = 11.0, 7.0 Hz, 1H, 10-H), 4.17 (dq, *J* = 11.0, 7.0 Hz, 1H, 10-H), 2.79 (d, *J* = 3.6 Hz, 1H, 6-H), 1.86 (s, 1H, 15-H), 1.33 (t, *J* = 7.2 Hz, 3H, 11-H), 1.05 (s, 9H, 13-H), –0.04 (s, 9H, 14-H); **^{13}C{^{1}H} NMR** (100 MHz, $CDCl_3$) 169.0 (s, C-7), 166.6 (s, C-9), 145.0 (s, C-4), 131.3 (d, C-8), 128.3 (d, C-2), 127.3 (d, C-1), 126.4 (d, C-3), 74.1 (d, C-5), 60.2 (t, C-10), 59.0 (d, C-6), 35.9 (s, C-12), 29.5 (q, C-13), 14.5 (q, C-11), 0.03 (q, C-14); **HRMS** (ESI$^+$) Calculated ($C_{20}H_{32}O_3NaGe$): 417.1455 ([M+Na]$^+$), Found: 417.1450 ([M+Na]$^+$).

^{1}H NMR (400 MHz, $CDCl_3$)

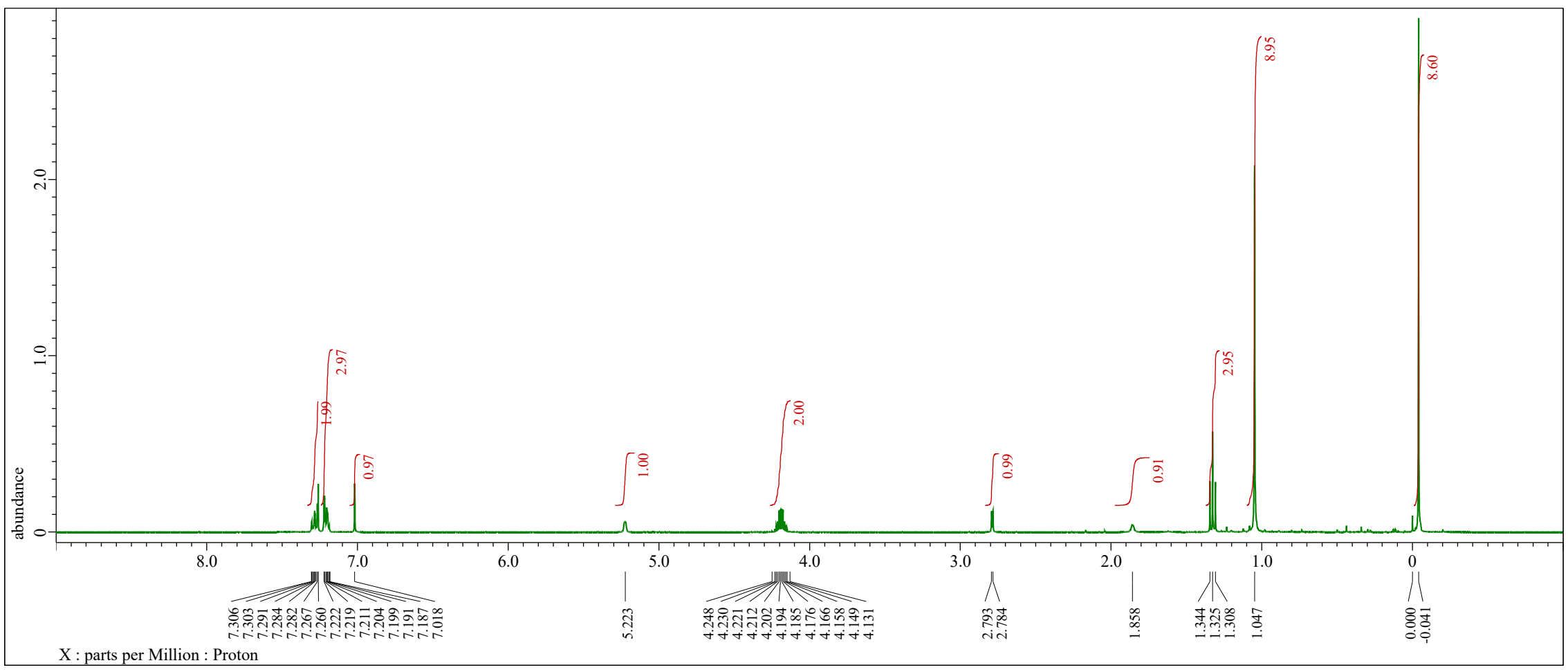

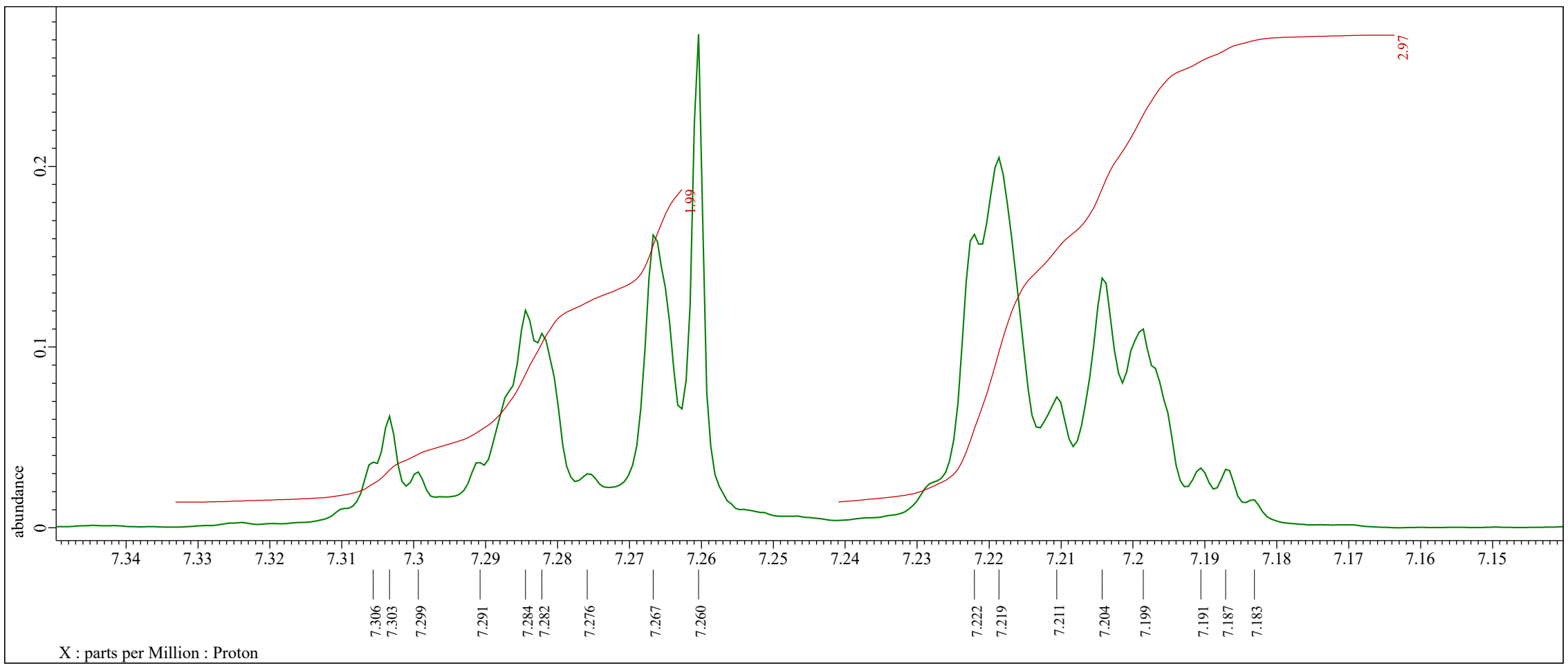

^{13}C{^{1}H} NMR (100 MHz, $CDCl_3$)

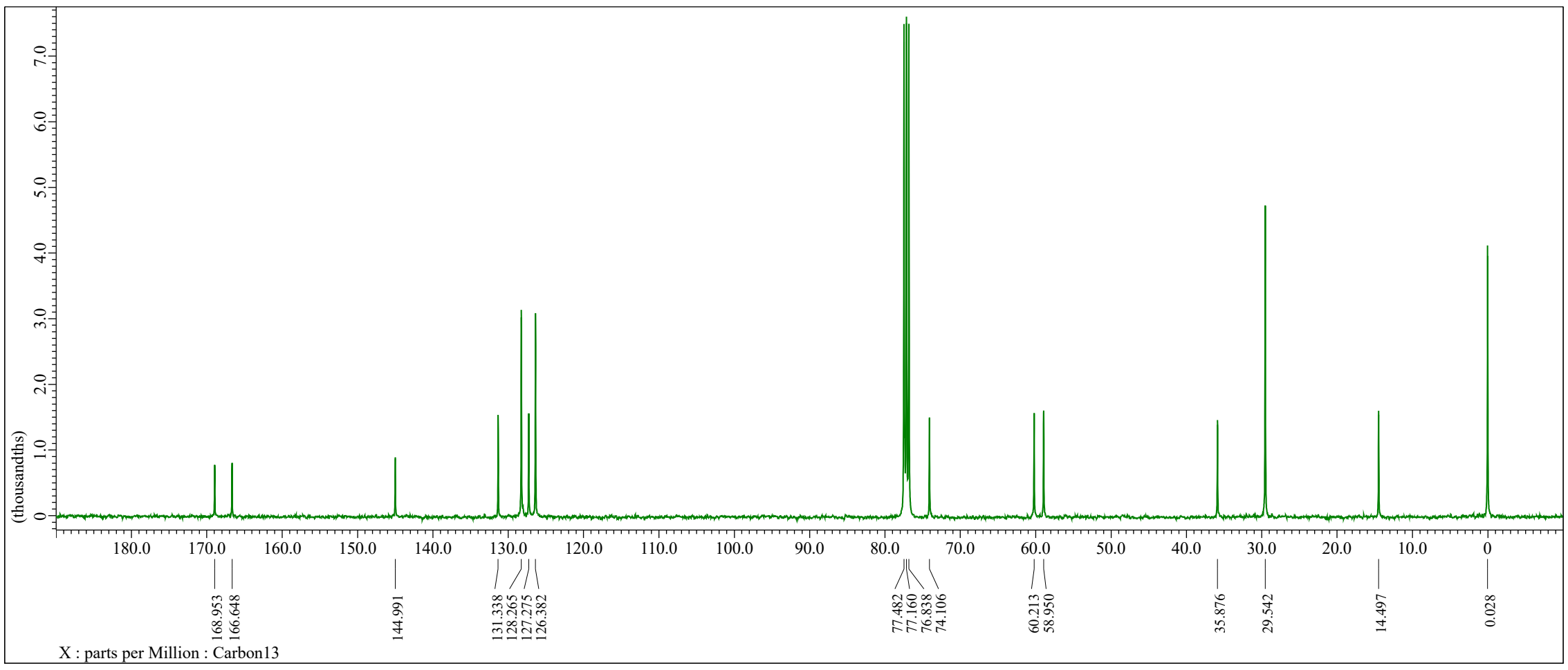

(1*R,2*S**,*Z*)-2-(*tert*-Butyl)-5-methyl-1-phenyl-3-(trimethylgermyl)hex-3-ene-1,5-diol** ***anti*-*γ*-6**

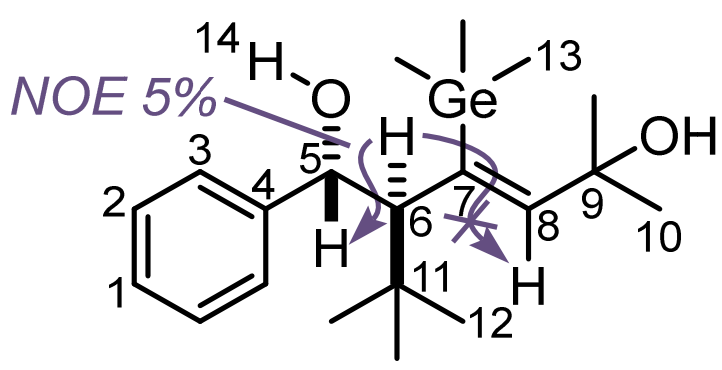

To a mixture of ethyl (*Z*)-2-{(4*R**,5*S**)-4-(*tert*-butyl)-2,2-dichloro-5-phenyl-1,2-oxagermolan-3-ylidene}acetate *anti*-*γ*-**4aa** (0.300 mmol) in THF (3.0 mL) was added MeMgBr (1.0 mL, 3 M in Et_2O, 3.0 mmol) at 0 °C. The reaction mixture was stirred for 16 h at room temperature and quenched with NH_4Cl aq. The organic phase was extracted with Et_2O and dried over Na_2SO_4. The filtrate was evaporated and the residue was purified by column chromatography on SiO_2 (Hexane/EtOAc = 70:30) to give *anti*-*γ*-**6** as a colorless solid of the single diastereomer (0.0944 g, 83%).

mp 98.2–99.0 °C; **IR** (ATR) 3566 (m), 3026 (w), 2961 (s), 1478 (w), 1362 (m), 1225 (m), 1097 (w), 827 (m) cm^{-1}; **^{1}H NMR** (400 MHz, $CDCl_3$) 7.32–7.19 (m, 5H), 6.66 (s, 1H, 8-H), 5.15 (t, *J* = 4.2 Hz, 1H, 5-H), 2.58 (d, *J* = 4.4 Hz, 1H, 6-H), 2.02 (d, *J* = 4.8 Hz, 1H, 14-H), 1.43 (s, 3H, 10-H), 1.39 (s, 3H, 10-H), 0.99 (s, 9H, 12-H), –0.02 (s,

9H, 13-H); **$^{13}C\{^1H\}$ NMR** (100 MHz, $CDCl_3$) 149.0 (d, C-8), 145.5 (s, C-4), 136.8 (s, C-7), 128.1 (d, C-2), 127.1 (d, C-1), 126.7 (d, C-3), 74.3 (d, C-5), 73.2 (s, C-9), 57.5 (d, C-6), 35.5 (s, C-11), 31.9 (q, C-10), 31.3 (q, C-10), 29.7 (q, C-12). 2.64 (q, C-13); **HRMS** (ESI^+) Calculated ($C_{20}H_{34}O_2NaGe$): 403.1663 ($[M+Na]^+$), Found: 403.1660 ($[M+Na]^+$).

^1H NMR (400 MHz, $CDCl_3$)

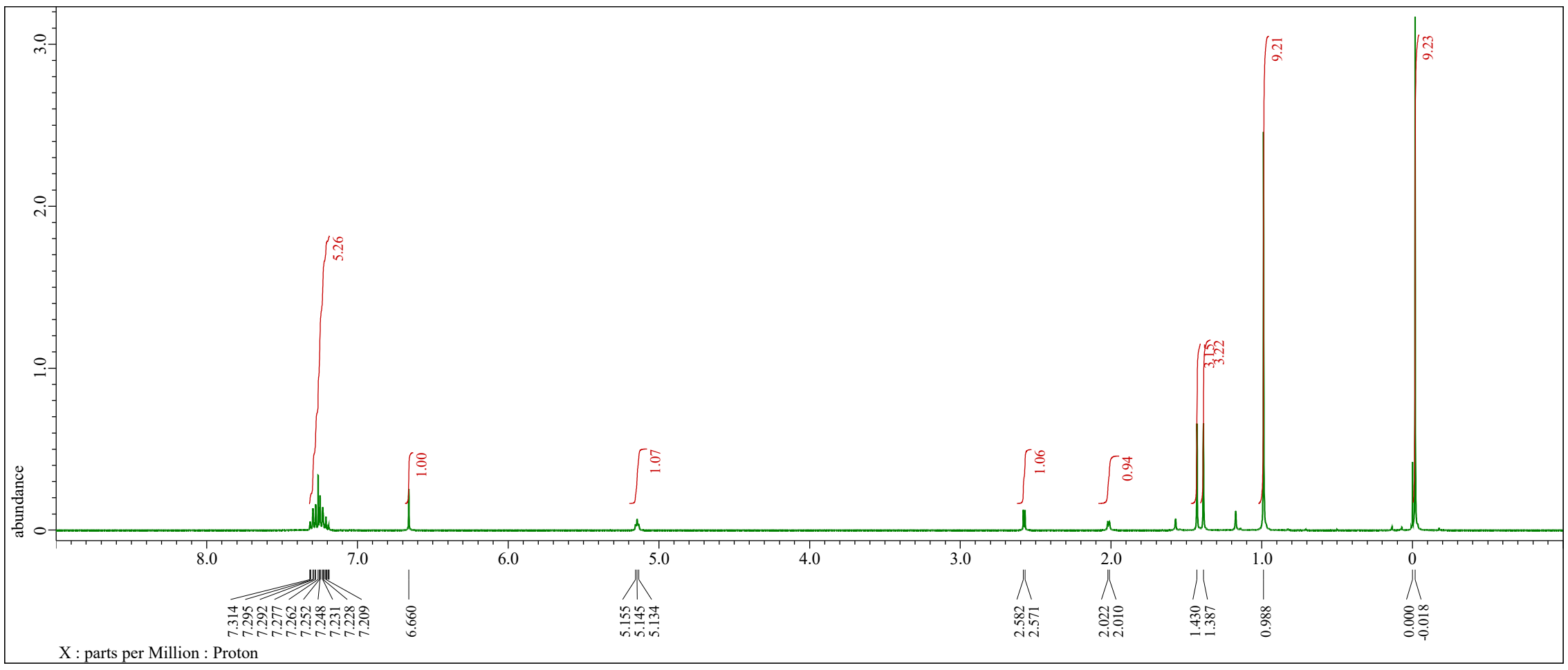

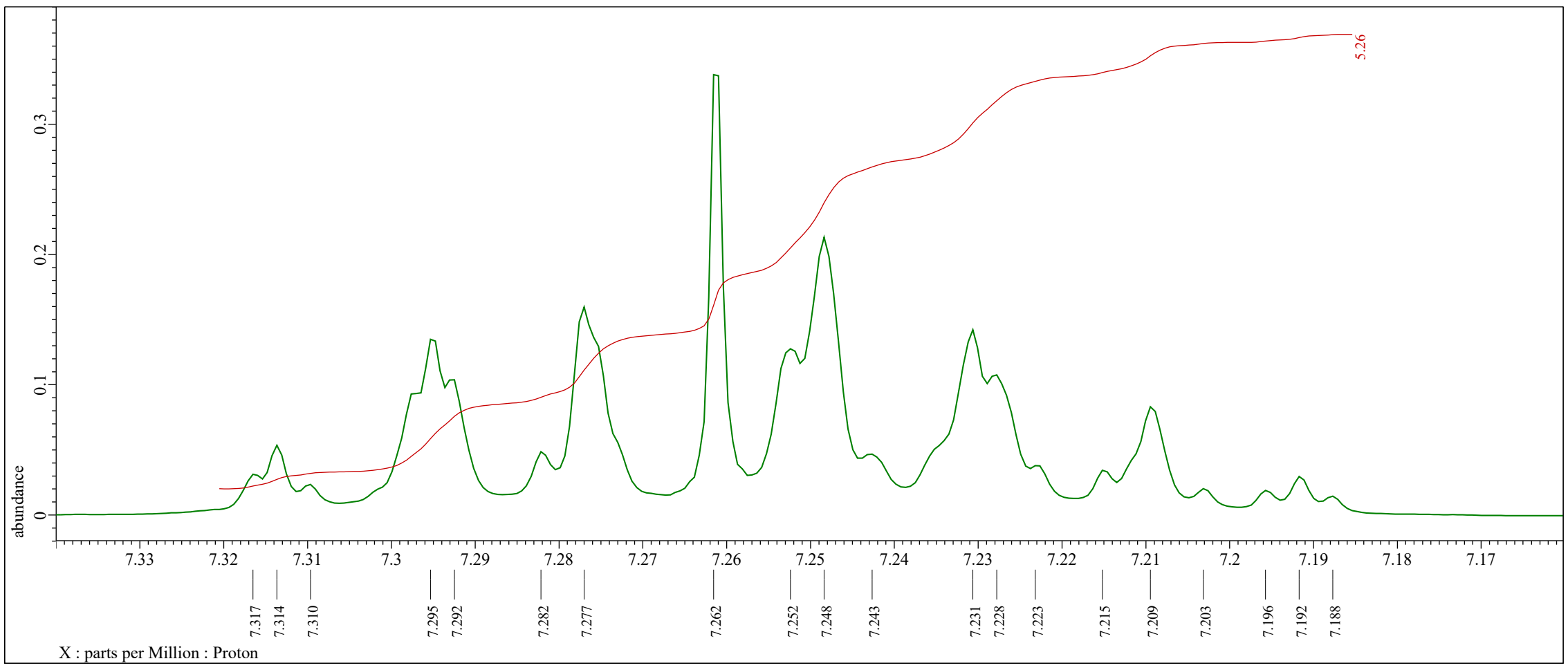

$^{13}C\{^1H\}$ NMR (100 MHz, $CDCl_3$)

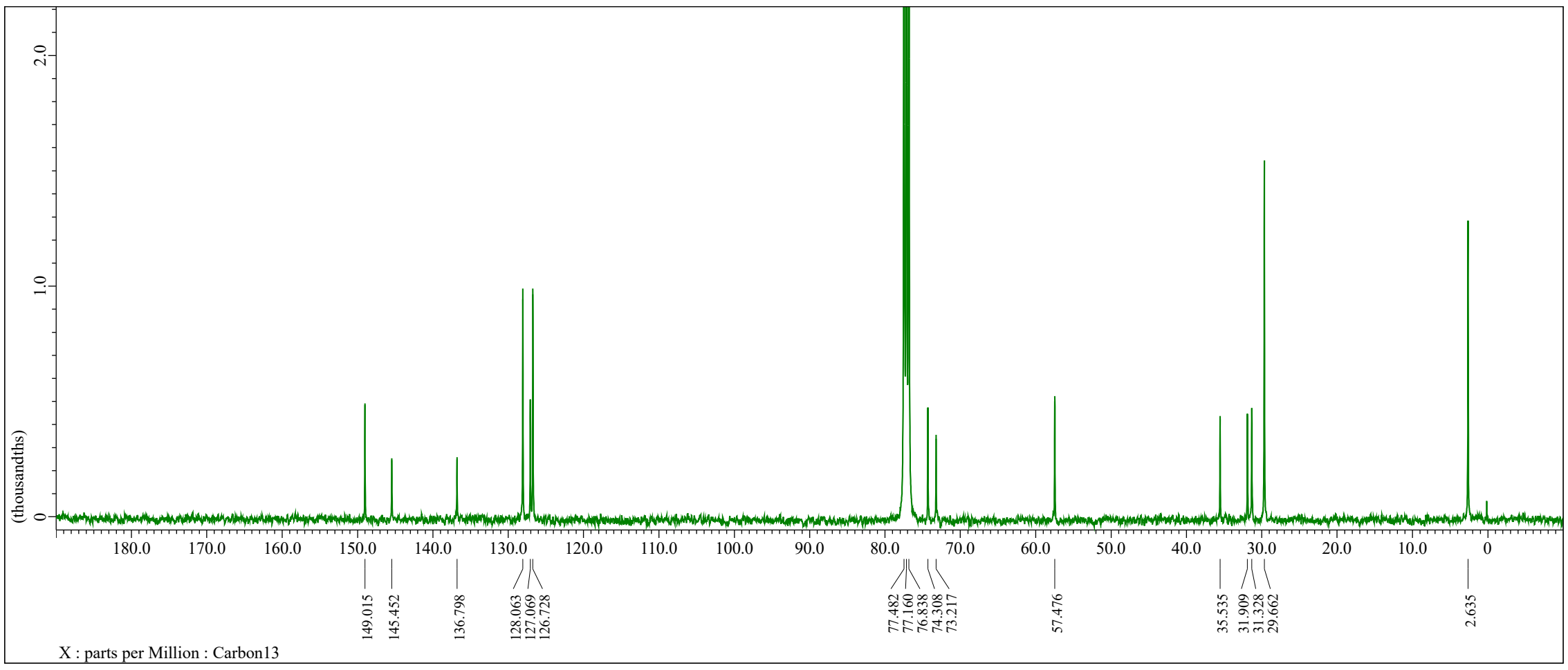

Ethyl (*Z*)-2-{(4*R,5*S**)-4-(*tert*-butyl)-2,2-dimethyl-5-phenyl-1,2-oxagermolan-3-ylidene}acetate 9**

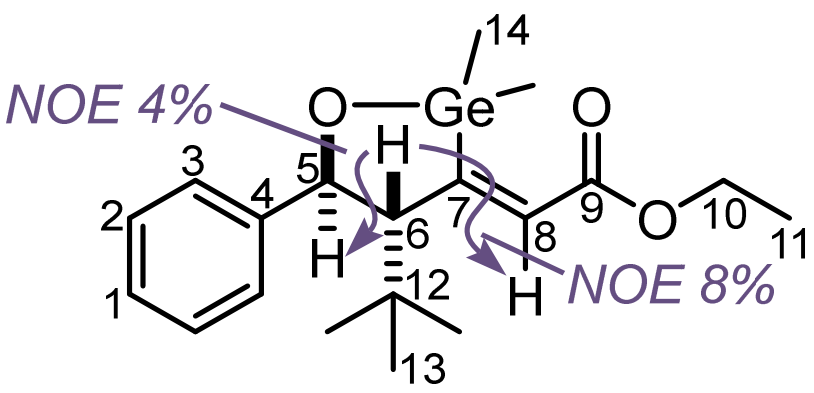

In a nitrogen filled glovebox, *anti*-*γ*-**5** (0.0427 g, 0.109 mmol), $(Ph_3P)AuCl$ (2.47 mg, 5.00 μmol) (+)-10-camphorsulfonic acid (0.0348 g, 0.150 mmol) and $PhI(OAc)_2$ (0.0483 g, 0.0150 mmol) were dissolved in 1,4-dioxane (1.0 mL). After the reaction mixture was stirred at 70 °C for 18 h, the solvent was removed under vacuum and the residue was purified by column chromatography on SiO_2 (Hexane/EtOAc = 80:20) to give **9** as colorless neat of the single diastereomer (0.0159 g, 39%).

IR (ATR) 3028 (w), 2960 (m), 1693 (s), 1368 (m), 1324 (s), 1208 (s), 1099 (m), 1031 (m), 813 (m) cm^{-1}; **^{1}H NMR** (400 MHz, $CDCl_3$) 7.31–7.26 (m, 4H), 7.20–7.16 (m, 1H, C-1), 6.46 (d, *J* = 0.8 Hz, 1H, 8-H), 5.46 (s, 1H, 5-H), 4.25 (dq, *J* = 11.0, 7.4 Hz, 1H, 10-H), 4.21 (dq, *J* = 11.0, 7.4 Hz, 1H, 10-H), 2.82 (s, 1H, 6-H), 1.32 (t, *J* = 7.2 Hz, 3H, 11-H), 1.07 (s, 9H, 13-H), 0.72 (s, 3H, 14-H), 0.46 (s, 3H, 14-H); **$^{13}C\{^1H\}$ NMR** (100 MHz, $CDCl_3$) 172.0 (s, C-7), 168.0 (s, C-9), 147.3 (s, C-4), 128.2 (d, C-2), 127.3 (d, C-8), 126.6 (d, C-1), 126.3 (d, C-3), 78.2 (d, C-5), 62.1 (d, C-6), 61.4 (t, C-10), 35.6 (s, C-12), 28.8 (q, C-13), 14.4 (q, C-11), 3.91 (q, C-14), 1.35 (q, C-14); **HRMS** ($DART^+$) Calculated ($C_{19}H_{29}O_3Ge$): 379.1323 ($[M+H]^+$), Found: 379.1330 ($[M+H]^+$).

^{1}H NMR (400 MHz, $CDCl_3$)

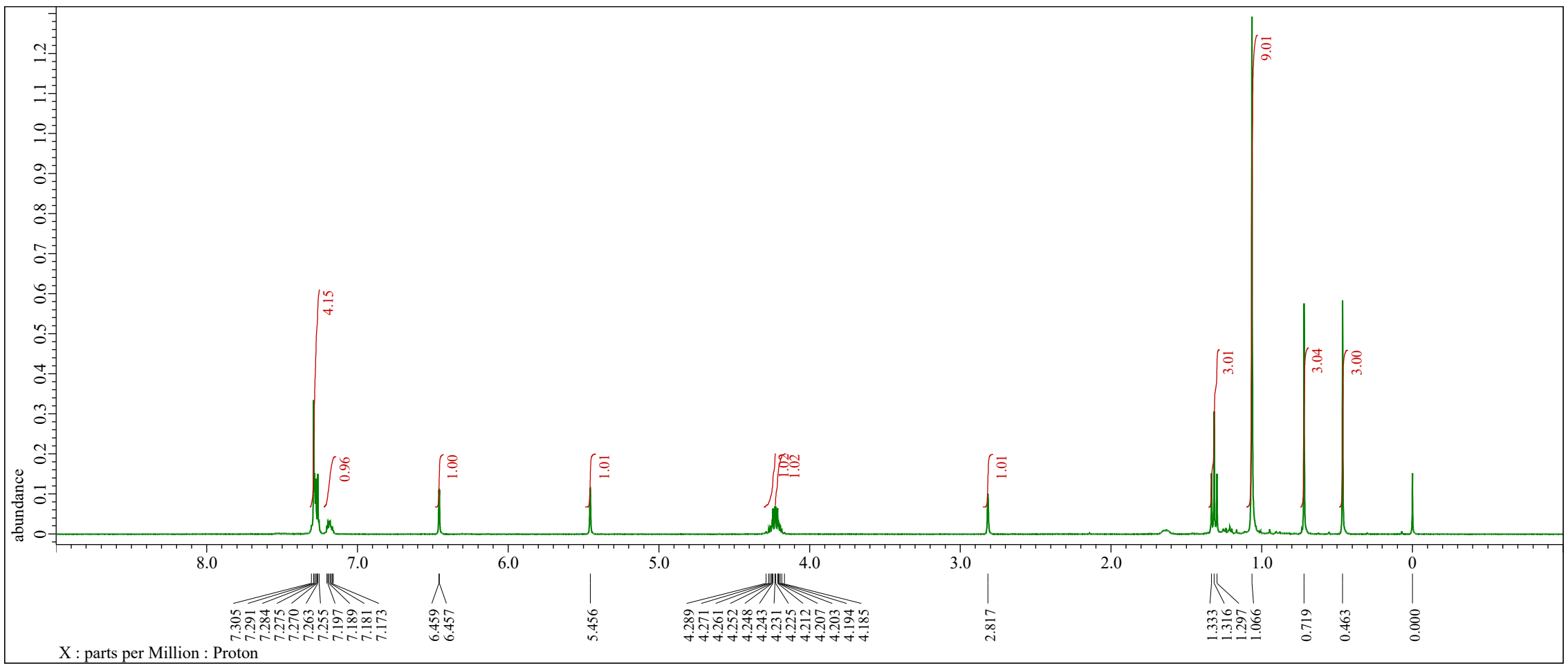

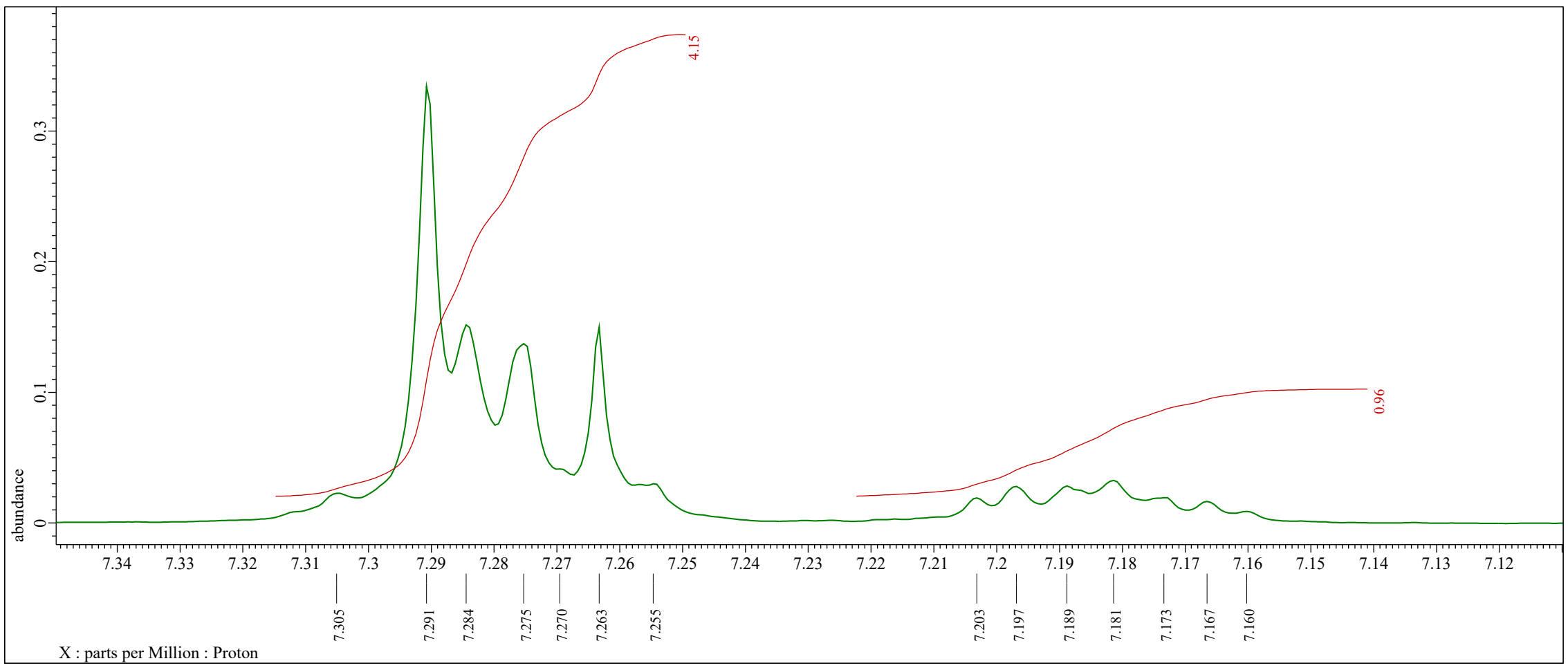

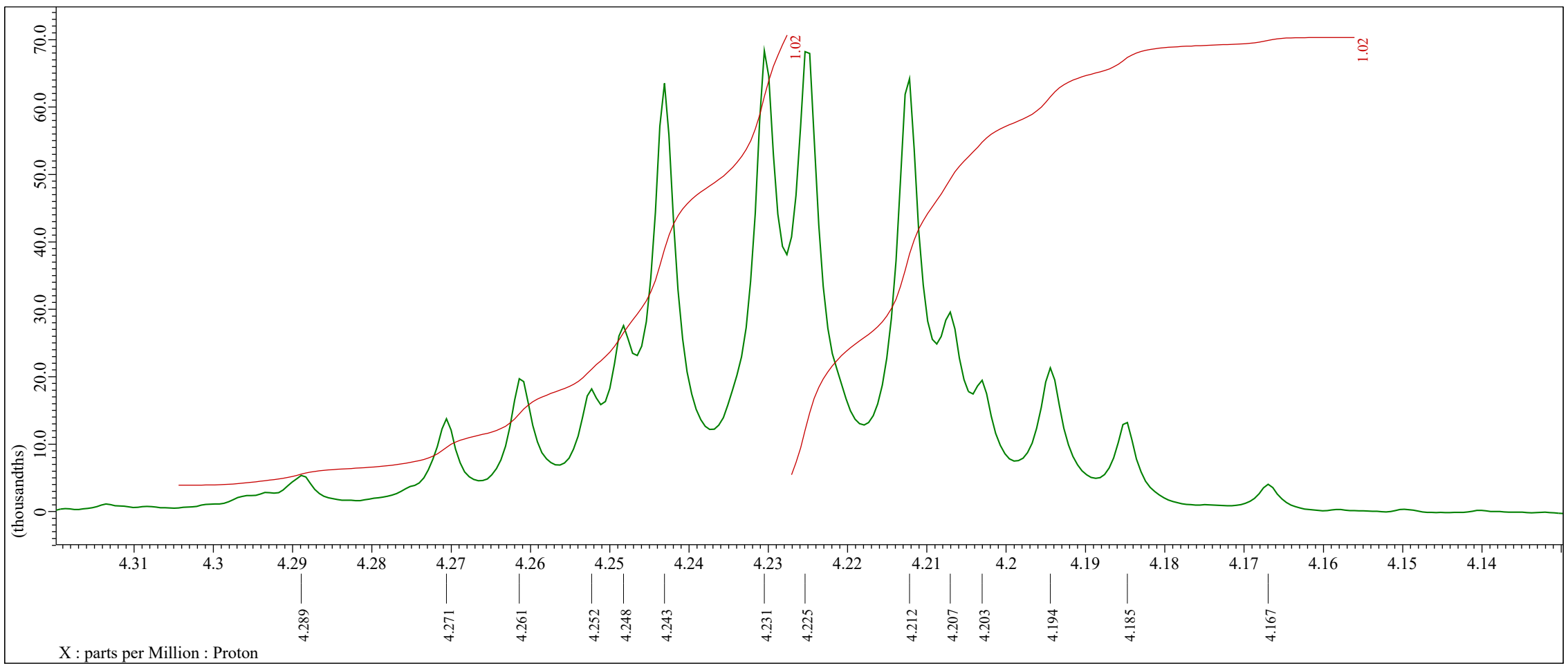

$^{13}C\{^1H\}$ **NMR** (100 MHz, CDCl$_3$)

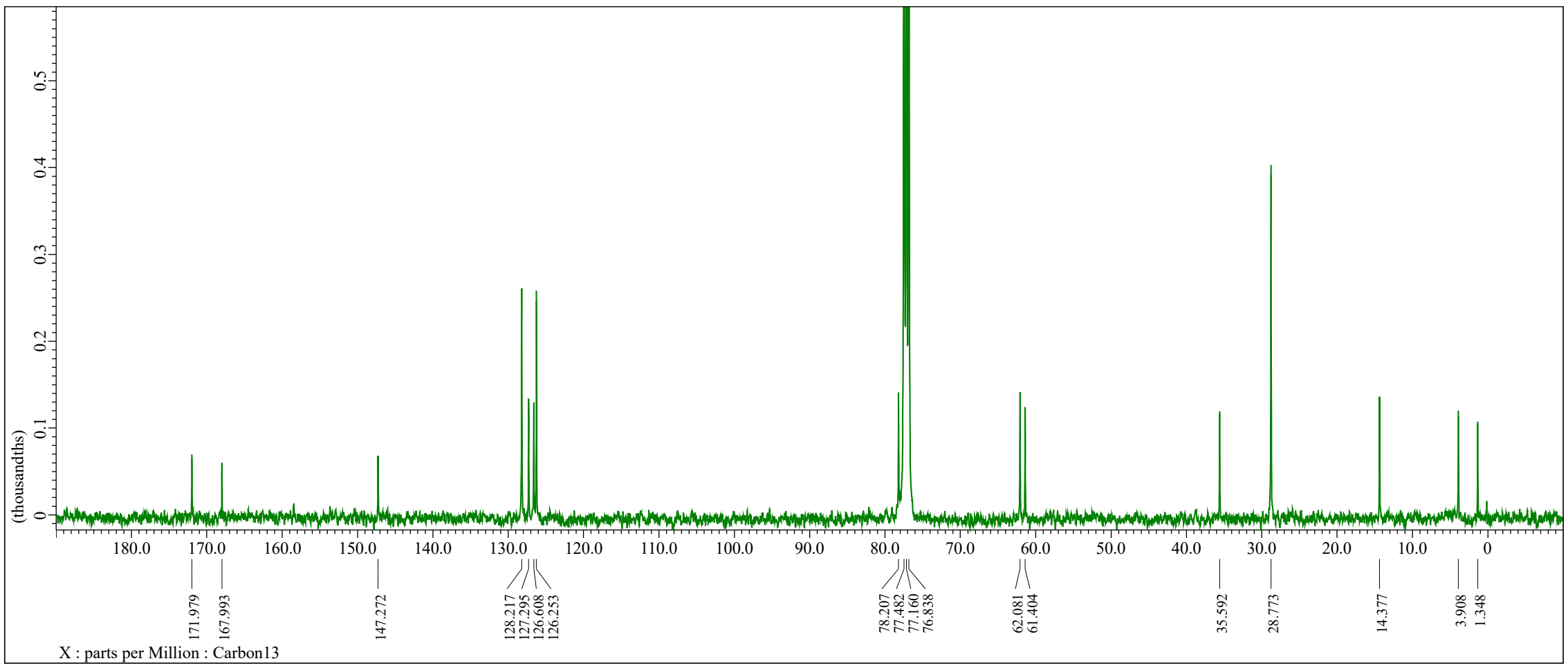

[(*R,*Z*)-2-Methoxy-5-{(*S**)-methoxy(phenyl)methyl}-2,6,6-trimethylhept-3-en-4-yl]trimethylgermane** *anti*-*γ*-**11**

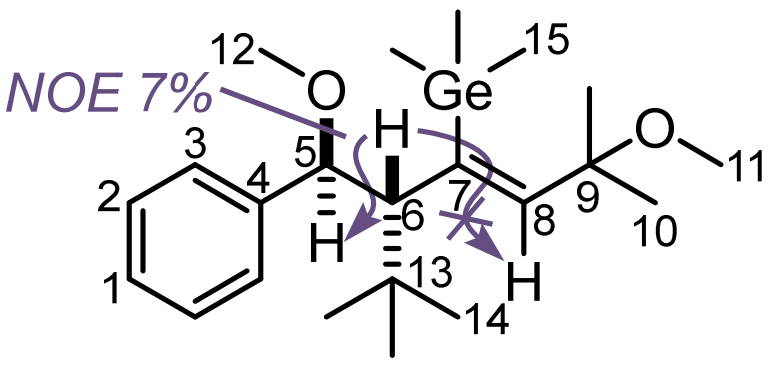

To a mixture of *anti*-*γ*-**6** (0.147 g, 0.388 mmol) in THF (3.9 mL) was added NaH (60% oil dispersion, 0.0745 g, ca. 1.86 mmol) at 0 °C. After the reaction mixture was stirred for 1 h at 0 °C, MeI (0.145 mL, 2.33 mmol) was added to the mixture. The mixture was stirred 14 h at room temperature and quenched with NH_4Cl aq. The organic phase was extracted with Et_2O and dried over Na_2SO_4. The filtrate was evaporated and the residue was purified by column chromatography on SiO_2 (Hexane/EtOAc = 97:3) to give *anti*-*γ*-**11** as a colorless solid of the single diastereomer (0.111 g, 91%).

mp 84.0–85.0 °C; **IR** (KBr) 3027 (m), 2970 (s), 2825 (s), 1613 (w), 1359 (s), 1224 (s), 1082 (s), 829 (s) cm^{-1}; ^{1}**H NMR** (400 MHz, CDCl$_3$) 7.27–7.23 (m, 2H, 2-H), 7.18–7.14 (m, 3H), 6.62 (s, 1H, 8-H), 4.59 (d, J = 3.2 Hz, 1H, 5-H), 3.19 (s, 3H, 12-H), 3.12 (s, 3H, 11-H), 2.33 (d, J = 3.2 Hz, 1H, 6-H), 1.29 (s, 3H, 10-H), 1.25 (s, 3H, 10-H), 1.03 (s, 9H, 14-H), –0.24 (s, 9H, 15-H); $^{13}C\{^1H\}$ **NMR** (100 MHz, CDCl$_3$) 150.2 (d, C-8), 142.8 (s, C-4), 133.4 (s, C-7), 127.8 (d, C-3), 127.6 (d, C-2), 126.4 (d, C-1), 84.9 (d, C-5), 76.3 (s, C-9), 56.9 (d, C-6), 56.6 (q, C-12), 48.3 (q, C-11), 35.8 (s, C-13), 29.6 (q, C-14), 25.6 (q, C-10), 24.6 (q, C-10), 2.30 (q, C-15); **HRMS** (EI$^+$, 70 eV) Calculated ($C_{21}H_{35}O_2Ge$): 393.1853 ([M–Me]$^+$), Found: 393.1854 ([M–Me]$^+$).

^{1}H NMR (400 MHz, $CDCl_3$)

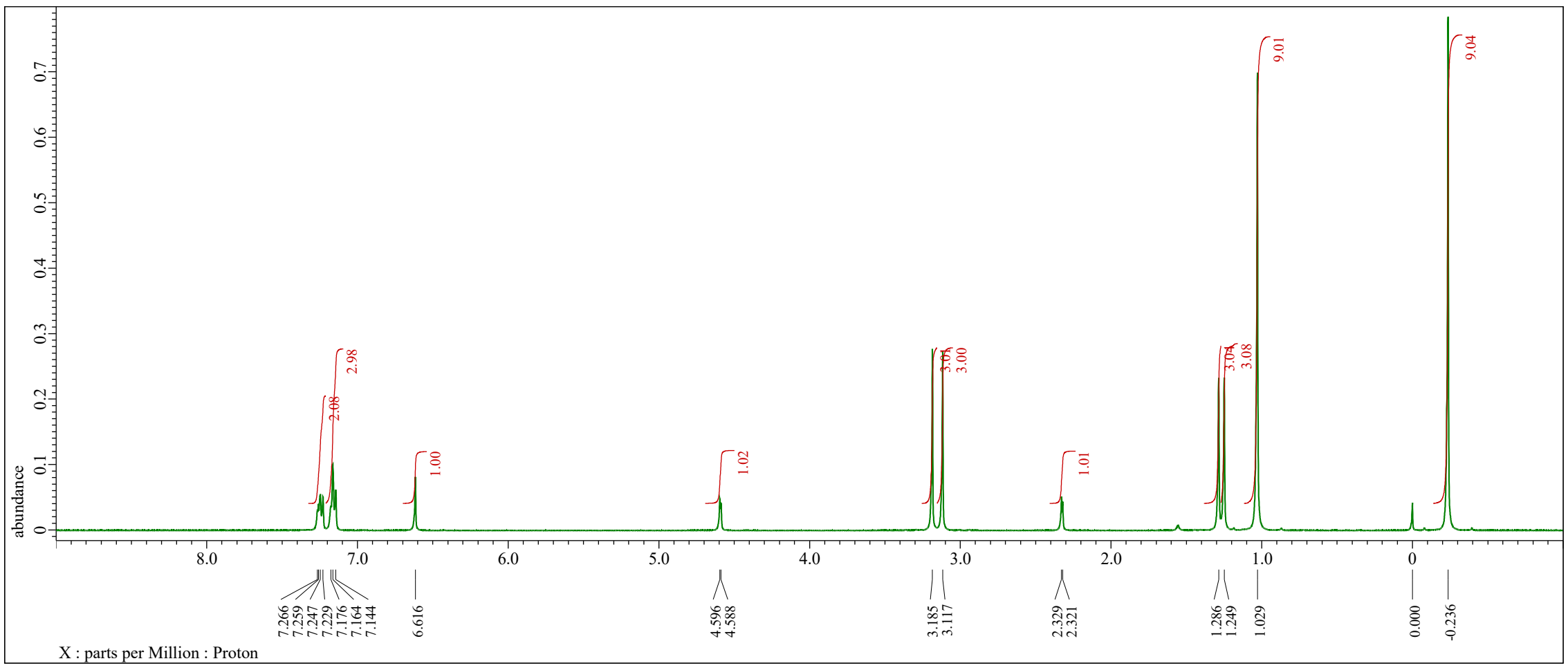

^{13}C{^{1}H} NMR (100 MHz, $CDCl_3$)

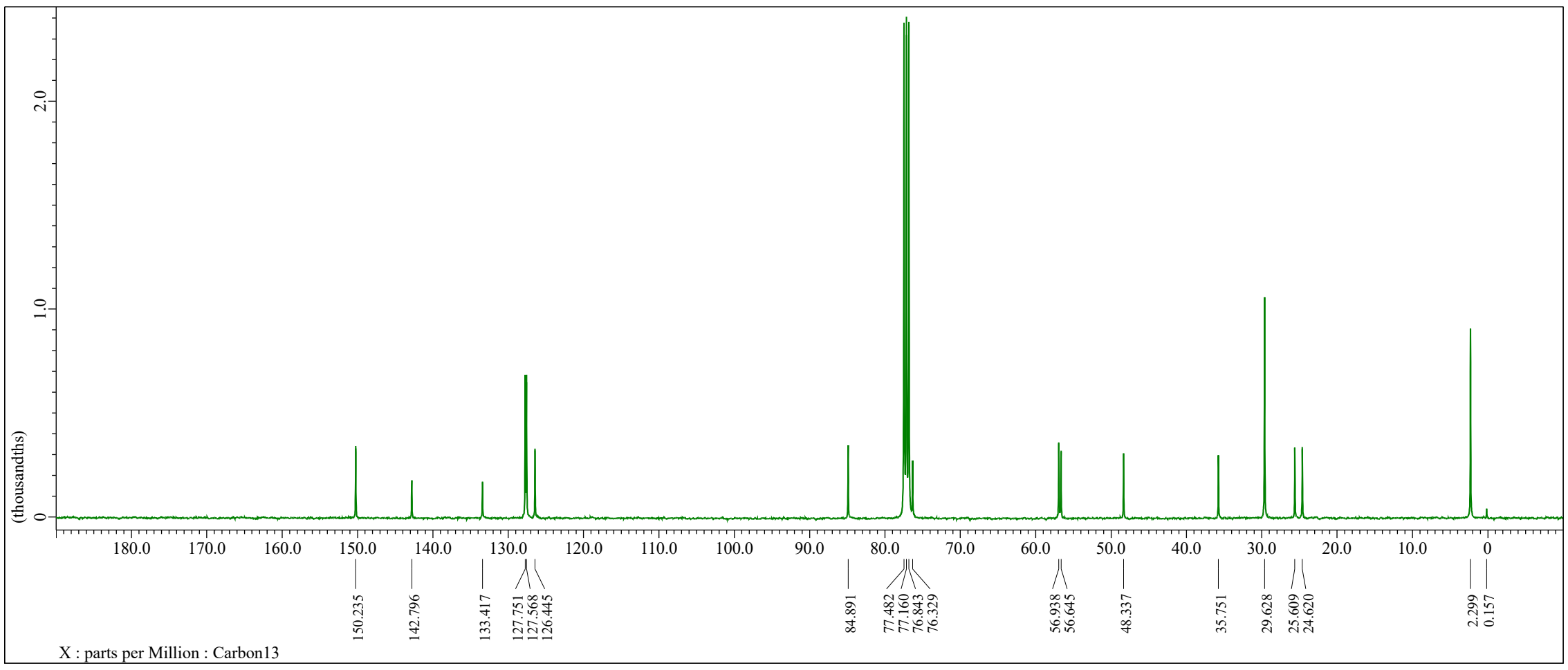

2-(*tert*-Butyl)-1-methoxy-3-(2-methylprop-1-en-1-yl)-1*H*-indene 12

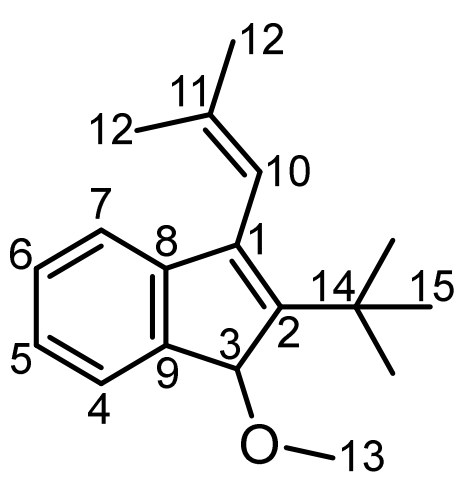

In a nitrogen filled glovebox, *anti*-*γ*-**11** (0.0404 g, 0.0992 mmol), (Ph_3P)AuCl (2.47 mg, 5.00 μmol) (+)-10-camphorsulfonic acid (0.0348 g, 0.150 mmol) and $PhI(OAc)_2$ (0.0483 g, 0.0150 mmol) were dissolved in 1,4-dioxane (1.0 mL). After the reaction mixture was stirred at 70 °C for 5 h, the solvent was removed under vacuum and the residue was purified by column chromatography on SiO_2 (Hexane/EtOAc = 95:5) to give **12** as colorless neat (0.00983 g, 39%).

IR (ATR) 3068 (w), 2952 (s), 1712 (w), 1603 (w), 1459 (m), 1192 (m), 1104 (s), 1068 (s), 893 (m) cm^{-1}; **^{1}H NMR**

(400 MHz, $CDCl_3$) 7.39 (d, *J* = 7.2 Hz, 1H, 4-H), 7.22 (t, *J* = 7.6 Hz, 1H, 6-H), 7.13 (t, *J* = 7.4 Hz, 1H, 5-H), 6.97 (d, *J* = 7.2 Hz, 1H, 7-H), 5.96 (s, 1H, 10-H), 5.16 (s, 1H, 3-H), 3.05 (s, 3H, 13-H), 1.91 (s, 3H, 12-H), 1.52 (s, 3H, 12-H), 1.30 (s, 9H, 15-H); **$^{13}C\{^1H\}$ NMR** (100 MHz, $CDCl_3$) 149.8 (s, C-2), 145.6 (s, C-8), 141.0 (s, C-9), 137.9 (s, C-1), 136.4 (s, C-11), 128.3 (d, C-6), 125.0 (d, C-5), 123.3 (d, C-4), 119.8 (d, C-7), 119.4 (d, C-10), 83.9 (d, C-3), 51.8 (q, C-13), 34.1 (s, C-14), 30.1 (q, C-15), 25.3 (q, C-12), 20.4 (q, C-12); **HRMS** (EI^+, 70 eV) Calculated ($C_{18}H_{24}O$): 256.1827 (M^+), Found: 256.1826 (M^+)

^1H NMR (400 MHz, $CDCl_3$)

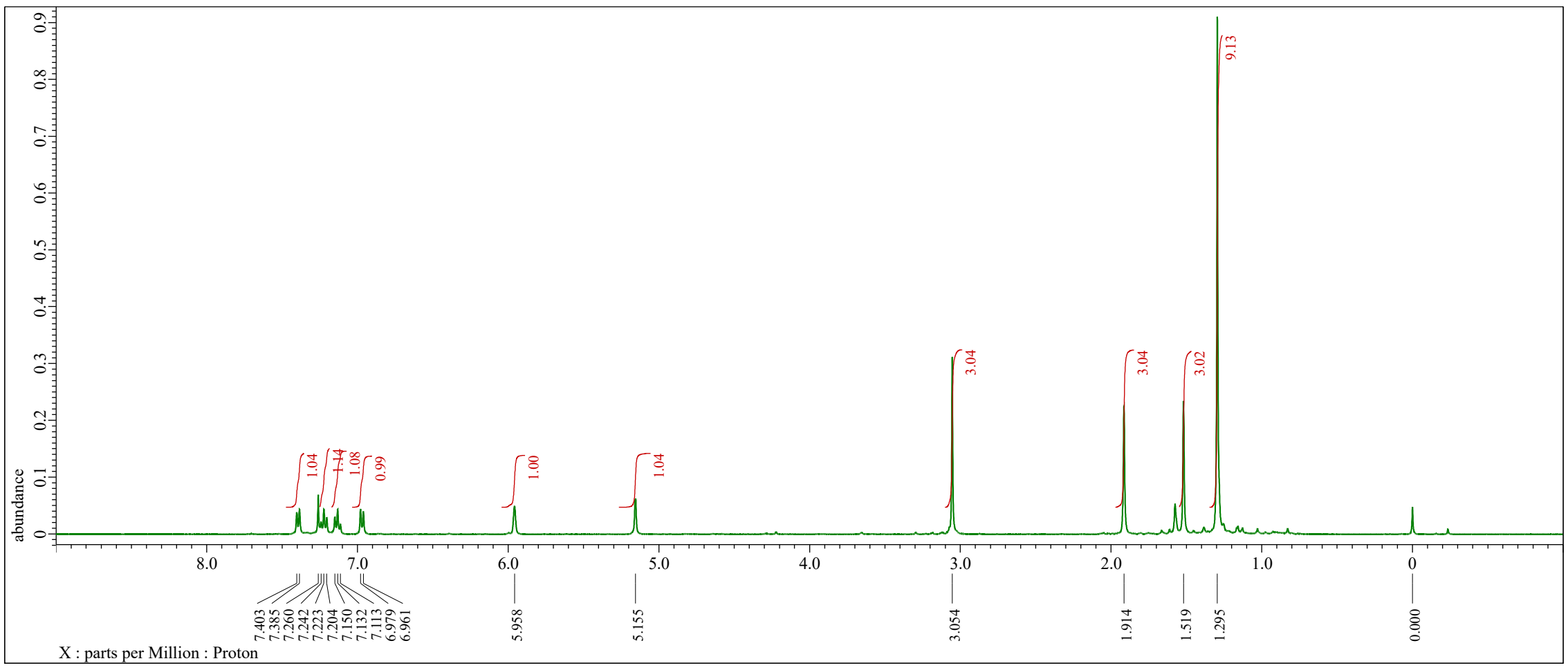

$^{13}C\{^1H\}$ NMR (100 MHz, $CDCl_3$)

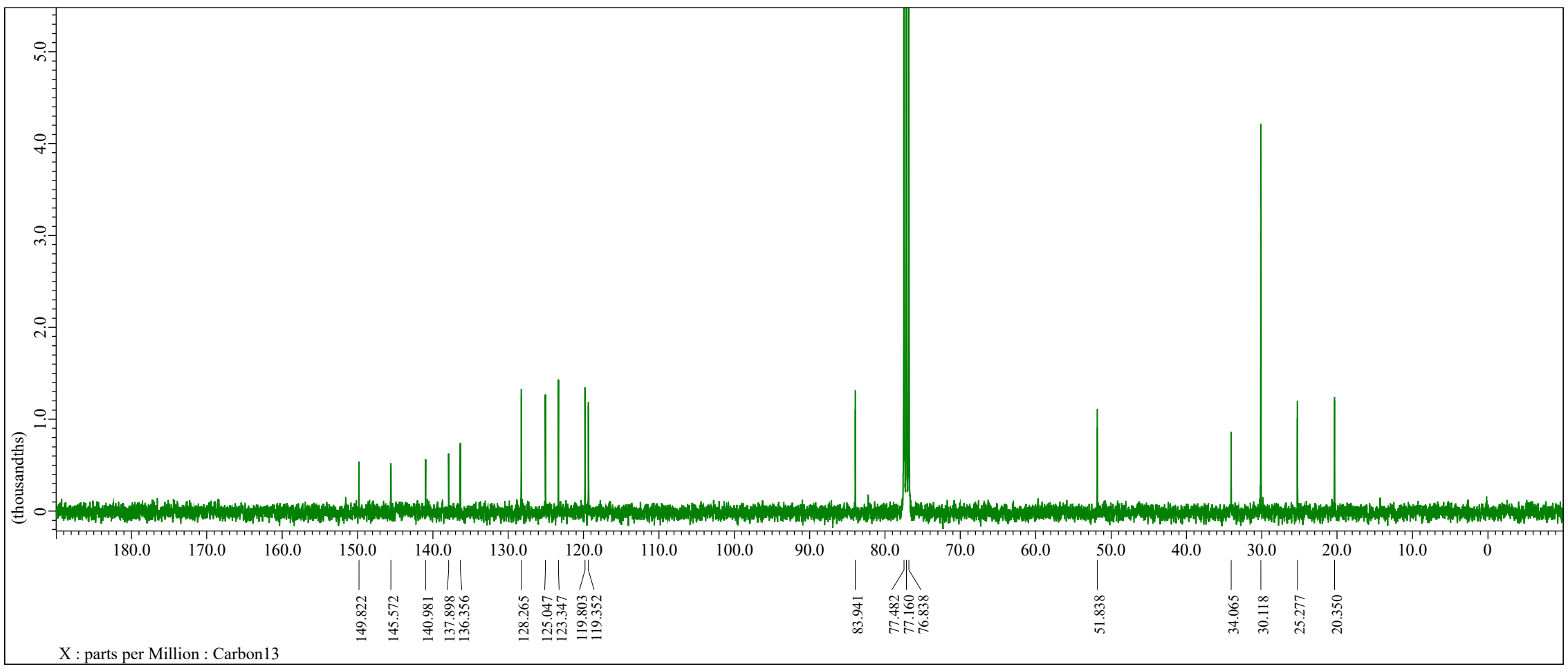

3-10. Derivatizations of *syn-α*-4aa

(1*R,2*R**)-2-{(*E*)-3,3-Dimethyl-1-(trimethylgermyl)but-1-en-1-yl}-3-methyl-1-phenylbutane-1,3-diol *syn-α*-6**

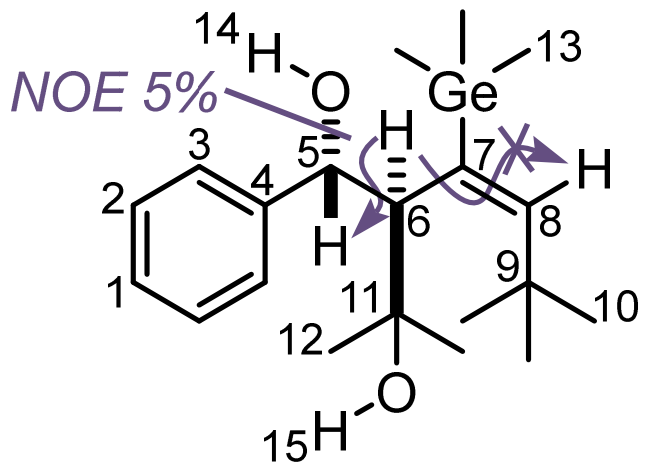

To a mixture of *syn-α*-**4aa** (0.158 g, 0.189 mmol) in THF (1.9 mL) was added MeMgBr (0.631 mL, 3 M in Et_2O, 1.89 mmol) at 0 °C. The reaction mixture was stirred for 20 h at room temperature and quenched with NH_4Cl aq. The organic phase was extracted with Et_2O and dried over Na_2SO_4. The filtrate was evaporated and the sum yield of diastereomers and the diastereo ratio were determined by ^{1}H NMR in the crude product, in which 1,1,2,2-tetrachloroethane was used as an internal standard (83%, *syn-α*-**6**/*anti-α*-**6** = 66/34). The crude was purified by column chromatography on SiO_2 (Hexane/EtOAc = 80:20) to give *syn-α*-**6** as a colorless solid (0.0813 g, 57%).

mp 112.0–112.5 °C; **IR** (ATR) 3292 (m), 3027 (w), 2962 (s), 1470 (m), 1362 (m), 1209 (m), 1148 (m), 950 (m), 829 (s) cm^{-1}; ^{1}**H NMR** (400 MHz, $CDCl_3$) 7.33–7.28 (m, 4H, 2-H, 3-H), 7.25–7.19 (m, 1H, 1-H), 5.71 (s, 1H, 8-H), 5.17 (dd, *J* = 5.2, 1.2 Hz, 1H, 5-H), 3.44 (d, *J* = 4.8 Hz, 1H, 6-H), 2.86 (d, *J* = 1.2 Hz, 1H, 14-H), 2.28 (s, 1H, 15-H), 1.29 (s, 3H, 12-H), 1.12 (s, 3-H, 12-H), 0.83 (s, 9H, 10-H), 0.35 (s, 9H, 13-H); ^{13}**C{^{1}H} NMR** (100 MHz, $CDCl_3$) 153.6 (d, C-8), 143.7 (s, C-4), 137.9 (s, C-7), 128.1 (d, C-2), 127.6 (d, C-1, C-3, two signals were overlapped.), 74.6 (d, C-5), 73.1 (s, C-11), 55.3 (d, C-6), 36.1 (s, C-9), 31.4 (q, C-10), 30.7 (q, C-12), 30.6 (q, C-12), 3.1 (q, C-13); **HRMS** (ESI^{+}) Calculated ($C_{20}H_{34}O_2NaGe$): 403.1663 ([M+Na]$^{+}$), Found: 403.1661 ([M+Na]$^{+}$).

^{1}**H NMR** (400 MHz, $CDCl_3$)

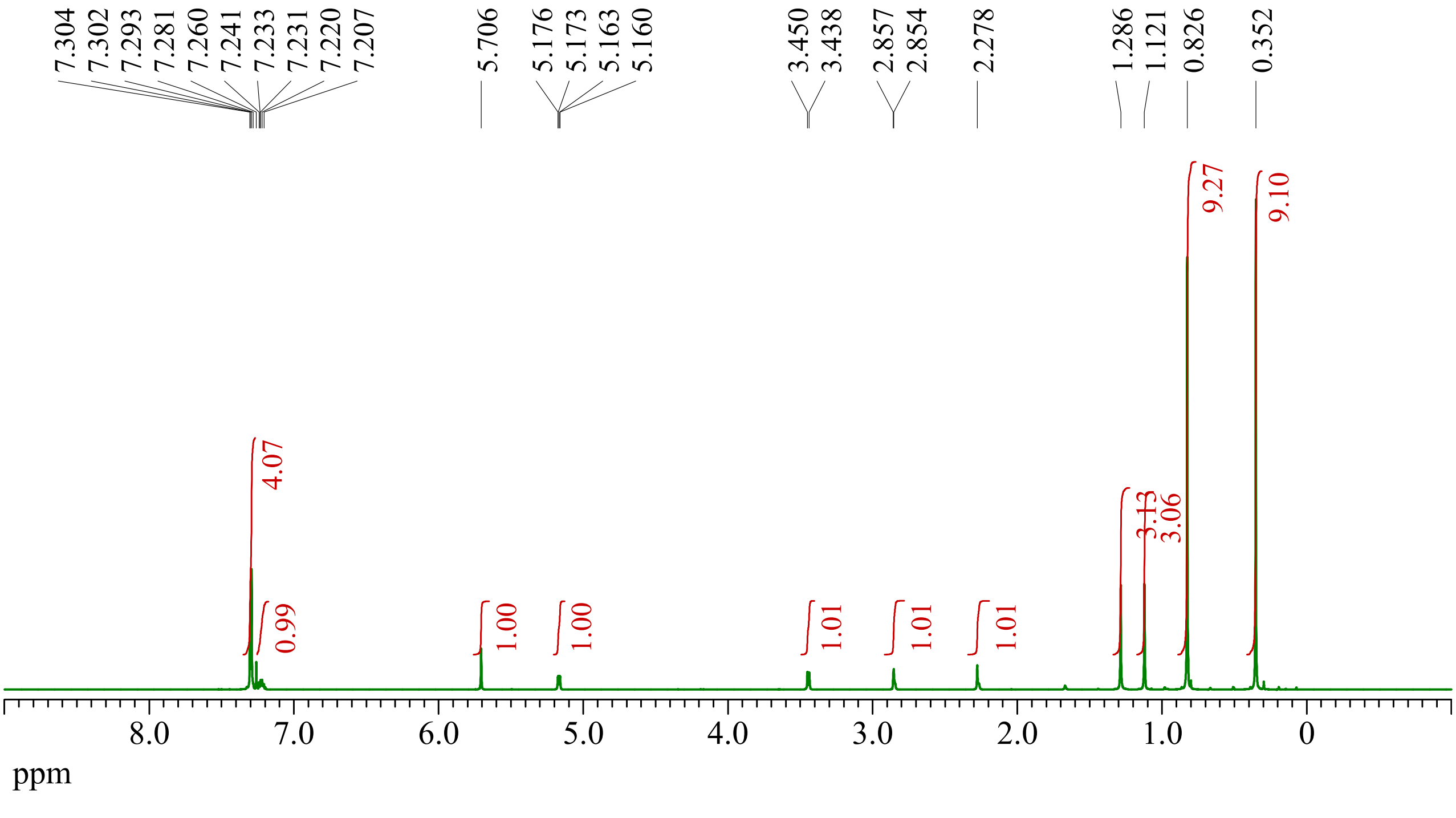

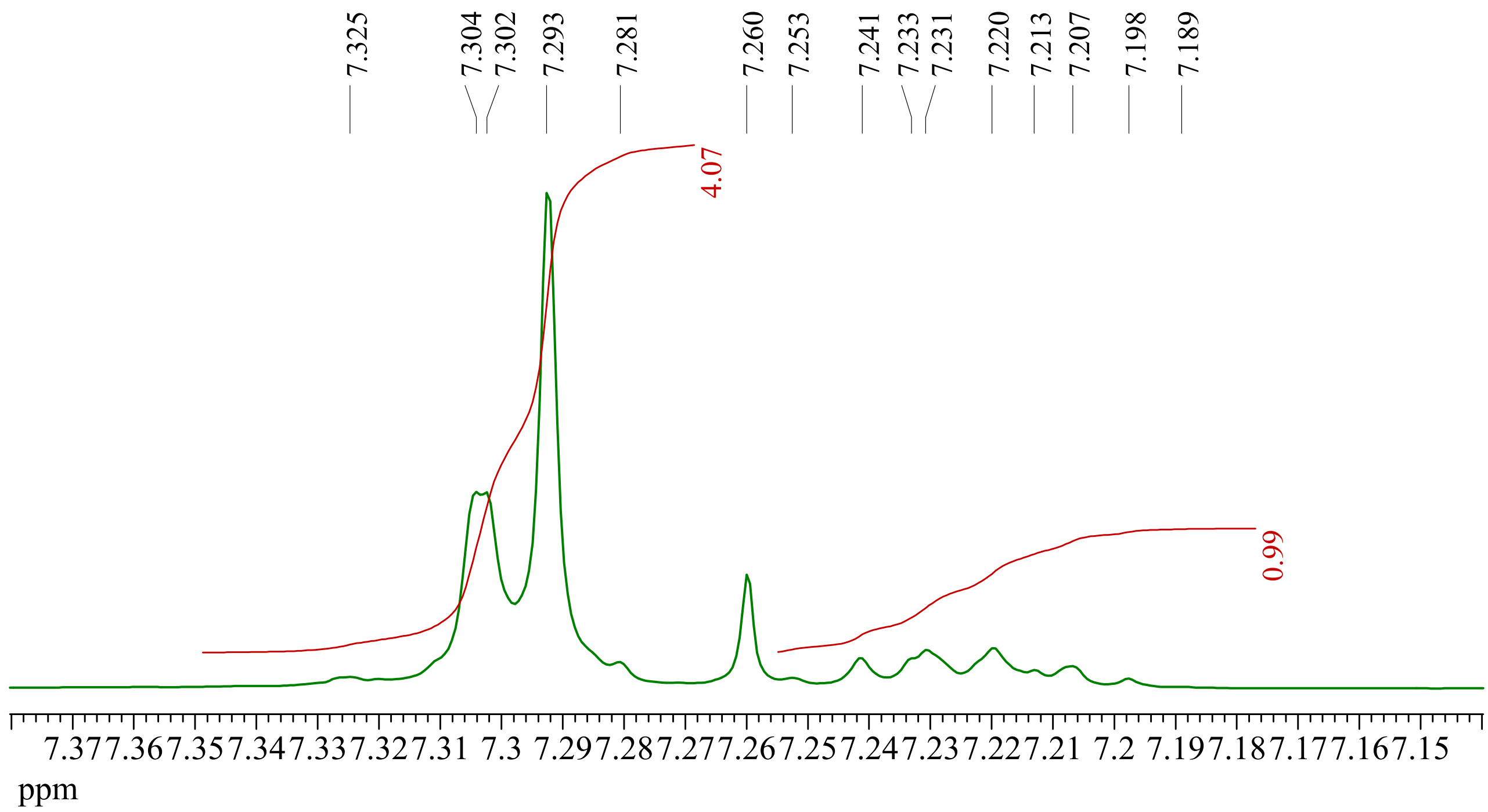

$^{13}C\{^{1}H\}$ NMR (100 MHz, $CDCl_3$)

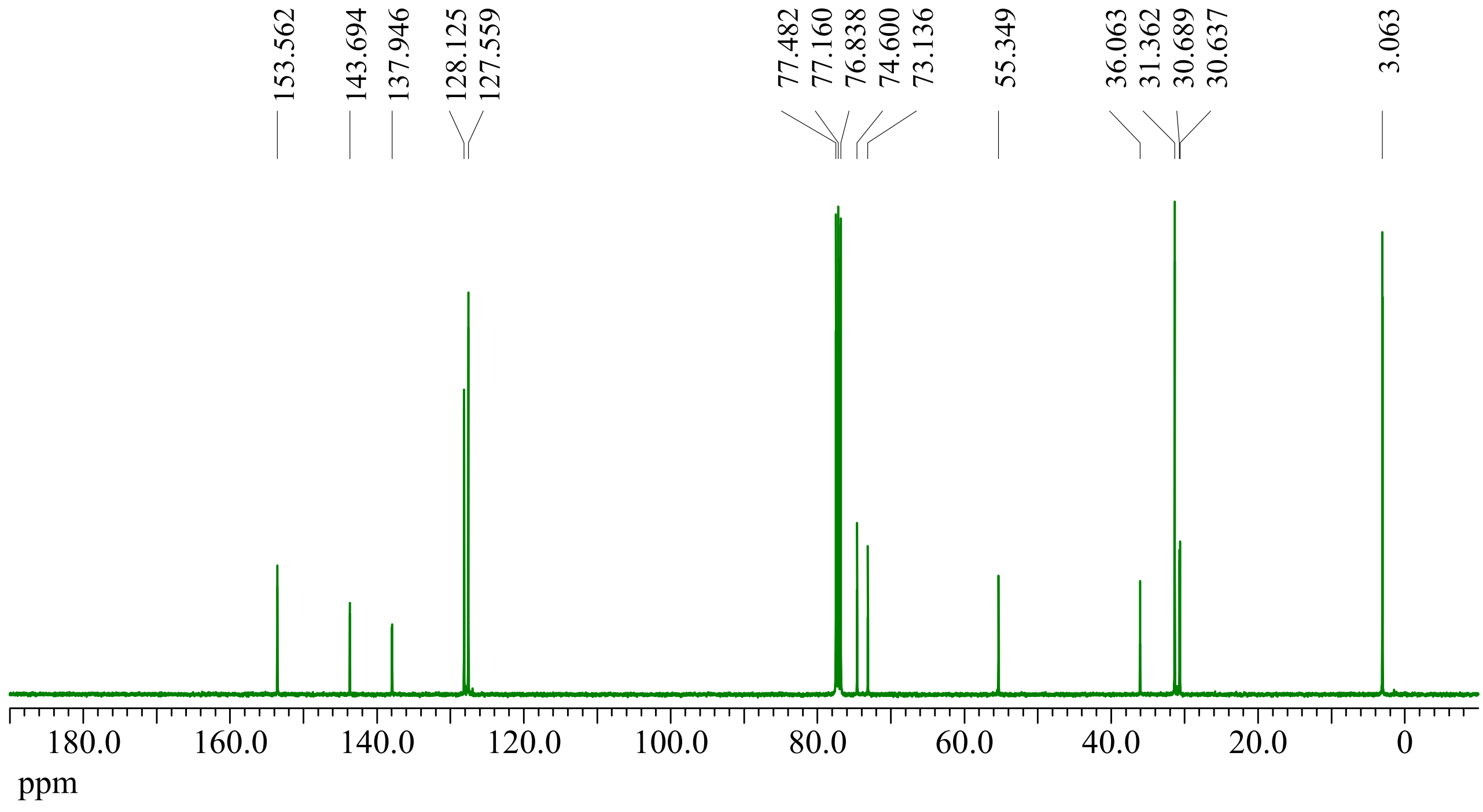

Compound 10

***Major form A*: (*R**)-{(*R**,*E*)-3-(2,2-Dimethylpropylidene)-2,2,5,5-tetramethyl-1,2-oxagermolan-4-yl}(phenyl)methanol**

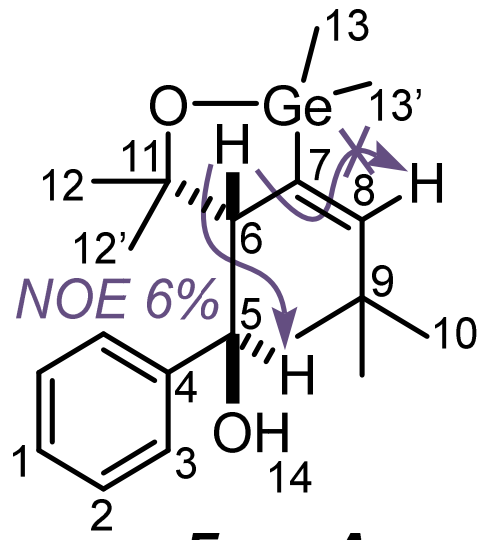

Form A

***Minor form B*: 2-{(4*R**,5*R**,*E*)-3-(2,2-Dimethylpropylidene)-2,2-dimethyl-5-phenyl-1,2-oxagermolan-4-yl}propan-2-ol**

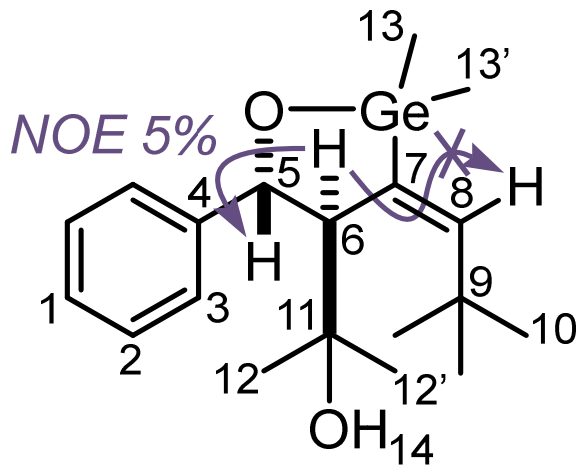

Form B

In a nitrogen filled glovebox, *syn*-*α*-**6** (0.0388 g, 0.102 mmol), (Ph_3P)AuCl (2.70 mg, 5.46 μmol), (+)-10-camphorsulfonic acid (0.0368 g, 0.158 mmol), and PhI(OAc)$_2$ (0.0501 g, 0.0156 mmol) were dissolved in 1,4-dioxane (1.0 mL). After the reaction mixture was stirred at 70 °C for 5 h, the solvent was removed under vacuum and the residue was purified by column chromatography on SiO_2 (Hexane/EtOAc = 50:50) to give inseparable mixture of two forms of compound **10** (*major form A* and *minor form B*) as a colorless solid of the single diastereomer (*form A*/*B* = 1:0.32, 0.0203 g, 55%).

IR: (ATR) 3313 (m), 3027 (w), 2958 (s), 1468 (m), 1362 (m), 1200 (m), 1135 (m), 945 (m), 848 (m) cm^{-1}; **^{1}H NMR** (400 MHz, $CDCl_3$) 7.31–7.17 (m, 6.6H, *forms A and B*), 5.74 (d, *J* = 0.8 Hz, 0.32H, *form B*, 8-H), 5.70 (d, *J* = 1.2 Hz, 1H, *form A*, 8-H), 5.47 (s, 0.32H, *form B*, 5-H), 5.11 (d, *J* = 4.0 Hz, 1H, *form A*, 5-H), 3.53 (s, 1H, *form A*, 14-H), 3.49 (s, 0.32H, *form B*, 6-H), 3.21 (dd, *J* = 4.2, 1.0 Hz, 1H, *form A*, 6-H), 2.53 (s, 0.32H, *form B*, 14-H), 1.49 (s, 3H, *form A*, 12-H), 1.43 (s, 0.96H, *form B*, 12-H), 1.34 (s, 0.96H, *form B*, 12-H), 1.14 (s, 3H, *form A*, 12-H), 1.00 (s, 2.88H, *form B*, 10-H), 0.75 (s, 3H, *form A*, 13-H), 0.68 (s, 9H, *form A*, 10-H), 0.66 (s, 0.96H, *form B*, 13-H), 0.56 (s, 3H, *form A*, 13-H), 0.50 (s, 0.96H, *form B*, 13-H); **^{13}C{^{1}H} NMR** (100 MHz, $CDCl_3$) 153.0 (d, *form B*, C-8), 152.7 (d, *form A*, C-8), 145.8 (s, *form B*, C-4), 142.6 (s, *form A*, C-4), 137.2 (s, *form A*, C-7), 136.0 (s, *form B*, C-7), 128.1 (d, *form A*, C-2; d, *form B*, C-2; two signals were overlapped.), 128.0 (d, *form A*, C-3), 127.5 (d, *form A*, C-1), 126.7 (d, *form B*, C-1), 125.6 (d, *form B*, C-3), 80.0 (s, *form A*, C-11; d, *form B*, C-5; two signals were overlapped.), 74.9 (d, *form A*, C-5), 72.7 (s, *form B*, C-11), 57.4 (d, *form B*, C-6), 55.3 (d, *form A*, C-6), 37.3 (s, *form B*, C-9), 36.9 (s, *form A*, C-9), 31.5 (q, *form A*, C-12), 30.4 (q, *form B*, C-10), 30.1 (q, *form A*, C-10), 29.6 (q, *form B*, C-12), 29.1 (q, *form B*, C-12), 27.8 (q, *form A*, C-12), 4.1 (q, *form B*, C-13), 3.9 (q, *form A*, C-13), 2.5 (q, *form B*, C-13), 1.8 (q, *form A*, C-13); **HRMS** (DART$^+$) Calculated ($C_{19}H_{31}O_2Ge$): 365.1530 ([M+H]$^+$), Found: 365.1535

([M+H]$^+$).

^{1}H NMR (400 MHz, $CDCl_3$)

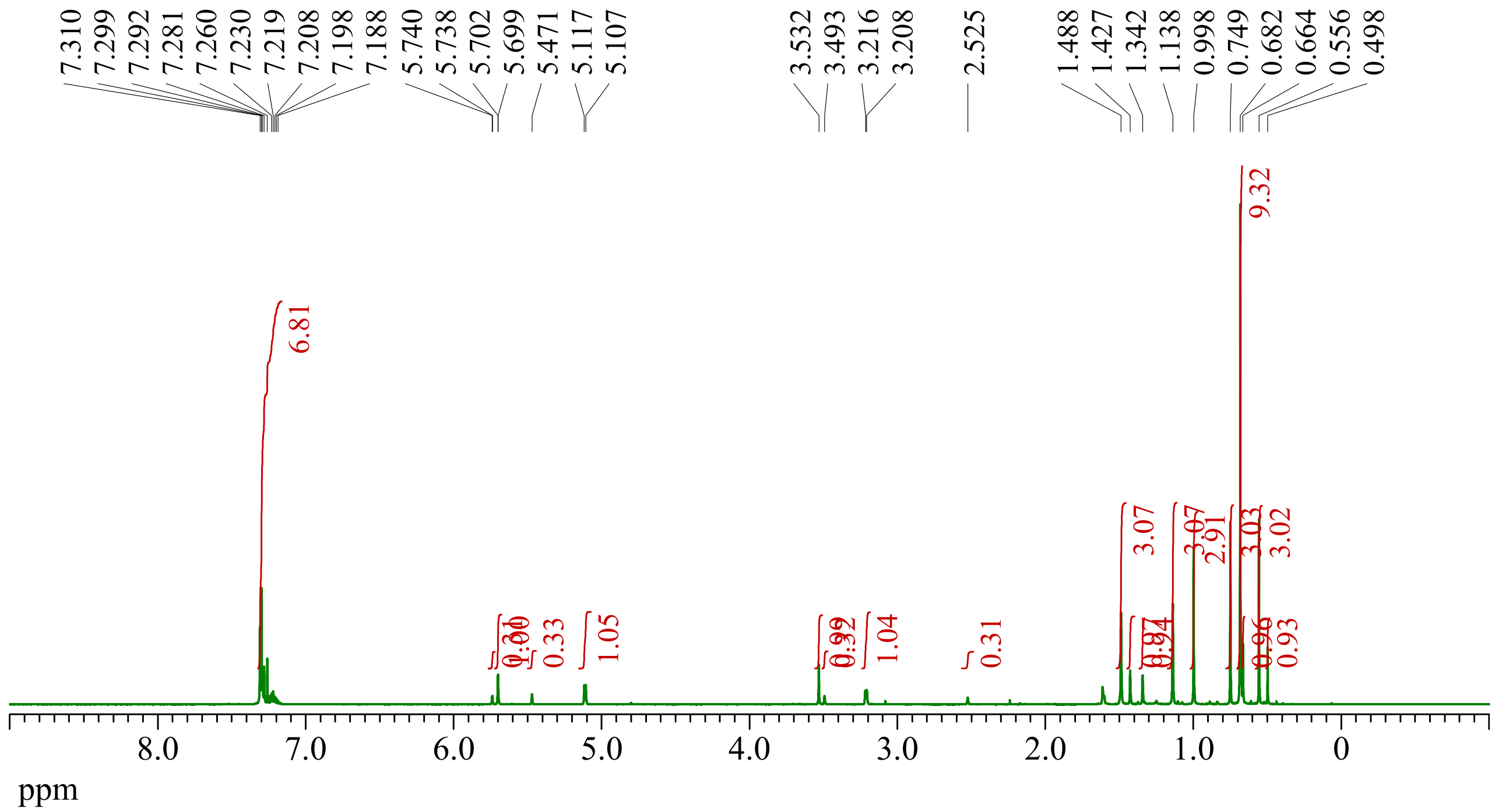

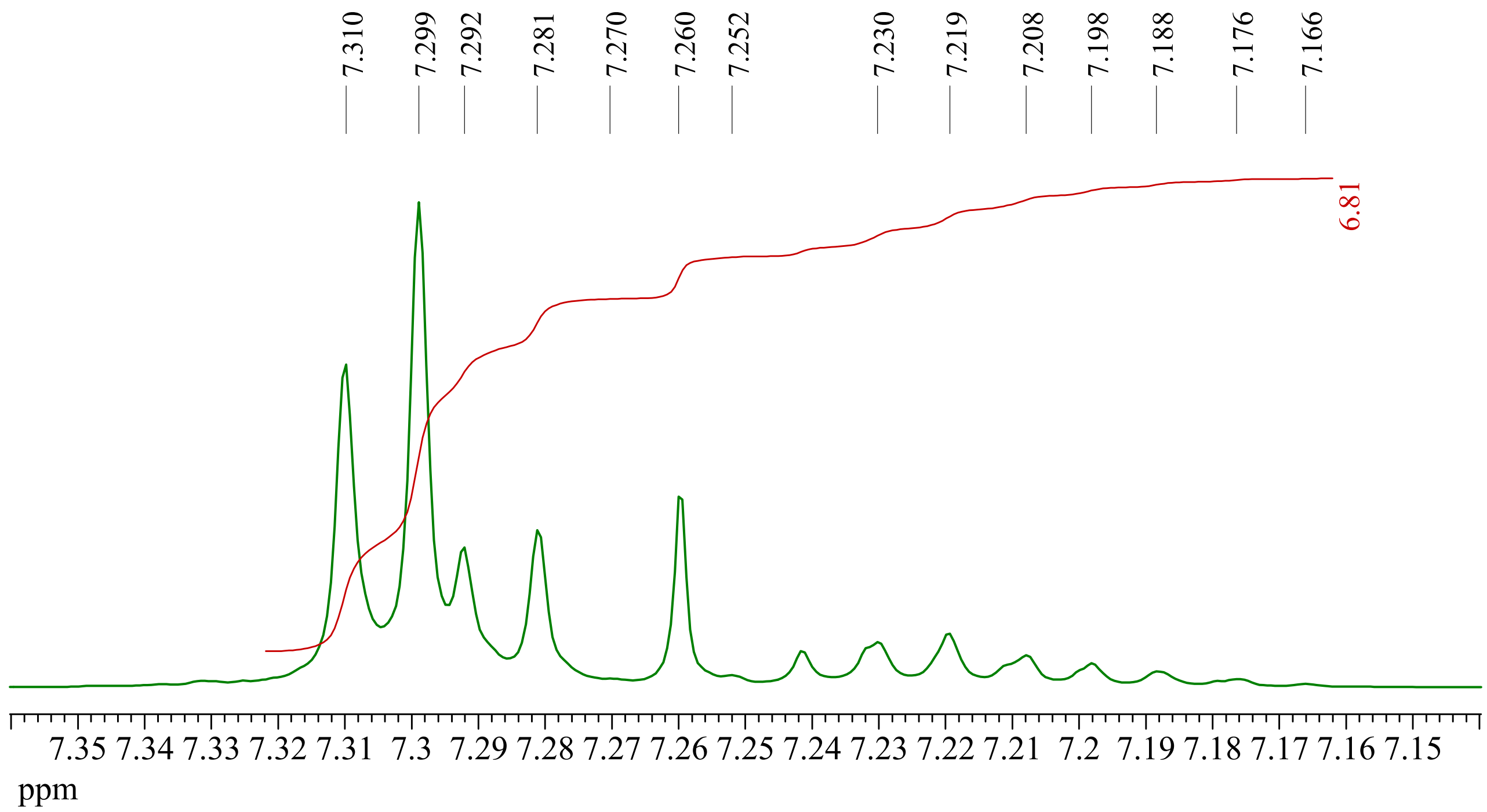

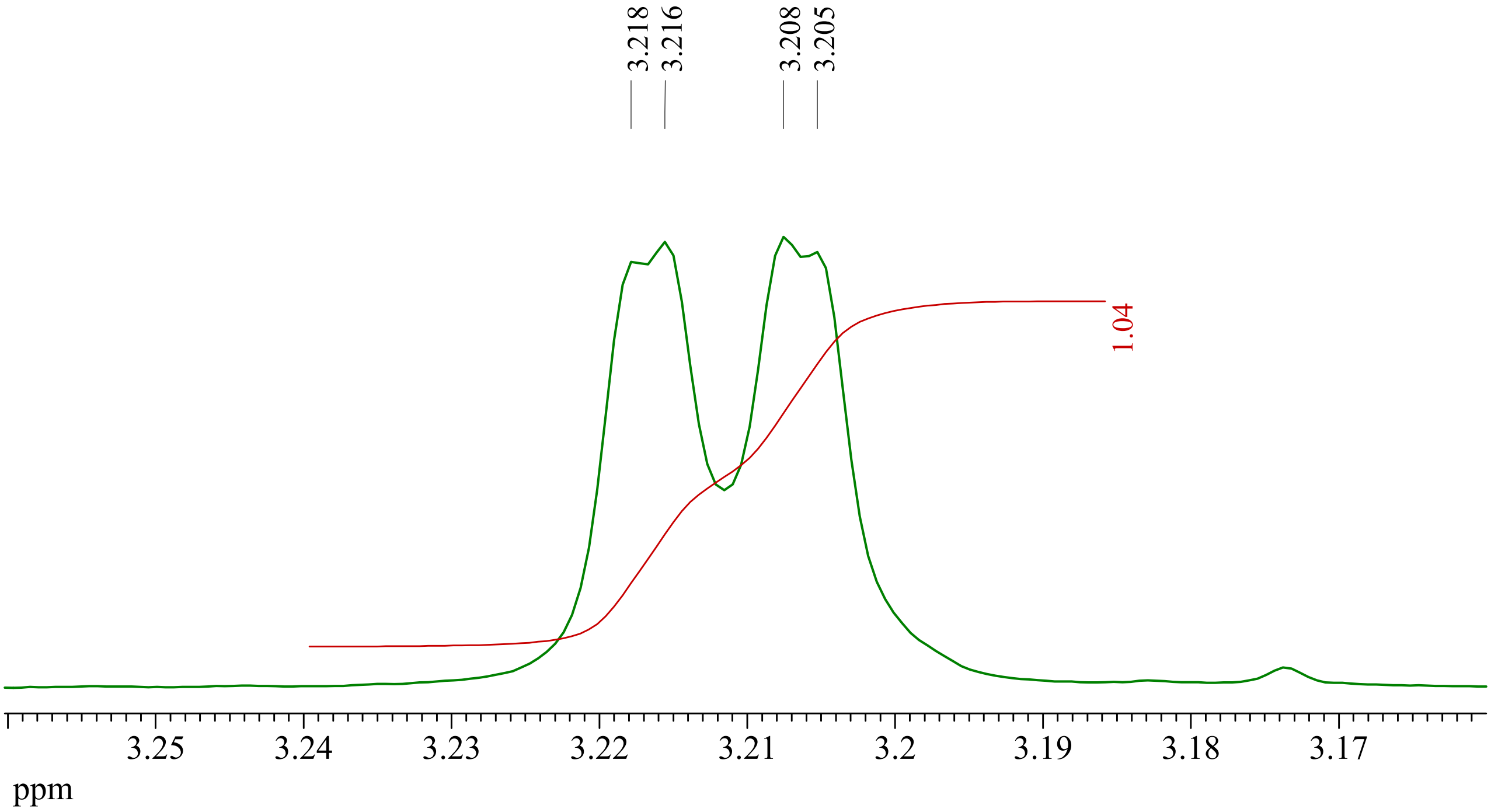

$^{13}C\{^1H\}$ NMR (100 MHz, $CDCl_3$)

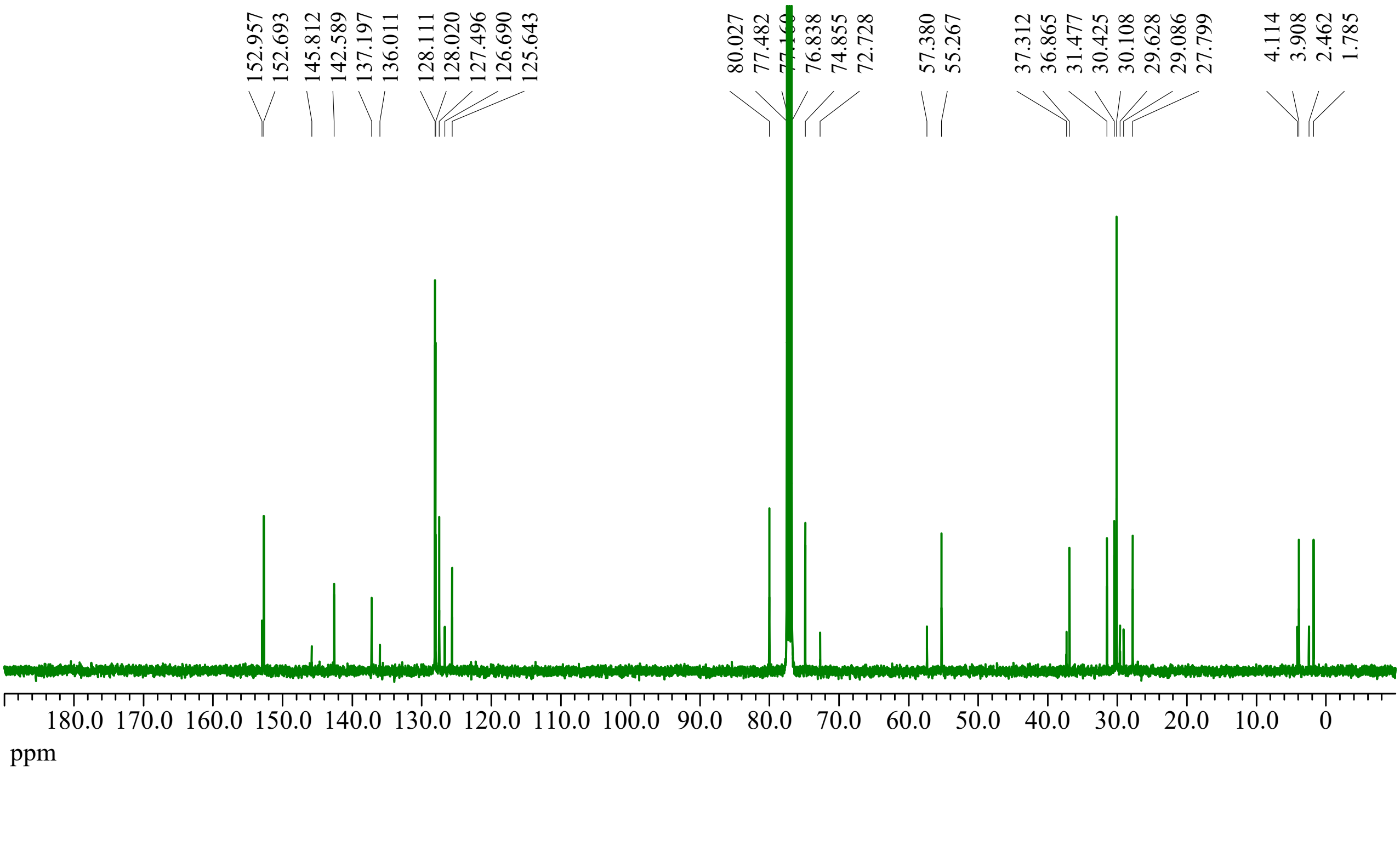

[(*R,*E*)-6-Methoxy-5-{(*R**)-methoxy(phenyl)methyl}-2,2,6-trimethylhept-3-en-4-yl]trimethylgermane** *syn*-*α*-**11**

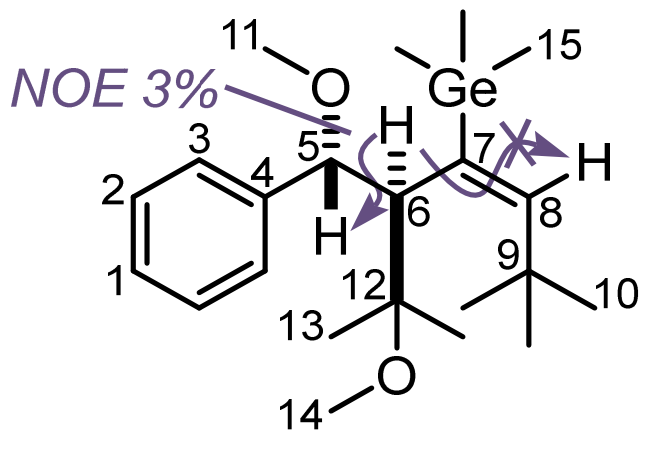

To a mixture of *syn*-*α*-**6** (0.0813 g, 0.214 mmol) in THF (2.1 mL) was added NaH (60% oil dispersion, 0.0343 g, ca. 0.858 mmol) at 0 °C. After the reaction mixture was stirred for 1 h at 0 °C, MeI (0.0801 mL, 1.29 mmol) was added to the mixture. The mixture was stirred 16 h at room temperature and quenched with NH_4Cl aq. The organic phase was extracted with Et_2O and dried over Na_2SO_4. The filtrate was evaporated and the residue was purified by column chromatography on SiO_2 (Hexane/EtOAc = 97:3) to give *syn*-*α*-**11** as colorless neat of the single diastereomer (0.0697 g, 80%).

IR (ATR) 3028 (w), 2964 (s), 1458 (m), 1362 (m), 1227 (m), 1184 (m), 1144 (m), 1095 (s), 824 (s) cm^{-1}; **^{1}H NMR** (400 MHz, $CDCl_3$) 7.31–7.23 (m, 5H), 5.58 (s, 1H, 8-H), 4.50 (d, *J* = 6.4 Hz, 1H, 5-H), 3.53 (d, *J* = 6.4 Hz, 1H, 6-H), 3.09 (s, 3H, 14-H), 2.99 (s, 3H, 11-H), 1.10 (s, 3H, 13-H), 1.00 (s, 9H, 10-H), 0.74 (s, 3H, 13-H), 0.27 (s, 9H, 15-H); **^{13}C{^{1}H} NMR** (100 MHz, $CDCl_3$) 149.3 (d, C-8), 144.0 (s, C-4), 140.4 (s, C-7), 128.4 (d, C-3), 128.1 (d, C-2), 127.2 (d, C-1), 82.1 (d, C-5), 76.2 (s, C-12), 56.2 (d, C-6), 55.5 (q, C-11), 48.5 (q, C-14), 35.7 (s, C-9), 31.8 (q, C-10), 25.1 (q, C-13), 25.0 (q, C-13), 3.0 (q, C-15); **HRMS** (ESI^+) Calculated ($C_{22}H_{38}O_2NaGe$): 431.1976 ($[M+Na]^+$), Found: 431.1992 ($[M+Na]^+$).

^{1}H NMR (400 MHz, $CDCl_3$)

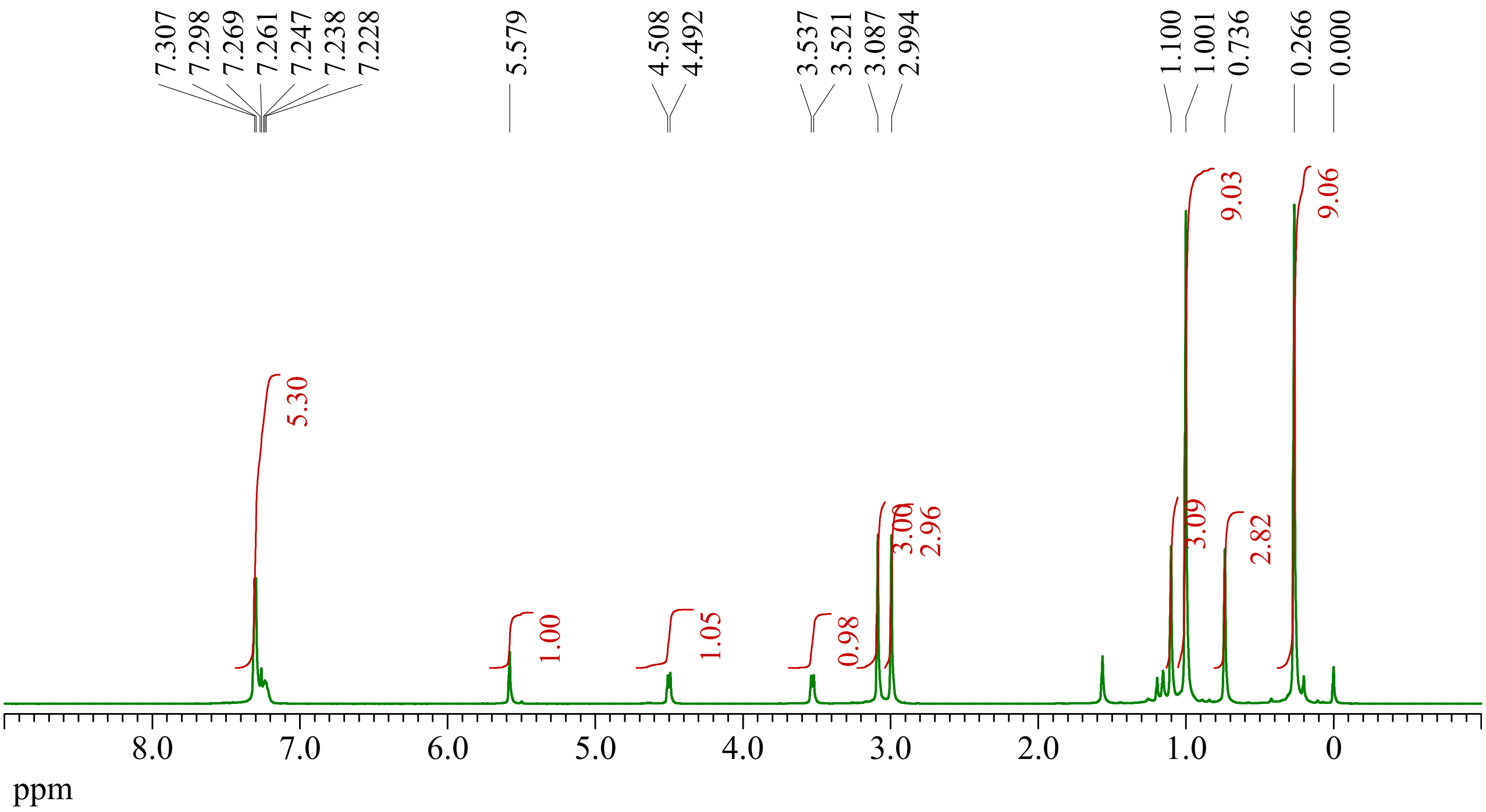

$^{13}C\{^1H\}$ **NMR** (100 MHz, $CDCl_3$)

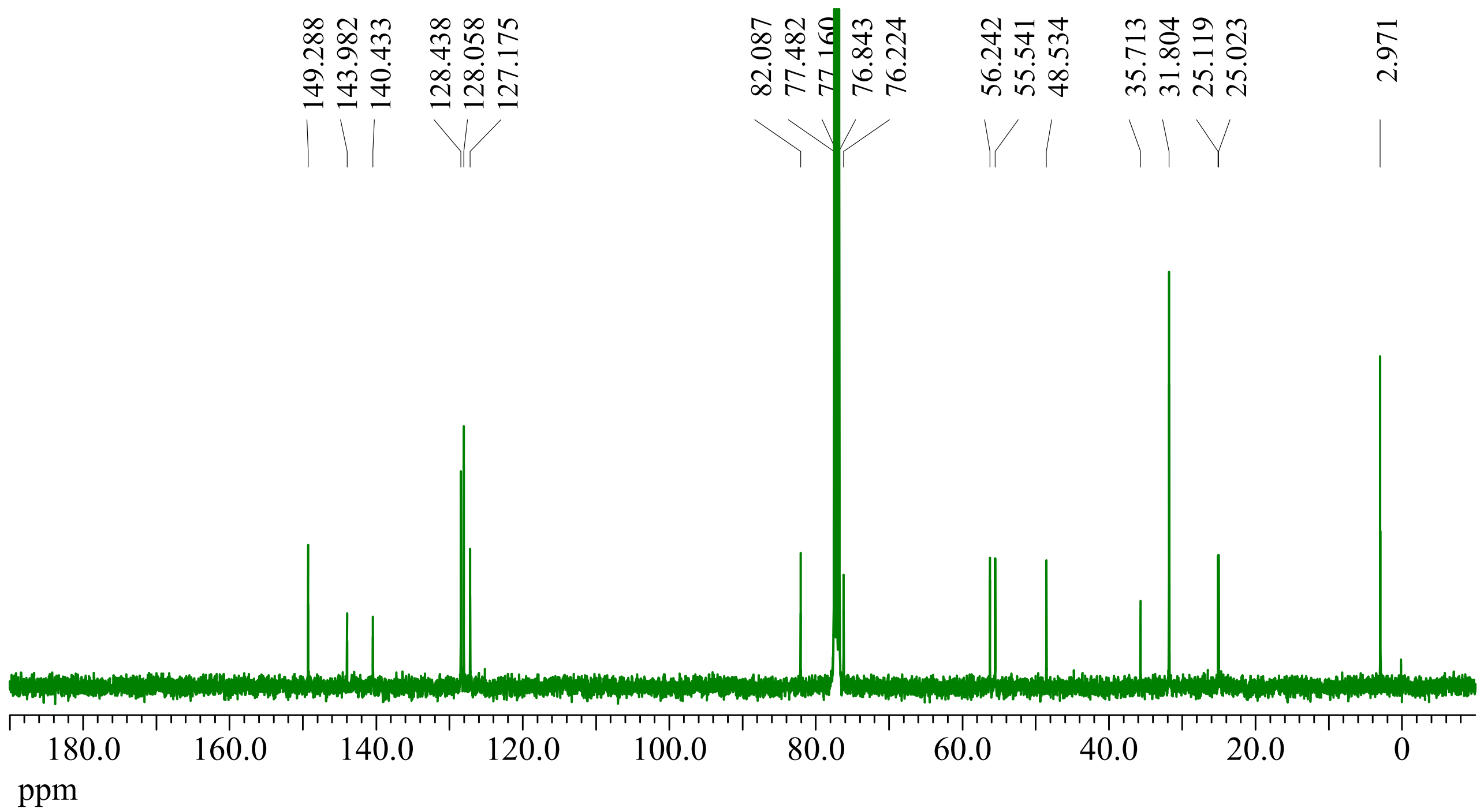

(2*R,3*R**,*E*)-1-(2,2-Dimethylpropylidene)-3-methoxy-2-(2-methoxypropan-2-yl)-2,3-dihydro-1*H*-indene 13**

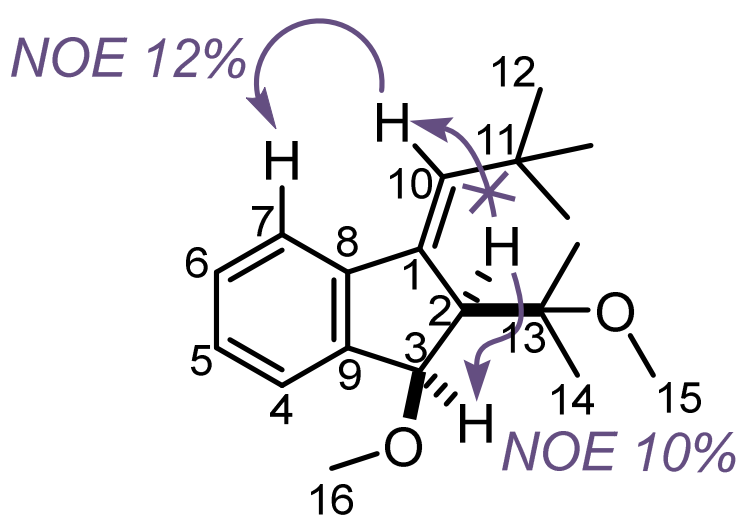

In a nitrogen filled glovebox, [(*R**,*E*)-6-methoxy-5-{(*R**)-methoxy(phenyl)methyl}-2,2,6-trimethylhept-3-en-4-yl]trimethylgermane *syn*-*α*-**11** (0.0406 g, 0.0998 mmol), $(Ph_3P)AuCl$ (2.50 mg, 5.05 μmol) (+)-10-camphorsulfonic acid (0.0345 g, 0.149 mmol) and $PhI(OAc)_2$ (0.0488 g, 0.0152 mmol) were dissolved in 1,4-dioxane (1.0 mL). After the reaction mixture was stirred at 70 °C for 5 h, the solvent was removed under vacuum and the residue was purified by column chromatography on SiO_2 (Hexane/EtOAc = 90:10) to give **13** as colorless neat of the single diastereomer (0.00995 g, 35%).

IR (ATR) 3025 (w), 2952 (s), 1464 (m), 1361 (m), 1245 (m), 1191 (m), 1144 (m), 1119 (s), 1078 (s) cm^{-1}; ^{1}**H NMR** (400 MHz, $CDCl_3$) 7.34 (d, *J* = 7.2 Hz, 1H, 7-H), 7.28 (d, *J* = 7.2 Hz, 1H, 4-H), 7.21–7.15 (m, 2H, 6-H, 5-H), 5.85 (s, 1H, 10-H), 4.76 (d, *J* = 6.4 Hz, 1H, 3-H), 3.84 (d, *J* = 6.4 Hz, 1H, 2-H), 3.65 (s, 3H, 16-H), 3.11 (s, 3-H, 15-H), 1.214 (s, 9H, 12-H), 1.209 (s, 3H, 14-H), 0.75 (s, 3H, 14-H); $^{13}C\{^1H\}$ **NMR** (100 MHz, $CDCl_3$) 143.7 (s, C-9), 143.4 (s, C-8), 136.5 (d, C-10), 134.3 (s, C-1), 127.7 (d, C-6), 127.0 (d, C-5), 122.5 (d, C-4), 119.0 (d, C-7), 86.3 (d, C-3), 77.3 (s, C-13), 60.1 (q, C-16), 54.3 (d, C-2), 48.1 (q, C-15), 34.9 (s, C-11), 30.9 (q, C-12), 25.1 (q, C-14), 21.9 (q, C-14); **HRMS**: (CI^+, 70 eV) Calculated ($C_{19}H_{29}O_2$): 289.2168 ($[M+H]^+$), Found: 289.2165 ($[M+H]^+$).

^{1}**H NMR** (400 MHz, $CDCl_3$)

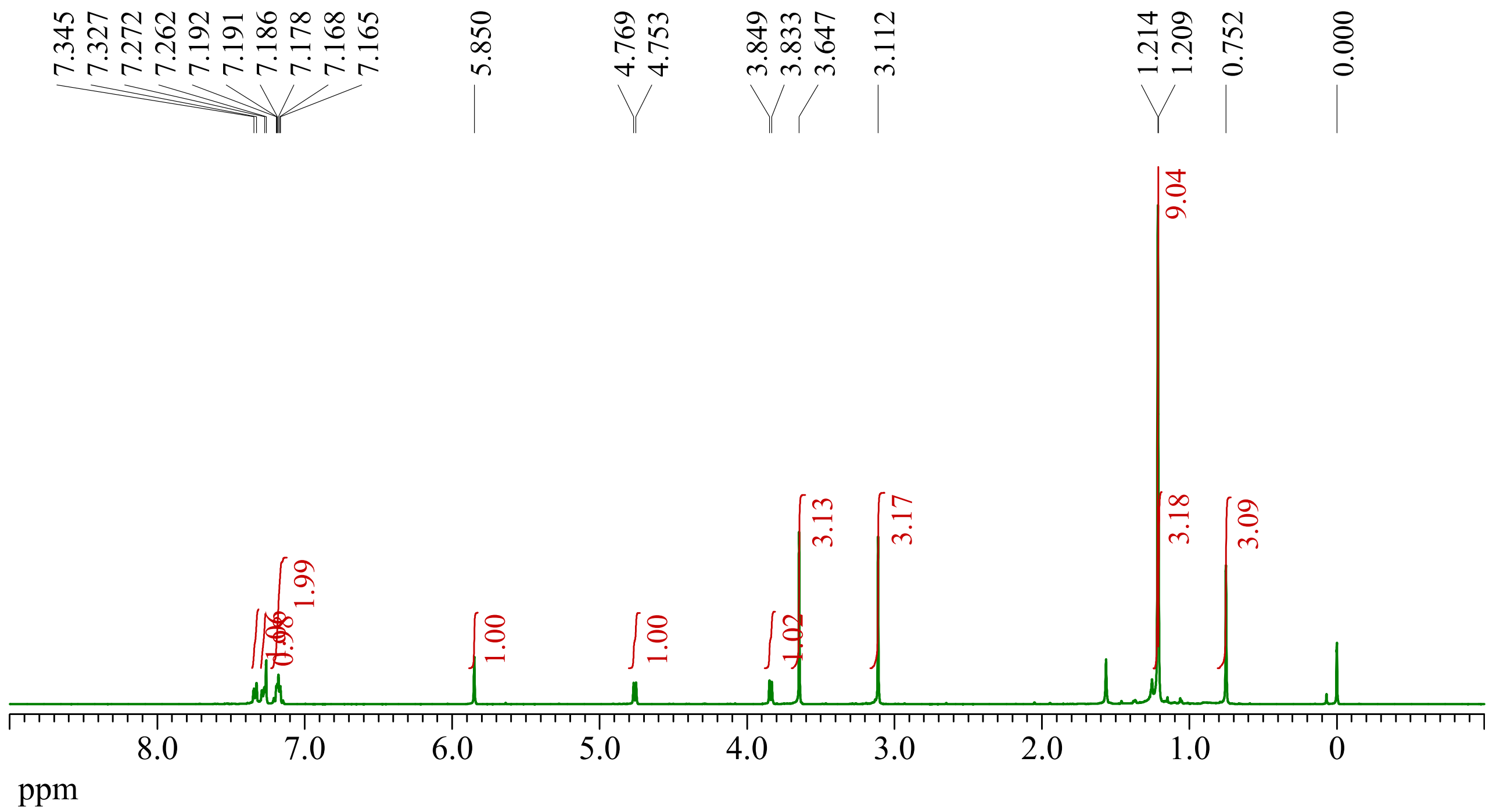

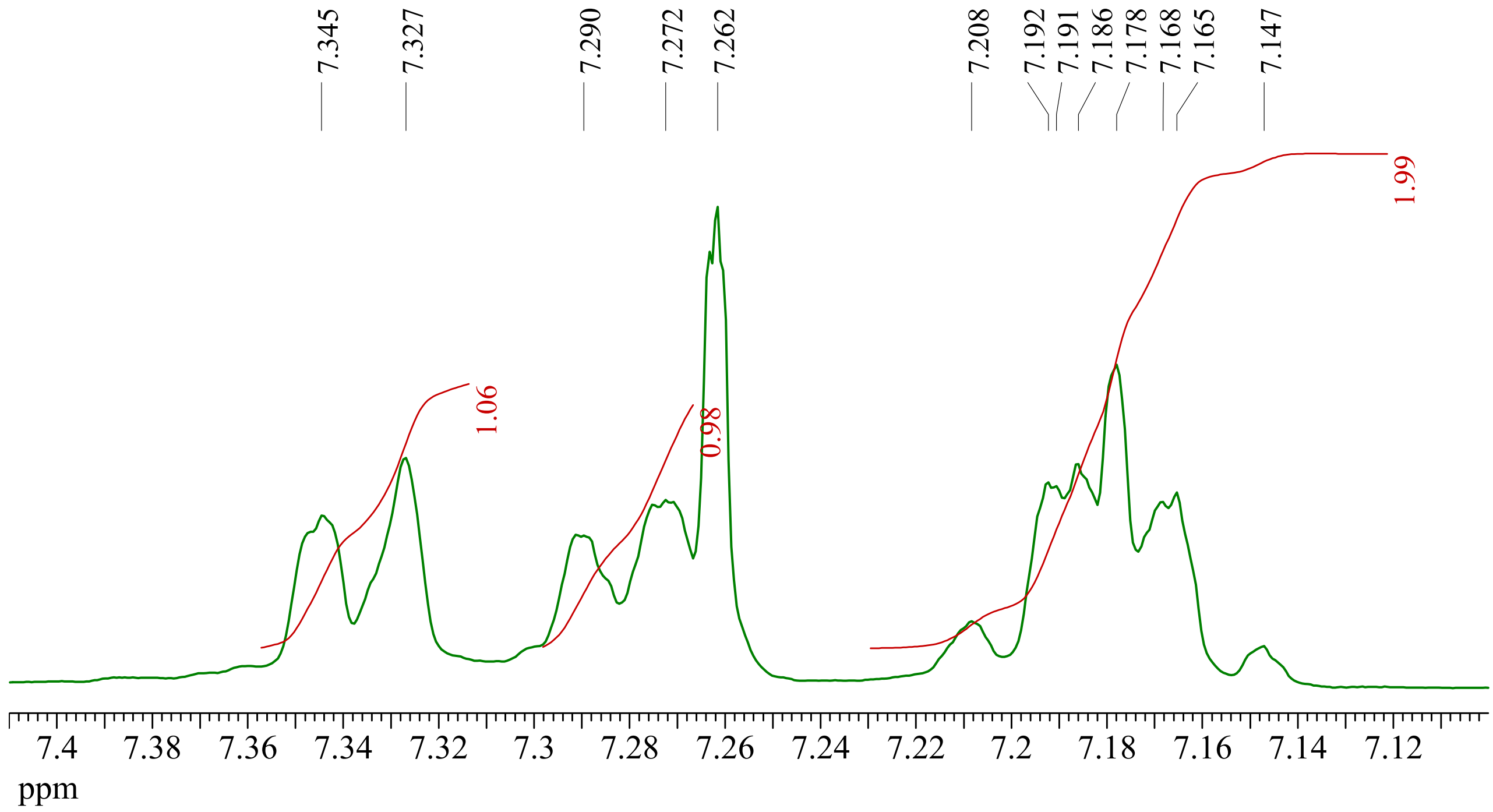

$^{13}C\{^{1}H\}$ NMR (100 MHz, $CDCl_3$)

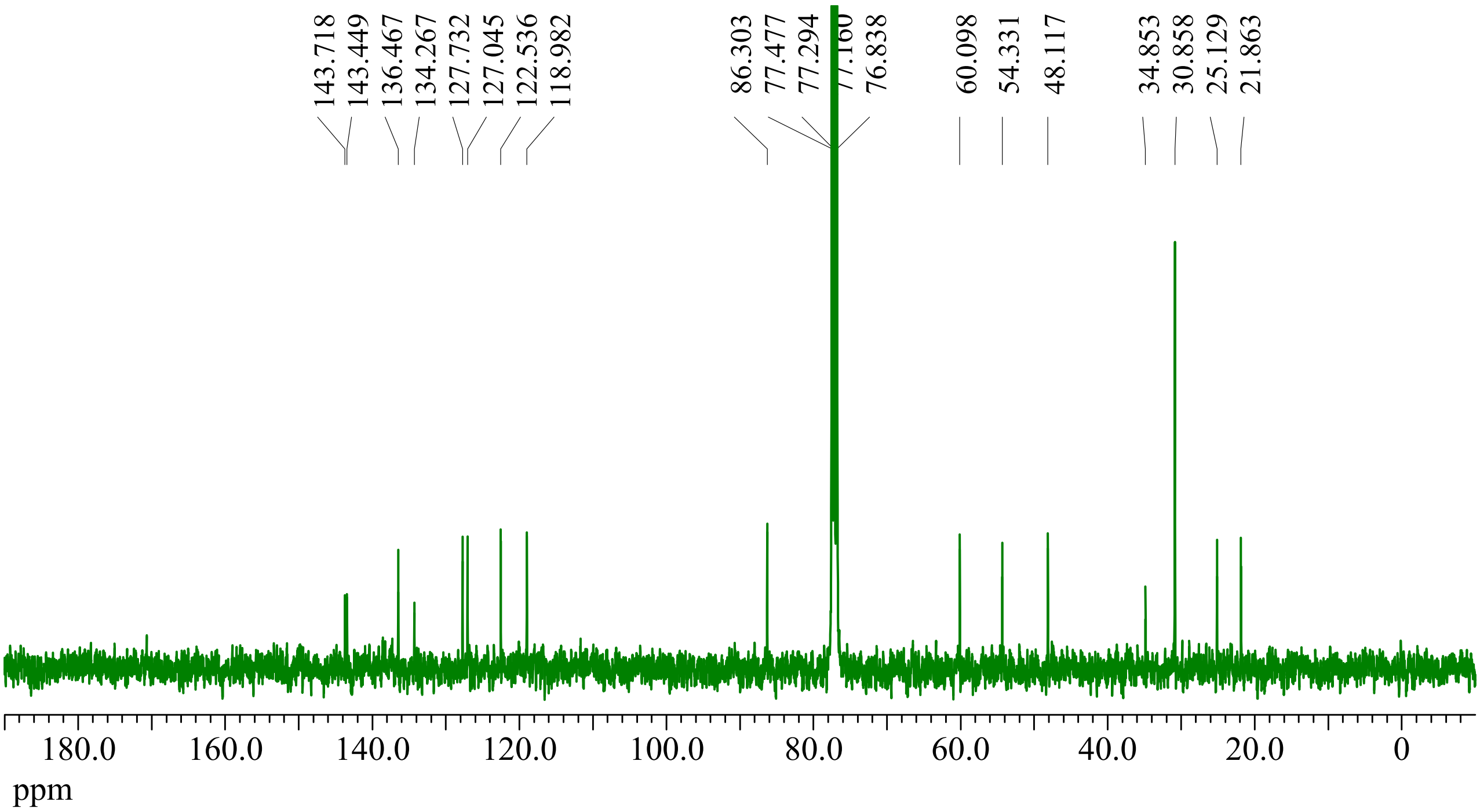

3-11. Synthesis of starting allenoates 1

Benzyl 5,5-dimethylhexa-2,3-dienoate 1c[2,8]

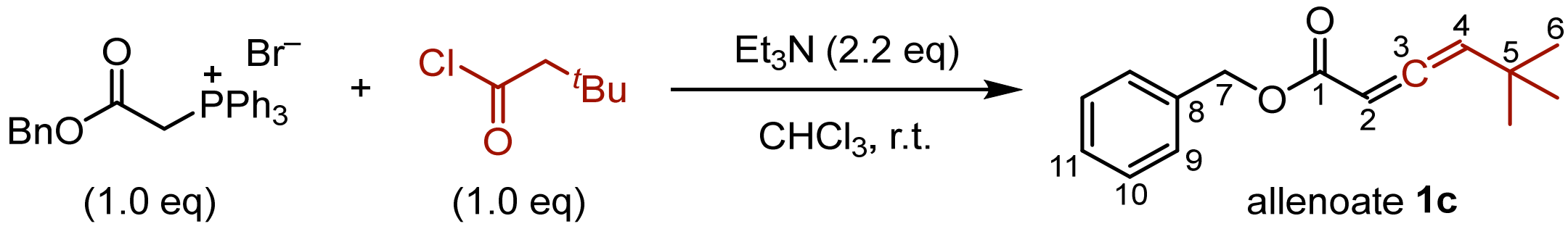

To a mixture of {2-(benzyloxy)-2-oxoethyl}triphenylphosphonium bromide (2.29 g, 4.66 mmol) in $CHCl_3$ (7.8 mL) was slowly added a solution of NEt_3 (1.43 mL, 10.2 mmol) in $CHCl_3$ (7.8 mL). The resulting solution was cooled to 0 °C, then a solution of 3,3-dimethylbutanoyl chloride (0.647 mL, 4.66 mmol) in $CHCl_3$ (7.8 mL) was added dropwise. After the reaction mixture was stirred at room temperature for 18 h, the solvent was removed under vacuum. The residue was purified by column chromatography on SiO_2 (Hexane/EtOAc = 90:10) to give benzyl 5,5-dimethylhexa-2,3-dienoate as colorless neat (0.869 g, 81%).

IR (ATR) 3034 (w), 2961 (m), 1957 (m), 1716 (s), 1459 (m), 1366 (m), 1236 (s), 1145 (s), 1007 (m) cm^{-1}; **^{1}H NMR** (400 MHz, $CDCl_3$) 7.36–7.28 (m, 5H), 5.68 (d, J = 5.6 Hz, 1H, 2-H), 5.61 (d, J = 6.0 Hz, 1H, 4-H), 5.22 (d, J = 12.8 Hz, 1H, 7-H), 5.12 (d, J = 12.8 Hz, 1H, 7-H), 1.10 (s, 9H, 6-H); **^{13}C{^{1}H} NMR** (100 MHz, $CDCl_3$) 210.9 (s, C-3), 166.1 (s, C-1), 136.2 (s, C-8), 128.6 (d, C-10), 128.2 (d, C-11), 128.0 (d, C-9), 107.0 (d, C-4), 89.6 (d, C-2), 66.4 (t, C-7), 32.8 (s, C-5), 30.1 (q, C-6); **HRMS** (ESI^{+}) Calculated ($C_{15}H_{18}O_2Na$): 253.1199 ([M+Na]$^{+}$), Found: 253.1204 ([M+Na]$^{+}$).

^{1}H NMR (400 MHz, $CDCl_3$)

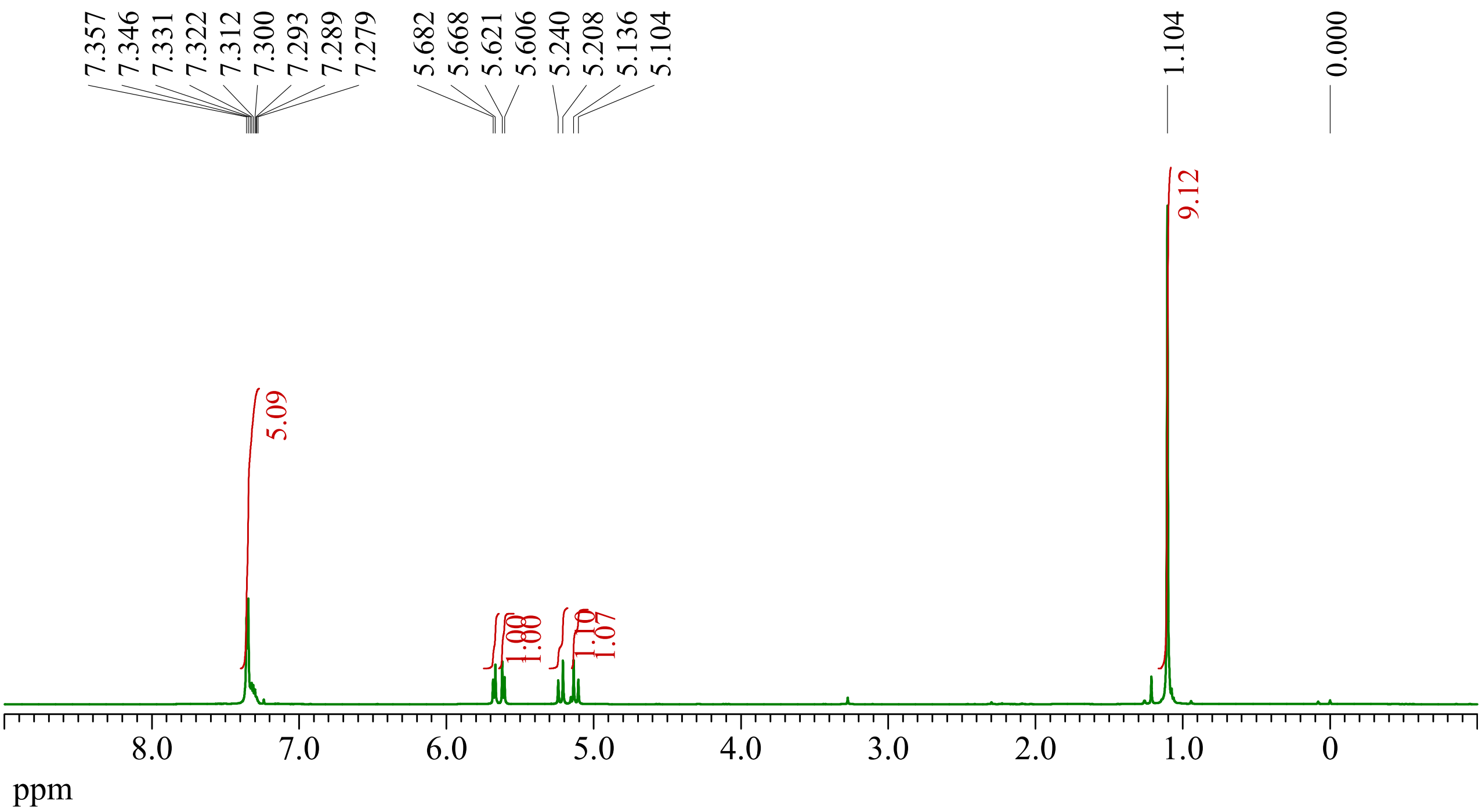

$^{13}C\{^{1}H\}$ NMR (100 MHz, $CDCl_3$)

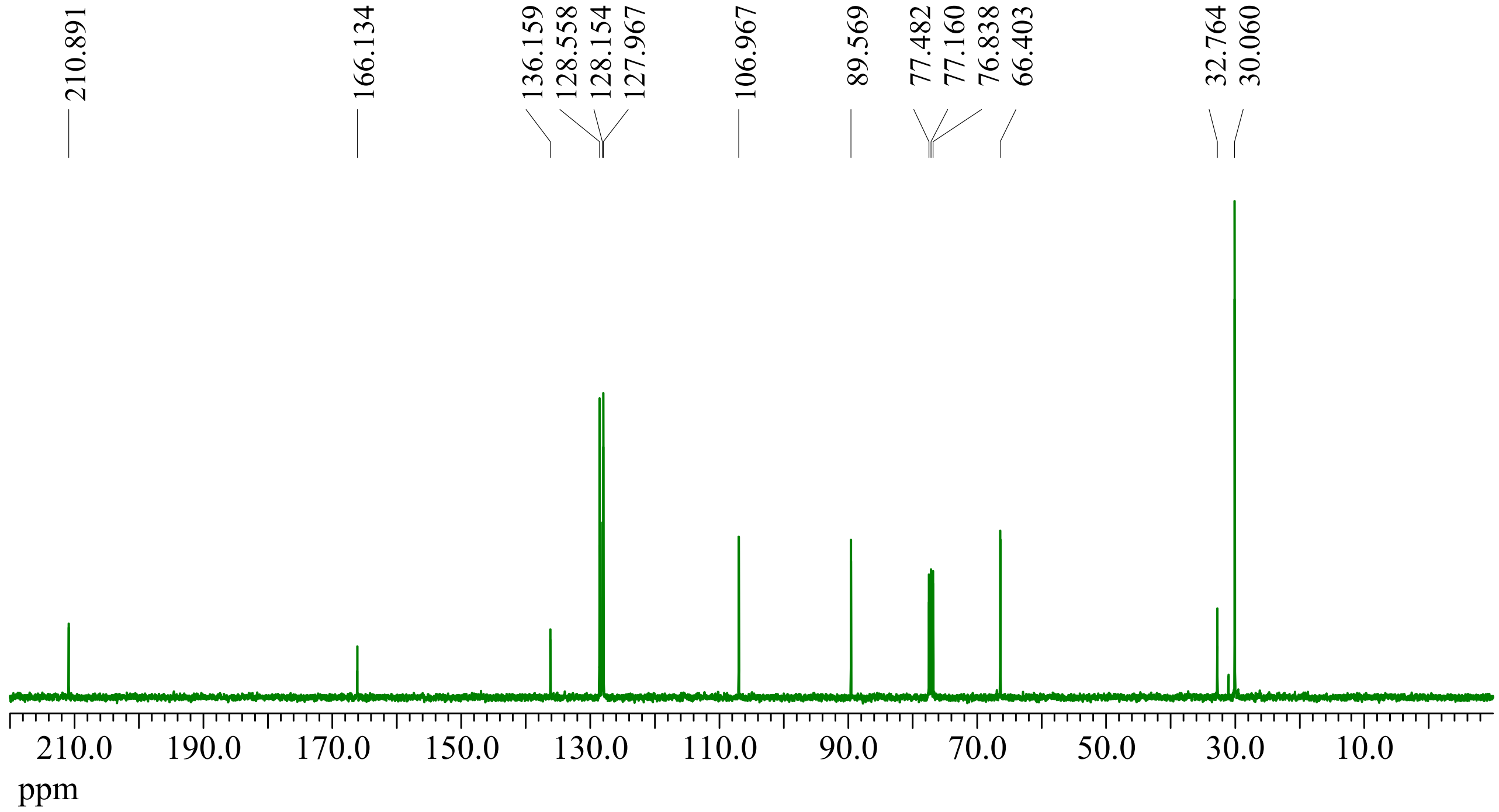

4. X-ray crystallographic data

4-1. Diethyl (3*E*,4*R**,5*R**,8*E*,9*S**,10*S**)-2,2,7,7-tetrachloro-3,8-bis(2,2-dimethylpropylidene)-5,10-diphenyl-1,6,2,7-dioxadigermecane-4,9-dicarboxylate *syn*-*α*-4aa

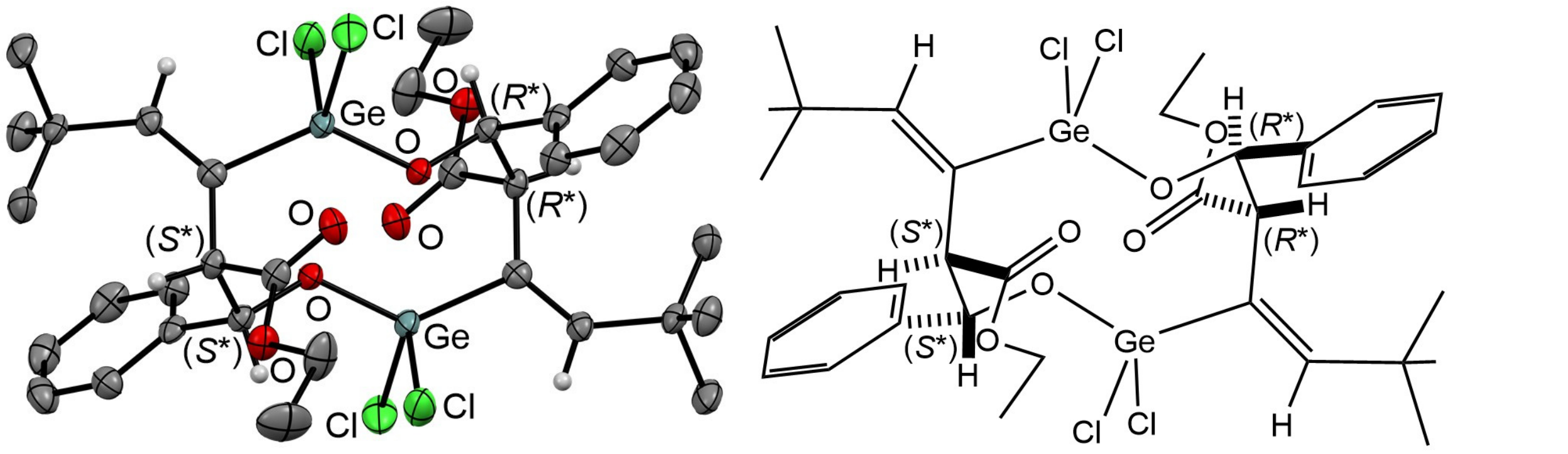

Figure S1. ORTEP drawings of *syn*-*α*-**4aa** at the 50% probability level.

No. CCDC	2548107	Space Group	$P2_1/c$ (#14)
Empirical Formula	$C_{34}H_{44}Cl_4Ge_2O_6$	Z value	2
Formula Weight	835.67	D_{calc}	1.494 g/cm^3
Crystal Color	colorless	F_{000}	856.0
Crystal Dimensions	0.12 × 0.1 × 0.04 mm	μ(CuK$_\alpha$)	4.979 mm^{-1}
Crystal System	monoclinic	Temperature	123 K
Lattice Type	Primitive	Data/restraints/parameters	3672/0/212
Lattice Parameters	a = 8.8643(2) Å	Residuals: R_1 ($I > 2.00\sigma(I)$)	0.0540
	b = 18.0654(3) Å	Residuals: wR_2 (all data)	0.1496
	c = 11.8551(3) Å	Goodness of Fit Indicator	1.039
	α = 90 °		
	β = 101.907(2) °		
	γ = 90 °		
	V = 1857.59(7) Å^3		

4-2. Diethyl (3*E*,4*R**,5*R**,8*E*,9*S**,10*S**)-2,2,7,7-tetrachloro-3,8-bis(2,2-dimethylpropylidene)-5,10-bis{4-(methoxycarbonyl)phenyl}-1,6,2,7-dioxadigermecane-4,9-dicarboxylate *syn*-*α*-4ac

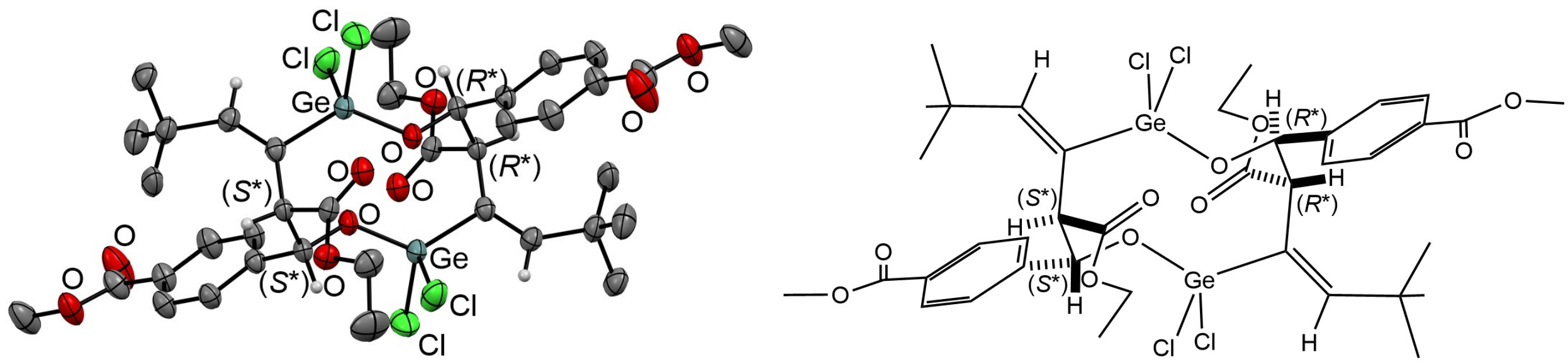

Figure S2. ORTEP drawings of *syn*-*α*-**4ac** at the 50% probability level.

No. CCDC	2548108	Space Group	P-1 (#2)
Empirical Formula	$C_{38}H_{48}Cl_4Ge_2O_{10}$	Z value	1
Formula Weight	951.74	D_{calc}	1.417 g/cm^3
Crystal Color	colorless	F_{000}	488.0
Crystal Dimensions	0.18 × 0.15 × 0.13 mm	μ(CuK$_\alpha$)	4.287 mm^{-1}
Crystal System	triclinic	Temperature	123 K
Lattice Type	Primitive	Data/restraints/parameters	4226/0/249
Lattice Parameters	a = 9.1244(6) Å	Residuals: R_1 ($I > 2.00\sigma(I)$)	0.0697
	b = 10.9314(6) Å	Residuals: wR_2 (all data)	0.2471
	c = 11.7769(5) Å	Goodness of Fit Indicator	1.016
	α = 102.296(4) °		
	β = 99.100(5) °		
	γ = 97.836(5) °		
	V = 1115.53(11) Å^3		

4-3. Diethyl (3*E*,4*R,5*R**,8*E*,9*S**,10*S**)-2,2,7,7-tetrachloro-3,8-bis(2,2-dimethylpropylidene)-5,10-di-*m*-tolyl-1,6,2,7-dioxadigermecane-4,9-dicarboxylate *syn*-*α*-4ae**

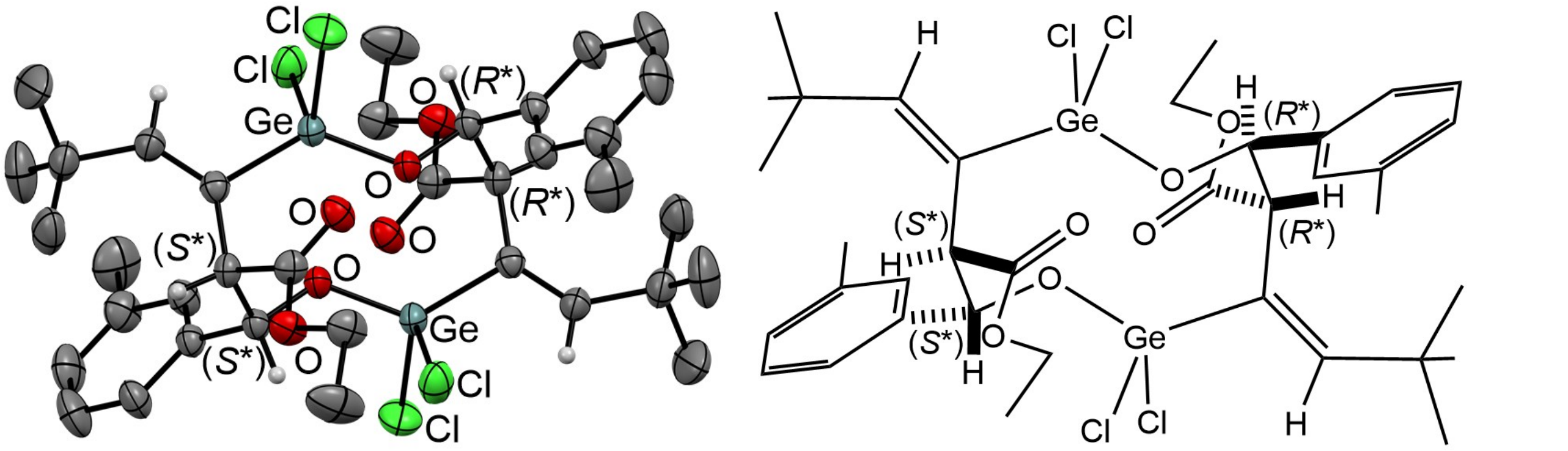

Figure S3. ORTEP drawings of *syn*-*α*-**4ae** at the 50% probability level.

No. CCDC	2548109	Space Group	$P2_1/c$ (#14)
Empirical Formula	$C_{36}H_{48}Cl_4Ge_2O_6$	Z value	2
Formula Weight	863.72	D_{calc}	1.427 g/cm^3
Crystal Color	colorless	F_{000}	888.0
Crystal Dimensions	0.32 × 0.18 × 0.12 mm	μ(CuK_α)	4.620 mm^{-1}
Crystal System	monoclinic	Temperature	123 K
Lattice Type	Primitive	Data/restraints/parameters	3843/21/253
Lattice Parameters	a = 12.6232(6) Å	Residuals: R_1 ($I > 2.00\sigma(I)$)	0.0566
	b = 11.4776(5) Å	Residuals: wR_2 (all data)	0.1559
	c = 14.3643(6) Å	Goodness of Fit Indicator	1.078
	α = 90 °		
	β = 105.016(4) °		
	γ = 90 °		
	V = 2010.09(16) Å^3		

4-4. Diethyl (3*E*,4*R,5*R**,8*E*,9*S**,10*S**)-2,2,7,7-tetrachloro-3,8-bis(2,2-dimethylpropylidene)-5,10-di(naphthalen-1-yl)-1,6,2,7-dioxadigermecane-4,9-dicarboxylate *syn*-*α*-4ah**

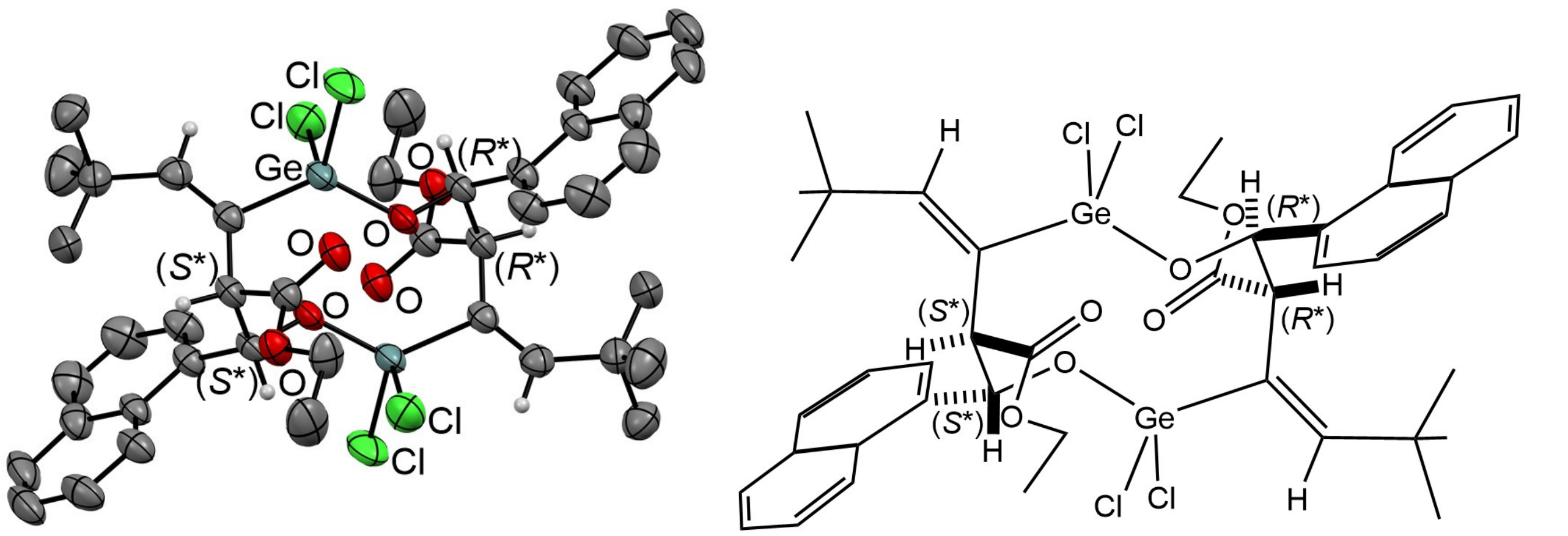

Figure S4. ORTEP drawings of *syn*-*α*-**4ah** at the 50% probability level.

No. CCDC	2548110	Space Group	$P2_1/n$ (#14)
Empirical Formula	$C_{42}H_{48}Cl_4Ge_2O_6$	Z value	2
Formula Weight	935.78	D_{calc}	1.452 g/cm^3
Crystal Color	colorless	F_{000}	960.0
Crystal Dimensions	0.09 × 0.08 × 0.06 mm	μ(CuK_α)	4.390 mm^{-1}
Crystal System	monoclinic	Temperature	123 K
Lattice Type	Primitive	Data/restraints/parameters	4126/0/248
Lattice Parameters	a = 11.0335(3) Å	Residuals: R_1 ($I > 2.00\sigma(I)$)	0.0596
	b = 13.4972(4) Å	Residuals: wR_2 (all data)	0.2588
	c = 14.5491(4) Å	Goodness of Fit Indicator	1.069
	α = 90 °		
	β = 99.045(3) °		
	γ = 90 °		
	V = 2139.73(11) Å^3		

4-5. Ethyl (4*R**,5*S**,*E*)-2,2-dichloro-3-(2,2-dimethylpropylidene)-5-phenyl-1,2-oxagermolane-4-carboxylate *anti*-*α*-4aa

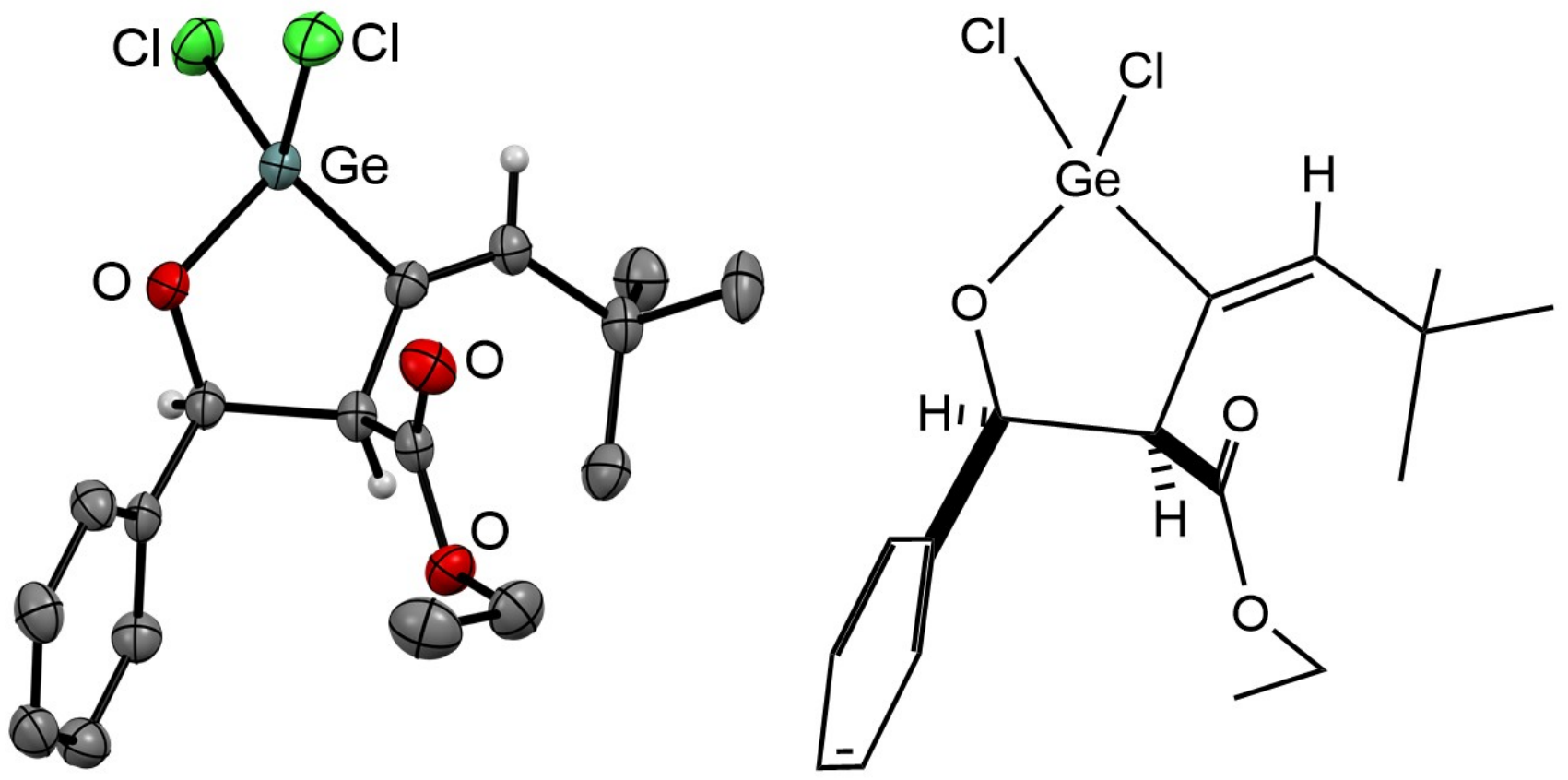

Figure S5. ORTEP drawings of *anti*-*α*-**4aa** at the 50% probability level.

No. CCDC	2548111	Space Group	*C*2/*c* (#15)
Empirical Formula	$C_{17}H_{22}Cl_2GeO_3$	*Z* value	8
Formula Weight	417.83	D_{calc}	1.487 g/cm^3
Crystal Color	colorless	F_{000}	1712.0
Crystal Dimensions	0.17 × 0.12 × 0.1 mm	μ(CuK_α)	4.955 mm^{-1}
Crystal System	monoclinic	Temperature	123 K
Lattice Type	C-centered	Data/restraints/parameters	3688/0/212
Lattice Parameters	a = 20.8910(5) Å	Residuals: R_1 ($I > 2.00\sigma(I)$)	0.0312
	b = 11.4733(3) Å	Residuals: wR_2 (all data)	0.0852
	c = 16.2977(4) Å	Goodness of Fit Indicator	1.061
	α = 90 °		
	β = 107.100(2) °		
	γ = 90 °		
	V = 3733.68(17) Å^3		

4-6. Ethyl (*Z*)-2-{(4*R**,5*S**)-4-(*tert*-butyl)-2,2-dichloro-5-phenyl-1,2-oxagermolan-3-ylidene)acetate *anti*-*γ*-4aa

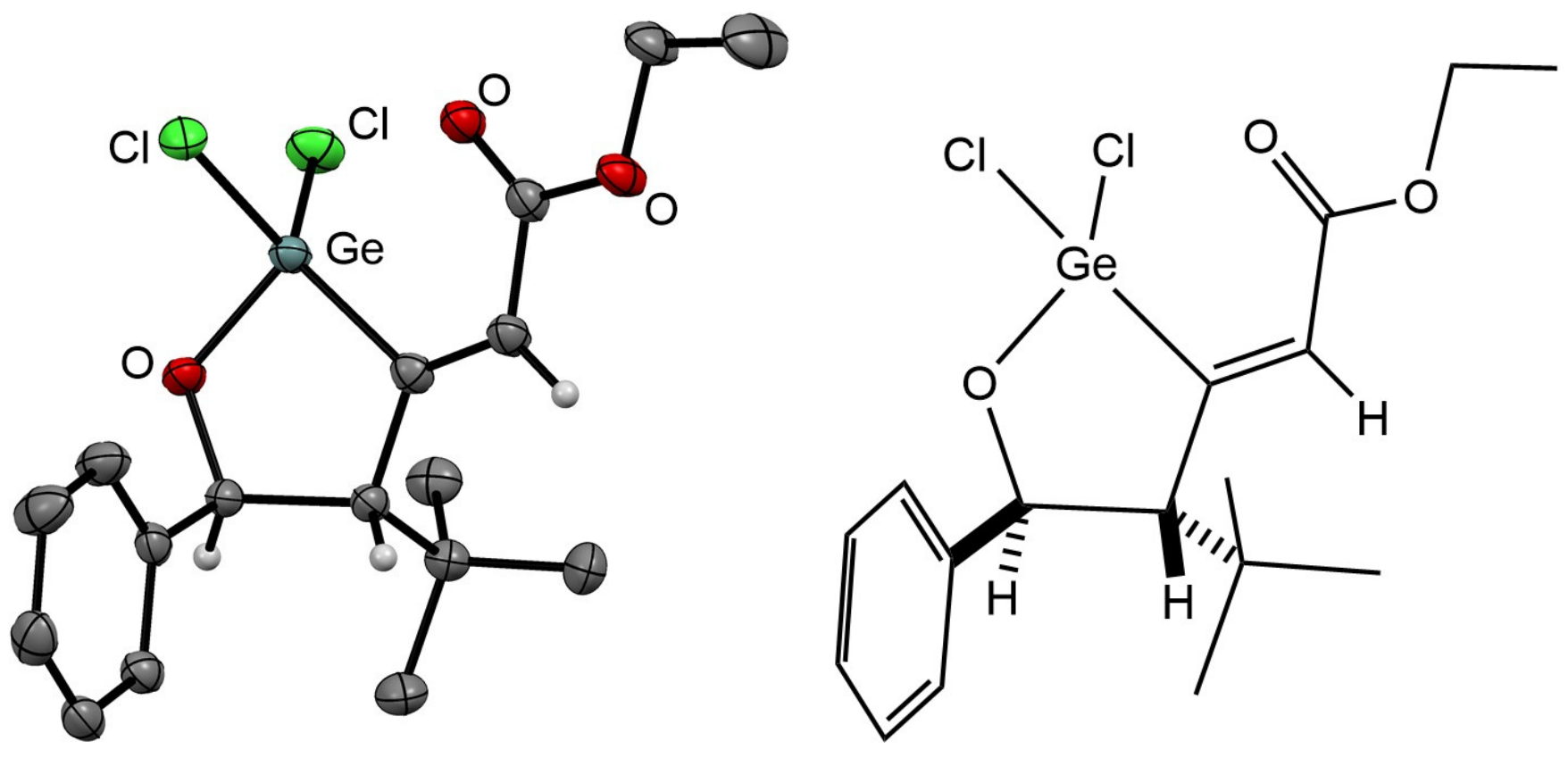

Figure S6. ORTEP drawings of *anti*-*γ*-**4aa** at the 50% probability level.

No. CCDC	2548112	Space Group	*P*-1 (#2)
Empirical Formula	$C_{17}H_{22}Cl_2GeO_3$	*Z* value	2
Formula Weight	417.89	D_{calc}	1.487 g/cm^3
Crystal Color	colorless	F_{000}	428.0
Crystal Dimensions	0.09 X 0.09 X 0.05 mm	μ(CuK_α)	4.957 mm^{-1}
Crystal System	Triclinic	Temperature	123 K
Lattice Type	Primitive	Data/restraints/parameters	3718/0/212
Lattice Parameters	a = 8.6531(3) Å	Residuals: R_1 ($I > 2.00\sigma(I)$)	0.0294
	b = 10.2562(3) Å	Residuals: wR_2 (all data)	0.0755
	c = 10.8893(4) Å	Goodness of Fit Indicator	1.063
	α = 83.166(3) °		
	β = 86.213(3) °		
	γ = 76.714(3) °		
	V = 933.11(6) Å^3		

4-7. Ethyl (*R,*Z*)-4-{(*S**)-hydroxy(phenyl)methyl}-5,5-dimethyl-3-(trimethylgermyl)hex-2-enoate *anti*-γ-5**

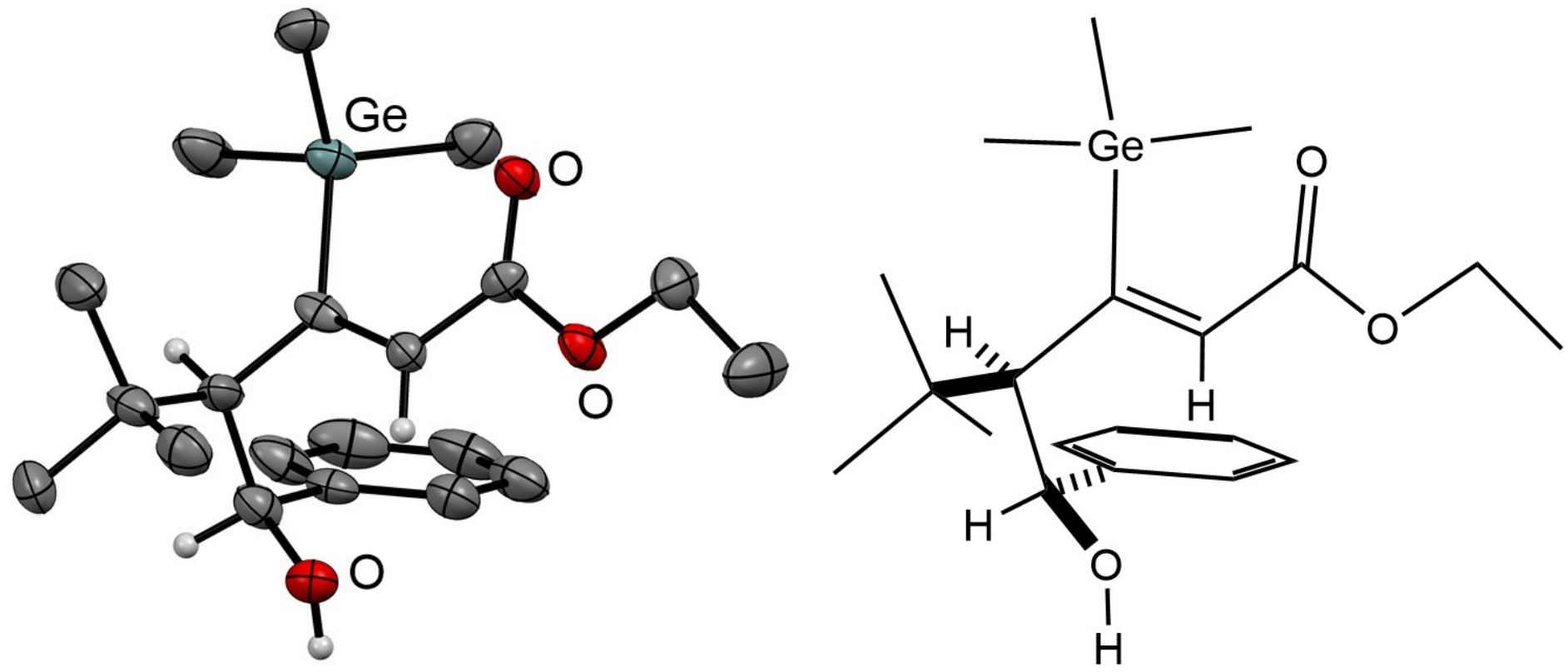

Figure S7. ORTEP drawings of *anti*-γ-**5** at the 50% probability level.

No. CCDC	2548113	Space Group	*C*2/*c* (#15)
Empirical Formula	$C_{20}H_{32}GeO_3$	*Z* value	32
Formula Weight	393.04	D_{calc}	1.253 g/cm^3
Crystal Color	colorless	F_{000}	6656.0
Crystal Dimensions	0.13 X 0.05 X 0.02 mm	μ(CuK$_\alpha$)	2.096 mm^{-1}
Crystal System	monoclinic	Temperature	123 K
Lattice Type	C-centered	Data/restraints/parameters	16516/0/897
Lattice Parameters	a = 50.332(4) Å	Residuals: R_1 ($I > 2.00\sigma(I)$)	0.0825
	b = 9.3689(5) Å	Residuals: wR_2 (all data)	0.2656
	c = 38.203(3) Å	Goodness of Fit Indicator	0.998
	α = 90 °		
	β = 112.268(9) °		
	γ = 90 °		
	V = 16672(2) Å^3		

4-8. (1*R,2*S**,*Z*)-2-(*tert*-Butyl)-5-methyl-1-phenyl-3-(trimethylgermyl)hex-3-ene-1,5-diol *anti*-γ-6**

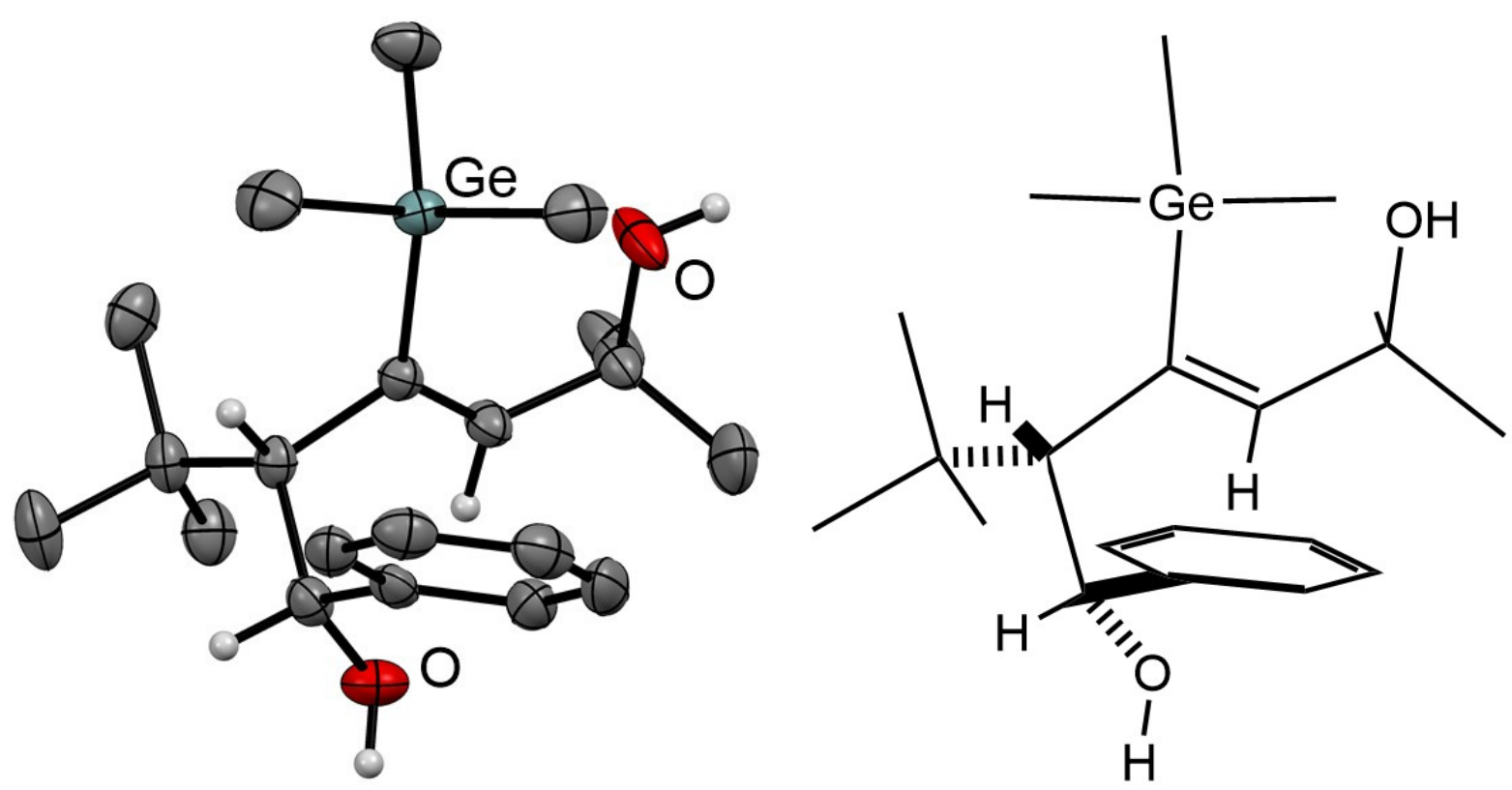

Figure S8. ORTEP drawings of *anti*-γ-**6** at the 50% probability level.

No. CCDC	2548114	Space Group	*P*2$_1$/*c* (#14)
Empirical Formula	$C_{20}H_{34}GeO_2$	*Z* value	8
Formula Weight	379.06	D_{calc}	1.201 g/cm^3
Crystal Color	colorless	F_{000}	1616.0
Crystal Dimensions	0.08 X 0.07 X 0.04 mm	μ(CuK$_\alpha$)	2.026 mm^{-1}
Crystal System	monoclinic	Temperature	123 K
Lattice Type	Primitive	Data/restraints/parameters	8274/0/435
Lattice Parameters	a = 18.1127(4) Å	Residuals: R_1 ($I > 2.00\sigma(I)$)	0.0433
	b = 15.3224(3) Å	Residuals: wR_2 (all data)	0.1307
	c = 16.5867(4) Å	Goodness of Fit Indicator	1.102
	α = 90 °		
	β = 114.371(3) °		
	γ = 90 °		
	V = 4193.12(18) Å^3		

4-9. [(*R,*Z*)-2-Methoxy-5-{(*S**)-methoxy(phenyl)methyl}-2,6,6-trimethylhept-3-en-4-yl]trimethylgermane**

anti-*γ*-**11**

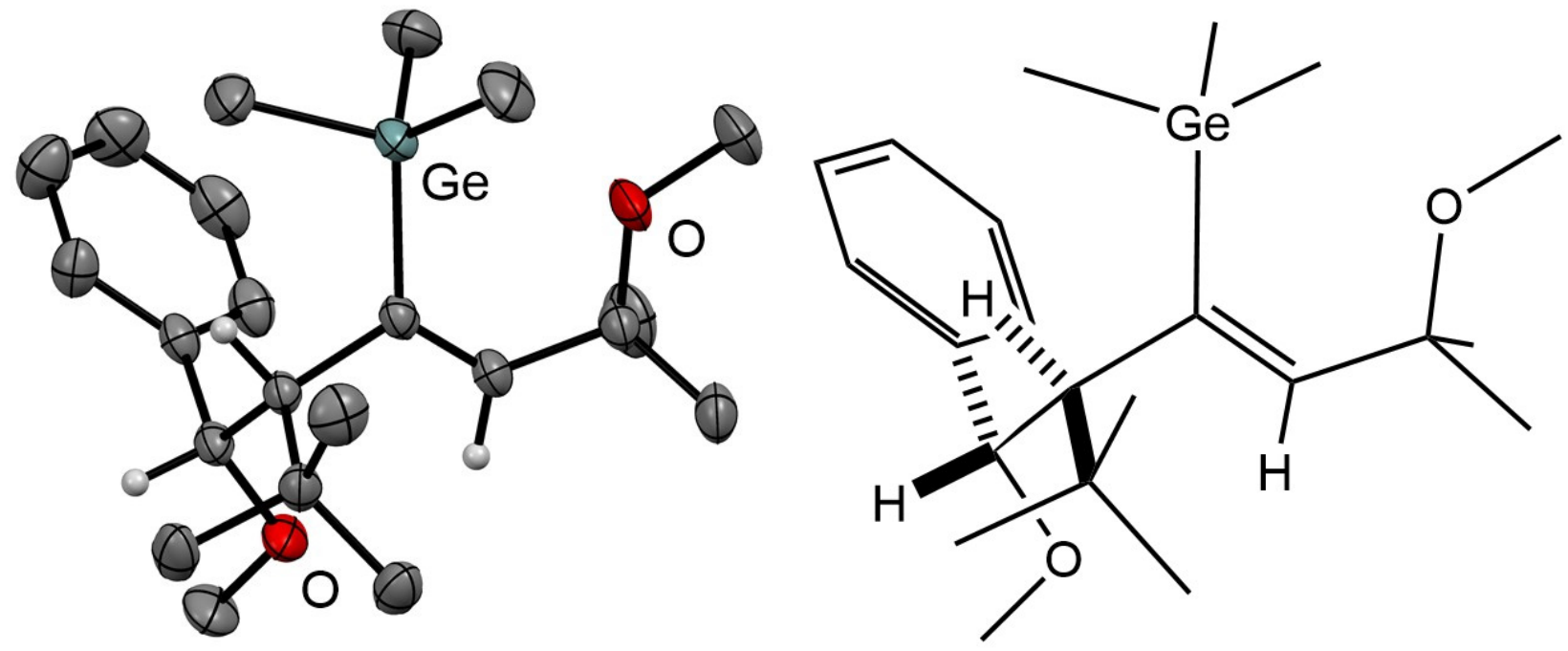

Figure S9. ORTEP drawings of *anti*-*γ*-**11** at the 50% probability level.

No. CCDC	2548115	Space Group	$P2_1/c$ (#14)
Empirical Formula	$C_{22}H_{38}GeO_2$	Z value	4
Formula Weight	407.11	D_{calc}	1.183 g/cm^3
Crystal Color	colorless	F_{000}	872.0
Crystal Dimensions	0.18 X 0.15 X 0.12 mm	μ(CuK$_\alpha$)	1.890 mm^{-1}
Crystal System	monoclinic	Temperature	123 K
Lattice Type	Primitive	Data/restraints/parameters	4549/0/236
Lattice Parameters	a = 11.2287(2) Å	Residuals: R_1 ($I > 2.00\sigma(I)$)	0.0339
	b = 10.8583(2) Å	Residuals: wR_2 (all data)	0.0954
	c = 19.2210(3) Å	Goodness of Fit Indicator	1.062
	α = 90 °		
	β = 102.670(2) °		
	γ = 90 °		
	V = 2286.45(7) Å^3		

5. Theoretical calculations

5-1. General

All calculations were conducted under vacuum at 298.15 K and 1 bar using the Gaussian 16 Rev. C. 01 program.[9] The geometry optimizations were carried out at ωB97XD density functional and 6-31G* basis set for all elements. All molecular geometries were fully optimized at the singlet states and were confirmed to have all positive vibrational frequencies for local minimum states or an imaginary vibrational frequency for transition states at the same theoretical level. By using the obtained optimized structures, the single points calculation to estimate the Gibbs free energies including contribution of vibrational entropy were performed by the ωB97XD/6-311G** level. Solvation effect was introduced using the SMD model and acetonitrile, hexane, or THF was used as a solvent. Natural bond orbital analyses[10,11] were performed under the optimized geometries using the NBO version 3.1 program as including in Gaussian.

5-2. Summary for molecular geometries included in the estimated reaction pathway

5-2-1. Formation of the dienolate **2b** or **IIa**

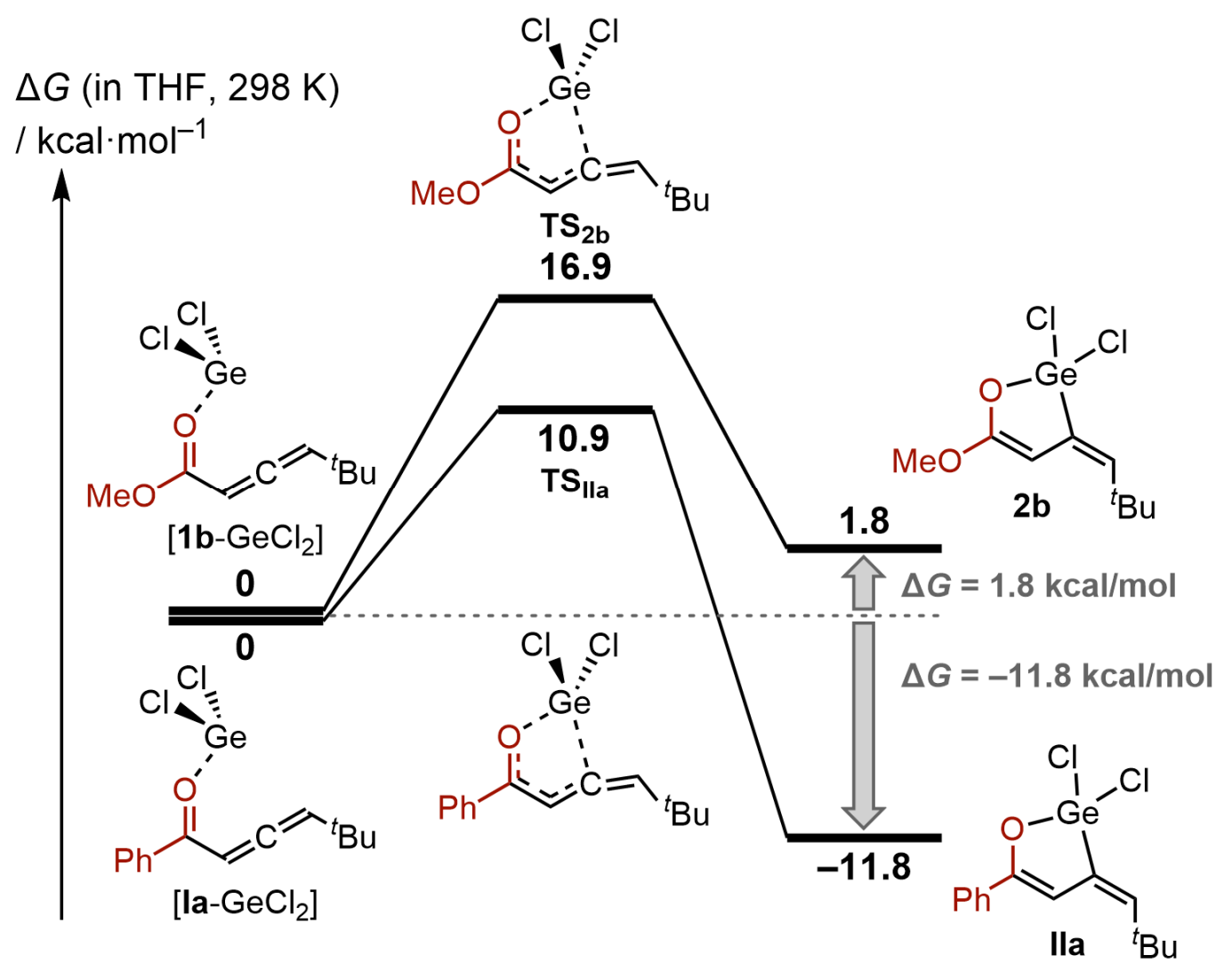

Figure S10. Energy profiles of the formation of the dienolate **2b** or **IIa** in THF. DFT calculations were performed at the ωB97XD/6-311G**/SMD(THF)//ωB97XD/6-31G* level.

Table S1. Summary for the energy of each reaction step in **Figure S10**

state	total energy / hartree [a]	ZPE / hartree [b]	Thermal correlation of Gibbs free energy / hartree [b]	total energy + ZPE / hartree	*G*(298 K) / hartree	imaginary frequency / cm^{-1} [b]
**1b**-$GeCl_2$	–3499.339917	0.219744	0.171304	–3499.120173	–3499.168613	–
$\mathbf{TS_{2b}}$	–3499.313807	0.219249	0.172091	–3499.094558	–3499.141716	227.8664*i*
**2b**	–3499.340910	0.221708	0.175129	–3499.119202	–3499.165781	–
**Ia**-$GeCl_2$	–3615.829762	0.268457	0.215872	–3615.561305	–3615.613890	–
$\mathbf{TS_{IIa}}$	–3615.813756	0.267808	0.217213	–3615.545948	–3615.596543	211.7585*i*
**IIa**	–3615.852788	0.270487	0.220166	–3615.582301	–3615.632622	–

a) ωB97XD/6-311G**/SMD(THF)//ωB97XD/6-31G*

b) ωB97XD/6-31G*

5-2-2. Aldol reaction of **2b** with **3a**·BF_3 in acetonitrile

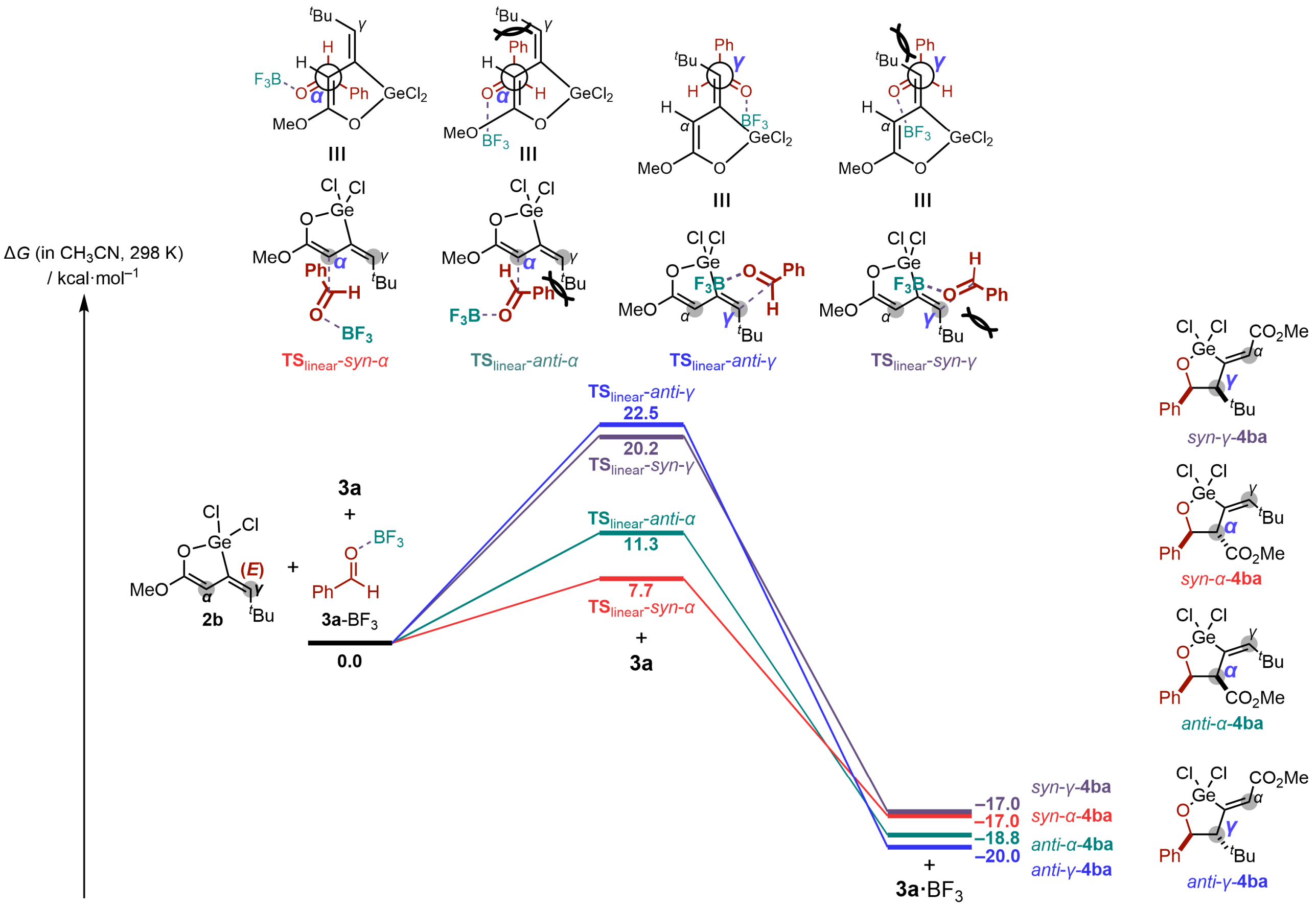

Figure S11. Energy profiles of the aldol reaction of **2b** with **3a**·BF_3 in acetonitrile. DFT calculations were performed at the ωB97XD/6-311G**/SMD(acetonitrile)//ωB97XD/6-31G* level.

Table S2. Summary for the energy of each reaction step in **Figure S11**

state	total energy / hartree [a)]	ZPE / hartree [b)]	Thermal correlation of Gibbs free energy / hartree [b)]	total energy + ZPE / hartree	*G*(298 K) / hartree	imaginary frequency / cm^{-1} [b)]
**2b**	–3499.341550	0.221708	0.175129	–3499.119842	–3499.166421	–
**3a**	–345.547917	0.111438	0.080935	–345.436479	–345.466982	–
**3a**·BF_3	–670.137877	0.126551	0.088561	–670.011326	–670.049316	–
$\mathbf{TS}_{\text{linear}}$-*syn*-*α*	–4169.495821	0.350996	0.292425	–4169.144825	–4169.203396	263.8786*i*
$\mathbf{TS}_{\text{linear}}$-*anti*-*α*	–4169.491713	0.351525	0.293980	–4169.140188	–4169.197733	273.6925*i*
$\mathbf{TS}_{\text{linear}}$-*syn*-*γ*	–4169.475863	0.350559	0.292363	–4169.125304	–4169.183500	292.7097*i*
$\mathbf{TS}_{\text{linear}}$-*anti*-*γ*	–4169.471426	0.350743	0.291467	–4169.120683	–4169.179959	334.2593*i*
syn-*α*-**4ba**	–3844.942397	0.338553	0.281968	–3844.603844	–3844.660429	–
anti-*α*-**4ba**	–3844.945938	0.338368	0.282535	–3844.607570	–3844.663403	–
syn-*γ*-**4ba**	–3844.946875	0.339273	0.286333	–3844.607602	–3844.660542	–
anti-*γ*-**4ba**	–3844.951143	0.339456	0.285936	–3844.611687	–3844.665207	–

a) ωB97XD/6-311G**/SMD(acetonitrile)//ωB97XD/6-31G*

b) ωB97XD/6-31G*

5-2-3. Aldol reaction of **2b** with **3a** in hexane

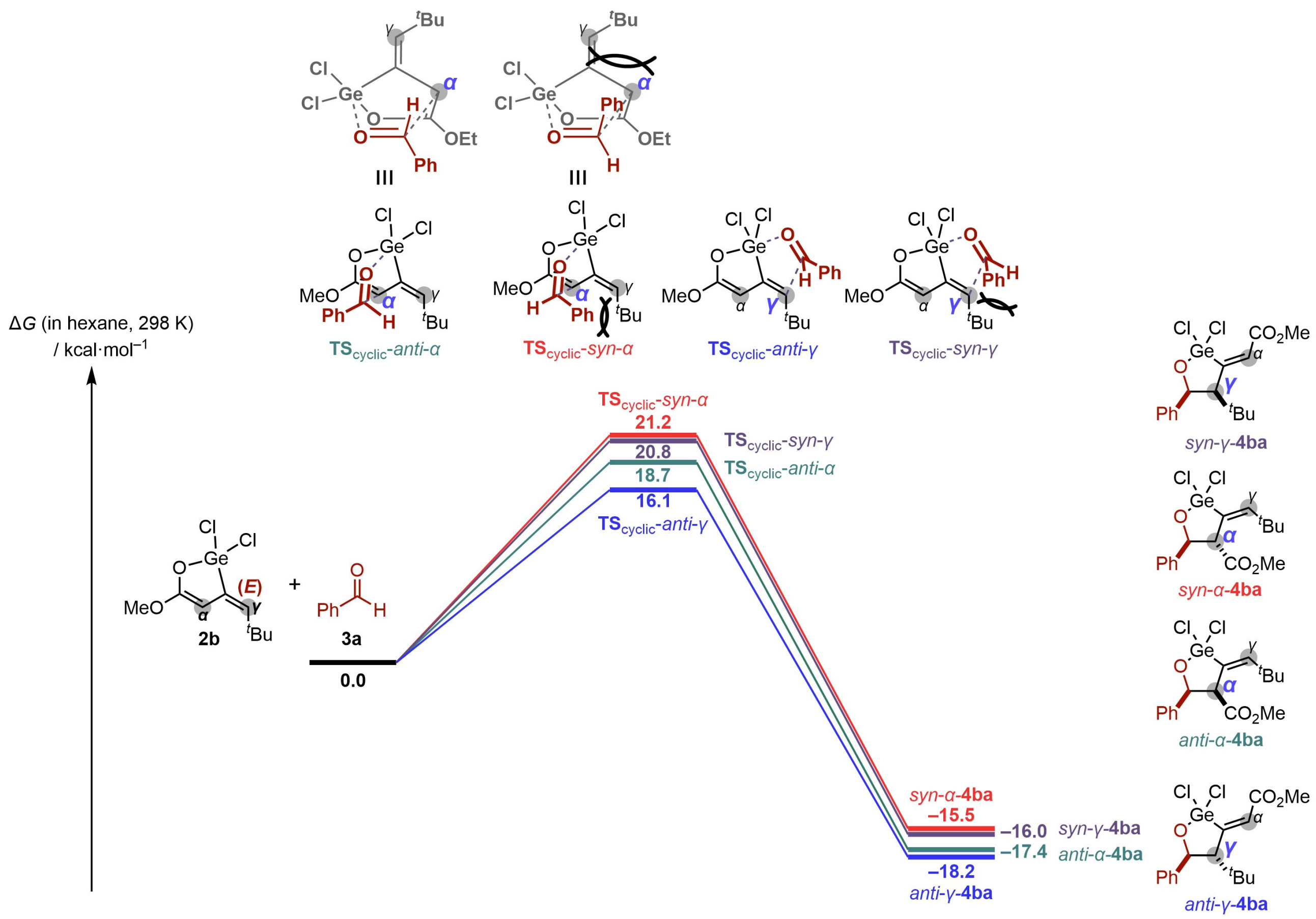

Figure S12. Energy profiles of the aldol reaction of **2b** with **3a** in hexane. DFT calculations were performed at the ωB97XD/6-311G**/SMD(*n*-hexane)//ωB97XD/6-31G* level.

Table S3. Summary for the energy of each reaction step in **Figure S12**

state	total energy / hartree [a)]	ZPE / hartree [b)]	Thermal correlation of Gibbs free energy / hartree [b)]	total energy + ZPE / hartree	G(298 K) / hartree	imaginary frequency / cm^{-1} [b)]
**2b**	–3499.339259	0.221708	0.175129	–3499.117551	–3499.164130	–
**3a**	–345.544852	0.111438	0.080935	–345.433414	–345.463917	–
$\mathbf{TS}_{cyclic}$-*syn*-*α*	–3844.875546	0.334645	0.281238	–3844.540901	–3844.594308	81.0638*i*
$\mathbf{TS}_{cyclic}$-*anti*-*α*	–3844.879554	0.335342	0.281359	–3844.544212	–3844.598195	54.2252*i*
$\mathbf{TS}_{cyclic}$-*syn*-*γ*	–3844.875871	0.334444	0.280949	–3844.541427	–3844.594922	309.1206*i*
$\mathbf{TS}_{cyclic}$-*anti*-*γ*	–3844.884334	0.335173	0.281928	–3844.549161	–3844.602406	290.6474*i*
syn-*α*-**4ba**	–3844.934699	0.338553	0.281968	–3844.596146	–3844.652731	–
anti-*α*-**4ba**	–3844.938352	0.338368	0.282535	–3844.599984	–3844.655817	–
syn-*γ*-**4ba**	–3844.939835	0.339273	0.286333	–3844.600562	–3844.653502	–
anti-*γ*-**4ba**	–3844.942915	0.339456	0.285936	–3844.603459	–3844.656979	–

a) ωB97XD/6-311G**/SMD(*n*-hexane)//ωB97XD/6-31G*

b) ωB97XD/6-31G*

5-2-4. Aldol reaction of [**2a**-Cl]⁻ with **3a** in THF

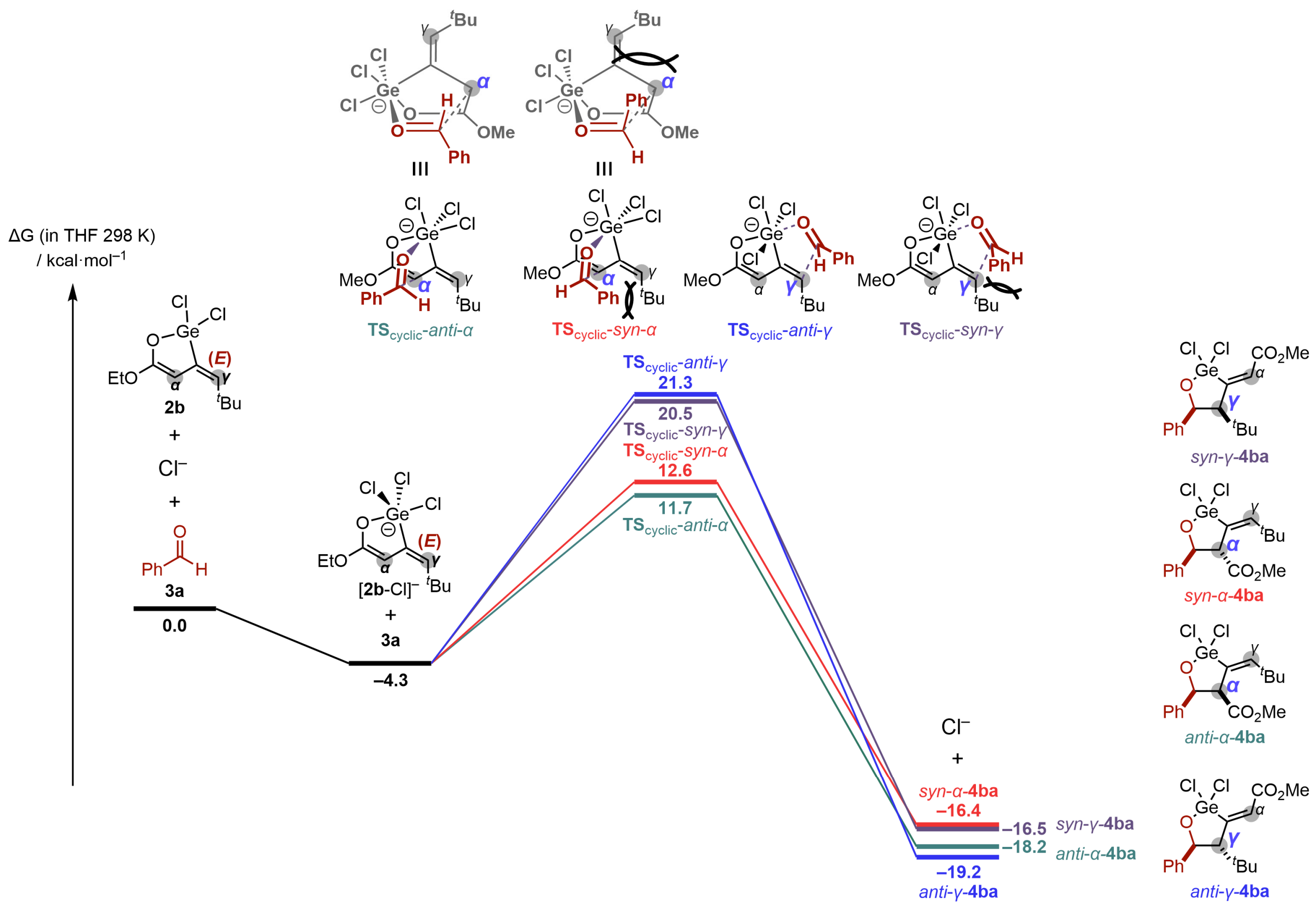

Figure S13. Energy profiles of the aldol reaction of [**2b**-Cl]⁻ with **3a** in THF. DFT calculations were performed at the ωB97XD/6-311G**/SMD(THF)//ωB97XD/6-31G* level.

Table S4. Summary for the energy of each reaction step in **Figure S13**

state	total energy / hartree [a)]	ZPE / hartree [b)]	Thermal correlation of Gibbs free energy / hartree [b)]	total energy + ZPE / hartree	G(298 K) / hartree	imaginary frequency / cm^{-1} [b)]
**2b**	–3499.340910	0.221708	0.175129	–3499.119202	–3499.165781	–
Cl^-	–460.375112	0	–0.015023	–460.375112	–460.390135	–
[**2b**-Cl]$^-$	–3959.735735	0.221631	0.173040	–3959.514104	–3959.562695	–
**3a**	–345.547118	0.111438	0.080935	–345.435680	–345.466183	–
$\mathbf{TS}_{cyclic}$-*syn*-*α*	–4305.281403	0.334638	0.279357	–4304.946765	–4305.002046	199.5634*i*
$\mathbf{TS}_{cyclic}$-*anti*-*α*	–4305.281026	0.334615	0.277635	–4304.946411	–4305.003391	210.4629*i*
$\mathbf{TS}_{cyclic}$-*syn*-*γ*	–4305.267767	0.334525	0.278412	–4304.933242	–4304.989355	245.1855*i*
$\mathbf{TS}_{cyclic}$-*anti*-*γ*	–4305.267299	0.334988	0.279147	–4304.932311	–4304.988152	239.5148*i*
syn-*α*-**4ba**	–3844.940042	0.338553	0.281968	–3844.601489	–3844.658074	–
anti-*α*-**4ba**	–3844.943469	0.338368	0.282535	–3844.605101	–3844.660934	–
syn-*γ*-**4ba**	–3844.944589	0.339273	0.286333	–3844.605316	–3844.658256	–
anti-*γ*-**4ba**	–3844.948557	0.339456	0.285936	–3844.609101	–3844.662621	–

a) ωB97XD/6-311G**/SMD(THF)//ωB97XD/6-31G*

b) ωB97XD/6-31G*

5-3. Changes in electronic and structural properties of 2b and 3c upon additive activation

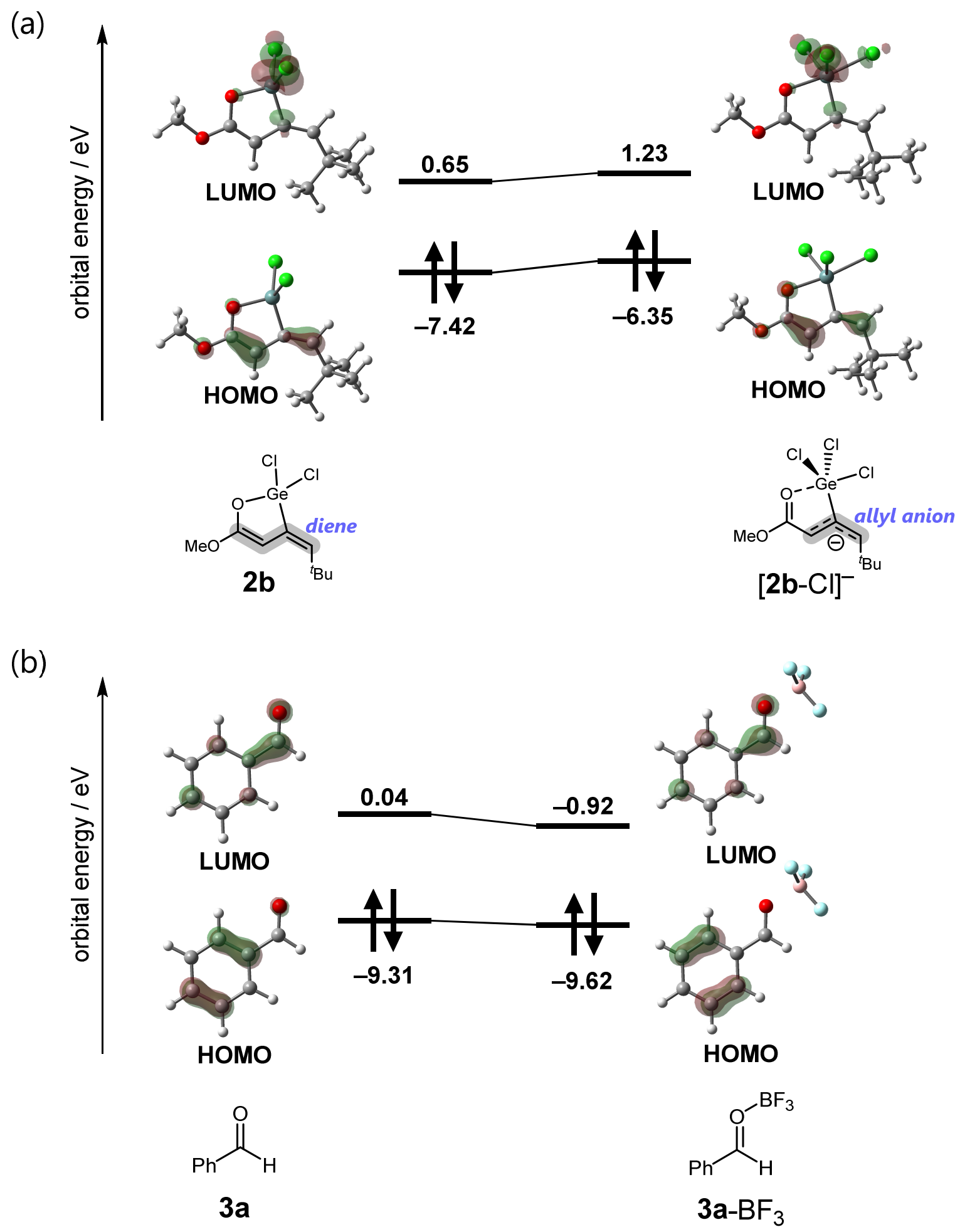

Figure S14. Calculated orbital energy diagram of HOMO and LUMO for (a) **2b** and [**2b**-Cl]$^{-}$, and (b) **3a** and **3a**-BF_3 at the ωB97XD/6-311G**(SMD)//ωB97XD/6-31G* level.

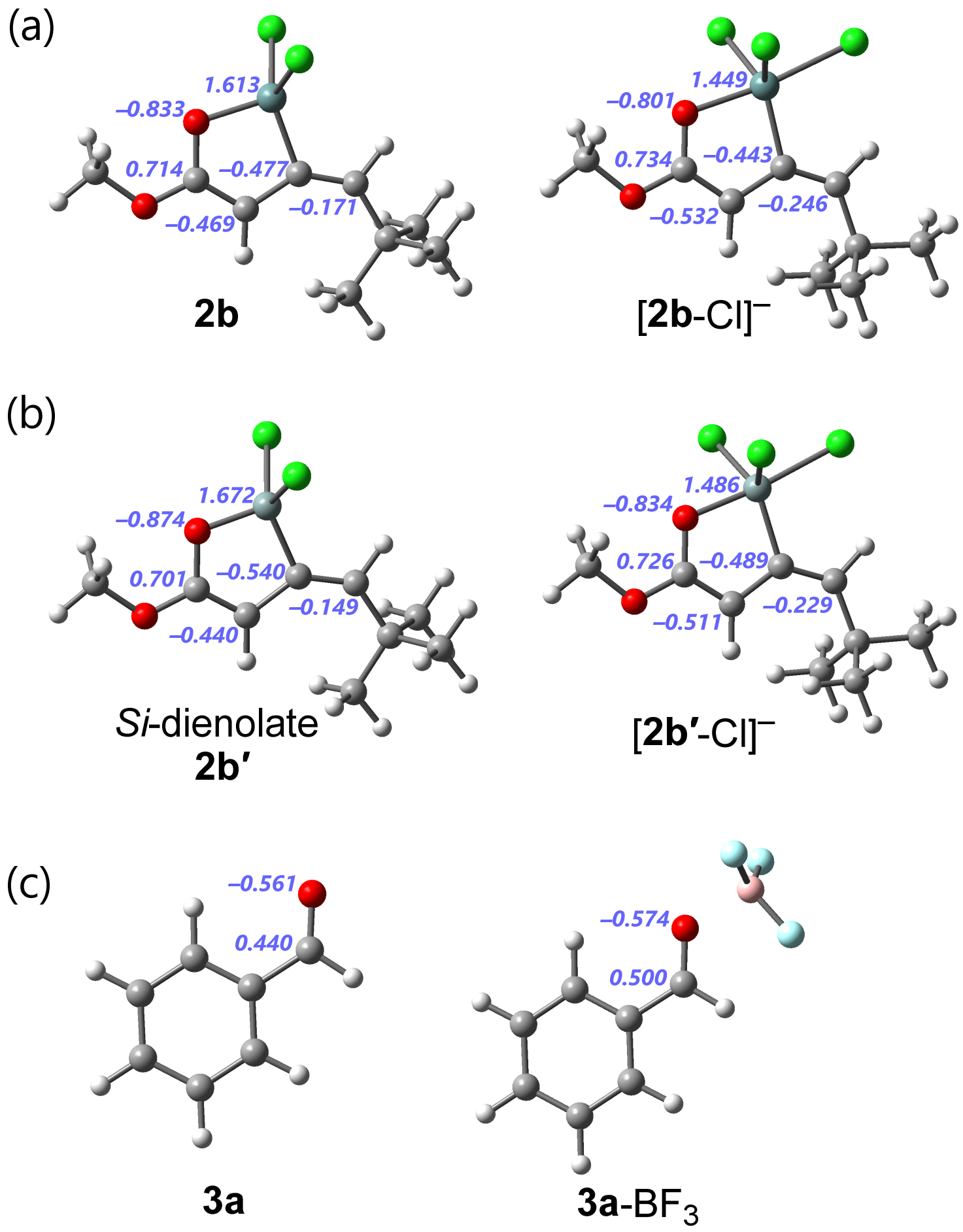

Figure S15. Summary of NBO charge values for (a) **2b** and [**2b**-Cl]$^{–}$, (b) **2b′** and [**2b′**-Cl]$^{–}$, and (c) **3a** and **3a**-BF_3 at the ωB97XD/6-311G**(SMD)//ωB97XD/6-31G* level.

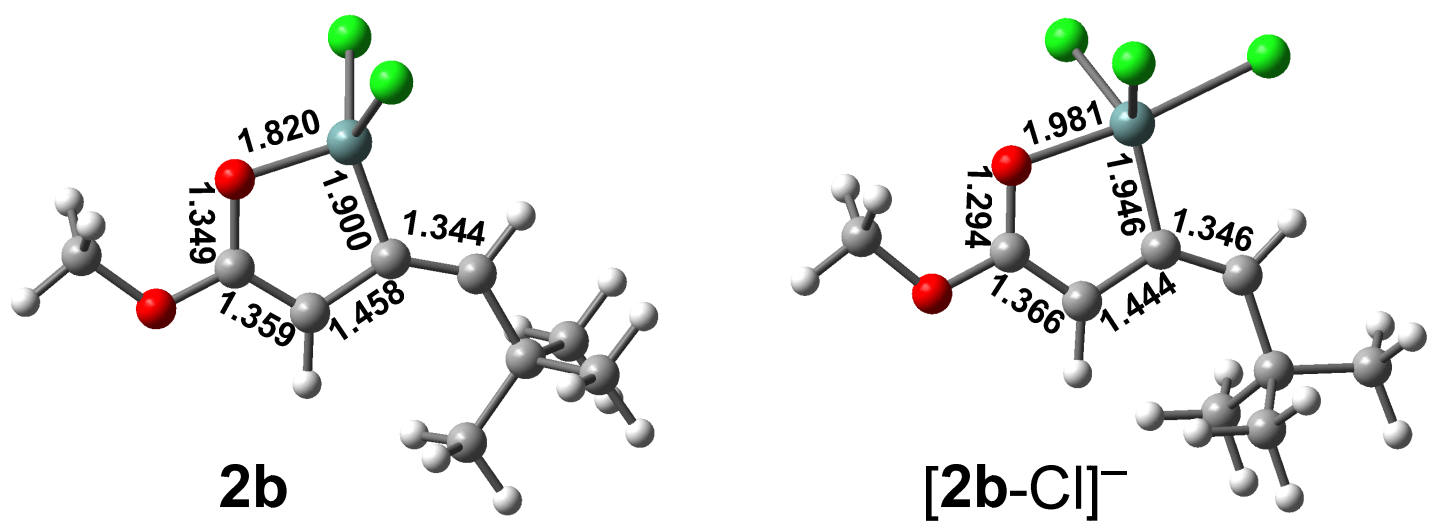

Figure S16. Summary of bond lengths (/Å) for **2b** and [**2b**-Cl]$^{–}$ at the ωB97XD/6-311G**(SMD)//ωB97XD/6-31G* level.

5-4. Cartesian coordinates for optimized geometries

5-4-1. Formation of the dienolate **2b** or **IIa** in THF

Table S5. Optimized Cartesian coordinates (Å) for **1b**-$GeCl_2$ in the singlet state at the ωB97XD/6-31G* level.

C	0.70242500	2.30344000	-0.16151500	H	3.38602200	-1.05788200	1.96607200
C	-0.66687000	1.83762500	-0.35963700	C	4.88941100	0.10527100	-0.01293000
H	0.84743400	3.27122800	0.30793300	H	5.31597700	0.14855800	-1.02166000
C	1.70841300	1.53588900	-0.52964200	H	5.03592900	1.08041900	0.46334700
C	2.68619500	0.76668600	-0.90320000	H	5.45068700	-0.64223300	0.55843400
H	3.04619700	0.88097400	-1.92720300	Ge	-0.93088200	-1.17488900	-0.22335700
C	3.39864400	-0.27392800	-0.04972900	Cl	-3.10936100	-1.48775400	-0.70384100
C	3.22911200	-1.64197000	-0.73101900	Cl	-1.15135200	-0.45555700	1.93592600
H	3.63035200	-1.63145600	-1.75097800	O	-0.96274000	0.78453000	-0.95481200
H	3.76685700	-2.41008200	-0.16426800	O	-1.57837400	2.64285000	0.11803000
H	2.17390400	-1.93001500	-0.78141500	C	-2.95095000	2.20374500	0.02936400
C	2.83784500	-0.31737900	1.37437000	H	-3.18597900	1.90892900	-0.99402500
H	2.93839500	0.65390500	1.87086900	H	-3.10057900	1.35721400	0.70090100
H	1.77930300	-0.59501700	1.39001100	H	-3.54058500	3.06289500	0.34186700

Table S6. Optimized Cartesian coordinates (Å) for $\mathbf{TS_{2b}}$ in the singlet state at the ωB97XD/6-31G* level.

C	0.37447100	1.74779500	-0.66828700	H	4.25671700	1.51728200	1.18498900
C	-0.86151800	1.86621600	0.00841300	C	4.43143700	0.00285000	-1.11343500
H	0.74193100	2.58880900	-1.24549800	H	4.45604800	-0.95392400	-1.64710800
C	0.93151700	0.50640500	-0.76821500	H	4.29692100	0.80194400	-1.85016200
C	2.00141600	-0.26638200	-0.77401400	H	5.40433000	0.14331600	-0.62882900
H	1.94838100	-1.23960500	-1.25704300	Ge	-1.00497300	-0.85218300	-0.42341000
C	3.31115800	0.02588200	-0.05836300	Cl	-3.09743600	-1.59366600	-0.64982300
C	3.52864600	-1.10812900	0.95947500	Cl	-0.38028800	-1.91831900	1.41647600
H	3.55570200	-2.08560800	0.46506700	O	-1.51497000	0.85464300	0.41617500
H	4.48106300	-0.96658200	1.48293500	O	-1.38513800	3.06787500	0.13952700
H	2.72158900	-1.12769300	1.69818000	C	-2.69164100	3.14476200	0.72842500
C	3.30300900	1.37341300	0.66622700	H	-3.40389100	2.55216700	0.15124700
H	3.16894600	2.20670500	-0.03127000	H	-2.66451600	2.77998200	1.75719400
H	2.49973000	1.41867200	1.40831500	H	-2.95259700	4.20110000	0.70105600

Table S7. Optimized Cartesian coordinates (Å) for dienolate **2b** in the singlet state at the ωB97XD/6-31G* level.

C	0.19674200	1.63209600	0.00005700	H	4.40003900	2.11797200	0.00032100
C	-1.15559200	1.76919200	-0.00013200	C	4.02559800	-0.27368100	-1.25516900
H	0.82165300	2.51173800	0.00022100	H	4.02472000	-1.36943100	-1.28170900
C	0.69680600	0.26289400	0.00001400	H	3.53804900	0.08776100	-2.16678800
C	1.90625800	-0.32312700	0.00003100	H	5.06903700	0.06275000	-1.26597000
H	1.91819200	-1.41494500	-0.00001700	Ge	-0.90204300	-0.76297900	-0.00005900
C	3.30474100	0.25502000	0.00008400	Cl	-1.40792200	-1.94180100	-1.70535800
C	4.02562100	-0.27395300	1.25520800	Cl	-1.40827300	-1.94134900	1.70546500
H	4.02474300	-1.36970900	1.28151000	O	-1.98129300	0.70270100	-0.00030200
H	5.06906000	0.06247500	1.26606300	O	-1.75311400	2.95730100	-0.00020000
H	3.53809100	0.08729100	2.16691600	C	-3.17829000	2.98652300	0.00007700
C	3.35591400	1.78550300	0.00025200	H	-3.58157900	2.49984300	-0.89153300
H	2.87420300	2.20318400	-0.88967200	H	-3.58122700	2.49992900	0.89189600
H	2.87414500	2.20298600	0.89023900	H	-3.43997000	4.04421900	0.00008000

Table S8. Optimized Cartesian coordinates (Å) for **Ia**-$GeCl_2$ in the singlet state at the ωB97XD/6-31G* level.

C	0.28008700	-1.82686600	-0.79327200	H	-3.60204400	-3.80513300	-0.46806300
C	1.16408200	-0.74231700	-0.36194900	H	-4.98830700	-3.07407600	0.36569600
H	0.70975600	-2.79596600	-1.02267900	Ge	-0.84089500	1.70745800	-0.49471500
C	-1.02130100	-1.64781200	-0.92241400	Cl	0.61945900	3.43113200	-0.53999300
C	-2.30243400	-1.46570700	-1.03689800	Cl	-1.21771000	1.62704900	1.74038400
H	-2.67241700	-1.11197100	-2.00038100	O	0.76221100	0.42300900	-0.20710100
C	-3.33807000	-1.69896900	0.05544700	C	2.59800400	-0.99154000	-0.10140700
C	-4.21265300	-0.43850200	0.16290600	C	3.15698000	-2.27517400	-0.06371500
H	-4.68010400	-0.19654400	-0.79866100	C	3.41336300	0.12595300	0.12999100
H	-5.01148200	-0.60661600	0.89357000	C	4.51191900	-2.43670600	0.19460500
H	-3.63095500	0.42666200	0.49477700	H	2.54467400	-3.15745900	-0.21440400
C	-2.66074100	-1.98834300	1.39798400	C	4.76702600	-0.04100700	0.38020300
H	-2.05293600	-2.89914300	1.34839400	H	2.96421500	1.11357700	0.10798100
H	-2.01805700	-1.15358300	1.69519000	C	5.31717200	-1.32116700	0.41201200
H	-3.41835100	-2.13039000	2.17543300	H	4.93946000	-3.43351700	0.22888600
C	-4.20392800	-2.89462900	-0.37795100	H	5.39390900	0.82747000	0.55367300
H	-4.68996900	-2.70758400	-1.34231400	H	6.37679500	-1.45081300	0.61067300

Table S9. Optimized Cartesian coordinates (Å) for **TS_{IIa}** in the singlet state at the ωB97XD/6-31G* level.

C	0.20360800	-1.19131200	-0.74262000	H	-3.37493800	-3.23348000	-1.86264800
C	1.18486400	-0.41974000	-0.08264200	H	-4.52815700	-3.61541700	-0.56774400
H	0.50132400	-2.01842600	-1.37912900	Ge	-0.65907100	1.59772700	-0.43598800
C	-1.08824200	-0.75278600	-0.76692500	Cl	0.31759200	3.65643200	-0.60996600
C	-2.38886500	-0.94889600	-0.72606100	Cl	-1.80915200	1.94982100	1.45963900
H	-3.05904700	-0.21911700	-1.17505300	O	0.87750000	0.74818600	0.36718100
C	-3.07194000	-2.10696200	-0.01213100	C	2.58869400	-0.83300300	0.03620600
C	-3.93710000	-1.50322300	1.10643500	C	3.56645800	0.13796400	0.29054900
H	-4.65122300	-0.77349000	0.70828800	C	2.95871300	-2.17876300	-0.08517400
H	-4.50395200	-2.29626400	1.60773800	C	4.89941900	-0.23314800	0.40374500
H	-3.31422700	-0.99923100	1.85277500	H	3.27143200	1.17777900	0.38344500
C	-2.07940200	-3.10801700	0.58044100	C	4.29288200	-2.54462500	0.03827800
H	-1.45637800	-3.56569100	-0.19584900	H	2.20569200	-2.94286200	-0.25196900
H	-1.42047600	-2.63138200	1.31417000	C	5.26365600	-1.57316100	0.27826000
H	-2.62759300	-3.90926400	1.08773600	H	5.65575800	0.52270900	0.59037200
C	-3.96958500	-2.80422400	-1.04868700	H	4.57604400	-3.58889100	-0.04857300
H	-4.69280900	-2.10468100	-1.48306500	H	6.30636600	-1.86196400	0.37119100

Table S10. Optimized Cartesian coordinates (Å) for dienolate **IIa** in the singlet state at the ωB97XD/6-31G* level.

C	0.25461300	1.02523300	-0.01126100	H	-3.17694400	2.40331000	2.11181900
C	1.31131500	0.17442800	0.01969000	H	-4.25016000	3.47310300	1.18505200
H	0.40813100	2.09236300	-0.05721200	Ge	-0.69089100	-1.43295200	0.02595100
C	-1.07765600	0.42723200	-0.00699300	Cl	-1.24253100	-2.57644100	1.74213300
C	-2.33150400	0.90902100	-0.02575200	Cl	-1.20515800	-2.62890700	-1.66745700
H	-3.13316400	0.16743100	-0.01925900	O	1.09238900	-1.17430800	0.04057300
C	-2.87872800	2.31870900	-0.05300600	C	2.73734600	0.54117800	0.01513300
C	-3.75161000	2.44761100	-1.31698700	C	3.69839300	-0.45157500	-0.20319200
H	-4.54083300	1.68753100	-1.33470800	C	3.15915400	1.86003700	0.22305700
H	-4.23162700	3.43260700	-1.34722400	C	5.05012700	-0.12841800	-0.22856200
H	-3.14786900	2.33117100	-2.22318800	H	3.37204900	-1.47399800	-0.35359300
C	-1.81563000	3.42070400	-0.06348300	C	4.50993600	2.17887900	0.19704500
H	-1.18479000	3.37923200	0.83044400	H	2.43327000	2.64211000	0.42231100
H	-1.17819100	3.35611300	-0.95128900	C	5.46087100	1.18619400	-0.03117100
H	-2.30508400	4.40079800	-0.07830500	H	5.78483200	-0.90873900	-0.40251500
C	-3.76865300	2.48841500	1.19426100	H	4.82273200	3.20539900	0.36263400
H	-4.55717200	1.72810700	1.22698100	H	6.51709100	1.43743300	-0.04911100

5-4-2. Aldol reaction of **2b** with **3a**·BF_3 in acetonitrile

Table S11. Optimized Cartesian coordinates (Å) for benzaldehyde **3a** in the singlet state at the ωB97XD/6-31G* level.

O	2.83751200	-0.39600700	-0.00033900	C	-2.21022200	-0.25049100	-0.00008700
C	1.98992000	0.46830000	0.00021800	H	2.27509400	1.54302500	0.00068800
C	0.53042800	0.21418200	0.00013000	H	0.02396400	2.30639500	0.00002800
C	-0.36022600	1.28853800	0.00001400	H	0.75893600	-1.91586800	0.00020800
C	0.04599600	-1.09716600	0.00014100	H	-2.42557100	1.89218800	-0.00024900
C	-1.73163900	1.05741000	-0.00012300	H	-1.70286700	-2.34425800	0.00010500
C	-1.32240700	-1.32742500	0.00005800	H	-3.28076000	-0.43350900	-0.00016800

Table S12. Optimized Cartesian coordinates (Å) for **3a**·BF_3 in the singlet state at the ωB97XD/6-31G* level.

O	1.09679600	0.41226500	0.00077900	H	-1.69705000	-2.29511200	0.00007900
C	0.24476800	-0.48031400	0.00040900	H	-0.94182700	1.93740600	0.00039300
C	-1.17949000	-0.20066500	0.00026700	H	-4.14280900	-1.86483900	-0.00031100
C	-2.07561000	-1.27623300	0.00005800	H	-3.39698500	2.36991000	-0.00004000
C	-1.65411200	1.11890200	0.00024600	H	-4.97967600	0.46574600	-0.00039600
C	-3.44309500	-1.03582300	-0.00015600	B	2.76761400	0.03192700	-0.00014400
C	-3.01978100	1.35262700	-0.00000200	F	3.17950200	0.60732100	-1.15228800
C	-3.91040000	0.27683500	-0.00020300	F	3.18174900	0.61011800	1.14973700
H	0.57742900	-1.52522700	0.00016800	F	2.77150400	-1.33717500	0.00153600

Table S13. Optimized Cartesian coordinates (Å) for **TS**$_{linear}$-*syn*-*α* in the singlet state at the ωB97XD/6-31G* level.

O	-2.61583800	-0.83756300	0.30657400	Cl	3.20013600	0.10229200	-2.39691600
C	-1.62182600	-0.18590600	0.79713500	Cl	3.09067500	-1.47254000	0.58688900
C	-0.64238600	-0.97525500	1.58748500	O	0.57427200	-1.36017700	-1.47171700
C	0.13464100	-0.35741400	2.57170200	H	-1.38549000	1.26062400	-1.06901600
C	-0.55176800	-2.35910300	1.40968900	H	2.13912800	2.27966800	0.61548100
C	0.97413000	-1.11513400	3.38044900	H	0.77095000	5.62432100	0.45864200
C	0.29273900	-3.11332700	2.21363700	H	2.20161500	4.62743400	0.77251700
C	1.05080600	-2.49376300	3.20513200	H	1.48485300	4.66421900	-0.85045600
H	-1.79553300	0.83566600	1.14767300	H	-1.40818300	4.64192400	0.03021000
H	0.05812900	0.71421600	2.72332100	H	-1.75183600	2.92183200	0.12925300
H	-1.18532600	-2.83599900	0.66931800	H	-0.83773100	3.56476400	-1.24960800
H	1.56148200	-0.62972800	4.15357600	H	0.96942400	3.38341200	2.69358400
H	0.34977500	-4.18872500	2.07740300	H	-0.35834300	4.51760000	2.38761800
H	1.70214800	-3.08636500	3.84024900	H	-0.67699800	2.77892100	2.39988800
C	-0.57547500	0.59927200	-0.79291600	C	-1.68803100	-2.39529000	-2.52930000
C	-0.49755000	-0.60140400	-1.52312600	H	-2.76196800	-2.56459300	-2.50311200
C	0.72038000	1.08570000	-0.30328600	H	-1.28456600	-2.52778900	-3.53460900
C	1.09938600	2.22747500	0.28711300	H	-1.16921700	-3.04563400	-1.82484400
C	0.32979300	3.49670100	0.59515200	O	-1.54658800	-1.01958900	-2.14391700
C	1.25549300	4.67147800	0.22053800	B	-3.88117600	-0.09424400	-0.21521500
C	-0.99125300	3.64886800	-0.16849400	F	-4.29844600	-0.82641300	-1.30349700
C	0.05442200	3.54111400	2.11078200	F	-4.78201300	-0.07295300	0.81764600
Ge	1.92028800	-0.31883100	-0.73916300	F	-3.47123000	1.20234700	-0.56581700

Table S14. Optimized Cartesian coordinates (Å) for **TS**$_{linear}$-*anti*-*α* in the singlet state at the ωB97XD/6-31G* level.

O	1.19416200	2.46112800	0.27306800	H	-5.36056100	-0.72984600	0.57859700
C	0.38106300	1.67113100	-0.32289200	H	-4.27567800	-0.04981100	-0.64733700
C	-0.01836700	0.06163400	1.03847700	C	-1.00519300	2.12888000	-0.54093500
C	1.31638800	-0.31240900	1.32512000	C	-1.79034600	1.50338600	-1.51149400
C	-0.73873100	-0.99504700	0.30930000	C	-1.50607600	3.22857100	0.16128100
C	-2.01335600	-1.40981800	0.24356700	C	-3.06987100	1.97863400	-1.78139300
C	-3.25254400	-1.01890600	1.01894900	H	-1.39496700	0.65019300	-2.05525600
C	-3.53211100	-2.19557200	1.97948900	C	-2.78971600	3.68899100	-0.09957800
C	-3.12919700	0.27299400	1.83043300	H	-0.87159000	3.71933900	0.89183500
C	-4.42562200	-0.89371900	0.03138800	C	-3.57269700	3.06525400	-1.07119700
Ge	0.66378100	-1.95186500	-0.53161800	H	-3.67168000	1.50370800	-2.55038200
Cl	0.68570000	-4.06417100	-0.36690200	H	-3.17833000	4.54416800	0.44453000
Cl	1.33431500	-1.47018600	-2.48861700	H	-4.57156500	3.43483600	-1.28239000
O	1.89586400	-1.29336200	0.67337600	H	0.78972800	1.01736000	-1.09604400
H	-0.50389000	0.68322400	1.77801900	C	3.40755100	0.03894800	2.35615400
H	-2.20607300	-2.25446100	-0.42146600	H	3.88470100	0.07979400	1.38008700
H	-4.45065300	-2.00584200	2.54652200	H	3.78372500	0.83010800	2.99775100
H	-3.66088700	-3.13697300	1.43353800	H	3.51851000	-0.94328500	2.81942900
H	-2.70846500	-2.32367800	2.68974000	B	2.71087400	2.37392600	-0.06834900
H	-4.09331700	0.50124000	2.29785400	F	2.93625400	3.25402600	-1.09122200
H	-2.85415200	1.12306900	1.20060100	F	2.94262300	1.04404700	-0.47026300
H	-2.39481700	0.17404400	2.63603000	F	3.38189900	2.67817900	1.09163500
H	-4.54236500	-1.80479800	-0.56718000	O	1.99906000	0.33195200	2.20999300

Table S15. Optimized Cartesian coordinates (Å) for **TS**$_{linear}$-*syn*-*γ* in the singlet state at the ωB97XD/6-31G* level.

O	0.97246300	1.75213300	0.20198000	Cl	-1.65635700	-0.56672900	-2.86597700
C	1.42736000	0.76815400	-0.49284300	Cl	-2.79862600	-2.70900400	-0.58555200
C	2.88414200	0.55638600	-0.54772300	O	-2.98362100	0.28615700	0.15308700
C	3.40841400	-0.30695900	-1.51393900	H	-0.68857000	0.26560300	2.55682200
C	3.74636800	1.25929700	0.29634000	H	1.00851800	-1.73160200	-0.44746300
C	4.77944900	-0.49946200	-1.60899400	H	3.44344400	-2.77862800	2.01349900
C	5.11846700	1.06040900	0.20377300	H	2.90122300	-2.83866200	0.33023900
C	5.63592600	0.17560500	-0.74065400	H	3.71376300	-1.39285300	0.95589500
H	0.89192200	0.50575300	-1.40581200	H	2.69521200	-0.87850900	3.39633100
H	2.73530200	-0.83440100	-2.18577300	H	0.99974900	-0.42066900	3.37735100
H	3.33141800	1.96651800	1.00635700	H	2.14209600	0.40484400	2.30880400
H	5.18223700	-1.17402400	-2.35789000	H	0.72882000	-3.62089800	1.54803800
H	5.78781100	1.60380800	0.86339100	H	1.37312400	-3.20619600	3.14856600
H	6.70864800	0.02310200	-0.81040800	H	-0.15208000	-2.49018300	2.58886100
C	-1.13106500	0.00290500	1.60867900	C	-4.15082200	2.02871000	1.69410700
C	-2.39630000	0.52677800	1.29886700	H	-4.92682800	1.37261200	1.29714200
C	-0.48068300	-0.68042500	0.58311700	H	-4.50870700	2.56613100	2.56973200
C	0.82213000	-1.14499600	0.45397400	H	-3.80371800	2.72456100	0.92867900
C	1.64242600	-1.63389100	1.65923300	O	-3.03110500	1.26019600	2.16450700
C	3.00491300	-2.18445400	1.20383700	B	-0.26086600	2.52972800	-0.28412200
C	1.87669800	-0.56250400	2.74031400	F	0.18429000	3.58528300	-1.02530700
C	0.84707000	-2.80734900	2.27296800	F	-1.01178100	1.63143700	-1.10117300
Ge	-1.81151400	-0.80594200	-0.77007400	F	-0.99748200	2.89722800	0.81911300

Table S16. Optimized Cartesian coordinates (Å) for **TS**$_{linear}$-*anti*-*γ* in the singlet state at the ωB97XD/6-31G* level.

O	0.40317700	0.34881900	-1.59695000	Cl	0.06534000	-2.86640200	-0.23499800
C	1.34613200	0.83674400	-0.85703900	Cl	-1.33022000	-2.12529900	2.75927700
C	2.52744300	-0.06137200	-0.70301800	O	-2.53326400	-1.09093300	0.00806900
C	3.60182700	0.22110500	0.14661100	H	-1.94933800	2.15654000	0.33301600
C	2.60483000	-1.18583000	-1.53250300	H	1.38364500	0.45571400	1.65614900
C	4.70884400	-0.61573300	0.19182400	H	2.38399300	3.94459300	2.28779500
C	3.71300700	-2.02385200	-1.48614900	H	2.87684200	2.25125900	2.35988800
C	4.76501000	-1.74696100	-0.61943500	H	2.72605300	3.08047000	0.78856500
H	1.52849500	1.91152100	-0.91638400	H	0.47060500	4.63182400	1.14224300
H	3.57722000	1.09863700	0.77813200	H	-1.00569500	3.68975500	1.19218600
H	1.79576300	-1.37788900	-2.22645500	H	0.10791300	3.48601600	-0.16315000
H	5.53133500	-0.38215600	0.86098600	H	0.82468100	1.85940800	3.79752500
H	3.75628200	-2.89104000	-2.13792900	H	0.32574200	3.55253900	3.61376800
H	5.63082100	-2.40128100	-0.58318900	H	-0.79217200	2.24317500	3.17994300
C	-1.76676800	1.10298300	0.46908600	C	-4.59320700	-0.26651200	-1.37145600
C	-2.71568100	0.20203900	-0.05206700	H	-4.93046300	-1.10891200	-0.76590700
C	-0.57067700	0.55854100	0.90875400	H	-5.43754300	0.32274600	-1.72202600
C	0.66875500	1.13364600	1.18986700	H	-3.98030400	-0.60496300	-2.20750000
C	0.80613400	2.57155400	1.72357000	O	-3.81386900	0.64472400	-0.57487000
C	2.28653700	2.97558200	1.78671900	B	-0.51583700	1.16865300	-2.51415000
C	0.05155800	3.64555600	0.91563500	F	-1.72430000	0.46812400	-2.51347200
C	0.25810700	2.55371300	3.16620100	F	0.06961900	1.19107800	-3.75308200
Ge	-0.89369800	-1.32636000	0.82987700	F	-0.65008000	2.44225500	-1.96848200

Table S17. Optimized Cartesian coordinates (Å) for *syn*-*α*-**4ba** in the singlet state at the ωB97XD/6-31G* level.

Ge	0.05889800	1.66298800	-0.42654900	H	2.94860300	-1.32199600	4.23697200
O	-1.03140500	0.76399300	-1.51308600	H	1.31007600	-0.66296800	4.06819600
O	2.16806500	-0.76668100	-1.84132400	Cl	1.32318600	2.93046100	-1.58096500
C	0.76755500	0.11963100	0.41348000	Cl	-0.99530800	2.98971500	0.90062300
C	-1.10813600	-0.60080000	-1.10489300	H	-1.21117400	-1.18712200	-2.02339800
C	1.24544400	-1.44392000	-1.45613200	C	-2.33551200	-0.85063300	-0.24720300
C	1.60773300	0.15868500	1.44845500	C	-3.45764500	-1.43610400	-0.83192800
H	1.84909900	1.15447900	1.82761900	C	-2.38772400	-0.49863700	1.10528900
C	0.22870900	-1.03686700	-0.39990900	C	-4.61356800	-1.66314600	-0.09049600
H	0.00123000	-1.91525600	0.20349500	H	-3.42857000	-1.71207700	-1.88310000
C	2.37296500	-0.92644600	2.17507600	C	-3.54232400	-0.72078000	1.84699600
C	1.84502300	-2.34911700	1.95881600	H	-1.52931100	-0.04335900	1.59138000
H	0.79074800	-2.43488700	2.24441700	C	-4.65798600	-1.30507900	1.25264500
H	2.41546400	-3.04511800	2.58354900	H	-5.47805400	-2.11923100	-0.56355700
H	1.95808000	-2.68234700	0.92263500	H	-3.56943100	-0.43787400	2.89499400
C	3.82630600	-0.83795700	1.66568800	H	-5.55798900	-1.47872800	1.83485700
H	3.86978900	-1.03320100	0.58943800	C	1.83537700	-3.13143100	-2.96755500
H	4.45281100	-1.57313800	2.18431100	H	1.47048600	-4.12303400	-3.23229200
H	4.25052600	0.15587800	1.84607300	H	1.78812500	-2.46025500	-3.82812800
C	2.33323000	-0.60691600	3.68004200	H	2.86708200	-3.18331000	-2.61192900
H	2.71875200	0.39846400	3.88357800	O	0.97187900	-2.66936500	-1.92608300

Table S18. Optimized Cartesian coordinates (Å) for *anti*-*α*-**4ba** in the singlet state at the ωB97XD/6-31G* level.

Ge	1.20904700	-1.53936400	0.03702900	H	1.08894000	3.02002900	-1.94682700
O	-0.50867800	-1.76425900	-0.37492200	H	1.62891200	4.42886400	-1.02693000
O	-1.70266000	2.11320000	0.69312300	H	0.46133600	3.32120600	-0.30837300
O	-0.61405200	0.55068400	1.89315600	C	-5.21459200	-0.13122900	-0.39611200
C	1.22048000	0.33214500	-0.28754000	H	-6.27830500	0.00651400	-0.22591400
C	-2.48272800	-0.49046900	-0.83574300	C	-4.61062700	0.42346400	-1.52177100
C	-0.98800500	-0.59973700	-1.02433200	H	-5.20116100	0.99419000	-2.23230300
H	-0.75048700	-0.66327400	-2.09632500	C	-2.46764600	2.43933800	1.85569900
C	-0.84819400	1.09585700	0.83942100	H	-1.81128700	2.69830900	2.68941000
C	2.31818000	1.08973000	-0.25417600	H	-3.09304000	1.58751300	2.13391300
H	3.25428400	0.56389800	-0.05160700	C	2.81821000	3.08136700	1.06907700
C	-0.23985700	0.68404500	-0.48861400	H	1.95494200	2.90996100	1.72072600
H	-0.39901000	1.47842600	-1.21686900	H	3.03921000	4.15498700	1.05822000
C	2.53034400	2.58507700	-0.36302500	H	3.67907000	2.56278400	1.50527800
C	-3.24922400	0.24177800	-1.74055300	C	3.76950700	2.82215400	-1.24503300
H	-2.77870800	0.67525700	-2.62045400	H	4.64623500	2.29606700	-0.85071800
C	-3.08963900	-1.05259900	0.28684400	H	4.00820900	3.89066900	-1.28439400
H	-2.48232900	-1.62887500	0.97620000	H	3.59676900	2.47307700	-2.26895900
C	-4.45256000	-0.87207300	0.50380900	H	-3.08545900	3.28951500	1.57001100
H	-4.92086800	-1.31683700	1.37701700	Cl	1.61355200	-2.31685900	1.97723500
C	1.35072200	3.37250200	-0.94307700	Cl	2.45528100	-2.67183000	-1.30112600

Table S19. Optimized Cartesian coordinates (Å) for *syn*-*γ*-**4ba** in the singlet state at the ωB97XD/6-31G* level.

Ge	1.00347700	-1.00645100	0.21795900	C	-2.58191000	2.38457300	0.70154300
Cl	1.66913100	-2.44487300	-1.23607300	H	-2.83916400	2.79102200	-0.28321400
Cl	1.66171600	-1.56154200	2.16541000	H	-2.78230800	3.16289100	1.44675300
O	-0.78029000	-1.22249300	0.25254900	H	-3.25303600	1.55055300	0.90698800
O	3.37866400	-0.13822700	-0.08069400	C	-2.83897000	-0.36790000	-0.62231500
C	0.80374200	0.79754600	-0.42581200	C	-3.49155200	-1.05684700	0.39683100
C	-1.33248600	-0.29972000	-0.67002500	C	-3.59054400	0.25629200	-1.61849000
C	-0.67088500	1.11749100	-0.46297100	C	-4.88306500	-1.07956600	0.44423900
H	-0.89184200	1.71874600	-1.35531000	H	-2.89651200	-1.57346500	1.14143900
C	1.84868000	1.52113100	-0.82154600	C	-4.97907300	0.23603700	-1.57027000
H	1.77521200	2.51028900	-1.26333300	H	-3.08519600	0.77244600	-2.43228200
C	3.19446200	0.94643300	-0.60937400	C	-5.63009600	-0.42608900	-0.53127600
C	-1.09664800	1.98816500	0.77940800	H	-5.38462900	-1.61410100	1.24572100
C	-0.83158300	1.27133000	2.11149300	H	-5.55482200	0.73292600	-2.34561100
H	-1.33559700	0.30332200	2.16111500	H	-6.71515300	-0.44329900	-0.49144400
H	-1.19089500	1.89291100	2.93948300	H	-1.03467500	-0.59333400	-1.69251700
H	0.23926300	1.10200600	2.27645700	O	4.17421100	1.72863000	-1.03976500
C	-0.28496200	3.29603200	0.76024000	C	5.50038200	1.21978800	-0.85241200
H	0.78338900	3.12510000	0.91786200	H	5.61998700	0.27369600	-1.38431900
H	-0.63116000	3.95332100	1.56487200	H	6.16335400	1.98034200	-1.26145300
H	-0.41388100	3.83494100	-0.18709100	H	5.69836200	1.06373300	0.21016400

Table S20. Optimized Cartesian coordinates (Å) for *anti*-*γ*-**4ba** in the singlet state at the ωB97XD/6-31G* level.

Ge	-0.65287300	-0.35616300	-1.04453500	H	-1.31452300	2.67871700	-0.83806300
Cl	-0.51501100	-2.49994800	-1.14175500	C	4.89479500	-1.16022600	0.31577800
Cl	-1.99869700	0.30549500	-2.56604600	H	5.94824800	-1.07967200	0.06447600
O	0.95834800	0.26398200	-1.52832000	C	4.44316200	-2.20757300	1.11133800
O	-2.81197300	-0.99120500	0.22930700	H	5.14101000	-2.95110800	1.48468800
O	-3.37835300	-0.72752100	2.38693800	C	2.19441300	-1.34711300	0.95044400
C	-0.52213900	0.50424600	0.66778400	H	1.14272300	-1.44409300	1.20656100
C	1.68934500	0.75807000	-0.41094600	C	-0.46029400	3.36782300	1.64814000
H	2.30340700	1.56999000	-0.80619200	H	-1.44931000	2.90099800	1.64527900
C	2.63610700	-0.29384700	0.14382200	H	-0.61123700	4.44932200	1.56267700
C	0.72615100	1.34233700	0.68166000	H	0.01272300	3.17219000	2.61860200
H	1.21780700	1.24870400	1.65760300	C	-4.54773400	-1.51764500	2.14281700
C	-1.40023100	0.28700000	1.64583500	H	-4.26481600	-2.51638100	1.80361700
H	-1.30543900	0.69617700	2.64721800	H	-5.17289300	-1.04325700	1.38325300
C	-2.58614600	-0.54010600	1.33846200	C	1.72998300	3.66752800	0.52419800
C	0.41397200	2.87148700	0.48702000	H	2.32738800	3.41009700	1.40690300
C	3.99394700	-0.21230700	-0.16163200	H	1.51114600	4.73981500	0.56888000
H	4.35043100	0.60100500	-0.78921100	H	2.34703100	3.50594800	-0.36421200
C	-0.31786600	3.13505600	-0.83606200	C	3.08919400	-2.29921000	1.42450600
H	0.23003900	2.73783400	-1.69616100	H	2.72753200	-3.11477000	2.04354500
H	-0.45082700	4.21297100	-0.98120200	H	-5.06961800	-1.56673900	3.09711700

5-4-3. Aldol reaction of **2b** with **3a** in hexane

Table S21. Optimized Cartesian coordinates (Å) for **TS**$_{cyclic}$-*syn-α* in the singlet state at the ωB97XD/6-31G* level.

Ge	-1.54850200	-0.36656000	-0.61811200	H	3.71520100	-1.33906800	2.25249800
O	-0.74870200	1.36234600	-1.37606000	H	2.59454900	-0.04349100	1.80096200
O	-2.28073600	0.94299500	0.60562700	Cl	-3.41322000	-1.46842900	-0.37044000
C	-0.11878700	-0.63364500	0.61576500	Cl	-1.15444700	-1.24130000	-2.62179500
C	0.15200200	1.94969000	-0.71486500	H	-0.10662100	2.87500800	-0.18211600
C	-1.33783200	1.24669600	1.44103200	C	1.56721000	1.63788800	-0.85816100
C	0.70658200	-1.69122700	0.55857800	C	2.51690500	2.41372600	-0.17941900
H	0.55173000	-2.38552300	-0.26742000	C	1.98261300	0.60212100	-1.70441300
C	-0.14760700	0.53650300	1.46494500	C	3.86879200	2.16079800	-0.34827600
H	0.64030900	0.82297000	2.14782400	H	2.18513300	3.21134600	0.48103700
C	1.79460800	-2.07568500	1.54885500	C	3.34053700	0.36190100	-1.87892100
C	1.20179300	-2.18321400	2.96543100	H	1.23920000	0.01809000	-2.23588200
H	0.79808100	-1.22630400	3.30893800	C	4.28025600	1.13718800	-1.20471900
H	1.97462200	-2.50158800	3.67507400	H	4.60510200	2.76054900	0.17707900
H	0.38883200	-2.91639700	2.99150700	H	3.66588500	-0.43192600	-2.54383300
C	2.35897300	-3.44457900	1.13921800	H	5.33999300	0.94642700	-1.34535100
H	1.57578100	-4.21116800	1.14522700	C	-2.74429800	3.01182800	2.09220400
H	3.14772500	-3.76067700	1.83087500	H	-3.59231200	2.35920400	2.31189800
H	2.78787000	-3.40603000	0.13099900	H	-2.69194200	3.82318300	2.81737700
C	2.93908100	-1.04675000	1.53484400	H	-2.85211800	3.41079400	1.08050400
H	3.39596800	-0.98437000	0.54313600	O	-1.51125400	2.30401300	2.23226300

Table S22. Optimized Cartesian coordinates (Å) for **TS**$_{cyclic}$-*anti-α* in the singlet state at the ωB97XD/6-31G* level.

Ge	0.99322700	-1.35370000	-0.23802500	H	1.77478100	2.58212600	2.35910600
O	-0.75886500	-1.05846300	-1.06059600	H	2.95162000	3.88109600	2.09620900
O	-1.60794200	-0.16295700	2.47133600	H	3.50004100	2.20654700	2.30905900
O	-0.25581200	-1.53309900	1.28692600	C	-5.12601100	1.33898900	-0.67126200
C	1.32749100	0.42341900	0.41675400	H	-6.16145600	1.64527700	-0.55729500
C	-2.48012400	0.55535100	-0.96152700	C	-4.15317000	2.29128400	-0.97301900
C	-1.08889500	0.16234600	-1.07968400	H	-4.42967100	3.33373100	-1.09255700
H	-0.33826600	0.91469800	-1.33674800	C	-2.31313400	-1.33758200	2.86546400
C	-0.55841500	-0.33753300	1.66797200	H	-1.66049700	-2.01607000	3.42039900
C	2.36199900	1.17185100	0.00266500	H	-2.71763900	-1.86183100	1.99652100
H	3.07294800	0.69871500	-0.67278100	C	4.01107000	3.00433600	-0.26436300
C	0.14741600	0.75502600	1.19579300	H	4.81169300	2.37748500	0.14423200
H	-0.11322900	1.75070100	1.52738200	H	4.25523600	4.05027400	-0.04719200
C	2.65275900	2.62467700	0.34517200	H	4.00397800	2.87587400	-1.35289200
C	-2.82895300	1.90178800	-1.11508900	C	1.56764700	3.53087300	-0.26908300
H	-2.05825600	2.63479000	-1.33957600	H	1.52710700	3.40240300	-1.35678900
C	-3.45878600	-0.39922300	-0.65743900	H	1.78642000	4.58487500	-0.05969400
H	-3.16163300	-1.43641500	-0.54080900	H	0.57528700	3.30816000	0.13648100
C	-4.77989500	-0.00348300	-0.51305900	H	-3.12304300	-0.98513600	3.50366100
H	-5.54402600	-0.73747900	-0.27812600	Cl	1.74395600	-3.29862500	0.29038500
C	2.71864800	2.83414300	1.86806000	Cl	1.91771600	-1.34565900	-2.28845800

Table S23. Optimized Cartesian coordinates (Å) for **TS**$_{cyclic}$-*syn-γ* in the singlet state at the ωB97XD/6-31G* level.

Ge	1.39602200	-0.40144400	-0.71611600	C	-3.07131200	2.38707400	-1.37077100
Cl	2.61841900	0.12760700	-2.39386200	H	-2.68226300	2.65658600	-2.36009200
Cl	1.82343800	-2.48550900	-0.21706900	H	-3.85211000	3.10970700	-1.10835900
O	-0.18062200	-1.10476700	-1.77854000	H	-3.54206700	1.40025100	-1.44512100
O	2.59631400	0.07082000	0.74708500	C	-2.12713900	-1.29631800	-0.29425500
C	0.20712900	0.99621600	-0.03463700	C	-1.53780900	-1.68718300	0.91607100
C	-1.38088700	-0.85703600	-1.48171300	C	-3.52348700	-1.30736000	-0.40805000
C	-0.87484500	1.41157900	-0.76406800	C	-2.33761400	-2.05883000	1.98617600
H	-0.79898400	1.29414600	-1.84378600	H	-0.46214000	-1.69240600	1.04084200
C	0.71156700	1.39235400	1.21646100	C	-4.32116700	-1.68739400	0.66324000
H	0.30255500	2.18375700	1.82914700	H	-3.98591000	-1.01304400	-1.34705100
C	1.96622100	0.87341700	1.53402400	C	-3.72710100	-2.05774700	1.86622100
C	-1.95380500	2.38723000	-0.31593700	H	-1.87417000	-2.35095200	2.92297700
C	-2.56746900	1.99861500	1.04006900	H	-5.40157500	-1.69142200	0.56069100
H	-3.03856300	1.01231400	0.99368800	H	-4.34447000	-2.34878700	2.71050700
H	-3.33601800	2.72829600	1.32136000	H	-2.02205900	-0.53250400	-2.31246900
H	-1.82552800	1.97240800	1.84170100	O	2.56105100	1.24105300	2.65713600
C	-1.35755100	3.80661900	-0.23427500	C	3.87652000	0.73158800	2.89050400
H	-0.55317600	3.86192500	0.50555900	H	4.56259400	1.06492400	2.10855300
H	-2.12999800	4.53273300	0.04813600	H	4.17268900	1.13909300	3.85623900
H	-0.94152500	4.10970800	-1.20137800	H	3.86697900	-0.35998000	2.92061100

Table S24. Optimized Cartesian coordinates (Å) for **TS**$_{cyclic}$-*anti*-*γ* in the singlet state at the ωB97XD/6-31G* level.

Ge	-1.32495500	-0.93513300	0.21241400	H	0.30758100	3.06983100	-1.43215900
Cl	-1.85715600	-2.13877100	1.89549500	C	4.89021600	-0.31320800	-1.37268100
Cl	-1.60427100	-1.95193200	-1.69961700	H	5.48768100	0.08176600	-2.18805900
O	0.56628900	-1.56168000	0.23680200	C	5.50775700	-0.79028700	-0.21827100
O	-3.12692800	-0.16710100	-0.01238400	H	6.58987900	-0.76251800	-0.13378600
O	-4.04288500	1.79453400	-0.58651500	C	3.35984600	-1.34369000	0.72662600
C	-0.68026900	0.90006600	0.24165400	H	2.74645700	-1.74428700	1.52742900
C	1.28029900	-0.84740700	-0.54016100	C	0.65596800	3.55606900	1.33118400
H	0.86616300	-0.49580200	-1.49002700	H	-0.38660600	3.73204600	1.05509500
C	2.73663000	-0.84885000	-0.42362000	H	1.18692600	4.51380200	1.26782800
C	0.64239700	1.16016000	0.55857700	H	0.67171500	3.22806800	2.37628600
H	1.09720100	0.48949800	1.28019000	C	-5.29597600	1.10605600	-0.61662400
C	-1.73018500	1.71818400	-0.19108200	H	-5.53766500	0.70514900	0.37031100
H	-1.68210800	2.79041100	-0.32098100	H	-5.26789200	0.28982100	-1.34144700
C	-2.97550000	1.07986900	-0.27302800	C	2.79710100	2.37444100	0.89043500
C	1.33744200	2.50956200	0.42557100	H	2.84675300	2.00633800	1.92217600
C	3.50528000	-0.34189500	-1.47539100	H	3.29674900	3.34954000	0.85827400
H	3.01289700	0.03440600	-2.36855200	H	3.36432600	1.68546600	0.25896600
C	1.32415600	2.98238900	-1.03713600	C	4.74292700	-1.30864100	0.82740300
H	1.87055500	2.27599500	-1.67255200	H	5.23036000	-1.68509300	1.72117100
H	1.80851700	3.96161900	-1.12860600	H	-6.02700700	1.85720900	-0.91270800

5-4-4. Aldol reaction of [**2b**-Cl]$^{-}$ with **3a** in THF

Table S25. Optimized Cartesian coordinates (Å) for Cl^{-} in the singlet state at the ωB97XD/6-31G* level.

Cl	0.00000000	0.00000000	0.00000000

Table S26. Optimized Cartesian coordinates (Å) for [**2b**-Cl]$^{-}$ in the singlet state at the ωB97XD/6-31G* level.

C	0.52401600	1.71562000	0.00006600	C	3.61378400	1.14423300	-1.26164100
C	-0.80785400	2.01848700	0.00005400	H	3.50859500	0.52775300	-2.16130000
H	1.26404000	2.50394500	0.00011000	H	2.88133100	1.95293300	-1.33091500
C	0.82751700	0.30353300	0.00001900	H	4.61929700	1.58793800	-1.26299700
C	2.01008000	-0.33958900	0.00001300	O	-1.73184500	1.11266500	0.00000700
H	1.98878100	-1.42531800	-0.00000600	O	-1.21290400	3.31042800	0.00009200
C	3.39725200	0.28818100	0.00004300	Ge	-0.85997900	-0.66594300	-0.00003100
C	4.43804900	-0.84256100	0.00013300	Cl	-1.99749800	-1.04287800	1.86565100
H	4.32110300	-1.47859100	-0.88525300	Cl	-1.99745300	-1.04277700	-1.86576100
H	5.45897100	-0.43860100	0.00016200	C	-2.61455600	3.51274600	0.00007500
H	4.32102500	-1.47852100	0.88555900	H	-2.75375700	4.59657500	0.00011000
C	3.61367600	1.14433900	1.26167400	H	-3.08208500	3.07479600	-0.88712200
H	2.88121600	1.95304400	1.33081500	H	-3.08211400	3.07473400	0.88722600
H	3.50841000	0.52793400	2.16137600	Cl	-0.08672400	-2.87382500	-0.00008100
H	4.61918900	1.58804300	1.26307700				

Table S27. Optimized Cartesian coordinates (Å) for **TS**$_{\text{cyclic}}$-*syn*-*α* in the singlet state at the ωB97XD/6-31G* level.

O	0.49560400	-1.57423700	-1.20447100	H	-0.32300800	-2.92381900	0.08179300
O	2.01530400	-0.99275200	1.02400400	C	-1.86022600	-1.64501100	-0.81734600
C	-0.11416200	0.51778900	0.56589400	C	-2.91582600	-2.32849000	-0.20949700
C	-0.45468700	-2.00733900	-0.51998700	C	-2.12853800	-0.63694000	-1.74700300
C	0.96113600	-1.29077400	1.65728800	C	-4.23115800	-2.01681200	-0.52970900
C	-0.86750700	1.62030300	0.48512500	H	-2.69693300	-3.10589800	0.52034800
H	-0.60631200	2.33674600	-0.29185700	C	-3.44683700	-0.33325200	-2.07211100
C	-0.25444200	-0.65658600	1.39194500	H	-1.29532600	-0.11190100	-2.20446400
H	-1.13253100	-0.90716900	1.97241500	C	-4.49782500	-1.01972800	-1.46843600
C	-1.98667900	2.05310100	1.42615700	H	-5.04923400	-2.55101200	-0.05373500
C	-1.47455900	2.08201600	2.87707400	H	-3.65304800	0.44917100	-2.79722700
H	-1.16709900	1.08643300	3.20986700	H	-5.52594300	-0.77707700	-1.72466000
H	-2.25711300	2.44730400	3.55637700	C	2.23417800	-3.00631400	2.60451400
H	-0.60315200	2.74012400	2.96104600	H	3.02095500	-2.34313900	2.97475900
C	-2.41646500	3.47464300	1.03040600	H	2.07008700	-3.82710100	3.30522300
H	-1.57218700	4.17022700	1.10017000	H	2.53599300	-3.38997400	1.62653400
H	-3.21752800	3.83943100	1.68622200	O	0.98912200	-2.32289200	2.52162600
H	-2.78403900	3.49538300	-0.00250300	Ge	1.53849200	0.32561900	-0.49190400
C	-3.21271500	1.13034400	1.32120800	Cl	0.90895400	1.51585500	-2.38573400
H	-3.60712400	1.12119700	0.30081800	Cl	2.72356200	1.99517500	0.48808400
H	-4.00862100	1.47350500	1.99701800	Cl	3.28562800	-0.58405600	-1.55885700
H	-2.97040900	0.09654600	1.58100300				

Table S28. Optimized Cartesian coordinates (Å) for **TS**$_{\text{cyclic}}$-*anti*-*α* in the singlet state at the ωB97XD/6-31G* level.

Ge	0.98882100	-1.26099700	-0.10027600	H	2.63764500	4.28035700	1.70201800
O	-0.59230000	-0.52961400	-1.56118300	H	3.08901700	2.61822700	2.15129600
O	-1.92981500	0.54438500	2.07355200	C	-5.26024100	0.84885400	-0.41798400
O	-0.59359200	-1.04995300	1.21054600	H	-6.31855800	0.90689200	-0.17700600
C	1.14192400	0.68632800	0.16421800	C	-4.53581800	2.01565800	-0.65766400
C	-2.54711200	0.70047500	-1.03641100	H	-5.02742300	2.98319300	-0.60020500
C	-1.10073900	0.58961800	-1.34903600	C	-2.66997900	-0.51955300	2.65825600
H	-0.59921700	1.50919100	-1.68974800	H	-2.05697700	-1.06770800	3.37971900
C	-0.85794400	0.18115500	1.35299000	H	-3.02859600	-1.21508100	1.89720400
C	2.24786200	1.36450300	-0.16323700	C	3.97939200	3.12539700	-0.38377200
H	3.05090600	0.80112700	-0.63401500	H	4.68620400	2.52086100	0.19612500
C	-0.11947900	1.16321400	0.69225400	H	4.23655700	4.18333000	-0.24345000
H	-0.37597100	2.20367100	0.84626000	H	4.11719900	2.87645200	-1.44258400
C	2.53479800	2.84510100	0.05973700	C	1.59159500	3.71764800	-0.78998800
C	-3.18241200	1.94017700	-0.96501600	H	1.68609300	3.46181700	-1.85132000
H	-2.60556700	2.84596600	-1.14105700	H	1.83199300	4.78312100	-0.66946700
C	-3.27340500	-0.46851100	-0.79653500	H	0.54440100	3.57516500	-0.50576200
H	-2.74343200	-1.41579700	-0.84501300	H	-3.51417000	-0.04297700	3.15934300
C	-4.62647900	-0.39109400	-0.48837300	Cl	2.33276400	-1.96047700	1.58774800
H	-5.19050200	-1.30085700	-0.29991600	Cl	2.50527200	-1.53786700	-1.80508100
C	2.40138400	3.21861800	1.54626700	Cl	0.05215600	-3.26711100	-0.50504600
H	1.39043000	3.03361100	1.91931900				

Table S29. Optimized Cartesian coordinates (Å) for **TS**$_{cyclic}$-*syn*-γ in the singlet state at the ωB97XD/6-31G* level.

O	-0.05805600	0.00988300	-1.73155600
O	-1.65585200	1.65981400	0.14238000
C	-0.07429100	-0.37358900	0.87914700
C	1.07163000	-0.51953600	-1.49994900
C	0.86248200	-1.37439900	0.74419100
H	0.48701000	-2.29516300	0.30037300
C	-0.10491000	0.77382100	1.66057900
H	0.41875300	0.91464100	2.59789600
C	-1.01173800	1.75708800	1.22060900
C	2.08751700	-1.61174600	1.62533000
C	2.97396200	-0.38290600	1.88599700
H	3.44753300	-0.02148300	0.97153700
H	3.77073300	-0.65318100	2.59224700
H	2.41357000	0.44882600	2.31655300
C	1.57319800	-2.14474300	2.97934500
H	0.95980800	-1.39087200	3.48421400
H	2.40984200	-2.41145800	3.64107000
H	0.94975800	-3.03411700	2.83478300
C	2.95172700	-2.70137700	0.97083900
H	2.36773800	-3.61336100	0.79855300
H	3.80689700	-2.95943500	1.60897900
H	3.34192300	-2.36225700	0.00544000
C	2.24281300	0.35498400	-1.34862600
C	2.05151700	1.68205800	-0.94715600
C	3.53325600	-0.10612100	-1.62570800
C	3.14881600	2.51626400	-0.77515900
H	1.03671200	2.02454700	-0.77871200
C	4.62661800	0.73582900	-1.46819800
H	3.67342100	-1.13069300	-1.96236800
C	4.43654600	2.04612100	-1.02862800
H	3.00063600	3.54029600	-0.44368500
H	5.62794900	0.37261700	-1.68295000
H	5.29191800	2.70296100	-0.89457300
H	1.24484100	-1.56309300	-1.78838900
O	-1.18329500	2.84343500	1.98711100
C	-2.17024200	3.75841200	1.52494600
H	-3.13347200	3.25737900	1.40414800
H	-2.23097300	4.53382800	2.29056700
H	-1.88064700	4.19452600	0.56436200
Ge	-1.58188800	-0.36841800	-0.43913100
Cl	-1.58152500	-2.57761600	-1.15019300
Cl	-3.24769400	-0.74823200	1.11751900
Cl	-2.95897400	0.30636900	-2.07183600

Table S30. Optimized Cartesian coordinates (Å) for **TS**$_{cyclic}$-*anti*-γ in the singlet state at the ωB97XD/6-31G* level.

Ge	-1.24889100	-0.85194400	0.13576900
Cl	-1.24918200	-0.93108200	2.47141500
Cl	-1.24196800	-0.82192600	-2.30006300
O	0.67805300	-1.52499300	0.05538200
O	-3.03675200	-0.06468600	0.00378600
O	-3.99590300	1.92385000	-0.37265000
C	-0.56397500	1.00160200	0.14415400
C	1.44117200	-0.83001800	-0.66415700
H	1.07761800	-0.41699300	-1.60721300
C	2.89319400	-0.86410300	-0.42855400
C	0.76018900	1.27285800	0.41012700
H	1.24670700	0.58543600	1.09552600
C	-1.65462800	1.83768800	-0.15103700
H	-1.62528200	2.91648100	-0.23836900
C	-2.88784600	1.19515900	-0.18104900
C	1.43283700	2.63664200	0.30785600
C	3.76570700	-0.39524200	-1.41278400
H	3.35884000	-0.00499600	-2.34243600
C	1.31634300	3.19186900	-1.12069000
H	1.81388700	2.51964200	-1.82945700
H	1.78700100	4.18162800	-1.19661900
H	0.27264700	3.28168500	-1.43525300
C	5.13873600	-0.41741700	-1.19630200
H	5.81566400	-0.04995800	-1.96231500
C	5.64291000	-0.90546000	0.00748200
H	6.71547500	-0.91630000	0.18150500
C	3.40193500	-1.36879900	0.77155100
H	2.70112500	-1.73127800	1.51763100
C	0.81100200	3.62619800	1.31466100
H	-0.25390400	3.78376000	1.12677700
H	1.31647600	4.60122200	1.26501300
H	0.90955700	3.23956000	2.33515400
C	-5.21873800	1.19940900	-0.37831200
H	-5.39117800	0.71298900	0.58581500
H	-5.21633200	0.43585500	-1.16036800
C	2.92265200	2.49324300	0.65815000
H	3.04796000	2.05968800	1.65791600
H	3.41818700	3.47312200	0.64892800
H	3.44202300	1.84335200	-0.05206700
C	4.77295900	-1.38173600	0.98877800
H	5.16848600	-1.76270000	1.92605000
H	-5.99503700	1.94199000	-0.57218700
Cl	-2.01798400	-2.94444600	0.05217600

5-4-5. The structures of corresponding cyclic-*Si*-dienolates **2b′** and **[2b′-Cl]⁻**

Table S31. Optimized Cartesian coordinates (Å) for **2b′** in the singlet state at the ωB97XD/6-31G* level.

C	-0.03984200	1.52040200	0.00004500	H	4.11600000	2.25868600	0.00032000
C	-1.39209300	1.55247800	-0.00009900	C	3.88675100	-0.15035900	-1.25493200
H	0.52445300	2.44022100	0.00017400	H	3.95382800	-1.24404800	-1.28122000
C	0.52271900	0.16591300	0.00000700	H	3.37816800	0.17996900	-2.16687100
C	1.77381700	-0.33120500	0.00004300	H	4.90736900	0.25007000	-1.26547600
H	1.85964000	-1.42070900	0.00000200	Cl	-1.31898900	-2.05581400	-1.64967300
C	3.13404400	0.33337900	0.00012300	Cl	-1.31926100	-2.05569600	1.64951600
C	3.88668800	-0.15052400	1.25515100	O	-2.10006500	0.38421300	-0.00022000
H	3.95377200	-1.24421500	1.28129400	O	-2.11471600	2.66733400	-0.00017900
H	4.90730200	0.24991100	1.26580400	C	-3.53516200	2.55378500	0.00022100
H	3.37805500	0.17967700	2.16710900	H	-3.88864000	2.03063500	-0.89206900
C	3.09359300	1.86421800	0.00022300	H	-3.88815700	2.03090300	0.89286200
H	2.58807500	2.25225900	-0.88988400	H	-3.90102100	3.58011700	0.00016800
H	2.58794900	2.25213800	0.89031200	Si	-0.99224100	-0.89006000	-0.00009700

Table S32. Optimized Cartesian coordinates (Å) for [**2b′**-Cl]⁻ in the singlet state at the ωB97XD/6-31G* level.

C	-0.35328300	1.62683400	-0.00007700	C	-3.44111200	1.20499200	1.26258100
C	0.98541100	1.85296400	-0.00073600	H	-3.36622600	0.58282000	2.16140200
H	-1.05791200	2.44667200	0.00043600	H	-2.67287000	1.97904000	1.33556900
C	-0.68464700	0.21178900	-0.00001300	H	-4.42548200	1.69415100	1.26289200
C	-1.90777400	-0.35797700	0.00003600	O	1.83423800	0.86240700	-0.00065200
H	-1.95318100	-1.44255100	0.00012000	O	1.50194700	3.10256600	-0.00013600
C	-3.26263200	0.34087600	0.00004800	Si	0.95532300	-0.77337600	-0.00012100
C	-4.35970200	-0.73635400	-0.00006500	Cl	1.98776800	-1.18176000	-1.82121800
H	-4.27500600	-1.37752700	0.88536400	Cl	1.98845300	-1.18049100	1.82091000
H	-5.35936800	-0.28200300	-0.00010700	C	2.91541300	3.18927300	0.00024000
H	-4.27489400	-1.37744500	-0.88554400	H	3.14213800	4.25830500	0.00078200
C	-3.44104500	1.20517400	-1.26236400	H	3.34587700	2.71557400	0.88789100
H	-2.67281900	1.97924900	-1.33519500	H	3.34626100	2.71638200	-0.88765000
H	-3.36609100	0.58313500	-2.16127200	Cl	0.09714500	-2.83116500	0.00078200
H	-4.42542900	1.69430000	-1.26265500				

6. References

[1] O. V. Dolomanov, L. J. Bourhis, R. J. Gildea, J. A. K. Howard, H. Puschmann, "OLEX2 : a complete structure solution, refinement and analysis program" *J. Appl. Cryst.* **2009**, *42*, 339–341.

[2] R. W. Lang, H. Hansen, "Eine einfache Allencarbonsäureester-Synthese mittels der Wittig -Reaktion" *Helv. Chim. Acta* **1980**, *63*, 438–455.

[3] G. Himbert, D. Fink, "Cycloadditionen. 26. Einfluß von Substituenten in der allenischen ω-Position auf das Thermische Verhalten von Allencarbonsäurearylester" *J. Prakt. Chem.* **1997**, *339*, 233–242.

[4] A. S. K. Hashmi, R. Döpp, C. Lothschütz, M. Rudolph, D. Riedel, F. Rominger, "Scope and Limitations of Palladium-Catalyzed Cross-Coupling Reactions with Organogold Compounds" *Adv. Synth. Catal.* **2010**, *352*, 1307–1314.

[5] Y. Kita, Y. Tsuzuki, S. Kitagaki, S. Akai, "Chemistry of Silylketenes: Simple Preparation of Silylallenes by the Reaction of Silylketenes with Phosphorus Ylides." *Chem. Pharm. Bull.* **1994**, *42*, 233–236.

[6] G. E. Keck, R. L. Giles, V. J. Cee, C. A. Wager, T. Yu, M. B. Kraft, "Total Synthesis of Epothilones B and D: Stannane Equivalents for β-Keto Ester Dianions" *J. Org. Chem.* **2008**, *73*, 9675–9691.

[7] P. Selig, W. Raven, "A Convenient Allenoate-Based Synthesis of 2-Quinolin-2-yl Malonates and β-Ketoesters" *Org. Lett.* **2014**, *16*, 5192–5195.

[8] R. W. Lang, H. Hansen, "α-Allenic Esters from α-Phosphoranylidene Esters and Acid Chlorides: Ethyl 2,3-Pentadienoate" *Org. Synth.* **1984**, *62*, 202.

[9] M. J. Frisch, G. W. Trucks, H. B. Schlegel, G. E. Scuseria, M. A. Robb, J. R. Cheeseman, G. Scalmani, V. Barone, G. A. Petersson, H. Nakatsuji, X. Li, M. Caricato, A. V. Marenich, J. Bloino, B. G. Janesko, R. Gomperts, B. Mennucci, H. P. Hratchian, J. V. Ortiz, A. F. Izmaylov, J. L. Sonnenberg, D. Williams-Young, F. Ding, F. Lipparini, F. Egidi, J. Goings, B. Peng, A. Petrone, T. Henderson, D. Ranasinghe, V. G. Zakrzewski, J. Gao, N. Rega, G. Zheng, W. Liang, M. Hada, M. Ehara, K. Toyota, R. Fukuda, J. Hasegawa, M. Ishida, T. Nakajima, Y. Honda, O. Kitao, H. Nakai, T. Vreven, K. Throssell, J. A. Montgomery Jr., J. E. Peralta, F. Ogliaro, M. J. Bearpark, J. J. Heyd, E. N. Brothers, K. N. Kudin, V. N. Staroverov, T. A. Keith, R. Kobayashi, J. Normand, K. Raghavachari, A. P. Rendell, J. C. Burant, S. S. Iyengar, J. Tomasi, M. Cossi, J. M. Millam, M. Klene, C. Adamo, R. Cammi, J. W. Ochterski, R. L. Martin, K. Morokuma, O. Farkas, J. B. Foresman, D. J. Fox, *Gaussian 16, Revision C.01*, Gaussian, Inc., Wallingford CT, **2016**.

[10] A. E. Reed, L. A. Curtiss, F. Weinhold, "Intermolecular interactions from a natural bond orbital, donor-acceptor viewpoint" *Chem. Rev.* **1988**, *88*, 899–926.

[11] E. D. Glendening, C. R. Landis, F. Weinhold, "Natural bond orbital methods" *WIREs Comput. Mol. Sci.* **2012**, *2*, 1–42.
